# Transcriptional Profiles of Cell Fate Transitions Reveal Early Drivers of Neuronal Apoptosis and Survival

**DOI:** 10.3390/cells10113238

**Published:** 2021-11-19

**Authors:** Giovanna Morello, Ambra Villari, Antonio Gianmaria Spampinato, Valentina La Cognata, Maria Guarnaccia, Giulia Gentile, Maria Teresa Ciotti, Pietro Calissano, Velia D’Agata, Cinzia Severini, Sebastiano Cavallaro

**Affiliations:** 1 Institute for Biomedical Research and Innovation, National Research Council (IRIB-CNR), Via Paolo Gaifami, 18, 95125 Catania, Italy; giovanna.morello@irib.cnr.it (G.M.); ambra.villari@gmail.com (A.V.); antogian.spampinato@gmail.com (A.G.S.); valentina.lacognata@cnr.it (V.L.C.); maria.guarnaccia@cnr.it (M.G.); giulia.gentile@cnr.it (G.G.); 2 Institute of Biochemistry and Cell Biology, National Research Council (IBBC-CNR), Via E. Ramarini, 32, Monterotondo Scalo, 00015 Rome, Italy; mariateresa.ciotti@cnr.it (M.T.C.); cinzia.severini@cnr.it (C.S.); 3 European Brain Research Institute (EBRI Foundation), Viale Regina Elena, 295, 00161 Rome, Italy; p.calissano@ebri.it; 4 Department of Biomedical and Biotechnological Sciences, Section of Human Anatomy and Histology, University of Catania, Via Santa Sofia, 87, 95123 Catania, Italy; vdagata@unict.it

**Keywords:** apoptosis, neurotrophic factors, survival, transcriptional analysis, drug targets, drug repurposing, functional enrichment, regulatory network, disease

## Abstract

Neuronal apoptosis and survival are regulated at the transcriptional level. To identify key genes and upstream regulators primarily responsible for these processes, we overlayed the temporal transcriptome of cerebellar granule neurons following induction of apoptosis and their rescue by three different neurotrophic factors. We identified a core set of 175 genes showing opposite expression trends at the intersection of apoptosis and survival. Their functional annotations and expression signatures significantly correlated to neurological, psychiatric and oncological disorders. Transcription regulatory network analysis revealed the action of nine upstream transcription factors, converging pro-apoptosis and pro-survival-inducing signals in a highly interconnected functionally and temporally ordered manner. Five of these transcription factors are potential drug targets. Transcriptome-based computational drug repurposing produced a list of drug candidates that may revert the apoptotic core set signature. Besides elucidating early drivers of neuronal apoptosis and survival, our systems biology-based perspective paves the way to innovative pharmacology focused on upstream targets and regulatory networks.

## 1. Introduction

Neuronal apoptosis and survival are regulated by cell fate decision processes that ensure the correct development of the central nervous system and its homeostasis throughout adulthood. The ability of neuronal cells to promote or evade apoptotic cell death is regulated by different and interrelated transcriptional, post-transcriptional and post-translational layers [1,2,3]. A shift in the fine balance between opposite pro-apoptotic and pro-survival signals can have drastic consequences for the fate of a neuron and contribute to acute and chronic neurological disorders [4,5,6,7]. Thus, characterizing the key molecular events underlying neuronal apoptosis and survival may help to devise strategies aimed at counteracting neurodegeneration.

In recent years, remarkable progress has been made to reveal the systems biology of neuronal survival and death. The advent of high-throughput technologies and powerful bioinformatic analysis is unveiling the signaling networks underlying neuronal apoptosis and survival in numerous in vitro and in vivo paradigms [8,9,10,11,12,13,14,15]. Among these, cerebellar granule neurons (CGNs) represent a model of election for examining the signal transduction mechanisms underlying neuronal apoptosis. In vitro, CGNs undergo rapid apoptotic cell death within 24 h after removal of serum and lowering of extracellular K^+^ concentration from 25 to 5 mM [16,17]. Apoptosis of CGNs requires transcription and protein synthesis, becoming irreversible after the first 6 h following its induction. Before this commitment point, apoptosis of CGNs can be rescued by the activation of specific signal transduction pathways or by the treatment with specific growth factors (GFs) [16,17,18]. We have previously detailed the transcriptional changes associated with CGNs apoptosis and its rescue by different GFs [19,20,21,22,23,24]. These studies, performed at the end of the commitment phase, were the first to investigate the transcriptional program underlying neuronal apoptosis and, lately, proposed the existence of a common transcriptional program governing neuronal survival. Indeed, although mediated by specific receptors and intracellular second messengers, the survival effects of Pituitary adenylate cyclase-activating polypeptide (Pacap) and insulin-like growth factor-1 (Igf1) were propagated by common transcriptional cascades [21]. Although these findings represent the first glimpse of how a neuron orchestrates its destruction/survival, the dynamic spectrum of physical and biological elements (hardware) that execute these processes is mostly unknown. Most important, the instruction set (software) that tells the hardware how to implement neuronal apoptosis or survival is still unexplored.

In this work, we provide a systems biology-based perspective of the earliest molecular events controlling neuronal fate decisions. Overlaying the temporal transcriptional profiles of CGNs during the early commitment phase of apoptosis or their rescue by three different GFs (Pacap, Igf1 and substance P, SP), we identified a *core set* of genes at the intersection of apoptosis and survival. Their subsequent promoter motif analysis revealed the upstream regulators, converging neuronal apoptosis and survival-inducing signals in a highly interconnected and temporally ordered manner. Genes in the apoptosis/survival core set are significantly correlated to neurological, psychiatric and oncological disorders, and encode for therapeutical targets whose modulation might exert anti-apoptotic or pro-survival effects.

## 2. Materials and Methods

### 2.1. Experimental Design

Animals were subjected to experimental protocols approved by the Local Animal Welfare Committee and the Veterinary Department of the Italian Ministry of Health (Aut. 527/2017-PR), and experiments were conducted according to the ethical and safety rules and guidelines for the use of animals in biomedical research provided by the relevant Italian laws and European Union’s directives (Italian Legislative Decree 26/2014 and 2010/63/EU). Primary cultures of CGNs were prepared from 8-day-old Wistar rats (Charles River, Calco, Italy) and were cultured as previously described [25]. In brief, cerebella were sliced, and tissue was dissociated through trypsinization in 0.025% trypsin solution (15 min at 37 °C) and trituration in presence of DNase (0.01%) and trypsin inhibitor (0.05%). Dissociated cells were collected through centrifugation and resuspended in basal Eagle’s medium (BME) supplemented with 10% fetal calf serum, 25 mm KCl, 2 mm glutamine and 100 μg/mL gentamycin. Cytosine arabinofuranoside (10 μM) was added after 18 h of culture to inhibit the growth of non-neuronal cells. After 6 days in vitro, extracellular KCl was shifted from 25 to 5 mm for neuronal apoptotic death induction. After two washes with serum-free BME containing 5 mm KCl, neurons were incubated with the same medium to induce apoptosis (K5), whereas control neurons were incubated with a serum-free medium supplemented with 25 mm KCl (K25). To investigate the rescue effects of GFs, K5 neurons were treated with a maximal effective dose of SP (200 nM), Pacap (100 nM) or Igf1 (3.26 pM). As previously demonstrated [18,20,21], CGNs undergo apoptotic cell death after the removal of serum and lowering of extracellular potassium from 25 to 5 mM, and can be rescued by treatment with SP, Pacap or Igf1 (Figure S1) [18,20,21]. All the substances were obtained from Sigma-Aldrich (Milano, Italy), unless otherwise specified.

### 2.2. RNA Isolation and Microarray Hybridization

Microarray experiments were performed in CGNs following incubation for different times (0.5, 1 and 3 h) with 25 mM KCl (K25), or 5 mM KCl (K5) in the absence or presence of a maximal effective dose of SP (200 nM), Pacap (100 nM) or Igf1 (3.26 pM). Total RNA was extracted with Trizol (Life Technologies, Monza, Italy) from a total of 60 samples, including four biological replicates for each experimental condition (K25, K5, K5 + SP, K5 + Pacap, K5 + Igf1) and time (0.5, 1 and 3 h). RNA quantity and quality were assessed using a NanoDrop 1000 Spectrophotometer (Thermo Fisher Scientific, Waltham, MA, USA) and a 2100 Bioanalyzer microfluidic electrophoresis platform (Agilent Technologies, Palo Alto, CA, USA), respectively, according to the manufacturer’s instructions. Complementary RNAs (cRNAs) labeled with Cy3-CTP were synthesized from 1 μg of total RNA of each sample using the Low RNA Input Fluorescent Linear Amplification Kit (Agilent Technologies, Palo Alto, CA, USA), following the manufacturer’s protocol.

Aliquots (750 ng) of Cy3-labeled cRNA targets were hybridized on arrays using the SurePrint G3 Rat Gene Expression 8 × 60 K microarray kit (Agilent Technologies, Palo Alto, CA, USA). Microarray hybridization and washing were performed using reagents and instruments (hybridization chambers and rotating oven) as indicated by the manufacturer (Agilent Technologies, Palo Alto, CA, USA). Arrays were then scanned at 3 µm resolution using an Agilent G4900DA SureScan Microarray Scanner System (Agilent Technologies, Palo Alto, CA, USA). Raw microarray data were acquired and analyzed using Agilent’s Feature Extraction v.12.1 software to assess the array spot quality, as well as to check signal and background intensity statistics in the default setting. Raw signal values were thresholded to 1, log2 transformed, normalized to the 75th percentile and baselined to the median of all samples using GeneSpringGX v.14.9 (Agilent Technologies, Palo Alto, CA, USA). Microarray data were deposited in NCBI’s Gene Expression Omnibus (GEO) with the accession number GSE179383.

### 2.3. Time-Point Differential Gene Expression Analysis

To identify genes with significant temporal expression changes and evaluate trend differences in CGNs induced to apoptosis (K5 vs. K25) or rescued by GFs (SP, Pacap or Igf1 treatment vs. K5), pre-processed normalized array data were analyzed by using the R package maSigPro (microarray Significant Profiles) [26]. MasigPro is a statistical procedure specifically designed for the analysis of transcriptomic time-course that provides information on genes that change over time and with respect to the control. This method is based on a two-step regression approach where experimental groups are defined by dummy variables: the first step uses a generic polynomial model to identify DEGs, whereas the second applies stepwise regression to study differences between groups and reveals the patterns of significant differential time profiles. Genes whose expression levels varied in a statistically significant way along time were detected using the linear step-up Benjamini-Hochberg false discovery rate (FDR) procedure, setting a corrected *p*-value ≤ 0.05. This analysis was performed for each GF individually. Intersecting probes (genes) were then selected through Venn diagrams and genes with opposite expression trends between apoptosis and GF-mediated rescue were selected for each time point for subsequent analyses.

### 2.4. Protein–Protein Interaction (PPI) Network Functional Enrichment Analysis

In order to investigate the biological significance for gene expression dynamics of GF-induced rescue from apoptosis over time, integrated networks of gene-encoded proteins were analyzed by STRING (version 11.0, http://string-db.org, accessed on 7 April 2021) [27]. The list of genes with opposite expression trend between apoptosis and GF-mediated rescue at each of the time points were used as input gene set, and protein–protein interactions (PPIs) were analyzed for experimentally validated interactions with a reliability threshold of a combined score of >0.4 as the setting for significant interaction pair. The interaction network was visualized by Cytoscape (version 3.8.2, http://www.cytoscape.org, accessed on 7 April 2021), and Cytoscape’s Network Analyzer plug-in was used to analyze the topological properties of nodes in the PPI network [28]. In particular, we generated a general PPI network including the 175 *core set* genes and, to characterize their time-dependent changes, the temporal PPI networks for *core set* genes differentially expressed at each time point (0.5, 1 and 3 h) were constructed. PPI networks in Cytoscape are visualized as graphs with “circles/nodes” indicating genes/proteins and “edges” depicting associated interactions between them. The number of edges that are connected to a node is the degree. Proteins with degree connectivity >50 were identified as hub proteins and represent the most significant nodes in the PPI network. To facilitate the visual identification of network hubs, node size and color were set based on their connectivity degree, where size and color of each node (in PPI network) were proportional to its node degree: larger circles indicate a higher degree, and as the circles become bigger and their color changes from light to dark, the value of the connectivity degree of the gene node increases.

Subsequently, biologically relevant subsets of network-related genes were selected from the entire set of genes by using the Molecular Complex Detection Algorithm (MCODE) plugin in Cytoscape, which is an app for Cytoscape that is used to cluster a given network to a densely connected area based on topology. The following cutoff parameters were used: Degree cutoff = 2, node score cutoff = 0.2, Haircut = true, Fluff = false, k-core = 2 and max depth = 100. Clustering modules having high node scores and connectivity degrees were considered as biologically significant clusters and were analyzed for functional enrichment with Gene Ontology (GO) terms and KEGG or Reactome pathways using the STRING app on Cytoscape. All genes in the genome were used as enrichment background. The significance of enriched pathways and P values was calculated based on the cumulative hypergeometric t-test, and false discovery rate (FDR) was used for multiple correction testing. Only terms with a corrected *p*-value < 0.05 were selected.

### 2.5. Upstream Transcriptional Regulator Analysis

To investigate common transcriptional regulators that can explain the observed gene expression changes in the *core set* genes, the iRegulon plugin (version 1.3) in Cytoscape, was used to predict potential upstream TFs regulating gene expression of *core set* genes [29]. The goal of iRegulon is to reverse engineer the transcriptional regulatory network underlying a co-expressed gene set using cis-regulatory sequence analysis. iRegulon implements a genome-wide ranking-and-recovery approach to detect enriched transcription factor motifs and their optimal sets of direct targets using the position weight matrix method. iRegulon uses >9000 known position weight matrices from various sources and different species and links them to candidate binding TFs using a “motif2TF” procedure. In particular, iRegulon detects the TFs and their targets by scanning known TF-binding promoter motifs as well as the predicted motifs discovered from the integrated databases TRANSFAC, JASPAR, the Encyclopedia of DNA Elements (ENCODE) Project chromatin immunoprecipitation-sequencing data (https://genome.ucsc.edu/ENCODE, accessed on 12 May 2021), SwissRegulon (http://swissregulon.unibas.ch/sr/, accessed on 12 May 2021), and HOMER (http://homer.ucsd.edu/homer/motif/motifDatabase.html, accessed on 12 May 2021). Motif enrichment analysis used several position weight matrices to sort and score for each motif. The preferred motif was used for predicting final TFs. The criteria set for TF binding motifs enrichment analysis were as follows: identity between orthologous genes ≥ 0, FDR on motif similarity ≤ 0.001, and TF motifs with a normalized enrichment score (NES) > 3. Predicted upstream TFs were rated and grouped according to the NES, and TFs with the highest NES were selected to construct TF-target networks. The ranking option for motif collection was set to 10K (9713 PWMs), and a putative regulatory search space of 20 kb centered around the Transcription Start Site (TSS, 7 species) was selected for the analysis. We executed iRegulon and looked for TFs for down- and upregulated *core set* genes, separately. Finally, the obtained transcription regulation relationships were merged into the TF-*core set* regulatory network, and the integrated network was visualized by Cytoscape software. Network analysis on significant differentially expressed *core set* genes (at each time point) was performed on the Cytoscape platform, integrating multiple apps/plug-ins such as Network Analyzer and STRING enrichment, using the default parameters described above.

### 2.6. Disease Biomarker Enrichment and Selection of Potential Drug Targets

To investigate the potential clinical implication of the *core set* genes, we firstly performed a gene/biomarker-disease association analysis by using DAVID online tool (version 6.8, https://david.ncifcrf.gov, accessed on 2 July 2021). The disease-associated genes were obtained from multiple databases, including the Genetic Association Database (GAD), Online Mendelian Inheritance in Man (OMIM), and Clinvar. DAVID calculates the *p*-value by hypergeometric distribution to evaluate the statistical significance of the enriched diseases (biomarkers) and uses FDR adjustment for multiple test correction. Statistically significant overrepresented disease (biomarker) terms were selected with a significance level *p* < 0.05.

Then, we searched for disease-specific gene expression signatures showing a positive correlation with our apoptotic-related gene set using the integrative NIH Library of Integrated Network-Based Cellular Signatures (iLINCS, http://ilincs.org, accessed on 12 July 2021), an integrative web-based platform for analysis of omics data and signatures of cellular perturbations [30]. This platform performs analysis of user-submitted omics signatures of diseases and cellular perturbations in the context of a large compendium of pre-computed signatures (>200,000), allowing data mining and re-analysis of a large collection of omics datasets (>12,000), pre-computed signatures and their connections [30]. Using iLINCS, users can search for signatures specific to pharmacological action, mechanism of action, or genetic or proteomic target. Users can also search available datasets for specific signatures of interest. Connected signatures can be further analyzed in terms of changes in gene/protein expression patterns that underlie the connectivity with the query signature, or through the analysis of gene/protein targets of connected perturbagens. In particular, the “Disease-Related Signatures” consisted of 9000 transcriptional signatures constructed by comparing sample groups within the collection of curated public domain transcriptional dataset (i.e., GEO42, EBI Expression Atlas). Each signature consists of differential expressions and associated p-values for all genes calculated using the Empirical Bayes linear model implemented in the limma package.

The iLINCS cloud was also used to identify repurposing drugs that could potentially reverse apoptosis, based on the extracted gene expression signatures. iLINCS provides over 40,000 transcriptomic profiles (signatures) of cell lines following treatment with chemical perturbagens such as FDA-approved drugs, chemical probes and screening library compounds including those with clinical utility and known mechanisms of action. To identify small drug-like molecules with inverse signatures of our *core gene* signatures, we probed iLINCS for small molecule perturbagens that result in L1000 transcriptomic signatures being highly discordant (anti-correlated as denoted by negative concordance values) with the expression of our apoptotic *core gene* set [30]. Only drug candidates with a negative connectivity value ≤ −0.321 (from at least one data source) between the reference gene sets and the small-molecule signature are displayed, excluding non-drug small molecules. For cmap candidates, we only included those with a *p*-value < 0.05. Drugs were statistically associated with the disease using the hypergeometric probability test and the top 50 signatures were considered. Finally, the ClinVar, Orphanet, DrugBank and Therapeutic Target Database (TTD) online tools were used to investigate if previously identified TFs targeting *core set* genes encode for drug targets whose modulation might exert anti-apoptotic or pro-survival effects. In particular, we focused on the encoded proteins that are primary targets of drugs approved by the US Food and Drug Administration (FDA) or in preclinical/clinical trials for the treatment of neurological disorders.

## 3. Results

### 3.1. Identification of a Converging Set of Apoptosis and Survival-Related Genes

**Figure 1:**
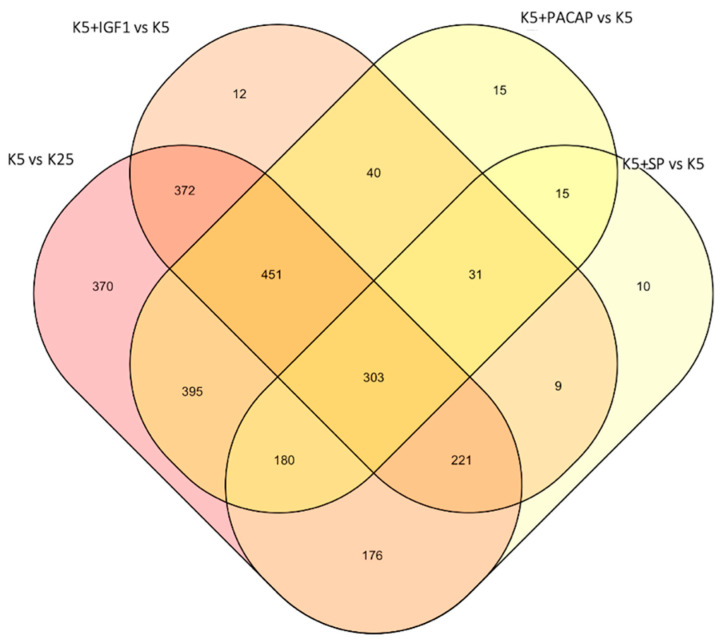
Venn diagram showing the number of genes differentially expressed in CGNs over time following induction of apoptosis (K5 vs. K25) or following rescue by SP, Pacap and Igf1. Of note, 262 genes (303 probes) were differentially expressed in all experimental conditions.

**Figure 2:**
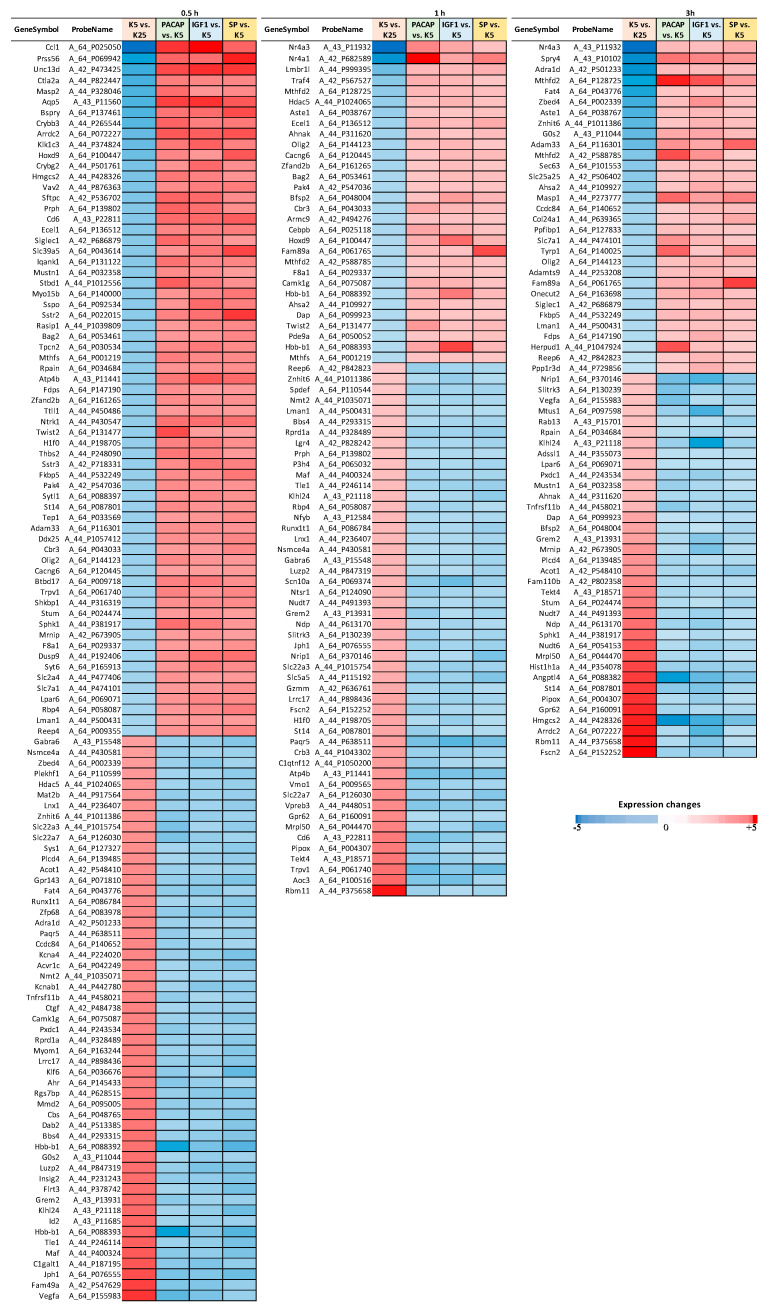
Heat maps showing the 175 *core set* genes, which were deregulated in CGNs during apoptosis or rescue by GFs and showed opposite expression in at least one-time point. Among them, 117 genes showed an opposite expression pattern at 0.5 h, 78 genes at 1 h and 67 genes at 3 h. Fold changes are shown by colors.

We performed a time-series whole-genome microarray analysis to investigate dynamic transcriptomic changes and capture temporal molecular events during the early commitment phase (0.5 h, 1 h and 3 h) of CGN apoptosis and its rescue by a maximal effective dose of Igf1(3.26 pM), Pacap (100 nM) and SP (200 nM) (Figure S1). Differentially expressed genes (DEGs) were identified using the R package MasigPro according to details described in Section 2. The numbers of temporal DEGs and related pairwise comparisons of different experimental conditions (apoptosis vs. control, GF-rescued vs. apoptosis) are summarized in Figure 1. The full gene lists with statistics and fold-change data at each time point are available in Tables S1–S4. In particular, when temporal gene expression profiles of control CGNs (cultured in 25 mM KCl) were compared with those induced to apoptosis (cultured in 5 mM KCl), 2054 genes referred to as “*apoptotic related genes*” (*ARGs*) showed significant temporal expression changes (Table S1). By comparing gene expression profiles in CGNs after the induction of apoptosis with those rescued by Pacap, Igf1 and SP treatment, we found 1185, 1261 and 820 deregulated genes (here referred to as “*survival-related genes*”, *SRGs*), respectively (Tables S2–S4). A total of 262 genes were found at the intersection of the four experimental conditions (*ARGs* and *SRGs*, Figure 1; Table S5). Of these, 175 genes, here defined as the “*core set*”, showed opposite expression trends between apoptosis and GF-mediated rescue in at least one-time point (Table S6). A comprehensive picture of their expression pattern at different time points is shown in Figure 2. In particular, the expression of 117 *core set* genes was significantly deregulated at 0.5 h, 78 genes at 1 h and 67 genes at 3 h (Figure 2).

### 3.2. Temporal PPI Network Analysis Identified Distinct Functional Clusters of Proteins Promoting Neuronal Survival

**Figure 3:**
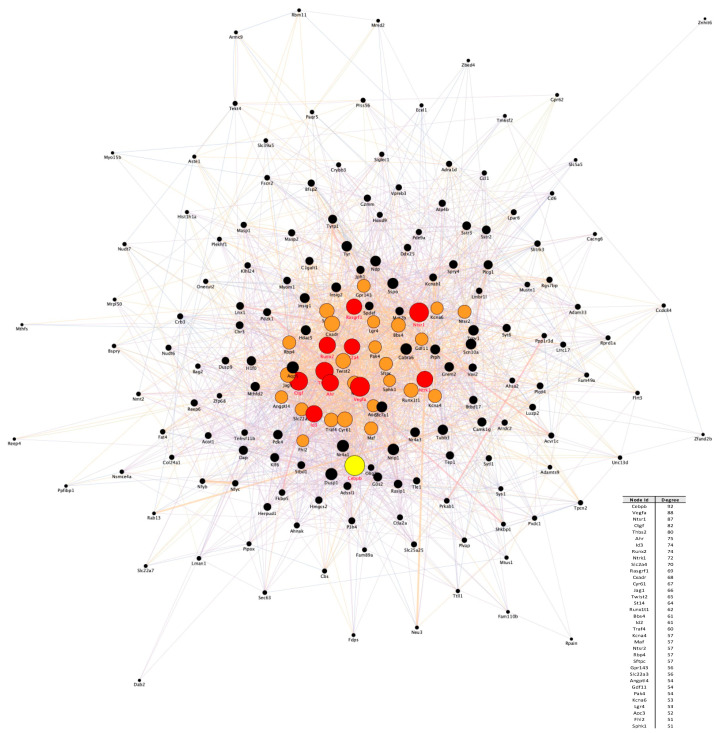
PPI network analysis. The PPI network for the 175 *core set* genes containing 187 nodes and 2668 interactions was constructed using the STRING website and visualized by Cytoscape (version: 3.8.2,) by mapping the “degree parameter” to node size. As the node size increased, the value of the connectivity degree of node genes increased. Proteins with a degree connectivity of >50 represent the most significant nodes in the PPI network and are colored from orange to red based on their node degree. Cebpb is the most interconnected node (hub) in the network and is colored in yellow. Differently colored “edges” indicate the type of evidence supporting each interaction: dark purple, co-expression; light purple, physical interaction; light blue, co-localization; light green, shared protein domain; orange, predicted; grey, other.

To explore the relationships among the *core set* genes and provide a global view of the network architecture that models the neuronal apoptotic/survival process, a PPI network analysis was conducted by using the STRING online database. The global molecular network constructed for the 175 *core set* genes had 187 nodes and 2668 interactions, at a combined score > 0.4 (Figure 3). The top five proteins with relatively high connectivity degrees within the network were: Cebpb (degree = 92), Vegfa (degree = 88), Ntsr1 (degree = 87), Ctgf (degree = 82) and Thbs2 (degree = 80) (Figure 3).

**Figure 4:**
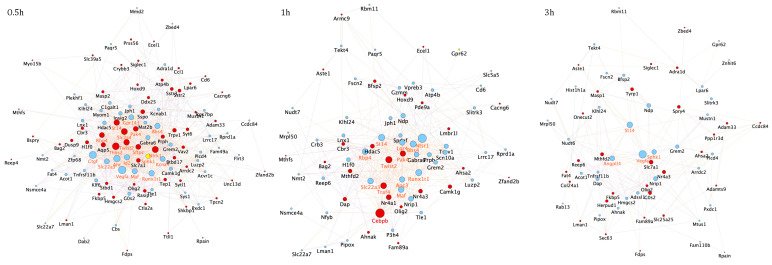
Temporal specific *core set* genes-related PPI networks. The three time-point (0.5, 1 and 3 h) specific *core set* genes-related PPI networks were constructed using the STRING website and visualized by Cytoscape (version: 3.8.2,) by mapping the “degree parameter” to node size. As the node size increased, the value of the connectivity degree of node genes increased. Light blue/red nodes indicate, respectively, down-/upregulated genes following treatment with all GFs compared with apoptotic CGNs (K5). Differently colored “edges” indicate the type of evidence supporting each interaction: dark purple, co-expression; light purple, physical interaction; light blue, co-localization; light green, shared protein domain; orange, predicted; grey, other.

To better characterize the time-dependent correlations between *core set* genes and assess their dynamic functional changes at various stages of the neuronal apoptotic/survival program, we constructed three interactome maps for up- or downregulated *core set* genes at each time point (0.5, 1 and 3 h) (Figure 4). Network analysis revealed Vegfa as the most highly interacting (hub) protein encoded by downregulated transcripts after 0.5 and 3 h following GFs treatment, whereas Cebpb was the most interacting protein encoded by up-regulated transcripts at 1 h (Figure 4). As expected, the number of nodes within each temporal PPI network and their physical interactions decreases over time, suggesting that the transcriptional activation of the majority of *core set* genes during the first hour after the induction of apoptosis and its rescue by GFs is required for the execution of these processes (Figure 4). Of note, in accordance with the assumption that many early response genes are dynamically regulated during neuronal activity or following neuronal insults, we found that the most interconnected *core set* genes activated during the early phases of the apoptotic/survival process encode transcription factors that in turn may coordinately regulate the transcriptional activation/repression of secondary response genes (Figure 4).

**Figure 5:**
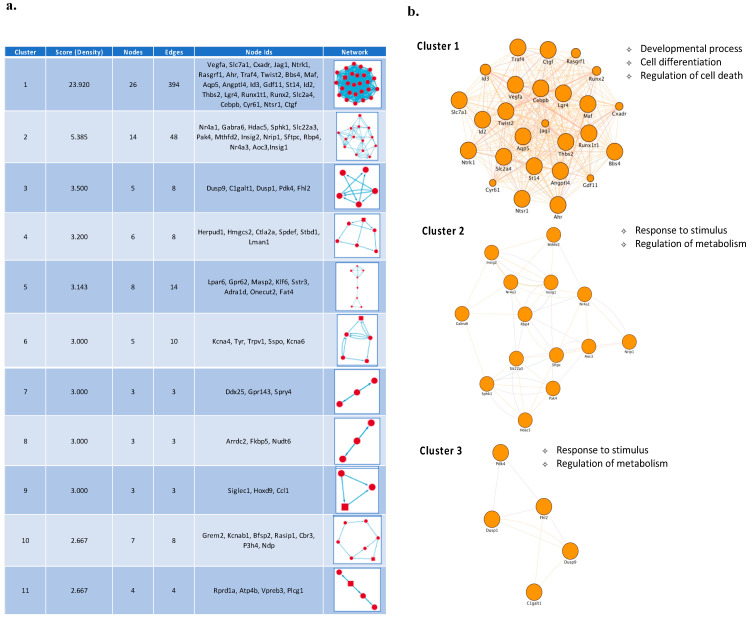
PPI network cluster analysis. (**a**) Sub-network analysis in the PPI network using MCODE identified 11 significant modules/clusters Cluster analysis in the PPI network resulted in 7 clusters, which include 72 nodes and 276 edges, and are enriched for several biological process GO terms. (**b**) Cluster 1, Cluster 2 and Cluster 3 of the top three network clusters in the sub-network analysis of PPI networks of core set genes. These clusters had the highest scores among the clusters. The cluster networks were visualized by Cytoscape by mapping the “degree parameter” to node size.

To further prioritize the leading candidate genes involved in CGNs apoptosis and survival we performed a subnetwork analysis of the general PPI network to identify significant modules/clusters. Cluster analysis of the PPI network resulted in 11 clusters that included 72 nodes and 276 edges (Figure 5a). The top 3 clusters are shown in Figure 5b. Cluster 1 exhibited the highest score and comprised 26 genes, among which are Cebpb, Vegfa, Id2, Maf, Ntrk1, Ahr, Traf4, Twist2, Thbs2 and Ntsr1 (Figure 5b). Biological processes enriched in Cluster 1 genes were associated with developmental processes, cell differentiation and regulation of cell death (Figure 5b). Genes in cluster 2 included Nr4a1, Nr4a3, Hdac5, Sphk1, Nrip1 and Rbp4, whereas genes in cluster 3 comprised Dusp9 and C1galt1. The main terms enriched for genes in both these modules were response to stimulus and metabolic process (Figure 5b). Given these clusters originated from the overlapping *core set* genes following the action of three GFs, genes involved in these clusters/biological processes may represent key mediators of a common neuronal survival program.

### 3.3. Transcription Regulatory Network Analysis Identified a Restricted Number of Master Regulators of Neuronal Apoptosis and Survival

**Figure 6:**
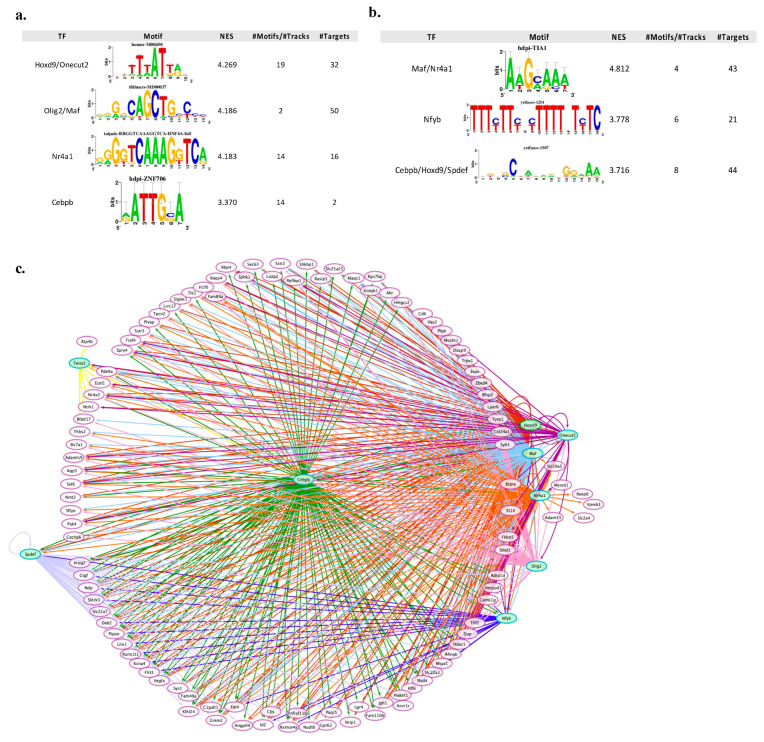
The upstream regulatory network is predicted to regulate the expression of the survival-related gene signatures in CGNs. (**a**) Result summary of the regulatory analysis with iRegulon on up-regulated *core set* genes. (**b**) Result summary of the regulatory analysis with iRegulon on downregulated *core set* genes. In particular, the top transcription binding motifs and their associated transcription factors (filtered for TF differentially expressed in our analysis) are shown. (**c**) The whole overview of the regulatory network of 9 key TFs together with their *core set* candidate targets. The network was visualized by *Cytoscape*. Targets are in white circle nodes with purple borders and TF in green hexagon nodes. Regulons for each TF are represented by different edge colors.

To identify candidate upstream regulators potentially involved in the time-dependent transcriptional regulation of neuronal apoptosis and survival, we performed an in silico analysis to predict transcription factors (TFs) whose binding sites are enriched in the promoter regions of *core set* genes using the iRegulon software. Our analysis yielded 118 significantly enriched motifs (66 for upregulated and 52 for downregulated genes) that clustered into 14 groups by similarity, with more than 100 transcription factors predicted to potentially bind the motifs present in the *core set* genes (Tables S7 and S8). We then filtered the list of enriched TFs, by including only those present in the *core set* gene. This led to the identification of the following nine transcription factors: Homeobox D9 (Hoxd9), Musculoaponeurotic fibrosarcoma (Maf), Nuclear receptor 4A1 (Nr4a1), CCAAT Enhancer Binding Protein Beta (Cebpb), Oligodendrocyte transcription factor2 (Olig2), One Cut Homeobox 2 (Onecut2), SAM Pointed Domain Containing ETS Transcription Factor (Spdef), Twist Family BHLH Transcription Factor 2 (Twist2) and Nuclear Transcription Factor Y Subunit Beta (Nfyb) (Figure 6). These nine transcription factors are predicted to directly regulate 66% (115/175) of the *core set* genes, with the majority of these predicted to be co-regulated by two or more of the transcription factors (Figure 6). In particular, the transcription factor Hoxd9 potentially targets the vast majority of the *core set* genes (degree = 142), as identified in the regulatory network analysis, and also represents the TF with the highest NES score for up-regulated genes (NES score = 4.269) (Figure 6a–c). On the other hand, the TF with the highest NES score for downregulated genes is Nr4a1 (NES score = 4.812) (Figure 6b). In addition, the “regulator-target genes analysis” identified Hoxd9, Maf, Nr4a1 and Cebpb as the most significantly overrepresented TFs in the promoters of both upregulated and downregulated *core set* genes (Figure 6a,b). Olig2 and Onecut2 were selectively enriched for upregulated *core set* genes, whereas Twist2, Nfyb and Spdef were predicted to potentially bind the motifs present only in downregulated *core set* genes (Figure 6a,b).

By using different online tools (i.e., ClinVar, Orphanet, DrugBank, TTD) we investigated the possibility to obtain a direct and selective hampering or enhancement of the expression of apoptotic/survival-related genes by rational targeting their regulatory transcription factors. Our analysis revealed that 5/9 TFs (Olig2, Twist2, Nr4a1, Cebpb, Onecut2) targeting *core set* genes are potential drug targets. Since the majority of these increase at 0.5 and 1 h during GF rescue, their pharmacological modulation might exert early anti-apoptotic or pro-survival effects.

**Figure 7:**
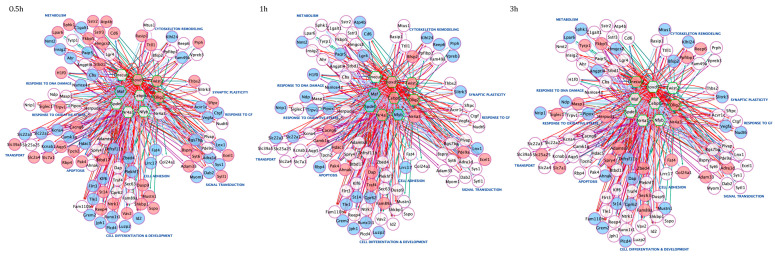
Time-course TF-gene regulatory networks revealed a common early and transient peak of transcription of upstream regulators modulating the expression of *core set* genes. Networks were visualized by *Cytoscape*. For each time-point, the node color is consistent with the logFC of each gene: genes in blue are downregulated by GF treatment, whereas the genes in red are upregulated. Targets are represented as circle nodes with purple borders and TF as green hexagon nodes. Regulons for each TF are represented by different edge colors. Target genes are grouped according to their biological functions.

Transcription regulatory network analysis of *core set* genes generated at each time point emphasizes how temporally distinct functional subprograms are orchestrated by interconnected TFs (Figure 7). This analysis predicted a high degree of cross-regulation among the nine transcription factors themselves and showed a common early (0.5 and 1 h) and transient peak of transcription for the majority of TFs, with the exception of Onecut2 that was activated 3 h after GF treatment (Figure 7). Consistent with their recognized role of immediate early genes, the rapid induction/inhibition of these transcription factors generate a coordinated transcriptional response in which *core set* genes and associated gene ontology functional groups are dynamically activated throughout the entire time course (Figure 7). For example, *core set* genes involved in apoptosis, cell differentiation and development exhibited rapid but transient transcriptional activation, suggesting their primary role in the immediate response to GF treatment, whereas the dynamic and constant regulation of genes associated with cytoskeleton organization, response to DNA damage, cell adhesion and metabolic processes supports their role as secondary effectors of the transcriptional program governing neuronal fate decisions (Figure 7).

### 3.4. Disease Enrichment Analysis Revealed a Strong Association of Core Set Genes with Neurological and Psychiatric Disorders

To investigate the potential clinical relevance of *core set* genes implicated in apoptosis and survival of CGNs, we performed a Disease Enrichment analysis with DAVID bioinformatics resources, including OMIM, KEGG DISEASE, and GAD catalogs. This analysis (Table 1) revealed that 70% of the *core set* genes (121/175) are associated with human diseases, including multiple disorders affecting the central nervous system.

Of particular interest was the significant association of 29 *core set* genes with cognitive/mental diseases (bipolar disorder, anxiety, depressive syndrome, schizophrenia, attention deficit and disruptive behavior disorders) (Table 2).

To further define the potential clinical implication of the *core set* genes, we also compared their gene expression changes with disease transcriptional signatures included in the integrative Library of Integrated Network-Based Cellular Signatures (iLINCS) web platform. A significant correlation was found with cancers and neuromuscular degenerative diseases (Amyotrophic Lateral Sclerosis and Duchenne muscular dystrophy, Table 3).

### 3.5. Identification of Repurposing Drugs That Could Revert the Transcriptional Regulation of the Core Set Genes during Neuronal Apoptosis

The integrative web platform iLINCS was also used to explore drugs that could revert the transcriptional regulation of the *core set* genes during the induction of apoptosis and, thus, represent putatively therapeutically useful candidates. The top 50 chemical compounds with high negative connectivity scores are listed in Table 4. Overall, almost all the LINCS perturbagens are established neuroprotective entities and were already found to be effective against neuronal cell death. Of particular interest, 15 out of 50 compounds are antipsychotic or antidepressant drugs (Table 4), further supporting a relationship between apoptosis and psychiatric disorders [31,32,33,34,35,36,37,38] and the potential use of neuroprotective agents [39,40,41,42,43,44,45].

## 4. Discussion

The ability of a neuron to undergo or evade apoptosis depends on the activity of an integrated network of genes and their encoded proteins. Here, for the first time, we overlayed transcriptome changes occurring in CGNs during the early stages (0.5, 1 and 3 h) of the pre-commitment phase of apoptosis and their rescue by three neurotrophic factors (Igf1, Pacap and SP) acting on different receptors/intracellular second messengers. Our analysis identified a *core set* of 175 genes deregulated with opposing trends during neuronal apoptosis and GF-mediated rescue (Figure 2), As expected, we identified both rapid and delayed transcriptional changes in response to GF rescue. In particular, 117 genes showed significant changes within 30 min of GF stimulation, as expected for immediate early genes, whereas 145 genes showed transcriptional changes after 1–3 h (Figure 2). The immediate early genes included a variety of elements involved in apoptosis, developmental process, transcriptional regulation and response to growth factor stimulus, supporting their potential role as transcriptional effectors in the induction of secondary response genes (Figure 4). On the other hand, delayed early response genes were enriched in functions related to many cellular processes, including cytoskeleton organization, response to DNA damage, cell adhesion and metabolic processes, suggesting their regulation may function as effectors of the transcriptional program governing neuronal fate decision (Figure 4). Interestingly, among these latter is Ahsa2, a gene encoding a co-chaperone that stimulates HSP90, an inducible molecular chaperone protecting neurons in neurodegenerative diseases, such as Alzheimer’s disease [46,47,48]. We found *Ahsa2* dynamically activated by GFs at 1 and 3 h following apoptotic induction. This supports the role of molecular chaperones as regulators in neuronal fate decision and indicators of cellular response to excessive protein damage (Figure 2, Figure 3 and Figure 4, Supplementary Table S6) [49,50,51,52,53].

According to the important role played by early response genes in response to neuronal activity and neuronal insults, functional analysis of the PPI network and its corresponding module screening, identified an important cluster of immediate early genes (i.e., *Ahr*, *Ntrk1*, *Id2*, *Cebpb*, *Twist2*, *Runx1t1*, *Maf*, *Runx2)* significantly associated with transcriptional regulation, cell differentiation and regulation of cell death (Figure 3, Figure 4 and Figure 5). Among these genes, *Cebpb* had the highest connectivity degree (degree = 92) in the general PPI network, and it was activated by all GFs tested at 1h following apoptotic induction (Figure 3). *Cebpb* encodes an important transcription factor regulating the expression of genes involved in immune and inflammatory responses as well as in synaptic plasticity, neurogenesis, neuronal proliferation and differentiation [54,55,56,57,58]. Previous studies demonstrated that upregulation of C/EBP β expression in rat primary cortical and cerebellar neuronal cultures plays neuroprotective and antiapoptotic effects, whereas reduced neuronal levels of this factor is associated with Parkinson’s disease and other synucleinopathies, suggesting it as a potential pharmacological target [59,60,61,62,63]. Another important gene belonging to this functional network module is *Ntrk1* (also known as *TrkA*), a gene activated by all three GFs in the first 0.5 h following apoptotic induction and encoding the high-affinity Nerve Growth Factor receptor exerting clinically relevant biological effects on neuronal cells, and promoting neuronal survival, proliferation and differentiation [64,65,66] (Figure 4 and Figure 5). On the other hand, decreased expression of the immediate early response gene *Id2,* encoding a transcriptional modulator critical for cell growth/differentiation and neural development, corroborates previous findings demonstrating that its suppression protects CGNs from apoptosis, whereas its overexpression induces neuronal death [67,68] (Figure 4 and Figure 5).

The dynamic of early state transcriptional changes underlying neuronal death/survival offered us the possibility to analyze for the first time the regulatory mechanisms underlying these processes. Our analysis revealed that temporally distinct modules of *core set* genes are regulated by the coordinated action of nine transcription factors *(Hoxd9*, *Maf*, *Nr4a1*, *Cebpb*, *Olig2*, *Onecut2*, *Spdef*, *Twist2*, *Nfyb*) (Figure 6 and Figure 7). The resulting TF-target gene regulatory network driving the neuronal shift between apoptosis and survival-inducing signals is highly interconnected. The temporal organization of this program reflects the interconnections of this network, and the activities of interconnected TFs are highly synchronous (Figure 6c and Figure 7). In particular, the extended analysis of regulatory networks suggests Hoxd9 as a master regulator of neuronal apoptosis and survival, as this TF is involved in the transcriptional regulation of both the vast majority of *core set* genes and other upstream regulators and is highly integrated with its targets through a plethora of interacting loops (Figure 6 and Figure 7). Hoxd9 belongs to a highly conserved family of homeobox-containing transcription factors that plays an important role during development of the central nervous system by regulating numerous processes, including cell proliferation, apoptosis, differentiation and angiogenesis [69,70] (Figure 7). We observed downregulation of Hoxd9 during apoptosis, whereas treatment with all three GFs increased its expression since the early stages (0.5 and 1 h) (Figure 2). Our results are in accordance with previous studies demonstrating that Hoxd9 regulates the expression of several genes involved in apoptosis, whereas its loss of function causes defects in axonal targeting and reduction in neural cell numbers [71]. Among the enriched TFs in upregulated *core set* genes, we identified *Olig2*, whose expression levels remain constantly increased throughout the entire time course, pointing to the essential role of this TF during different stages of GF rescue from neuronal apoptosis (Figure 6a and Figure 7). Olig2 has a critical function in regulating the appearance and development of different types of neurons in the developing central nervous system, directing neuronal fate choices and promoting cell proliferation through multiple cellular pathways [72,73,74]. Of note are previous studies showing activation of *Olig2* in response to different GFs (including FGF, GDNF and PDGF) elicits neuroprotective and pro-survival effects in different neuronal types [75,76,77,78]. Reduced expression of *Olig2* in neuronal cells switches cell fate from differentiation to death, contributing to acute/chronic diseases, including psychiatric disorders, Alzheimer’s disease and Amyotrophic Lateral Sclerosis (ALS), and thus proposing this factor as a potential therapeutic target for treatment of these conditions [79,80,81,82,83,84]. On the contrary, downregulated *core set* genes are commonly regulated by Nr4a1, a key component of the Nr4a orphan nuclear receptor family of transcription factors that are rapidly and strongly upregulated in response to a diverse range of signals, including growth factors, cytokines, membrane depolarization, excitotoxic and stressful insults to the central nervous system (Figure 6b and Figure 7). Among the 43 candidate *core set* genes that are targets of Nr4a1, there are a number of genes already involved in the regulation of apoptosis and RNA metabolic process, including other enriched TFs (*Maf*, *Nfyb* and *Spdef)* known to promote neuronal apoptosis and whose expression levels were reduced in response to GF-induced rescue effects [85,86,87] (Figure 6b). Nr4a sub-family members are categorized as early response genes with pleiotropic physiological roles, including maintaining neuronal integrity, regulating the density and distribution of spines and synapses, suppressing apoptosis and inducing pro-survival genes [88,89,90,91]. Interestingly, a marked decrease in *Nr4a1* was associated with a variety of neurological conditions, including Alzheimer’s and Parkinson’s diseases, schizophrenia and bipolar disorders, and various activators and modulators of this TF have been investigated as probable therapeutic drugs in neuroinflammatory and neuronal cell death models [92,93,94,95,96,97,98,99,100,101].

Our promoter motif analysis provides a detailed portrait of the dynamic regulatory cascade underlying trophic factor-induced neuronal survival and highlights how dysfunctions in this very intricate regulatory program may play a key role in the development of different neurological and neuropsychiatric disorders, sustaining the existence of a universal transcriptional software regulating apoptosis and survival in different neurons, cell types and species. This aspect is further confirmed by our disease and drug repositioning analysis that demonstrated the clinical implication of the *core set* genes in cancer as well as in multiple disorders affecting the central nervous system, including ALS, Duchenne muscular dystrophy, schizophrenia and other neuropsychiatric conditions, supporting their role as potential targets of new or already existent drugs for the treatment of these severe conditions (Table 1, Table 2, Table 3 and Table 4). Indeed, among the top list of drugs that could reverse the transcriptional regulation of the *core set* genes during the induction of apoptosis, are several compounds that protect neurons from apoptosis by exerting antioxidative, anti-inflammatory or neuroprotective effects. The list also included antidepressant and antipsychotic medications, as well as a variety of other compound classes or specific drugs (i.e., tozasertib, pentoxifylline) used in different clinical settings that may be further investigated in the context of neuronal apoptosis and related brain disorders [39,40,41,42,43,44,45] (Table 4).

## 5. Conclusions

Our analysis offers for the first time a systems biology-based perspective of the complex and coordinated temporal transcriptional programs underlying apoptosis and its rescue by neurotrophic factors, further sustaining that, although acting through different upstream signaling pathways, the GF-mediated survival effects were propagated by common transcriptional regulatory cascades. Besides elucidating the common mechanism and key genes by which neurotrophic factors elicit neuronal survival, we identify potential transcription factors that act as master regulatory switches to control neuronal apoptosis and survival in a temporally ordered manner. Finally, the functional exploitation of survival-related mediators allowed us to unravel their potential contribution in the pathogenesis of neurological and neuropsychiatric diseases, suggesting their role as targets for the development of therapies for these severe brain disorders. Future studies are needed to confirm these findings and to explore the role of other regulatory mechanisms, including microRNA-mediated transcriptional and post-transcriptional regulation [102,103,104,105,106,107,108].

Overall, the results presented here provide the foundation for further work to fully examine the universality of the transcriptional program governing neuronal life or death and what are the effects of its perturbation in human pathology. Similar to computers, where faults often arise from malfunctioning software, neuronal fate may critically depend on its transcription software. Thus, cracking the code of neuronal life or death may help in finding a patch for neurodegeneration through an innovative pharmacology focused on upstream targets and regulatory networks.

## Figures and Tables

**Table 1:** Disease-based functional annotation enrichment results of *core set* genes. The table shows disease enrichment results for all diseases significantly enriched with an adjusted *p*-value < 0.05.

Disease Class	*p* Value	Genes Number
Vision (*Glaucoma*, *Macular retinal edema*, …)	4.9 × 10^−3^	15
Psychiatric disorders	1.4 × 10^−2^	29
Hematological disease	4.4 × 10^−2^	22
Cardiovascular disease	4.7 × 10^−2^	50
Immune disease	5.0 × 10^−2^	37

**Table 2:** Disease genes from the “psychiatric disease” family and related disorders.

Gene Symbol	Gene Name	Disease
*ATP4B*	ATPase H+/K+ transporting beta subunit	Bipolar Disorder
*FAT4*	FAT atypical cadherin 4	Bipolar Disorder
*FKBP5*	FK506 binding protein 5	Depression, affective psychoses, post-traumatic stress disorder, bipolar disorders
*RASIP1*	Ras interacting protein 1	Bipolar Disorder
*ADRA1D*	Adrenoceptor alpha 1D	Several psychiatric disorders
*AHR*	Aryl hydrocarbon receptor	Dementia
*C1GALT1*	Core 1 synthase, glycoprotein-N-acetylgalactosamine 3-beta-galactosyltransferase 1	Bipolar Disorder
*CBS*	Cystathionine-beta-synthase	Dementia (AD), migraine disorders, schizophrenia
*DAP*	Death-associated protein	Schizophrenia
*GABRA6*	Gamma-aminobutyric acid type A receptor alpha-6-subunit	Schizophrenia, anxiety disorder
*ID2*	Inhibitor of DNA binding 2, HLH protein	Attention-deficit hyperactivity disorder
*INSIG2*	Insulin-induced gene 2	Schizophrenia
*MASP2*	Mannan binding lectin serine peptidase 2	Dementia
*NTSR1*	Neurotensin receptor 1	Schizophrenia, several psychiatric disorders
*NTRK1*	Neurotrophic receptor tyrosine kinase 1	Several psychiatric disorders, autism, dementia
*NR4A1*	Nuclear receptor subfamily 4 group A member 1	Schizophrenia, bipolar disorder
*NR4A3*	Nuclear receptor subfamily 4 group A member 3	Schizophrenia, bipolar disorder
*NUDT6*	Nudix hydrolase 6	Schizophrenia, bipolar disorder
*OLIG2*	Oligodendrocyte lineage transcription factor 2	Schizophrenia, obsessive compulsive disorder, Tourette syndrome, dementia
*PDE9A*	Phosphodiesterase 9A	Depression
*PLCD4*	Phospholipase C delta 4	Several psychiatric disorders
*PAQR5*	Progestin and adipoQ receptor family member 5	Mental Disorders
*SSTR2*	Somatostatin receptor 2	Several psychiatric disorders
*SSTR3*	Somatostatin receptor 3	Several psychiatric disorders
*SPRY4*	Sprouty RTK signaling antagonist 4	Schizophrenia
*SYT6*	Synaptotagmin 6	Mental Disorders
*TRPV1*	Transient receptor potential cation channel subfamily V member 1	Autism
*VEGFA*	Vascular endothelial growth factor A	Major depressive disorder, autism, dementia
*ZBED4*	Zinc finger BED-type containing 4	Schizophrenia, bipolar disorder

**Table 3:** The top 10 disease transcriptional signatures from iLINCS positively correlated with apoptotic CGN-related temporal expression changes.

Disease State	Concordance	*p* Value	No. of Genes
Lean	0.64	1.50 × 10^−3^	21
Adenocarcinoma	0.58	1.17 × 10^−6^	159
Hypernephroma	0.50	1.23 × 10^−2^	159
Carcinosarcoma	0.49	3.01 × 10^−2^	159
Renal_cell_carcinoma	0.49	4.11 × 10^−2^	159
Amyotrophic_lateral_sclerosis_	0.48	4.95 × 10^−5^	65
No_atrial_fibrillation	0.47	5.53 × 10^−5^	65
Duchenne_muscular_dystrophy	0.46	5.58 × 10^−5^	87
Carcinoma	0.42	1.22 × 10^−4^	80
B-cell acute lymphoblastic leukemia	0.42	3.19 × 10^−2^	26

**Table 4:** List of the top 50 repurposable drug candidates with a potential to reverse apoptotic CGNs transcriptomic signature.

Rank	Perturbation	*p*-Value	Correlation Score	Mechanism of Action	Pharmacological Class (Current Indication)
1	Tozasertib	2.08 × 10^−5^	−0.98	Aurora A/B/C kinases inhibitor	Chemotherapeutic
2	Necrostatin	5.29 × 10^−5^	−0.97	RIP1 kinase inhibitor	Inhibitor of necroptosis
3	Tianeptine	8.71 × 10^−5^	−0.97	Mu-type opioid receptor agonist	Tricyclic antidepressant
4	L-Sulforaphane	1.00 × 10^−4^	−0.97	N/A	Anticancer
5	Pentoxifylline	1.77 × 10^−4^	−0.96	Phosphodiesterase inhibitor	Hemorheological agent
6	Purmorphamine	1.80 × 10^−4^	−0.96	Sonic Hedgehog agonist	-
7	Nicergoline	1.91 × 10^−4^	−0.96	Alpha-1A adrenergic receptor antagonist	Vasodilator Agent
8	Pifithrin	2.95 × 10^−30^	−0.95	p53 inhibitor	-
9	5-Nonyloxytryptamine	2.72 × 10^−4^	−0.95	Serotonin Receptor Agonist	-
10	Nifedipine	2.79 × 10^−4^	−0.95	Specific blocker of L-type calcium channels	Antihypertensive, Antianginal
11	Tyrphostin	1.54 × 10^−15^	−0.92	EGFR inhibitor	Antineoplastic
12	Parthenolide	4.11 × 10^−15^	−0.92	NFKB inhibitor	-
13	Atorvastatin	1.03 × 10^−6^	−0.90	HMG-CoA inhibitor	Statin (used to lower lipid levels and reduce the risk of cardiovascular disease)
14	Tanespimycin	7.69 × 10^−5^	−0.86	HSP inhibitor	Anticancer
15	Monorden/Radicicol	6.94 × 10^−3^	−0.85	HSP inhibitor	-
16	Azacyclonol	1.55 × 10^−2^	−0.81	N/A	Antipsychotic
17	Rapamycin	1.66 × 10^−6^	−0.54	mTOR inhibitor	Immunosuppressive
18	Amitriptyline	7.97 × 10^−6^	−0.50	Norepinephrine and serotonin reuptake inhibitor	Tricyclic antidepressant
19	Allopurinol	8.01 × 10^−6^	−0.50	Xanthine dehydrogenase/oxidase inhibitor	Xanthine Oxidase Inhibitors; Antigout Agents
20	Nortriptyline	8.62 × 10^−6^	−0.50	Multiple	Tricyclic antidepressant
21	Bupropion	1.57 × 10^−5^	−0.49	Norepinephrine/dopamine-reuptake inhibitor	Antidepressant
22	Roflumilast	1.84 × 10^−5^	−0.48	Phosphodiesterase-4 inhibitor	Tricyclic antidepressant
23	Tranilast	3.41 × 10^−5^	−0.47	Hematopoietic prostaglandin D synthase inhibitor	Antiallergic
24	Indomethacin	3.10 × 10^−5^	−0.47	COX inhibitor	Non-steroidal anti-inflammatory drug
25	Nystatin	3.24 × 10^−5^	−0.47	Channel-forming ionophore	Antifungal
26	Theophylline	3.80 × 10^−5^	−0.47	Adenosine receptor antagonist	Bronchodilator
27	Citalopram	3.85 × 10^−5^	−0.47	Reuptake of serotonin inhibitor	Antidepressant
28	Piracetam	4.11 × 10^−5^	−0.47	Acetylcholine receptor agonist	Antipsychotic
29	Tacrolimus	5.22 × 10^−5^	−0.46	Peptidyl-prolyl cis-trans isomerase FKBP1A, inhibitor	Immunosuppressive
30	Diazepam	8.10 × 10^−5^	−0.45	GABA(A) Receptor positive allosteric modulator	Anxiolytic, sedative
31	Iproniazid	7.33 × 10^−5^	−0.45	MAO inhibitor	Antidepressant
32	Promazine hydrochloride	5.32 × 10^−4^	−0.45	Dopamine receptor antagonist	Antipsychotic
33	Cyproheptadine	1.62 × 10^−2^	−0.45	Histamine receptor antagonist	Antiallergic
34	Dipyrone	1.07 × 10^−4^	−0.44	N/A	Non-steroidal anti-inflammatory drug
35	Ethosuximide	1.0 × 10^−4^	−0.44	T-type voltage sensitive calcium channels inhibitor	Anticonvulsants
36	Phenotiazine	1.09 × 10^−4^	−0.44	N/A	Antipsychotic
37	Sulfanilamide	1.12 × 10^−4^	−0.44	Dihydropteroate synthase inhibitor	Antibiotic
38	Clozapine	1.35 × 10^−4^	−0.44	Dopamine/Serotonin antagonist	Antipsychotic
39	Lamotrigine	1.68 × 10^−4^	−0.43	Multiple	Antiepileptic
40	Doxepin	1.94 × 10^−4^	−0.43	Selective histamine H1 receptor blocker	Tricyclic antidepressant
41	Moclobemide	2.22 × 10^−4^	−0.43	MAO inhibitor	Antidepressant
42	Rifabutin	2.34 × 10^−4^	−0.43	DNA-dependent RNA polymerase inhibitor	Antibiotic
43	Rolipram	2.15 × 10^−4^	−0.43	N/A	Antidepressant
44	Enaplapril	2.75 × 10^−4^	−0.42	ACE inhibitor	Antihypertensive
45	Geldanamycin	1.62 × 10^−6^	−0.42	N/A	Anticancer
46	Sibutramine	3.77 × 10^−4^	−0.41	Dopamine, norepinephrine, serotonin transporter inhibitor	Antiobesity
47	Phenelzine	9.79 × 10^−4^	−0.38	MAO inhibitor	Antidepressant
48	Thioridazine	4.51 × 10^−5^	−0.36	Dopamine receptor antagonist	Antipsychotic
49	Artemisinin	1.09 × 10^−5^	−0.36	N/A	Antimalarials
50	Withaferin A	9.48 × 10^−5^	−0.35	N/A	Anticancer

## Data Availability

Data are contained within the Supplementary Materials. Microarray data were deposited in NCBI’s Gene Expression Omnibus (GEO) with the accession number GSE179383.

## Supplementary Materials

The following are available online at https://www.mdpi.com/article/10.3390/cells10113238/s1: Figure S1: Induction of apoptosis in CGNs and rescue by GFs treatment; (a) After 6 days in vitro, extracellular KCl was shifted from 25 to 5 mm for inducing apoptotic death in CGNs, whereas control neurons were incubated with a serum-free medium supplemented with 25 mm KCl (K25). To investigate the rescue effects of GFs, K5 neurons were treated with a maximal effective dose of SP, Pacap or Igf1. 48 h after apoptosis induction or rescue by GFs, neuronal viability was assessed and values shown represent the mean ±S.E.M. of four to eight determinations in two different experiments; (b) Representative fluorescence microscopy images of Hoechst 33342 staining of CGCs in control conditions (K25), 72 h after induction of apoptosis (K5) or its rescue by SP treatment. Apoptotic CGNs show chromatin condensation; Table S1: Lists of genes with significant temporal expression changes in apoptotic CGNs (K5 vs. K25); Table S2. Lists of genes with significant temporal expression changes in CGNs during rescue by Pacap (K5 + Pacap vs. K5); Table S3: Lists of genes with significant temporal expression changes in CGNs during rescue by Igf1 (K5 + Igf1 vs. K5); Table S4: Lists of genes with significant temporal expression changes in CGNs during rescue by SP (K5 + SP vs. K5); Table S5: Lists of genes with significant temporal expression changes in CGNs at the intersection of the four experimental conditions (K5 vs. K25, K5 + SP vs. K5, K5 + Pacap vs. K5, K5 + Igf1 vs. K5); Table S6: Lists of *core set* genes at the intersection of apoptosis (K5 vs. K25) and GF-mediated neuronal rescue (K5 + SP vs. K5, K5 + Pacap vs. K5, K5 + Igf1 vs. K5); Table S7: The results of upstream regulator analysis from up-regulated core set genes based on iRegulon; Table S8: The results of upstream regulator analysis from downregulated core set genes based on iRegulon.
