# Supplementary material for: Transcriptional Profiles of Cell Fate Transitions Reveal Early Drivers of Neuronal Apoptosis and Survival
Source: Cells. 2021 Nov 19;10(11):3238. doi: 10.3390/cells10113238 (PMC8620386; doi:10.3390/cells10113238)
Supplement: Supplementary file 1 [file cells-10-03238-s001.zip › cells-1448701-supplementary.pdf]

Original Article

Transcriptional profiles of cell fate transitions reveal early drivers of neuronal apoptosis and survival

Giovanna Morello, Ambra Villari, Antonio Gianmaria Spampinato, Valentina La Cognata, Maria Guarnaccia, Giulia Gentile, Maria Teresa Ciotti, Pietro Calissano, Velia D'Agata, Cinzia Severini, Sebastiano Cavallaro

Supplemental Figure & Table Guide

Figure S1. Induction of apoptosis in CGNs and rescue by Igf1, Pacap and SP treatment. (a) Cultured CGNs at 6 days 'in vitro' were switched into serum-free medium containing lower concentrations of extracellular K+ (5 instead of 25 mM) for an acute induction of apoptotic death. For-ty-eight hours later, neuronal viability was assessed by counting the number of intact nuclei. Val-ues for neuronal viability represent the mean ± S.E.M. of four to eight determinations in two dif-ferent experiments; (b) Representative immunofluorescence photomicrographs showing CGCs stained with Hoechst in control conditions (K25) and after 72h of low K+ concentration (K5). As previously demonstrated, apoptosis induced by K5 conditions, characterized by shrunken and condensed nuclei, was reversed by SP treatment (*Amadoro et al., 2007*).

Table S1. Lists of genes with significant temporal expression changes in apoptotic CGNs (K5 vs K25).

Table S2. Lists of genes with significant temporal expression changes in CGNs during rescue by PACAP (K5 + PACAP vs K5).

Table S3. Lists of genes with significant temporal expression changes in CGNs during rescue by IGF1 (K5 + IGF1 vs K5).

Table S4. Lists of genes with significant temporal expression changes in CGNs during rescue by SP (K5 + SP vs K5).

Table S5. Lists of genes with significant temporal expression changes in CGNs at the intersection of the four experimental conditions (K5 vs K25, K5+SP vs K5, K5+PACAP vs K5, K5+IGF1 vs K5).

Table S6. Lists of *core set* genes at the intersection of apoptosis (K5 vs K25) and GF-mediated neuronal rescue (K5+SP vs K5, K5+PACAP vs K5, K5+IGF1 vs K5)

Table S7. The results of upstream regulator analysis from up-regulated *core set* genes based on *iRegulon*.

Table S8. The results of upstream regulator analysis from down-regulated *core set* genes based on *iRegulon*.

a.

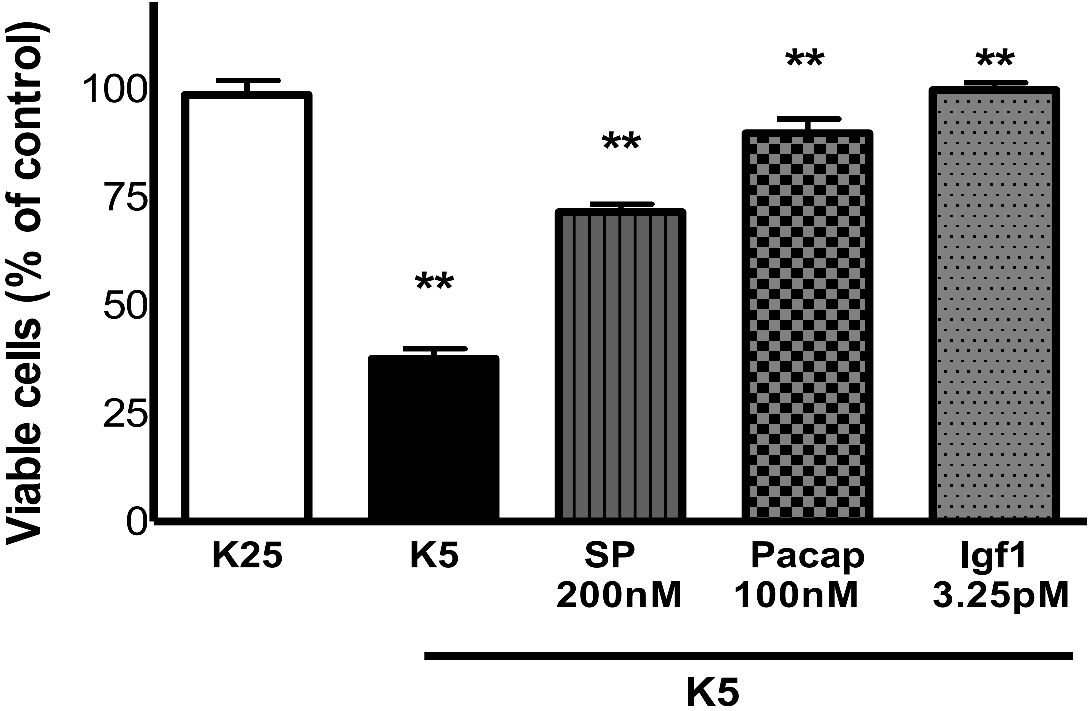
Viable cells (% of control)
100
75
50
25
0
**
**
**
**
K25
K5
SP
200nM
Pacap
100nM
Igf1
3.25pM
K5

b.

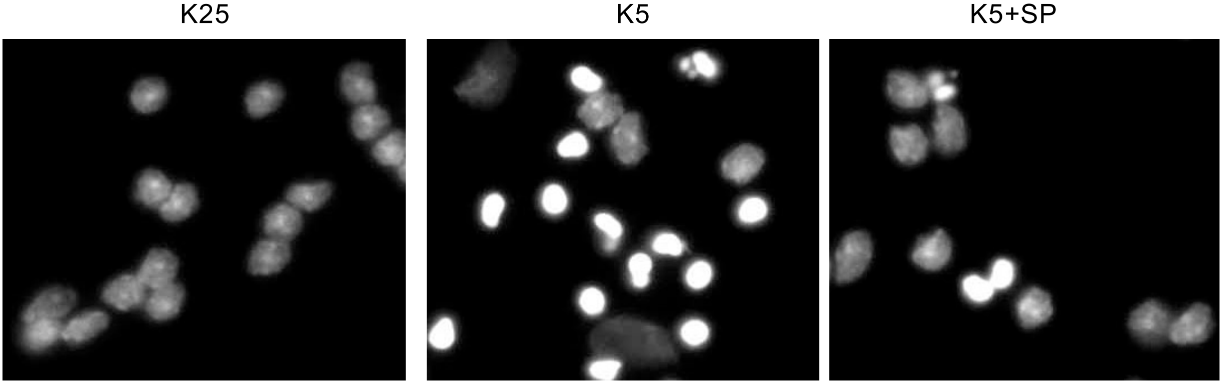
K25
K5
K5+SP

Supplementary Table 1. Lists of genes with significant temporal expression changes in apoptotic CGNs (K5 vs K25).

ProbeName	GeneSymbol.x	p-value	R-squared	K5_vs_K25_05h	K5_vs_K25_1h	K5_vs_K25_3h
A_42_P453976	Ifitm6	2,45E-18	0,849122394	-1,03	1,16	3,20
A_42_P457692	Zfand2a	6,91E-13	0,696657286	1,06	-1,17	-1,02
A_42_P459212	Tnfrsf21	3,49E-17	0,756548439	1,14	-1,01	-3,02
A_42_P459431	Bphl	6,18E-10	0,607016283	-1,00	1,22	1,70
A_42_P459855	RGD1562618	2,94E-08	0,630994556	-1,35	-1,11	1,02
A_42_P461144	Skida1	1,84E-13	0,668711645	-1,08	1,22	1,71
A_42_P461794	Clic3	1,19E-23	0,869492097	-2,28	1,22	12,98
A_42_P462015	Stn1	1,03E-24	0,880649895	-1,11	1,00	-1,33
A_42_P462257	Rprml	2,45E-31	0,953960826	1,10	1,56	3,97
A_42_P463998	Abcc6	9,33E-16	0,763663563	-1,33	1,11	3,16
A_42_P464378	Itih3	6,43E-14	0,722735839	1,26	1,15	2,11
A_42_P467956	Sestd1	2,04E-11	0,607373758	1,07	-1,00	-1,49
A_42_P471507	Unc50	6,25E-17	0,769956405	-1,04	-1,07	-1,51
A_42_P472375	Pdk2	1,36E-10	0,650600208	-1,04	-1,13	1,85
A_42_P472699	Amn	2,17E-21	0,854700388	1,33	1,50	4,06
A_42_P473398	Cxcl1	2,97E-26	0,912550029	2,01	1,04	1,38
A_42_P473425	Unc13d	5,48E-19	0,86832868	-1,97	1,42	5,57
A_42_P473594	Egr2	3,03E-26	0,895081447	2,27	1,16	1,12
A_42_P475623	Klf5	2,13E-13	0,666940465	1,49	-1,09	-2,01
A_42_P475871	Prss27	1,20E-12	0,645546403	1,05	-1,00	1,65
A_42_P478451	Wscd1	2,34E-27	0,89485579	1,00	1,27	2,64
A_42_P479802	Dffa	3,31E-17	0,757009064	1,12	-1,18	-2,10
A_42_P481087	Pdzrn3	2,28E-16	0,717402425	1,26	1,11	-1,72
A_42_P482623	Dstyk	2,45E-16	0,77525487	1,11	1,02	1,32
A_42_P484738	Ctgf	2,06E-21	0,876696956	1,30	-1,09	1,04
A_42_P485189		2,10E-21	0,828225017	-1,05	3,48	7,49
A_42_P486964	Rph3al	7,34E-24	0,871786527	1,47	1,52	12,46
A_42_P491454	Gnat1	2,92E-23	0,886339724	1,12	1,05	1,82
A_42_P491505	Purb	4,37E-14	0,70723156	1,06	1,20	1,27
A_42_P492882	Nckipsd	1,80E-11	0,634187812	1,30	-1,13	-2,49
A_42_P493380	Myc	1,55E-25	0,914758821	-1,11	-4,82	-5,02
A_42_P493925	Usp48	3,88E-14	0,745738479	1,26	1,21	1,35
A_42_P494276	Armc9	1,06E-09	0,642175204	-1,11	-1,11	-1,38
A_42_P495001	Slc17a5	7,94E-12	0,667195876	-1,13	1,06	1,41
A_42_P495579	Ano1	3,07E-30	0,924994154	1,17	1,67	25,19
A_42_P497323	Ppp1r10	4,03E-29	0,91761436	1,05	2,23	7,16
A_42_P499221	Fgd5	5,70E-14	0,704336224	1,18	1,24	2,93
A_42_P499282	Msrb2	6,43E-13	0,697488438	-1,07	1,18	1,77
A_42_P501233	Adra1d	1,17E-24	0,907811724	1,23	-1,15	-3,73
A_42_P501972	Ppp2r1b	5,03E-12	0,626713905	1,15	1,19	1,16
A_42_P502621	Avil	9,70E-18	0,767493965	-1,08	2,07	3,50
A_42_P504358	RGD1305347	2,41E-15	0,693036653	-1,14	1,02	4,13
A_42_P504653	F10	3,62E-15	0,732913491	-1,49	1,35	3,33
A_42_P505320	Cipc	1,75E-13	0,669286368	1,34	1,76	2,36
A_42_P506402	Slc25a25	3,29E-12	0,678163418	1,01	-1,42	-1,89
A_42_P508921	Aqp3	6,40E-17	0,801384405	-1,44	1,78	3,55
A_42_P509967	Slc7a9	1,63E-11	0,657976399	-1,15	-1,04	2,22
A_42_P511118	Slc17a6	2,66E-13	0,664241998	1,02	1,00	1,35
A_42_P514244	Dyrk1a	1,31E-18	0,764201957	1,12	-1,34	-1,61
A_42_P515405	Ier3	5,01E-21	0,891250978	1,59	-1,00	1,41
A_42_P517554	Polg2	1,27E-14	0,699079972	1,06	1,35	1,22
A_42_P518033	Hsd17b1	1,52E-11	0,611491517	1,08	1,07	1,73
A_42_P522194	Atp2a3	5,03E-27	0,918255793	-1,17	2,34	8,84
A_42_P522638	Pqlc3	3,54E-13	0,704239891	1,14	1,78	1,53
A_42_P523517	Ttyh2	3,17E-19	0,810512686	1,05	1,23	4,18
A_42_P523612	Mef2c	2,52E-12	0,701052746	1,39	-1,04	-1,93
A_42_P527724	Fcnb	2,25E-16	0,717550006	-1,19	1,09	2,70
A_42_P528106	Glce	8,08E-16	0,764933358	1,02	1,52	1,57
A_42_P528537	Cdkn2b	1,04E-10	0,654347621	-1,22	-1,10	1,03
A_42_P531971	Hist3h2a	3,73E-20	0,824794995	1,25	1,90	3,09
A_42_P535152	Asap3	6,72E-18	0,770532368	1,00	1,22	1,45
A_42_P535644	Adamts1	4,73E-13	0,700981015	1,43	-1,00	-1,22
A_42_P536292	Ngb	6,38E-25	0,909944188	-1,14	1,37	2,85
A_42_P536702	Sftpc	2,61E-23	0,886838817	-1,41	2,96	6,83
A_42_P538868	Dvl1	4,95E-11	0,643172217	1,27	-1,03	-1,82
A_42_P540950	Ier2	8,42E-30	0,951959369	1,39	1,15	1,22
A_42_P540972	Rasl11b	2,64E-15	0,75420941	1,59	1,13	1,12
A_42_P541025	Hspa1l	1,33E-27	0,88598109	1,44	1,74	3,84

A_42_P541884	Irak2	3,87E-22	0,851777651	1,02	1,32	1,79
A_42_P542960	Rerg	4,20E-10	0,655246957	1,05	-1,23	1,43
A_42_P542985	Cbln2	4,02E-20	0,837932664	1,29	-1,09	-3,39
A_42_P543004	Socs2	1,56E-16	0,762109574	-1,12	-1,23	-1,41
A_42_P545100	Aspdh	9,19E-15	0,723579196	-1,18	1,25	1,90
A_42_P546179	Tbc1d1	7,69E-10	0,603707673	1,02	1,13	-1,32
A_42_P547036	Pak4	2,91E-10	0,697205897	-1,16	-1,12	1,08
A_42_P547629	Fam49a	1,15E-11	0,719245696	1,92	1,13	-1,68
A_42_P548410	Acot1	1,18E-12	0,69045879	1,19	1,34	2,16
A_42_P550241	Fat2	3,73E-18	0,775341039	-1,18	1,04	2,05
A_42_P550344	Tle3	1,08E-18	0,81666687	1,19	1,05	1,15
A_42_P554066	Tasp1	8,32E-12	0,619880912	1,01	1,10	1,27
A_42_P555131	Tmbim7	1,29E-13	0,695301448	-1,24	1,24	4,37
A_42_P555140	Izumo4	5,38E-13	0,718463966	1,08	1,13	-1,17
A_42_P555780	C5	2,52E-14	0,732383613	-1,21	1,61	4,10
A_42_P558703	Adamts15	1,32E-18	0,828862433	1,33	1,04	-2,89
A_42_P559038	Plekhg2	1,57E-11	0,611053723	-1,11	1,28	2,73
A_42_P559414	Wnt4	3,78E-32	0,936103718	1,35	2,31	3,68
A_42_P565301	B4galt5	3,25E-12	0,604734878	1,00	-1,07	-1,37
A_42_P566264	Mfng	6,92E-24	0,882758247	-1,08	1,17	3,55
A_42_P567527	Traf4	4,67E-12	0,673855816	-1,01	-1,53	-2,07
A_42_P568916	Gatad2a	1,57E-10	0,62712196	1,01	-1,06	-1,25
A_42_P574859	Hamp	2,76E-13	0,773934318	-1,55	1,11	2,42
A_42_P577431	Bcl3	3,07E-28	0,902223332	1,10	1,51	1,67
A_42_P580973	Car4	1,84E-21	0,829030526	1,91	1,68	2,77
A_42_P581135	Ist1	3,64E-12	0,67692096	1,04	-1,16	-1,19
A_42_P582467	Podxl	5,03E-12	0,626735081	-1,04	1,17	1,42
A_42_P583546	Lims2	7,19E-19	0,788225938	1,25	1,01	2,99
A_42_P584179	Lrrc73	7,30E-17	0,76864026	1,18	-1,15	-2,28
A_42_P584734	Gdf10	5,37E-31	0,936262669	-1,28	2,77	12,79
A_42_P588785	Mthfd2	8,01E-20	0,81982588	1,12	-1,07	-2,00
A_42_P589201	Slc15a4	4,20E-11	0,622503408	1,11	-1,03	1,61
A_42_P592981	Mfap2	3,27E-14	0,688650229	-1,11	-1,06	1,80
A_42_P593020	Rln1	5,87E-25	0,883089223	1,20	3,19	5,09
A_42_P597322	LOC100125367	1,50E-20	0,866772702	1,06	2,63	2,51
A_42_P599074	Srsf3	5,69E-12	0,625071293	1,17	1,46	2,40
A_42_P603213	Elfn2	1,58E-22	0,868223343	1,28	1,40	2,37
A_42_P603461	Isoc1	2,60E-13	0,726305858	-1,02	-1,01	-1,37
A_42_P604033	Eaf2	8,62E-18	0,801815603	1,11	1,28	1,60
A_42_P605985	Ddit4	1,83E-12	0,685268254	1,37	1,61	1,05
A_42_P608768	Bbs12	7,69E-18	0,802662896	1,21	1,58	2,31
A_42_P610788	Ampd3	8,17E-18	0,768917989	1,06	1,46	2,38
A_42_P612977	Cyb561	3,20E-20	0,839313148	1,27	1,53	3,08
A_42_P614175	Gal	7,35E-24	0,859714292	1,34	2,09	5,34
A_42_P615837	Ckmt2	3,09E-13	0,724447252	-1,13	1,04	-1,48
A_42_P618217	Fgf21	5,96E-19	0,820704335	-1,14	1,18	3,39
A_42_P618256	Tmem5	6,79E-16	0,706383943	-1,03	-1,42	-1,90
A_42_P619806		1,10E-28	0,922310651	1,18	1,15	3,46
A_42_P621872	Gcgr	2,85E-14	0,665245654	1,77	1,39	2,04
A_42_P623839	Ttc39b	1,34E-23	0,879818133	1,15	-1,06	-3,42
A_42_P624726	Cant1	4,40E-15	0,686476392	-1,01	1,22	1,88
A_42_P625147	Pglyrp1	4,12E-10	0,635105336	-1,23	-1,18	1,42
A_42_P629321	Wisp2	4,74E-11	0,664771209	-1,03	1,09	1,73
A_42_P629370	Klf15	1,24E-13	0,750888277	1,40	1,45	-2,02
A_42_P629500	Eif2ak3	2,26E-16	0,739678078	1,30	1,13	3,38
A_42_P630572	Cpne8	1,01E-09	0,621964218	1,74	1,05	-1,75
A_42_P636761	Gzmm	4,82E-15	0,808847477	1,72	1,52	2,33
A_42_P637189	Apln	1,50E-19	0,815644541	1,60	1,15	-1,60
A_42_P638181	Serinc5	7,01E-13	0,65232153	1,15	1,14	1,88
A_42_P640792	Gjb6	1,97E-12	0,70388471	1,23	1,69	1,94
A_42_P641004	Il1a	1,28E-18	0,783807256	1,55	1,10	-1,49
A_42_P646922	Sgcb	2,77E-20	0,852382831	1,07	1,04	-1,48
A_42_P648200	Pak1ip1	8,96E-12	0,685904066	1,08	1,14	-1,95
A_42_P648475	Plxnc1	8,43E-14	0,700041456	-1,06	1,04	1,65
A_42_P652586	Stat5a	6,47E-14	0,655466145	1,11	1,30	2,99
A_42_P654865	Otub2	2,78E-13	0,637384435	1,08	1,37	2,00
A_42_P655897	Tspan9	2,26E-22	0,877126145	-1,28	-1,28	3,65
A_42_P656495	Actn3	3,51E-13	0,660885592	1,00	1,06	1,72
A_42_P661018	Phlpp1	1,38E-12	0,643780347	1,07	-1,22	-1,90
A_42_P663500	Lgi3	5,31E-17	0,752857479	1,33	1,94	4,02
A_42_P664544	Rftn2	4,98E-12	0,673053345	1,18	1,16	-1,08

A_42_P664913	Ankrd37	4,36E-31	0,936757961	1,20	-1,03	-1,33
A_42_P667148	Plscr1	2,05E-15	0,718192606	1,10	1,47	1,50
A_42_P667696	Dclk3	2,13E-14	0,693450117	1,17	1,17	1,64
A_42_P669103	Lnx2	3,29E-21	0,839704122	-1,15	1,16	1,92
A_42_P670580	Vipr1	2,94E-33	0,947476406	1,23	2,03	2,67
A_42_P670631	RGD1305645	6,60E-20	0,834896191	1,21	1,04	1,39
A_42_P671422	Xkr5	3,30E-21	0,80886414	-1,19	1,12	2,15
A_42_P673905	Mrnip	3,96E-20	0,861635086	-1,08	1,13	2,12
A_42_P674843	Scn4b	1,50E-21	0,814064113	1,17	1,99	2,78
A_42_P675031	Kcnb1	4,37E-13	0,658204183	1,01	1,22	2,32
A_42_P676553	Tnni3	9,18E-13	0,672374677	1,27	1,11	1,33
A_42_P677662	Ttc30b	5,05E-18	0,772877111	1,12	1,52	2,09
A_42_P681441	Eps8l2	7,28E-25	0,882167042	1,33	1,69	6,09
A_42_P682589	Nr4a1	1,03E-28	0,951719014	1,09	-6,26	-15,25
A_42_P683837	Serpinb6b	3,05E-10	0,617484041	1,07	1,44	2,19
A_42_P683840	Ccl27	3,87E-20	0,850478647	-1,03	-1,00	-1,50
A_42_P684538	Slc2a1	1,72E-17	0,796629922	1,30	-1,24	-1,63
A_42_P684885	Atp12a	6,04E-18	0,788865975	-1,16	1,59	5,11
A_42_P685145	Cmpk2	2,13E-13	0,666915165	-1,21	1,14	1,83
A_42_P685330	Ap1s2	1,89E-16	0,760380359	1,09	1,30	1,84
A_42_P686879	Siglec1	6,32E-10	0,649523091	-1,32	-1,16	-1,37
A_42_P687819	Atoh8	1,08E-33	0,949395502	-1,00	2,61	5,06
A_42_P688266	Arap3	1,98E-12	0,684276925	-1,39	1,06	1,80
A_42_P689873	Cmtm8	1,59E-21	0,829930783	-1,28	-1,11	3,42
A_42_P694161	Arl14ep	3,47E-10	0,615612636	1,17	1,32	1,09
A_42_P694928	Arl5c	5,61E-19	0,834376323	2,00	2,09	3,40
A_42_P695042	Csf1	2,40E-18	0,83756443	1,12	-1,04	-1,63
A_42_P703023	Slc38a3	1,89E-11	0,608434964	1,18	1,25	3,10
A_42_P707838	Man2a1	1,38E-12	0,667436715	1,23	1,16	-2,48
A_42_P708593	Per2	3,99E-18	0,821451542	1,43	1,44	-3,87
A_42_P709423	Mlph	1,32E-17	0,744281873	-1,49	-1,10	4,65
A_42_P712297	Fa2h	3,63E-14	0,687492189	1,23	2,09	3,02
A_42_P712801	Entpd3	4,90E-27	0,925444078	1,05	1,32	1,50
A_42_P714311	Ccl3	1,78E-16	0,793430339	1,27	-1,24	-1,30
A_42_P718331	Sstr3	6,97E-37	0,975058259	-1,17	2,44	6,66
A_42_P721692	Inpp4b	8,16E-21	0,859112078	1,17	1,04	-1,94
A_42_P723173	Id1	1,08E-31	0,950760412	1,38	1,24	1,05
A_42_P723540	Dyrk3	2,60E-20	0,827108147	1,25	-2,02	-3,66
A_42_P723641	Popdc2	2,56E-18	0,758600676	1,45	1,35	3,83
A_42_P726234	Tex33	6,40E-09	0,614973196	-1,37	1,01	2,04
A_42_P727022	Rab40b	1,76E-10	0,601838374	1,03	1,48	1,36
A_42_P729012	Cercam	1,35E-13	0,714857127	-1,02	-1,07	1,38
A_42_P730320	Clhc1	4,36E-14	0,726781595	-1,15	1,08	4,70
A_42_P734476	Ttll3	1,77E-15	0,719676831	-1,26	1,42	2,40
A_42_P735670	Mpp5	8,49E-15	0,743147782	-1,12	-1,03	1,60
A_42_P738559	Pdgfa	2,21E-11	0,631376092	1,42	1,50	1,06
A_42_P739860	Dusp5	6,20E-19	0,820446307	1,43	2,32	3,91
A_42_P740370	Cpxm2	3,63E-28	0,918784627	1,11	1,36	4,06
A_42_P740939	St3gal4	2,82E-13	0,637202771	1,25	1,07	-1,62
A_42_P743580	Rgs4	1,05E-28	0,929437293	-1,05	-1,35	-1,93
A_42_P746838	Spib	5,54E-30	0,923364885	-1,19	4,25	7,40
A_42_P750340	Ifitm10	1,05E-16	0,782308144	-1,30	-1,03	3,14
A_42_P752416	Twf2	1,75E-13	0,643199038	-1,21	-1,01	1,78
A_42_P754654	RGD1562378	3,78E-25	0,911757871	1,32	2,41	3,20
A_42_P754888	Dlk1	1,65E-11	0,635330857	-1,84	-1,58	-2,02
A_42_P756652	Dnph1	1,93E-13	0,668113297	-1,20	1,26	1,69
A_42_P758200	Rnf112	2,46E-12	0,660182055	1,07	1,15	1,92
A_42_P759843	Arl4c	1,35E-16	0,795599055	1,36	1,01	-2,14
A_42_P759993	Mefv	5,64E-12	0,671508243	-1,00	1,60	3,28
A_42_P760354	Pla2g2c	8,41E-15	0,703513819	-1,28	1,36	5,17
A_42_P761023	Vstm5	2,33E-24	0,896772908	-1,03	1,60	3,22
A_42_P762829	Cebpd	7,83E-26	0,900794423	1,10	-1,00	1,23
A_42_P764359	Serbp1	4,44E-10	0,611960788	1,13	1,08	-1,36
A_42_P766909	Ppp1r14a	2,26E-14	0,76717353	-1,31	-1,06	2,22
A_42_P767077	Gja4	1,01E-11	0,68441906	1,18	2,15	2,30
A_42_P768024	Spn	2,58E-17	0,759178175	1,01	2,50	3,42
A_42_P768355	Hrk	1,27E-34	0,953265179	1,04	1,41	11,37
A_42_P768883	Perp	5,81E-17	0,787097571	1,37	1,35	1,17
A_42_P777268	Cep126	5,16E-17	0,771573539	1,20	1,77	1,47
A_42_P779933	Capn3	3,18E-11	0,669977703	-1,02	1,15	1,89
A_42_P780613	Hs3st2	4,24E-28	0,91022213	1,12	-1,02	2,64

A_42_P781659	Reps1	6,86E-15	0,705669623	1,19	-1,02	-2,04
A_42_P787775	Plekhd1	2,04E-18	0,825970661	-1,10	-1,02	1,77
A_42_P791677	Areg	1,77E-13	0,711937816	2,10	1,45	-1,00
A_42_P794120	Stra6	3,17E-21	0,852615142	1,14	1,21	1,80
A_42_P794748	Acad11	3,94E-11	0,623392177	1,16	1,13	2,03
A_42_P795269	Lgalsl	2,15E-12	0,720992591	1,19	1,13	-1,63
A_42_P797218	Gpt	1,99E-09	0,611722902	-1,06	1,02	-1,56
A_42_P800852	Ubr3	4,49E-15	0,710127323	1,30	1,03	-1,77
A_42_P800949	Hipk3	3,71E-09	0,623458536	1,55	-1,07	-1,09
A_42_P802358	Fam110b	9,61E-10	0,60030019	1,05	1,23	2,37
A_42_P803590	Tatdn3	7,06E-16	0,705981071	1,05	1,31	1,90
A_42_P803810	Myo5c	9,61E-11	0,634015497	1,46	1,44	2,21
A_42_P808442	Siae	4,02E-22	0,83809296	-1,04	1,27	2,45
A_42_P808945	Mustn1	6,51E-14	0,680844355	-1,05	1,22	1,29
A_42_P812805	Smoc2	9,92E-09	0,608037116	1,07	1,09	1,49
A_42_P814235	P2ry6	5,20E-15	0,684643544	1,20	1,18	1,84
A_42_P816878	Rgs7bp	6,46E-10	0,606328068	1,43	1,13	-1,55
A_42_P817417	PVR	2,56E-11	0,629358033	1,31	1,06	1,05
A_42_P820657	Il1r2	8,45E-13	0,747680923	1,03	1,16	1,41
A_42_P821898	Efcab7	2,14E-14	0,734007734	-1,03	1,02	1,91
A_42_P822638	Tsc22d3	6,98E-15	0,705493854	1,06	1,34	1,30
A_42_P822726	Myo1g	5,02E-12	0,692908712	1,08	1,32	1,80
A_42_P824369	Foxf1	6,48E-22	0,882136732	1,55	3,70	13,34
A_42_P827974	Phf14	3,08E-29	0,900094294	-1,01	1,05	-1,39
A_42_P828242	Lgr4	9,43E-12	0,685275637	1,28	1,13	-1,11
A_42_P834031	Hrasls	4,59E-12	0,652269679	-1,07	2,00	2,59
A_42_P841704	Sh3tc1	3,30E-34	0,940190363	-1,16	1,38	6,34
A_42_P842823	Reep6	5,65E-09	0,637185947	1,29	1,01	-1,04
A_43_P10011	Pnisr	1,95E-13	0,668011469	1,23	1,16	-2,74
A_43_P10047	Slc20a2	7,41E-14	0,721247418	1,27	1,05	1,72
A_43_P10102	Spry4	1,06E-17	0,814599362	1,24	-1,39	-7,91
A_43_P10133	Spns2	7,25E-28	0,92405592	1,25	1,27	2,19
A_43_P10136	Amz2	6,43E-12	0,669859485	1,10	1,04	-1,19
A_43_P10225	Fgfr2	2,72E-11	0,628562077	1,22	1,01	-1,06
A_43_P10290	Tspan1	5,82E-29	0,907871474	-1,14	1,27	3,86
A_43_P10292	St6galnac4	4,32E-11	0,665989363	-1,08	1,14	2,08
A_43_P10498	Bag2	6,17E-28	0,924520993	-1,01	-1,17	-3,09
A_43_P10689	Synm	2,77E-18	0,757915033	1,05	1,27	1,88
A_43_P10824	Cib2	5,79E-10	0,630169569	1,11	1,08	-1,25
A_43_P10935	RGD1562339	2,49E-09	0,649420917	1,26	1,12	-1,50
A_43_P11044	G0s2	1,50E-20	0,855785142	1,45	1,27	-2,46
A_43_P11116	Prss30	9,77E-13	0,671614147	-1,10	1,56	2,99
A_43_P11136	Col9a1	4,74E-12	0,673668725	1,05	1,13	2,04
A_43_P11144	Pdrg1	5,77E-10	0,608049886	-1,25	-1,05	1,24
A_43_P11146	Lyplal1	6,87E-13	0,652589017	1,05	1,25	3,01
A_43_P11259	Mrpl19	3,25E-14	0,688722186	1,04	-1,10	-2,05
A_43_P11268	Cdkn1b	1,83E-11	0,608922725	1,15	-1,03	-1,60
A_43_P11380	Ncapd3	7,89E-11	0,613551103	-1,05	1,13	2,33
A_43_P11441	Atp4b	1,80E-19	0,853197569	-1,24	1,84	8,27
A_43_P11443	C4bpa	6,13E-26	0,901697378	1,90	2,08	8,10
A_43_P11471	Grin2c	4,61E-23	0,910101011	1,52	1,34	2,85
A_43_P11472	Hmox1	3,61E-09	0,602466186	1,40	-1,32	-1,01
A_43_P11476	Inha	6,98E-11	0,638455962	1,21	1,43	2,12
A_43_P11527	Adm	4,16E-18	0,807159068	1,83	1,35	-1,36
A_43_P11560	Aqp5	1,97E-12	0,783598759	-1,84	1,18	2,79
A_43_P11563	Cnr1	1,94E-11	0,633168463	1,57	1,64	4,11
A_43_P11580	Abcc2	1,21E-13	0,734328621	-1,30	2,43	3,64
A_43_P11616	Atf3	2,58E-17	0,845844687	2,97	1,40	2,58
A_43_P11654	Pnoc	2,03E-18	0,838601154	1,83	1,03	-2,83
A_43_P11683	F3	1,54E-15	0,721052092	1,63	1,58	2,32
A_43_P11684	Alpl	1,72E-13	0,669467699	1,44	1,32	1,08
A_43_P11685	Id2	4,67E-17	0,830209422	1,57	1,41	2,69
A_43_P11754	Akr7a3	2,24E-21	0,827800924	-1,08	1,50	3,77
A_43_P11820	Kcnj8	1,31E-13	0,750344385	1,09	1,08	1,23
A_43_P11861	Dio3	1,36E-24	0,898876428	1,05	1,74	5,67
A_43_P11901	Kcnj5	7,45E-31	0,935482859	1,14	1,75	11,55
A_43_P11906	Kcnab2	8,32E-17	0,727237374	1,13	1,23	2,18
A_43_P11932	Nr4a3	1,78E-34	0,961556291	-2,09	-13,03	-51,54
A_43_P11994	Cacng1	3,56E-18	0,775713775	1,11	2,29	4,15
A_43_P12018	Kcnn1	1,82E-16	0,760713135	-1,39	1,33	6,30
A_43_P12023	Nr4a2	4,96E-15	0,748295786	1,32	-4,23	-5,37

A_43_P12073	Uts2r	8,63E-15	0,742990395	-1,20	1,72	2,41
A_43_P12090	Pold1	8,10E-17	0,799578825	-1,10	1,55	2,36
A_43_P12097	Gdf9	2,79E-17	0,737491556	-1,41	1,92	6,71
A_43_P12151	Nrbf2	1,45E-11	0,612174993	1,16	-1,38	-2,46
A_43_P12160	Unc5b	2,88E-12	0,634166721	1,14	1,35	3,69
A_43_P12535	Klf10	2,06E-25	0,897155155	2,36	4,08	6,67
A_43_P12559	Camkk2	9,77E-13	0,671617743	-1,11	1,25	2,41
A_43_P12584	Nfyb	3,65E-13	0,739998929	1,12	1,25	1,27
A_43_P12618	Nr1h3	7,26E-18	0,803093157	-1,21	1,25	2,17
A_43_P12619	Nr4a3	1,27E-24	0,879762397	1,44	-2,42	-26,95
A_43_P12768	Bhlhe40	6,61E-14	0,655211598	1,24	-1,11	-2,16
A_43_P12769	Igfals	3,10E-16	0,714359449	1,10	1,75	4,10
A_43_P12821	Spa17	9,07E-12	0,618691856	-1,39	-1,08	2,13
A_43_P12825	Entpd6	2,69E-10	0,641170114	1,20	-1,04	-1,32
A_43_P12827	Nme3	1,04E-14	0,758335556	1,06	-1,03	-1,52
A_43_P12865	Nudt4	2,96E-11	0,602068027	1,16	1,10	-1,53
A_43_P12908	Klf4	1,21E-23	0,907266589	1,58	-1,29	-1,12
A_43_P12915	Bcl2l10	2,89E-24	0,876087524	1,47	2,80	3,65
A_43_P12924	Abcg5	1,98E-17	0,795520142	-2,08	1,79	6,10
A_43_P12927	Dusp1	5,16E-21	0,849908009	1,16	-2,41	-1,59
A_43_P12967	Cap2	1,96E-14	0,783444359	1,17	1,01	-1,30
A_43_P12995	Kcns1	2,02E-18	0,760587825	-1,52	1,69	5,59
A_43_P13023	Neu3	1,35E-14	0,71965811	-1,02	1,73	4,67
A_43_P13029	Acsl3	2,05E-12	0,662504383	1,15	1,00	-2,00
A_43_P13041	Nr0b2	6,74E-11	0,660117737	1,10	1,33	1,78
A_43_P13057	Baiap2	7,82E-13	0,650961657	1,25	1,24	1,83
A_43_P13083	Tacr2	7,75E-18	0,769355487	-1,68	1,61	4,67
A_43_P13109	Cyp26a1	1,97E-13	0,74629897	1,29	1,53	-1,13
A_43_P13170	Tmlhe	1,39E-20	0,831014296	1,04	1,06	-1,95
A_43_P13221	Ppp6c	1,04E-15	0,70195687	1,03	-1,27	-1,68
A_43_P13290	Pbld1	3,02E-22	0,865016362	-1,01	1,29	2,38
A_43_P13355	Bambi	1,21E-16	0,781136286	1,22	-1,64	-1,87
A_43_P13418	Kcnk2	2,80E-11	0,650828656	-1,24	1,01	2,21
A_43_P13859	LOC102548162	1,74E-18	0,761855255	-1,09	1,19	1,82
A_43_P13906	Zmiz1	4,46E-12	0,628343167	1,06	1,21	2,09
A_43_P13931	Grem2	7,78E-11	0,636960134	1,53	1,44	1,89
A_43_P14012	Mlh3	7,26E-13	0,624967896	1,07	1,17	1,99
A_43_P14131	Rgs2	3,39E-12	0,656142246	1,42	-1,15	-1,69
A_43_P14163	Odc1	1,52E-22	0,84362954	1,24	1,50	1,89
A_43_P14558	Klk1b3	6,56E-19	0,769851542	-1,62	1,25	4,62
A_43_P14648	Art3	1,88E-12	0,684947845	1,45	1,41	-1,85
A_43_P14687	Fez2	1,51E-10	0,627667566	1,22	1,11	-1,51
A_43_P14690	Cdk17	1,81E-12	0,640206616	1,04	-1,01	1,40
A_43_P14726	Zc3h4	2,19E-13	0,640409645	-1,03	1,20	3,12
A_43_P14752	LOC102554727	8,04E-13	0,714043632	-1,09	1,35	2,24
A_43_P14796	Apcdd1	3,14E-21	0,809190566	1,13	1,27	1,91
A_43_P14802	Ncoa6	5,14E-14	0,683551966	-1,01	-1,12	-1,52
A_43_P14809		1,87E-09	0,633808146	-1,24	-1,39	-1,06
A_43_P14849	Azi2	2,42E-17	0,738781079	1,05	-1,04	-1,81
A_43_P14872	Sdc1	2,79E-21	0,840669296	1,08	1,37	3,62
A_43_P14882	Errfi1	8,14E-21	0,847325477	1,91	1,45	-1,18
A_43_P14911	Il1b	2,68E-14	0,765574431	1,79	1,51	-1,03
A_43_P14954	Sos1	7,20E-20	0,834358412	-1,02	-1,19	-1,86
A_43_P14977	Tsc22d1	7,74E-13	0,651082153	-1,06	-1,21	-1,28
A_43_P15021	Slc25a29	1,61E-26	0,897483223	-1,04	1,20	1,97
A_43_P15024	Sypl2	8,10E-20	0,786140688	1,57	1,67	7,38
A_43_P15154	Ihh	2,29E-18	0,81143918	-1,00	1,74	3,72
A_43_P15205	Srf	2,41E-21	0,85412917	1,38	1,44	1,29
A_43_P15246	Pthlh	1,65E-31	0,944745077	-1,10	-1,27	-8,22
A_43_P15253	Icam1	5,50E-17	0,771031514	1,28	-1,06	1,35
A_43_P15362	Slc9a5	8,55E-12	0,644144451	1,36	1,22	-1,71
A_43_P15390	Smad7	2,05E-14	0,783034366	1,01	1,55	-1,02
A_43_P15406	Grm4	4,06E-15	0,731765389	1,12	1,04	1,56
A_43_P15424	Rapgef3	9,57E-14	0,650710888	-1,56	1,05	4,04
A_43_P15427	Sct	7,36E-21	0,84789863	-1,42	1,78	4,38
A_43_P15447	Bglap	1,21E-15	0,743496572	1,07	1,16	3,02
A_43_P15544	Ltb4r	1,70E-11	0,609931184	-1,55	1,49	4,54
A_43_P15548	Gabra6	3,08E-13	0,741748159	1,00	1,27	-1,28
A_43_P15701	Rab13	5,23E-11	0,642432356	-1,13	1,04	1,26
A_43_P15805	Mlnr	1,06E-16	0,746658425	1,31	1,30	-1,00
A_43_P15824	Acvr2b	2,43E-11	0,630104835	1,03	1,11	1,64

A_43_P15837	Ntrk2	5,08E-13	0,736576686	1,26	2,07	2,01
A_43_P15933	Chrm4	2,32E-12	0,720167366	1,20	-1,22	-3,09
A_43_P16232	Htr5b	1,17E-20	0,800175846	-1,13	2,49	2,10
A_43_P16449	Pelo	5,38E-11	0,682635509	1,02	-1,29	-1,70
A_43_P16491	Col16a1	9,36E-24	0,891163634	1,08	1,14	1,66
A_43_P16529	Gadd45b	2,49E-17	0,793753291	1,40	1,20	1,66
A_43_P16567	Glyctk	1,07E-21	0,832333544	-1,23	1,34	3,70
A_43_P16965	Saysd1	8,49E-14	0,652167259	-1,12	1,46	2,60
A_43_P16989	Snx9	1,68E-10	0,626125629	1,23	1,22	-1,33
A_43_P17060	Them5	4,81E-18	0,773266417	-1,15	2,57	3,47
A_43_P17061	Pcgf3	1,83E-10	0,601236514	1,37	1,04	-1,31
A_43_P17271	Eva1c	5,71E-11	0,618176986	1,50	1,04	2,96
A_43_P17298	Naaa	8,42E-15	0,743228901	1,05	1,88	3,79
A_43_P17330	Nup35	4,01E-15	0,731895379	-1,06	-1,18	-1,42
A_43_P17405		2,47E-21	0,841388964	-1,21	1,77	3,12
A_43_P17786	Setmar	1,32E-16	0,744660847	-1,13	1,26	2,44
A_43_P17925	Abca8	1,72E-11	0,657229861	-1,07	1,15	1,36
A_43_P18332	Lrfn2	2,49E-26	0,920592462	1,13	-1,01	7,62
A_43_P18356	Ift57	7,48E-10	0,604113042	1,16	1,13	-1,09
A_43_P18571	Tekt4	2,37E-18	0,824971662	1,18	2,43	2,50
A_43_P18638	Asb18	1,06E-14	0,740982854	1,30	1,12	1,79
A_43_P19279	Lyzl4	4,03E-27	0,902538826	-1,70	1,25	26,14
A_43_P19743	Krtcap3	5,30E-18	0,819487294	1,47	1,77	1,39
A_43_P19848	RGD1309870	8,17E-12	0,64474308	-1,48	-1,13	1,58
A_43_P19889	Angptl6	3,20E-11	0,60093684	1,06	1,16	1,74
A_43_P19891	Rrh	2,29E-25	0,905495126	1,63	2,35	2,49
A_43_P20486		8,18E-19	0,787236595	1,56	4,23	6,17
A_43_P20515	Zfand1	9,57E-12	0,664822281	-1,08	1,18	1,67
A_43_P20684	Tceanc2	3,64E-14	0,728633743	-1,33	1,38	2,42
A_43_P20963	Wdr86	3,37E-21	0,839555377	1,10	1,33	2,72
A_43_P21118	Klhl24	7,44E-14	0,721201993	1,54	1,23	1,40
A_43_P21176	Glce	3,43E-14	0,66305607	-1,19	1,26	2,03
A_43_P21279	Pip5k1b	6,66E-10	0,605872172	-1,26	1,01	1,21
A_43_P21414	Atg10	2,06E-16	0,740519411	-1,37	1,27	3,21
A_43_P21913	Usp53	8,24E-13	0,694626213	1,86	1,15	-1,70
A_43_P21945	Ppl	5,61E-14	0,704515613	-1,06	-1,09	2,88
A_43_P22225	Mdga1	3,27E-22	0,87538247	-1,54	1,02	3,74
A_43_P22501	LOC100909860	1,49E-18	0,799421987	1,09	1,41	1,96
A_43_P22555	Fam189a2	9,92E-25	0,890949219	1,23	-1,13	-3,42
A_43_P22744	Adcy9	2,03E-11	0,607421381	1,24	-1,11	-2,16
A_43_P22811	Cd6	1,40E-22	0,927130608	-1,35	2,32	13,37
A_43_P22825	Cabyr	2,26E-16	0,758818104	1,08	2,06	3,56
A_43_P22864	Rad54l	3,70E-29	0,925398513	-1,01	1,63	2,76
A_43_P23014	Lpxn	4,18E-11	0,622577919	1,05	1,14	3,06
A_43_P23152	Rhoh	2,98E-11	0,650007134	-1,28	-1,11	4,20
A_43_P23155	Mcm10	6,18E-11	0,617037221	1,15	2,12	3,01
A_43_P23187		8,58E-16	0,780526318	1,23	1,15	1,32
A_43_P23274	Dmrtc1a	2,87E-18	0,777445995	1,06	1,53	3,45
A_44_P1000400	Safb2	1,04E-12	0,670809483	1,11	-1,05	-1,48
A_44_P1001782	Myf6	4,17E-13	0,65876667	1,16	1,25	-3,07
A_44_P1003794	Nr3c1	6,73E-13	0,676118417	-1,08	-1,02	1,45
A_44_P1004376	Ins2	2,16E-15	0,737951764	-1,46	1,50	2,75
A_44_P1004790	Slc30a2	1,36E-17	0,764672275	-1,40	3,62	4,05
A_44_P1004840	Tnfsf9	7,51E-10	0,604055585	1,06	1,21	-1,39
A_44_P1006068	Exoc3l4	2,53E-18	0,824547262	1,20	1,53	4,22
A_44_P1006261	Nme4	1,59E-17	0,781208651	-1,01	2,18	2,63
A_44_P1007215	Upp1	4,27E-12	0,694842222	-1,07	1,01	1,45
A_44_P1011386	Znhit6	1,49E-09	0,67538751	1,14	1,03	-2,50
A_44_P1011464	Rhobtb1	1,97E-12	0,663000237	1,22	1,46	-1,70
A_44_P1011953	Gpcpd1	6,95E-12	0,646865115	1,27	1,48	1,52
A_44_P1012556	Stbd1	8,51E-10	0,700405534	-1,28	-1,22	1,13
A_44_P1012606	Nup50	4,35E-12	0,600661436	1,16	-1,09	-1,73
A_44_P1012940	RGD1306072	4,61E-12	0,627887891	-1,25	1,29	2,15
A_44_P1014018	Mex3d	4,88E-14	0,759963918	-1,19	-1,68	-2,43
A_44_P1014659	Plek2	1,09E-31	0,926400336	1,16	1,89	6,85
A_44_P101533	St6galnac5	2,17E-22	0,877313431	-1,16	1,11	3,94
A_44_P1015388	Ankrd24	1,32E-12	0,644326878	-1,16	1,16	1,93
A_44_P1015754	Slc22a3	1,43E-16	0,822389127	1,14	1,47	2,44
A_44_P1015839	Mroh7	1,86E-26	0,921491231	1,38	1,60	6,10
A_44_P1016170	Prpf4b	4,91E-27	0,892020203	1,08	-1,01	-2,18
A_44_P1016691	Fhl4	4,38E-27	0,932236694	1,24	3,50	6,02

A_44_P1016829	Trib1	1,37E-18	0,783273152	2,48	-2,81	-5,63
A_44_P1017206	Podnl1	7,66E-14	0,653416861	-1,04	2,02	2,23
A_44_P1017458	Spata20	9,94E-23	0,830963082	-1,08	1,15	2,54
A_44_P1017595	Mospd4	1,32E-09	0,618010056	1,31	1,90	1,81
A_44_P1017720	Ankrd34a	3,20E-18	0,776574985	1,29	1,06	-2,27
A_44_P1018090	Lgi4	5,61E-22	0,849755462	-1,25	1,30	2,79
A_44_P1018447	Micall2	9,39E-11	0,65567414	1,06	-1,06	1,57
A_44_P1018622	Rflnb	5,57E-18	0,772083308	1,28	1,35	2,57
A_44_P1018696	Muc19	2,91E-12	0,679656348	-1,24	1,13	2,01
A_44_P1019407	Rnf103	5,99E-11	0,640568409	1,07	1,09	1,61
A_44_P1019480	Cdc42ep2	5,00E-15	0,765090953	-1,06	1,17	2,68
A_44_P1019726	Leng9	1,40E-11	0,612682609	1,11	1,47	1,66
A_44_P1021808	RGD1561796	4,93E-08	0,622600385	-1,33	1,16	1,46
A_44_P1022395	Fbxl3	1,23E-16	0,764122017	1,02	-1,15	-1,21
A_44_P102369	Socs1	8,35E-13	0,69447158	-1,11	1,35	2,30
A_44_P1024065	Hdac5	5,36E-21	0,881850931	1,10	-1,39	-2,78
A_44_P1024627	Kcnv1	2,78E-23	0,886552252	1,34	1,09	-4,82
A_44_P1025790	Gsto1	2,71E-13	0,664017486	-1,02	1,02	-1,17
A_44_P102774	Polr3e	3,24E-12	0,604759344	1,06	-1,27	-2,34
A_44_P1028743	Cfc1	1,67E-22	0,867959854	1,45	3,98	11,13
A_44_P1029253	Mas1	1,63E-11	0,657975451	-1,44	-1,12	4,25
A_44_P1029805	Krt7	1,19E-29	0,935019361	1,19	1,16	4,60
A_44_P1030258	Cnr1	2,17E-17	0,778723157	-1,15	1,20	2,25
A_44_P103127		2,80E-25	0,895974094	1,06	1,12	3,77
A_44_P1033459	Uxs1	2,28E-13	0,688827206	-1,03	-1,05	-1,47
A_44_P1034541	Pim3	3,65E-18	0,822059173	-1,13	-1,60	-2,54
A_44_P1034637	Uros	1,11E-14	0,676144411	-1,26	1,21	3,60
A_44_P1034668	Shroom1	1,42E-25	0,898565077	1,52	2,80	8,41
A_44_P1035007	Chrne	9,07E-22	0,83330377	1,18	1,18	2,13
A_44_P1035071	Nmt2	1,42E-11	0,699109593	1,27	1,07	-1,12
A_44_P1035706	Lyrm9	1,22E-12	0,645358147	-1,28	1,12	1,60
A_44_P1037041	Map3k6	1,13E-27	0,897559618	-1,18	1,65	3,53
A_44_P1037706	Glod5	4,46E-20	0,860991454	1,42	1,19	8,82
A_44_P1037806	Hpx	4,06E-27	0,902509589	-1,55	1,48	10,97
A_44_P1038028	Tnfrsf12a	5,97E-14	0,703837029	1,22	-1,14	1,20
A_44_P1039213	Cpa2	1,77E-14	0,695455461	-1,25	1,20	2,47
A_44_P1039809	Rasip1	1,40E-12	0,707833068	-1,27	1,02	1,67
A_44_P1039927	Eepd1	1,32E-17	0,764915506	-1,04	-1,35	-4,32
A_44_P104054	Crhbp	4,65E-31	0,942537979	-1,09	1,08	1,27
A_44_P1042696	Smim29	1,62E-16	0,761781466	-1,15	-1,27	-1,27
A_44_P1043302	Crb3	2,94E-15	0,785187838	1,39	1,74	2,56
A_44_P1044030	Klhdc9	2,01E-27	0,921057741	1,18	1,98	3,62
A_44_P1044046	Ndufaf1	5,45E-25	0,893355505	1,10	1,88	2,65
A_44_P1044565	Cdadc1	1,46E-14	0,755187181	1,05	-1,26	-1,91
A_44_P1044984	Atg16l2	3,88E-13	0,682634306	1,17	1,16	1,33
A_44_P1045612	Fxyd3	1,15E-23	0,843289786	-1,36	1,61	6,06
A_44_P1045652	Gnb1l	1,63E-13	0,692626754	1,04	1,04	2,05
A_44_P1045734	Stac3	7,10E-14	0,771845705	-1,83	2,13	2,08
A_44_P104652	Slc38a2	2,80E-29	0,910245219	1,11	-1,23	-2,73
A_44_P1047924	Herpud1	9,88E-22	0,898196144	1,31	-1,12	-1,10
A_44_P1048999	Rhpn1	7,51E-14	0,679202713	-1,02	-1,13	1,58
A_44_P1050015	Vcpip1	2,14E-11	0,606686241	1,23	-1,39	-1,49
A_44_P1050200	C1qtnf12	1,91E-17	0,823802738	1,76	1,75	1,79
A_44_P1050510		9,97E-20	0,81836946	-1,07	2,19	3,53
A_44_P1053605	Hcn1	1,64E-13	0,778756892	1,36	-1,05	-1,34
A_44_P1053951		1,24E-26	0,915415157	1,35	1,71	5,76
A_44_P1055082	Dyrk2	1,23E-18	0,800868363	-1,17	-1,33	-1,56
A_44_P1056589	Fxyd7	6,85E-16	0,729074382	-1,12	1,03	1,76
A_44_P1057412	Ddx25	8,86E-16	0,764115857	-1,13	1,13	1,46
A_44_P105749	Rimbp3	4,69E-27	0,918472924	-1,04	5,01	8,29
A_44_P1058112	Yars2	8,46E-18	0,801954885	-1,18	-1,59	-7,34
A_44_P1058570	Sigirr	5,32E-17	0,80278447	-1,48	1,62	3,95
A_44_P1059903	Lzts1	5,04E-28	0,925095734	1,11	1,00	-2,69
A_44_P1060096	Cideb	1,05E-21	0,816384184	1,26	2,52	6,06
A_44_P108003	Nipal2	3,51E-17	0,774772107	1,01	1,04	3,31
A_44_P109927	Ahsa2	2,17E-11	0,654232207	-1,17	-1,05	-1,83
A_44_P112082	Ccdc89	1,31E-11	0,681242714	1,55	2,16	2,74
A_44_P114638	Tsen2	9,78E-12	0,617663037	-1,05	1,09	1,26
A_44_P114692		4,14E-17	0,804673899	1,01	1,24	1,38
A_44_P114788		9,33E-11	0,611134308	-1,31	-1,06	1,85
A_44_P114900	March10	2,17E-12	0,610310041	-1,25	-1,01	7,52

A_44_P115192	Slc5a5	1,31E-09	0,694691105	1,42	1,49	1,42
A_44_P116283	Klhl41	1,96E-13	0,710811997	1,40	1,52	-1,37
A_44_P117119	Hpdl	2,33E-16	0,791256331	1,17	2,16	1,07
A_44_P117216	RGD1310852	9,23E-13	0,621793849	-1,46	1,44	1,59
A_44_P118666	Hs3st1	1,07E-22	0,858602714	1,20	-1,01	-1,98
A_44_P118724	Arc	3,20E-24	0,89552772	5,53	3,58	4,24
A_44_P119177	Parp2	1,94E-11	0,608093873	1,06	1,39	2,11
A_44_P119575		2,07E-11	0,607152322	1,12	1,56	3,28
A_44_P122386	Hspb3	2,31E-18	0,811368271	-1,29	-1,26	-7,85
A_44_P123033	Klhl5	2,30E-15	0,717032584	1,28	1,14	-1,24
A_44_P125733	Madcam1	9,17E-12	0,618548912	1,00	1,07	1,69
A_44_P126154	Cdc37l1	6,31E-24	0,892784484	1,09	-1,00	-2,04
A_44_P127597	Dnajb9	3,77E-11	0,667761118	-1,12	-1,33	1,16
A_44_P127749	Tmem150c	1,08E-20	0,83257793	1,00	1,10	1,76
A_44_P128062	Syt17	1,07E-10	0,692277315	1,08	-1,13	1,56
A_44_P128488	Pbx4	1,74E-13	0,669356452	-1,21	1,06	2,00
A_44_P131470	Hexb	9,80E-16	0,779399882	1,20	-1,04	-1,50
A_44_P132610	Slc20a1	5,70E-12	0,671362773	1,23	-1,07	-1,30
A_44_P133335		1,97E-25	0,906037183	1,02	1,04	2,58
A_44_P134634		1,67E-14	0,671473958	-1,07	1,85	2,37
A_44_P135224	Plk3	5,05E-10	0,652686008	-1,21	-1,51	1,03
A_44_P135356	RGD1565844	3,66E-16	0,787613829	1,13	-1,33	-1,13
A_44_P137633	Adora1	1,86E-11	0,608693553	1,02	1,35	2,44
A_44_P138301	B9d1	3,18E-10	0,616902601	1,01	1,13	-1,45
A_44_P138355		4,75E-10	0,610943453	1,00	-1,22	1,34
A_44_P140148	Cd248	1,63E-15	0,740632439	-1,17	1,08	1,75
A_44_P142242	Cdh1	1,07E-15	0,7446511	1,51	1,01	1,72
A_44_P144277	Pkn1	1,62E-10	0,603068155	1,01	-1,16	-1,78
A_44_P149510	Numbl	4,11E-12	0,601454884	-1,13	1,71	4,77
A_44_P151482	Il22ra2	2,59E-11	0,629230568	1,57	2,31	3,06
A_44_P151582	Il17re	1,68E-16	0,742415305	-1,04	1,25	2,87
A_44_P152275		3,57E-10	0,615164069	1,37	1,10	3,73
A_44_P156262	Myod1	1,07E-20	0,832630999	-1,13	2,95	3,75
A_44_P156589	Ngef	8,37E-14	0,65234442	-1,19	1,03	1,81
A_44_P156966	Ube2q2l	7,25E-18	0,817314131	1,30	1,53	2,04
A_44_P159171	Mylip	4,46E-14	0,685172481	-1,07	-2,58	-3,46
A_44_P160846	Rnasel	1,81E-20	0,829390681	1,35	1,67	4,70
A_44_P161220	Brinp1	4,00E-16	0,753715018	1,25	1,06	-1,37
A_44_P161770		5,94E-12	0,670849458	-1,08	1,49	1,68
A_44_P163242	Epcam	7,65E-16	0,810010766	-1,12	1,08	2,18
A_44_P165643	Sptb	2,87E-16	0,715119851	-1,04	1,49	3,84
A_44_P165989	Idh2	1,78E-11	0,609328554	1,21	1,02	-2,10
A_44_P165999	Coq8a	3,74E-15	0,750965223	-1,06	-1,05	1,97
A_44_P173568	Pop1	1,95E-18	0,812547603	1,28	1,07	-1,84
A_44_P175875	Pigv	3,27E-11	0,648781715	-1,06	-1,45	-1,27
A_44_P179145	Dgkg	1,41E-10	0,628658868	1,41	1,14	1,87
A_44_P180268	Lrrc8d	4,21E-16	0,770630611	-1,02	1,23	2,20
A_44_P182807	Alg11	5,79E-15	0,683457534	-1,13	-1,25	-2,29
A_44_P184484	Orc1	6,00E-17	0,751766311	-1,08	1,50	2,71
A_44_P184685	Sertad1	1,80E-09	0,613276981	-1,13	-1,10	1,38
A_44_P185294		6,16E-32	0,946775522	1,27	1,10	4,02
A_44_P185355	Bcl11b	1,36E-14	0,719574777	-1,00	-1,03	2,61
A_44_P185441	Tnfrsf21	4,04E-13	0,659174482	1,02	-1,04	-3,26
A_44_P187195	C1galt1	3,81E-15	0,797316672	1,72	1,14	-1,94
A_44_P189406	Spint1	6,87E-10	0,605404501	1,04	1,05	2,34
A_44_P190229	Fam58b	2,83E-12	0,634376611	-1,09	1,41	1,63
A_44_P191285	RGD1564171	2,71E-17	0,758746636	1,18	1,44	2,53
A_44_P192083	Ffar4	7,29E-22	0,860495687	1,67	2,20	5,66
A_44_P192406	Dusp9	2,95E-27	0,926891303	-1,07	4,46	5,73
A_44_P194925	Pigx	4,56E-12	0,712513353	-1,20	1,07	-1,34
A_44_P195648	Ufm1	1,98E-10	0,600111923	-1,19	-1,07	-1,35
A_44_P196296	Plppr4	4,97E-12	0,626896599	1,04	1,04	-1,85
A_44_P196856	Cir1	5,05E-11	0,619897592	-1,07	-1,02	1,55
A_44_P198620	Nos3	1,42E-19	0,842876363	1,82	2,53	4,57
A_44_P198705	H1f0	5,44E-22	0,908525853	-1,19	1,63	2,88
A_44_P201028	Nfil3	2,11E-28	0,893119039	1,10	-3,98	-5,92
A_44_P201295	Mybpc2	9,41E-16	0,745830257	-2,02	-1,07	3,83
A_44_P202201	Grp	1,33E-22	0,879589088	-1,46	1,48	10,23
A_44_P203549	Egln3	1,13E-13	0,696731021	1,42	1,01	-1,22
A_44_P203859	Piezo2	9,07E-10	0,601177517	1,83	2,03	2,99
A_44_P204207	Smyd4	2,96E-10	0,617931362	-1,06	1,16	2,45

A_44_P205528	Ppp1r32	2,35E-20	0,79521553	1,14	-1,00	2,19
A_44_P206844	Hcrtr2	3,50E-11	0,647864896	1,30	1,36	-2,30
A_44_P210492	Syt2	6,59E-25	0,909833124	-1,29	1,07	4,12
A_44_P210736		7,49E-16	0,765607405	-1,18	-1,10	-1,69
A_44_P210898		1,32E-16	0,763520396	1,36	1,18	2,52
A_44_P211037	Adgrb2	2,32E-20	0,85338351	-1,12	-1,15	1,90
A_44_P211061	Etv1	1,88E-11	0,656076242	-1,31	-1,11	-1,31
A_44_P214288	Cabp7	1,00E-32	0,939117486	-1,41	1,57	8,73
A_44_P216395	Gadd45a	1,86E-17	0,779958724	1,08	1,10	1,31
A_44_P220481	Tmco4	1,28E-10	0,62994803	1,10	1,05	2,72
A_44_P220610	Fahd1	1,43E-12	0,643307906	1,04	-1,03	-1,45
A_44_P220861	Npff	6,65E-20	0,821044154	-1,01	1,39	2,19
A_44_P222004	Il15	1,99E-13	0,667778309	-1,01	1,03	2,10
A_44_P222124	Tssk3	2,17E-22	0,841636909	-1,24	3,54	6,90
A_44_P222533	Actrt3	2,23E-14	0,751116003	1,02	1,82	2,68
A_44_P224020	Kcna4	2,85E-31	0,943592452	1,25	-1,21	-5,91
A_44_P224547	Plcb1	3,06E-11	0,601589375	1,01	-1,05	-1,26
A_44_P226597	Pycard	1,49E-26	0,887646349	-1,25	1,07	-2,03
A_44_P226658	Scnn1g	3,60E-28	0,918813445	-1,28	1,87	18,40
A_44_P227221	Syde2	1,20E-19	0,876227354	1,26	2,66	2,41
A_44_P227409	Fam228a	3,76E-23	0,836631302	1,50	1,24	18,05
A_44_P229299	Mepce	8,25E-11	0,612917683	-1,01	1,03	2,32
A_44_P229677	LOC363337	1,56E-18	0,827755619	1,05	1,09	1,65
A_44_P230697	Igdcc4	1,96E-17	0,779535605	1,12	1,02	1,71
A_44_P231243	Insig2	9,33E-11	0,655766369	1,50	1,24	-1,12
A_44_P231583	Kcnip3	1,20E-22	0,869594075	-1,05	1,39	2,41
A_44_P231597	Hpcal1	8,90E-15	0,723905462	-1,09	-1,07	2,28
A_44_P233080	Egr1	4,99E-30	0,957373853	2,34	1,68	9,48
A_44_P234393	Shb	1,03E-19	0,818158934	-1,08	-1,28	2,80
A_44_P236407	Lnx1	8,62E-24	0,900365503	1,14	1,26	-1,30
A_44_P236600	Lrrtm3	5,03E-24	0,884141799	-1,00	-1,20	-1,79
A_44_P237621	Txnip	2,09E-22	0,896160474	1,20	3,35	10,51
A_44_P238204	Mid1ip1	5,05E-18	0,752768953	1,03	-1,14	-3,17
A_44_P238257	Trip10	7,13E-22	0,834732239	-1,09	-1,10	-1,32
A_44_P240696	Nppc	2,79E-20	0,873824691	-1,52	-2,07	1,16
A_44_P241190	Cwc27	1,75E-12	0,613200426	-1,15	-1,12	-1,65
A_44_P241277	Traf2	1,97E-15	0,695203045	-1,05	1,20	2,76
A_44_P241409	Vgll4	3,76E-17	0,80539583	1,20	1,10	1,67
A_44_P243004	Amigo2	8,10E-33	0,939592801	1,17	-1,19	-3,53
A_44_P243355	Atp10a	1,44E-20	0,844041242	-1,03	-1,15	-3,30
A_44_P243477		6,70E-15	0,818981266	-1,10	1,48	3,35
A_44_P243534	Pxdc1	5,49E-12	0,710365891	1,30	1,35	1,42
A_44_P245640	Sftpb	9,25E-17	0,747877811	-1,03	1,24	5,66
A_44_P246030	Gse1	8,09E-36	0,953038185	1,10	4,21	5,44
A_44_P246114	Tle1	1,00E-13	0,736212666	1,62	1,20	-1,38
A_44_P247880	Csf2	2,51E-24	0,876717145	1,44	1,16	1,23
A_44_P248090	Thbs2	1,91E-09	0,689639607	-1,18	-1,09	1,13
A_44_P250203	Rbm12	1,78E-25	0,906395956	-1,12	-1,37	-3,59
A_44_P250594	Uggt2	6,89E-12	0,62247025	1,49	1,61	1,38
A_44_P250620	Fhdc1	1,36E-14	0,6738598	1,52	-1,17	-2,16
A_44_P252483	Ackr3	1,31E-19	0,830618454	2,02	1,77	1,87
A_44_P252657	Pou6f1	8,50E-22	0,833692852	1,14	-1,72	-2,52
A_44_P252976	Gsto2	2,92E-22	0,824446718	1,46	1,72	3,11
A_44_P253208	Adamts9	2,01E-17	0,79543505	-1,05	-1,08	-1,42
A_44_P254016	Avp	2,22E-22	0,841494904	1,01	1,91	2,54
A_44_P256677	Plcxd3	1,07E-11	0,702506766	1,21	1,25	-1,37
A_44_P257681	Zfp78	8,66E-20	0,833208296	1,15	-1,57	-1,44
A_44_P260348		2,65E-15	0,692008548	1,04	1,28	2,14
A_44_P260725	Tefm	2,56E-12	0,635699283	-1,03	2,16	1,89
A_44_P260751	Nptx1	8,15E-28	0,923716062	1,92	2,36	2,23
A_44_P263440	Hbp1	1,53E-15	0,741262146	1,04	1,04	2,40
A_44_P265013	Pear1	1,65E-18	0,781831453	-1,01	1,31	2,34
A_44_P265434	Mif4gd	3,13E-11	0,601265045	1,05	1,60	1,86
A_44_P265544	Crybb3	4,49E-16	0,838485367	-1,76	1,42	2,04
A_44_P265709	Il2rb	3,45E-28	0,89124995	-2,32	3,60	23,28
A_44_P266776	Dpagt1	4,50E-12	0,600170368	-1,07	-1,12	-1,14
A_44_P267041	Lat	2,24E-11	0,631233025	-1,23	-1,02	1,66
A_44_P267199	Spred3	4,10E-16	0,77086586	1,45	1,11	-1,88
A_44_P269085	Chrna4	1,21E-13	0,673631157	-1,15	-1,10	2,55
A_44_P269522	Tmem183a	7,78E-10	0,625835734	1,11	1,01	-1,49
A_44_P269808	Slc9a8	4,80E-13	0,657050783	1,39	2,05	3,46

A_44_P269930	Mfsd2a	4,28E-10	0,634525367	1,05	1,29	1,25
A_44_P269984	Kdm4d	4,95E-15	0,729808658	-1,35	1,77	4,92
A_44_P271643	Nln	1,24E-21	0,815299141	-1,02	-1,23	-1,79
A_44_P271658	Reg3b	4,09E-16	0,75350531	-2,10	-1,28	-2,04
A_44_P273777	Masp1	2,43E-13	0,727011863	1,06	1,18	-1,74
A_44_P274762	Cpped1	3,31E-16	0,772707545	-1,24	1,02	2,32
A_44_P276087	Ampd1	2,02E-12	0,684043541	2,18	2,22	5,56
A_44_P276614		3,91E-15	0,711559851	-1,56	1,48	3,64
A_44_P277010	Imp3	3,19E-14	0,688940725	-1,04	-1,17	-1,46
A_44_P277669	Myo19	4,23E-10	0,612678568	1,31	1,21	-3,12
A_44_P277876	Tpst1	1,67E-13	0,643810654	-1,06	-1,05	1,60
A_44_P278387	Wipf3	1,09E-17	0,745994643	1,40	1,89	2,38
A_44_P278562	Mtfr2	3,79E-12	0,602573966	1,15	1,30	1,86
A_44_P279222	Faap20	1,18E-11	0,639844229	-1,24	1,43	2,06
A_44_P279337	Slc22a5	7,86E-25	0,881836841	1,30	1,51	2,47
A_44_P280786	Cyp2c11	1,99E-09	0,611735069	-1,21	-1,01	2,96
A_44_P282164	Pdia2	1,68E-27	0,921595318	1,31	2,40	1,82
A_44_P282853	Smarcd2	2,39E-21	0,827409571	1,12	1,62	2,53
A_44_P283176	Spats1	1,15E-17	0,799688798	-1,26	1,67	3,87
A_44_P283790	Mfhas1	3,06E-13	0,685406098	1,29	-1,04	-1,54
A_44_P284067		1,41E-10	0,670081942	1,12	1,01	-2,12
A_44_P284753	Ankrd1	1,78E-14	0,753266058	-1,08	1,54	3,37
A_44_P285694	Bfsp1	6,66E-19	0,769733529	-1,22	1,18	2,64
A_44_P287301	Bcl6	1,24E-15	0,792397826	1,04	1,22	-1,16
A_44_P287958	Dnmt3b	1,30E-24	0,899029071	1,19	1,72	1,97
A_44_P288241	Medag	8,07E-16	0,747262089	1,32	2,39	5,19
A_44_P288982	Zbtb8a	3,28E-16	0,736157867	1,19	1,20	1,93
A_44_P289125	Spsb4	4,69E-20	0,823318534	-1,97	1,72	3,12
A_44_P289173	Grtp1	2,61E-13	0,638196691	-1,06	-1,16	1,69
A_44_P289445	Parp16	1,44E-18	0,782857482	1,03	2,30	1,84
A_44_P290168	Fah	3,47E-23	0,864288637	-1,01	1,19	2,82
A_44_P290187	Sfrp4	1,89E-13	0,729657452	1,77	1,87	2,51
A_44_P291028	Arhgef19	1,57E-17	0,837492777	1,35	1,59	3,20
A_44_P291102	Smpd5	2,22E-17	0,760484325	-2,10	2,05	4,15
A_44_P292437	Riox2	4,11E-14	0,727396714	-1,05	1,55	1,44
A_44_P292510	Slc2a3	2,32E-15	0,716938689	-1,26	-2,69	-2,41
A_44_P292980	Slitrk1	1,87E-14	0,752840373	1,40	-1,08	-1,19
A_44_P293315	Bbs4	2,28E-09	0,609636454	1,44	1,10	-1,37
A_44_P293357	Ccdc51	9,56E-14	0,676410153	-1,04	1,47	1,61
A_44_P295789	Comp	4,92E-22	0,836919947	-1,02	1,16	3,49
A_44_P296180	Arg2	1,95E-11	0,655594557	-1,14	1,02	-2,06
A_44_P297633	Irx3	2,29E-26	0,896152124	1,03	-1,03	-3,16
A_44_P298331	Gsdma	2,04E-17	0,795302633	-1,39	-1,18	6,53
A_44_P302536	Pnn	4,74E-12	0,67365655	1,01	-1,49	-2,72
A_44_P303883	Tbx2	2,92E-15	0,735020012	1,03	1,13	1,48
A_44_P303982	Susd2	1,21E-18	0,784250201	-1,43	1,13	5,23
A_44_P305801	Jakmip1	6,77E-31	0,935711575	1,17	1,54	6,37
A_44_P306204	Tph1	4,26E-30	0,90679587	-1,34	3,27	15,37
A_44_P306602	Irf1	4,54E-10	0,63370084	-1,02	1,47	3,38
A_44_P306664	Tbxa2r	2,48E-26	0,895847601	-1,10	1,10	5,44
A_44_P307181		5,33E-19	0,834702864	2,58	3,69	9,03
A_44_P307559	Chrna3	7,71E-17	0,768169437	-1,56	1,67	4,69
A_44_P307978	Cited2	3,72E-13	0,683141076	-1,17	-2,50	-3,10
A_44_P309081	Hspa2	1,47E-15	0,74163209	-1,31	1,05	3,23
A_44_P309455	Fam207a	7,45E-15	0,744415627	-1,10	-1,02	1,20
A_44_P309780	Dnajb1	2,10E-14	0,6935995	1,03	-1,29	-1,66
A_44_P311106	Pou3f3	2,52E-18	0,778481314	-1,02	-1,23	-2,34
A_44_P311620	Ahnak	2,16E-07	0,617074443	1,07	-1,23	1,44
A_44_P311917	LOC679711	1,48E-21	0,878289098	1,13	1,09	2,14
A_44_P313272	Slc6a13	8,95E-12	0,704645209	1,47	1,72	2,15
A_44_P313542	Sgk1	2,17E-13	0,6405335	1,03	-1,83	-2,55
A_44_P314295	Zmynd12	3,81E-26	0,903423721	-1,07	3,21	7,23
A_44_P315905	Acr	3,06E-16	0,77336815	-1,08	1,74	2,54
A_44_P316319	Shkbp1	4,30E-16	0,770455353	-1,09	1,25	1,77
A_44_P316342	Det1	1,43E-13	0,69414636	-1,18	1,48	1,90
A_44_P316611	Pknox1	8,06E-13	0,673939582	1,62	1,45	1,53
A_44_P317388	Mcam	1,57E-22	0,868263472	1,15	1,16	1,91
A_44_P318662	Tac3	1,31E-32	0,93853331	-1,33	1,71	6,12
A_44_P320752	Rasl11a	1,86E-23	0,878340096	1,48	1,95	3,93
A_44_P321329	Polr2c	2,38E-12	0,660636846	1,04	1,02	-1,72
A_44_P321510	Slc25a33	1,30E-12	0,689261528	1,26	1,17	-2,42

A_44_P321605		1,07E-13	0,717311696	-1,07	1,24	1,53
A_44_P322860	Camkk1	6,16E-12	0,623993167	1,14	1,20	1,93
A_44_P323404	Cntf	4,28E-13	0,720968323	1,17	1,41	1,26
A_44_P324430	Mdh1	3,99E-10	0,613540964	-1,01	1,09	-1,13
A_44_P325189	Rcan2	4,96E-16	0,769212532	1,15	-1,06	-2,61
A_44_P325560	Apom	9,38E-23	0,859271065	1,18	1,37	3,01
A_44_P327992	LOC100360244	1,64E-17	0,796990093	-1,08	1,46	2,42
A_44_P328046	Masp2	2,35E-23	0,896404427	-1,86	2,30	5,66
A_44_P328283	Fan1	1,07E-11	0,641146318	1,30	1,50	2,49
A_44_P328489	Rprd1a	1,39E-09	0,617152777	1,31	1,13	-1,95
A_44_P330188	Acox2	2,32E-17	0,794301619	1,67	2,98	3,67
A_44_P330594	Prkd2	2,25E-09	0,631001934	1,46	2,58	1,33
A_44_P330847	LOC690467	1,06E-30	0,94070936	1,66	1,59	6,01
A_44_P332896	Kctd14	4,92E-19	0,791083567	-1,38	1,36	2,83
A_44_P333078	Adamtsl2	2,39E-25	0,896589689	-1,04	1,52	5,51
A_44_P334715	Klc3	2,08E-15	0,772896884	1,25	-1,17	1,91
A_44_P334736	Edn1	2,60E-16	0,757568535	1,55	1,13	1,30
A_44_P335079	Tff3	2,99E-23	0,90386701	-1,43	1,28	2,97
A_44_P335446	Dusp2	1,09E-23	0,899460386	1,10	2,05	2,11
A_44_P335578	LOC108348348	1,55E-16	0,779101875	-1,11	-1,10	3,62
A_44_P337300	Il4r	1,10E-12	0,670133972	-1,18	-1,22	-1,38
A_44_P337335	Ctrc	1,63E-20	0,843300817	1,00	-1,03	4,82
A_44_P337351	Cxcl12	3,50E-19	0,824251053	1,04	1,50	2,49
A_44_P338068	Ccdc134	6,55E-25	0,871354401	-1,14	1,39	3,02
A_44_P339535	Tfpi2	5,03E-12	0,651067762	1,30	1,20	1,51
A_44_P340357	Rnf113a2	2,99E-19	0,794775195	1,03	1,41	1,69
A_44_P341870	Prkag2	2,02E-16	0,77687687	1,11	1,16	1,91
A_44_P344181	Map6	1,35E-11	0,66036737	1,10	-1,15	-1,33
A_44_P346408	Egfr	3,12E-11	0,601311583	1,23	1,09	1,23
A_44_P346832	Tesc	7,40E-18	0,842308436	-1,62	1,01	2,68
A_44_P346913		2,16E-19	0,827387405	-1,17	-1,12	1,50
A_44_P348812	Hist1h1t	4,23E-21	0,823849193	1,27	3,81	9,60
A_44_P349475	Cdh2	6,95E-10	0,627503551	1,12	1,02	-1,50
A_44_P349553	Col6a1	9,15E-10	0,623437642	1,13	1,02	1,48
A_44_P351211	Phlda1	3,30E-22	0,864566884	1,13	-1,89	-2,80
A_44_P351723	Cldn10	4,09E-12	0,69536705	-1,18	1,28	1,77
A_44_P352268	Plk2	1,52E-09	0,675084427	1,34	-1,19	-1,06
A_44_P352331	Bves	2,11E-09	0,631945262	1,17	1,54	-1,67
A_44_P353157	Tmem30b	5,68E-16	0,78398024	-1,33	2,54	3,37
A_44_P354078	Hist1h1a	2,77E-10	0,640737498	1,22	3,07	3,62
A_44_P354444	Bcs1l	5,25E-13	0,655934238	-1,09	-1,01	-1,56
A_44_P355073	Adssl1	2,49E-10	0,699165401	1,16	-1,15	1,40
A_44_P356027	Sc5d	1,33E-12	0,667889827	1,01	-1,11	-2,21
A_44_P356738	LOC102555114	8,63E-19	0,803414834	-1,07	-1,14	2,22
A_44_P356829		3,21E-12	0,698217543	2,01	2,89	1,90
A_44_P357089	Fbxl20	7,37E-14	0,721308545	-1,15	-1,17	1,66
A_44_P357870	Cpt1a	7,04E-24	0,901147006	-1,00	1,15	5,63
A_44_P358216	Pank4	2,33E-13	0,665839953	-1,02	-1,13	-1,38
A_44_P358227	Mapk15	4,26E-12	0,653216844	-1,33	1,15	2,17
A_44_P358974	LOC316124	1,32E-12	0,708465665	-2,11	1,78	1,83
A_44_P359052	Otub2	5,03E-14	0,70569222	-1,32	1,69	2,81
A_44_P359684	Nfkbiz	7,40E-13	0,651645672	1,29	-1,18	-2,29
A_44_P360501	Gabrb1	3,50E-17	0,774802537	1,08	-1,27	-1,28
A_44_P361416	Colca2	4,13E-12	0,601366083	1,01	2,15	3,59
A_44_P363291	Kcnk10	1,75E-11	0,657046793	1,13	1,25	1,21
A_44_P367465	Fzd9	8,11E-13	0,69480948	1,32	1,38	1,54
A_44_P368249	Spag8	2,66E-12	0,635205188	-1,14	-1,57	-1,49
A_44_P369612	Antxr2	3,06E-12	0,657453983	-1,27	-1,02	1,16
A_44_P369997	Trpm4	1,63E-14	0,717650006	1,26	1,19	2,01
A_44_P370052	Ldhc	2,28E-11	0,711247321	1,75	1,46	2,53
A_44_P374618	Cdkn1c	5,67E-13	0,717896613	1,18	1,29	2,74
A_44_P374708	Cry1	4,72E-11	0,620851193	1,38	-1,12	-1,94
A_44_P374824	Klk1c3	3,70E-13	0,703756198	-1,63	1,67	3,61
A_44_P375185	Slc39a14	1,60E-13	0,692860363	1,84	1,06	-1,23
A_44_P375658	Rbm11	4,83E-19	0,835327096	-1,01	4,01	6,32
A_44_P378742	Flrt3	2,16E-11	0,711917398	1,51	-1,14	-1,48
A_44_P378845	Rasgrp3	1,44E-17	0,743512368	1,21	1,20	-2,20
A_44_P379360	Fut7	1,12E-30	0,911062419	-1,12	2,93	11,69
A_44_P379891	Pspc1	7,26E-13	0,715175472	-1,06	1,13	1,14
A_44_P380182	Mthfd2	2,86E-13	0,686191653	1,05	-1,08	-1,46
A_44_P381917	Sphk1	2,65E-13	0,759329845	-1,09	1,38	2,95

A_44_P383899	Fut8	4,75E-11	0,643723832	-1,18	1,07	2,18
A_44_P383960	Kcna5	1,39E-14	0,698068606	-1,01	1,21	3,82
A_44_P384090	Des	9,65E-25	0,908487136	-1,38	-1,02	3,92
A_44_P384712	Rad54b	9,30E-17	0,726168335	-1,01	1,19	2,73
A_44_P388959	Lrp3	2,30E-16	0,775790499	-1,02	-1,24	-1,79
A_44_P389178	Cracr2b	2,12E-15	0,772731607	-1,17	1,09	2,37
A_44_P390385	Phf13	2,31E-17	0,822478042	-1,09	-3,10	-3,15
A_44_P390700	Fhl2	4,65E-15	0,709756844	-1,26	1,06	2,08
A_44_P393343	St3gal6	1,85E-11	0,656276789	1,23	1,03	-1,42
A_44_P393696	LOC308990	1,99E-11	0,632833059	-1,31	-1,37	-2,25
A_44_P393822	Plekho1	1,80E-25	0,897659543	1,01	-1,04	-2,96
A_44_P398230	Heca	3,44E-17	0,774952238	1,11	-1,56	-1,92
A_44_P398300	Fam160a2	4,35E-13	0,631636931	-1,09	-1,19	-1,81
A_44_P398376	Nudt18	4,80E-21	0,823042264	1,11	1,64	2,83
A_44_P398969	Gtpbp2	4,11E-12	0,601440671	1,12	-1,13	-1,39
A_44_P399414	Klk1	1,25E-12	0,689786914	-1,31	1,24	2,13
A_44_P400324	Maf	6,04E-10	0,62955023	1,69	1,16	-1,20
A_44_P401955	Zfp706	6,58E-13	0,697222782	1,10	-1,15	-1,63
A_44_P402980	Nkx6-2	3,79E-11	0,623935291	-1,02	1,19	1,16
A_44_P403475	Slc43a2	3,87E-17	0,755640575	-1,14	1,88	2,34
A_44_P403755	Dusp12	1,73E-12	0,685949379	1,11	1,28	1,53
A_44_P404064	Tiparp	1,24E-18	0,829292569	1,03	1,23	-1,36
A_44_P406805	Rfc3	5,84E-15	0,683345075	-1,24	1,09	1,57
A_44_P406825	Phc2	2,66E-11	0,603617103	-1,02	1,35	1,76
A_44_P409518	Gmpr	6,17E-12	0,670366599	-1,08	-1,18	-1,82
A_44_P409709	Phldb1	1,76E-12	0,640609137	-1,01	1,14	1,80
A_44_P409729	Opa1	3,99E-11	0,704555643	1,05	1,06	-1,52
A_44_P409803	Cd5	6,75E-16	0,766518264	-1,30	1,51	7,52
A_44_P409820	Faslg	3,85E-19	0,848772737	1,12	1,38	1,66
A_44_P409860		4,60E-16	0,752435924	1,29	1,95	2,87
A_44_P412121	Zfp335	5,81E-12	0,649199256	1,15	-1,14	-2,01
A_44_P414460	Rnd3	1,31E-12	0,668030342	1,33	-1,17	-1,95
A_44_P414527	Efna2	1,51E-15	0,775697554	-1,01	1,65	1,41
A_44_P414615	Daxx	2,26E-17	0,739431901	1,00	-1,16	-1,54
A_44_P416738	Fscn3	2,69E-10	0,619314796	2,40	2,01	1,99
A_44_P419676	Zc3hc1	9,17E-11	0,63467522	-1,04	-1,22	-1,29
A_44_P419746	Dennd1c	1,43E-11	0,612384246	-1,10	1,39	1,30
A_44_P419922	Haghl	2,08E-10	0,644775541	-1,20	1,59	1,10
A_44_P420640	Nup43	1,68E-19	0,841844413	-1,11	-1,05	-1,74
A_44_P421295	Cyp11b3	9,66E-12	0,642537631	-2,18	-1,65	-1,70
A_44_P421887	Ankrd33b	1,50E-17	0,781702517	1,02	1,66	2,01
A_44_P423490	Palmd	3,41E-23	0,851795304	-1,52	-1,06	-2,58
A_44_P424076	Dhdh	5,59E-17	0,787403314	1,20	3,38	5,58
A_44_P424723	Sik1	3,26E-18	0,822827036	1,01	-2,57	-1,35
A_44_P425619	Pgm3	1,22E-11	0,614566734	1,11	1,25	1,14
A_44_P426079	Kcnc1	7,24E-14	0,679625853	1,08	1,53	2,27
A_44_P428326	Hmgcs2	6,88E-10	0,605384196	-1,44	1,12	4,28
A_44_P428455	Cygb	3,06E-19	0,7946134	1,22	1,17	2,92
A_44_P428467	Homez	1,04E-11	0,61674681	-1,04	1,63	2,80
A_44_P429453	Lama5	4,17E-11	0,704018627	1,12	1,04	1,66
A_44_P430547	Ntrk1	1,24E-27	0,941252005	-1,21	1,54	5,68
A_44_P430581	Nsmce4a	8,21E-14	0,770501682	1,03	1,27	-1,21
A_44_P431049	Rrad	6,98E-29	0,907268074	-1,13	-1,83	-5,30
A_44_P432314	Fam98a	3,61E-16	0,771952367	1,02	-1,16	-2,81
A_44_P434271	Cntn2	1,54E-11	0,61135328	1,07	1,44	2,44
A_44_P435713	Amy1a	1,98E-15	0,756871488	-1,15	-1,05	9,99
A_44_P436040	Rassf6	9,92E-18	0,840442574	-1,92	1,48	1,60
A_44_P436399		3,07E-13	0,636118881	1,03	1,52	2,49
A_44_P437594	Gin1	2,94E-16	0,737201508	1,18	1,79	1,80
A_44_P437896	Bdnf	5,01E-18	0,772946166	1,22	-1,63	-3,17
A_44_P437945	Bbc3	2,60E-20	0,840550173	1,19	1,19	3,77
A_44_P437956	Stc1	2,16E-24	0,905588152	-1,04	-1,77	-2,21
A_44_P438492	Mak16	2,62E-13	0,638159475	-1,10	-1,12	-1,14
A_44_P440556	Muc13	2,47E-11	0,652512125	-1,33	1,32	3,54
A_44_P440558	Wfs1	1,32E-14	0,719859652	-1,14	1,03	2,08
A_44_P440944		4,56E-28	0,938035432	1,17	-1,02	2,13
A_44_P442780	Kcnab1	9,98E-15	0,789268909	1,28	1,33	-1,28
A_44_P445031	Fbxo32	6,88E-12	0,669008138	1,39	1,61	7,55
A_44_P445044	Apold1	1,21E-11	0,661820035	1,02	-1,20	-1,53
A_44_P446713	Pabpn1	3,42E-30	0,907511866	-1,18	-1,19	-2,83
A_44_P448051	Vpreb3	3,70E-10	0,711084256	1,75	2,04	2,85

A_44_P450307	Eps8	5,54E-16	0,784189896	1,34	1,19	2,44
A_44_P450486	Ttll1	2,33E-11	0,711035543	-1,21	1,10	-1,17
A_44_P450518	Rangrf	1,47E-18	0,828137049	-1,30	1,20	-1,59
A_44_P451120	Lmbrd1	3,95E-16	0,753828062	1,00	-1,08	-1,48
A_44_P455059	Azin2	3,85E-19	0,792910909	-1,30	1,02	3,35
A_44_P455101	RGD1561102	1,40E-12	0,643578823	1,09	3,22	2,21
A_44_P456172	Mterf2	9,01E-11	0,656228694	-1,40	-1,23	1,09
A_44_P456483	Eif2b5	1,06E-13	0,649476186	-1,04	-1,06	-1,39
A_44_P456599	Zdhhc23	3,20E-10	0,616777708	1,32	1,56	1,31
A_44_P458021	Tnfrsf11b	1,97E-10	0,645533227	1,28	1,25	1,46
A_44_P458901	Neurod2	7,85E-11	0,636836903	1,12	1,49	3,17
A_44_P459492	Lmo2	1,39E-13	0,672027519	1,26	1,07	1,82
A_44_P461383		7,45E-16	0,796498063	1,51	1,36	2,18
A_44_P463488	Amh	1,46E-19	0,829916594	1,10	1,74	2,11
A_44_P463831	Zfp423	1,20E-17	0,799339751	1,00	-1,10	2,20
A_44_P464330	RGD1304963	4,92E-17	0,753534035	1,18	1,38	1,76
A_44_P465880	Snap47	6,49E-16	0,749277157	-1,06	1,02	1,81
A_44_P465953	Rgs8	2,14E-22	0,841717232	-1,01	1,27	2,31
A_44_P466434	Fam212b	5,57E-13	0,699130704	1,06	1,15	-1,41
A_44_P467202	Pcdh8	8,84E-22	0,859486932	1,07	1,32	2,48
A_44_P467591	Neu2	3,74E-27	0,902803262	-1,31	1,26	4,15
A_44_P468239	Sgtb	5,03E-13	0,700290857	1,21	-1,08	-1,90
A_44_P470528	Ndufaf4	1,58E-14	0,754423021	1,20	1,01	-3,15
A_44_P470976	Rhobtb2	1,41E-14	0,697933184	1,89	-1,37	-1,58
A_44_P471818	Apcdd1	7,18E-17	0,728643495	1,04	1,40	2,47
A_44_P472661	Klf2	3,68E-18	0,821999782	2,56	2,02	1,06
A_44_P473234	LOC102554302	1,00E-12	0,692362996	-1,05	1,41	2,30
A_44_P473928	Spry1	4,06E-17	0,75523049	1,40	1,18	-1,67
A_44_P474101	Slc7a1	4,63E-21	0,862130684	-1,04	-1,33	-1,48
A_44_P475406	Hsf1	4,00E-16	0,771082503	1,01	-1,29	-1,98
A_44_P477406	Slc2a4	2,30E-15	0,755484697	-1,06	1,12	1,97
A_44_P477568	Sdccag8	3,57E-12	0,655471087	-1,03	1,02	1,32
A_44_P479942	Kcnk15	8,01E-21	0,819763466	1,06	1,38	2,96
A_44_P480573	Cytip	2,19E-22	0,866612871	1,37	1,28	-2,25
A_44_P480623		6,53E-24	0,901435277	-1,23	-1,69	-2,77
A_44_P480696	Ap1s2	1,12E-18	0,801536666	1,07	1,56	2,58
A_44_P480925	LOC691995	5,96E-13	0,677559542	-1,03	1,27	1,40
A_44_P481892	RGD1309079	2,36E-13	0,727329462	1,20	1,44	3,28
A_44_P481915	Id4	1,87E-13	0,762673314	1,32	1,46	1,24
A_44_P482519	Itgb7	2,57E-18	0,778326675	-1,19	1,46	3,49
A_44_P483728	Rps6ka1	1,48E-11	0,636844124	1,00	1,25	1,88
A_44_P484836	Tjp2	3,17E-23	0,864749593	1,24	-1,01	-1,98
A_44_P484984	Gnmt	1,86E-11	0,656262525	-1,15	1,31	1,96
A_44_P485388	Chd7	1,59E-14	0,736966676	-1,20	1,27	3,60
A_44_P485546	Trim43a	1,17E-12	0,618634015	1,53	2,14	2,50
A_44_P485948	Cux2	6,88E-12	0,707745127	1,28	-1,13	1,54
A_44_P487910	Nrros	2,13E-12	0,63812586	1,18	2,02	4,16
A_44_P489688	B3gnt8	1,51E-15	0,721286733	-1,18	-1,03	2,67
A_44_P491393	Nudt7	7,93E-10	0,603238402	1,07	1,37	2,72
A_44_P491759	Krt24	2,23E-18	0,77945973	-1,53	1,71	3,26
A_44_P493005	Foxq1	1,59E-12	0,724339605	1,23	-1,21	5,19
A_44_P493606	Camk2n1	2,50E-18	0,778549295	1,31	1,44	2,44
A_44_P493911	Slc13a5	8,66E-16	0,746599735	1,18	1,47	2,97
A_44_P493980	Nptxr	1,79E-17	0,810859116	-1,50	-1,21	1,96
A_44_P494044	Cdh15	6,81E-28	0,899394948	-1,48	1,20	14,90
A_44_P494310	Bri3	2,65E-14	0,666114044	1,02	-1,11	-1,34
A_44_P494439	Cnnm2	5,09E-14	0,683660798	1,06	-1,26	-1,15
A_44_P496447	Ntf3	3,16E-24	0,89557226	-1,32	1,31	2,32
A_44_P497174	Gramd1b	1,31E-09	0,618084755	1,33	1,03	-1,22
A_44_P497193	Senp8	4,73E-16	0,732648014	-1,12	1,51	2,20
A_44_P499377	Dmac1	1,48E-17	0,743270298	1,03	-1,17	-1,76
A_44_P500431	Lman1	4,50E-09	0,640632932	-1,01	1,10	-1,30
A_44_P501051		1,52E-21	0,856594367	-1,03	-1,06	-2,19
A_44_P501761	Crybg2	4,81E-19	0,835342262	-1,46	1,31	2,77
A_44_P501827	Cpsf6	1,59E-10	0,626948053	1,08	-1,08	-1,72
A_44_P503115	Sfrp2	1,11E-18	0,76556054	1,29	-1,07	-2,23
A_44_P503669	Zbed5	6,19E-23	0,848591535	1,03	1,44	1,85
A_44_P503699	Sergef	2,07E-20	0,841896053	-1,09	-1,04	1,58
A_44_P504750	Rassf9	3,33E-09	0,603712828	1,09	-1,49	1,28
A_44_P506299	Bcl2l15	4,94E-26	0,918455181	1,57	4,68	9,90
A_44_P506635	Ifrd2	4,35E-13	0,701927746	1,12	1,10	-1,27

A_44_P506980	Kcnk13	3,03E-20	0,810971277	1,00	1,38	6,48
A_44_P507571	Ccne1	5,43E-16	0,731334437	1,07	1,01	-1,57
A_44_P508055	Bcl2l11	5,58E-12	0,625313283	-1,13	1,07	3,88
A_44_P508434	Neu1	1,90E-12	0,612116762	-1,03	-1,33	-1,54
A_44_P508691	Tm6sf2	1,36E-28	0,92170148	1,16	1,57	2,42
A_44_P508809	Dpm1	5,38E-19	0,771459477	-1,07	-1,16	-2,03
A_44_P511359	Mum1	4,23E-18	0,774315476	-1,11	1,21	1,74
A_44_P512136	Dhcr7	3,10E-23	0,864846271	-1,06	1,07	1,63
A_44_P512728	Pxn	3,27E-21	0,808928387	1,13	1,06	2,51
A_44_P513385	Dab2	4,46E-08	0,62424575	1,42	-1,08	1,09
A_44_P514927	Fam69b	2,41E-15	0,755074798	1,09	-1,23	-1,90
A_44_P514987	Tshz1	3,77E-14	0,728278237	1,09	1,16	1,53
A_44_P517033	Rab11fip5	5,63E-12	0,649609423	1,03	-1,08	-1,47
A_44_P520700	LOC501266	7,15E-18	0,830451337	-1,07	1,02	2,13
A_44_P520929	C1qtnf1	6,41E-11	0,616536438	-2,00	1,31	4,86
A_44_P522047	LOC684871	3,96E-12	0,747354883	1,44	-1,15	-1,95
A_44_P522396	Etfdh	9,66E-13	0,648288101	-1,04	-1,14	-1,35
A_44_P522994		3,14E-11	0,626562489	1,10	1,32	-2,36
A_44_P523028		3,76E-27	0,919156763	1,27	1,24	4,38
A_44_P524630	Qrfp	2,83E-12	0,634407405	-1,67	1,91	3,61
A_44_P525059	Ficd	7,15E-16	0,728653125	1,02	1,29	2,90
A_44_P527270	Pth1r	6,13E-12	0,648504846	-1,13	-1,07	2,32
A_44_P529240	Rhbg	1,23E-18	0,784103033	-1,48	1,65	5,48
A_44_P529904	Bend7	1,17E-11	0,662298694	1,02	-1,33	2,11
A_44_P530131	Asb2	2,05E-30	0,933013633	1,03	3,25	13,70
A_44_P530547	Prom2	8,36E-20	0,803952819	-1,07	1,70	1,97
A_44_P532249	Fkbp5	2,06E-12	0,783155091	-1,16	-1,24	-1,33
A_44_P532476		4,28E-19	0,822925587	-1,04	-1,12	1,93
A_44_P533560	Fxn	2,60E-11	0,629168785	-1,12	-1,04	-1,36
A_44_P534089	Ccnb1	2,07E-11	0,654840673	1,08	-1,17	-1,26
A_44_P536613	Ch25h	4,35E-25	0,884366605	1,19	-1,26	1,28
A_44_P537652	Arid2	2,25E-15	0,772237761	-1,05	-1,09	-1,44
A_44_P538165	Tspan17	4,15E-11	0,62265911	1,01	-1,01	-1,23
A_44_P538521	Star	9,76E-15	0,741795502	-1,03	1,45	1,68
A_44_P538870	Calhm2	5,64E-14	0,682495116	-1,03	1,38	1,98
A_44_P538970	Ptpdc1	2,16E-13	0,709742604	1,40	1,42	2,10
A_44_P540065	Rbm10	1,05E-17	0,746328243	1,07	1,05	-1,49
A_44_P541020	B3galt4	4,39E-17	0,789320196	-1,09	1,67	2,58
A_44_P541692	Hexim2	1,97E-13	0,641727717	-1,21	1,28	3,06
A_44_P543017	Nhlrc1	3,19E-11	0,600980967	1,11	1,77	2,08
A_44_P543917	Fbxo42	2,47E-11	0,673238871	1,35	1,11	1,44
A_44_P545193	Hsd11b2	5,46E-12	0,671913136	1,17	1,38	2,40
A_44_P545887	Ccdc155	3,73E-19	0,809379286	-1,04	1,17	3,04
A_44_P546432		1,26E-12	0,668487403	1,88	2,54	3,97
A_44_P547771	Cited1	2,22E-22	0,854766183	1,05	1,24	1,77
A_44_P547788	Clk3	7,52E-24	0,859601099	1,01	-1,31	-2,03
A_44_P548241	Zfp51	7,81E-15	0,72522987	1,12	3,12	3,35
A_44_P548420	Pmvk	7,54E-17	0,76835875	-1,05	1,15	1,60
A_44_P548426	Ankrd35	1,36E-10	0,650645321	-1,62	-1,38	3,87
A_44_P548590	Ribc1	1,82E-11	0,608978315	1,07	1,33	2,80
A_44_P548726	Zfp414	6,88E-21	0,835318762	-1,28	-1,23	-2,09
A_44_P548730	Bcl9l	7,17E-12	0,646463772	1,06	-1,16	-1,82
A_44_P552533	Upb1	1,80E-23	0,855129944	-1,50	2,05	6,68
A_44_P554973	Cacna1g	1,39E-19	0,782028328	-1,38	1,06	3,84
A_44_P555253	Dnaja1	4,26E-21	0,838175629	-1,06	-1,08	-1,58
A_44_P556319	Fgf18	1,48E-16	0,762557242	-1,01	1,84	2,65
A_44_P556989	Ngfr	2,58E-16	0,830563218	-1,77	1,00	2,60
A_44_P557376	Drd1	5,82E-12	0,624751147	-1,01	-1,08	-2,01
A_44_P558163	Lipt1	1,92E-13	0,642086221	-1,18	1,77	1,20
A_44_P558411	Scrg1	8,70E-10	0,601824044	-1,07	1,09	1,12
A_44_P561324	Akap1	4,90E-13	0,656794643	1,37	-1,01	-2,19
A_44_P562505	Plxdc1	4,34E-22	0,837655098	-1,54	-1,17	5,91
A_44_P570046		1,39E-13	0,646098375	1,17	1,42	2,05
A_44_P576822	Inhbb	1,46E-26	0,906816725	1,21	2,39	2,07
A_44_P577108	Rasgef1c	3,08E-20	0,839534473	1,02	1,16	2,46
A_44_P579382		4,25E-12	0,746599062	-1,80	1,20	1,91
A_44_P586876	Tmem41a	1,07E-10	0,632485039	-1,16	-1,18	-2,04
A_44_P588645	Rrp1b	9,64E-16	0,745598983	-1,05	-1,06	-1,41
A_44_P592627	Samd10	1,12E-10	0,608508544	-1,10	-1,19	-1,27
A_44_P606049		5,41E-11	0,61893277	-1,57	3,75	4,00
A_44_P606216	Tmem269	7,51E-24	0,87167909	-1,04	2,49	5,44

A_44_P606502	Fam83f	6,08E-29	0,916363103	-1,69	4,99	12,56
A_44_P607542	Btbd11	1,80E-14	0,753158724	-1,11	1,14	1,65
A_44_P607583	Trmt61a	6,31E-12	0,648135695	1,20	1,20	-1,50
A_44_P608167	Tmem116	5,06E-23	0,87372165	1,24	1,85	5,29
A_44_P608318	Fam167a	5,47E-11	0,618773296	1,01	1,21	2,46
A_44_P608485	Robo3	1,29E-20	0,856642841	1,04	1,78	2,82
A_44_P611866	Tmem200c	6,48E-13	0,626468969	1,07	-1,13	6,76
A_44_P612186	Mylip	4,80E-24	0,873756372	1,17	-2,60	-4,10
A_44_P613170	Ndp	2,34E-15	0,771887242	-1,14	1,44	2,84
A_44_P620106	Tgfbi	5,69E-11	0,64127927	1,04	1,21	1,35
A_44_P620941	Tmem200a	4,65E-19	0,82236695	1,23	1,06	1,58
A_44_P622113	Sept8	3,42E-13	0,704643869	1,28	1,75	1,89
A_44_P623083	Slx1b	9,64E-13	0,671780522	-1,04	-1,08	-1,25
A_44_P623694		9,68E-16	0,763336593	-1,19	-1,03	-1,43
A_44_P625333	Ctdspl	6,90E-12	0,668977549	-1,10	-1,03	-1,14
A_44_P628515	Rgs7bp	4,46E-14	0,77610683	1,37	1,02	-1,55
A_44_P634770	Cxxc5	9,77E-22	0,846672796	-1,04	1,12	1,84
A_44_P635089	Etv5	3,83E-12	0,630365749	1,08	1,02	-1,67
A_44_P635769	Mks1	1,36E-12	0,667595728	1,23	1,48	2,70
A_44_P638290	Zfp467	1,10E-21	0,846028823	-1,01	1,25	3,80
A_44_P638511	Paqr5	2,82E-26	0,927060066	1,23	1,72	10,03
A_44_P639365	Col24a1	1,30E-10	0,723900863	1,11	1,38	-1,51
A_44_P649046	Lrrc20	1,11E-12	0,670031316	-1,05	-1,00	2,23
A_44_P653193	Dtx1	3,53E-19	0,793559808	-1,14	-1,04	2,33
A_44_P668185	Endod1	5,03E-11	0,66400832	-1,09	1,02	-1,37
A_44_P670825	Tmem182	5,06E-11	0,619887435	1,23	1,19	-1,83
A_44_P671340	LOC498601	2,56E-11	0,60415737	-1,16	1,14	2,58
A_44_P674018	Acer2	1,98E-12	0,611523976	1,28	2,34	5,15
A_44_P680749	RGD1565033	5,85E-14	0,682070297	1,11	1,60	2,58
A_44_P684579	Zbtb39	4,91E-10	0,61045977	1,07	1,94	1,60
A_44_P684795	Irs2	8,87E-14	0,719343757	1,01	-1,77	-1,10
A_44_P685298	Ice2	4,79E-09	0,619514777	-1,00	1,37	1,21
A_44_P686406	RGD1562310	4,17E-26	0,893851035	1,27	1,52	1,81
A_44_P694872	Fubp1	9,52E-11	0,610847536	-1,02	1,06	-2,73
A_44_P696669	Ehd1	1,71E-19	0,814757972	-1,24	1,06	1,98
A_44_P713311	Ncor1	4,29E-16	0,733586594	1,08	1,05	-1,49
A_44_P714007	Cidec	1,89E-19	0,798135703	-1,38	1,31	5,25
A_44_P714618	Arl4c	9,01E-13	0,649170276	1,31	-1,09	-2,12
A_44_P714839	Txlnb	1,57E-21	0,843998042	1,36	1,14	-1,91
A_44_P715517	Slc35f6	3,97E-12	0,60192525	1,04	1,14	1,28
A_44_P716637	Msx2	2,41E-19	0,826702301	1,12	1,17	1,07
A_44_P720576	Lrrn4cl	6,79E-10	0,605587991	-1,35	1,03	2,20
A_44_P727824	Ppp2r5a	8,63E-14	0,737747517	1,09	1,03	2,06
A_44_P728793	Tppp2	9,30E-12	0,618358144	-1,91	3,96	5,24
A_44_P729856	Ppp1r3d	1,69E-14	0,811774028	1,17	1,53	-1,04
A_44_P732517	Zadh2	8,01E-19	0,787400204	1,17	1,87	3,05
A_44_P744809	RGD1563056	4,29E-16	0,711066465	-1,24	1,49	3,73
A_44_P745585	Tprn	2,02E-19	0,797634295	-1,02	1,14	2,71
A_44_P745710	Olfml2a	1,20E-12	0,669102548	1,40	1,14	1,18
A_44_P760697	Nhej1	1,25E-10	0,630306565	-1,72	-1,38	2,89
A_44_P764174	Fcmr	4,45E-23	0,893798951	1,11	3,65	8,64
A_44_P768777	Klf14	2,37E-19	0,812520175	1,71	1,73	3,79
A_44_P770253	Rfx8	5,65E-10	0,608359276	-1,23	-1,29	4,46
A_44_P775631	RGD1564854	1,80E-11	0,609131652	-1,52	4,35	4,56
A_44_P776231	Tp53i11	4,21E-24	0,874362353	-1,60	-1,02	5,01
A_44_P776390	Kcnk5	4,49E-18	0,773829233	-1,02	1,11	2,04
A_44_P777424	LOC100362783	2,89E-13	0,75845308	1,06	-1,02	1,36
A_44_P779296	LOC502684	5,97E-26	0,881927766	1,23	1,54	4,32
A_44_P781945	Tbx4	6,74E-11	0,638953169	-1,46	-1,01	3,53
A_44_P787936	Stat3	2,52E-15	0,771216365	1,06	-1,29	-1,37
A_44_P790674	Opn3	3,09E-23	0,852305341	1,23	1,05	-1,91
A_44_P791030		3,29E-18	0,756459833	1,04	1,23	3,83
A_44_P791908	Asprv1	6,54E-14	0,655343836	-1,84	3,09	2,45
A_44_P792628	Spef1	1,31E-09	0,618064807	-1,06	1,16	1,73
A_44_P793551	Lin28a	2,34E-18	0,779071027	-1,22	1,87	5,32
A_44_P794669	Afdn	2,34E-14	0,733131714	-1,11	-1,30	-2,65
A_44_P807585	Prcd	1,45E-30	0,933870119	-1,01	1,52	3,64
A_44_P808544	Gpr61	2,73E-16	0,737875609	1,18	-1,33	-1,75
A_44_P808710	Rd3	4,22E-12	0,675090931	1,55	1,95	3,06
A_44_P809374	Clcf1	6,00E-14	0,723462738	-1,21	-1,06	3,46
A_44_P809486		7,19E-22	0,8605643	-1,42	1,21	4,08

A_44_P810179	Acd	7,04E-29	0,930486502	1,00	-1,08	-2,38
A_44_P810190	Srrt	4,87E-13	0,679948926	1,13	-1,12	-2,11
A_44_P817760	Tspan15	1,29E-22	0,857608887	-1,05	-1,05	3,95
A_44_P819401	Slc40a1	8,16E-13	0,731564894	1,66	1,46	1,34
A_44_P821368	RGD1560958	1,45E-20	0,844004008	1,05	2,71	4,74
A_44_P822447	Ctla2a	3,25E-15	0,784336771	-1,88	1,21	2,09
A_44_P822557	S100a14	3,55E-14	0,709453673	1,34	1,54	2,37
A_44_P823666	Zhx2	1,37E-13	0,694630016	1,25	-1,09	1,74
A_44_P823711	Tm2d1	1,29E-18	0,764342613	1,04	1,41	6,91
A_44_P823749	Scn5a	6,78E-10	0,648517067	-1,11	-1,10	1,89
A_44_P832655	Dedd2	2,23E-09	0,63113308	-1,14	-1,46	1,39
A_44_P837888	Pxylp1	1,76E-12	0,613128267	1,11	1,25	1,81
A_44_P838583	Psrc1	5,16E-21	0,837036103	1,10	1,26	3,93
A_44_P839176	Usp25	1,09E-16	0,782032062	1,15	1,05	-3,37
A_44_P839270	Pax1	2,08E-21	0,828257042	-1,10	1,45	1,60
A_44_P839975	Tmem132e	5,67E-12	0,649522741	-1,09	1,02	3,21
A_44_P847319	Luzp2	4,17E-14	0,776710041	1,48	1,34	-1,19
A_44_P852017	Gpr146	1,13E-12	0,669857625	-1,02	1,30	1,65
A_44_P852545	Tmem196	9,58E-22	0,870172597	1,13	1,23	-4,13
A_44_P853916	Slc43a2	2,48E-26	0,90494715	1,14	2,54	6,46
A_44_P868418	Igsf3	3,92E-11	0,623466282	1,03	1,10	1,61
A_44_P868890	Gli2	1,86E-17	0,810594262	1,38	1,12	4,16
A_44_P874636	Cpsf6	3,28E-16	0,713781901	1,06	-1,11	-2,55
A_44_P876363	Vav2	9,13E-24	0,935240588	-1,41	-1,18	2,45
A_44_P878711		2,90E-09	0,605912324	-1,21	1,35	3,68
A_44_P879764	B4galt1	1,47E-10	0,604474192	1,06	1,29	1,55
A_44_P883462		6,32E-24	0,872485356	1,03	1,41	1,61
A_44_P884080	Smim11	9,83E-12	0,617586413	-1,08	-1,13	-1,49
A_44_P885003	Arap2	1,75E-11	0,609504005	1,20	-1,05	-1,51
A_44_P885889	Fam81a	6,92E-15	0,745126607	-1,25	1,18	1,70
A_44_P886690	Lpar2	7,73E-11	0,613850435	1,61	2,27	2,05
A_44_P897247	Rab11a	2,56E-10	0,620032415	-1,13	1,05	-1,24
A_44_P898436	Lrrc17	2,68E-16	0,790137703	1,31	1,60	1,41
A_44_P899127	LOC500300	1,72E-31	0,944662039	1,16	1,17	-1,60
A_44_P899293	Snai1	7,45E-20	0,834139402	1,27	1,27	-1,01
A_44_P899938	Fam124a	1,53E-12	0,706827724	1,13	1,52	1,63
A_44_P901088	Ccnb1ip1	9,92E-13	0,671428741	-1,23	2,12	1,74
A_44_P912777	RGD1310553	2,09E-11	0,654680347	1,09	-1,05	-1,70
A_44_P913774	Cct8l1	5,67E-13	0,654966534	-1,33	1,75	5,69
A_44_P914607	Plxnd1	4,06E-12	0,629608933	1,15	1,27	1,53
A_44_P917327	Lrtm2	8,31E-32	0,946166942	1,14	3,11	6,77
A_44_P917564	Mat2b	1,32E-15	0,77687816	1,10	-1,02	-1,63
A_44_P929423	Itga9	2,84E-16	0,774004621	-1,10	-1,04	1,85
A_44_P929530		5,58E-33	0,951401963	1,48	1,53	3,06
A_44_P930152	Mpped1	1,28E-22	0,898155721	-1,99	1,14	2,46
A_44_P930534	Gas2	1,72E-16	0,778206333	-1,10	1,21	3,71
A_44_P932103		1,07E-16	0,746543429	1,20	1,29	-2,52
A_44_P944353	Armc2	5,47E-13	0,699333674	1,07	1,20	1,44
A_44_P945336	Phf20l1	2,33E-11	0,605511324	-1,03	1,02	1,47
A_44_P945404	Zfp703	4,19E-16	0,753285216	-1,03	-1,03	2,87
A_44_P945723	Zc3h12d	4,79E-15	0,781007713	-2,74	3,69	2,50
A_44_P945759	Cep41	6,59E-18	0,803805367	1,27	1,60	3,09
A_44_P946504	Foxj2	1,68E-11	0,610092191	1,16	-1,13	-2,10
A_44_P951676	LOC497899	3,80E-21	0,863164468	-1,20	1,34	2,49
A_44_P956272	Ogt	2,08E-14	0,715122327	-1,06	1,03	-1,59
A_44_P959683	Tmem74	3,64E-13	0,660439561	1,20	1,48	1,55
A_44_P960146	Gga3	1,39E-14	0,69811416	1,17	-1,05	-1,88
A_44_P961602	Rasl12	1,29E-18	0,800506993	1,34	1,39	5,04
A_44_P961744	Cdh11	3,42E-12	0,631878887	-1,12	-1,04	-3,13
A_44_P962473	Nhsl2	8,92E-17	0,726568742	-1,28	1,49	2,52
A_44_P962701	Nol4l	1,50E-20	0,855786916	1,20	1,07	4,37
A_44_P963812	Ctnnal1	3,33E-10	0,616225652	1,07	-1,26	-2,11
A_44_P964460	Itk	2,89E-12	0,634113597	-1,63	-1,41	6,62
A_44_P974651	Tmem74b	1,26E-24	0,86830854	1,04	-1,16	-3,69
A_44_P975327	B3gnt2	1,98E-11	0,655449337	1,72	1,33	1,47
A_44_P975731	Zfp292	1,14E-12	0,669730557	1,04	-1,23	-1,40
A_44_P977318	Tfcp2l1	4,93E-19	0,772148669	-1,14	1,64	3,17
A_44_P988767	Crem	6,12E-12	0,648535038	-1,06	-2,15	-3,67
A_44_P989558	Slfnl1	3,36E-12	0,677914856	-1,10	-1,06	-1,76
A_44_P991472	Rnf39	3,05E-22	0,875724014	1,14	1,46	2,35
A_44_P993359		1,52E-22	0,843628069	1,08	2,03	2,76

A_44_P994550	Rars2	1,27E-11	0,638919698	-1,00	1,24	1,92
A_44_P997843	Csrp3	5,34E-20	0,848630737	1,16	-1,04	-2,14
A_44_P998638	Pprc1	1,65E-17	0,780920463	1,13	-1,17	-3,79
A_44_P999125	Masp2	1,44E-18	0,852322783	-1,24	4,52	2,79
A_44_P999395	Lmbr1l	2,06E-09	0,688606121	1,03	-1,83	-1,53
A_64_P000041	Cfap58	6,07E-25	0,910121777	-1,12	-1,33	2,22
A_64_P000095		3,76E-19	0,793090678	-1,09	1,50	5,18
A_64_P000171	Dhcr24	6,60E-17	0,750920825	-1,12	1,03	1,40
A_64_P000281	Nkain4	1,30E-11	0,613671846	1,09	1,20	2,27
A_64_P000386	Isl2	2,32E-09	0,609365796	-1,55	-1,32	2,39
A_64_P000645	Ttc30a1	4,69E-18	0,773481369	-1,19	1,80	3,83
A_64_P000708	Rnf125	2,15E-13	0,689470927	1,32	1,33	1,19
A_64_P000876	Kcna7	3,91E-16	0,753905225	-1,12	1,52	2,34
A_64_P000895	LOC108348336	8,74E-15	0,742869556	-1,00	1,36	1,61
A_64_P001096		4,12E-22	0,851443556	1,01	1,00	2,40
A_64_P001219	Mthfs	7,29E-10	0,647495278	-1,24	-1,01	1,42
A_64_P001249	Chrnb2	2,26E-13	0,640008668	1,01	1,14	1,35
A_64_P001344		2,46E-13	0,687933459	1,04	-1,07	1,58
A_64_P001364	Prickle1	4,79E-17	0,753771174	1,26	-1,20	-3,51
A_64_P001426	Tmc8	3,42E-19	0,793787392	-1,20	1,57	3,17
A_64_P001461	Dnmt3l	3,55E-19	0,809720541	-1,54	1,33	4,75
A_64_P001799	Fam110d	5,67E-11	0,641323103	-1,37	1,96	1,83
A_64_P002097		6,66E-21	0,820952522	-1,04	1,04	5,21
A_64_P002110	LOC363337	1,24E-20	0,867749973	1,30	1,17	1,43
A_64_P002339	Zbed4	8,75E-11	0,676383332	1,04	-1,07	-2,81
A_64_P002479		3,60E-22	0,852176704	-1,21	-1,06	2,35
A_64_P002809	Grhl1	4,70E-22	0,850720237	1,21	3,42	4,66
A_64_P002899		2,34E-13	0,744546856	-1,36	-1,30	-1,70
A_64_P002949	Disp1	8,83E-20	0,819176825	-1,09	-1,13	1,30
A_64_P003261	Tbc1d5	1,71E-14	0,695864169	1,07	1,45	2,59
A_64_P003306	Eif2d	2,86E-16	0,715152933	-1,01	-1,00	-1,59
A_64_P003327	Lix1l	6,62E-17	0,750897022	1,08	1,06	-1,13
A_64_P003378	Tuba3b	5,26E-12	0,728128832	-1,33	1,14	1,19
A_64_P003604	Mylk2	2,35E-12	0,609190756	1,07	1,57	1,89
A_64_P003694	Abcc12	1,21E-14	0,699647684	-1,29	1,59	3,95
A_64_P003749	Pax4	3,71E-13	0,722491728	-1,01	1,25	1,86
A_64_P003876	Fxyd3	3,01E-27	0,893904139	-1,51	1,42	4,91
A_64_P003948	Slc24a4	2,05E-13	0,710332119	1,80	-1,07	1,79
A_64_P003972	Slc22a4	1,69E-10	0,602480725	-1,39	-1,02	1,66
A_64_P004007	Rd3l	1,11E-28	0,922281475	1,14	-1,15	-4,51
A_64_P004279		4,46E-13	0,631313003	1,13	-1,09	-1,28
A_64_P004307	Pipox	1,29E-12	0,689378333	-1,07	2,36	3,95
A_64_P004337	Dtl	1,11E-11	0,640713473	1,19	1,75	1,93
A_64_P004344	Hist2h2aa2	1,05E-15	0,744784296	1,30	1,60	1,88
A_64_P004406		1,38E-14	0,755725398	1,67	1,74	-1,15
A_64_P004539	Rnase12	5,75E-29	0,907907262	-1,21	5,65	16,58
A_64_P004584	Cacna1g	2,31E-30	0,925774713	1,13	1,39	3,35
A_64_P004702	Akap3	1,41E-26	0,88786419	-1,54	3,19	16,96
A_64_P005053	Vsig8	4,00E-17	0,790059461	1,17	3,45	2,91
A_64_P005223	Ppp1r1b	2,20E-11	0,606294894	-2,04	-1,13	3,73
A_64_P005228	Ppp1r1b	1,57E-22	0,868272513	1,03	-1,06	2,91
A_64_P005320	Bfar	2,35E-19	0,79656079	-1,18	-1,86	-3,55
A_64_P005391	Drgx	5,80E-23	0,861719617	1,51	2,92	4,34
A_64_P005847		9,49E-14	0,676494993	1,15	1,45	1,82
A_64_P006072	Zfp536	4,17E-13	0,702402995	1,17	1,09	-1,15
A_64_P006097	Gjc2	3,96E-14	0,661343144	1,47	1,58	2,91
A_64_P006183	Gjd3	1,91E-30	0,926279577	1,62	2,07	2,28
A_64_P006285	Lhx1	6,78E-19	0,805151492	-1,00	1,48	3,97
A_64_P006473		1,55E-23	0,889047561	-1,04	1,18	2,58
A_64_P006475		4,19E-15	0,710843347	-1,58	1,05	2,08
A_64_P006478	Slc25a34	1,30E-20	0,831444365	1,10	1,07	4,27
A_64_P006525	LOC681364	3,25E-29	0,932498521	1,10	1,24	3,77
A_64_P006538		4,19E-12	0,601184737	-1,05	-1,17	-1,79
A_64_P007133		8,70E-21	0,819229328	1,14	1,45	3,61
A_64_P007135	LOC108349417	9,19E-19	0,786352549	1,57	2,43	3,12
A_64_P007148	Nav1	2,65E-12	0,60753784	1,13	1,26	1,61
A_64_P007163	Mybphl	7,29E-17	0,750024395	-1,28	-1,13	3,88
A_64_P007178		4,26E-17	0,789570019	-3,35	1,99	3,42
A_64_P007518	Ttc39a	5,01E-11	0,620016608	1,11	1,05	1,49
A_64_P007633	C4bpb	6,08E-14	0,723327176	1,54	1,26	2,07
A_64_P007783	LOC103691261	6,80E-12	0,669146417	1,27	1,57	2,58

A_64_P007793	Prob1	7,57E-16	0,765518502	1,82	3,09	5,47
A_64_P007822	Fezf1	4,80E-15	0,709430202	1,09	1,57	1,93
A_64_P007833	LOC685125	2,51E-18	0,795578454	1,52	1,20	3,17
A_64_P007904		5,06E-13	0,656380337	-1,84	2,39	3,61
A_64_P008042	Ankrd9	1,20E-13	0,696139182	1,20	-1,25	-3,34
A_64_P008060	LOC108348293	1,60E-35	0,956720473	1,16	1,26	3,91
A_64_P008161	Lingo3	1,31E-17	0,782780118	-1,10	2,14	3,57
A_64_P008177	Tchh	1,05E-12	0,691838949	2,00	2,40	2,55
A_64_P008747	Stambpl1	1,21E-13	0,734258895	1,17	-1,03	-1,82
A_64_P008777	Ccdc28a	1,10E-19	0,831703174	1,09	1,13	3,45
A_64_P008879	Thpo	4,70E-14	0,706444159	1,19	1,10	1,83
A_64_P009137	Olr337	5,64E-13	0,655050946	1,09	2,42	2,22
A_64_P009355	Reep4	9,78E-15	0,758926133	-1,01	1,37	1,81
A_64_P009565	Vmo1	2,01E-24	0,913639105	1,06	1,91	5,31
A_64_P009618		1,80E-14	0,670622459	-1,17	1,31	1,83
A_64_P009718	Btbd17	6,71E-36	0,969565118	-1,10	2,81	17,36
A_64_P009738	RGD1559714	1,42E-18	0,81477548	-1,33	1,08	8,27
A_64_P009803	Btg2	1,49E-24	0,945382706	2,56	1,11	3,42
A_64_P009857	Tbx1	1,30E-17	0,813192721	1,64	1,65	2,06
A_64_P009892	Lbx1	1,31E-13	0,733490937	-1,22	-1,38	-2,64
A_64_P009897	Ppp2r1b	2,43E-22	0,895521942	2,03	5,22	5,10
A_64_P009996	Tac1	2,07E-15	0,772945611	1,45	1,30	1,18
A_64_P010037	RGD1309139	6,96E-11	0,615343878	1,10	1,76	2,42
A_64_P010139		2,75E-10	0,640858268	5,12	-1,09	1,75
A_64_P010234	Gys1	3,35E-18	0,79338216	1,02	-1,13	-2,11
A_64_P010269	LOC108351885	2,93E-12	0,633915252	1,06	1,17	1,32
A_64_P010298	Sh2d3c	9,29E-19	0,854869571	1,05	-1,31	3,99
A_64_P010393	Clcn3	1,91E-14	0,694623001	1,20	1,32	1,41
A_64_P010960		4,51E-15	0,781524476	1,16	1,21	1,66
A_64_P011045	Hrh3	2,89E-13	0,70652162	-1,07	1,24	1,65
A_64_P011070	Asic4	5,95E-24	0,883415057	-1,01	1,20	3,07
A_64_P011099	Rpl3l	5,86E-14	0,682055841	1,08	1,29	1,52
A_64_P011135	Rnf151	8,31E-16	0,727182973	-1,04	1,47	1,65
A_64_P011489	Gdf15	1,08E-09	0,621019743	1,63	1,27	1,90
A_64_P011654	Zfp296	2,33E-18	0,825098792	1,18	1,89	2,49
A_64_P011779	Sult1a1	9,01E-18	0,768110257	1,06	1,22	3,59
A_64_P011959	Slc2a2	1,13E-23	0,88058169	1,51	1,84	13,10
A_64_P011979		6,38E-12	0,669956291	1,16	1,16	1,83
A_64_P012014		6,37E-16	0,707041814	2,34	15,60	7,59
A_64_P012165		6,27E-24	0,872520445	-1,03	1,17	3,58
A_64_P012166	LOC108349295	1,67E-22	0,86795642	1,29	1,05	2,49
A_64_P012198	Agrp	3,52E-13	0,72306365	-1,03	1,21	1,88
A_64_P012325	Slc35d1	3,60E-20	0,838602477	1,46	-1,32	-3,25
A_64_P012328	Slc35d1	2,31E-14	0,692516613	1,27	-1,03	-4,76
A_64_P012467	LOC679711	5,88E-18	0,804635734	-1,10	-1,09	3,42
A_64_P012471	LOC363324	1,30E-27	0,922357832	1,98	1,45	4,55
A_64_P012477	LOC363337	1,03E-20	0,845957181	-1,07	1,10	2,57
A_64_P012846	LOC108349386	4,57E-28	0,931987964	1,50	1,62	4,35
A_64_P012868	Tnxa-ps1	7,30E-11	0,659063682	1,29	-1,00	1,93
A_64_P012947	Dapk1	3,59E-10	0,615109735	-1,23	-1,08	1,22
A_64_P012967	Nif3l1	1,19E-13	0,673879586	1,13	2,48	2,32
A_64_P012972	Tecta	1,13E-12	0,669820811	1,27	1,27	1,94
A_64_P013288	Stmn4	1,19E-14	0,720942701	-1,12	1,01	2,47
A_64_P013398	Gne	4,15E-13	0,658829194	1,22	-1,04	-2,37
A_64_P013402	Rab33a	3,20E-17	0,820192329	-1,10	-1,11	-1,90
A_64_P013753	LOC108348296	8,89E-29	0,929868614	1,41	1,55	4,40
A_64_P014096	Htr3a	2,97E-16	0,737095236	-1,07	1,59	4,43
A_64_P014159	Plpp5	4,01E-10	0,613463501	-1,20	1,12	1,31
A_64_P014178		4,09E-11	0,686125166	-1,16	1,77	1,08
A_64_P014356	Map2k6	8,11E-17	0,826410798	-1,49	-1,03	2,93
A_64_P014504		7,27E-22	0,834622408	-1,03	1,07	6,67
A_64_P015104	Loxhd1	2,32E-15	0,693445876	-1,43	1,80	4,88
A_64_P015366	Mfsd4a	9,16E-18	0,785619778	1,15	1,07	-1,56
A_64_P015370	Mfsd3	7,44E-12	0,621406509	-1,12	1,33	1,94
A_64_P015395	Spesp1	1,87E-10	0,600958978	-1,42	1,59	-1,51
A_64_P015460	Ccl4	9,75E-12	0,703632987	1,28	-1,38	1,04
A_64_P015479	Rfc5	6,77E-16	0,748895626	-1,09	1,11	1,40
A_64_P015549	Lyl1	2,39E-13	0,72718632	1,32	1,51	1,31
A_64_P015911	Srsf7	1,21E-12	0,618188506	1,21	-1,58	-2,30
A_64_P016746	Lhx4	7,10E-15	0,726194473	1,54	1,85	3,45
A_64_P016751	Tmem243	4,59E-11	0,621242793	-1,04	-1,02	-1,69

A_64_P016766		5,58E-14	0,758674546	-1,25	2,46	5,82
A_64_P016828	Myo1b	3,33E-12	0,67801528	-1,02	-1,10	-1,37
A_64_P016951	LOC108348348	1,80E-24	0,897774414	-1,11	1,39	4,02
A_64_P017243	Tbc1d8	7,64E-18	0,802714699	1,25	1,00	-2,10
A_64_P017283	Speg	6,39E-14	0,655628761	1,07	1,50	3,71
A_64_P017363	Slc30a3	3,23E-19	0,810372711	1,11	-1,02	1,43
A_64_P017495		6,27E-11	0,639933386	-1,36	-1,70	1,98
A_64_P017631	Med9	1,39E-13	0,64614313	-1,07	1,42	2,03
A_64_P017705		7,98E-15	0,679862551	-1,25	1,38	3,05
A_64_P017706	LOC102547811	4,34E-31	0,942688137	1,17	1,27	3,80
A_64_P017758	Morn2	4,68E-17	0,788810201	-1,01	1,19	1,68
A_64_P018053	LOC108348337	6,17E-28	0,908984812	-1,05	1,16	4,67
A_64_P018427		3,48E-11	0,668823578	1,68	1,05	-1,35
A_64_P018633		1,75E-16	0,807780768	-1,76	1,08	1,48
A_64_P018761	Calca	4,61E-10	0,611412326	-1,06	1,10	2,11
A_64_P018766	Calca	8,02E-25	0,909142511	1,28	3,99	3,95
A_64_P018891		9,02E-23	0,831539895	1,36	1,75	8,16
A_64_P018901	Rprml	4,33E-12	0,628720729	1,20	1,24	1,36
A_64_P018907	Tnfrsf4	5,54E-18	0,819180894	1,37	2,51	1,69
A_64_P019016	LOC501396	3,20E-11	0,707231675	-1,29	3,19	1,21
A_64_P019081	LOC690490	6,48E-14	0,722665265	1,87	2,44	2,06
A_64_P019130	Ccpg1	2,03E-17	0,795334103	-1,00	-1,20	-1,46
A_64_P019135	Ccpg1	1,54E-17	0,781479037	-1,06	-1,12	-1,38
A_64_P019175		2,07E-24	0,905733367	-1,01	1,23	2,72
A_64_P019280	Pik3ip1	1,24E-30	0,927424846	1,11	1,78	3,31
A_64_P019401	Sdf2l1	1,02E-22	0,890320788	-1,17	1,03	1,12
A_64_P019608	Prkca	1,67E-11	0,697220967	1,48	1,05	-1,35
A_64_P019673	Arhgap20	3,50E-23	0,885557752	1,49	1,12	-2,81
A_64_P019756		1,25E-13	0,715716683	-1,42	-1,40	9,31
A_64_P019836	Ceacam18	2,87E-19	0,795093117	-1,03	1,69	1,58
A_64_P020154	Plxnb2	7,67E-13	0,695463468	-1,04	1,15	1,48
A_64_P020238		7,49E-10	0,647100765	1,59	4,35	1,72
A_64_P020248	Hist1h2bo	3,39E-16	0,755210738	-1,10	1,81	2,10
A_64_P020269	Hist1h2bh	1,26E-21	0,845228885	-1,06	2,06	4,15
A_64_P020388		1,36E-16	0,76325343	1,59	8,82	4,42
A_64_P020431	LOC687560	2,31E-17	0,794355146	1,58	1,80	3,71
A_64_P020471	Lrrc3b	9,67E-20	0,832517867	1,23	1,09	-1,90
A_64_P020506	Crtam	1,63E-24	0,867084522	-1,49	1,34	12,51
A_64_P020621	Dlk2	4,29E-10	0,612470419	1,39	1,21	-1,46
A_64_P020741	Rsph1	1,30E-11	0,660937087	1,24	1,16	1,62
A_64_P021043	Pcsk9	8,06E-21	0,819727107	-1,17	1,78	5,58
A_64_P021149	LOC108349347	1,13E-14	0,700368544	1,53	1,62	2,47
A_64_P021206		1,97E-19	0,813793561	-1,02	1,21	6,32
A_64_P021221		1,72E-29	0,940090103	1,47	1,48	4,95
A_64_P021241	Nradd	2,43E-10	0,620824386	-1,06	1,08	1,20
A_64_P021320	Pqlc1	8,60E-14	0,677637477	1,09	-1,37	-1,63
A_64_P021428	Slc16a6	5,85E-14	0,723729836	-1,36	2,06	2,01
A_64_P021606	Mycbpap	3,45E-23	0,837119526	1,18	1,64	4,22
A_64_P021631	Nxnl2	8,64E-12	0,666122601	1,28	1,32	-1,91
A_64_P021686	Epor	2,41E-11	0,673511922	1,21	1,18	1,81
A_64_P021702		8,04E-11	0,657763379	1,06	-1,09	-1,80
A_64_P021845	Car7	2,47E-29	0,92651594	1,12	-1,38	-11,09
A_64_P022015	Sstr2	2,56E-24	0,887029972	-1,27	1,60	5,62
A_64_P022155	Slc2a5	2,89E-13	0,663262752	-1,01	1,31	1,98
A_64_P022907	LOC108349337	1,35E-28	0,935118543	1,29	1,12	3,65
A_64_P022913	Nat8f1	9,45E-18	0,767709924	1,23	4,00	4,14
A_64_P022943	Rara	3,09E-25	0,904406059	1,59	2,58	3,50
A_64_P023098	Otor	1,31E-19	0,782511379	1,16	3,79	7,26
A_64_P023102	Otor	1,76E-19	0,780220062	1,38	1,69	3,08
A_64_P023179	Olr1388	1,16E-11	0,701619923	1,41	2,47	5,01
A_64_P023203	Wdr73	8,74E-12	0,619204829	1,05	1,02	-1,35
A_64_P023250	Ascl2	8,51E-19	0,803518275	1,11	1,13	2,40
A_64_P023381	Col6a5	1,57E-10	0,64862935	1,05	1,31	3,28
A_64_P023561	Cox10	9,03E-13	0,69357435	1,08	1,12	-1,15
A_64_P023823	Wnt7a	9,72E-28	0,898103938	1,04	1,12	5,59
A_64_P024112		3,47E-11	0,647969625	-1,09	-1,52	-2,41
A_64_P024272		1,01E-10	0,609959469	1,49	1,38	-2,15
A_64_P024356	Sh2b2	2,65E-13	0,687097801	-1,26	-1,20	-2,12
A_64_P024474	Stum	2,84E-18	0,83647863	-1,09	1,18	2,59
A_64_P024570	Il12a	3,76E-14	0,661979205	-1,12	1,20	3,34
A_64_P024813		2,35E-13	0,665742395	-1,11	1,54	2,96

A_64_P024826	Crygd	9,47E-16	0,702932369	-1,10	2,26	2,64
A_64_P024841	Cryga	1,08E-26	0,888923517	-1,08	1,75	2,84
A_64_P025050	Ccl1	3,87E-16	0,850541594	-3,43	2,62	11,89
A_64_P025098	Cebpg	2,05E-16	0,819834489	1,15	1,15	1,44
A_64_P025118	Cebpb	3,07E-14	0,819479878	1,49	-1,11	1,00
A_64_P025169	Phlda2	1,78E-18	0,826897445	-1,17	1,75	3,38
A_64_P025173	Phlda2	4,64E-17	0,788881894	2,33	2,80	2,60
A_64_P025248		1,83E-10	0,601296828	-1,70	1,21	-2,23
A_64_P025325	LOC501396	7,42E-18	0,80292841	1,00	1,27	1,46
A_64_P025496	Maff	1,67E-10	0,686563268	1,01	-1,47	2,01
A_64_P025535		1,43E-33	0,958335831	-1,00	1,25	4,36
A_64_P025599	Eif4g2	2,35E-09	0,60912454	-1,03	-1,03	-1,28
A_64_P025618	Slc25a35	1,17E-12	0,709857273	1,04	1,88	1,25
A_64_P025664	Cfap57	2,46E-19	0,826546404	-1,07	1,16	5,22
A_64_P025678	Tbc1d16	1,53E-18	0,851939912	1,14	1,13	1,96
A_64_P025963	Ccnf	9,99E-11	0,674649903	1,11	-1,20	-1,52
A_64_P026297	Insl6	2,61E-24	0,904885744	-1,32	2,30	6,74
A_64_P026374	Flt3	3,85E-13	0,65976289	-1,13	-1,07	1,98
A_64_P026442	Tesmin	6,41E-26	0,881623854	1,00	1,81	6,17
A_64_P026883	Ick	1,43E-11	0,637285975	1,11	-1,12	-1,78
A_64_P027263	RGD1584023	4,96E-11	0,701900274	1,19	5,94	1,61
A_64_P027530		2,89E-14	0,711662726	-1,03	-1,02	-1,86
A_64_P027625	Rxrg	3,00E-12	0,605835249	1,10	1,29	2,49
A_64_P027640	Tp53bp1	2,49E-13	0,638812886	1,14	-1,03	-1,27
A_64_P027707	Erich4	2,65E-15	0,735946252	-1,53	1,00	7,54
A_64_P027847	Fer	6,54E-11	0,732024483	1,20	-1,12	-1,18
A_64_P027911		4,24E-18	0,821032908	-1,26	1,28	4,19
A_64_P028290	Yrdc	4,30E-27	0,910958362	-1,30	-1,62	-2,82
A_64_P028379		1,59E-12	0,706344701	1,66	3,20	1,93
A_64_P028627	Appl1	8,51E-13	0,622874062	1,10	-1,05	-1,50
A_64_P028788	Mcrip2	2,13E-14	0,714876369	1,03	-1,04	-1,98
A_64_P029077		5,18E-11	0,701352264	-1,74	-1,44	2,91
A_64_P029213	H1f0	9,58E-14	0,676378639	-1,07	2,51	4,95
A_64_P029282	Man1c1	1,13E-16	0,764914128	1,34	1,23	2,80
A_64_P029337	F8a1	1,65E-08	0,620535661	-1,08	-1,06	-1,15
A_64_P029476	Ptpn22	1,79E-23	0,855173373	-1,12	1,92	8,75
A_64_P029482		4,36E-11	0,62197362	-1,25	2,85	3,57
A_64_P029501	Tnnc1	2,40E-17	0,82220434	-1,03	1,05	1,76
A_64_P029785	Olr203	9,82E-16	0,702549578	-1,65	2,52	8,84
A_64_P029805	Junb	1,32E-21	0,868576206	1,89	-1,11	-1,93
A_64_P029878	Clcnkb	2,67E-12	0,635173704	1,49	1,56	1,81
A_64_P029887	Focad	5,14E-14	0,742969187	1,33	1,46	3,60
A_64_P029932	Tmie	5,43E-28	0,88950975	1,09	1,88	3,25
A_64_P030090		1,29E-13	0,672900604	1,63	2,41	4,55
A_64_P030180	Mmd	3,13E-15	0,752645527	1,02	1,04	-1,57
A_64_P030362	Ephb3	1,55E-21	0,856489752	-1,24	1,41	2,53
A_64_P030464	Tnxb	9,12E-15	0,79003426	1,30	1,07	1,96
A_64_P030534	Tpcn2	3,46E-27	0,926439896	-1,25	1,44	3,53
A_64_P030614	Nyx	1,30E-12	0,668080894	-1,06	1,01	2,29
A_64_P030644	Inhbb	1,29E-11	0,638705515	-1,37	1,33	1,82
A_64_P031046	Draxin	1,88E-15	0,757353941	1,82	1,42	5,54
A_64_P031058	Trpm6	5,90E-17	0,770434509	-1,06	1,46	2,37
A_64_P031190		5,58E-21	0,822086901	-1,03	1,16	3,43
A_64_P031219	Rad9b	1,66E-16	0,74255178	-1,28	1,02	3,13
A_64_P031476	Slc26a9	3,81E-11	0,623857616	-1,09	1,64	3,72
A_64_P031511		5,77E-21	0,871651749	1,14	1,08	3,64
A_64_P032037	Uri1	1,56E-16	0,762093057	1,08	-1,19	-1,52
A_64_P032127	Ttll7	4,24E-11	0,685651324	1,54	1,19	-1,52
A_64_P032148		4,14E-18	0,791770714	1,31	3,62	7,55
A_64_P032257		9,44E-21	0,846479566	1,14	2,54	4,46
A_64_P032302	ago-01	1,88E-18	0,761206109	1,01	-1,18	-2,12
A_64_P032358	Mustn1	3,28E-15	0,811870887	-1,28	1,21	1,42
A_64_P032574	Hk2	4,56E-11	0,621340636	1,51	1,00	1,28
A_64_P032806	RGD1308106	1,81E-12	0,704846971	1,11	1,49	1,32
A_64_P032904	Mbnl2	9,58E-18	0,801030513	-1,00	-1,02	1,73
A_64_P033559	LOC679711	1,34E-26	0,915158253	1,24	-1,09	2,52
A_64_P033569	Tep1	2,24E-09	0,609879611	-1,13	1,11	1,67
A_64_P033795	Hgd	1,73E-20	0,866050272	-1,59	3,06	7,65
A_64_P033845	Lmo1	1,67E-23	0,897760515	-1,01	-1,10	1,44
A_64_P033901	Pars2	3,98E-14	0,727714416	1,00	1,91	1,99
A_64_P033979	Cdh22	1,25E-20	0,831689322	1,01	1,07	2,17

A_64_P034155	Ccdc166	2,48E-29	0,94982725	1,45	9,08	4,71
A_64_P034466	Nkain1	8,92E-24	0,858738976	-1,09	1,11	1,93
A_64_P034541	Rnf225	2,20E-17	0,809366484	-1,02	4,62	4,47
A_64_P034684	Rpain	1,05E-09	0,621332606	-1,24	1,01	1,28
A_64_P034744	Acrbp	1,18E-13	0,716294252	1,01	-1,10	2,10
A_64_P035041	Zfp111	2,32E-18	0,796167436	-1,05	1,18	1,48
A_64_P035192	Kif26a	3,70E-14	0,709015652	1,11	1,42	3,31
A_64_P035237	LOC363337	1,69E-13	0,692223795	-1,25	-1,05	1,97
A_64_P035238		3,90E-29	0,93816837	1,17	1,36	3,24
A_64_P035663		4,35E-27	0,918710049	-1,28	-1,16	1,90
A_64_P035719	Dnajb3	3,75E-19	0,809325802	-1,16	1,42	2,08
A_64_P036083	LOC367516	6,36E-31	0,941852305	1,19	1,47	4,06
A_64_P036174	Slc25a47	7,70E-26	0,900854478	1,15	3,42	10,62
A_64_P036514	Chrm2	9,87E-15	0,74168803	1,91	1,68	-2,52
A_64_P036576	RGD1563667	1,39E-14	0,738307693	-1,38	1,04	5,03
A_64_P036676	Klf6	1,27E-10	0,671536587	1,31	-1,87	-1,61
A_64_P036700	Hist1h1c	9,47E-28	0,907552926	1,83	3,94	4,93
A_64_P036770		1,13E-12	0,66980167	1,74	2,37	1,90
A_64_P036900	Kcnk3	3,53E-14	0,709519153	1,19	1,24	1,42
A_64_P036991	Cpz	2,20E-14	0,693064263	-1,14	-1,13	2,13
A_64_P037229		8,99E-12	0,618821575	1,06	1,51	1,12
A_64_P037245	Shisa2	8,57E-30	0,929349475	1,01	-1,54	-5,71
A_64_P037259	Ankar	1,85E-16	0,807345913	-1,59	-1,04	5,37
A_64_P037328		1,39E-11	0,612814071	-1,02	-1,10	-2,26
A_64_P037567	Il25	7,02E-20	0,787211908	-1,34	1,76	5,26
A_64_P037626	Bricd5	6,49E-13	0,676553598	1,09	1,00	-1,35
A_64_P037758	Stk24	3,57E-11	0,647591376	-1,09	1,15	2,20
A_64_P038352	Appl2	5,42E-15	0,747451112	1,02	1,14	1,62
A_64_P038625		1,89E-14	0,768810676	-1,01	1,14	1,54
A_64_P038767	Aste1	5,98E-14	0,741444707	1,08	-1,38	-2,73
A_64_P038839		1,31E-11	0,717790094	-1,35	1,98	4,03
A_64_P038902		2,96E-17	0,820740995	1,11	1,08	1,31
A_64_P038926	Prodh1	3,28E-09	0,603956249	1,04	1,23	1,44
A_64_P039006	Ccdc116	3,89E-22	0,851754294	1,91	3,35	3,75
A_64_P039165	Gcat	4,12E-12	0,629404963	1,05	1,42	1,65
A_64_P039342	Arhgap8	1,13E-13	0,674438069	1,12	1,10	1,52
A_64_P039432	Clk1	2,34E-10	0,621364981	1,35	-1,80	-1,70
A_64_P039737	Scx	6,02E-27	0,917697816	1,10	1,50	5,77
A_64_P039809		1,11E-28	0,935609318	1,06	1,31	4,51
A_64_P039834		3,45E-26	0,894589508	1,19	1,39	4,03
A_64_P039911		7,85E-14	0,700825808	1,21	1,79	2,56
A_64_P040095		1,07E-13	0,735517546	1,06	1,13	1,90
A_64_P040317	LOC102549583	8,70E-12	0,643918449	-1,38	-1,27	1,78
A_64_P040320		2,11E-18	0,825765616	1,07	2,20	3,48
A_64_P040351	LOC102553962	1,27E-13	0,673046515	-1,33	1,35	1,82
A_64_P040528	Zbtb22	1,91E-16	0,760327478	-1,13	-1,26	-1,67
A_64_P040535	Zbtb32	1,11E-21	0,816038057	-1,41	1,99	7,68
A_64_P041104	Dpf1	2,70E-16	0,737999302	1,13	1,01	1,46
A_64_P041184	Vipr2	1,08E-14	0,740845663	-1,04	1,16	2,77
A_64_P041205	LOC108349320	7,97E-19	0,803993611	-1,68	-1,12	10,06
A_64_P041208		1,98E-19	0,813751679	-1,23	2,35	3,55
A_64_P041219	Pcsk1	1,17E-20	0,832064465	1,10	-1,92	-4,93
A_64_P041228	Igsf3	1,54E-10	0,603800134	1,01	-1,02	2,33
A_64_P041498	Pla2g4f	9,96E-20	0,818380465	-1,05	1,21	2,27
A_64_P041996	Stac	4,59E-20	0,84949569	1,20	-1,06	-3,27
A_64_P042142	Prr35	6,88E-28	0,899355824	-1,15	1,35	7,67
A_64_P042249	Acvr1c	8,24E-19	0,844208698	1,26	1,11	-1,77
A_64_P042278	Crem	6,48E-21	0,860348992	1,20	-1,46	-2,52
A_64_P042283	Crem	6,31E-27	0,900924146	1,14	-1,52	-2,34
A_64_P042684	Zbtb24	3,70E-12	0,630822107	1,31	1,56	1,84
A_64_P042885	Fn3k	1,83E-10	0,62491671	1,07	1,07	1,50
A_64_P042930	Hcfc2	1,26E-13	0,695569389	1,04	-1,09	-1,59
A_64_P043033	Cbr3	9,66E-11	0,693596233	-1,13	-1,12	-1,48
A_64_P043259	P2rx6	7,80E-16	0,747582023	1,07	-1,04	1,49
A_64_P043361	RGD1307443	7,01E-17	0,800690543	1,62	1,31	1,89
A_64_P043392	Megf11	8,18E-26	0,916843262	1,09	1,16	2,32
A_64_P043411	Lrrc26	1,18E-23	0,857335434	-1,06	2,17	3,54
A_64_P043421		4,06E-26	0,903190671	-1,12	1,17	3,63
A_64_P043561	LOC494538	1,35E-11	0,660441803	1,62	1,80	2,67
A_64_P043614	Slc39a5	2,85E-19	0,850548775	-1,31	1,43	3,01
A_64_P043731	Vps37b	2,47E-10	0,620543465	1,04	1,02	1,52

A_64_P043776	Fat4	1,63E-16	0,821469821	1,20	-1,20	-3,11
A_64_P044087	Nap1l5	1,15E-10	0,631453313	1,05	1,17	1,18
A_64_P044101	Mgme1	8,31E-15	0,7433596	-1,28	1,68	4,17
A_64_P044177	Zswim7	6,65E-20	0,787620743	1,02	1,14	1,60
A_64_P044310	Lgals7	8,23E-13	0,650313757	-2,39	1,14	2,72
A_64_P044440	Fam43a	7,27E-22	0,848319439	1,15	-1,35	-6,62
A_64_P044445	Fam43a	4,00E-16	0,711779333	-1,02	-1,53	-5,18
A_64_P044470	Mrpl50	3,42E-12	0,677675589	-1,04	2,22	3,31
A_64_P044776	Itih4	3,29E-20	0,810406586	1,13	2,02	6,13
A_64_P045022	Arhgap42	1,91E-10	0,600652771	1,25	1,00	-1,50
A_64_P045062	Otog	2,00E-13	0,667678697	1,01	1,09	2,03
A_64_P045122	Mesp1	1,14E-27	0,915240915	-1,55	2,37	9,74
A_64_P045141	Spatc1	5,73E-18	0,78928297	-1,56	3,12	4,97
A_64_P045300	Tesk1	1,03E-12	0,647523237	1,01	-1,03	-1,93
A_64_P045305		5,01E-20	0,822892511	1,10	-1,39	3,07
A_64_P045353		3,21E-13	0,705343552	2,73	5,28	10,59
A_64_P045779	Plcl2	5,86E-25	0,883097179	1,03	-1,11	-2,44
A_64_P046082	RGD1560585	3,25E-11	0,626073486	1,78	1,78	3,09
A_64_P046120		1,25E-12	0,668606284	1,83	3,54	5,14
A_64_P046205	LOC685125	1,45E-17	0,781983783	1,14	1,13	2,44
A_64_P046358	LOC102546495	5,89E-16	0,730549507	-1,53	1,53	3,32
A_64_P046552	Sdcbp2	5,78E-25	0,910289906	1,41	2,71	12,88
A_64_P046754	Igfbp3	2,17E-18	0,779648862	1,55	-1,04	-4,16
A_64_P046853	Il17ra	1,85E-16	0,777631235	-1,01	1,11	1,45
A_64_P046885	LOC363337	2,06E-22	0,877558882	1,28	1,50	2,35
A_64_P047131		3,33E-10	0,616218506	-1,79	1,67	5,70
A_64_P047201	Tmem181	1,55E-16	0,762144722	1,07	1,32	1,95
A_64_P047386		1,46E-08	0,622422426	1,30	2,86	1,58
A_64_P047445	Tnfaip8	3,69E-20	0,824875692	1,06	1,16	-1,97
A_64_P047730	Eif1	5,38E-18	0,819392156	-1,13	-1,20	-1,71
A_64_P047774		1,23E-10	0,607071514	-1,02	1,32	3,00
A_64_P048004	Bfsp2	2,81E-07	0,631321851	-1,11	-1,12	1,84
A_64_P048033	Agpat3	5,85E-14	0,704048984	1,07	1,19	2,43
A_64_P048043	Krt7	1,63E-26	0,906433171	-1,29	-1,08	4,27
A_64_P048136	Brca2	1,46E-14	0,718854165	1,13	1,28	2,91
A_64_P048235	Tmem42	3,66E-14	0,687410429	1,08	1,21	1,94
A_64_P048481	Dmrtc1b	3,79E-25	0,894794042	1,03	1,27	2,18
A_64_P048516	Kctd8	2,86E-13	0,663370499	1,46	1,08	-1,94
A_64_P048606	Rabgef1	3,40E-13	0,661262217	-1,07	-1,33	-1,51
A_64_P048668	Pla2g2c	1,60E-21	0,829873056	-1,33	1,20	4,42
A_64_P048705	LOC100363225	3,35E-24	0,903957109	1,21	1,30	2,52
A_64_P048765	Cbs	3,76E-11	0,738367648	1,42	1,04	-1,05
A_64_P048852		5,35E-10	0,651864183	-1,06	-1,15	-1,44
A_64_P048897	Tmem60	3,09E-16	0,773297062	-1,04	-1,20	-1,51
A_64_P049153		9,80E-18	0,78508355	-1,72	1,15	3,15
A_64_P049161		2,31E-24	0,877095005	-1,09	1,18	2,15
A_64_P049401	RGD1310507	2,20E-25	0,876273156	1,09	1,48	5,82
A_64_P049648	RGD1562963	1,93E-18	0,797522739	1,22	1,60	3,22
A_64_P049808	Nptx2	8,40E-27	0,889926847	-1,11	1,29	2,36
A_64_P049897		4,14E-30	0,916182869	1,14	3,07	6,07
A_64_P049921	Sned1	2,44E-13	0,665288837	1,39	1,33	2,73
A_64_P049948	Dkk1	2,05E-20	0,82860107	1,77	1,34	-11,81
A_64_P050030	Nptxr	7,52E-10	0,626335299	-2,16	-1,31	1,84
A_64_P050052	Pde9a	1,75E-26	0,921675356	-1,02	-1,02	3,45
A_64_P050079	Zfp951	3,16E-12	0,657017312	1,11	1,91	1,29
A_64_P050099		9,38E-25	0,908589895	-1,11	-1,47	-1,23
A_64_P050145		1,53E-13	0,644883184	1,19	1,65	1,45
A_64_P050171	Sema4c	4,86E-17	0,80346452	-1,24	1,86	3,94
A_64_P050430	LOC108348293	3,84E-15	0,711761186	1,46	1,42	1,63
A_64_P050802	Syngr3	3,45E-12	0,655933368	1,11	-1,11	-1,99
A_64_P050994	Sema4a	1,41E-21	0,830666738	1,04	1,56	2,86
A_64_P051082	Wfdc6a	1,99E-17	0,761417836	-1,31	1,22	4,29
A_64_P051098	LOC102554371	6,36E-18	0,788465124	-1,18	2,43	4,63
A_64_P051104	Lmx1a	2,03E-10	0,623436972	1,23	1,08	1,49
A_64_P051125	Bpifb3	7,97E-12	0,62047621	-1,33	-1,24	1,60
A_64_P051139		1,72E-16	0,778251143	1,20	1,57	2,90
A_64_P051208	Mcm8	3,88E-14	0,686733426	1,01	1,42	1,72
A_64_P051272	Zfp775	1,18E-14	0,699853731	-1,14	1,59	3,29
A_64_P051430	Gpx2	2,46E-14	0,691822236	-1,11	2,31	3,57
A_64_P051507		1,44E-32	0,954449804	1,26	1,25	2,47
A_64_P051631	Pde4b	8,83E-14	0,737525276	1,72	1,06	-2,89

A_64_P051816	Bbof1	5,10E-13	0,629598754	-1,07	1,21	2,38
A_64_P051836		4,21E-18	0,791643775	-1,80	-1,18	5,13
A_64_P051967	LOC103690296	1,33E-11	0,638240719	-1,73	1,46	3,41
A_64_P052422	Otud3	7,18E-14	0,701801938	1,04	1,08	-1,30
A_64_P052770	Cdh11	4,09E-15	0,78237378	1,32	1,43	-2,41
A_64_P052882	Cdpf1	3,89E-13	0,703195852	-1,27	1,20	2,18
A_64_P052892	LOC108349386	7,02E-24	0,871991719	1,28	1,50	4,10
A_64_P053084	Grifin	8,37E-27	0,916660572	1,13	1,27	4,92
A_64_P053353	Jun	4,21E-16	0,753233012	2,50	2,10	5,68
A_64_P053392	Kiaa0408L	1,62E-14	0,696453106	-1,09	-1,06	-1,30
A_64_P053461	Bag2	3,63E-22	0,884759581	-1,26	-1,12	-2,90
A_64_P053531	RGD1310819	2,42E-19	0,796327791	1,29	1,02	1,94
A_64_P053785	Adra2a	8,59E-14	0,719683358	-1,42	1,28	3,92
A_64_P053861	Ppp4r4	7,77E-21	0,834582447	1,05	1,23	3,07
A_64_P053979		7,09E-19	0,788330071	1,14	1,75	2,80
A_64_P054153	Nudt6	6,76E-25	0,909741906	-1,01	1,75	3,28
A_64_P054193	Rsrp1	3,73E-11	0,647014881	-1,17	-1,32	-1,41
A_64_P054197		3,43E-22	0,852434707	1,27	2,36	3,97
A_64_P054337	Efna2	8,41E-16	0,727068831	1,22	1,55	1,29
A_64_P054441	Adra2c	5,46E-10	0,608862172	-1,07	1,21	1,46
A_64_P054461	Bhlha9	3,80E-12	0,654698106	-1,61	1,81	1,74
A_64_P054626	Hcn4	6,60E-11	0,616102619	1,70	1,15	1,37
A_64_P054765	Eml1	2,93E-17	0,792491885	-1,07	1,07	2,31
A_64_P054856	Wdr25	2,80E-16	0,77412999	1,06	1,06	1,98
A_64_P055112		1,65E-14	0,717529892	-1,19	-1,51	3,05
A_64_P055135		5,12E-18	0,819737331	-1,22	-1,25	-1,61
A_64_P055279	Tmem121	1,24E-10	0,630388127	1,01	-1,26	-1,74
A_64_P055313	Disp3	3,97E-24	0,903319379	1,39	2,00	3,56
A_64_P055433	Tmem131	2,04E-10	0,665189688	1,68	1,42	-1,51
A_64_P055634	LOC100363225	7,30E-14	0,679527709	1,04	1,04	2,17
A_64_P055699	Timm9	2,32E-21	0,876129468	-1,04	-1,16	-2,75
A_64_P055738	Itgb4	1,72E-14	0,671121573	1,14	1,29	2,05
A_64_P055763	Rhbdl1	6,96E-14	0,721907806	1,15	1,01	-2,18
A_64_P056247	Msh5	5,64E-19	0,790057606	1,39	1,95	4,15
A_64_P056426		7,39E-29	0,923456696	1,11	1,45	3,26
A_64_P056427		1,56E-33	0,948694332	1,10	1,27	3,63
A_64_P056648	Crlf1	6,24E-10	0,629081022	-1,40	-1,07	1,35
A_64_P056861	Slc30a8	2,94E-14	0,664878436	1,20	2,00	4,65
A_64_P057295		2,70E-19	0,811625804	1,28	1,39	1,24
A_64_P057397		1,10E-12	0,710543664	1,04	1,08	-1,33
A_64_P057516	Mrto4	1,21E-12	0,645449748	1,02	1,09	-1,22
A_64_P057587	Kcnn1	4,02E-15	0,731872176	-1,44	1,01	5,52
A_64_P057877	Aatk	1,61E-14	0,696521698	1,28	-1,00	1,58
A_64_P057975	Hmgn1	3,91E-11	0,623522475	1,13	1,22	1,20
A_64_P058015	Gimap1	3,22E-16	0,788647615	1,04	1,67	2,84
A_64_P058087	Rbp4	1,92E-18	0,861402225	-1,02	1,23	1,92
A_64_P058280	En1	3,14E-26	0,88461529	1,09	-1,58	-3,55
A_64_P058539	Zmynd19	1,05E-15	0,724869232	-1,11	-1,15	-2,73
A_64_P058749		1,13E-16	0,764894502	1,10	-1,07	-1,71
A_64_P058878	LOC102554532	1,81E-12	0,664022576	-1,17	1,35	1,61
A_64_P058988		4,30E-17	0,77309383	-1,56	1,49	2,82
A_64_P059046	Arrdc3	3,25E-11	0,626068131	3,54	5,53	3,95
A_64_P059081		2,03E-15	0,71828913	1,04	1,24	1,60
A_64_P059120	Aen	8,26E-13	0,694607849	-1,14	-1,09	-1,52
A_64_P059192	Cdc42ep5	1,54E-09	0,615622588	-1,33	1,01	1,29
A_64_P059456	Plcd3	1,12E-19	0,801898661	1,03	1,19	3,56
A_64_P059495	Ddit3	1,66E-19	0,874595564	1,10	1,21	2,50
A_64_P059580	Atp6v1e2	3,51E-13	0,660893146	1,01	-1,00	1,57
A_64_P060094	Lrrc2	2,57E-14	0,749773262	-1,13	-1,36	-1,51
A_64_P060202	Luc7l3	1,49E-24	0,867515655	1,02	-1,40	-2,87
A_64_P060302	LOC365085	1,23E-08	0,644610112	-1,25	-1,28	1,12
A_64_P060303	Lsr	3,46E-11	0,625214302	1,05	1,17	2,82
A_64_P060319	Gsg1l	3,44E-21	0,852160841	-1,23	1,48	4,42
A_64_P060608		1,90E-24	0,888270089	1,25	1,90	6,20
A_64_P060804	Dcbld1	2,33E-13	0,688557791	1,24	-1,03	-2,76
A_64_P061291	Micall1	1,59E-12	0,724349046	1,20	1,25	-1,07
A_64_P061323	Kcna10	3,21E-12	0,698227306	-1,31	1,03	4,45
A_64_P061543	Cby3	7,44E-20	0,786776436	1,08	1,73	2,63
A_64_P061590	LOC688126	2,67E-15	0,735894475	-1,29	2,28	5,69
A_64_P061615	Slc6a7	2,85E-36	0,954788591	-1,10	1,78	19,28
A_64_P061740	Trpv1	3,52E-15	0,797959193	-1,10	2,51	4,65

A_64_P061746	Nlrx1	9,49E-17	0,747649429	-1,19	1,16	3,12
A_64_P061765	Fam89a	7,92E-10	0,625570122	-1,34	-1,08	-1,38
A_64_P061770	Psrc1	3,09E-18	0,776836177	-1,07	1,38	3,92
A_64_P061830	Fam84a	3,91E-14	0,686656271	1,14	1,09	-2,05
A_64_P061835	Fam84a	2,03E-15	0,718263809	1,14	-1,06	-1,94
A_64_P062048	Znf354b	1,67E-11	0,67818764	-1,04	2,20	2,49
A_64_P062223	Terb1	8,71E-22	0,880769653	1,19	1,50	3,79
A_64_P062285	Vpreb2	4,72E-10	0,633133088	-1,30	1,05	2,27
A_64_P062388		8,78E-14	0,719459026	-1,19	-1,25	-2,19
A_64_P062506		6,86E-28	0,930909909	-1,06	1,21	2,66
A_64_P062570		2,78E-16	0,715434629	1,37	3,79	8,93
A_64_P062593	Chst5	1,26E-20	0,867689371	-1,13	1,80	7,24
A_64_P062608	Chst7	1,19E-16	0,745616527	1,03	1,03	1,28
A_64_P062618	Chst1	3,14E-15	0,713832727	1,18	1,84	3,02
A_64_P062638	Ptger1	5,68E-24	0,861007486	-1,31	1,40	3,85
A_64_P062670	Chst8	7,89E-21	0,847508578	1,67	1,73	3,37
A_64_P062890	Oaz3	7,54E-18	0,817038035	-1,06	2,00	4,42
A_64_P062965	Ghrh	5,99E-13	0,627497399	1,20	1,25	2,01
A_64_P063019	Aox4	1,37E-17	0,798331621	-1,11	1,57	4,61
A_64_P063035		5,10E-13	0,719046783	1,24	2,50	2,93
A_64_P063047	Rpp25	5,39E-10	0,722614254	-1,00	1,19	1,46
A_64_P063309	Dennd2a	1,57E-13	0,713238322	1,06	1,21	1,69
A_64_P063353	Fam83e	8,67E-19	0,767596275	-1,12	2,09	7,16
A_64_P063398	Ptger3	4,08E-17	0,789910346	1,99	1,53	-13,70
A_64_P063636	RGD1562660	2,97E-11	0,627311826	1,11	1,50	2,67
A_64_P063644		1,09E-12	0,710649678	-1,14	1,13	1,39
A_64_P063953	Xkr7	1,28E-13	0,673030475	1,33	-1,30	-6,29
A_64_P063962	LOC108350705	3,76E-12	0,654818613	-1,13	-1,12	1,97
A_64_P063993	Ttc4	5,39E-19	0,85799654	1,16	1,49	1,66
A_64_P064128		2,31E-18	0,796176766	1,59	2,93	4,08
A_64_P064129	Igsf3	5,42E-18	0,789714358	1,35	3,36	3,70
A_64_P064134	Prss27	5,37E-12	0,625848273	-1,61	-1,62	4,50
A_64_P064321	Scrn3	2,39E-17	0,794088871	1,30	1,88	3,36
A_64_P064371	Spata2L	9,54E-17	0,783086503	1,34	-1,99	-2,19
A_64_P064498		1,90E-17	0,779807517	1,43	-1,17	-3,43
A_64_P064531	Npas4	6,04E-37	0,965604441	-1,28	-11,79	-58,19
A_64_P064606	Dll3	4,02E-20	0,809031028	1,37	1,75	3,75
A_64_P064660	Ndel1	1,33E-12	0,616919161	-1,07	-1,38	-1,94
A_64_P064820	Cacng1	8,91E-17	0,726578819	-1,14	2,22	3,44
A_64_P064866	LOC100909776	1,58E-20	0,855506879	-1,48	1,56	3,68
A_64_P065032	P3h4	6,25E-10	0,669002395	-1,09	1,15	1,72
A_64_P065063	Fosl2	3,23E-12	0,678385535	1,12	-1,98	-2,70
A_64_P065102	Evpl	1,83E-19	0,841316455	1,23	1,32	3,58
A_64_P065131	LOC102555083	3,03E-12	0,679144153	1,01	1,67	1,82
A_64_P065165		5,89E-20	0,821845142	1,29	1,40	2,82
A_64_P065280	Fam151a	2,88E-25	0,86230026	1,10	2,96	5,75
A_64_P065301	LOC102554444	3,24E-20	0,862710149	-1,03	1,05	3,34
A_64_P065368	Acot4	3,50E-20	0,792354206	1,31	2,62	2,96
A_64_P065492	RGD1565222	6,39E-17	0,75121023	-1,49	1,21	8,64
A_64_P065629	LOC102552540	7,93E-29	0,930172759	1,29	1,29	2,18
A_64_P065691	Adat3	4,29E-12	0,653141304	1,02	-1,62	-2,02
A_64_P065868	Acvr1c	5,04E-29	0,931364524	1,03	-1,19	-2,17
A_64_P065939	B3galt2	4,03E-13	0,682192739	-1,05	-1,13	-1,22
A_64_P066081	Kcnh2	6,61E-22	0,835183906	-1,20	-1,05	2,74
A_64_P066181		6,38E-13	0,653514405	1,14	-1,00	1,25
A_64_P066544	Smim3	1,24E-10	0,607029104	1,03	1,17	1,25
A_64_P066577		6,13E-27	0,917641431	1,07	1,06	2,57
A_64_P066586		3,37E-16	0,755264585	-1,10	1,23	1,39
A_64_P066782	LOC108348347	1,80E-25	0,928167651	1,12	1,25	5,36
A_64_P067000	Prima1	9,86E-27	0,923393256	1,12	1,17	6,83
A_64_P067143	Spata31d1d	5,23E-12	0,650576173	1,90	1,68	1,37
A_64_P067366	Cacna1h	2,24E-14	0,733560864	-1,01	2,32	5,04
A_64_P067447	Acot4	4,05E-22	0,851535284	1,30	2,49	2,80
A_64_P067654	Aldh3a1	9,85E-13	0,711780211	-1,19	1,02	1,51
A_64_P067868	B3gnt9	1,43E-10	0,604966247	-1,04	1,09	2,03
A_64_P067883	Trim47	4,45E-23	0,874322221	1,12	1,63	3,19
A_64_P067884	Trim47	8,68E-27	0,9085981	1,05	1,40	3,07
A_64_P067903	LOC108350705	6,55E-20	0,787735791	1,12	1,93	3,52
A_64_P067908	Trim17	2,16E-34	0,957036291	2,13	9,22	57,95
A_64_P067915	Nudt8	5,63E-10	0,670435834	-1,23	1,16	1,63
A_64_P068038	Hexb	3,69E-14	0,687298279	1,16	1,04	-1,34

A_64_P068152	Smad6	2,75E-22	0,886008758	-1,04	1,20	-1,16
A_64_P068237	Fam149a	3,87E-11	0,64651287	1,18	1,06	-1,77
A_64_P068389	Mast3	1,85E-12	0,612420079	1,19	1,38	1,97
A_64_P068458	Emc1	9,39E-15	0,702328429	1,64	2,64	2,66
A_64_P068548	Arhgap22	5,36E-24	0,883866848	-1,62	-1,30	6,25
A_64_P068594	Car6	4,03E-14	0,708092803	-1,01	1,22	3,56
A_64_P068786	Zfp629	1,48E-13	0,693703745	-1,11	-1,52	-1,64
A_64_P068801	Zranb2	7,68E-19	0,787721651	1,05	-1,03	-1,98
A_64_P068965	Sowaha	7,49E-13	0,674821415	1,10	1,71	2,56
A_64_P069071	Lpar6	1,52E-08	0,601093345	-1,02	-1,18	1,42
A_64_P069081	RGD1310495	2,54E-10	0,681111588	1,35	2,14	2,33
A_64_P069136		4,81E-16	0,73249857	1,06	1,38	2,78
A_64_P069211	Cdc42bpg	1,36E-09	0,617470588	1,05	1,46	2,66
A_64_P069261	Arl4d	5,48E-11	0,641784946	1,16	-1,01	2,21
A_64_P069374	Scn10a	3,24E-28	0,938869208	-1,06	1,36	5,06
A_64_P069394	Styxl1	2,22E-19	0,81294887	-1,13	1,10	2,11
A_64_P069462		4,54E-25	0,873030815	1,04	3,47	5,97
A_64_P069654	Smtn	2,08E-17	0,77908298	1,20	1,27	2,47
A_64_P069664	Eya2	1,42E-13	0,694206678	-1,17	-1,11	3,42
A_64_P069739	Ankrd23	3,02E-19	0,810836666	-1,18	1,91	2,93
A_64_P069789	LOC363337	6,56E-23	0,84827438	-1,43	2,33	5,66
A_64_P069819	Rrp8	2,14E-11	0,654370151	1,01	-1,11	-1,58
A_64_P069942	Prss56	2,67E-20	0,852578277	-2,23	1,62	6,67
A_64_P070068	LOC501416	9,37E-23	0,870783748	-1,01	1,43	2,43
A_64_P070184	Exosc9	4,94E-13	0,700493654	1,02	1,09	-1,89
A_64_P070628	Cfap57	5,94E-14	0,703883441	-1,98	1,18	5,98
A_64_P070713	Dll3	6,50E-12	0,623248535	1,06	1,10	2,17
A_64_P070756	Anxa11	9,70E-15	0,677666522	1,08	1,24	1,74
A_64_P070891	Cacng6	1,40E-12	0,688399269	1,00	1,10	1,38
A_64_P070937	LOC501346	3,43E-15	0,798174992	-1,25	-1,42	4,77
A_64_P071178	Mgat5b	2,27E-16	0,758798904	-1,05	1,16	1,91
A_64_P071810	Gpr143	1,31E-08	0,603506634	1,20	1,25	1,35
A_64_P071983	Ppp1r3c	5,27E-10	0,65207486	1,42	1,11	-1,64
A_64_P072035	Ighmbp2	2,90E-26	0,884937523	-1,03	1,12	2,84
A_64_P072088	Lrrtm2	1,26E-11	0,639021619	1,09	1,25	1,68
A_64_P072227	Arrdc2	3,16E-12	0,749714612	-1,65	1,84	4,38
A_64_P072343	LOC501317	5,37E-27	0,918056731	1,18	1,49	3,83
A_64_P072558	Grm6	4,54E-17	0,732969049	1,22	1,67	4,21
A_64_P072748	Cage1	7,28E-10	0,604523064	-1,09	-1,02	1,75
A_64_P073550	March11	6,27E-13	0,653721804	-1,16	-1,08	-3,20
A_64_P073614	Lin7a	3,04E-13	0,662639732	1,20	1,28	-1,58
A_64_P073845	Cysrt1	1,36E-21	0,857223117	-1,13	1,88	2,95
A_64_P073944	Sox11	1,15E-09	0,620014492	-1,03	1,31	2,24
A_64_P074102	Wnt2b	2,44E-13	0,70840093	1,42	1,44	1,55
A_64_P074127	LOC685203	5,51E-28	0,924840347	1,04	-1,05	-4,36
A_64_P074267	Pnrc1	5,69E-12	0,671395352	-1,06	-2,62	-1,30
A_64_P074357		2,64E-23	0,876759026	1,09	1,22	2,17
A_64_P074440	Rbm24	1,82E-23	0,855092303	1,25	-1,40	-3,27
A_64_P074544	Ankrd61	1,37E-14	0,698264863	1,44	1,23	1,32
A_64_P074851	Atp4a	4,41E-15	0,710314455	-1,09	1,20	2,29
A_64_P074914	Prtn3	4,01E-26	0,871505172	-1,09	2,37	6,83
A_64_P075008	Birc7	1,25E-23	0,880148773	-1,18	1,49	3,36
A_64_P075087	Camk1g	5,37E-15	0,807989105	1,30	-1,05	-1,41
A_64_P075124		4,48E-11	0,621594328	1,07	-1,06	-1,94
A_64_P075357	Cyr61	2,92E-23	0,886349261	1,32	-1,65	-1,14
A_64_P076097	Obscn	4,64E-24	0,884490936	-1,25	-1,03	2,27
A_64_P076217	Bhlhe22	2,59E-20	0,827135438	-1,20	1,54	2,79
A_64_P076352	Hacl1	4,97E-12	0,673072582	-1,02	1,22	-1,25
A_64_P076436		1,63E-26	0,914533957	-1,01	1,30	3,43
A_64_P076450	Pomc	5,42E-14	0,704876497	-1,20	1,28	1,67
A_64_P076466	Pnpla1	9,37E-18	0,747348266	-1,10	1,23	6,42
A_64_P076555	Jph1	5,58E-15	0,779678788	1,80	1,45	-1,75
A_64_P076832	RGD1311447	9,47E-24	0,929122166	1,43	1,81	4,66
A_64_P076891	Nudt17	1,27E-12	0,617528549	-1,12	1,11	1,71
A_64_P076900	Nedd1	1,26E-11	0,700603248	1,10	1,30	1,33
A_64_P076997	RGD1561442	5,95E-11	0,617590205	-1,14	1,44	3,06
A_64_P077240	RGD1562660	2,28E-27	0,927624521	1,22	1,21	2,31
A_64_P077242		1,93E-13	0,729443644	1,01	1,06	1,78
A_64_P077392		4,74E-14	0,789882183	1,61	-1,18	1,84
A_64_P077806	Lipt2	1,97E-17	0,740667469	1,06	1,95	2,54
A_64_P077846	Vom2r47	5,20E-19	0,771726814	-1,32	1,43	1,95

A_64_P078140	Amigo2	2,56E-26	0,895726882	1,20	-1,02	-2,85
A_64_P078230	RGD1312005	2,04E-13	0,690099615	1,10	1,14	2,76
A_64_P078303	Angpt4	1,94E-17	0,810290381	-1,05	1,37	5,27
A_64_P078875		3,72E-13	0,68314265	1,22	1,52	2,31
A_64_P078894	Il23a	7,20E-28	0,908474381	-1,01	3,26	6,06
A_64_P079220	Gprin3	1,45E-22	0,868663831	-1,06	1,25	6,57
A_64_P079260	Ttc36	2,89E-13	0,686087039	-1,78	-1,33	-1,17
A_64_P079408	Wnt9b	9,82E-31	0,92038665	1,26	1,68	8,44
A_64_P079523		2,81E-21	0,853272474	-1,18	1,13	2,15
A_64_P079553	Myh8	8,46E-11	0,695269386	-1,33	-1,12	1,14
A_64_P079666	Cyp4f5	9,76E-22	0,870078772	1,34	1,15	-1,08
A_64_P079731	Zfyve21	4,41E-16	0,770228091	-1,04	-1,02	-1,32
A_64_P079822		7,41E-16	0,765706944	1,30	1,40	1,82
A_64_P080154	Klf11	9,18E-15	0,742391432	1,09	1,42	3,63
A_64_P080288	Kctd18	6,83E-14	0,654823301	1,07	1,41	1,44
A_64_P080329	Paxx	3,07E-11	0,649604763	-1,02	1,12	1,37
A_64_P080499		2,04E-13	0,690098339	-1,26	3,85	9,94
A_64_P080509	Fut1	2,11E-21	0,854848323	1,08	3,38	6,91
A_64_P080580		9,46E-22	0,880383957	-1,18	-1,08	3,26
A_64_P080667		1,95E-14	0,734965778	1,83	3,07	3,01
A_64_P080697	Cand2	2,07E-23	0,854406495	-1,13	1,18	2,86
A_64_P080941	LOC102556967	1,70E-14	0,695928876	1,12	1,08	1,77
A_64_P081063		1,14E-24	0,88021415	2,25	3,07	8,26
A_64_P081069		3,15E-20	0,810691066	1,13	5,20	7,36
A_64_P081217	Ptrh2	8,27E-12	0,619959854	-1,26	-1,27	-1,99
A_64_P081222	Ptrh2	8,25E-19	0,818509412	-1,03	-1,06	-2,10
A_64_P081247	Ankrd39	2,82E-21	0,809914459	-1,07	1,28	1,73
A_64_P081328	Hmgn2	1,38E-19	0,800411862	-1,07	1,25	1,79
A_64_P081352	Kcnc3	3,71E-14	0,66212992	1,40	1,79	1,77
A_64_P081546	Plag1	5,59E-13	0,678323807	-1,01	1,14	1,91
A_64_P081738	LOC108349423	1,67E-26	0,914440791	1,35	1,31	2,48
A_64_P081762	Trnau1ap	1,02E-12	0,711393897	-1,03	1,10	-1,30
A_64_P081788	Olfml3	4,53E-13	0,701480962	-1,13	1,21	2,14
A_64_P081822	Ticrr	4,62E-10	0,65392158	1,07	-1,26	1,71
A_64_P081977	Ppcdc	4,52E-10	0,611699692	-1,26	-1,19	1,43
A_64_P082032	Zfc3h1	2,92E-28	0,902390017	-1,22	-1,67	-3,77
A_64_P082356		2,66E-11	0,628830171	-1,33	1,32	1,25
A_64_P082386	LOC102553270	1,70E-09	0,654965418	1,51	1,12	-1,39
A_64_P082495	RGD1564937	2,76E-14	0,690556298	1,24	3,17	5,29
A_64_P082678	Nkd1	7,78E-12	0,62080605	1,18	-1,16	1,69
A_64_P082693	Egr1	1,15E-25	0,935547258	2,29	1,55	10,20
A_64_P082718	Otop2	4,48E-17	0,733096372	1,51	2,07	3,01
A_64_P082949	Ube2j2	7,18E-12	0,621897387	-1,04	-1,27	-1,61
A_64_P083029	Arhgef33	1,35E-25	0,878439536	1,36	3,30	8,34
A_64_P083034	Asmt	1,80E-31	0,938799441	1,22	3,76	4,87
A_64_P083277	LOC102548071	1,46E-12	0,61567968	1,27	1,32	1,68
A_64_P083329	Rmt1	3,05E-21	0,840152781	-1,16	2,55	4,07
A_64_P083559		5,56E-25	0,859083288	-1,61	4,04	14,35
A_64_P083610	Dbh	2,25E-28	0,903299565	-1,71	2,26	22,95
A_64_P083625	Klhl3	1,60E-11	0,635781415	1,02	1,23	1,95
A_64_P083944	Samd11	4,07E-13	0,702696285	1,22	1,55	1,14
A_64_P083978	Zfp68	4,01E-20	0,871983576	1,23	-1,16	-1,79
A_64_P084058	LOC685183	3,10E-27	0,919748315	1,53	2,02	5,67
A_64_P084059	LOC685183	8,02E-30	0,941839758	1,43	1,40	4,23
A_64_P084333	Lrfn4	8,23E-11	0,612946437	-1,03	-1,11	-1,56
A_64_P084458	Egfl8	4,80E-12	0,627363151	-1,16	1,01	1,47
A_64_P084473	Pear1	1,87E-16	0,760518042	-1,01	1,15	2,30
A_64_P084483	Immp1l	1,59E-13	0,692941888	-1,31	-1,06	-1,43
A_64_P084538	Tjp3	1,03E-23	0,870205378	1,16	1,16	3,09
A_64_P084851	Rcan2	8,73E-13	0,693956726	1,24	-1,02	-1,46
A_64_P085004	Kiss1r	4,66E-25	0,902905464	-1,25	1,63	4,31
A_64_P085058		8,57E-12	0,686455169	-1,38	1,07	3,05
A_64_P085530	Scd	8,23E-11	0,657453423	1,40	1,11	-1,86
A_64_P085635		3,89E-21	0,838714913	1,52	2,65	4,30
A_64_P085650	Slc22a14	1,69E-23	0,878775338	1,02	2,98	6,03
A_64_P085670	LOC108348910	1,51E-13	0,713635165	-1,28	-1,05	2,13
A_64_P085671		7,44E-20	0,868791872	1,35	1,45	2,75
A_64_P086041	Syndig1	1,22E-14	0,699488097	1,09	-1,14	-2,45
A_64_P086383	Mboat4	1,36E-16	0,744371883	-1,06	1,31	4,03
A_64_P086398		9,62E-12	0,664759813	1,11	1,08	1,28
A_64_P086404	Polr2h	3,75E-09	0,601873132	1,08	1,06	-1,64

A_64_P086445	Nr2c2ap	3,70E-13	0,683197713	-1,13	-1,35	-1,64
A_64_P086472	Fbxl19	2,45E-15	0,716384809	-1,01	1,07	-1,53
A_64_P086550		1,72E-11	0,634831095	1,77	2,00	2,79
A_64_P086565	Rras2	2,11E-18	0,779863239	-1,07	1,12	1,48
A_64_P086784	Runx1t1	9,06E-15	0,759637827	1,22	1,25	-1,25
A_64_P086989	Slc13a1	6,57E-13	0,653143464	-1,12	1,34	2,27
A_64_P087014	Slc18a1	9,44E-20	0,818736979	1,07	1,82	5,39
A_64_P087039		1,11E-22	0,880412924	1,10	1,21	2,19
A_64_P087040		1,09E-29	0,935241312	1,01	1,15	3,42
A_64_P087233	Pias4	6,60E-11	0,660403043	1,03	-1,17	-1,33
A_64_P087238	Harbi1	4,01E-13	0,682246231	1,10	1,60	2,46
A_64_P087380	Efna3	6,70E-25	0,882523927	1,07	2,09	6,30
A_64_P087424	F2rl1	2,57E-11	0,651999303	1,74	1,22	1,18
A_64_P087659	Prrt4	3,67E-18	0,75552013	-1,06	1,09	3,51
A_64_P087801	St14	1,09E-14	0,740759993	-1,14	1,63	3,82
A_64_P087842		1,48E-17	0,763956907	2,64	15,97	16,73
A_64_P087867	Sys1	2,80E-10	0,61873004	1,04	-1,07	-1,24
A_64_P087906	Abtb2	6,25E-22	0,835509713	-1,09	-1,43	-1,70
A_64_P088126	Mrps27	2,56E-12	0,659701206	-1,04	1,14	-1,90
A_64_P088382	Angptl4	6,78E-16	0,823697943	1,59	1,38	3,79
A_64_P088392	Hbb-b1	7,98E-09	0,734603203	1,45	-1,05	2,40
A_64_P088393	Hbb-b1	5,21E-09	0,638425709	1,60	-1,02	2,86
A_64_P088397	Sytl1	1,77E-22	0,912687538	-1,14	1,23	1,79
A_64_P088695	Tmtc4	2,48E-11	0,692356573	1,50	1,23	2,82
A_64_P088817	Carns1	9,34E-12	0,642977244	-1,47	1,14	1,72
A_64_P088995	LOC685668	2,13E-19	0,840413303	1,01	1,19	1,46
A_64_P089016	LOC108349297	1,11E-25	0,908064093	1,31	1,38	2,47
A_64_P089020	Ier5	3,11E-17	0,806814052	1,66	2,61	2,18
A_64_P089025	Ier5	1,93E-14	0,715905847	1,25	2,62	3,75
A_64_P089040	Ovol1	2,72E-20	0,883493769	1,70	4,67	-1,18
A_64_P089075	Nme4	1,63E-14	0,671765953	-1,56	1,54	2,24
A_64_P089201	Tcp11	1,70E-13	0,712326301	-1,54	-1,78	1,04
A_64_P089400	Hpse	6,60E-13	0,697187499	1,41	1,12	-1,76
A_64_P089457	Pcca	2,01E-20	0,828713221	-1,07	-1,03	-1,21
A_64_P089475		2,26E-15	0,717193487	1,07	1,22	1,36
A_64_P089637	Rnf223	2,37E-19	0,796487	-1,06	1,26	2,47
A_64_P089662	Atp8b2	1,13E-11	0,615611722	1,01	1,02	1,32
A_64_P089987	Tdrd7	6,56E-14	0,655307682	-1,04	1,02	-2,11
A_64_P090304	Ccdc88b	5,14E-26	0,931722153	-1,11	1,23	3,36
A_64_P090354	Syne3	9,22E-12	0,665305814	1,20	1,83	1,67
A_64_P090449		3,35E-13	0,704881567	-1,02	-1,13	1,72
A_64_P090866	Sstr1	4,91E-19	0,791093809	1,15	-1,22	-2,94
A_64_P091208		1,79E-17	0,796306017	1,04	1,87	5,27
A_64_P091618	Map3k14	1,81E-22	0,842643533	-1,07	1,29	2,68
A_64_P091662	RGD1306233	2,67E-20	0,794313907	-1,10	1,26	3,74
A_64_P091728	Slc22a1	8,33E-30	0,914060291	-1,02	2,93	7,56
A_64_P091886	Zfp207	1,29E-20	0,799496451	-1,08	-1,03	-1,75
A_64_P091940	Gal	1,19E-28	0,905467452	1,10	1,66	4,67
A_64_P091953	Spink4	8,86E-15	0,759845218	-1,01	-1,10	2,55
A_64_P092005	Stat2	1,96E-15	0,756980127	-1,05	1,15	1,79
A_64_P092026	Bhlha15	3,28E-15	0,733882844	1,16	1,78	1,09
A_64_P092067	LOC100363225	7,42E-19	0,804506018	1,48	1,34	1,87
A_64_P092122	Zmynd8	9,55E-09	0,6086459	1,23	1,02	2,54
A_64_P092534	Sspo	1,11E-24	0,915643325	-1,28	1,42	4,62
A_64_P092744	Wnt11	1,19E-14	0,720895342	-1,11	2,09	2,32
A_64_P092782	Wnt16	3,20E-13	0,66202555	1,77	2,37	4,13
A_64_P092971	Fndc5	2,10E-19	0,778883896	-1,07	1,41	3,22
A_64_P093026	Anks6	4,86E-12	0,693315995	1,37	1,30	-1,68
A_64_P093044		5,29E-18	0,844407699	1,12	1,74	1,46
A_64_P093389		6,39E-15	0,762856755	-1,08	1,21	2,06
A_64_P093452	RGD1305464	9,42E-22	0,846877533	1,02	1,04	3,01
A_64_P093522	Dtna	2,59E-12	0,700745232	1,10	-1,30	-1,42
A_64_P093899	Elf3	1,37E-16	0,722415398	1,22	2,53	3,26
A_64_P094107	Calhm3	2,13E-12	0,638127164	-1,22	-1,58	1,98
A_64_P094249	Card14	9,38E-15	0,742176208	1,02	-1,08	3,01
A_64_P094267	Pde6g	7,56E-13	0,624427159	1,30	1,80	2,11
A_64_P094362	Cabin1	2,36E-17	0,739027907	-1,01	-1,05	2,01
A_64_P094515	LOC100360619	1,87E-17	0,823946711	-1,04	1,01	1,53
A_64_P094722		2,72E-18	0,810199864	-1,20	1,66	2,82
A_64_P095005	Mmd2	2,72E-13	0,774060842	1,38	1,13	1,40
A_64_P095266		7,67E-14	0,720888951	1,43	3,16	1,90

A_64_P095586	LOC498316	1,23E-17	0,813574721	-1,49	1,16	2,27
A_64_P095830	Adgrg1	4,84E-14	0,706114556	1,19	1,01	1,53
A_64_P095900	Rgs12	4,03E-12	0,653928835	-1,06	1,25	1,95
A_64_P096006	Xrra1	1,30E-09	0,618170926	1,11	1,34	3,08
A_64_P096041		3,48E-26	0,894550562	1,63	1,39	5,35
A_64_P096083		1,24E-12	0,689852935	1,02	-1,19	-1,28
A_64_P096624	Oma1	2,29E-11	0,605711558	-1,03	1,06	1,94
A_64_P096647	Tmprss5	2,43E-14	0,667119031	1,10	1,37	2,42
A_64_P096748		1,16E-18	0,829713385	-1,26	1,23	5,74
A_64_P096763	Atoh8	9,29E-24	0,858537795	-1,16	2,60	5,60
A_64_P096782	Ier5l	6,49E-16	0,782876826	1,10	1,56	2,73
A_64_P096787	Plpp4	4,49E-15	0,781557368	1,00	1,08	1,44
A_64_P097024		1,39E-15	0,742119895	1,56	2,19	4,18
A_64_P097078	Pdp2	2,88E-19	0,795063155	1,17	-1,33	-3,35
A_64_P097218		4,26E-14	0,72700958	1,36	1,29	2,50
A_64_P097332	Vom1r73	3,01E-14	0,71121971	1,61	3,84	1,14
A_64_P097340	Zfp867	1,98E-15	0,71851833	1,00	1,54	2,04
A_64_P097385	Kcng2	5,07E-19	0,790867523	1,34	1,68	5,34
A_64_P097578		7,86E-15	0,680039396	1,03	1,37	2,45
A_64_P097598	Mtus1	3,60E-12	0,696882745	-1,12	1,10	1,26
A_64_P097754		2,22E-15	0,737680357	1,15	-1,08	2,73
A_64_P097857	Fam196a	4,24E-30	0,931176629	1,19	1,41	3,30
A_64_P097947	Pus7l	2,01E-13	0,66760148	-1,15	1,51	1,92
A_64_P097974	St3gal5	8,92E-12	0,665714717	-1,12	-1,01	1,77
A_64_P097994	Npepps	8,68E-13	0,622609707	1,11	1,07	-1,43
A_64_P098006	Fitm1	1,95E-20	0,854334418	-1,16	1,55	2,16
A_64_P098081		2,26E-29	0,933416577	1,86	3,05	5,71
A_64_P098255	Gadd45g	1,29E-29	0,928268591	1,31	2,74	5,86
A_64_P098334	Ttc22	1,82E-31	0,932339272	-1,15	3,09	14,50
A_64_P098552	Spem1	1,71E-12	0,613550388	1,03	1,15	1,45
A_64_P098622	Rpusd2	7,27E-18	0,787427263	1,07	-1,15	-1,77
A_64_P098698	Nkpd1	9,21E-20	0,857001306	-1,04	1,37	3,35
A_64_P098926	Ubap1l	2,11E-13	0,667056251	-1,21	1,44	5,26
A_64_P099032	Hist2h3c2	8,02E-20	0,786221338	1,12	2,30	4,57
A_64_P099151		1,43E-10	0,628400067	-1,11	1,19	2,49
A_64_P099307	Tcf7l2	2,70E-14	0,731699287	-1,18	-1,08	-1,36
A_64_P099583		5,01E-19	0,807301564	-1,40	1,27	4,71
A_64_P099624	Lcn12	6,87E-10	0,605412841	-1,16	1,12	1,66
A_64_P099633	Lcn10	7,95E-24	0,871410702	-1,37	1,57	4,53
A_64_P099886	Vegfa	5,37E-14	0,683061013	1,35	1,08	-1,25
A_64_P099923	Dap	7,04E-12	0,724857117	-1,08	-1,05	1,82
A_64_P099983		2,17E-13	0,709674463	1,28	-1,07	-3,91
A_64_P100008	Ptpro	1,60E-13	0,692867786	1,03	-1,00	1,35
A_64_P100158	LOC102549583	2,26E-22	0,86647366	1,60	1,61	5,86
A_64_P100208	LOC363337	2,87E-13	0,66331901	-1,14	1,01	3,39
A_64_P100279	Zdhhc8	4,12E-11	0,645674365	1,26	-1,17	-2,37
A_64_P100318	Marc1	6,91E-14	0,721989896	-1,09	1,50	2,88
A_64_P100346		2,15E-32	0,953736642	-1,07	1,26	2,89
A_64_P100447	Hoxd9	1,30E-11	0,700203942	-1,58	-1,09	3,18
A_64_P100516	Aoc3	1,89E-15	0,828412426	1,69	2,64	5,77
A_64_P100788	Ing3	8,38E-11	0,612681925	-1,15	-1,30	1,15
A_64_P100853	LOC689600	3,18E-14	0,688971986	1,09	1,46	2,98
A_64_P101150	Radil	8,76E-14	0,651788581	1,04	1,08	1,85
A_64_P101204	Slc40a1	1,19E-14	0,720914696	1,67	1,04	1,10
A_64_P101336	Rfx5	1,31E-10	0,629623715	-1,99	-1,42	-2,39
A_64_P101553	Sec63	1,15E-12	0,710069849	-1,05	-1,40	-1,89
A_64_P101618	Slc25a28	1,61E-14	0,77025553	1,53	1,02	-1,99
A_64_P101804	Efnb1	1,92E-17	0,810346874	1,39	1,91	1,92
A_64_P101908	Hmcn2	1,83E-15	0,789154827	1,24	1,31	1,48
A_64_P102141		3,68E-13	0,72260109	1,47	1,19	2,53
A_64_P102545	Tmod4	3,08E-11	0,670376935	-1,04	1,16	1,15
A_64_P102758	LOC100910585	8,58E-12	0,61945535	-1,03	-1,07	1,88
A_64_P102801	LOC108348140	1,90E-10	0,600690781	-1,75	-2,10	-2,30
A_64_P102935	Cd27	1,73E-15	0,719912585	-1,57	2,25	4,94
A_64_P103055	Pnpla3	1,32E-19	0,816517328	-1,07	1,25	3,63
A_64_P103238	Zfp52	1,27E-12	0,668395485	1,17	1,34	1,97
A_64_P103254	Klhl36	1,13E-16	0,764924524	1,24	1,03	-1,69
A_64_P103304	LOC500475	5,65E-13	0,62824922	-1,04	1,54	2,11
A_64_P104029	Spdef	5,55E-18	0,772109583	1,15	1,06	1,75
A_64_P104159	Kiaa0895l	6,45E-16	0,766920068	-1,29	1,27	2,27
A_64_P104583	Sstr2	3,92E-29	0,932011684	-1,53	2,00	9,38

A_64_P104590	Cfap157	4,69E-14	0,706453589	1,36	1,21	1,72
A_64_P104810	Podnl1	1,47E-08	0,601738529	-1,30	1,32	1,75
A_64_P104865	Lyrm1	6,66E-16	0,749048067	1,05	1,32	1,86
A_64_P105122	Smim5	1,25E-26	0,898426687	-1,56	8,42	22,12
A_64_P105152	Sall2	1,79E-14	0,695333505	1,03	1,17	2,08
A_64_P105977	LOC108349320	5,13E-23	0,862337688	-1,42	1,15	3,74
A_64_P106291	Tmem268	9,59E-18	0,767590692	1,28	1,36	2,44
A_64_P106490		1,53E-28	0,934812335	1,16	1,14	2,58
A_64_P106643		6,47E-24	0,860357227	-1,68	6,18	8,98
A_64_P106853		3,82E-12	0,630412465	-1,13	1,89	3,93
A_64_P106893		7,40E-14	0,679378117	1,14	1,13	1,78
A_64_P107185		3,91E-12	0,654304638	-1,36	1,34	8,84
A_64_P107851	Smarcd3	8,31E-14	0,700189869	-1,07	-1,07	1,66
A_64_P108009	Nrarp	9,58E-24	0,891070792	1,85	1,98	1,93
A_64_P108245		8,94E-12	0,618891997	1,08	2,01	3,40
A_64_P108379	Cops7b	3,08E-11	0,649554405	1,23	1,69	1,32
A_64_P108424		5,35E-22	0,836424472	-1,39	1,89	4,32
A_64_P108454	Spata5l1	1,84E-13	0,691291115	1,07	-1,09	-2,64
A_64_P108459		2,00E-22	0,904583474	-1,11	1,32	3,79
A_64_P108876	Dusp13	8,64E-15	0,703228409	-1,33	1,58	3,36
A_64_P108881	Pde1c	1,73E-14	0,717037101	-1,14	1,15	4,10
A_64_P108948	Atxn2	3,43E-12	0,63182576	1,09	-1,20	-1,73
A_64_P109342		2,45E-09	0,608509429	-1,46	-2,06	2,73
A_64_P109531	Tgfb2	5,21E-10	0,652234652	1,91	1,23	-1,44
A_64_P109657	Ednra	4,94E-17	0,732172895	-1,37	-1,83	-6,30
A_64_P109899	Lgals4	3,02E-15	0,714232196	-1,13	1,99	7,15
A_64_P109900	Lgals4	3,67E-23	0,875231444	-1,70	3,79	11,46
A_64_P109938	Gnb1l	7,75E-13	0,69533923	-1,22	-1,13	2,18
A_64_P110389	Fibcd1	1,08E-16	0,746514164	-1,08	1,35	3,25
A_64_P110394	Fibcd1	3,03E-13	0,706002089	-1,06	1,01	3,42
A_64_P110469	Fdxr	1,93E-12	0,704143658	1,30	1,21	1,47
A_64_P110544	Spdef	6,20E-14	0,801090847	1,06	1,05	1,91
A_64_P110574		6,11E-12	0,624084617	1,04	1,65	1,63
A_64_P110599	Plekhf1	1,52E-23	0,906428279	1,07	-1,62	-1,43
A_64_P110898		2,67E-11	0,672212587	-2,50	-1,29	2,87
A_64_P111099	Trpc7	2,10E-16	0,776539262	-1,63	1,17	3,48
A_64_P111345	Gmps	2,32E-20	0,795320016	-1,06	-1,37	-2,74
A_64_P111500	Slc1a5	1,39E-11	0,63769923	-1,17	1,10	1,42
A_64_P111599	Bcor	4,44E-15	0,766167124	1,15	-1,08	-1,64
A_64_P111649	Crtc1	1,05E-17	0,746369769	-1,19	1,00	1,65
A_64_P111674	Olr206	7,09E-17	0,785491154	1,25	2,72	5,57
A_64_P111776	Rab4a	8,89E-16	0,764092801	1,01	-1,08	-1,30
A_64_P111791	Abhd18	3,72E-12	0,696497401	1,58	1,28	1,11
A_64_P111903	Mmp17	2,83E-12	0,679987176	-1,06	-1,03	1,05
A_64_P112141	Ptpn13	5,27E-13	0,629153163	1,29	1,49	1,24
A_64_P112181	RGD1562660	8,83E-32	0,946043771	1,10	1,30	3,14
A_64_P112185		4,59E-18	0,833368359	-1,35	1,27	4,95
A_64_P112295	Slc1a7	5,23E-10	0,609512993	-1,59	-1,08	2,69
A_64_P112406	Chac1	9,83E-15	0,758882557	1,02	-1,23	1,10
A_64_P112767	Ralbp1	4,19E-13	0,702346666	1,01	-1,26	-1,27
A_64_P112816	gen-01	2,01E-21	0,842567171	1,13	1,81	3,60
A_64_P112909	Yjefn3	2,17E-12	0,610300985	-1,74	1,58	2,48
A_64_P113458		2,99E-14	0,711309154	-1,02	1,35	3,87
A_64_P113645	RGD1561551	2,12E-13	0,666978863	-1,03	1,07	2,12
A_64_P113692	Myo7b	8,01E-27	0,878567388	-1,32	1,55	7,30
A_64_P113695	Myo7a	5,19E-20	0,822668027	-1,09	1,98	3,92
A_64_P114263	RGD1564786	3,25E-16	0,736247758	1,02	1,20	3,10
A_64_P114308	Heyl	3,17E-12	0,605047208	-1,16	-1,01	2,29
A_64_P114338	RGD1563263	2,77E-15	0,753780544	-1,09	3,29	1,33
A_64_P114396		7,09E-24	0,909206562	-1,51	1,68	9,15
A_64_P114495	LOC363324	1,90E-19	0,828238741	1,06	-1,01	2,15
A_64_P114838		4,96E-30	0,942910665	1,50	1,50	4,74
A_64_P114960	Kcnk4	3,48E-12	0,655810165	-1,27	2,15	2,03
A_64_P115222	Pitpnm2	6,83E-17	0,827608605	1,16	-1,04	-1,67
A_64_P115417	Ntrk1	6,73E-14	0,702522566	-2,01	-1,04	7,92
A_64_P115566	Acer2	3,72E-17	0,756001567	1,33	2,02	4,03
A_64_P115631	Lime1	5,92E-13	0,627647398	1,03	-1,08	-1,91
A_64_P115786	LOC299312	5,26E-11	0,619328791	1,04	-1,08	-1,23
A_64_P116171	Ak8	7,14E-18	0,842528857	1,05	-1,25	3,12
A_64_P116298		2,68E-33	0,952736211	1,22	1,41	5,19
A_64_P116299	LOC102557206	5,14E-11	0,663710321	1,04	-1,03	1,74

A_64_P116301	Adam33	1,18E-09	0,640548093	-1,13	1,27	-2,39
A_64_P116501	Kcnf1	2,22E-12	0,637599384	1,33	1,37	2,58
A_64_P116537	Nphp4	1,14E-21	0,81583311	1,15	1,07	1,73
A_64_P116600	Rcan1	1,94E-22	0,896461393	1,35	1,14	1,01
A_64_P116606	Rcan1	1,44E-10	0,628291338	1,36	1,27	1,51
A_64_P116972	Crh	1,26E-10	0,606749063	1,95	1,20	-1,37
A_64_P117126	Slc25a19	1,12E-17	0,814220259	1,06	-1,14	-1,55
A_64_P117173	Wfdc10	4,62E-13	0,701258718	-1,29	-1,08	1,97
A_64_P117233		1,30E-13	0,646944757	-1,15	1,06	1,67
A_64_P117468	Ky	1,58E-17	0,763391219	-1,05	1,91	7,02
A_64_P117726	Slu7	2,40E-08	0,614446395	1,04	-1,12	-1,02
A_64_P117901	Trib3	3,59E-20	0,838620102	1,14	1,83	1,56
A_64_P118308		8,53E-20	0,819406287	-1,14	2,03	4,13
A_64_P118392	Nlrx1	2,01E-12	0,611338814	1,00	1,48	3,12
A_64_P118427		8,16E-13	0,69474442	1,23	3,32	1,22
A_64_P118457	Cited4	3,15E-28	0,926420908	1,07	1,04	-1,87
A_64_P118499	RGD1561916	4,32E-18	0,754122143	1,11	1,65	2,28
A_64_P118526		8,20E-11	0,636228764	-1,27	-3,15	-1,42
A_64_P118602	Rtel1	2,32E-10	0,621455218	1,04	1,17	-1,74
A_64_P118628	Bdkrb1	4,13E-15	0,711003738	1,03	-1,23	1,78
A_64_P118698		1,93E-16	0,741135987	-1,54	1,36	3,72
A_64_P119022	Bsnd	3,68E-32	0,929210749	-1,28	6,67	14,77
A_64_P119169	LOC684270	3,53E-13	0,634330245	-1,15	-1,07	-1,32
A_64_P119369	Baat	1,57E-21	0,856430231	-1,07	1,46	4,39
A_64_P119680	Alkal2	1,37E-12	0,688718302	1,71	1,20	-1,51
A_64_P119777	Pxdn	1,24E-10	0,607005122	-1,03	1,16	1,16
A_64_P119821	RGD1311084	1,97E-24	0,927518576	-1,38	1,38	3,16
A_64_P120036		2,02E-12	0,684042969	-1,60	-1,84	-2,92
A_64_P120050	Epc1	1,11E-12	0,64650284	-1,56	-1,56	-2,01
A_64_P120065	Jade1	5,33E-19	0,790478938	1,35	1,25	1,18
A_64_P120070	Insl6	1,41E-21	0,857021007	-1,36	1,87	5,50
A_64_P120445	Cacng6	8,37E-21	0,869776082	-1,12	-1,18	-1,61
A_64_P120564	Zfp90	9,16E-12	0,665378924	-1,19	1,82	1,81
A_64_P120634	Pgf	1,56E-23	0,879156384	1,29	1,78	1,86
A_64_P120640	Pgf	1,14E-21	0,85813963	1,07	1,64	2,71
A_64_P120679	Cck	1,62E-15	0,720567762	1,58	1,26	1,81
A_64_P120861	Efna1	5,07E-13	0,679478673	1,11	1,80	2,51
A_64_P120866	Bnipl	3,84E-22	0,863791436	1,47	5,05	5,35
A_64_P120881	Hils1	1,22E-15	0,743352137	-1,20	1,21	1,33
A_64_P120882	Hils1	4,50E-15	0,730766368	-1,00	1,45	2,04
A_64_P120931	Asb3	6,87E-12	0,647034051	-1,20	-1,25	-1,49
A_64_P121051	Myo15b	1,14E-18	0,78468393	-1,31	1,21	3,08
A_64_P121136		3,02E-23	0,876128317	2,96	6,32	7,04
A_64_P121318	Leng8	8,22E-13	0,694657961	1,23	1,85	1,49
A_64_P121796	Perm1	2,01E-34	0,952461101	-1,02	-1,07	-4,66
A_64_P122238	Tuba4a	2,92E-13	0,7064118	-1,13	-1,19	-1,64
A_64_P122247	Hcrtr1	2,60E-17	0,793421552	1,14	1,39	3,92
A_64_P122624	Sphkap	9,93E-29	0,935890037	1,23	1,34	-2,03
A_64_P122671	Avpi1	1,19E-15	0,723659929	1,01	1,15	1,75
A_64_P122676	Crygs	1,51E-16	0,762384232	-1,48	6,01	7,54
A_64_P122790	Acss1	5,72E-15	0,746949636	-1,05	1,08	-1,19
A_64_P122944	Phf20l1	2,29E-19	0,827009308	1,15	1,31	1,51
A_64_P122975		1,14E-13	0,716682191	-1,08	1,35	1,37
A_64_P123118	Fosl1	6,11E-20	0,847840396	1,12	-1,19	1,22
A_64_P123243		6,93E-14	0,654637189	1,08	1,41	2,20
A_64_P123792		5,96E-12	0,62443539	1,07	1,24	2,42
A_64_P123949	Gk	2,03E-25	0,905927222	-1,06	-1,08	-2,04
A_64_P124090	Ntsr1	2,03E-29	0,954842786	1,03	1,37	8,97
A_64_P124126	LOC100910970	2,16E-15	0,717652832	1,11	1,43	2,21
A_64_P124210	Greb1l	1,47E-15	0,74161451	-1,01	1,51	2,35
A_64_P124567	Shc2	1,01E-22	0,830894117	1,07	1,27	3,79
A_64_P124898	LOC363337	3,79E-18	0,821807074	-1,08	-1,10	3,30
A_64_P125305	B9d2	1,89E-11	0,633518103	1,19	2,85	5,22
A_64_P125350	Kcnh6	9,43E-27	0,916284175	1,14	1,56	3,59
A_64_P125611	Tbx6	4,75E-20	0,849302168	1,04	-1,19	1,44
A_64_P125715	Serinc4	7,89E-10	0,603312399	-1,53	1,90	3,94
A_64_P125858	Socs5	1,39E-11	0,680494975	1,13	1,14	-1,19
A_64_P125883	Socs3	2,18E-21	0,827967343	1,42	1,98	1,49
A_64_P125938	Snx27	1,14E-12	0,618966051	1,09	1,01	-1,92
A_64_P125978	Atp2a1	4,28E-16	0,753098095	-1,37	1,26	1,97
A_64_P126030	Slc22a7	8,44E-14	0,784795012	1,16	2,03	1,46

A_64_P126135	Rasl10b	3,04E-11	0,601689593	1,03	1,02	-1,97
A_64_P126355	Crispld2	6,35E-20	0,78796176	1,59	1,32	2,74
A_64_P126450	LOC100909409	1,73E-11	0,634762854	-1,60	1,11	2,45
A_64_P126493	Crem	1,60E-15	0,720665008	1,06	-1,56	-2,49
A_64_P126630	Gsc	4,40E-20	0,808417363	-1,38	1,16	4,60
A_64_P126818	Tspan18	3,91E-21	0,838683327	1,05	1,38	2,90
A_64_P126955	Rybp	1,11E-12	0,710398095	-1,05	-1,16	1,80
A_64_P127223	Mocs3	1,48E-20	0,815762267	1,00	1,08	1,77
A_64_P127253	Abracl	5,19E-16	0,731758629	-1,08	-1,03	-1,87
A_64_P127327	Sys1	2,71E-14	0,749232571	1,17	-1,07	-1,58
A_64_P127332		9,10E-13	0,693480629	-1,03	1,32	2,20
A_64_P127533		2,46E-17	0,759583669	1,54	2,41	5,63
A_64_P127833	Ppfibp1	4,23E-11	0,666281799	1,12	-1,20	-1,50
A_64_P128225	Klhl40	5,96E-13	0,654361515	-1,21	1,26	1,24
A_64_P128438	Irf1	1,43E-14	0,697757791	1,03	1,69	2,22
A_64_P128454	Nrf1	1,03E-11	0,641629245	1,27	-1,04	-2,73
A_64_P128725	Mthfd2	3,11E-13	0,685214172	1,10	-1,46	-3,18
A_64_P128833	Wnt3	1,59E-11	0,61089062	1,12	1,63	1,55
A_64_P128874	MGC116197	4,47E-12	0,674387588	1,08	1,28	1,32
A_64_P129004	Kcnj12	1,99E-19	0,863527901	1,08	1,25	5,66
A_64_P129273	Hrasls	4,42E-15	0,686429766	-1,17	1,11	2,15
A_64_P129563	Arid5a	9,33E-12	0,618312126	1,24	1,36	2,81
A_64_P129730	Tmem79	1,51E-18	0,84048715	1,17	3,13	5,68
A_64_P129855	Meis1	1,02E-12	0,647577708	-1,12	-1,14	-1,61
A_64_P129951	Nup205	3,78E-20	0,824712792	1,14	-1,14	-2,82
A_64_P130025	Dll1	7,51E-14	0,721109771	-1,18	2,42	3,46
A_64_P130201	Trpv1	9,05E-13	0,693549958	-1,12	1,19	3,53
A_64_P130239	Slitrk3	1,40E-09	0,617053559	1,33	1,45	1,21
A_64_P130280	Upk1a	2,35E-21	0,811131983	-1,02	1,31	2,03
A_64_P130294	Hfm1	2,08E-15	0,694624488	1,65	1,58	2,54
A_64_P130324	Lcn5	1,56E-20	0,830318702	-1,19	1,15	2,07
A_64_P130380		1,67E-18	0,827311127	-1,18	-1,25	-1,93
A_64_P130390	LOC681364	2,99E-19	0,83831524	1,16	1,68	2,44
A_64_P130551	Ptchd3	3,48E-14	0,709656844	-1,31	3,01	4,63
A_64_P130568	Hist1h2bcl1	2,04E-13	0,690074536	-1,07	1,00	1,29
A_64_P130873	Csrnp3	1,75E-19	0,814576283	1,07	1,45	2,68
A_64_P131051	Fos	7,66E-33	0,945574198	1,23	-2,72	-12,49
A_64_P131086	Rab30	1,15E-15	0,743978339	1,09	1,37	2,42
A_64_P131122	Iqank1	8,39E-16	0,780716544	-1,31	1,15	3,72
A_64_P131141		9,80E-16	0,763220363	-1,08	1,12	2,48
A_64_P131151	Opn4	3,95E-15	0,767227623	-1,06	1,62	2,74
A_64_P131370	Calr4	2,56E-16	0,738503782	-1,33	1,49	3,58
A_64_P131409	Pdgfa	2,56E-11	0,629386286	-1,08	1,03	1,22
A_64_P131451	LOC102550991	1,17E-30	0,940488869	1,45	1,46	4,27
A_64_P131477	Twist2	2,80E-11	0,650862426	-1,20	-1,04	1,91
A_64_P131697	Sema3f	4,43E-17	0,754453753	1,01	1,25	3,28
A_64_P131736	Fas	2,99E-14	0,71130191	-1,17	1,46	1,37
A_64_P131791	Shroom3	5,20E-20	0,848778653	1,05	-1,13	-1,99
A_64_P131834		1,16E-14	0,721219589	-1,10	1,12	2,30
A_64_P131869	Cntrob	6,63E-16	0,729391945	-1,13	1,31	2,82
A_64_P132044	Spocd1	8,96E-13	0,693660956	1,17	1,39	2,67
A_64_P132199	B3galt5	2,48E-24	0,887166797	-1,50	1,63	5,02
A_64_P132327	Dnase1	1,41E-12	0,707750799	-1,18	1,06	1,81
A_64_P132716	Oacyl	2,00E-25	0,897271772	1,13	3,78	11,56
A_64_P132721	Oacyl	5,89E-29	0,916464263	1,32	1,57	12,58
A_64_P132976	Gprc5c	8,28E-18	0,768806315	-1,03	1,61	5,48
A_64_P132981	Gprc5c	8,98E-34	0,938009655	-1,02	1,53	12,57
A_64_P133096	Neurl2	1,72E-12	0,740274718	-1,38	1,09	1,33
A_64_P133152	Neurod6	2,01E-10	0,684186644	-1,12	-1,44	-1,65
A_64_P133167	Duoxa2	1,04E-15	0,793788071	-1,34	2,85	2,44
A_64_P133276	Dact2	4,41E-22	0,837557844	-1,25	1,12	3,17
A_64_P133430	Mat2b	1,88E-10	0,6008753	1,20	-1,08	-2,00
A_64_P133479	Vezt	2,01E-12	0,611359064	-1,04	-1,19	-1,41
A_64_P133502		8,39E-15	0,703536581	-1,15	2,24	5,08
A_64_P133518	Card10	2,45E-15	0,716388875	-1,46	1,23	7,31
A_64_P134150	Tex26	1,38E-17	0,764541052	1,61	2,05	5,26
A_64_P134263		9,38E-15	0,759315033	1,00	1,31	2,74
A_64_P134426		2,02E-25	0,897229107	1,82	1,48	5,94
A_64_P134445	Tmem179	9,07E-13	0,67252279	1,27	1,55	2,45
A_64_P134460	Jsrp1	6,06E-18	0,804419086	-1,49	1,69	2,75
A_64_P134699	Rft1	2,09E-15	0,717989046	-1,00	1,40	1,96

A_64_P134744	Hpn	2,13E-18	0,825690953	-1,27	1,53	5,18
A_64_P134748	Hpn	2,66E-27	0,904008157	1,01	3,02	13,14
A_64_P135152	Msantd1	1,40E-19	0,830185087	1,21	1,47	2,49
A_64_P135229	Nrbf2	6,24E-12	0,623803476	1,18	-1,44	-2,48
A_64_P135474	Cxcl12	3,88E-24	0,862885822	-1,38	1,38	4,91
A_64_P135502		1,15E-14	0,740198696	-1,00	1,13	3,34
A_64_P135843	Grb7	3,28E-30	0,931828003	1,11	1,95	14,63
A_64_P136178	LOC108349417	5,25E-23	0,86221938	-1,04	3,85	9,49
A_64_P136367	Cbx8	1,49E-09	0,637114942	1,04	1,19	-1,27
A_64_P136382	Acvr1	1,59E-10	0,603323589	-1,08	-1,25	-1,81
A_64_P136432	Armcx6	1,42E-17	0,782164468	-1,02	-1,05	-1,62
A_64_P136512	Ecel1	5,39E-15	0,764419578	-1,33	-1,25	2,29
A_64_P136676		1,14E-23	0,880549589	-1,03	1,20	2,85
A_64_P136751	Rgs6	6,42E-10	0,628660836	1,37	1,48	1,64
A_64_P137070	Zfp318	1,81E-12	0,664069349	1,09	-1,01	-1,63
A_64_P137075	Col7a1	6,20E-18	0,788666211	-1,07	1,15	1,84
A_64_P137189	Zpbp2	1,19E-11	0,6148858	-1,30	1,64	2,15
A_64_P137262	Fam163a	1,26E-12	0,66854866	-1,66	-1,08	-1,01
A_64_P137320	Ccdc113	1,75E-25	0,877296126	1,24	2,04	2,54
A_64_P137436	LOC688286	3,24E-26	0,904007594	-1,41	2,08	6,75
A_64_P137461	Bspry	1,12E-12	0,744739134	-1,83	-1,05	1,11
A_64_P137784	Plvap	3,84E-27	0,911334354	1,01	1,44	4,38
A_64_P138011	Adra1b	3,63E-21	0,851869344	1,15	-1,07	2,22
A_64_P138086	Pcdh20	2,28E-11	0,605775487	-1,02	1,10	-1,71
A_64_P138091		4,25E-14	0,660511063	1,14	1,01	-2,39
A_64_P138096	RGD1562683	8,45E-17	0,727077976	-1,37	1,45	8,24
A_64_P138127	RGD1562660	3,49E-24	0,87521761	1,23	1,55	3,52
A_64_P138216	Gch1	4,02E-14	0,761802708	1,36	-1,03	-1,38
A_64_P138291	Cenpk	2,86E-22	0,876027387	1,42	4,73	5,08
A_64_P138415	Cldn19	6,33E-17	0,786406354	1,18	-1,06	1,23
A_64_P138430	Odf3b	1,14E-19	0,855787011	1,20	1,31	1,84
A_64_P138565	Narf	4,61E-18	0,773612625	1,04	-1,10	-1,52
A_64_P138575		1,95E-13	0,746419185	-1,54	1,53	2,55
A_64_P138636	Nr2f1	2,25E-18	0,779356671	1,21	-1,17	-2,57
A_64_P138800	Myadml2	9,87E-28	0,915705662	1,25	1,13	1,05
A_64_P139250	Tmprss9	7,87E-32	0,934374645	-1,08	1,92	10,14
A_64_P139457	Zfp14	8,26E-13	0,67365378	-1,02	1,73	1,83
A_64_P139485	Plcd4	1,43E-08	0,60215902	1,17	1,20	2,12
A_64_P139559		4,98E-14	0,705805099	-1,14	2,53	4,08
A_64_P139802	Prph	2,06E-11	0,745081454	-1,37	1,13	1,54
A_64_P140000	Myo15b	2,11E-25	0,921015326	-1,28	1,41	3,28
A_64_P140025	Tyrp1	6,08E-12	0,690618941	-1,07	-1,28	-1,47
A_64_P140225		7,50E-25	0,892077596	1,21	1,30	4,02
A_64_P140226		2,29E-18	0,779223494	2,08	2,54	5,74
A_64_P140318	LOC108349340	1,57E-14	0,718069007	1,04	-1,04	1,50
A_64_P140323	LOC108349386	3,69E-24	0,885477122	1,65	1,79	4,75
A_64_P140437	Cxcl2	6,68E-20	0,805529984	2,45	1,34	1,27
A_64_P140652	Ccdc84	1,18E-08	0,605298499	1,24	1,17	-1,51
A_64_P141727	Rhob	2,32E-15	0,716935684	1,28	1,33	5,15
A_64_P141737	RGD1566325	5,85E-13	0,677778004	1,66	2,32	3,31
A_64_P141962	LOC501297	1,79E-24	0,878224133	-1,36	-1,03	4,44
A_64_P142046	Nudt15	4,84E-13	0,656944882	-1,13	1,17	2,59
A_64_P142141	Cldn23	3,51E-13	0,704359338	1,61	1,47	1,57
A_64_P142266	Tex38	1,25E-13	0,695625058	1,27	2,27	3,85
A_64_P142320	Zfp458	3,47E-14	0,746842803	1,27	1,82	2,02
A_64_P142625	Gprc5a	4,95E-24	0,873622214	1,48	1,11	1,03
A_64_P142774	Fgf5	4,23E-21	0,82383978	1,14	2,26	8,36
A_64_P142776	Fgf5	2,38E-12	0,660619667	-1,23	1,57	3,88
A_64_P142804	Nudt13	1,96E-25	0,887685128	1,02	2,09	4,58
A_64_P142809	Sipa1l3	3,50E-15	0,751577731	-1,15	-1,61	1,40
A_64_P142937	Cabp2	1,01E-16	0,747120738	-1,15	1,33	2,36
A_64_P142942	Scx	4,16E-29	0,925074452	-1,04	1,25	4,58
A_64_P142988	Tmprss9	1,99E-29	0,954877387	1,19	2,47	8,54
A_64_P143072	Aanat	1,14E-18	0,784681857	-1,42	-1,06	2,47
A_64_P143186	LOC108353713	5,70E-18	0,804874123	1,41	3,89	5,49
A_64_P143480		8,47E-27	0,916625045	2,01	3,14	7,04
A_64_P143557	Phc1	2,21E-12	0,637611504	1,00	-1,02	-1,49
A_64_P143675	Zfp518a	1,37E-23	0,856552147	-1,14	1,98	2,80
A_64_P143810	Vom2r46	1,94E-18	0,780529526	-1,60	1,89	2,15
A_64_P144053	Dyrk2	8,00E-17	0,727604046	1,32	-1,26	-1,68
A_64_P144123	Olig2	3,03E-24	0,886315176	-1,13	-1,19	-1,46

A_64_P144134	RGD1566029	3,76E-16	0,801912623	-1,07	-1,06	3,35
A_64_P144153	Klhl35	5,52E-20	0,789002161	1,18	2,18	3,76
A_64_P144243		3,47E-12	0,655847035	1,29	2,91	2,89
A_64_P144253	Shank2	4,60E-11	0,621224284	1,32	1,21	-1,35
A_64_P144968	Calb2	7,22E-18	0,787487676	-1,44	1,18	2,63
A_64_P145058	Dmrta2	1,96E-19	0,779391258	1,38	1,39	2,68
A_64_P145433	Ahr	1,56E-08	0,621429522	1,32	1,04	-1,17
A_64_P145696	N6amt1	8,04E-16	0,704632528	-1,12	1,14	1,65
A_64_P145828	LOC691712	5,34E-33	0,946301519	1,12	1,32	4,10
A_64_P145829	LOC685324	2,57E-28	0,926989822	1,17	1,19	4,01
A_64_P146135		8,22E-12	0,620047028	-1,06	1,63	2,04
A_64_P146243	Pou2f3	1,88E-21	0,855477914	-1,32	3,46	7,60
A_64_P146298	Rab20	1,73E-11	0,60972225	1,09	-1,57	-2,63
A_64_P146692	Slc22a1	7,14E-24	0,859864289	1,02	2,62	6,12
A_64_P146964	Unkl	1,01E-15	0,725226062	1,09	1,16	1,17
A_64_P146970	Cacng2	3,49E-17	0,805955506	-1,09	-1,07	1,35
A_64_P147094	Sept8	1,47E-21	0,830391635	1,00	1,41	2,39
A_64_P147190	Fdps	3,24E-11	0,648875176	-1,24	-1,02	-1,28
A_64_P147403	LOC100911864	1,20E-11	0,661954829	1,05	1,17	1,25
A_64_P147438	Slc35f4	1,38E-17	0,764507535	1,20	-1,07	-2,29
A_64_P147898		2,48E-22	0,84086263	-1,12	-1,06	1,43
A_64_P147913	Optn	3,55E-14	0,687735859	1,20	1,25	1,42
A_64_P148042	Mex3b	1,90E-22	0,87794395	1,06	-3,19	-2,05
A_64_P148265		6,62E-15	0,726892774	-1,02	1,80	2,71
A_64_P148325		3,14E-10	0,638971203	-1,23	1,69	1,30
A_64_P148330		3,75E-13	0,68303729	-1,53	-1,33	1,12
A_64_P148508	Prr7	7,09E-14	0,756373858	1,32	1,36	1,08
A_64_P148535	Napepld	9,86E-13	0,746091641	-1,19	1,71	2,50
A_64_P148613	Elk4	2,61E-16	0,716083835	1,16	-1,43	-2,26
A_64_P149119	Rftn1	4,66E-13	0,657405591	1,15	-1,04	1,79
A_64_P149280	Vegfb	1,34E-18	0,783422439	1,12	-1,12	-1,51
A_64_P149300	Cacnb4	1,56E-22	0,878848619	1,22	1,14	-1,92
A_64_P149421		1,64E-25	0,877571607	1,20	-2,29	-8,49
A_64_P149461	Cdc42ep1	3,02E-11	0,627093722	-1,32	-1,41	1,94
A_64_P149482	Mrgprf	2,63E-32	0,922032717	-1,43	3,29	27,85
A_64_P149689	RGD1305733	3,60E-09	0,623888327	1,10	1,11	-1,11
A_64_P149735	Sqstm1	1,31E-15	0,76065426	1,02	-1,03	-1,63
A_64_P150181	Rab23	5,98E-20	0,821742577	1,15	-1,03	-1,70
A_64_P150251	Epn3	1,16E-13	0,648358739	-1,02	1,10	2,59
A_64_P150256	Epn3	9,01E-17	0,748121506	1,28	1,57	3,33
A_64_P150326	LOC108348357	3,54E-20	0,825132216	-1,00	1,92	4,21
A_64_P150338	Tmem132c	1,85E-13	0,711452001	1,01	-1,18	1,46
A_64_P150361	Pdzd3	4,41E-12	0,600469804	-1,94	1,84	3,33
A_64_P150397	Tex38	1,66E-15	0,740463961	1,06	2,20	5,40
A_64_P150466	Vstm2l	2,81E-14	0,711959628	1,22	1,35	1,80
A_64_P150509	Ccr10	1,88E-17	0,795935719	-1,25	1,55	3,10
A_64_P150691	Araf	5,14E-15	0,72944113	-1,01	1,01	-1,36
A_64_P150726	Ahsp	5,40E-11	0,618951568	1,36	1,55	2,01
A_64_P150814	Rnf19a	4,09E-15	0,78236148	1,05	-1,29	-1,50
A_64_P150876	Cst6	8,20E-11	0,657499619	1,03	1,15	1,47
A_64_P151071	Hmgcs1	2,67E-19	0,811691073	-1,19	-1,28	-1,95
A_64_P151393	Mpp4	1,02E-13	0,697923809	1,65	1,66	2,22
A_64_P151448	Fancf	5,00E-12	0,651146353	1,12	1,54	1,60
A_64_P151627	Trim16	8,64E-20	0,845817465	1,04	1,24	2,47
A_64_P152214	Rtn4r	8,01E-11	0,613336054	-1,22	1,01	2,50
A_64_P152252	Fscn2	1,31E-26	0,907196407	1,15	1,60	10,44
A_64_P152800		3,02E-23	0,886203979	-1,17	1,01	3,24
A_64_P152878	Shisa8	2,72E-19	0,795478276	1,02	1,37	3,18
A_64_P152888	Nphp4	2,89E-19	0,795047191	1,14	1,03	1,89
A_64_P152919	Gprin3	1,67E-10	0,626189694	1,15	1,34	6,96
A_64_P153002	Gpr20	2,19E-26	0,913567671	-1,33	1,57	5,77
A_64_P153062	Tspan9	1,20E-23	0,890135748	-1,33	1,08	5,44
A_64_P153091	Sel1l3	9,96E-13	0,671383127	1,21	1,26	1,82
A_64_P153105	RGD1562890	1,85E-15	0,719199589	1,02	1,85	2,83
A_64_P153149	Kcnab1	1,38E-14	0,719399649	-1,11	-1,09	-1,36
A_64_P153282	Fpr2l	7,24E-17	0,800438915	-1,10	1,33	6,06
A_64_P153301	Ccdc160	7,88E-13	0,623881236	1,09	1,81	1,93
A_64_P153363	Dmrtc1b	1,45E-17	0,797892114	1,01	1,12	2,14
A_64_P153533	Ubtd1	1,60E-14	0,717882746	1,19	1,14	1,37
A_64_P153596	Adam4l1	2,29E-12	0,637181711	1,44	1,44	3,19
A_64_P153694	LOC108348293	2,33E-18	0,837751856	1,11	1,28	2,75

A_64_P154034	Hapln4	2,62E-18	0,795238622	2,36	3,40	2,22
A_64_P154039	Hapln4	1,35E-18	0,763963556	1,32	2,28	3,58
A_64_P154082	Tp53bp2	4,29E-10	0,634505697	-1,11	-1,09	1,38
A_64_P154292	Hnrnpa3	6,49E-10	0,606275926	-1,01	-1,09	-1,44
A_64_P154430		1,45E-11	0,698898255	-1,19	1,01	1,89
A_64_P154469	Ccdc166	1,12E-25	0,908045082	1,71	9,85	6,31
A_64_P154525	Ypel4	3,83E-16	0,771458212	-1,09	1,01	1,39
A_64_P154952	Nr4a2	8,01E-15	0,760781268	1,17	-4,69	-4,19
A_64_P155059	Slc22a12	1,31E-13	0,672747853	-1,50	1,63	1,85
A_64_P155201	LOC690126	1,79E-26	0,906101412	2,27	4,20	5,81
A_64_P155265		4,19E-30	0,916148794	-1,68	5,71	23,88
A_64_P155311	Prss22	1,40E-20	0,844200963	-1,07	1,66	6,93
A_64_P155393	Ccnd1	1,78E-12	0,685550633	-1,06	1,24	1,38
A_64_P155463		5,43E-27	0,91017949	-1,04	1,05	3,23
A_64_P155795	Lpo	3,59E-12	0,655394585	-1,51	1,21	1,69
A_64_P155895	Thnsl2	8,30E-11	0,612819707	1,24	1,27	1,71
A_64_P155983	Vegfa	8,19E-12	0,723151336	2,04	-1,17	1,26
A_64_P156263	Lmcd1	9,71E-17	0,782946717	1,03	1,08	1,51
A_64_P156303	Cyb5d2	1,34E-19	0,830438436	-1,05	1,19	2,95
A_64_P156632	Itpr1	5,85E-10	0,630012598	-1,01	-1,05	-1,65
A_64_P156662		1,25E-18	0,815687745	-1,02	1,51	2,37
A_64_P156762	Gpr156	1,80E-11	0,634168753	-1,51	1,04	2,83
A_64_P156791	Hagh	2,54E-15	0,736351925	-1,16	-1,06	-1,23
A_64_P157029	Usp10	2,81E-14	0,690356836	1,09	-1,12	-1,83
A_64_P157044	Cbx2	3,75E-21	0,85168597	1,04	2,41	-1,13
A_64_P157131	Pkig	1,12E-10	0,608499321	-1,00	1,01	-1,44
A_64_P157239		1,12E-19	0,831608708	1,15	1,24	-1,45
A_64_P157244	Sfxn2	1,56E-15	0,759017729	1,27	1,31	-1,22
A_64_P157296	Efcab8	7,14E-16	0,78207109	-1,69	-1,01	1,82
A_64_P157313	Grhpr	9,89E-13	0,62087875	-1,20	1,09	1,65
A_64_P157385	Dus4l	2,01E-17	0,779341167	-1,44	1,84	2,55
A_64_P157405	Reep1	3,61E-16	0,754622949	1,04	1,07	-1,38
A_64_P157490	Luc7l3	1,12E-15	0,74417388	-1,19	-1,25	-3,58
A_64_P157604	Cpne7	6,25E-11	0,639984224	1,29	1,18	-1,20
A_64_P157728	Zcchc9	1,94E-11	0,633181477	1,17	1,12	-1,60
A_64_P157743	Ccl20	1,72E-29	0,911804023	1,04	-1,01	1,15
A_64_P157873	Nhlh2	1,54E-11	0,611349625	-1,30	1,40	4,28
A_64_P158103	LOC103690708	2,91E-12	0,65807304	-1,09	-1,20	2,02
A_64_P158441	Rfx2	1,32E-15	0,742644587	1,44	1,38	1,35
A_64_P158504	Klhl2	5,76E-11	0,641096151	1,24	1,51	-1,13
A_64_P158778	Ocm2	3,67E-10	0,636759568	-2,39	1,09	1,82
A_64_P158883	Mxd1	5,60E-10	0,608500245	1,36	1,41	1,87
A_64_P158903	Has2	1,15E-16	0,836111691	2,30	1,29	1,44
A_64_P158945	Tcap	1,09E-22	0,880493391	1,16	-1,37	-1,49
A_64_P159175	Adamts6	2,22E-16	0,739811439	-1,20	1,11	2,15
A_64_P159275	Inip	3,75E-17	0,805413312	1,11	1,05	-1,75
A_64_P159543	Gem	4,03E-12	0,653930385	1,38	-1,71	-1,54
A_64_P159608	Chml	7,70E-11	0,613902173	2,16	1,47	-1,91
A_64_P159858	Chchd6	5,03E-17	0,771777647	-1,17	1,07	1,37
A_64_P160008	Nxnl2	1,90E-14	0,768733953	1,17	1,31	-1,89
A_64_P160072	RGD1309651	1,06E-14	0,802504832	1,41	-1,17	1,30
A_64_P160091	Gpr62	5,39E-22	0,872988534	1,38	2,08	4,15
A_64_P160096	Mei1	1,19E-13	0,673889358	-1,14	1,37	3,35
A_64_P160373		1,46E-15	0,759663231	1,20	1,48	6,73
A_64_P160393	Tcf4	2,81E-10	0,618713208	1,50	1,17	-1,25
A_64_P161057	Actr3b	2,35E-13	0,688480381	1,22	-1,07	-3,12
A_64_P161185	Odf2l	5,52E-14	0,657390738	1,14	1,37	1,90
A_64_P161265	Zfand2b	5,04E-16	0,825836404	-1,23	-1,16	-1,67
A_64_P161354		2,52E-11	0,604353856	-1,85	-1,98	-1,62
A_64_P161630	Amigo3	3,50E-22	0,852322588	1,15	1,39	2,71
A_64_P161635	RGD1310507	8,06E-21	0,819723295	1,05	1,77	6,15
A_64_P161833	Snai3	6,66E-18	0,788112654	1,40	1,98	2,28
A_64_P162002		2,20E-09	0,610182662	4,99	2,11	1,67
A_64_P162047	Ccm2l	1,97E-31	0,924831481	1,20	2,03	5,15
A_64_P162125		1,24E-23	0,880176116	-1,25	5,73	10,45
A_64_P162272		1,57E-13	0,670549983	1,03	1,28	3,47
A_64_P162599	Psat1	3,93E-10	0,613753741	1,06	1,03	-1,31
A_64_P162700	Kank3	2,07E-17	0,795187491	-1,06	1,53	2,26
A_64_P162760		6,48E-09	0,654296236	-1,06	-1,17	1,76
A_64_P162810	RGD1310110	3,86E-20	0,824585844	1,30	1,01	-1,67
A_64_P162940	Crxos1	1,04E-18	0,854202024	1,36	11,49	7,87

A_64_P163070	Grm4	7,79E-13	0,650998217	1,20	1,01	1,64
A_64_P163100	Ripply2	7,30E-16	0,781887953	-1,08	-1,20	-1,45
A_64_P163160	Lyrm7	7,42E-19	0,787980078	-1,14	1,27	3,26
A_64_P163165		3,81E-15	0,750805746	-1,39	1,73	7,01
A_64_P163244	Myom1	5,99E-10	0,607481811	1,31	1,21	-1,04
A_64_P163564	Elfn2	2,39E-21	0,827401638	-1,22	1,30	2,58
A_64_P163698	Onecut2	9,15E-14	0,78407322	-1,05	1,16	-1,38
A_64_P163787	Haspin	4,25E-14	0,66051714	-1,01	1,28	2,09
A_64_P163822	Cks2	6,20E-12	0,670312376	-1,05	-1,28	-1,14
A_64_P164081		3,87E-32	0,936047894	1,22	1,42	4,54
A_64_P164215		8,77E-12	0,704885628	-1,02	1,27	1,28
A_64_P164240		1,47E-12	0,687874428	1,03	1,26	1,36
A_64_P164340	Tead1	5,00E-12	0,626799567	1,07	1,11	1,70
A_64_P164351		6,37E-16	0,729786965	-1,14	1,40	2,54
A_64_P164547	LOC108349417	1,29E-24	0,899059413	1,23	2,85	7,45
A_64_P164642	Dqx1	6,06E-12	0,624217126	1,77	1,60	1,80
A_64_P164682	Etl4	4,15E-12	0,675293632	1,01	-1,33	-1,41
A_64_P164797	Ehd1	2,71E-14	0,6907587	-1,32	1,23	2,19
A_64_P164864	Kdf1	6,21E-23	0,833727269	1,45	1,48	5,78
A_64_P164926	LOC691931	8,88E-21	0,80210783	1,19	1,01	-3,03
A_64_P164961	Cyp2j3	4,54E-10	0,633678453	1,03	1,11	-1,40
A_64_P165015	Shcbp1l	4,17E-12	0,653487325	-1,98	1,44	4,22
A_64_P165075	Vom2r53	3,75E-26	0,871805	-1,38	1,81	18,32
A_64_P165085	Cdkn1c	2,67E-16	0,757347856	-1,09	1,05	2,49
A_64_P165521	Bpifb2	9,94E-11	0,610224765	-1,06	-1,02	1,30
A_64_P165848	Tmem51	7,10E-19	0,769215516	-1,11	1,29	4,37
A_64_P165913	Syt6	1,34E-27	0,929095258	-1,07	2,87	7,22
A_64_P165964	Hpca	3,69E-22	0,838593868	-1,01	1,09	2,45
A_64_P165986	Sh3bgrl2	2,16E-13	0,689418566	-1,21	1,86	4,33
A_64_P166218	Bfar	4,21E-20	0,790991871	-1,01	-1,43	-3,03
A_64_P166241		4,13E-15	0,687170189	1,49	2,04	2,51
A_64_P166385	Kifc1	5,54E-12	0,691742707	-1,02	-1,19	1,11
A_64_P203665	Tnxb	1,40E-17	0,743738639	1,27	2,19	2,91
A_64_P204094	RGD1308134	1,25E-12	0,689761351	-1,16	-1,02	1,23
A_64_P229432	Pparg	6,53E-21	0,860307858	1,27	1,04	2,37
A_64_P235760	LOC102549471	3,44E-13	0,661131104	1,04	1,18	2,54
A_64_P236773	Nudt6	2,04E-23	0,854493903	1,01	2,08	4,65
A_64_P236835	Ccdc96	2,58E-16	0,757661253	1,15	1,36	1,69
A_64_P237784	Rbm24	8,63E-30	0,947020639	1,29	-1,19	-2,21
A_64_P246030	Pfkfb1	1,81E-10	0,646684508	-1,35	1,25	2,34
A_64_P247541	Pcdh7	1,07E-19	0,817907768	1,12	1,00	-1,77
A_64_P264316	Mfsd5	4,15E-12	0,675312803	1,22	-1,02	-1,89
A_64_P267124	Rbfox3	2,46E-13	0,687942067	1,21	1,26	1,34
A_64_P267897	Efcab6	2,28E-13	0,709142539	-1,01	1,30	1,74
A_64_P271601	Nol4l	3,67E-10	0,636730149	-1,19	-1,88	2,78
A_64_P280765	Cd44	8,11E-09	0,631667418	1,44	2,10	-1,09
A_64_P281840	Nrg1	3,28E-11	0,706930438	-1,08	1,25	-1,14
A_64_P286274	Ttll7	2,15E-17	0,778781069	1,11	-1,00	-2,38
A_64_P307602	Tmem116	1,27E-16	0,745006832	-1,53	1,08	9,93
A_64_P312847	Zfp618	2,64E-19	0,811777196	-1,07	1,25	1,67
A_64_P315172	Nat2	1,82E-14	0,695160382	1,25	4,01	3,83
A_64_P327151	Rxrb	6,50E-10	0,628482816	-1,24	-1,29	-1,38
A_64_P351272	Rcor2	7,28E-27	0,878972617	-1,32	2,02	7,76
A_64_P351634	Csf1r	1,05E-12	0,670768239	1,51	3,16	1,06
A_64_P370146	Nrip1	7,78E-10	0,60351711	1,60	1,46	1,19
A_64_P386965	Ccdc191	1,77E-25	0,897729952	-1,05	1,17	3,23
A_64_P393739	LOC108348277	1,28E-13	0,715422798	1,05	1,15	2,13

Supplementary Table 2. Lists of genes with significant temporal expression changes in CGNs during rescue by PACAP (K5 + PACAP vs K5).

ProbeName	GeneSymbol.x	p-value	R-squared	K5_PACAP_vs_K5_05h	K5_PACAP_vs_K5_1h	K5_PACAP_vs_K5_3h
A_42_P453976	Ifitm6	2,45E-18	0,849122394	-1,351296524	1,102410002	1,699523487
A_42_P457692	Zfand2a	6,91E-13	0,696657286	-1,108275548	1,008309189	1,373516756
A_42_P459212	Tnfrsf21	3,49E-17	0,756548439	1,082158537	1,046684341	1,388812752
A_42_P459431	Bphl	6,18E-10	0,607016283	-1,097864359	-1,232374273	-1,021241606
A_42_P459855	RGD1562618	2,94E-08	0,630994556	1,356447452	1,116525819	1,30617144
A_42_P462257	Rprml	2,45E-31	0,953960826	1,204474708	1,19677727	1,293154991
A_42_P464378	Itih3	6,43E-14	0,722735839	-1,162466056	-1,00951291	-1,325471987
A_42_P467956	Sestd1	2,04E-11	0,607373758	-1,006727919	-1,007570781	1,261594565
A_42_P473398	Cxcl1	2,97E-26	0,912550029	1,250719005	1,91785661	-1,316330991
A_42_P473425	Unc13d	5,48E-19	0,86832868	1,849554382	1,133449387	1,41804528
A_42_P475871	Prss27	1,20E-12	0,645546403	-1,040955005	1,14077526	1,346523119
A_42_P479802	Dffa	3,31E-17	0,757009064	-1,080940625	-1,208082643	-1,332011797
A_42_P482623	Dstyk	2,45E-16	0,77525487	-1,108393507	-1,128813811	-1,171105234
A_42_P484738	Ctgf	2,06E-21	0,876696956	-1,032684867	-1,050195362	-2,097389347
A_42_P486203	Agtr1a	3,91E-11	0,646354473	-1,026496046	1,140062015	-1,399227879
A_42_P486964	Rph3al	7,34E-24	0,871786527	-1,02059011	-1,208000688	2,016952908
A_42_P491454	Gnat1	2,92E-23	0,886339724	-1,171304482	1,02096309	1,33570721
A_42_P491505	Purb	4,37E-14	0,70723156	-1,042259354	1,037552109	1,224479058
A_42_P493380	Myc	1,55E-25	0,914758821	1,003204872	-1,29776273	-1,523889971
A_42_P493925	Usp48	3,88E-14	0,745738479	-1,068914591	-1,13773504	1,145195725
A_42_P494276	Armc9	1,06E-09	0,642175204	1,051478367	1,08717253	-1,09390866
A_42_P495001	Slc17a5	7,94E-12	0,667195876	1,103366334	-1,131378665	-1,093825507
A_42_P495579	Ano1	3,07E-30	0,924994154	1,276031921	-1,112271801	2,667326996
A_42_P499221	Fgd5	5,70E-14	0,704336224	1,03332495	-1,009797062	-1,47592509
A_42_P499282	Msrb2	6,43E-13	0,697488438	1,146162368	-1,260483911	1,120973
A_42_P501233	Adra1d	1,17E-24	0,907811724	-1,114974565	-1,224769308	1,38582434
A_42_P501972	Ppp2r1b	5,03E-12	0,626713905	-1,178252155	-1,075617049	-1,074804659
A_42_P504653	F10	3,62E-15	0,732913491	1,525409437	1,121357557	1,144064626
A_42_P506402	Slc25a25	3,29E-12	0,678163418	1,016513033	-1,014740459	1,227100193
A_42_P508921	Aqp3	6,40E-17	0,801384405	1,10780634	-1,04421045	2,09357195
A_42_P515405	Ier3	5,01E-21	0,891250978	1,477422445	2,033594988	1,174545523
A_42_P517554	Polg2	1,27E-14	0,699079972	-1,033190748	-1,210479868	-1,098103515
A_42_P522194	Atp2a3	5,03E-27	0,918255793	1,474021523	-1,070792857	1,583694213
A_42_P523612	Mef2c	2,52E-12	0,701052746	-1,127241944	-1,048889308	-1,401387089
A_42_P528106	Glce	8,08E-16	0,764933358	-1,181677719	1,047068589	1,173971105
A_42_P528537	Cdkn2b	1,04E-10	0,654347621	1,330412567	-1,040799667	-1,196461364
A_42_P535152	Asap3	6,72E-18	0,770532368	1,069583771	-1,200308358	-1,115548215
A_42_P535644	Adamts1	4,73E-13	0,700981015	1,148788371	2,076038857	2,864810596
A_42_P536702	Sftpc	2,61E-23	0,886838817	1,569928788	1,14127817	1,452100293
A_42_P536741	Derl3	4,78E-11	0,620670756	1,723322523	-1,063182682	3,368461033
A_42_P538868	Dvl1	4,95E-11	0,643172217	-1,079094042	-1,101420736	-1,092627129
A_42_P540950	Ier2	8,42E-30	0,951959369	1,596535774	1,859609836	1,112906877
A_42_P540972	Rasl11b	2,64E-15	0,75420941	1,775982837	3,629037362	1,827268912
A_42_P541884	Irak2	3,87E-22	0,851777651	-1,107960939	1,073547481	1,184875396
A_42_P542960	Rerg	4,20E-10	0,655246957	1,535922157	1,233439882	1,257686334
A_42_P542985	Cbln2	4,02E-20	0,837932664	1,012414679	-1,195412285	-1,731561706
A_42_P546179	Tbc1d1	7,69E-10	0,603707673	1,00149539	-1,181420168	-1,21211783
A_42_P547036	Pak4	2,91E-10	0,697205897	1,205546601	1,051377241	-1,007811246
A_42_P547629	Fam49a	1,15E-11	0,719245696	-1,389828095	-1,255214071	-1,566871111
A_42_P548410	Acot1	1,18E-12	0,69045879	-1,124158561	-1,115393923	-1,424867729
A_42_P554157	Dnajb11	4,36E-14	0,685415329	1,098668577	1,066287008	2,137679415
A_42_P555140	Izumo4	5,38E-13	0,718463966	1,061845303	-1,146574422	-1,341337969
A_42_P555780	C5	2,52E-14	0,732383613	1,27103438	-1,351980812	1,075714552
A_42_P558703	Adamts15	1,32E-18	0,828862433	-1,152434304	-1,393699702	-2,067467857
A_42_P566264	Mfng	6,92E-24	0,882758247	1,147466467	-1,016783193	1,61286099
A_42_P567527	Traf4	4,67E-12	0,673855816	1,013003866	1,444243108	1,22417236
A_42_P568916	Gatad2a	1,57E-10	0,62712196	1,09764521	1,001677298	1,241588389
A_42_P573441	Klf3	2,45E-11	0,604794796	-1,145071302	-1,235763986	-1,141603751
A_42_P574859	Hamp	2,76E-13	0,773934318	1,880977798	1,310129549	1,674235665
A_42_P580973	Car4	1,84E-21	0,829030526	-1,177182973	-1,239363309	1,325654757
A_42_P581135	Ist1	3,64E-12	0,67692096	-1,012380216	-1,006497361	-1,256063691
A_42_P582467	Podxl	5,03E-12	0,626735081	1,199283071	-1,035529254	1,60495339
A_42_P584734	Gdf10	5,37E-31	0,936262669	1,313036299	1,02849557	1,695877662
A_42_P588785	Mthfd2	8,01E-20	0,81982588	1,093018424	1,044535095	3,467775757
A_42_P592157	Pbx2	9,33E-23	0,846348138	1,071959381	1,143856968	1,092686015

A_42_P593020	Rln1	5,87E-25	0,883089223	1,239458064	-1,653003512	1,615381027
A_42_P597322	LOC100125367	1,50E-20	0,866772702	1,173047318	-1,306012142	1,082058749
A_42_P603213	Elfn2	1,58E-22	0,868223343	1,026103913	-1,104976218	1,455656143
A_42_P603461	Isoc1	2,60E-13	0,726305858	-1,05156699	-1,094782169	1,251024757
A_42_P604033	Eaf2	8,62E-18	0,801815603	-1,120560163	-1,222430937	-1,304651388
A_42_P605985	Ddit4	1,83E-12	0,685268254	1,171771949	1,115315495	1,380451852
A_42_P608768	Bbs12	7,69E-18	0,802662896	-1,194170722	-1,304289658	1,001225986
A_42_P610788	Ampd3	8,17E-18	0,768917989	-1,040855828	-1,090619652	1,304249156
A_42_P612977	Cyb561	3,20E-20	0,839313148	-1,292464227	-1,258050322	-1,048120653
A_42_P614175	Gal	7,35E-24	0,859714292	-1,021873349	1,048417231	3,24901409
A_42_P618217	Fgf21	5,96E-19	0,820704335	1,461714608	1,146612502	2,968459994
A_42_P623839	Ttc39b	1,34E-23	0,879818133	-1,176833426	1,002130639	-1,31169291
A_42_P625147	Pglyrp1	4,12E-10	0,635105336	1,205983087	-1,031211494	1,334526719
A_42_P629321	Wisp2	4,74E-11	0,664771209	1,339840003	1,004514131	1,236777028
A_42_P629370	Klf15	1,24E-13	0,750888277	-1,215934794	-1,33264329	-1,405623971
A_42_P629500	Eif2ak3	2,26E-16	0,739678078	-1,236301714	1,158496983	1,46759114
A_42_P630572	Cpne8	1,01E-09	0,621964218	-1,624013865	-1,18500679	-1,09664627
A_42_P636761	Gzmm	4,82E-15	0,808847477	-1,245285035	-1,165687751	1,269983013
A_42_P638181	Serinc5	7,01E-13	0,65232153	1,019264363	1,078900036	1,408816294
A_42_P640792	Gjb6	1,97E-12	0,70388471	1,433181955	1,485224944	2,969267148
A_42_P646922	Sgcb	2,77E-20	0,852382831	-1,042969751	-1,08691744	-1,25269179
A_42_P648200	Pak1ip1	8,96E-12	0,685904066	-1,015233014	-1,043313666	1,354460011
A_42_P655897	Tspan9	2,26E-22	0,877126145	1,134903397	1,332106363	1,51794235
A_42_P656495	Actn3	3,51E-13	0,660885592	1,100339703	1,07966735	1,495036596
A_42_P661018	Phlpp1	1,38E-12	0,643780347	-1,183388074	-1,173294602	-1,093494217
A_42_P663500	Lgi3	5,31E-17	0,752857479	1,038543993	-1,254523217	1,477601975
A_42_P667148	Plscr1	2,05E-15	0,718192606	1,121511831	1,132073195	1,262623974
A_42_P668126	Sema6c	4,07E-17	0,773551266	1,105496038	-1,002332208	-1,745290052
A_42_P669103	Lnx2	3,29E-21	0,839704122	1,021892424	1,09544364	1,37740355
A_42_P670580	Vipr1	2,94E-33	0,947476406	-1,076361579	1,055110611	1,437967331
A_42_P670631	RGD1305645	6,60E-20	0,834896191	-1,154783221	1,024513795	1,078774257
A_42_P673905	Mrnip	3,96E-20	0,861635086	1,018300989	-1,071950527	-1,253815178
A_42_P681441	Eps8l2	7,28E-25	0,882167042	1,002773996	-1,33061022	1,695670788
A_42_P682589	Nr4a1	1,03E-28	0,951719014	2,084134579	4,48161681	1,700305165
A_42_P683837	Serpinb6b	3,05E-10	0,617484041	1,113318927	-1,424080193	1,204871499
A_42_P683840	Ccl27	3,87E-20	0,850478647	1,03932039	-1,039819977	-1,140005591
A_42_P684538	Slc2a1	1,72E-17	0,796629922	1,093147289	1,049672401	1,159508881
A_42_P686234	Muc4	2,84E-10	0,618531385	1,600323888	-2,03711718	-2,542738958
A_42_P686879	Siglec1	6,32E-10	0,649523091	1,544182443	1,352496706	1,302038177
A_42_P687819	Atoh8	1,08E-33	0,949395502	1,116442809	1,314343683	1,147342243
A_42_P688266	Arap3	1,98E-12	0,684276925	1,340183797	1,022568571	1,538015828
A_42_P694161	Arl14ep	3,47E-10	0,615612636	-1,313860246	-1,077467653	1,151060912
A_42_P694928	Arl5c	5,61E-19	0,834376323	-1,598720147	-1,103633137	1,820223278
A_42_P695042	Csf1	2,40E-18	0,83756443	1,090149156	-1,317914052	-1,193258406
A_42_P695401	Ccl2	2,50E-33	0,942131263	1,24740208	1,185344562	1,521650045
A_42_P708068	Egr4	6,61E-19	0,833330457	3,045726676	3,024568385	1,621135353
A_42_P708593	Per2	3,99E-18	0,821451542	-1,181270135	1,297422353	2,036096384
A_42_P712801	Entpd3	4,90E-27	0,925444078	-1,063228698	-1,155368094	1,697163433
A_42_P714311	Ccl3	1,78E-16	0,793430339	1,127073218	-1,356803362	-1,155151039
A_42_P718331	Sstr3	6,97E-37	0,975058259	1,199149888	1,193388487	-1,235806871
A_42_P721692	Inpp4b	8,16E-21	0,859112078	-1,203996205	-1,031941569	-1,160855966
A_42_P726234	Tex33	6,40E-09	0,614973196	1,344454387	-1,191821443	1,443473228
A_42_P727022	Rab40b	1,76E-10	0,601838374	1,083232624	1,002031114	1,56959922
A_42_P727368	Cdca7	8,62E-17	0,748523938	-1,02318988	-1,279234084	1,424352431
A_42_P735670	Mpp5	8,49E-15	0,743147782	1,159188422	1,031216186	1,138148006
A_42_P739860	Dusp5	6,20E-19	0,820446307	1,51038973	1,807693769	1,515612979
A_42_P740370	Cpxm2	3,63E-28	0,918784627	-1,03904802	-1,159792304	1,680059235
A_42_P743580	Rgs4	1,05E-28	0,929437293	1,046767023	-1,085930138	-1,702749667
A_42_P746838	Spib	5,54E-30	0,923364885	1,102921262	-1,12502248	2,307441545
A_42_P750340	Ifitm10	1,05E-16	0,782308144	1,344763462	1,301111427	1,114823663
A_42_P754654	RGD1562378	3,78E-25	0,911757871	1,161942486	-1,254321067	1,22712167
A_42_P758200	Rnf112	2,46E-12	0,660182055	1,099207051	1,073425781	1,12628557
A_42_P759843	Arl4c	1,35E-16	0,795599055	-1,17616255	-1,219123739	-1,222861742
A_42_P759993	Mefv	5,64E-12	0,671508243	1,238150974	-1,574144347	-1,146984663
A_42_P761023	Vstm5	2,33E-24	0,896772908	-1,235389891	-1,290313221	1,272938659
A_42_P762829	Cebpd	7,83E-26	0,900794423	1,156587087	1,318759165	1,506690127
A_42_P766909	Ppp1r14a	2,26E-14	0,76717353	1,275007267	1,29531261	1,129283256
A_42_P767077	Gja4	1,01E-11	0,68441906	1,23480595	-1,138008269	-1,203776256

A_42_P768024	Spn	2,58E-17	0,759178175	1,445946106	-1,117824546	1,72015165
A_42_P768355	Hrk	1,27E-34	0,953265179	-1,178474172	2,016641699	1,417805665
A_42_P771346	Acvrl1	4,64E-12	0,627795227	1,182450508	1,249335159	1,467498135
A_42_P776018	Stard4	9,07E-15	0,702699891	1,043283669	-1,035293292	1,231038379
A_42_P779933	Capn3	3,18E-11	0,669977703	-1,057176521	-1,019899926	-1,248129579
A_42_P780613	Hs3st2	4,24E-28	0,91022213	-1,104717669	-1,136212051	2,502670571
A_42_P787775	Plekhd1	2,04E-18	0,825970661	-1,193550616	-1,151179048	1,338628003
A_42_P791677	Areg	1,77E-13	0,711937816	1,290815995	1,861691985	2,333392737
A_42_P794120	Stra6	3,17E-21	0,852615142	-1,084394422	1,014347707	3,105075827
A_42_P794613	Mvd	2,53E-11	0,629516431	1,059504357	-1,094978673	-1,140792795
A_42_P795269	Lgalsl	2,15E-12	0,720992591	-1,274690502	-1,263146794	-1,376770624
A_42_P800949	Hipk3	3,71E-09	0,623458536	-1,203249697	-1,189727343	-1,134334776
A_42_P802358	Fam110b	9,61E-10	0,60030019	1,070973284	-1,158414466	-1,351557603
A_42_P803810	Myo5c	9,61E-11	0,634015497	-1,096651705	-1,209620303	2,142478312
A_42_P808945	Mustn1	6,51E-14	0,680844355	1,198977097	-1,192005614	-1,576555993
A_42_P811308	Phf21a	9,75E-22	0,858971106	1,058786364	1,028063167	-1,254864682
A_42_P812805	Smoc2	9,92E-09	0,608037116	1,009829018	-1,199822473	1,979585939
A_42_P817417	PVR	2,56E-11	0,629358033	-1,010948595	1,136358429	1,677527415
A_42_P820657	Il1r2	8,45E-13	0,747680923	1,013583685	-1,105606751	1,039417694
A_42_P822726	Myo1g	5,02E-12	0,692908712	1,197699964	-1,072553893	1,16426375
A_42_P824369	Foxf1	6,48E-22	0,882136732	1,413707252	1,870048517	3,703217538
A_42_P828242	Lgr4	9,43E-12	0,685275637	1,085177748	-1,178276498	-1,285082268
A_42_P829031	Coasy	2,27E-13	0,70919253	1,03036904	-1,062591885	-1,259182451
A_42_P834031	Hrasls	4,59E-12	0,652269679	1,112343398	-1,528063871	-1,281253548
A_42_P841193	Ddit4l2	2,67E-21	0,875440963	1,361859036	-1,495553769	1,498335291
A_42_P841704	Sh3tc1	3,30E-34	0,940190363	1,182262422	1,030805307	1,529485786
A_42_P842823	Reep6	5,65E-09	0,637185947	-1,199652554	-1,336874916	1,097071246
A_42_P842833	Srxn1	5,14E-18	0,752611684	1,053932375	1,030651064	2,100464991
A_43_P10102	Spry4	1,06E-17	0,814599362	1,151933138	1,89760679	2,742562883
A_43_P10133	Spns2	7,25E-28	0,92405592	-1,199176636	-1,218456753	1,153473532
A_43_P10136	Amz2	6,43E-12	0,669859485	1,027770644	-1,157413674	-1,300807202
A_43_P10225	Fgfr2	2,72E-11	0,628562077	-1,045306127	1,095001108	1,698335281
A_43_P10292	St6galnac4	4,32E-11	0,665989363	1,114575889	-1,13545025	1,031634303
A_43_P10498	Bag2	6,17E-28	0,924520993	-1,039954544	-1,115578724	-1,597225599
A_43_P10935	RGD1562339	2,49E-09	0,649420917	-1,178678665	-1,130349956	-1,307001249
A_43_P11044	G0s2	1,50E-20	0,855785142	-1,006630266	1,024338996	1,732548093
A_43_P11136	Col9a1	4,74E-12	0,673668725	-1,143334977	1,127289732	1,747016217
A_43_P11441	Atp4b	1,80E-19	0,853197569	1,392767699	-1,725754808	1,038927009
A_43_P11443	C4bpa	6,13E-26	0,901697378	-1,831671546	-1,700123771	1,017543803
A_43_P11471	Grin2c	4,61E-23	0,910101011	-1,232251888	-1,16808242	1,370703184
A_43_P11472	Hmox1	3,61E-09	0,602466186	1,04996803	1,022577616	-1,473175403
A_43_P11476	Inha	6,98E-11	0,638455962	-1,025147463	-1,374016691	-1,460065792
A_43_P11527	Adm	4,16E-18	0,807159068	1,335848871	-1,092545539	1,274289276
A_43_P11560	Aqp5	1,97E-12	0,783598759	1,940739833	-1,129453756	-1,031579422
A_43_P11616	Atf3	2,58E-17	0,845844687	1,070515689	1,203632736	1,498978406
A_43_P11654	Pnoc	2,03E-18	0,838601154	-1,583873154	1,193698167	5,262309575
A_43_P11683	F3	1,54E-15	0,721052092	1,134542403	1,00526143	-1,665265142
A_43_P11684	Alpl	1,72E-13	0,669467699	-1,598503009	-1,38890518	-1,324147301
A_43_P11685	Id2	4,67E-17	0,830209422	-1,052471736	1,321144044	1,064174722
A_43_P11820	Kcnj8	1,31E-13	0,750344385	1,232907078	1,22527259	1,572632347
A_43_P11861	Dio3	1,36E-24	0,898876428	1,192707104	1,101404532	2,145144302
A_43_P11897	Gabrd	2,10E-19	0,797364984	1,060133293	-1,175550837	-1,320189162
A_43_P11901	Kcnj5	7,45E-31	0,935482859	-1,166146531	-1,009700612	1,883692926
A_43_P11932	Nr4a3	1,78E-34	0,961556291	1,508148196	2,145019168	1,400960617
A_43_P11994	Cacng1	3,56E-18	0,775713775	1,104578843	-1,184065607	1,316867324
A_43_P12023	Nr4a2	4,96E-15	0,748295786	1,602655696	1,956006291	1,179071551
A_43_P12090	Pold1	8,10E-17	0,799578825	1,282371475	-1,079039835	1,19689191
A_43_P12151	Nrbf2	1,45E-11	0,612174993	-1,227954424	-1,402904837	-1,02573262
A_43_P12160	Unc5b	2,88E-12	0,634166721	1,09659842	1,288487508	1,130032801
A_43_P12246	Prpsap1	5,02E-12	0,651096535	-1,074130087	-1,050721709	-1,222920907
A_43_P12451	Slc16a3	1,09E-14	0,740692149	1,478417255	-1,047249944	1,301073622
A_43_P12460	Lrrn3	7,38E-18	0,802966625	-1,111479855	1,028053143	-1,448606807
A_43_P12535	Klf10	2,06E-25	0,897155155	-1,196020734	1,347150618	1,360711456
A_43_P12559	Camkk2	9,77E-13	0,671617743	-1,281891894	-1,342068572	-1,744452603
A_43_P12584	Nfyb	3,65E-13	0,739998929	1,027338134	-1,084101726	1,171248833
A_43_P12618	Nr1h3	7,26E-18	0,803093157	1,321375051	-1,056240235	1,253400436
A_43_P12619	Nr4a3	1,27E-24	0,879762397	-1,066489846	1,483058599	2,018066478
A_43_P12825	Entpd6	2,69E-10	0,641170114	-1,04447813	-1,094496709	-1,12038683

A_43_P12827	Nme3	1,04E-14	0,758335556	1,02797177	-1,096371826	-1,279429949
A_43_P12908	Klf4	1,21E-23	0,907266589	1,5449063	2,695912682	1,553824043
A_43_P12927	Dusp1	5,16E-21	0,849908009	1,199225196	1,836652907	1,088736553
A_43_P12967	Cap2	1,96E-14	0,783444359	-1,008083428	1,025481436	-1,203895108
A_43_P13041	Nr0b2	6,74E-11	0,660117737	-1,214208401	-1,607491501	-3,084842857
A_43_P13083	Tacr2	7,75E-18	0,769355487	1,375682336	-1,113333005	2,080477376
A_43_P13109	Cyp26a1	1,97E-13	0,74629897	-1,026045842	1,315847589	2,637505898
A_43_P13290	Pbld1	3,02E-22	0,865016362	-1,184618334	-1,289511483	1,177934729
A_43_P13355	Bambi	1,21E-16	0,781136286	1,162786543	1,410204794	1,741215877
A_43_P13906	Zmiz1	4,46E-12	0,628343167	-1,024897437	-1,099033221	1,366851205
A_43_P13931	Grem2	7,78E-11	0,636960134	-1,289140202	-1,406254551	-1,334074006
A_43_P14131	Rgs2	3,39E-12	0,656142246	1,030995003	-1,20677925	-1,537898946
A_43_P14752	LOC102554727	8,04E-13	0,714043632	1,106340029	-1,02198733	-1,31066474
A_43_P14809		1,87E-09	0,633808146	1,163489972	-1,01662332	1,230966867
A_43_P14872	Sdc1	2,79E-21	0,840669296	1,176514425	1,135091163	1,523991441
A_43_P14882	Errfi1	8,14E-21	0,847325477	1,028264011	1,21909372	1,427683216
A_43_P14911	Il1b	2,68E-14	0,765574431	1,336704926	2,080127846	1,564591356
A_43_P14977	Tsc22d1	7,74E-13	0,651082153	1,071273855	-1,019633151	-1,202199246
A_43_P15021	Slc25a29	1,61E-26	0,897483223	-1,050071089	1,156282847	1,378851319
A_43_P15154	Ihh	2,29E-18	0,81143918	1,396350442	1,219641833	1,615288655
A_43_P15246	Pthlh	1,65E-31	0,944745077	1,047597869	-1,206620317	-2,965060208
A_43_P15362	Slc9a5	8,55E-12	0,644144451	-1,066896702	-1,165159651	-1,096494671
A_43_P15390	Smad7	2,05E-14	0,783034366	1,383148051	1,023754122	-1,062979335
A_43_P15427	Sct	7,36E-21	0,84789863	1,820044659	-1,001537514	2,267241388
A_43_P15548	Gabra6	3,08E-13	0,741748159	-1,133674654	-1,024928941	-1,470404534
A_43_P15600	Rab29	3,12E-17	0,792019025	1,051987622	-1,160985465	-1,01145494
A_43_P15701	Rab13	5,23E-11	0,642432356	1,212772744	1,112869078	-1,147653652
A_43_P15805	Mlnr	1,06E-16	0,746658425	-1,134967637	-1,102059358	-1,521173224
A_43_P15824	Acvr2b	2,43E-11	0,630104835	1,01757569	1,041761431	-1,60988842
A_43_P15933	Chrm4	2,32E-12	0,720167366	-1,112236234	-1,333672941	-1,043872522
A_43_P16449	Pelo	5,38E-11	0,682635509	-1,079483411	-1,234056651	-1,174317669
A_43_P16457	Creld2	2,78E-15	0,735493768	1,132193495	-1,033477036	2,549740086
A_43_P16491	Col16a1	9,36E-24	0,891163634	-1,006430829	1,000840862	1,877447089
A_43_P17298	Naaa	8,42E-15	0,743228901	1,205440211	1,103567007	1,337802401
A_43_P17405		2,47E-21	0,841388964	-1,171286576	1,041529556	-1,822917828
A_43_P17681	Jmjd6	2,60E-11	0,603913998	1,124287028	-1,102502281	-1,261167572
A_43_P18332	Lrfn2	2,49E-26	0,920592462	1,074537722	1,05879521	1,455589551
A_43_P18571	Tekt4	2,37E-18	0,824971662	1,078205243	-1,474862902	-1,386866769
A_43_P18638	Asb18	1,06E-14	0,740982854	-1,216674895	-1,288121732	-1,691118606
A_43_P19279	Lyzl4	4,03E-27	0,902538826	1,441532293	-1,022652481	2,595695228
A_43_P19743	Krtcap3	5,30E-18	0,819487294	1,096942619	1,035844182	1,331779714
A_43_P19763	Tlr2	5,83E-19	0,770807838	1,214425038	1,322588204	1,092653751
A_43_P19848	RGD1309870	8,17E-12	0,64474308	1,3624173	1,087021802	1,200773913
A_43_P20515	Zfand1	9,57E-12	0,664822281	-1,314825298	1,091760505	-1,09554303
A_43_P20684	Tceanc2	3,64E-14	0,728633743	1,152569613	-1,113409556	-1,257895347
A_43_P21118	Klhl24	7,44E-14	0,721201993	-1,057835796	-1,018694506	-1,65714974
A_43_P21913	Usp53	8,24E-13	0,694626213	-1,635882967	-1,288473983	-1,123635531
A_43_P21945	Ppl	5,61E-14	0,704515613	1,181207367	1,641602603	2,14081095
A_43_P22225	Mdga1	3,27E-22	0,87538247	1,365735125	-1,097828897	1,357529736
A_43_P22501	LOC100909860	1,49E-18	0,799421987	-1,215875432	-1,257515704	-1,159201744
A_43_P22555	Fam189a2	9,92E-25	0,890949219	-1,264516077	-1,04229139	-1,848045978
A_43_P22744	Adcy9	2,03E-11	0,607421381	-1,077860521	-1,340759653	-1,186604978
A_43_P22811	Cd6	1,40E-22	0,927130608	1,522271543	-1,754436685	-1,02149497
A_43_P22864	Rad54l	3,70E-29	0,925398513	1,101651394	1,065656788	1,48311308
A_43_P23187		8,58E-16	0,780526318	-1,304870151	-1,051056979	-1,10912773
A_44_P1001782	Myf6	4,17E-13	0,65876667	1,10446147	-1,555285051	-1,826353331
A_44_P1004376	Ins2	2,16E-15	0,737951764	1,427514797	-1,195825934	1,53122724
A_44_P1004790	Slc30a2	1,36E-17	0,764672275	1,390307302	-2,564119767	-1,377817493
A_44_P1006068	Exoc3l4	2,53E-18	0,824547262	1,089391768	-1,526152913	1,157656388
A_44_P1007215	Upp1	4,27E-12	0,694842222	1,44852685	-1,071409117	1,354300757
A_44_P1011386	Znhit6	1,49E-09	0,67538751	-1,322464504	-1,072288406	1,20255751
A_44_P1012556	Stbd1	8,51E-10	0,700405534	1,648371928	1,178654994	1,798555077
A_44_P1014018	Mex3d	4,88E-14	0,759963918	-1,230332231	1,241077054	1,052618459
A_44_P1014659	Plek2	1,09E-31	0,926400336	1,09725991	-1,143579688	1,76080238
A_44_P101533	St6galnac5	2,17E-22	0,877313431	1,114600395	1,155274071	1,24898461
A_44_P1015388	Ankrd24	1,32E-12	0,644326878	1,008627167	-1,022903987	-1,129186589
A_44_P1015754	Slc22a3	1,43E-16	0,822389127	-1,466155547	-1,230839936	1,610997773
A_44_P1015839	Mroh7	1,86E-26	0,921491231	-1,14758385	-1,263726925	2,324501247

A_44_P1016691	Fhl4	4,38E-27	0,932236694	-1,043276235	-1,339215843	1,07686194
A_44_P1017595	Mospd4	1,32E-09	0,618010056	1,007673101	-1,579398855	1,130947963
A_44_P1017720	Ankrd34a	3,20E-18	0,776574985	-1,090748175	-1,394232778	-2,619698896
A_44_P1018090	Lgi4	5,61E-22	0,849755462	1,161504288	1,222476286	1,371811134
A_44_P1018447	Micall2	9,39E-11	0,65567414	1,087466059	-1,074966929	1,648225367
A_44_P1018696	Muc19	2,91E-12	0,679656348	1,043836223	1,080828451	1,593169759
A_44_P1019480	Cdc42ep2	5,00E-15	0,765090953	1,192456406	1,529013379	1,288450489
A_44_P1021808	RGD1561796	4,93E-08	0,622600385	1,385894385	-1,155536134	-1,011410808
A_44_P1022002	Ccl7	7,20E-32	0,934587173	1,339851536	1,07318207	1,138916201
A_44_P102369	Socs1	8,35E-13	0,69447158	1,389231376	1,854506957	1,041036029
A_44_P1024065	Hdac5	5,36E-21	0,881850931	-1,040735422	1,16270372	-1,512149039
A_44_P1024627	Kcnv1	2,78E-23	0,886552252	-1,038138666	-1,338125417	-2,25102394
A_44_P1028743	Cfc1	1,67E-22	0,867959854	-1,643031683	-1,706346178	-1,483860944
A_44_P1029805	Krt7	1,19E-29	0,935019361	-1,033140547	1,021455291	3,017883052
A_44_P1030258	Cnr1	2,17E-17	0,778723157	-1,08053269	1,202470679	1,754568168
A_44_P103127		2,80E-25	0,895974094	-1,520778826	1,094667834	3,448986304
A_44_P1031390	Trappc4	4,10E-18	0,791843836	1,164528054	-1,020249892	-1,192505293
A_44_P1033459	Uxs1	2,28E-13	0,688827206	1,115003026	1,253018834	-1,090162697
A_44_P1034018	N4bp3	1,36E-11	0,613117007	-1,054585024	1,048999994	-1,307340687
A_44_P1035007	Chrne	9,07E-22	0,83330377	1,0162097	1,10080377	1,738516308
A_44_P1035071	Nmt2	1,42E-11	0,699109593	-1,017916111	-1,08335633	-1,225864296
A_44_P1037806	Hpx	4,06E-27	0,902509589	1,214885627	1,24735982	1,677077664
A_44_P1039809	Rasip1	1,40E-12	0,707833068	1,038790136	1,129343544	1,31899665
A_44_P104054	Crhbp	4,65E-31	0,942537979	1,393492657	6,045981649	10,65458525
A_44_P1042696	Smim29	1,62E-16	0,761781466	1,132664726	1,072443475	-1,26557245
A_44_P1043302	Crb3	2,94E-15	0,785187838	1,049388125	-1,326806541	1,243971472
A_44_P1044030	Klhdc9	2,01E-27	0,921057741	1,014722942	-1,210396558	-1,28540492
A_44_P1044565	Cdadc1	1,46E-14	0,755187181	-1,194276304	-1,130238807	-1,078552049
A_44_P1044984	Atg16l2	3,88E-13	0,682634306	1,032917162	1,116275908	1,190006761
A_44_P1045652	Gnb1l	1,63E-13	0,692626754	-1,043670633	-1,260784632	1,175632421
A_44_P1045734	Stac3	7,10E-14	0,771845705	-1,101187417	-1,03566822	2,566016238
A_44_P1047158	Tnip2	1,50E-13	0,671138657	-1,126628763	-1,186095841	1,160185384
A_44_P1047924	Herpud1	9,88E-22	0,898196144	-1,11127837	-1,025290633	3,380549425
A_44_P1048999	Rhpn1	7,51E-14	0,679202713	-1,026668161	1,265806516	1,27169262
A_44_P1050200	C1qtnf12	1,91E-17	0,823802738	-1,210200428	-1,408514793	1,529812965
A_44_P1053605	Hcn1	1,64E-13	0,778756892	-1,317320258	-1,008045113	-1,2707347
A_44_P1053951		1,24E-26	0,915415157	1,238153857	1,388832535	1,081654335
A_44_P1054213	Hspa5	1,44E-21	0,830512849	1,173147642	1,14749321	2,668014487
A_44_P1057412	Ddx25	8,86E-16	0,764115857	1,184015609	1,028571796	1,211642787
A_44_P1058570	Sigirr	5,32E-17	0,80278447	2,156994563	-1,263252317	1,315151499
A_44_P1059903	Lzts1	5,04E-28	0,925095734	-1,040855647	-1,105838092	-4,879184034
A_44_P108003	Nipal2	3,51E-17	0,774772107	-1,064887563	1,062792613	1,188629449
A_44_P109927	Ahsa2	2,17E-11	0,654232207	1,090796659	1,254340759	1,185953628
A_44_P112082	Ccdc89	1,31E-11	0,681242714	1,000987501	-1,515496185	-1,480564488
A_44_P114638	Tsen2	9,78E-12	0,617663037	1,126915151	1,077706816	1,208602926
A_44_P114692		4,14E-17	0,804673899	-1,210057556	-1,13802181	-1,092424201
A_44_P115192	Slc5a5	1,31E-09	0,694691105	1,187536132	-1,390443777	1,745265168
A_44_P116283	Klhl41	1,96E-13	0,710811997	-1,736714647	-1,685911248	-1,353663151
A_44_P118666	Hs3st1	1,07E-22	0,858602714	-1,156747275	-1,217174854	-1,935034877
A_44_P118724	Arc	3,20E-24	0,89552772	1,285114139	1,768562556	1,890342805
A_44_P122386	Hspb3	2,31E-18	0,811368271	1,100072151	-1,733192225	-2,042512631
A_44_P125733	Madcam1	9,17E-12	0,618548912	1,189930164	1,121067295	1,235405535
A_44_P126154	Cdc37l1	6,31E-24	0,892784484	-1,185731114	-1,038249861	-1,221818051
A_44_P127597	Dnajb9	3,77E-11	0,667761118	-1,04211571	1,154196541	2,055251257
A_44_P127749	Tmem150c	1,08E-20	0,83257793	-1,087961549	-1,171339425	1,226883447
A_44_P128062	Syt17	1,07E-10	0,692277315	-1,012519423	-1,148777358	1,283056703
A_44_P131470	Hexb	9,80E-16	0,779399882	-1,106388304	-1,099042483	1,076429512
A_44_P132610	Slc20a1	5,70E-12	0,671362773	-1,020176717	1,122587142	1,398878417
A_44_P133335		1,97E-25	0,906037183	-1,26236422	1,22343174	2,659467119
A_44_P135224	Plk3	5,05E-10	0,652686008	1,105964195	1,752310815	1,219472335
A_44_P135356	RGD1565844	3,66E-16	0,787613829	1,062215547	1,235804721	1,431590981
A_44_P137633	Adora1	1,86E-11	0,608693553	1,279384722	1,035186534	1,471464891
A_44_P138355		4,75E-10	0,610943453	-1,014290212	1,186162486	1,459008633
A_44_P140148	Cd248	1,63E-15	0,740632439	1,318874903	1,196231428	1,63132579
A_44_P142242	Cdh1	1,07E-15	0,7446511	-1,393307053	-1,026114184	1,726339128
A_44_P152275		3,57E-10	0,615164069	-1,730255945	1,032036388	1,404615032
A_44_P153774	Plce1	6,12E-11	0,617188916	1,233228316	1,001812633	1,105467913
A_44_P156966	Ube2q2l	7,25E-18	0,817314131	-1,265118566	-1,064018386	1,174716267

A_44_P161220	Brinp1	4,00E-16	0,753715018	-1,114445859	1,075702547	-1,272369681
A_44_P163242	Epcam	7,65E-16	0,810010766	1,478750955	-1,299698983	1,440895291
A_44_P165999	Coq8a	3,74E-15	0,750965223	1,017756318	1,038080698	-1,381789836
A_44_P166161	Adh7	1,02E-21	0,869875625	1,258801812	-1,159216874	1,405496538
A_44_P166967	Npy1r	8,72E-18	0,76837784	1,039342629	5,118460002	23,0277478
A_44_P173568	Pop1	1,95E-18	0,812547603	-1,070502687	-1,087615231	-1,245400178
A_44_P174445	Lrrn2	4,65E-15	0,730430792	1,063254809	-1,097646481	-1,135379132
A_44_P175875	Pigv	3,27E-11	0,648781715	1,237836424	1,496584145	-1,284355246
A_44_P179145	Dgkg	1,41E-10	0,628658868	-1,172627398	-1,023076427	1,360849619
A_44_P180268	Lrrc8d	4,21E-16	0,770630611	-1,101745146	1,185528709	1,291539932
A_44_P184685	Sertad1	1,80E-09	0,613276981	-1,206607772	1,304963738	1,203148984
A_44_P185294		6,16E-32	0,946775522	-1,295519409	1,395129013	4,686679698
A_44_P185355	Bcl11b	1,36E-14	0,719574777	1,571654024	1,265299671	2,662258388
A_44_P185441	Tnfrsf21	4,04E-13	0,659174482	-1,033049375	1,216692394	1,510447668
A_44_P187195	C1galt1	3,81E-15	0,797316672	-1,296456176	-1,366357063	-1,673704528
A_44_P190229	Fam58b	2,83E-12	0,634376611	-1,006750466	-1,197763601	-1,166406909
A_44_P192083	Ffar4	7,29E-22	0,860495687	-1,255392374	-1,304590918	-1,306929663
A_44_P192406	Dusp9	2,95E-27	0,926891303	1,167771575	1,122240832	1,868790959
A_44_P196146	Aplnr	5,23E-11	0,642431508	1,208316051	1,273170386	-2,00314202
A_44_P196856	Cir1	5,05E-11	0,619897592	-1,122226087	-1,036322576	-1,111345194
A_44_P198705	H1f0	5,44E-22	0,908525853	1,300094786	-1,063956664	1,369548446
A_44_P202201	Grp	1,33E-22	0,879589088	1,151766288	-1,171892595	2,293886008
A_44_P203549	Egln3	1,13E-13	0,696731021	1,113594353	1,116720314	1,50944365
A_44_P204207	Smyd4	2,96E-10	0,617931362	-1,061104425	-1,039238123	-1,530285302
A_44_P206844	Hcrtr2	3,50E-11	0,647864896	-1,289375742	-1,092966589	-1,40010287
A_44_P209788	Enpep	1,85E-11	0,608764992	1,051467509	1,17213751	3,146340349
A_44_P210898		1,32E-16	0,763520396	-1,611012864	-1,351091873	-1,164596832
A_44_P211037	Adgrb2	2,32E-20	0,85338351	1,021335955	1,270506004	1,201859207
A_44_P211061	Etv1	1,88E-11	0,656076242	1,138088761	1,212423953	-1,256507803
A_44_P213175	Ccdc68	2,95E-13	0,685815734	1,196808619	1,374713746	4,835560415
A_44_P214288	Cabp7	1,00E-32	0,939117486	1,249713993	1,579593055	2,218780113
A_44_P216395	Gadd45a	1,86E-17	0,779958724	1,095283264	1,306190656	1,516329283
A_44_P220481	Tmco4	1,28E-10	0,62994803	1,243905623	-1,095859645	1,199191897
A_44_P220861	Npff	6,65E-20	0,821044154	1,06896724	-1,226626964	1,037338834
A_44_P221077	Tmem100	1,07E-19	0,802216441	-1,026348809	1,376635584	1,961661589
A_44_P224020	Kcna4	2,85E-31	0,943592452	-1,15357167	-1,017522636	-2,433360382
A_44_P227221	Syde2	1,20E-19	0,876227354	-1,008219037	1,306097203	-1,177522845
A_44_P229677	LOC363337	1,56E-18	0,827755619	-1,400990546	-1,192328324	3,197555278
A_44_P231243	Insig2	9,33E-11	0,655766369	-1,236574191	-1,108858856	-1,436384017
A_44_P231457	Kcna1	2,93E-15	0,714549263	-1,062991278	1,217372403	1,02029997
A_44_P231583	Kcnip3	1,20E-22	0,869594075	1,076168236	-1,011941135	1,287689001
A_44_P233080	Egr1	4,99E-30	0,957373853	1,928919318	3,23888014	-1,297878536
A_44_P236407	Lnx1	8,62E-24	0,900365503	-1,106415368	-1,055938828	-1,556263692
A_44_P236600	Lrrtm3	5,03E-24	0,884141799	-1,016123705	-1,057193903	-1,581072912
A_44_P240696	Nppc	2,79E-20	0,873824691	1,447950778	4,216470772	3,927858778
A_44_P241409	Vgll4	3,76E-17	0,80539583	1,048155825	1,112383569	1,298751576
A_44_P243355	Atp10a	1,44E-20	0,844041242	-1,115446179	1,02734204	-2,476859613
A_44_P243477		6,70E-15	0,818981266	-1,0687861	-1,246752483	1,02886914
A_44_P243534	Pxdc1	5,49E-12	0,710365891	-1,280095318	1,055632794	-1,529090451
A_44_P245640	Sftpb	9,25E-17	0,747877811	-1,33914615	-1,179926448	-1,295478255
A_44_P246114	Tle1	1,00E-13	0,736212666	-1,103872908	-1,19493648	-1,481234821
A_44_P247880	Csf2	2,51E-24	0,876717145	1,338218042	-1,620741594	-1,592545288
A_44_P248090	Thbs2	1,91E-09	0,689639607	1,414476751	1,043092824	1,175374256
A_44_P250203	Rbm12	1,78E-25	0,906395956	-1,113350206	1,079910929	1,017990713
A_44_P253012	Spry2	6,71E-13	0,67614065	-1,000926224	1,563514972	-1,112632498
A_44_P253208	Adamts9	2,01E-17	0,79543505	1,071637729	1,061116086	1,331300138
A_44_P258241	Dbx2	6,36E-14	0,703138308	-1,088494328	1,037767147	1,515816788
A_44_P260751	Nptx1	8,15E-28	0,923716062	-1,178968931	1,729960455	1,165769046
A_44_P265544	Crybb3	4,49E-16	0,838485367	1,603764457	-1,126188426	1,19153263
A_44_P267041	Lat	2,24E-11	0,631233025	1,040682632	1,039984788	-1,406427349
A_44_P269522	Tmem183a	7,78E-10	0,625835734	-1,143257795	1,121707202	1,174385402
A_44_P269930	Mfsd2a	4,28E-10	0,634525367	1,007486776	-1,014284931	1,255840479
A_44_P271658	Reg3b	4,09E-16	0,75350531	2,061853001	-1,044526294	1,764204901
A_44_P273777	Masp1	2,43E-13	0,727011863	-1,055475792	1,147969582	2,963853873
A_44_P276614		3,91E-15	0,711559851	1,253835906	1,62560162	2,401548259
A_44_P277010	Imp3	3,19E-14	0,688940725	1,057269907	1,013364613	-1,170782835
A_44_P279222	Faap20	1,18E-11	0,639844229	1,099533896	-1,069650055	-1,253571956
A_44_P282164	Pdia2	1,68E-27	0,921595318	-1,070421315	-1,091974146	1,410283102

A_44_P284067		1,41E-10	0,670081942	-1,169871304	1,079566813	1,217152653
A_44_P287301	Bcl6	1,24E-15	0,792397826	1,245567287	-1,129004667	-1,095235284
A_44_P287958	Dnmt3b	1,30E-24	0,899029071	-1,016079721	1,455966158	1,555180064
A_44_P290187	Sfrp4	1,89E-13	0,729657452	1,073234922	1,289421133	2,85437602
A_44_P291028	Arhgef19	1,57E-17	0,837492777	1,064869167	1,004201592	1,620015144
A_44_P291102	Smpd5	2,22E-17	0,760484325	2,305036523	1,148729051	1,663606354
A_44_P292437	Riox2	4,11E-14	0,727396714	1,002496945	1,012356624	1,270323377
A_44_P292980	Slitrk1	1,87E-14	0,752840373	1,046994352	1,048905431	-2,430809289
A_44_P293315	Bbs4	2,28E-09	0,609636454	-1,147481425	-1,066055841	-1,377001585
A_44_P293357	Ccdc51	9,56E-14	0,676410153	1,008980107	-1,141415385	-1,203570282
A_44_P296180	Arg2	1,95E-11	0,655594557	1,055370878	1,009893942	1,303976184
A_44_P297633	Irx3	2,29E-26	0,896152124	-1,034989549	-1,528490428	-2,116248976
A_44_P302536	Pnn	4,74E-12	0,67365655	-1,294499207	-1,182260772	1,014390521
A_44_P304740		2,77E-14	0,665580774	1,319261491	1,286439643	1,334244672
A_44_P305801	Jakmip1	6,77E-31	0,935711575	-1,348280584	1,013093603	1,489539209
A_44_P306602	Irf1	4,54E-10	0,63370084	-1,037648645	1,530226126	-1,138521054
A_44_P307181		5,33E-19	0,834702864	-1,958051078	-1,624582028	-1,061797243
A_44_P307559	Chrna3	7,71E-17	0,768169437	-1,214178507	1,094126797	2,330566291
A_44_P311620	Ahnak	2,16E-07	0,617074443	1,23325237	1,161266486	-1,36132126
A_44_P311917	LOC679711	1,48E-21	0,878289098	-1,436891851	1,052357208	4,174485216
A_44_P313272	Slc6a13	8,95E-12	0,704645209	1,036570935	-1,574224207	2,145874792
A_44_P314295	Zmynd12	3,81E-26	0,903423721	-1,19615156	-1,251064248	-1,190193299
A_44_P315905	Acr	3,06E-16	0,77336815	1,112388712	1,210473504	-1,42846323
A_44_P316319	Shkbp1	4,30E-16	0,770455353	1,168663997	1,083308529	1,270879689
A_44_P317388	Mcam	1,57E-22	0,868263472	1,103883299	1,527748462	1,900623404
A_44_P318662	Tac3	1,31E-32	0,93853331	1,175134883	-1,018992027	1,651652749
A_44_P321329	Polr2c	2,38E-12	0,660636846	-1,188872864	-1,064805377	-1,137061936
A_44_P321605		1,07E-13	0,717311696	-1,156641763	-1,048727945	-1,129869601
A_44_P323404	Cntf	4,28E-13	0,720968323	-1,112806553	-1,190877882	-1,209653379
A_44_P324430	Mdh1	3,99E-10	0,613540964	-1,102119545	-1,156189988	-1,104388928
A_44_P325189	Rcan2	4,96E-16	0,769212532	-1,44187776	1,242552294	-1,256080738
A_44_P325560	Apom	9,38E-23	0,859271065	1,158832459	-1,024977557	1,151715754
A_44_P327992	LOC100360244	1,64E-17	0,796990093	-1,302904219	-1,09444797	1,084241024
A_44_P328046	Masp2	2,35E-23	0,896404427	1,501872873	-1,244259559	2,221108701
A_44_P328489	Rprd1a	1,39E-09	0,617152777	-1,175399899	-1,289355082	-1,231506464
A_44_P330188	Acox2	2,32E-17	0,794301619	-1,194768519	1,137865235	1,610504732
A_44_P330847	LOC690467	1,06E-30	0,94070936	-1,34869876	-1,15209677	3,207177942
A_44_P333078	Adamtsl2	2,39E-25	0,896589689	1,156240141	1,259004753	1,867227353
A_44_P334736	Edn1	2,60E-16	0,757568535	-1,068804458	-1,346490891	-2,16556349
A_44_P335079	Tff3	2,99E-23	0,90386701	1,504266634	1,028894299	1,19937566
A_44_P335446	Dusp2	1,09E-23	0,899460386	1,17288806	1,724192938	1,492901106
A_44_P335578	LOC108348348	1,55E-16	0,779101875	-1,308240761	1,407688825	3,072125174
A_44_P337351	Cxcl12	3,50E-19	0,824251053	1,107627747	1,265347725	1,316500236
A_44_P338068	Ccdc134	6,55E-25	0,871354401	1,00927837	1,250745582	1,525584615
A_44_P339535	Tfpi2	5,03E-12	0,651067762	-1,389875059	2,379795017	3,172035089
A_44_P341870	Prkag2	2,02E-16	0,77687687	-1,220427757	1,074484276	1,175390954
A_44_P342538	Tcaf1	1,21E-14	0,720787093	1,014578976	1,013387055	-1,2836186
A_44_P344181	Map6	1,35E-11	0,66036737	-1,070855947	1,007434415	-1,391755062
A_44_P346832	Tesc	7,40E-18	0,842308436	1,380112658	-1,000527729	1,844175429
A_44_P346913		2,16E-19	0,827387405	1,161501792	1,167904167	1,218140751
A_44_P349553	Col6a1	9,15E-10	0,623437642	1,117938808	1,098643344	1,360147187
A_44_P351211	Phlda1	3,30E-22	0,864566884	1,158742837	1,481280336	1,201559008
A_44_P351723	Cldn10	4,09E-12	0,69536705	-1,003436839	-1,042457278	1,592752056
A_44_P352268	Plk2	1,52E-09	0,675084427	1,081610004	1,614616355	1,414189119
A_44_P353157	Tmem30b	5,68E-16	0,78398024	1,210380668	-1,999912097	1,300893628
A_44_P354078	Hist1h1a	2,77E-10	0,640737498	1,220065473	-1,000029154	-1,614272628
A_44_P354444	Bcs1l	5,25E-13	0,655934238	-1,043074466	1,012303842	-1,198754017
A_44_P355073	Adssl1	2,49E-10	0,699165401	-1,308195902	-1,017138057	-1,139310055
A_44_P356027	Sc5d	1,33E-12	0,667889827	-1,097164436	1,075524443	1,281080875
A_44_P356738	LOC102555114	8,63E-19	0,803414834	-1,233905236	1,29611677	2,913295614
A_44_P356829		3,21E-12	0,698217543	1,341272242	-1,291219579	1,842593961
A_44_P358216	Pank4	2,33E-13	0,665839953	-1,032446997	1,021910997	-1,241904494
A_44_P358974	LOC316124	1,32E-12	0,708465665	1,356866234	1,048162493	2,192412553
A_44_P360501	Gabrb1	3,50E-17	0,774802537	-1,145574389	-1,009476623	-1,311364041
A_44_P363549	Nsun6	5,74E-15	0,707558484	-1,054685675	1,009799649	-1,213218145
A_44_P369612	Antxr2	3,06E-12	0,657453983	1,261144412	1,202466288	1,37283894
A_44_P374618	Cdkn1c	5,67E-13	0,717896613	1,348156583	1,075216108	1,404548653
A_44_P374708	Cry1	4,72E-11	0,620851193	-1,247596696	-1,055873912	1,274803009

A_44_P374824	Klk1c3	3,70E-13	0,703756198	1,393384539	-1,128752443	2,110956647
A_44_P375658	Rbm11	4,83E-19	0,835327096	-1,174049389	-1,291343678	-1,326392818
A_44_P378742	Flrt3	2,16E-11	0,711917398	-1,003343976	-1,530584123	-1,261326682
A_44_P379891	Pspc1	7,26E-13	0,715175472	1,111340443	1,143292559	1,001720671
A_44_P380182	Mthfd2	2,86E-13	0,686191653	1,215949868	1,464960088	3,034686551
A_44_P381917	Sphk1	2,65E-13	0,759329845	1,472107982	1,677739363	-1,042102881
A_44_P383960	Kcna5	1,39E-14	0,698068606	-1,208358666	1,558539047	2,025912549
A_44_P390385	Phf13	2,31E-17	0,822478042	-1,013669867	1,953250387	1,113896973
A_44_P393822	Plekho1	1,80E-25	0,897659543	-1,045396127	-1,071376187	-1,659703003
A_44_P398376	Nudt18	4,80E-21	0,823042264	1,070541705	-1,034251005	1,173980789
A_44_P400324	Maf	6,04E-10	0,62955023	-1,318594199	-1,222522463	-1,291568186
A_44_P401955	Zfp706	6,58E-13	0,697222782	-1,277900492	-1,156219982	-1,066252822
A_44_P402980	Nkx6-2	3,79E-11	0,623935291	1,407687317	1,390982028	1,697988119
A_44_P404064	Tiparp	1,24E-18	0,829292569	-1,004796212	1,191526229	-1,185815188
A_44_P409518	Gmpr	6,17E-12	0,670366599	-1,140099983	-1,012337553	-2,104523997
A_44_P409803	Cd5	6,75E-16	0,766518264	-1,428653021	-1,013559398	-1,860317182
A_44_P409820	Faslg	3,85E-19	0,848772737	1,018483593	-1,059782265	1,439903622
A_44_P412121	Zfp335	5,81E-12	0,649199256	-1,200452577	-1,03909773	-1,213478443
A_44_P412236	Stag3	7,98E-12	0,620455067	1,126421651	1,382667794	1,899266778
A_44_P414527	Efna2	1,51E-15	0,775697554	1,010684494	-1,3725049	-1,186781498
A_44_P416738	Fscn3	2,69E-10	0,619314796	1,107225942	1,050735851	1,544670314
A_44_P419676	Zc3hc1	9,17E-11	0,63467522	-1,074314002	-1,009516492	-1,440370672
A_44_P419922	Haghl	2,08E-10	0,644775541	1,276271505	-1,046508609	-1,714135266
A_44_P420640	Nup43	1,68E-19	0,841844413	1,052169657	-1,064196347	-1,14011081
A_44_P421887	Ankrd33b	1,50E-17	0,781702517	-1,32238703	-1,146992778	1,139380326
A_44_P424723	Sik1	3,26E-18	0,822827036	2,111747832	6,722919151	4,220417813
A_44_P428326	Hmgcs2	6,88E-10	0,605384196	1,402256636	1,588408295	-3,808522582
A_44_P429453	Lama5	4,17E-11	0,704018627	1,082784826	1,019816498	1,493218378
A_44_P430547	Ntrk1	1,24E-27	0,941252005	1,444027257	-1,140482235	1,650257895
A_44_P430581	Nsmce4a	8,21E-14	0,770501682	-1,154571981	-1,034549138	-1,150745092
A_44_P431049	Rrad	6,98E-29	0,907268074	-1,12536053	-1,413595272	-3,785388569
A_44_P437956	Stc1	2,16E-24	0,905588152	1,216522497	1,855872475	2,742250048
A_44_P440556	Muc13	2,47E-11	0,652512125	-1,081340132	2,766774529	1,327008029
A_44_P440944		4,56E-28	0,938035432	-1,421819641	1,297076074	4,679038826
A_44_P442780	Kcnab1	9,98E-15	0,789268909	-1,040934506	-1,092304711	-2,384404878
A_44_P445044	Apold1	1,21E-11	0,661820035	1,108003601	1,676358626	-1,014197434
A_44_P448051	Vpreb3	3,70E-10	0,711084256	-1,063853282	-1,58940752	-1,003044324
A_44_P450486	Ttll1	2,33E-11	0,711035543	1,05730031	1,151605754	-1,23854769
A_44_P455059	Azin2	3,85E-19	0,792910909	1,192123019	1,49625634	1,202530512
A_44_P456599	Zdhhc23	3,20E-10	0,616777708	-1,120477128	1,064542952	1,7669248
A_44_P457099	Plpp5	8,36E-12	0,686753337	1,121743808	1,181051217	1,485151554
A_44_P457548	Parp9	5,52E-11	0,618641028	1,14620556	1,048306269	1,346445701
A_44_P458021	Tnfrsf11b	1,97E-10	0,645533227	-1,225588606	-1,355091036	-1,867161372
A_44_P458901	Neurod2	7,85E-11	0,636836903	1,209299501	1,346194517	-1,14696369
A_44_P461383		7,45E-16	0,796498063	-1,760390303	-1,217653681	1,013117847
A_44_P463749	Rasd1	9,48E-18	0,785346524	1,164979437	-1,3557516	-1,789765408
A_44_P464330	RGD1304963	4,92E-17	0,753534035	-1,046662286	1,310579639	1,280019031
A_44_P466434	Fam212b	5,57E-13	0,699130704	-1,029856975	1,0443851	-1,236198937
A_44_P467202	Pcdh8	8,84E-22	0,859486932	1,017720744	1,215417778	1,457305857
A_44_P467591	Neu2	3,74E-27	0,902803262	1,056251068	-1,083215794	1,578057419
A_44_P468239	Sgtb	5,03E-13	0,700290857	-1,274761294	-1,008575503	-1,80048933
A_44_P469584	Cd14	2,23E-12	0,637529702	1,268950929	1,106804348	1,04112437
A_44_P470528	Ndufaf4	1,58E-14	0,754423021	-1,087188342	-1,144807914	1,316451709
A_44_P473234	LOC102554302	1,00E-12	0,692362996	-1,034369119	-1,129742638	-1,279571638
A_44_P474101	Slc7a1	4,63E-21	0,862130684	1,062345013	1,104183796	2,204558178
A_44_P477406	Slc2a4	2,30E-15	0,755484697	1,13244911	-1,034368441	-1,489661273
A_44_P480573	Cytip	2,19E-22	0,866612871	-1,23624808	-1,108135622	-1,704848806
A_44_P481915	Id4	1,87E-13	0,762673314	1,114709814	1,251757865	1,458394171
A_44_P482519	Itgb7	2,57E-18	0,778326675	-1,524112186	-1,10476961	1,163588223
A_44_P483728	Rps6ka1	1,48E-11	0,636844124	1,165522287	1,087674634	1,181314334
A_44_P484984	Gnmt	1,86E-11	0,656262525	1,146787417	-1,020789402	-1,314414581
A_44_P485948	Cux2	6,88E-12	0,707745127	-1,383753341	-1,06205687	1,288523063
A_44_P491393	Nudt7	7,93E-10	0,603238402	-1,045011457	-1,138114346	-1,400248554
A_44_P491759	Krt24	2,23E-18	0,77945973	1,148357219	1,086520364	2,081955309
A_44_P493005	Foxq1	1,59E-12	0,724339605	2,496717058	2,254201685	1,117129627
A_44_P493911	Slc13a5	8,66E-16	0,746599735	1,086619572	-1,151375115	2,70744072
A_44_P493980	Nptxr	1,79E-17	0,810859116	1,313214987	1,542754294	2,031872675
A_44_P494439	Cnnm2	5,09E-14	0,683660798	1,00574989	1,079332679	-1,408990183

A_44_P496447	Ntf3	3,16E-24	0,89557226	-1,147615334	1,239027965	3,18223262
A_44_P497174	Gramd1b	1,31E-09	0,618084755	-1,177479191	-1,09187481	-1,308248991
A_44_P500431	Lman1	4,50E-09	0,640632932	1,025048108	-1,090692562	1,208567178
A_44_P501761	Crybg2	4,81E-19	0,835342262	1,109347979	-1,056995538	1,74332917
A_44_P501827	Cpsf6	1,59E-10	0,626948053	-1,189542535	-1,10206855	-1,231445201
A_44_P504750	Rassf9	3,33E-09	0,603712828	1,028870331	-1,250076184	-1,573661846
A_44_P506299	Bcl2l15	4,94E-26	0,918455181	-1,391490539	-1,679812623	1,111940815
A_44_P508691	Tm6sf2	1,36E-28	0,92170148	-1,087237287	-1,045390512	1,283829636
A_44_P511359	Mum1	4,23E-18	0,774315476	-1,052739364	-1,076986104	-1,652954336
A_44_P512136	Dhcr7	3,10E-23	0,864846271	1,104770613	1,01081478	1,277564916
A_44_P513385	Dab2	4,46E-08	0,62424575	-1,010574765	1,253839622	1,144760617
A_44_P514927	Fam69b	2,41E-15	0,755074798	-1,049433214	-1,072005618	-1,175178657
A_44_P520700	LOC501266	7,15E-18	0,830451337	1,117225724	1,109919922	1,201519972
A_44_P522047	LOC684871	3,96E-12	0,747354883	1,272695597	1,558896856	2,537962267
A_44_P523028		3,76E-27	0,919156763	-1,159714014	-1,094976046	2,67376005
A_44_P524630	Qrfp	2,83E-12	0,634407405	1,638377888	1,64359862	3,369012544
A_44_P525059	Ficd	7,15E-16	0,728653125	1,23507534	1,661508189	1,920327334
A_44_P527270	Pth1r	6,13E-12	0,648504846	1,201436972	1,123225045	-1,473914472
A_44_P530131	Asb2	2,05E-30	0,933013633	1,089062914	-1,369708497	-1,11555976
A_44_P532249	Fkbp5	2,06E-12	0,783155091	1,197369461	-1,029586573	1,358736537
A_44_P534089	Ccnb1	2,07E-11	0,654840673	-1,173593536	-1,168098731	-1,756278808
A_44_P537652	Arid2	2,25E-15	0,772237761	-1,143802248	1,025247929	-1,073619515
A_44_P538521	Star	9,76E-15	0,741795502	-1,150694133	-1,135795751	1,000405876
A_44_P540517	RGD1564664	2,62E-13	0,707625437	1,237168195	1,028199945	-1,276396051
A_44_P541020	B3galt4	4,39E-17	0,789320196	1,154646402	-1,000842348	-1,245685245
A_44_P545887	Ccdc155	3,73E-19	0,809379286	1,020709277	1,074442197	-1,293705914
A_44_P547771	Cited1	2,22E-22	0,854766183	1,122227118	-1,001221849	1,447495988
A_44_P547788	Clk3	7,52E-24	0,859601099	-1,11800144	-1,024190541	-1,382751493
A_44_P548726	Zfp414	6,88E-21	0,835318762	1,064455612	1,146255757	-1,423505039
A_44_P548730	Bcl9l	7,17E-12	0,646463772	1,134983017	1,008613588	-1,955240698
A_44_P556989	Ngfr	2,58E-16	0,830563218	1,886738381	1,119711968	1,404924692
A_44_P557376	Drd1	5,82E-12	0,624751147	-1,251013389	-2,21682936	-3,118481499
A_44_P561324	Akap1	4,90E-13	0,656794643	-1,114446971	-1,259609951	-1,528608153
A_44_P577108	Rasgef1c	3,08E-20	0,839534473	-1,037169812	-1,163682169	-1,507054447
A_44_P579382		4,25E-12	0,746599062	1,669379853	1,16145659	1,0866809
A_44_P592627	Samd10	1,12E-10	0,608508544	1,022813048	1,126906863	-1,267083605
A_44_P606049		5,41E-11	0,61893277	-1,029408214	-1,992076844	1,204192886
A_44_P606216	Tmem269	7,51E-24	0,87167909	1,060213378	-1,649304925	1,152110585
A_44_P606502	Fam83f	6,08E-29	0,916363103	2,587847011	1,229995656	1,850566013
A_44_P607542	Btbd11	1,80E-14	0,753158724	-1,255110026	-1,0631262	2,196335501
A_44_P608167	Tmem116	5,06E-23	0,87372165	-1,174980586	-1,602243348	1,031986287
A_44_P608485	Robo3	1,29E-20	0,856642841	-1,097914623	-1,704280604	1,562026901
A_44_P612762	Lrrc8c	2,84E-12	0,679967328	1,037454798	1,481426123	1,315022287
A_44_P613170	Ndp	2,34E-15	0,771887242	1,053415907	-1,093193737	-1,535448581
A_44_P620106	Tgfbi	5,69E-11	0,64127927	1,016688934	1,000294534	1,262797185
A_44_P620941	Tmem200a	4,65E-19	0,82236695	-1,325867208	-1,404497226	1,47781236
A_44_P622113	Sept8	3,42E-13	0,704643869	1,128475283	1,073043037	1,454469254
A_44_P623083	Slx1b	9,64E-13	0,671780522	-1,062661243	-1,035527628	-1,35076716
A_44_P623694		9,68E-16	0,763336593	1,167449334	-1,080781028	-1,299955133
A_44_P625333	Ctdspl	6,90E-12	0,668977549	1,08587029	1,06844887	1,328208844
A_44_P628515	Rgs7bp	4,46E-14	0,77610683	-1,167956951	1,046783805	-1,374813268
A_44_P634770	Cxxc5	9,77E-22	0,846672796	-1,03531296	1,140438419	1,098599408
A_44_P635089	Etv5	3,83E-12	0,630365749	1,154996028	1,019826442	1,566192584
A_44_P638511	Paqr5	2,82E-26	0,927060066	-1,224662949	-1,748349727	1,118656807
A_44_P639365	Col24a1	1,30E-10	0,723900863	-1,161231081	-1,243588775	1,124030747
A_44_P671089	Foxc2	2,03E-12	0,683990762	1,442897602	6,636823005	6,031077608
A_44_P684795	Irs2	8,87E-14	0,719343757	-1,098236361	1,356926127	1,15299378
A_44_P685298	Ice2	4,79E-09	0,619514777	-1,180575081	-1,06332519	1,176079169
A_44_P686406	RGD1562310	4,17E-26	0,893851035	1,09266471	1,184681173	1,413107382
A_44_P696669	Ehd1	1,71E-19	0,814757972	1,187780984	1,209131837	1,121350041
A_44_P715030	Vstm2a	1,68E-11	0,6101296	-1,01100907	1,287120451	2,90729563
A_44_P716637	Msx2	2,41E-19	0,826702301	1,197125903	1,534768447	1,451094233
A_44_P727824	Ppp2r5a	8,63E-14	0,737747517	1,047342713	1,085113545	1,36890241
A_44_P729856	Ppp1r3d	1,69E-14	0,811774028	-1,035838873	-1,181922337	1,050224961
A_44_P761066	Atg5	1,43E-10	0,604957201	-1,055266678	1,04162354	1,107136819
A_44_P764174	Fcmr	4,45E-23	0,893798951	1,369099235	-1,308928906	1,59586056
A_44_P776231	Tp53i11	4,21E-24	0,874362353	1,40790179	1,532491732	1,553389479
A_44_P776390	Kcnk5	4,49E-18	0,773829233	1,394449488	1,187683141	1,929931042

A_44_P777016	Ypel4	6,62E-19	0,833322469	-1,1711567	1,022523202	-1,110683142
A_44_P777424	LOC100362783	2,89E-13	0,75845308	-1,433086269	-1,474235776	-1,31534906
A_44_P779296	LOC502684	5,97E-26	0,881927766	-1,0426723	-1,13611921	-1,173920085
A_44_P787936	Stat3	2,52E-15	0,771216365	-1,103058129	1,179056028	-1,089736325
A_44_P790674	Opn3	3,09E-23	0,852305341	-1,290848474	-1,127282278	-1,635992915
A_44_P792628	Spef1	1,31E-09	0,618064807	1,086951027	-1,248169177	1,11060605
A_44_P808544	Gpr61	2,73E-16	0,737875609	-1,132759916	-1,295656438	-1,289392592
A_44_P808710	Rd3	4,22E-12	0,675090931	1,077791064	-1,233182162	-1,460778848
A_44_P809374	Clcf1	6,00E-14	0,723462738	1,290490857	1,15021749	1,553865509
A_44_P809486		7,19E-22	0,8605643	-1,007609738	1,465215377	2,770827505
A_44_P817760	Tspan15	1,29E-22	0,857608887	-1,035392857	1,652859575	1,300968021
A_44_P821368	RGD1560958	1,45E-20	0,844004008	-1,018084176	-2,017835029	-1,294361992
A_44_P822447	Ctla2a	3,25E-15	0,784336771	1,31629247	1,333728154	1,534987067
A_44_P822557	S100a14	3,55E-14	0,709453673	-1,205336135	-1,605722846	1,302600784
A_44_P823666	Zhx2	1,37E-13	0,694630016	-1,161952673	-1,066469757	1,268887768
A_44_P823749	Scn5a	6,78E-10	0,648517067	1,55389208	-1,152981209	2,220487377
A_44_P832655	Dedd2	2,23E-09	0,63113308	-1,168999417	1,10627596	-1,514026125
A_44_P839176	Usp25	1,09E-16	0,782032062	-1,342705702	-1,01965775	-1,171005701
A_44_P839270	Pax1	2,08E-21	0,828257042	1,077899573	1,937587738	2,940500019
A_44_P839975	Tmem132e	5,67E-12	0,649522741	1,203979097	-1,015569378	-1,278473777
A_44_P847319	Luzp2	4,17E-14	0,776710041	-1,164391812	-1,119639058	-1,777083129
A_44_P852545	Tmem196	9,58E-22	0,870172597	-1,072244637	-1,225582634	-2,317627165
A_44_P868418	Igsf3	3,92E-11	0,623466282	1,120692564	-1,033687302	1,663523135
A_44_P868890	Gli2	1,86E-17	0,810594262	-1,216638307	1,010478906	3,544070247
A_44_P870348	Slc35d3	2,33E-12	0,6093104	1,287229898	1,33199431	1,522876315
A_44_P876363	Vav2	9,13E-24	0,935240588	1,203377569	-1,121287597	1,910643214
A_44_P878711		2,90E-09	0,605912324	1,559769515	-1,342995639	-1,246449757
A_44_P879764	B4galt1	1,47E-10	0,604474192	1,035617819	1,182217992	1,365111301
A_44_P883462		6,32E-24	0,872485356	-1,056798167	-1,838522803	-6,777401262
A_44_P898436	Lrrc17	2,68E-16	0,790137703	-1,159758574	-1,186434015	1,325579871
A_44_P899127	LOC500300	1,72E-31	0,944662039	-1,045684765	-4,687492883	-5,219305864
A_44_P899293	Snai1	7,45E-20	0,834139402	1,343391532	1,190371912	1,536083775
A_44_P912777	RGD1310553	2,09E-11	0,654680347	-1,184320507	-1,133073792	1,116585232
A_44_P917327	Lrtm2	8,31E-32	0,946166942	-1,151481554	-1,008777365	1,265305328
A_44_P917564	Mat2b	1,32E-15	0,77687816	-1,216716518	-1,093027722	-1,580013002
A_44_P929423	Itga9	2,84E-16	0,774004621	1,168717389	1,027399426	3,037543607
A_44_P929530		5,58E-33	0,951401963	-1,091782608	1,307771057	3,311200336
A_44_P930152	Mpped1	1,28E-22	0,898155721	1,664388828	1,245532727	3,238417901
A_44_P930534	Gas2	1,72E-16	0,778206333	1,325781751	-1,278144962	1,377624017
A_44_P940501	Tnrc6c	1,28E-11	0,638767333	-1,261371816	-1,063391965	1,355366295
A_44_P944353	Armc2	5,47E-13	0,699333674	-1,085707992	-1,106780751	-1,898920886
A_44_P945723	Zc3h12d	4,79E-15	0,781007713	2,256524793	1,120471925	1,309536526
A_44_P945759	Cep41	6,59E-18	0,803805367	-1,07829123	-1,113761316	1,193491755
A_44_P946504	Foxj2	1,68E-11	0,610092191	-1,139855646	1,050220009	-1,294301209
A_44_P951676	LOC497899	3,80E-21	0,863164468	-1,005313684	-1,19968071	1,0000814
A_44_P956272	Ogt	2,08E-14	0,715122327	-1,093353997	-1,109501883	-1,292101792
A_44_P962701	Nol4l	1,50E-20	0,855786916	-1,24554446	-1,090646325	1,398608133
A_44_P963812	Ctnnal1	3,33E-10	0,616225652	-1,112545901	1,012637145	-1,261412557
A_44_P988767	Crem	6,12E-12	0,648535038	-1,278135775	3,389804004	4,798813016
A_44_P989558	Slfnl1	3,36E-12	0,677914856	1,025446102	-1,128282284	-1,205341908
A_44_P994550	Rars2	1,27E-11	0,638919698	1,068268961	-1,136029277	1,177557779
A_44_P997843	Csrp3	5,34E-20	0,848630737	-1,257141174	-1,392216067	-2,152855114
A_44_P999125	Masp2	1,44E-18	0,852322783	-1,065574823	-1,647537474	2,029908714
A_44_P999395	Lmbr1l	2,06E-09	0,688606121	1,023256513	1,285052472	-1,249975965
A_64_P000041	Cfap58	6,07E-25	0,910121777	1,227328973	1,233685945	1,737973519
A_64_P000095		3,76E-19	0,793090678	1,071961164	1,724331551	3,876902088
A_64_P000708	Rnf125	2,15E-13	0,689470927	1,132911644	1,124707296	1,327576935
A_64_P000895	LOC108348336	8,74E-15	0,742869556	-1,184913685	-1,050962925	-1,07803796
A_64_P001096		4,12E-22	0,851443556	-1,253821279	1,283534823	2,792592561
A_64_P001219	Mthfs	7,29E-10	0,647495278	1,311916546	1,100069341	1,193384348
A_64_P001426	Tmc8	3,42E-19	0,793787392	-1,007844218	1,026734245	2,923660919
A_64_P001461	Dnmt3l	3,55E-19	0,809720541	1,342677963	1,398900494	2,232065711
A_64_P002097		6,66E-21	0,820952522	1,356519644	2,338580191	3,238307756
A_64_P002110	LOC363337	1,24E-20	0,867749973	-1,539437472	-1,153854974	2,548446205
A_64_P002339	Zbed4	8,75E-11	0,676383332	-1,134189983	1,019349936	1,34562355
A_64_P002479		3,60E-22	0,852176704	1,099206782	1,158433702	2,714947311
A_64_P002899		2,34E-13	0,744546856	1,449379546	1,202437085	2,358829922
A_64_P002949	Disp1	8,83E-20	0,819176825	-1,021656786	1,016823522	1,536526128

A_64_P003327	Lix1l	6,62E-17	0,750897022	-1,043321078	-1,06194182	-1,32092332
A_64_P003378	Tuba3b	5,26E-12	0,728128832	1,238425197	1,000161641	-1,782846526
A_64_P003749	Pax4	3,71E-13	0,722491728	-1,0769578	-1,22624552	1,141188964
A_64_P003876	Fxyd3	3,01E-27	0,893904139	1,409158898	1,017452265	1,174193276
A_64_P003948	Slc24a4	2,05E-13	0,710332119	-1,496248437	-1,140681943	1,782984424
A_64_P004007	Rd3l	1,11E-28	0,922281475	-1,083812071	-1,204178483	-1,456981541
A_64_P004307	Pipox	1,29E-12	0,689378333	-1,00741401	-1,443534062	-1,288829731
A_64_P004584	Cacna1g	2,31E-30	0,925774713	-1,047446741	1,123233298	1,268168722
A_64_P005053	Vsig8	4,00E-17	0,790059461	-1,513825905	-1,608993045	1,622760814
A_64_P005228	Ppp1r1b	1,57E-22	0,868272513	1,036389195	1,097164256	1,479589739
A_64_P005391	Drgx	5,80E-23	0,861719617	1,271410625	-1,258490547	1,410216533
A_64_P005987	Agfg2	2,34E-13	0,727431096	-1,049927689	-1,169690649	1,159300972
A_64_P006072	Zfp536	4,17E-13	0,702402995	-1,126716742	-1,147791729	1,346798902
A_64_P006183	Gjd3	1,91E-30	0,926279577	-1,016152766	-1,253289722	-1,209345861
A_64_P006473		1,55E-23	0,889047561	-1,108145523	1,023075581	3,560311077
A_64_P006475		4,19E-15	0,710843347	1,190473701	1,412072507	4,143041294
A_64_P006525	LOC681364	3,25E-29	0,932498521	-1,166407798	1,053875074	3,063837114
A_64_P007133		8,70E-21	0,819229328	1,150095075	1,726412218	3,782642152
A_64_P007135	LOC108349417	9,19E-19	0,786352549	-1,054047768	1,244700187	2,871240742
A_64_P007178		4,26E-17	0,789570019	1,1839072	2,093646831	4,931057582
A_64_P007508	Csf3	7,82E-16	0,747555044	1,188135066	1,152851932	-1,498925521
A_64_P007518	Ttc39a	5,01E-11	0,620016608	-1,209582314	-1,296806224	1,290570828
A_64_P007633	C4bpb	6,08E-14	0,723327176	-1,377700831	-1,242627347	1,196764014
A_64_P007833	LOC685125	2,51E-18	0,795578454	-1,415502719	-1,093557644	3,043972869
A_64_P008042	Ankrd9	1,20E-13	0,696139182	-1,0486789	1,03154139	1,476356157
A_64_P008060	LOC108348293	1,60E-35	0,956720473	-1,317024556	1,087831832	3,769806569
A_64_P008161	Lingo3	1,31E-17	0,782780118	-1,188655789	-1,002070283	-1,884270319
A_64_P008177	Tchh	1,05E-12	0,691838949	-1,204852074	1,149053435	1,650230099
A_64_P008747	Stambpl1	1,21E-13	0,734258895	-1,173984494	-1,135615385	1,057259424
A_64_P008879	Thpo	4,70E-14	0,706444159	-1,026918619	-1,094274261	1,323616976
A_64_P009355	Reep4	9,78E-15	0,758926133	1,126441194	1,183369691	1,428391256
A_64_P009565	Vmo1	2,01E-24	0,913639105	1,024393852	-1,421015706	-1,000765735
A_64_P009718	Btbd17	6,71E-36	0,969565118	1,304733864	1,311376585	1,14233239
A_64_P009803	Btg2	1,49E-24	0,945382706	1,648172449	2,932796359	1,161893338
A_64_P009857	Tbx1	1,30E-17	0,813192721	-1,557151915	-1,483474595	1,21183632
A_64_P009897	Ppp2r1b	2,43E-22	0,895521942	-1,011114262	-1,810368102	-1,031779795
A_64_P009996	Tac1	2,07E-15	0,772945611	-1,000957473	-1,247782069	1,975275601
A_64_P009999	Tac1	2,64E-11	0,651621671	1,048084375	-1,207551295	2,59835251
A_64_P010298	Sh2d3c	9,29E-19	0,854869571	-1,025060726	1,612862398	-1,06164336
A_64_P010393	Clcn3	1,91E-14	0,694623001	-1,114822833	1,013281638	1,282719712
A_64_P010960		4,51E-15	0,781524476	-1,413892714	-1,082804058	1,146862751
A_64_P011045	Hrh3	2,89E-13	0,70652162	-1,159595114	-1,099071368	-1,184790912
A_64_P011070	Asic4	5,95E-24	0,883415057	1,146955433	1,008039281	1,408315234
A_64_P011324	Ahrr	1,46E-22	0,843874346	1,242274293	-1,127500271	1,252216573
A_64_P011489	Gdf15	1,08E-09	0,621019743	1,391180427	3,066934871	2,502051006
A_64_P011654	Zfp296	2,33E-18	0,825098792	-1,137201841	-1,278585342	-1,249616666
A_64_P011894	Xbp1	2,24E-16	0,717601796	1,01738089	1,085722163	2,11384564
A_64_P011959	Slc2a2	1,13E-23	0,88058169	-2,151790913	-1,591511281	1,058070017
A_64_P011979		6,38E-12	0,669956291	1,132275078	1,320000906	1,168008101
A_64_P012165		6,27E-24	0,872520445	-1,138598678	-1,104435924	3,609022156
A_64_P012166	LOC108349295	1,67E-22	0,86795642	-1,820801309	1,2996308	3,391370725
A_64_P012198	Agrp	3,52E-13	0,72306365	1,060875612	-1,46458331	-1,090676837
A_64_P012325	Slc35d1	3,60E-20	0,838602477	-1,096982188	-1,331731971	-1,729345825
A_64_P012467	LOC679711	5,88E-18	0,804635734	-1,449738958	1,301047586	3,840684617
A_64_P012471	LOC363324	1,30E-27	0,922357832	-1,294320821	1,650342612	4,200351318
A_64_P012477	LOC363337	1,03E-20	0,845957181	-1,279170444	-1,072026701	3,423375865
A_64_P012846	LOC108349386	4,57E-28	0,931987964	-1,272534606	-1,056908639	3,183736198
A_64_P012868	Tnxa-ps1	7,30E-11	0,659063682	-1,194450887	1,000329251	1,204461592
A_64_P012902	Sdf2l1	6,30E-22	0,849113724	1,176492259	1,054284258	3,636263632
A_64_P012947	Dapk1	3,59E-10	0,615109735	1,156102478	1,054439948	1,350327882
A_64_P013402	Rab33a	3,20E-17	0,820192329	-1,19941904	-1,101175037	-1,905281147
A_64_P013753	LOC108348296	8,89E-29	0,929868614	-1,322932598	-1,146323669	3,2984362
A_64_P014159	Plpp5	4,01E-10	0,613463501	1,014901546	1,298380421	1,368830034
A_64_P014356	Map2k6	8,11E-17	0,826410798	1,421656579	1,732228776	2,525445135
A_64_P014504		7,27E-22	0,834622408	1,426301787	1,150585361	1,552244263
A_64_P015366	Mfsd4a	9,16E-18	0,785619778	-1,213608782	-1,092968387	-1,293746267
A_64_P015549	Lyl1	2,39E-13	0,72718632	1,040962653	-1,123807217	1,423221214
A_64_P016766		5,58E-14	0,758674546	1,302034431	-1,66728051	-1,654306439

A_64_P016951	LOC108348348	1,80E-24	0,897774414	-1,203789719	-1,038598394	3,179078851
A_64_P017243	Tbc1d8	7,64E-18	0,802714699	-1,46829612	1,02811414	-2,341465855
A_64_P017363	Slc30a3	3,23E-19	0,810372711	1,05754653	1,149228339	1,740142919
A_64_P017706	LOC102547811	4,34E-31	0,942688137	-1,224233857	1,13895953	3,967307987
A_64_P018053	LOC108348337	6,17E-28	0,908984812	1,3438645	1,613061104	3,235199322
A_64_P018427		3,48E-11	0,668823578	-1,563093089	-1,528946939	-1,676496882
A_64_P018633		1,75E-16	0,807780768	-1,056638073	1,098965571	1,771179992
A_64_P018761	Calca	4,61E-10	0,611412326	1,621987139	1,202250903	1,133186524
A_64_P018766	Calca	8,02E-25	0,909142511	1,520126242	-1,066305593	2,650411742
A_64_P018901	Rprml	4,33E-12	0,628720729	-1,322210572	-1,154574373	-1,330815099
A_64_P018907	Tnfrsf4	5,54E-18	0,819180894	-1,090572612	-1,322593146	1,051612518
A_64_P019016	LOC501396	3,20E-11	0,707231675	-1,092070731	-1,529698066	-1,10014506
A_64_P019081	LOC690490	6,48E-14	0,722665265	-1,205008915	-1,381807981	-1,119036768
A_64_P019130	Ccpg1	2,03E-17	0,795334103	-1,037662886	-1,026089162	-1,245353045
A_64_P019175		2,07E-24	0,905733367	-1,325679962	-1,017623368	3,660747837
A_64_P019200	RGD1560608	7,27E-11	0,63790576	-1,065303304	-1,506952476	2,682785817
A_64_P019401	Sdf2l1	1,02E-22	0,890320788	1,338303772	1,085984518	3,252804238
A_64_P019608	Prkca	1,67E-11	0,697220967	-1,345268027	-1,053500603	1,128273705
A_64_P019673	Arhgap20	3,50E-23	0,885557752	-1,071668184	-1,11016271	-1,535814209
A_64_P020154	Plxnb2	7,67E-13	0,695463468	1,097161357	-1,042222666	-1,212098212
A_64_P020248	Hist1h2bo	3,39E-16	0,755210738	1,05650619	-1,210932871	1,45393799
A_64_P020269	Hist1h2bh	1,26E-21	0,845228885	1,462756957	-1,0933979	1,531087358
A_64_P020431	LOC687560	2,31E-17	0,794355146	-1,48347298	-1,183371053	1,320680874
A_64_P020471	Lrrc3b	9,67E-20	0,832517867	-1,175902725	-1,150372802	-2,09741018
A_64_P020621	Dlk2	4,29E-10	0,612470419	-1,271084371	-1,116464372	-1,185704247
A_64_P020741	Rsph1	1,30E-11	0,660937087	-1,021273333	-1,114855168	1,096788995
A_64_P021141	Rgs1	4,41E-14	0,707124502	1,368303627	1,475310815	1,123045555
A_64_P021149	LOC108349347	1,13E-14	0,700368544	1,188736708	1,179551144	1,623892353
A_64_P021221		1,72E-29	0,940090103	-1,194873782	-1,122100636	3,145083456
A_64_P021241	Nradd	2,43E-10	0,620824386	1,239663094	1,178912141	1,014378048
A_64_P021521	Plekhh3	7,26E-13	0,651886667	1,001968685	-1,075142369	-1,217212876
A_64_P021631	Nxnl2	8,64E-12	0,666122601	-1,340545187	-1,496676134	-1,499194453
A_64_P021686	Epor	2,41E-11	0,673511922	-1,141472445	-1,457514565	1,445249847
A_64_P021845	Car7	2,47E-29	0,92651594	-1,26220924	-1,321616368	-2,771036665
A_64_P022015	Sstr2	2,56E-24	0,887029972	1,291355663	2,188585901	4,05156223
A_64_P022813	Exosc10	9,90E-16	0,74535753	1,081342628	1,050125421	1,201586488
A_64_P022907	LOC108349337	1,35E-28	0,935118543	-1,349308371	1,060999392	4,963934572
A_64_P023203	Wdr73	8,74E-12	0,619204829	-1,05610811	-1,077956899	-1,134988917
A_64_P023250	Ascl2	8,51E-19	0,803518275	-1,23334745	-1,141785821	1,022150505
A_64_P023381	Col6a5	1,57E-10	0,64862935	1,833544601	-1,235456873	-1,078671574
A_64_P023561	Cox10	9,03E-13	0,69357435	-1,037756516	-1,262703078	1,112402876
A_64_P024272		1,01E-10	0,609959469	-1,358604535	-1,449859398	-1,259446012
A_64_P024474	Stum	2,84E-18	0,83647863	1,068278229	-1,380373847	-1,29168152
A_64_P024841	Cryga	1,08E-26	0,888923517	-1,029667159	-1,203594349	-1,118360646
A_64_P025050	Ccl1	3,87E-16	0,850541594	2,043828113	1,046961652	1,429195919
A_64_P025098	Cebpg	2,05E-16	0,819834489	1,053153067	-1,080973142	1,223906578
A_64_P025118	Cebpb	3,07E-14	0,819479878	1,076692142	1,621287237	1,951458632
A_64_P025169	Phlda2	1,78E-18	0,826897445	-1,448001232	1,158796451	1,721051418
A_64_P025496	Maff	1,67E-10	0,686563268	1,052463303	1,855724829	1,026759368
A_64_P025535		1,43E-33	0,958335831	-1,150667126	1,067253715	3,605616607
A_64_P025618	Slc25a35	1,17E-12	0,709857273	1,052037784	-1,27167381	-1,233478614
A_64_P025664	Cfap57	2,46E-19	0,826546404	1,327792622	-1,488722512	-1,203678164
A_64_P025678	Tbc1d16	1,53E-18	0,851939912	1,31068966	-1,105872269	1,145968509
A_64_P026297	Insl6	2,61E-24	0,904885744	1,234393098	-1,226804446	1,778912192
A_64_P026943	Gck	3,29E-13	0,684577489	-1,071399112	-1,14629175	-1,667786066
A_64_P027263	RGD1584023	4,96E-11	0,701900274	-1,285318579	-1,717171541	1,210603813
A_64_P027565	Zfp451	6,97E-13	0,675691315	-1,337873452	-1,292574423	-1,016728744
A_64_P027911		4,24E-18	0,821032908	1,635461015	1,454762366	2,682788457
A_64_P028379		1,59E-12	0,706344701	1,476381174	1,14134493	1,821454984
A_64_P029077		5,18E-11	0,701352264	1,390068583	1,957136704	3,443922554
A_64_P029337	F8a1	1,65E-08	0,620535661	1,040618137	1,049343903	-1,199115211
A_64_P029476	Ptpn22	1,79E-23	0,855173373	-1,031209279	-1,384165825	-1,431505487
A_64_P029501	Tnnc1	2,40E-17	0,82220434	1,144546671	1,177353879	1,030306893
A_64_P029805	Junb	1,32E-21	0,868576206	1,45797522	1,933123046	1,313245372
A_64_P030180	Mmd	3,13E-15	0,752645527	-1,110932611	-1,050015229	-1,210339374
A_64_P030362	Ephb3	1,55E-21	0,856489752	1,099375002	1,167760858	2,228610569
A_64_P030464	Tnxb	9,12E-15	0,79003426	-1,132188065	1,062130496	1,258891782
A_64_P030534	Tpcn2	3,46E-27	0,926439896	1,214472809	1,050988633	1,419500837

A_64_P030644	Inhbb	1,29E-11	0,638705515	-1,026635177	-1,023011845	-1,538824464
A_64_P030834		6,19E-12	0,648384846	1,412096614	4,704334121	1,787544701
A_64_P031511		5,77E-21	0,871651749	-1,755981408	1,284402457	3,149001621
A_64_P032037	Uri1	1,56E-16	0,762093057	-1,064975026	-1,018273895	-1,183453589
A_64_P032127	Ttll7	4,24E-11	0,685651324	-1,322079037	-1,120927244	-1,070211094
A_64_P032148		4,14E-18	0,791770714	-1,242138461	1,045734272	2,919476503
A_64_P032257		9,44E-21	0,846479566	1,91155378	2,203044795	3,153017768
A_64_P032358	Mustn1	3,28E-15	0,811870887	1,202030681	-1,056799742	-1,662418386
A_64_P032574	Hk2	4,56E-11	0,621340636	1,327036958	1,245039537	1,687342793
A_64_P032806	RGD1308106	1,81E-12	0,704846971	-1,309326128	-1,259580284	-1,041632928
A_64_P032836	Rprm	1,42E-21	0,844577352	-1,218079358	-1,231055667	-2,307743531
A_64_P033559	LOC679711	1,34E-26	0,915158253	-1,274533585	1,371721584	2,924834717
A_64_P033569	Tep1	2,24E-09	0,609879611	1,034756377	-1,138487748	-1,221010968
A_64_P033795	Hgd	1,73E-20	0,866050272	2,02000085	-1,473198622	1,362397507
A_64_P033845	Lmo1	1,67E-23	0,897760515	-1,13568257	1,083838218	1,500108238
A_64_P033901	Pars2	3,98E-14	0,727714416	-1,06604571	-1,13788086	-1,342157398
A_64_P033979	Cdh22	1,25E-20	0,831689322	1,161679836	-1,124075053	-1,337886472
A_64_P034090	Adm2	1,05E-10	0,654216305	1,633842866	-1,217698155	3,627595764
A_64_P034155	Ccdc166	2,48E-29	0,94982725	1,044450992	-1,49132268	-1,234942577
A_64_P034541	Rnf225	2,20E-17	0,809366484	1,245499809	-1,672910884	2,620558944
A_64_P034684	Rpain	1,05E-09	0,621332606	1,1139835	1,006106718	-1,222477808
A_64_P035041	Zfp111	2,32E-18	0,796167436	-1,060548075	-1,111080872	-1,100635418
A_64_P035237	LOC363337	1,69E-13	0,692223795	-1,232753339	1,011776924	2,992772885
A_64_P035238		3,90E-29	0,93816837	-1,493017401	-1,039344263	2,666954293
A_64_P035719	Dnajb3	3,75E-19	0,809325802	1,243994227	1,008000965	1,310481194
A_64_P036083	LOC367516	6,36E-31	0,941852305	-1,294250217	-1,049243675	2,943850688
A_64_P036174	Slc25a47	7,70E-26	0,900854478	-1,102736165	-1,548080915	1,139762001
A_64_P036514	Chrm2	9,87E-15	0,74168803	-1,316690074	-1,361209443	-2,336645217
A_64_P036576	RGD1563667	1,39E-14	0,738307693	1,463791077	1,68472852	1,556599825
A_64_P036676	Klf6	1,27E-10	0,671536587	-1,239926669	-1,678936725	-1,236521882
A_64_P037245	Shisa2	8,57E-30	0,929349475	-1,05293006	-1,517276439	-1,153974545
A_64_P038625		1,89E-14	0,768810676	-1,03964403	-1,187708358	1,075554477
A_64_P038767	Aste1	5,98E-14	0,741444707	1,048958995	1,097750697	1,260077056
A_64_P038839		1,31E-11	0,717790094	1,143187328	-1,808836827	1,905197684
A_64_P038902		2,96E-17	0,820740995	-1,437955237	-1,062916882	2,723792873
A_64_P038926	Prodh1	3,28E-09	0,603956249	-1,187431541	-1,271054026	1,239900963
A_64_P038972	Ppfia4	6,26E-14	0,681293138	-1,024563488	1,098568182	-1,518576197
A_64_P039342	Arhgap8	1,13E-13	0,674438069	1,083372443	-1,016030098	1,752952921
A_64_P039809		1,11E-28	0,935609318	-1,441982734	-1,094244145	3,497271259
A_64_P039834		3,45E-26	0,894589508	-1,277491702	1,427996002	3,915190213
A_64_P040095		1,07E-13	0,735517546	-1,524929768	-1,01778003	2,425931896
A_64_P040317	LOC102549583	8,70E-12	0,643918449	1,010406517	1,717127433	2,364042775
A_64_P040320		2,11E-18	0,825765616	1,137126393	1,351002234	4,303407622
A_64_P040528	Zbtb22	1,91E-16	0,760327478	1,0528903	1,228093233	-1,055031102
A_64_P041205	LOC108349320	7,97E-19	0,803993611	-1,101847757	1,357968599	3,751906824
A_64_P041208		1,98E-19	0,813751679	1,277182154	-1,274864946	3,269770095
A_64_P041228	Igsf3	1,54E-10	0,603800134	1,273609577	1,065309292	1,467989409
A_64_P041996	Stac	4,59E-20	0,84949569	-1,157117812	1,008250373	-1,960766213
A_64_P042142	Prr35	6,88E-28	0,899355824	1,154594117	1,286577626	1,276267816
A_64_P042249	Acvr1c	8,24E-19	0,844208698	-1,024016903	-1,153438405	-2,24897278
A_64_P042278	Crem	6,48E-21	0,860348992	-1,195036203	1,920209591	3,160728158
A_64_P042283	Crem	6,31E-27	0,900924146	-1,161523387	1,852059541	2,970064599
A_64_P043033	Cbr3	9,66E-11	0,693596233	1,040061183	1,59326366	1,449730226
A_64_P043392	Megf11	8,18E-26	0,916843262	1,065106144	-1,080505827	1,928335327
A_64_P043421		4,06E-26	0,903190671	-1,054888327	-1,06989871	3,351392829
A_64_P043614	Slc39a5	2,85E-19	0,850548775	1,128265032	-1,146993058	1,746223352
A_64_P043776	Fat4	1,63E-16	0,821469821	-1,242326231	-1,023212287	1,186304222
A_64_P044087	Nap1l5	1,15E-10	0,631453313	-1,051151386	-1,170727884	1,191331505
A_64_P044101	Mgme1	8,31E-15	0,7433596	-1,170387795	-1,395627451	-1,166330533
A_64_P044310	Lgals7	8,23E-13	0,650313757	1,352292988	1,350398653	2,647839546
A_64_P044470	Mrpl50	3,42E-12	0,677675589	1,202071116	-1,201857174	-1,356876692
A_64_P044776	Itih4	3,29E-20	0,810406586	-1,003534176	-1,444370808	-1,629024498
A_64_P045122	Mesp1	1,14E-27	0,915240915	1,198632087	-1,249593263	-1,259293791
A_64_P045141	Spatc1	5,73E-18	0,78928297	2,08082545	1,311162283	1,730057155
A_64_P045305		5,01E-20	0,822892511	1,568711188	2,555928945	2,78311592
A_64_P045779	Plcl2	5,86E-25	0,883097179	-1,189445723	1,090886972	-1,343056502
A_64_P046205	LOC685125	1,45E-17	0,781983783	-1,076095859	-1,366605672	2,903390048
A_64_P046552	Sdcbp2	5,78E-25	0,910289906	1,097010854	-1,425796219	1,301556735

A_64_P046853	Il17ra	1,85E-16	0,777631235	1,154260637	-1,214903589	1,320820283
A_64_P046885	LOC363337	2,06E-22	0,877558882	-1,088669902	-1,183070617	3,025700104
A_64_P047131		3,33E-10	0,616218506	1,283853245	1,235351229	2,068387943
A_64_P047201	Tmem181	1,55E-16	0,762144722	1,171913608	1,073006853	1,351381912
A_64_P047386		1,46E-08	0,622422426	-1,194932438	-1,677918357	-1,476386172
A_64_P047445	Tnfaip8	3,69E-20	0,824875692	1,097861277	-1,245519559	-1,852124911
A_64_P047774		1,23E-10	0,607071514	1,038927758	-1,062594603	-1,396086701
A_64_P048004	Bfsp2	2,81E-07	0,631321851	1,151198167	1,12368428	-1,220316935
A_64_P048033	Agpat3	5,85E-14	0,704048984	1,213194494	1,060029328	1,211393269
A_64_P048043	Krt7	1,63E-26	0,906433171	1,080620293	1,211915133	2,798114501
A_64_P048055	Alkal2	1,68E-19	0,841842333	-1,559404335	-1,384201622	-2,296495633
A_64_P048481	Dmrtc1b	3,79E-25	0,894794042	-1,155700851	1,164511801	-1,00963393
A_64_P048705	LOC100363225	3,35E-24	0,903957109	-1,224778413	-1,101333917	2,827348262
A_64_P048712		1,45E-11	0,612195346	1,285274738	1,07655823	3,35596735
A_64_P048765	Cbs	3,76E-11	0,738367648	-1,173913502	1,298111709	1,780012366
A_64_P048852		5,35E-10	0,651864183	-1,098282643	-1,179437999	-1,314406983
A_64_P048897	Tmem60	3,09E-16	0,773297062	1,021398234	1,018727341	-1,1917127
A_64_P049153		9,80E-18	0,78508355	-1,140053051	-1,045824926	2,865910587
A_64_P049808	Nptx2	8,40E-27	0,889926847	1,194633182	1,338055428	1,1186413
A_64_P049897		4,14E-30	0,916182869	1,305487686	1,44122334	3,554099324
A_64_P049948	Dkk1	2,05E-20	0,82860107	1,028567447	-3,200476222	-2,665441465
A_64_P050030	Nptxr	7,52E-10	0,626335299	1,741944443	1,367476495	1,628295002
A_64_P050052	Pde9a	1,75E-26	0,921675356	-1,040845853	1,06357659	1,405898801
A_64_P050099		9,38E-25	0,908589895	1,092876002	1,096347184	-2,386652213
A_64_P050171	Sema4c	4,86E-17	0,80346452	1,190553292	1,660806943	1,052116805
A_64_P050430	LOC108348293	3,84E-15	0,711761186	-1,194738923	1,021609759	2,426063816
A_64_P050994	Sema4a	1,41E-21	0,830666738	-1,010020783	-1,047928932	1,297472805
A_64_P051082	Wfdc6a	1,99E-17	0,761417836	1,037337454	-1,037629872	1,937862354
A_64_P051125	Bpifb3	7,97E-12	0,62047621	1,446623918	1,098084457	1,746028805
A_64_P051139		1,72E-16	0,778251143	-1,02148779	-1,161653918	-1,234195432
A_64_P051272	Zfp775	1,18E-14	0,699853731	1,024647466	-1,096163935	-1,351286034
A_64_P051507		1,44E-32	0,954449804	-1,294654951	-1,030198098	3,348889329
A_64_P051836		4,21E-18	0,791643775	-1,050552165	2,063391159	3,087161591
A_64_P052422	Otud3	7,18E-14	0,701801938	1,365303883	1,079351228	1,49627044
A_64_P052770	Cdh11	4,09E-15	0,78237378	-1,24320381	-1,068130648	1,676854834
A_64_P052882	Cdpf1	3,89E-13	0,703195852	1,052627331	1,00165462	-1,287789909
A_64_P052892	LOC108349386	7,02E-24	0,871991719	-1,17274474	-1,222280546	3,275242436
A_64_P053031	LOC501467	1,92E-12	0,639466899	1,601981914	2,592213327	2,914107907
A_64_P053392	Kiaa0408L	1,62E-14	0,696453106	1,062056774	-1,157326463	-1,17913436
A_64_P053461	Bag2	3,63E-22	0,884759581	1,069209279	1,046049549	-1,880688091
A_64_P053785	Adra2a	8,59E-14	0,719683358	1,309455716	1,572968418	2,172580299
A_64_P053979		7,09E-19	0,788330071	1,059781215	1,022893171	1,850019991
A_64_P054153	Nudt6	6,76E-25	0,909741906	-1,13590892	-1,121531763	-1,268162305
A_64_P054193	Rsrp1	3,73E-11	0,647014881	-1,138345707	-1,050233893	-1,161595174
A_64_P054197		3,43E-22	0,852434707	-1,259761599	1,335713209	2,827534839
A_64_P054441	Adra2c	5,46E-10	0,608862172	-1,154721773	1,597288403	1,763373319
A_64_P054626	Hcn4	6,60E-11	0,616102619	-1,148186724	-1,118109535	-1,57785934
A_64_P054765	Eml1	2,93E-17	0,792491885	1,182012088	1,046756647	1,309854623
A_64_P054856	Wdr25	2,80E-16	0,77412999	1,220679485	-1,109102976	1,37339308
A_64_P055112		1,65E-14	0,717529892	1,640227783	2,358701527	2,625406651
A_64_P055279	Tmem121	1,24E-10	0,630388127	1,017824857	1,189079183	-1,333820049
A_64_P055313	Disp3	3,97E-24	0,903319379	-1,255863617	-1,025866714	1,391184149
A_64_P055634	LOC100363225	7,30E-14	0,679527709	-1,051227739	1,441197492	3,825945174
A_64_P055699	Timm9	2,32E-21	0,876129468	-1,029629621	-1,21467212	-1,007492012
A_64_P056247	Msh5	5,64E-19	0,790057606	1,124216715	-1,082525305	1,803944308
A_64_P056426		7,39E-29	0,923456696	-1,327198867	1,018240405	2,650485228
A_64_P056427		1,56E-33	0,948694332	-1,256519621	1,098376753	3,909816232
A_64_P056648	Crlf1	6,24E-10	0,629081022	1,624969636	1,188813241	1,466028368
A_64_P057295		2,70E-19	0,811625804	-1,001061036	-1,405116168	-1,900274111
A_64_P057397		1,10E-12	0,710543664	-1,235937175	-1,099599856	-1,481249975
A_64_P058015	Gimap1	3,22E-16	0,788647615	1,119016516	-1,286631629	1,268927374
A_64_P058087	Rbp4	1,92E-18	0,861402225	1,063174627	-1,17811236	1,136866854
A_64_P058280	En1	3,14E-26	0,88461529	-1,028692149	1,000863188	1,272619436
A_64_P058336	Ucn	3,41E-22	0,875183724	-1,357705765	1,267097324	1,486729414
A_64_P058988		4,30E-17	0,77309383	1,402003062	1,301274539	3,492178008
A_64_P059046	Arrdc3	3,25E-11	0,626068131	1,878645075	1,013582759	1,328440185
A_64_P059120	Aen	8,26E-13	0,694607849	1,127061249	-1,12128204	1,153270278
A_64_P059192	Cdc42ep5	1,54E-09	0,615622588	1,392754898	1,160062967	1,105019215

A_64_P059456	Plcd3	1,12E-19	0,801898661	1,057068647	1,165754313	1,627483255
A_64_P059495	Ddit3	1,66E-19	0,874595564	1,227744213	1,231403403	1,983222577
A_64_P059580	Atp6v1e2	3,51E-13	0,660893146	-1,331006946	-1,429559472	1,030861011
A_64_P060094	Lrrc2	2,57E-14	0,749773262	1,039734494	1,25057951	-1,260406736
A_64_P060202	Luc7l3	1,49E-24	0,867515655	-1,22102005	-1,232589353	-1,149593413
A_64_P060302	LOC365085	1,23E-08	0,644610112	1,240701174	1,077879767	-1,07787166
A_64_P060303	Lsr	3,46E-11	0,625214302	1,171575022	1,281917822	1,351651195
A_64_P060319	Gsg1l	3,44E-21	0,852160841	1,388346397	-1,000537902	1,545327907
A_64_P060608		1,90E-24	0,888270089	1,131719189	1,218011518	3,009830201
A_64_P061291	Micall1	1,59E-12	0,724349046	1,123230699	1,301767634	1,346097525
A_64_P061323	Kcna10	3,21E-12	0,698227306	1,137507368	-1,291826249	2,604191425
A_64_P061590	LOC688126	2,67E-15	0,735894475	1,219869907	-2,633411178	1,288641713
A_64_P061740	Trpv1	3,52E-15	0,797959193	1,305937252	-1,894916189	1,192641133
A_64_P061765	Fam89a	7,92E-10	0,625570122	1,16309384	1,264835837	1,549209947
A_64_P061830	Fam84a	3,91E-14	0,686656271	1,010293394	-1,308647507	-1,611358416
A_64_P061835	Fam84a	2,03E-15	0,718263809	-1,09060957	-1,071399375	-1,641550462
A_64_P062048	Znf354b	1,67E-11	0,67818764	-1,163302897	-1,508143586	-1,078568795
A_64_P062223	Terb1	8,71E-22	0,880769653	-1,707997484	-1,289050844	-1,00766379
A_64_P062506		6,86E-28	0,930909909	-1,058754881	1,055057601	3,036987352
A_64_P062593	Chst5	1,26E-20	0,867689371	1,155768295	1,215857733	1,321346099
A_64_P062638	Ptger1	5,68E-24	0,861007486	1,199462655	1,037522188	1,554934597
A_64_P062771	Washc1	9,38E-15	0,723370189	1,064852716	1,076770412	-1,289928607
A_64_P062890	Oaz3	7,54E-18	0,817038035	1,32998289	-1,630131535	-1,006339014
A_64_P063035		5,10E-13	0,719046783	1,21403896	-1,338867099	1,25953677
A_64_P063047	Rpp25	5,39E-10	0,722614254	-1,049821246	-1,361828433	1,022702513
A_64_P063309	Dennd2a	1,57E-13	0,713238322	1,128738265	-1,006736989	1,379283834
A_64_P063636	RGD1562660	2,97E-11	0,627311826	-1,02197222	-1,011898903	2,539400946
A_64_P063644		1,09E-12	0,710649678	-1,111029913	-1,043458508	-1,166495765
A_64_P063993	Ttc4	5,39E-19	0,85799654	-1,231373589	-1,086088172	1,238187895
A_64_P064128		2,31E-18	0,796176766	1,273862507	1,682263618	1,793544698
A_64_P064129	Igsf3	5,42E-18	0,789714358	1,453856542	1,584961394	1,60078261
A_64_P064531	Npas4	6,04E-37	0,965604441	-1,02444512	-2,103091078	-2,973479852
A_64_P065032	P3h4	6,25E-10	0,669002395	1,389241141	-1,097153738	1,00616025
A_64_P065063	Fosl2	3,23E-12	0,678385535	-1,02113993	1,579974879	-1,040116101
A_64_P065102	Evpl	1,83E-19	0,841316455	-1,0404295	1,31404167	-1,067386374
A_64_P065131	LOC102555083	3,03E-12	0,679144153	-1,254518921	-1,076355603	-1,240495842
A_64_P065165		5,89E-20	0,821845142	-1,165214915	-1,054927191	2,966086353
A_64_P065301	LOC102554444	3,24E-20	0,862710149	-1,575693104	-1,174158059	3,096417912
A_64_P065629	LOC102552540	7,93E-29	0,930172759	-1,259160817	-1,09777764	3,260299273
A_64_P065868	Acvr1c	5,04E-29	0,931364524	-1,055184187	1,113771437	-1,980549831
A_64_P066544	Smim3	1,24E-10	0,607029104	1,207258586	1,103817631	1,519802883
A_64_P066577		6,13E-27	0,917641431	-1,219059768	1,018859762	2,922572472
A_64_P066621	Msc	4,25E-20	0,861253575	1,272001613	2,2526739	3,619436988
A_64_P066782	LOC108348347	1,80E-25	0,928167651	-1,236520545	-1,094251563	3,784470602
A_64_P067000	Prima1	9,86E-27	0,923393256	-1,300711235	-1,203792177	1,430792737
A_64_P067143	Spata31d1d	5,23E-12	0,650576173	-1,405642795	-1,314375156	-1,088553149
A_64_P067366	Cacna1h	2,24E-14	0,733560864	1,348676352	-1,077020364	2,603472197
A_64_P067447	Acot4	4,05E-22	0,851535284	-1,363139395	-1,573652124	-1,096862664
A_64_P067654	Aldh3a1	9,85E-13	0,711780211	1,293596638	-1,014561124	-1,099361285
A_64_P067659	Aldh1a1	4,39E-11	0,644807753	1,092996474	1,193813179	1,098505909
A_64_P067883	Trim47	4,45E-23	0,874322221	1,107836249	-1,209640287	1,08775617
A_64_P067908	Trim17	2,16E-34	0,957036291	-1,087324906	-1,478725412	1,16139055
A_64_P067915	Nudt8	5,63E-10	0,670435834	1,31462432	-1,004338431	-1,0326463
A_64_P068027	LOC363337	1,18E-14	0,757193507	-1,081714819	2,137310437	7,89209433
A_64_P068308	Msx2	1,59E-15	0,697451507	1,171981291	1,38257273	1,360894547
A_64_P068548	Arhgap22	5,36E-24	0,883866848	1,582191767	1,169520459	1,549109472
A_64_P069071	Lpar6	1,52E-08	0,601093345	1,109566338	-1,164314267	-1,176827382
A_64_P069081	RGD1310495	2,54E-10	0,681111588	-1,374651957	-1,464597126	1,392722568
A_64_P069211	Cdc42bpg	1,36E-09	0,617470588	1,269822345	-1,203653426	1,694170531
A_64_P069261	Arl4d	5,48E-11	0,641784946	-1,536423143	1,193611375	-2,283839078
A_64_P069374	Scn10a	3,24E-28	0,938869208	-1,064681676	-1,452246346	1,420597646
A_64_P069462		4,54E-25	0,873030815	-1,010604634	1,826666309	3,662851469
A_64_P069664	Eya2	1,42E-13	0,694206678	1,413785809	1,197586456	1,772654531
A_64_P069789	LOC363337	6,56E-23	0,84827438	-1,179489929	1,495100536	3,77268566
A_64_P069942	Prss56	2,67E-20	0,852578277	1,572261295	1,557672706	1,462029377
A_64_P070068	LOC501416	9,37E-23	0,870783748	-1,013972707	-1,194166771	2,337443025
A_64_P070628	Cfap57	5,94E-14	0,703883441	1,112419693	-1,648992572	-1,385405494
A_64_P070891	Cacng6	1,40E-12	0,688399269	1,121814259	-1,060397791	-2,476953586

A_64_P070937	LOC501346	3,43E-15	0,798174992	1,148474226	1,9033666	4,325192369
A_64_P071178	Mgat5b	2,27E-16	0,758798904	1,092778478	1,049441368	1,1489393
A_64_P071810	Gpr143	1,31E-08	0,603506634	-1,185593368	-1,009832773	1,182511898
A_64_P071983	Ppp1r3c	5,27E-10	0,65207486	-1,086585726	-1,125858681	1,856064054
A_64_P072227	Arrdc2	3,16E-12	0,749714612	1,700742114	1,235870492	-1,307522887
A_64_P072343	LOC501317	5,37E-27	0,918056731	-1,56278908	-1,044905255	4,172704337
A_64_P073129	Map3k12	1,58E-12	0,641987674	-1,102073291	1,005124884	-1,131631758
A_64_P073944	Sox11	1,15E-09	0,620014492	1,492614669	1,003380873	1,669785099
A_64_P074127	LOC685203	5,51E-28	0,924840347	-1,112021478	-1,34889513	-1,063475871
A_64_P074357		2,64E-23	0,876759026	-1,424175165	-1,121151784	2,629685192
A_64_P074440	Rbm24	1,82E-23	0,855092303	-1,003735711	-1,151223095	-2,048814545
A_64_P074490	Wipi1	5,35E-18	0,819422822	1,218410534	1,096545052	-1,283860275
A_64_P075008	Birc7	1,25E-23	0,880148773	1,286400314	1,103847544	1,057880744
A_64_P075087	Camk1g	5,37E-15	0,807989105	-1,098036468	1,403732289	1,794771089
A_64_P075357	Cyr61	2,92E-23	0,886349261	1,24584134	-1,415590328	-2,642691099
A_64_P075910	Ajap1	1,83E-14	0,735614874	-1,204339482	-1,252374275	-1,012925606
A_64_P076217	Bhlhe22	2,59E-20	0,827135438	1,15284631	-1,153735799	1,476467674
A_64_P076352	Hacl1	4,97E-12	0,673072582	1,098424403	-1,11786252	1,231089031
A_64_P076436		1,63E-26	0,914533957	-1,053543264	1,089961906	4,612485577
A_64_P076450	Pomc	5,42E-14	0,704876497	1,178268223	-1,161405817	1,148683227
A_64_P076555	Jph1	5,58E-15	0,779678788	-1,463112658	-1,280218756	-1,699296969
A_64_P076832	RGD1311447	9,47E-24	0,929122166	-1,332952435	-1,204301876	1,2372639
A_64_P076900	Nedd1	1,26E-11	0,700603248	1,347451289	1,012989472	1,514798859
A_64_P077240	RGD1562660	2,28E-27	0,927624521	-1,521718175	-1,038258098	2,754954516
A_64_P077242		1,93E-13	0,729443644	-1,807690511	-1,089063805	2,689173056
A_64_P077392		4,74E-14	0,789882183	1,717875877	1,294011761	1,021640668
A_64_P078230	RGD1312005	2,04E-13	0,690099615	-1,252533448	1,309128345	1,492157373
A_64_P078303	Angpt4	1,94E-17	0,810290381	1,281040334	1,931695373	1,880372778
A_64_P078894	Il23a	7,20E-28	0,908474381	1,185301263	-1,208442451	1,830366306
A_64_P079220	Gprin3	1,45E-22	0,868663831	1,276066414	1,248928282	1,257470949
A_64_P079408	Wnt9b	9,82E-31	0,92038665	-1,276713877	1,179261811	1,650752808
A_64_P079523		2,81E-21	0,853272474	1,077222755	1,08525809	1,503314605
A_64_P079553	Myh8	8,46E-11	0,695269386	1,500411785	-1,010372368	-1,04830705
A_64_P079666	Cyp4f5	9,76E-22	0,870078772	-1,105083038	-1,109284445	-1,26338959
A_64_P079731	Zfyve21	4,41E-16	0,770228091	-1,051439274	-1,124439489	-1,305234838
A_64_P079822		7,41E-16	0,765706944	1,002516096	-1,035323743	1,182839051
A_64_P080329	Paxx	3,07E-11	0,649604763	1,051749131	-1,063898995	-1,178748202
A_64_P080499		2,04E-13	0,690098339	-1,083613145	-2,567765295	1,349215502
A_64_P080509	Fut1	2,11E-21	0,854848323	2,30453997	-1,52726355	8,46599948
A_64_P080580		9,46E-22	0,880383957	-1,295253112	1,165072622	3,7449701
A_64_P080667		1,95E-14	0,734965778	1,209050018	1,008058763	1,901159218
A_64_P080697	Cand2	2,07E-23	0,854406495	1,17573462	1,045185208	1,219804717
A_64_P081069		3,15E-20	0,810691066	1,152817907	1,181885193	3,121666897
A_64_P081222	Ptrh2	8,25E-19	0,818509412	1,056371858	-1,136306788	-1,08720119
A_64_P081738	LOC108349423	1,67E-26	0,914440791	-1,248515966	-1,09655484	3,069825394
A_64_P081788	Olfml3	4,53E-13	0,701480962	1,32623438	-1,007404115	1,205408547
A_64_P082386	LOC102553270	1,70E-09	0,654965418	-1,456832003	-1,227203205	-1,049458444
A_64_P082693	Egr1	1,15E-25	0,935547258	2,274928496	3,609367641	-1,262431409
A_64_P083329	Rmt1	3,05E-21	0,840152781	1,399778884	-1,588888866	1,957316826
A_64_P083610	Dbh	2,25E-28	0,903299565	1,358789618	1,032849314	2,37045877
A_64_P083944	Samd11	4,07E-13	0,702696285	-1,027703054	-1,005722883	-1,222967699
A_64_P083978	Zfp68	4,01E-20	0,871983576	-1,229354027	-1,111457628	-1,16624856
A_64_P084058	LOC685183	3,10E-27	0,919748315	-1,319254093	-1,325407042	3,495381438
A_64_P084059	LOC685183	8,02E-30	0,941839758	-1,342398734	-1,097477073	3,698372397
A_64_P084473	Pear1	1,87E-16	0,760518042	1,223843654	1,17419231	1,188314656
A_64_P084538	Tjp3	1,03E-23	0,870205378	1,045494003	1,008372677	1,381329406
A_64_P084851	Rcan2	8,73E-13	0,693956726	-1,070926917	-1,103766825	-1,519962394
A_64_P085004	Kiss1r	4,66E-25	0,902905464	1,076808154	-1,010788881	1,282637062
A_64_P085058		8,57E-12	0,686455169	1,141204612	1,080510738	2,089765643
A_64_P085650	Slc22a14	1,69E-23	0,878775338	1,305858248	-1,458617215	1,597516907
A_64_P085670	LOC108348910	1,51E-13	0,713635165	-1,315679736	-1,131242479	3,21234891
A_64_P085671		7,44E-20	0,868791872	-1,260688061	-1,092017821	2,377183875
A_64_P086041	Syndig1	1,22E-14	0,699488097	-1,138569222	-1,150024379	-1,553578693
A_64_P086404	Polr2h	3,75E-09	0,601873132	-1,026118843	-1,041948503	-1,089505478
A_64_P086445	Nr2c2ap	3,70E-13	0,683197713	1,15530738	-1,013141569	-1,223311242
A_64_P086784	Runx1t1	9,06E-15	0,759637827	-1,019639713	-1,11340376	-1,345743447
A_64_P086979	Pdzrn4	1,02E-12	0,620424712	1,143797708	1,057505632	1,573678242
A_64_P087014	Slc18a1	9,44E-20	0,818736979	-1,31831849	-1,218592271	-1,085647614

A_64_P087039		1,11E-22	0,880412924	-1,263965136	1,035632618	2,993073278
A_64_P087040		1,09E-29	0,935241312	-1,222755827	1,147366825	3,310276667
A_64_P087233	Pias4	6,60E-11	0,660403043	1,06121096	-1,072118653	-1,192668472
A_64_P087380	Efna3	6,70E-25	0,882523927	1,021660383	1,002453884	1,708735269
A_64_P087424	F2rl1	2,57E-11	0,651999303	1,172880608	1,586863583	3,309335066
A_64_P087801	St14	1,09E-14	0,740759993	1,23345232	-1,354634502	-1,875707968
A_64_P087906	Abtb2	6,25E-22	0,835509713	-1,136812222	1,103389307	-2,095490034
A_64_P088382	Angptl4	6,78E-16	0,823697943	-1,13101049	-1,645436213	-3,457448351
A_64_P088392	Hbb-b1	7,98E-09	0,734603203	-2,155859113	1,39306232	-1,505902755
A_64_P088393	Hbb-b1	5,21E-09	0,638425709	-2,27860468	1,629283535	-1,500123595
A_64_P088397	Sytl1	1,77E-22	0,912687538	1,152697053	-1,105669242	-1,097147679
A_64_P088995	LOC685668	2,13E-19	0,840413303	-1,150462533	-1,036846086	-1,061846709
A_64_P089016	LOC108349297	1,11E-25	0,908064093	-1,215244552	-1,111418499	3,28636495
A_64_P089040	Ovol1	2,72E-20	0,883493769	-1,471070716	-1,099463759	2,091610764
A_64_P089400	Hpse	6,60E-13	0,697187499	-1,2359717	-1,365816597	-1,23380318
A_64_P089475		2,26E-15	0,717193487	1,114760839	1,114080341	1,119321036
A_64_P090354	Syne3	9,22E-12	0,665305814	1,001437892	1,036383894	1,54051988
A_64_P090449		3,35E-13	0,704881567	-1,366807179	1,013146007	2,890080344
A_64_P090866	Sstr1	4,91E-19	0,791093809	-1,114676474	-1,120913725	1,357953708
A_64_P091208		1,79E-17	0,796306017	1,148695727	-1,297979133	1,251049783
A_64_P091618	Map3k14	1,81E-22	0,842643533	1,003094621	1,437642556	1,593517143
A_64_P091728	Slc22a1	8,33E-30	0,914060291	-1,008463872	-1,108136928	2,026878627
A_64_P091940	Gal	1,19E-28	0,905467452	1,067543827	1,223715028	3,424381887
A_64_P091953	Spink4	8,86E-15	0,759845218	1,077245278	-1,344600238	1,535060589
A_64_P092005	Stat2	1,96E-15	0,756980127	1,16278481	-1,070236557	1,210602412
A_64_P092067	LOC100363225	7,42E-19	0,804506018	-1,20288268	1,075324502	2,666102968
A_64_P092534	Sspo	1,11E-24	0,915643325	1,096657413	-1,165936769	1,736937328
A_64_P092744	Wnt11	1,19E-14	0,720895342	1,436343292	1,202017583	1,811115721
A_64_P093026	Anks6	4,86E-12	0,693315995	-1,185247605	-1,278110845	1,062410495
A_64_P093044		5,29E-18	0,844407699	1,336613583	-1,203146301	1,882383918
A_64_P093389		6,39E-15	0,762856755	-1,500308745	-1,001990792	3,586807203
A_64_P093452	RGD1305464	9,42E-22	0,846877533	1,116693738	1,123210471	1,862553582
A_64_P093522	Dtna	2,59E-12	0,700745232	-1,077771117	-1,162688926	-1,235067173
A_64_P093599	Abcc4	2,16E-11	0,60659399	1,099914275	1,122396818	2,583870676
A_64_P094515	LOC100360619	1,87E-17	0,823946711	1,026332197	-1,145324144	1,124623376
A_64_P094722		2,72E-18	0,810199864	1,108464018	1,192352666	1,747839849
A_64_P095005	Mmd2	2,72E-13	0,774060842	-1,203143126	-1,056034634	1,049154371
A_64_P095266		7,67E-14	0,720888951	-1,005940706	1,590263463	1,8432914
A_64_P095900	Rgs12	4,03E-12	0,653928835	1,039713786	-1,057585155	1,266359103
A_64_P096041		3,48E-26	0,894550562	-1,25771689	1,705022905	3,496721753
A_64_P096083		1,24E-12	0,689852935	1,018076844	1,206470274	1,343858491
A_64_P096748		1,16E-18	0,829713385	1,895902715	2,434736965	2,900127042
A_64_P096782	Ier5l	6,49E-16	0,782876826	1,607060149	1,30245985	1,136581368
A_64_P096787	Plpp4	4,49E-15	0,781557368	1,087922442	1,001819421	1,21640741
A_64_P097078	Pdp2	2,88E-19	0,795063155	-1,00067432	-1,310938894	-1,43534983
A_64_P097598	Mtus1	3,60E-12	0,696882745	1,119135152	1,004573563	-1,656698497
A_64_P097754		2,22E-15	0,737680357	-1,075868423	-1,014681599	2,255062783
A_64_P097857	Fam196a	4,24E-30	0,931176629	-1,220544842	1,136056499	1,562970881
A_64_P097974	St3gal5	8,92E-12	0,665714717	1,116031607	1,128458498	1,088762113
A_64_P098081		2,26E-29	0,933416577	-1,023046173	-1,274915929	1,367268801
A_64_P098255	Gadd45g	1,29E-29	0,928268591	1,441887288	1,29780026	1,182878538
A_64_P098698	Nkpd1	9,21E-20	0,857001306	-1,116457652	-1,312106596	-1,060197835
A_64_P098926	Ubap1l	2,11E-13	0,667056251	-1,471290063	-1,359820967	-1,20082074
A_64_P099055	Lsmem2	1,77E-11	0,65691802	-1,06779019	1,292265275	-3,172707452
A_64_P099151		1,43E-10	0,628400067	1,546670757	1,158960583	-1,176470169
A_64_P099583		5,01E-19	0,807301564	-1,044303967	-1,134161124	2,872278616
A_64_P099624	Lcn12	6,87E-10	0,605412841	1,101811414	-1,166452014	-1,351591779
A_64_P099923	Dap	7,04E-12	0,724857117	1,331719745	1,126649528	-1,050670844
A_64_P100008	Ptpro	1,60E-13	0,692867786	1,123562925	1,019774282	1,149635407
A_64_P100158	LOC102549583	2,26E-22	0,86647366	1,412416852	2,140792208	3,411297788
A_64_P100208	LOC363337	2,87E-13	0,66331901	-1,220928334	2,509276323	4,468078398
A_64_P100318	Marc1	6,91E-14	0,721989896	-1,059569955	-1,030857002	1,586709369
A_64_P100346		2,15E-32	0,953736642	1,110105853	-1,130399456	1,004615542
A_64_P100447	Hoxd9	1,30E-11	0,700203942	1,256984326	1,44106413	3,17197412
A_64_P100516	Aoc3	1,89E-15	0,828412426	-1,138817904	-1,617819528	1,851710612
A_64_P100544	Itsn2	6,54E-11	0,660523398	-1,260859113	-1,197687688	1,252545247
A_64_P101553	Sec63	1,15E-12	0,710069849	-1,000930511	-1,093489699	1,215988648
A_64_P101618	Slc25a28	1,61E-14	0,77025553	-1,198058273	-1,169007042	-1,358688251

A_64_P101908	Hmcn2	1,83E-15	0,789154827	-1,288436726	-1,062672133	1,658485904
A_64_P102545	Tmod4	3,08E-11	0,670376935	1,060804691	-1,085390455	-1,183960433
A_64_P103716	Sco1	2,24E-12	0,682815342	-1,098337266	-1,001820501	1,04334401
A_64_P104029	Spdef	5,55E-18	0,772109583	-1,274581477	-1,488223288	-1,360770415
A_64_P104159	Kiaa0895l	6,45E-16	0,766920068	1,214878086	1,055791381	-1,152779633
A_64_P104583	Sstr2	3,92E-29	0,932011684	1,888399885	2,388890788	3,97792424
A_64_P105122	Smim5	1,25E-26	0,898426687	2,369484543	1,176062352	1,832394325
A_64_P105977	LOC108349320	5,13E-23	0,862337688	1,141468027	2,080838042	2,489095873
A_64_P106291	Tmem268	9,59E-18	0,767590692	-1,039794465	-1,208461624	1,331404362
A_64_P106490		1,53E-28	0,934812335	-1,318974296	1,086465064	2,546141856
A_64_P106853		3,82E-12	0,630412465	-1,541166103	-1,049197984	-1,135894188
A_64_P108009	Nrarp	9,58E-24	0,891070792	-1,097170327	1,358803011	1,535628946
A_64_P108424		5,35E-22	0,836424472	-1,068355841	1,257298451	3,650676166
A_64_P108459		2,00E-22	0,904583474	1,008400026	-1,013855916	4,822314356
A_64_P108881	Pde1c	1,73E-14	0,717037101	1,10361553	1,065919325	1,307176181
A_64_P109342		2,45E-09	0,608509429	1,050717724	2,796593838	-1,208642183
A_64_P109531	Tgfb2	5,21E-10	0,652234652	-1,062693384	-1,48882842	1,36499523
A_64_P109900	Lgals4	3,67E-23	0,875231444	1,390492295	-1,674156944	1,683592281
A_64_P110389	Fibcd1	1,08E-16	0,746514164	1,127142662	1,031795257	1,923692427
A_64_P110394	Fibcd1	3,03E-13	0,706002089	1,811210491	1,155267388	1,244120478
A_64_P110469	Fdxr	1,93E-12	0,704143658	-1,206990449	-1,027680802	1,035187219
A_64_P110544	Spdef	6,20E-14	0,801090847	-1,478677772	-1,296323482	-1,471247384
A_64_P110599	Plekhf1	1,52E-23	0,906428279	-1,072182803	-1,060633425	-2,041883167
A_64_P110798	Serinc2	7,57E-10	0,603937607	-1,10782655	-1,00931181	1,44288388
A_64_P110898		2,67E-11	0,672212587	1,423079737	1,948891265	-1,090187512
A_64_P111099	Trpc7	2,10E-16	0,776539262	-1,433641038	1,005340845	1,330440692
A_64_P111599	Bcor	4,44E-15	0,766167124	-1,433204805	-1,001089734	1,202609383
A_64_P111674	Olr206	7,09E-17	0,785491154	-1,230300495	-2,073986222	-1,558332724
A_64_P111776	Rab4a	8,89E-16	0,764092801	1,047167564	-1,071242159	-1,115243972
A_64_P111903	Mmp17	2,83E-12	0,679987176	1,045632751	-1,046002091	1,795182645
A_64_P112181	RGD1562660	8,83E-32	0,946043771	-1,170287912	-1,056131848	3,576866318
A_64_P112185		4,59E-18	0,833368359	1,070262118	-1,116838523	2,379133784
A_64_P112327	Ftsj1	3,48E-12	0,655825109	1,105792414	1,01945051	-1,092733505
A_64_P112406	Chac1	9,83E-15	0,758882557	1,061322685	1,342691392	2,601251947
A_64_P112767	Ralbp1	4,19E-13	0,702346666	1,072701014	1,186165034	-1,014275881
A_64_P113458		2,99E-14	0,711309154	1,652978523	-1,091657938	2,273811564
A_64_P113645	RGD1561551	2,12E-13	0,666978863	-1,018761772	-1,000532444	2,196059434
A_64_P113695	Myo7a	5,19E-20	0,822668027	1,188075459	1,027160992	1,280125274
A_64_P114396		7,09E-24	0,909206562	2,374753057	2,818498598	3,777008158
A_64_P114495	LOC363324	1,90E-19	0,828238741	1,000196677	1,047228485	2,524417973
A_64_P114838		4,96E-30	0,942910665	-1,454318043	-1,102609512	3,027145458
A_64_P115222	Pitpnm2	6,83E-17	0,827608605	-1,1272114	-1,151976921	-1,396083034
A_64_P115786	LOC299312	5,26E-11	0,619328791	1,08738132	1,022383802	1,442843136
A_64_P116298		2,68E-33	0,952736211	-1,361243509	1,164358421	4,016467214
A_64_P116299	LOC102557206	5,14E-11	0,663710321	-1,928112311	-1,04615094	2,831540078
A_64_P116301	Adam33	1,18E-09	0,640548093	1,173522839	-1,007515747	2,137691802
A_64_P116600	Rcan1	1,94E-22	0,896461393	1,058621016	1,223664881	1,275582518
A_64_P116606	Rcan1	1,44E-10	0,628291338	1,249099177	1,336918546	1,244027019
A_64_P116972	Crh	1,26E-10	0,606749063	-1,1692929	1,755960364	2,479322494
A_64_P117126	Slc25a19	1,12E-17	0,814220259	-1,014132916	-1,018025042	-1,59750135
A_64_P117468	Ky	1,58E-17	0,763391219	-1,005827011	-1,343019396	-1,400579639
A_64_P117726	Slu7	2,40E-08	0,614446395	-1,015830724	-1,082128128	-1,224309937
A_64_P117901	Trib3	3,59E-20	0,838620102	1,551692644	1,128505305	5,016738226
A_64_P118457	Cited4	3,15E-28	0,926420908	-1,042796033	-1,107120461	-3,572498734
A_64_P118698		1,93E-16	0,741135987	1,73903781	1,573536234	3,30079239
A_64_P119022	Bsnd	3,68E-32	0,929210749	1,201960179	-1,220983936	1,677439926
A_64_P119369	Baat	1,57E-21	0,856430231	-1,368556797	-1,376851736	-1,005941773
A_64_P119680	Alkal2	1,37E-12	0,688718302	-1,490285459	-1,551465074	-2,08337013
A_64_P119821	RGD1311084	1,97E-24	0,927518576	1,4750991	1,017888103	1,468457244
A_64_P120036		2,02E-12	0,684042969	-1,170573123	-1,554481277	-1,533508752
A_64_P120065	Jade1	5,33E-19	0,790478938	-1,167512333	-1,037946038	1,406437029
A_64_P120070	Insl6	1,41E-21	0,857021007	1,18510041	-1,101467242	1,622669065
A_64_P120342	Onecut1	2,45E-12	0,636294052	1,204662946	1,064589661	-1,278655074
A_64_P120445	Cacng6	8,37E-21	0,869776082	1,011518377	1,086011528	-2,433312987
A_64_P120564	Zfp90	9,16E-12	0,665378924	-1,068336846	-1,089027285	-1,327311948
A_64_P120634	Pgf	1,56E-23	0,879156384	1,28301888	1,076799772	1,552197784
A_64_P120640	Pgf	1,14E-21	0,85813963	1,20781554	1,651964822	1,516620091
A_64_P120679	Cck	1,62E-15	0,720567762	-1,034412969	1,287341898	2,420560074

A_64_P120881	Hils1	1,22E-15	0,743352137	-1,036913594	-1,562886445	-1,121822248
A_64_P120882	Hils1	4,50E-15	0,730766368	1,015637099	-1,775027099	-1,217948474
A_64_P121796	Perm1	2,01E-34	0,952461101	-1,2175246	-1,938688935	-10,75137147
A_64_P122238	Tuba4a	2,92E-13	0,7064118	1,081089441	-1,070339852	-1,86938659
A_64_P122247	Hcrtr1	2,60E-17	0,793421552	-1,047441129	1,164204485	2,408423334
A_64_P122624	Sphkap	9,93E-29	0,935890037	-1,170931054	1,015101739	-4,419252611
A_64_P122671	Avpi1	1,19E-15	0,723659929	1,058873158	-1,020430654	1,175548507
A_64_P122676	Crygs	1,51E-16	0,762384232	1,315929354	-1,768270859	1,588191192
A_64_P122944	Phf20l1	2,29E-19	0,827009308	-1,167243399	-1,002945777	1,336731324
A_64_P123118	Fosl1	6,11E-20	0,847840396	1,069162722	-1,346048695	-1,709764115
A_64_P123792		5,96E-12	0,62443539	1,275433071	1,388393783	1,079660584
A_64_P123949	Gk	2,03E-25	0,905927222	1,009053143	-1,060642458	-1,215883447
A_64_P124090	Ntsr1	2,03E-29	0,954842786	-1,185087481	-1,31941391	1,525183787
A_64_P124898	LOC363337	3,79E-18	0,821807074	-1,203562031	1,538837316	5,987092251
A_64_P125350	Kcnh6	9,43E-27	0,916284175	-1,091327387	-1,311439012	-1,032586757
A_64_P125715	Serinc4	7,89E-10	0,603312399	-1,044880055	-1,911250859	1,178276803
A_64_P125858	Socs5	1,39E-11	0,680494975	-1,044904047	-1,12421011	1,253657103
A_64_P125883	Socs3	2,18E-21	0,827967343	1,483759843	1,315832261	1,053465523
A_64_P126030	Slc22a7	8,44E-14	0,784795012	-1,447850277	-1,375841941	1,272922874
A_64_P126450	LOC100909409	1,73E-11	0,634762854	1,153032941	1,356611549	3,617678744
A_64_P126493	Crem	1,60E-15	0,720665008	-1,209246046	1,968152226	2,645109597
A_64_P126630	Gsc	4,40E-20	0,808417363	1,185304106	1,259126375	2,265433434
A_64_P126818	Tspan18	3,91E-21	0,838683327	1,39882975	1,111416297	1,429874473
A_64_P126955	Rybp	1,11E-12	0,710398095	1,005440702	1,261142017	1,474782712
A_64_P127327	Sys1	2,71E-14	0,749232571	-1,019043883	-1,164859117	-1,161652402
A_64_P127833	Ppfibp1	4,23E-11	0,666281799	-1,064996146	1,016860321	1,304137277
A_64_P128725	Mthfd2	3,11E-13	0,685214172	-1,004492805	1,313989767	4,331690182
A_64_P128833	Wnt3	1,59E-11	0,61089062	1,032288753	-1,044128396	1,355940901
A_64_P128874	MGC116197	4,47E-12	0,674387588	-1,105296914	-1,077194893	-1,083057201
A_64_P129563	Arid5a	9,33E-12	0,618312126	1,236640098	1,332594422	1,178592589
A_64_P129730	Tmem79	1,51E-18	0,84048715	1,045887755	-1,757335222	-1,072258015
A_64_P130025	Dll1	7,51E-14	0,721109771	1,351074679	1,777031346	1,766773624
A_64_P130239	Slitrk3	1,40E-09	0,617053559	1,006692485	-1,313880641	-2,110003453
A_64_P130380		1,67E-18	0,827311127	1,002001387	-1,212473741	-1,308298099
A_64_P130390	LOC681364	2,99E-19	0,83831524	1,122793744	1,172098082	3,191398136
A_64_P130551	Ptchd3	3,48E-14	0,709656844	-1,040032326	-1,90637248	1,977030325
A_64_P130873	Csrnp3	1,75E-19	0,814576283	1,210762166	1,421551543	1,151967568
A_64_P131051	Fos	7,66E-33	0,945574198	1,168165271	1,384031559	-1,355107214
A_64_P131122	Iqank1	8,39E-16	0,780716544	1,44227158	1,521347294	1,393604802
A_64_P131141		9,80E-16	0,763220363	-1,228918422	1,077580017	-1,067373989
A_64_P131151	Opn4	3,95E-15	0,767227623	1,008087509	-1,576385124	-1,117457374
A_64_P131409	Pdgfa	2,56E-11	0,629386286	1,129000934	1,025335108	-1,193329324
A_64_P131451	LOC102550991	1,17E-30	0,940488869	-1,177182948	2,061219834	3,871231813
A_64_P131477	Twist2	2,80E-11	0,650862426	1,819403218	1,812144575	2,988120728
A_64_P131791	Shroom3	5,20E-20	0,848778653	-1,068451169	-1,042139823	-1,129479793
A_64_P131834		1,16E-14	0,721219589	1,026724986	-1,03595529	3,839314648
A_64_P132044	Spocd1	8,96E-13	0,693660956	-1,012392419	-1,24768093	1,957019458
A_64_P132199	B3galt5	2,48E-24	0,887166797	1,50286075	1,291559662	1,415328065
A_64_P132327	Dnase1	1,41E-12	0,707750799	1,156948591	-1,012187502	-1,437896546
A_64_P132721	Oacyl	5,89E-29	0,916464263	-1,500873203	-1,338810239	-1,007723608
A_64_P132976	Gprc5c	8,28E-18	0,768806315	1,286406372	1,336096138	1,129785899
A_64_P132981	Gprc5c	8,98E-34	0,938009655	-1,022022466	1,621907894	1,080909022
A_64_P133096	Neurl2	1,72E-12	0,740274718	1,347985947	-1,115073878	-1,657695091
A_64_P133152	Neurod6	2,01E-10	0,684186644	-1,031015108	1,500355875	1,392924198
A_64_P133430	Mat2b	1,88E-10	0,6008753	-1,061260766	-1,324754259	-1,426753003
A_64_P133632	Card19	1,94E-19	0,82807898	1,066562471	1,101848384	1,273878921
A_64_P134263		9,38E-15	0,759315033	1,040242978	-1,151007186	3,203123891
A_64_P134445	Tmem179	9,07E-13	0,67252279	-1,116965418	-1,338442897	1,048328722
A_64_P134508	Sync	5,01E-15	0,708988736	-1,117373066	-1,139168476	-1,5950972
A_64_P134744	Hpn	2,13E-18	0,825690953	-1,264893509	1,761461937	1,849824881
A_64_P134748	Hpn	2,66E-27	0,904008157	1,111772652	1,201560257	1,79170485
A_64_P135152	Msantd1	1,40E-19	0,830185087	-1,10063316	-1,1323809	1,487355949
A_64_P135229	Nrbf2	6,24E-12	0,623803476	-1,274245369	-1,26630667	-1,000811429
A_64_P135474	Cxcl12	3,88E-24	0,862885822	1,157975589	1,079499112	1,478476385
A_64_P135502		1,15E-14	0,740198696	-1,029752058	-1,333661923	-1,747326681
A_64_P135843	Grb7	3,28E-30	0,931828003	-1,437683662	-1,308121975	1,485373762
A_64_P136178	LOC108349417	5,25E-23	0,86221938	-1,461487004	1,446002975	3,6050021
A_64_P136367	Cbx8	1,49E-09	0,637114942	1,169966037	1,18627069	1,176457646

A_64_P136382	Acvr1	1,59E-10	0,603323589	1,008655769	1,501458396	-1,035124142
A_64_P136394	Col20a1	1,76E-11	0,656931213	1,374734433	1,006063153	2,076317687
A_64_P136512	Ecel1	5,39E-15	0,764419578	1,276300707	1,337312101	1,861201881
A_64_P136676		1,14E-23	0,880549589	-1,311912972	-1,030255896	4,052018329
A_64_P137075	Col7a1	6,20E-18	0,788666211	1,227493042	1,166984363	2,017057848
A_64_P137461	Bspry	1,12E-12	0,744739134	1,429697818	1,091041665	1,124321287
A_64_P137784	Plvap	3,84E-27	0,911334354	1,237082812	-1,078273413	1,714255558
A_64_P138011	Adra1b	3,63E-21	0,851869344	-1,079015314	-1,196170835	2,183199849
A_64_P138127	RGD1562660	3,49E-24	0,87521761	-1,235713951	-1,070898594	2,756504201
A_64_P138216	Gch1	4,02E-14	0,761802708	-1,359742869	-1,273781858	-1,28449651
A_64_P138415	Cldn19	6,33E-17	0,786406354	1,027032242	1,169310484	1,057440267
A_64_P138430	Odf3b	1,14E-19	0,855787011	-1,181747146	-1,152740297	-1,563484482
A_64_P138575		1,95E-13	0,746419185	-1,331731648	1,032647152	-1,039425249
A_64_P138800	Myadml2	9,87E-28	0,915705662	-1,037128504	-1,562928803	-4,022999978
A_64_P139485	Plcd4	1,43E-08	0,60215902	-1,005647089	-1,026945856	-1,463617996
A_64_P139559		4,98E-14	0,705805099	1,572093474	2,411260554	2,23613745
A_64_P139802	Prph	2,06E-11	0,745081454	1,596753453	-1,025444153	1,628841756
A_64_P140000	Myo15b	2,11E-25	0,921015326	1,11300529	1,086003901	1,523029409
A_64_P140025	Tyrp1	6,08E-12	0,690618941	1,270032301	1,466546809	3,059579656
A_64_P140225		7,50E-25	0,892077596	-1,293996343	1,113448551	3,390197684
A_64_P140226		2,29E-18	0,779223494	-2,059806706	1,755209367	3,655916449
A_64_P140318	LOC108349340	1,57E-14	0,718069007	-1,349949632	-1,047897677	3,167961802
A_64_P140323	LOC108349386	3,69E-24	0,885477122	-1,300722202	-1,212503994	2,833707012
A_64_P140437	Cxcl2	6,68E-20	0,805529984	1,871982567	2,676598082	1,709209333
A_64_P140652	Ccdc84	1,18E-08	0,605298499	-1,171721986	-1,2075928	1,063866305
A_64_P140658	Anks1a	1,29E-11	0,661027898	-1,027413517	-1,146671718	1,208488186
A_64_P141727	Rhob	2,32E-15	0,716935684	1,424697746	1,596920774	1,284050239
A_64_P141962	LOC501297	1,79E-24	0,878224133	-1,049183424	1,945066257	3,550774644
A_64_P142625	Gprc5a	4,95E-24	0,873622214	1,563799476	6,193937691	20,63260275
A_64_P142776	Fgf5	2,38E-12	0,660619667	-1,027802916	1,600680927	1,101825685
A_64_P142804	Nudt13	1,96E-25	0,887685128	1,038170774	-1,460843682	-1,148439375
A_64_P142942	Scx	4,16E-29	0,925074452	-1,138147722	1,254595854	1,21801576
A_64_P142988	Tmprss9	1,99E-29	0,954877387	-1,02485746	-1,323636683	1,365181908
A_64_P143072	Aanat	1,14E-18	0,784681857	1,226981758	1,506632369	1,489994288
A_64_P143186	LOC108353713	5,70E-18	0,804874123	-1,033933924	-1,322024309	-1,293306447
A_64_P143480		8,47E-27	0,916625045	1,00286821	1,806883762	3,665239062
A_64_P144123	Olig2	3,03E-24	0,886315176	1,221306622	1,204377992	1,298412532
A_64_P144134	RGD1566029	3,76E-16	0,801912623	1,338570859	1,70298273	1,473919672
A_64_P144968	Calb2	7,22E-18	0,787487676	1,192466949	1,094595925	2,162205881
A_64_P145433	Ahr	1,56E-08	0,621429522	-1,328341079	-1,064533893	-1,202722172
A_64_P145828	LOC691712	5,34E-33	0,946301519	-1,272974077	1,038433213	3,022295449
A_64_P145829	LOC685324	2,57E-28	0,926989822	-1,126472848	1,167991599	2,953783528
A_64_P146243	Pou2f3	1,88E-21	0,855477914	-1,026762741	-1,850032506	-1,018289233
A_64_P146298	Rab20	1,73E-11	0,60972225	-1,217903933	1,168108957	-1,887772614
A_64_P146692	Slc22a1	7,14E-24	0,859864289	1,030208651	1,190269224	2,153262043
A_64_P146970	Cacng2	3,49E-17	0,805955506	-1,034783389	-1,151771908	1,492536973
A_64_P147094	Sept8	1,47E-21	0,830391635	1,048570243	-1,101793938	1,308053818
A_64_P147164	Tubg1	3,62E-13	0,703996965	1,039159121	-1,153313554	-1,249305032
A_64_P147190	Fdps	3,24E-11	0,648875176	1,179465868	-1,000938784	1,142493439
A_64_P147403	LOC100911864	1,20E-11	0,661954829	1,186090744	-1,191413412	-1,974397541
A_64_P147769	Gucy1b2	1,67E-17	0,780814829	1,280146623	1,377504121	4,097991239
A_64_P147898		2,48E-22	0,84086263	1,085624737	1,103715479	2,242257863
A_64_P148042	Mex3b	1,90E-22	0,87794395	-1,103599111	-1,401354694	-1,005018912
A_64_P148265		6,62E-15	0,726892774	-1,034859407	-1,214716184	-1,598172162
A_64_P148325		3,14E-10	0,638971203	-1,232941116	-1,175131877	1,212164805
A_64_P148535	Napepld	9,86E-13	0,746091641	-1,006186111	-1,297659242	-1,038935435
A_64_P149119	Rftn1	4,66E-13	0,657405591	-1,203382342	-1,080430206	-1,166284121
A_64_P149300	Cacnb4	1,56E-22	0,878848619	-1,215248356	-1,078760798	-1,482727264
A_64_P149461	Cdc42ep1	3,02E-11	0,627093722	1,293641853	1,252019802	1,426365903
A_64_P149689	RGD1305733	3,60E-09	0,623888327	-1,155997211	-1,136291205	-1,195937106
A_64_P150181	Rab23	5,98E-20	0,821742577	-1,166470426	-1,018113201	-1,403104567
A_64_P150326	LOC108348357	3,54E-20	0,825132216	-1,40930073	1,299879292	2,51182891
A_64_P150466	Vstm2l	2,81E-14	0,711959628	1,00942424	-1,261466872	1,322320229
A_64_P150691	Araf	5,14E-15	0,72944113	-1,099214317	-1,111047914	-1,091726677
A_64_P150814	Rnf19a	4,09E-15	0,78236148	-1,13072399	-1,071342907	-1,178493817
A_64_P151393	Mpp4	1,02E-13	0,697923809	-1,5676011	-1,259394932	1,034730557
A_64_P151448	Fancf	5,00E-12	0,651146353	-1,251365204	-1,19228596	-1,280879658
A_64_P151627	Trim16	8,64E-20	0,845817465	1,062181657	-1,042401116	1,252027491

A_64_P152252	Fscn2	1,31E-26	0,907196407	1,080080663	-1,295063791	-1,591046499
A_64_P152800		3,02E-23	0,886203979	-1,133734133	1,284227357	3,286268822
A_64_P152888	Nphp4	2,89E-19	0,795047191	-1,179188827	1,13264451	1,16410875
A_64_P152993	Sipa1l1	7,59E-14	0,720995582	1,070308544	-1,019231683	1,544459843
A_64_P153002	Gpr20	2,19E-26	0,913567671	1,705699697	1,905745325	1,573011589
A_64_P153062	Tspan9	1,20E-23	0,890135748	1,105376375	1,312893835	1,30976801
A_64_P153282	Fpr2l	7,24E-17	0,800438915	-1,297742455	-2,342089488	-1,080711619
A_64_P153442	Zfp717	2,80E-17	0,758474691	-1,083870098	1,038159616	1,333048555
A_64_P153533	Ubtd1	1,60E-14	0,717882746	-1,03444314	-1,119610382	1,264993262
A_64_P153694	LOC108348293	2,33E-18	0,837751856	-1,194977155	-1,035495541	1,823471688
A_64_P154430		1,45E-11	0,698898255	1,399609459	1,036888662	1,060774029
A_64_P154469	Ccdc166	1,12E-25	0,908045082	-1,026739362	-1,703722051	-1,250218296
A_64_P154952	Nr4a2	8,01E-15	0,760781268	1,757917493	2,027490845	1,021903566
A_64_P155059	Slc22a12	1,31E-13	0,672747853	1,528792314	-1,103529533	2,331679715
A_64_P155201	LOC690126	1,79E-26	0,906101412	-1,183504835	-1,497631298	1,366189018
A_64_P155393	Ccnd1	1,78E-12	0,685550633	1,494988244	-1,096593981	-1,047607396
A_64_P155463		5,43E-27	0,91017949	-1,392469087	1,171980616	3,984002354
A_64_P155983	Vegfa	8,19E-12	0,723151336	-1,699104635	-1,175452442	-2,156217243
A_64_P156263	Lmcd1	9,71E-17	0,782946717	1,095205433	1,080230427	-1,336668144
A_64_P156303	Cyb5d2	1,34E-19	0,830438436	1,074945251	-1,223115665	-1,106279403
A_64_P156662		1,25E-18	0,815687745	1,034670018	-1,19144826	1,179806122
A_64_P157296	Efcab8	7,14E-16	0,78207109	1,147960937	1,011027956	1,548332694
A_64_P157405	Reep1	3,61E-16	0,754622949	-1,062125406	-1,126032102	-1,304539059
A_64_P157604	Cpne7	6,25E-11	0,639984224	-1,047227618	-1,196149984	-1,168197761
A_64_P158103	LOC103690708	2,91E-12	0,65807304	1,312642928	1,30766591	4,216842682
A_64_P158778	Ocm2	3,67E-10	0,636759568	1,843742908	1,733874021	2,417589903
A_64_P158903	Has2	1,15E-16	0,836111691	1,165915571	4,333384193	2,94372071
A_64_P158945	Tcap	1,09E-22	0,880493391	-1,094827767	-1,468623215	-3,774406934
A_64_P159275	Inip	3,75E-17	0,805413312	-1,082356527	-1,121002546	1,153220749
A_64_P159543	Gem	4,03E-12	0,653930385	1,020918974	4,044731119	4,345039222
A_64_P159858	Chchd6	5,03E-17	0,771777647	1,187762923	1,034741667	1,204616562
A_64_P160008	Nxnl2	1,90E-14	0,768733953	-1,252047411	-1,562737042	-1,293038472
A_64_P160091	Gpr62	5,39E-22	0,872988534	-1,03225624	-1,028025963	-1,463761261
A_64_P160373		1,46E-15	0,759663231	1,270416777	-1,117101844	-1,621418827
A_64_P161265	Zfand2b	5,04E-16	0,825836404	1,197133421	1,025365187	-1,164159199
A_64_P161630	Amigo3	3,50E-22	0,852322588	-1,148942923	1,373293794	1,55594946
A_64_P161635	RGD1310507	8,06E-21	0,819723295	1,063891879	-1,327741386	1,346922554
A_64_P162002		2,20E-09	0,610182662	-1,650513633	-2,256539183	1,238232319
A_64_P162125		1,24E-23	0,880176116	1,012134276	-1,806080854	1,012414096
A_64_P162272		1,57E-13	0,670549983	1,005954512	1,146942547	-1,491581698
A_64_P162599	Psat1	3,93E-10	0,613753741	1,121925598	-1,252634046	1,66307622
A_64_P162700	Kank3	2,07E-17	0,795187491	1,094282938	-1,148105689	-1,266031301
A_64_P162760		6,48E-09	0,654296236	1,419291641	1,309123167	1,064707712
A_64_P162940	Crxos1	1,04E-18	0,854202024	-1,012502591	-3,009246315	1,299661123
A_64_P163100	Ripply2	7,30E-16	0,781887953	1,056045628	-1,14324873	1,020523745
A_64_P163165		3,81E-15	0,750805746	1,033106582	-1,622285493	-1,312954026
A_64_P163244	Myom1	5,99E-10	0,607481811	-1,124140998	1,019442504	-1,350913602
A_64_P163564	Elfn2	2,39E-21	0,827401638	1,187673621	-1,008675013	1,483729297
A_64_P163698	Onecut2	9,15E-14	0,78407322	-1,013313875	-1,037991234	1,502764026
A_64_P163822	Cks2	6,20E-12	0,670312376	-1,044599139	-1,151351615	-1,411709928
A_64_P164081		3,87E-32	0,936047894	-1,292207497	1,072722824	2,864474113
A_64_P164215		8,77E-12	0,704885628	-1,290531378	-1,006479062	1,231716736
A_64_P164240		1,47E-12	0,687874428	-1,092022241	-1,124911297	-1,117043684
A_64_P164351		6,37E-16	0,729786965	1,107483604	1,100172335	1,319301133
A_64_P164547	LOC108349417	1,29E-24	0,899059413	1,395916514	1,968080988	4,132511402
A_64_P164682	Etl4	4,15E-12	0,675293632	-1,444176288	-1,07442789	-1,515687664
A_64_P164797	Ehd1	2,71E-14	0,6907587	1,396339379	1,223731415	1,245544443
A_64_P165015	Shcbp1l	4,17E-12	0,653487325	1,519568699	-1,400542147	-1,043673316
A_64_P165085	Cdkn1c	2,67E-16	0,757347856	1,406351936	1,143572416	1,741398034
A_64_P165521	Bpifb2	9,94E-11	0,610224765	1,08756742	-1,144885584	1,413970381
A_64_P165913	Syt6	1,34E-27	0,929095258	1,105378456	1,291185757	1,598818975
A_64_P166385	Kifc1	5,54E-12	0,691742707	1,023043291	-1,19496916	-1,458924368
A_64_P204094	RGD1308134	1,25E-12	0,689761351	1,154339228	1,097361366	1,043080238
A_64_P237784	Rbm24	8,63E-30	0,947020639	-1,083592325	-1,585649465	-1,885397082
A_64_P246030	Pfkfb1	1,81E-10	0,646684508	1,408895543	-1,195510872	1,052815037
A_64_P247541	Pcdh7	1,07E-19	0,817907768	-1,073906537	-1,098854337	-1,429408131
A_64_P264316	Mfsd5	4,15E-12	0,675312803	-1,045932696	-1,215495608	-1,284970608
A_64_P267124	Rbfox3	2,46E-13	0,687942067	-1,288555252	-1,168438435	1,502129506

A_64_P267897	Efcab6	2,28E-13	0,709142539	1,298344162	1,434882926	2,257627083
A_64_P281840	Nrg1	3,28E-11	0,706930438	-1,000997589	-1,144087148	-1,319716916
A_64_P327151	Rxrb	6,50E-10	0,628482816	1,268378766	1,076484674	-1,125042561
A_64_P370146	Nrip1	7,78E-10	0,60351711	1,287569404	-1,112067231	-2,241903927
A_64_P393739	LOC108348277	1,28E-13	0,715422798	-1,236484453	-1,23092639	-1,450395958

Supplementary Table 3. Lists of genes with significant temporal expression changes in CGNs during rescue by IGF1 (K5 + IGF1 vs K5).

ProbeName	GeneSymbol.x	p-value	R-squared	K5_IGF_vs_K5_05h	K5_IGF_vs_K5_1h	K5_IGF_vs_K5_3h
A_42_P453976	Ifitm6	2,45E-18	0,849122394	-1,184233122	1,312117444	-1,632047504
A_42_P457003	Pdgfra	4,38E-13	0,658164838	1,028064193	-1,059881399	-1,364515195
A_42_P457692	Zfand2a	6,91E-13	0,696657286	-1,130032441	1,040810765	1,336313412
A_42_P459431	Bphl	6,18E-10	0,607016283	1,095625778	-1,273103578	-1,024250467
A_42_P459855	RGD1562618	2,94E-08	0,630994556	1,317893153	1,080438157	1,417795513
A_42_P461144	Skida1	1,84E-13	0,668711645	1,047776805	-1,071184097	-1,219271625
A_42_P462015	Stn1	1,03E-24	0,880649895	1,028513664	1,245059287	-1,10401127
A_42_P462257	Rprml	2,45E-31	0,953960826	1,038613152	1,282105748	1,207794744
A_42_P463998	Abcc6	9,33E-16	0,763663563	1,261748665	-1,037245831	-1,319987713
A_42_P470864	Acot13	1,20E-12	0,61826487	1,127851089	-1,118384521	-1,258774275
A_42_P471507	Unc50	6,25E-17	0,769956405	1,030556877	-1,131824676	-1,262239486
A_42_P472375	Pdk2	1,36E-10	0,650600208	1,006494697	1,207032155	-1,595552478
A_42_P472699	Amn	2,17E-21	0,854700388	1,142903975	-1,217584969	1,635451924
A_42_P473425	Unc13d	5,48E-19	0,86832868	2,102066922	-1,07156087	1,184971039
A_42_P475623	Klf5	2,13E-13	0,666940465	-1,346843857	-1,250431962	-1,681004795
A_42_P482623	Dstyk	2,45E-16	0,77525487	-1,083042706	-1,063805813	-1,198747686
A_42_P484738	Ctgf	2,06E-21	0,876696956	-1,141066949	-1,08863563	-1,322758465
A_42_P485189		2,10E-21	0,828225017	1,288912172	-1,527733201	-2,168781647
A_42_P486203	Agtr1a	3,91E-11	0,646354473	-1,170770159	1,309075449	-1,373096005
A_42_P491454	Gnat1	2,92E-23	0,886339724	-1,023647462	1,080100119	1,512004675
A_42_P491505	Purb	4,37E-14	0,70723156	1,051085202	1,115757663	1,177284418
A_42_P492882	Nckipsd	1,80E-11	0,634187812	-1,103086564	-1,101427649	1,959764392
A_42_P493380	Myc	1,55E-25	0,914758821	-1,162308903	-1,180908954	-1,288950089
A_42_P494276	Armc9	1,06E-09	0,642175204	1,007557957	1,118660583	-1,076588567
A_42_P495001	Slc17a5	7,94E-12	0,667195876	1,1098715	-1,154744193	-1,842162526
A_42_P497323	Ppp1r10	4,03E-29	0,91761436	-1,002856122	-1,259776487	-1,515018279
A_42_P499282	Msrb2	6,43E-13	0,697488438	1,199881361	-1,23020354	-1,728516729
A_42_P501233	Adra1d	1,17E-24	0,907811724	-1,034629678	-1,052660815	1,373226649
A_42_P506402	Slc25a25	3,29E-12	0,678163418	-1,050054098	1,179424346	1,379006497
A_42_P509967	Slc7a9	1,63E-11	0,657976399	1,08669491	2,035401914	-1,127516847
A_42_P511118	Slc17a6	2,66E-13	0,664241998	-1,077165792	1,024636847	-1,250949604
A_42_P515405	Ier3	5,01E-21	0,891250978	1,178176596	1,211054034	-1,618325341
A_42_P518033	Hsd17b1	1,52E-11	0,611491517	-1,018836018	1,300966732	1,220560646
A_42_P522638	Pqlc3	3,54E-13	0,704239891	1,04163326	-1,133353114	-1,485928183
A_42_P523612	Mef2c	2,52E-12	0,701052746	-1,274286955	1,106307768	-1,432672593
A_42_P528106	Glce	8,08E-16	0,764933358	-1,136977008	-1,214299506	1,192315104
A_42_P531971	Hist3h2a	3,73E-20	0,824794995	1,054958049	-1,177188701	-1,177992603
A_42_P536292	Ngb	6,38E-25	0,909944188	1,274067152	-1,12296835	1,219247437
A_42_P536702	Sftpc	2,61E-23	0,886838817	1,524102287	-1,088816179	-1,524211072
A_42_P538868	Dvl1	4,95E-11	0,643172217	-1,115574852	1,047424238	1,299228793
A_42_P540950	Ier2	8,42E-30	0,951959369	1,064573294	1,495749646	1,062911706
A_42_P540972	Rasl11b	2,64E-15	0,75420941	1,077809673	1,038390828	-1,431493168
A_42_P542985	Cbln2	4,02E-20	0,837932664	-1,131700206	1,141992258	-1,311763102
A_42_P543004	Socs2	1,56E-16	0,762109574	1,072202287	-1,01667448	-1,637325616
A_42_P545100	Aspdh	9,19E-15	0,723579196	1,088808715	1,357475703	-1,005602652
A_42_P547036	Pak4	2,91E-10	0,697205897	1,115305911	1,124679505	1,438300086
A_42_P547629	Fam49a	1,15E-11	0,719245696	-1,385074704	1,211631474	-1,560193859
A_42_P548410	Acot1	1,18E-12	0,69045879	-1,046970044	1,034756628	-1,571375313
A_42_P550241	Fat2	3,73E-18	0,775341039	-1,049558608	1,137987771	1,365500413
A_42_P550344	Tle3	1,08E-18	0,81666687	-1,054610905	-1,047499452	1,490274499
A_42_P554066	Tasp1	8,32E-12	0,619880912	1,018287776	-1,319206237	-1,169203919
A_42_P554157	Dnajb11	4,36E-14	0,685415329	1,04393179	-1,104518417	1,529764538
A_42_P555131	Tmbim7	1,29E-13	0,695301448	1,148515877	1,773123162	-1,3669198
A_42_P555140	Izumo4	5,38E-13	0,718463966	1,09818372	1,083822904	-1,255812554
A_42_P555780	C5	2,52E-14	0,732383613	1,223533947	-1,269846687	-1,51529848
A_42_P558703	Adamts15	1,32E-18	0,828862433	-1,082167485	-1,094068114	-1,390118432
A_42_P559414	Wnt4	3,78E-32	0,936103718	-1,075569164	1,011941969	1,479190024
A_42_P567527	Traf4	4,67E-12	0,673855816	-1,03629278	1,297685784	1,56982724
A_42_P568916	Gatad2a	1,57E-10	0,62712196	1,115239372	-1,032699885	1,519349601
A_42_P573441	Klf3	2,45E-11	0,604794796	-1,081972919	1,025778643	-1,180263742
A_42_P574859	Hamp	2,76E-13	0,773934318	1,415438975	-1,054078601	-1,731284072
A_42_P581135	Ist1	3,64E-12	0,67692096	-1,040171626	1,154930942	-1,087254997
A_42_P584179	Lrrc73	7,30E-17	0,76864026	-1,049148481	1,203616434	1,096925313
A_42_P584734	Gdf10	5,37E-31	0,936262669	1,660482594	-1,470250984	1,751973557
A_42_P588785	Mthfd2	8,01E-20	0,81982588	-1,024221096	1,238917302	2,158586505
A_42_P589201	Slc15a4	4,20E-11	0,622503408	1,023106184	1,022130227	-1,263980591
A_42_P592157	Pbx2	9,33E-23	0,846348138	-1,017938485	1,026929562	1,437698132

A_42_P597322	LOC100125367	1,50E-20	0,866772702	1,156571518	-1,312334505	-1,390868822
A_42_P603213	Elfn2	1,58E-22	0,868223343	1,091618614	-1,43452836	1,313917962
A_42_P603461	Isoc1	2,60E-13	0,726305858	1,044912676	-1,187564004	1,15936918
A_42_P605985	Ddit4	1,83E-12	0,685268254	-1,09317043	1,322465375	-1,005732849
A_42_P608768	Bbs12	7,69E-18	0,802662896	-1,098993088	-1,01660106	-1,284530984
A_42_P615837	Ckmt2	3,09E-13	0,724447252	1,099232666	1,157654133	-1,05334471
A_42_P623839	Ttc39b	1,34E-23	0,879818133	-1,163938152	1,227080613	-1,15104513
A_42_P629370	Klf15	1,24E-13	0,750888277	-1,185785393	-1,042325149	-1,440822584
A_42_P630572	Cpne8	1,01E-09	0,621964218	-1,366599237	-1,181052187	-1,290289342
A_42_P636761	Gzmm	4,82E-15	0,808847477	-1,025645067	-1,286266171	1,49966639
A_42_P640792	Gjb6	1,97E-12	0,70388471	1,298469368	1,167698534	-1,703452903
A_42_P646922	Sgcb	2,77E-20	0,852382831	-1,093961089	-1,044765658	-1,104373955
A_42_P648200	Pak1ip1	8,96E-12	0,685904066	1,030373554	-1,277487481	1,61934045
A_42_P655897	Tspan9	2,26E-22	0,877126145	1,003901428	1,233185114	1,617522073
A_42_P664544	Rftn2	4,98E-12	0,673053345	-1,003755199	1,073636015	-1,746514257
A_42_P664913	Ankrd37	4,36E-31	0,936757961	1,047334727	-1,15571346	-1,400017179
A_42_P668126	Sema6c	4,07E-17	0,773551266	1,10913872	1,216395045	-1,801579754
A_42_P669103	Lnx2	3,29E-21	0,839704122	1,028796878	-1,071818472	1,212458023
A_42_P670580	Vipr1	2,94E-33	0,947476406	-1,014413676	1,070039994	1,276463336
A_42_P670631	RGD1305645	6,60E-20	0,834896191	-1,030078931	1,21816864	1,076787674
A_42_P673905	Mrnip	3,96E-20	0,861635086	1,096268736	1,018037155	-2,122909838
A_42_P676553	Tnni3	9,18E-13	0,672374677	-1,098543032	1,11557647	1,417453689
A_42_P677662	Ttc30b	5,05E-18	0,772877111	1,005064591	-1,233117968	-1,952477956
A_42_P682589	Nr4a1	1,03E-28	0,951719014	-1,056825505	1,634142167	-1,129164596
A_42_P683840	Ccl27	3,87E-20	0,850478647	-1,035695779	1,302599368	-1,040421747
A_42_P685330	Ap1s2	1,89E-16	0,760380359	1,098219665	-1,128734818	-1,482983905
A_42_P686234	Muc4	2,84E-10	0,618531385	1,255716998	-1,362443554	-1,478295477
A_42_P686879	Siglec1	6,32E-10	0,649523091	1,285859064	-1,345591754	1,145646424
A_42_P694161	Arl14ep	3,47E-10	0,615612636	-1,109893967	-1,119612326	-1,004530571
A_42_P695042	Csf1	2,40E-18	0,83756443	-1,036140544	-1,214734733	1,472261477
A_42_P703023	Slc38a3	1,89E-11	0,608434964	-1,104494412	1,558437178	-1,036865958
A_42_P708068	Egr4	6,61E-19	0,833330457	1,251462707	1,892365521	1,202254278
A_42_P708593	Per2	3,99E-18	0,821451542	-1,355952593	1,164812546	1,731133469
A_42_P712801	Entpd3	4,90E-27	0,925444078	1,085164299	-1,429506668	1,34317874
A_42_P718331	Sstr3	6,97E-37	0,975058259	1,391593167	1,19416934	1,35239548
A_42_P721692	Inpp4b	8,16E-21	0,859112078	-1,222406085	1,024842223	-1,105046164
A_42_P723173	Id1	1,08E-31	0,950760412	1,058982533	1,362595833	1,251557002
A_42_P723540	Dyrk3	2,60E-20	0,827108147	-1,187580097	1,118283789	-1,468015245
A_42_P727368	Cdca7	8,62E-17	0,748523938	1,073503392	-1,158432468	1,483794564
A_42_P729012	Cercam	1,35E-13	0,714857127	1,075384751	1,139808166	1,231227483
A_42_P730320	Clhc1	4,36E-14	0,726781595	-1,000552112	1,880147516	-1,243739374
A_42_P738559	Pdgfa	2,21E-11	0,631376092	-1,02806911	-1,010860558	1,464441852
A_42_P743580	Rgs4	1,05E-28	0,929437293	-1,066931087	1,319833167	-1,342524996
A_42_P754654	RGD1562378	3,78E-25	0,911757871	1,163535243	-1,144376169	-1,4011085
A_42_P756652	Dnph1	1,93E-13	0,668113297	1,238604489	1,03977427	1,147600538
A_42_P758200	Rnf112	2,46E-12	0,660182055	1,00784056	1,01776481	1,16454442
A_42_P759843	Arl4c	1,35E-16	0,795599055	-1,125724915	1,218627819	1,188756171
A_42_P759993	Mefv	5,64E-12	0,671508243	1,212657091	-1,214885119	-1,649238871
A_42_P760354	Pla2g2c	8,41E-15	0,703513819	1,500078947	-1,297674956	-1,650848753
A_42_P761023	Vstm5	2,33E-24	0,896772908	1,072429559	-1,379247507	1,143278989
A_42_P766909	Ppp1r14a	2,26E-14	0,76717353	1,130333328	1,362032577	-1,222887001
A_42_P768883	Perp	5,81E-17	0,787097571	-1,19060553	1,296637478	-1,295908897
A_42_P776018	Stard4	9,07E-15	0,702699891	-1,08739084	1,203628264	1,718741144
A_42_P777268	Cep126	5,16E-17	0,771573539	-1,046818227	-1,800027877	1,196570582
A_42_P779933	Capn3	3,18E-11	0,669977703	1,029569813	1,461530311	-1,356349843
A_42_P787775	Plekhd1	2,04E-18	0,825970661	1,173750352	-1,14629345	1,716746592
A_42_P791436	Gfra2	2,64E-19	0,826102124	1,141446225	-1,312886246	1,117662993
A_42_P791677	Areg	1,77E-13	0,711937816	-1,077357974	-1,55281668	-1,210759775
A_42_P794120	Stra6	3,17E-21	0,852615142	-1,280488643	1,030587362	1,67734726
A_42_P794613	Mvd	2,53E-11	0,629516431	-1,010606833	1,143539714	1,680202699
A_42_P794748	Acad11	3,94E-11	0,623392177	-1,071249596	-1,126385536	-1,390697246
A_42_P795269	Lgalsl	2,15E-12	0,720992591	-1,13097441	-1,17199563	1,117446747
A_42_P797218	Gpt	1,99E-09	0,611722902	1,024403832	1,051117343	1,673181974
A_42_P800949	Hipk3	3,71E-09	0,623458536	-1,151370258	1,274934685	-1,015309105
A_42_P802358	Fam110b	9,61E-10	0,60030019	1,098874129	1,082419666	-1,322250334
A_42_P808442	Siae	4,02E-22	0,83809296	1,098325747	-1,110973006	-1,555684128
A_42_P811308	Phf21a	9,75E-22	0,858971106	-1,023998122	1,070956825	-1,410604513
A_42_P812805	Smoc2	9,92E-09	0,608037116	1,032365354	-1,270778882	-1,778830936
A_42_P816878	Rgs7bp	6,46E-10	0,606328068	-1,249021577	-1,058744291	-1,653855237
A_42_P820657	Il1r2	8,45E-13	0,747680923	1,07454527	1,211902319	-1,177575317

A_42_P821898	Efcab7	2,14E-14	0,734007734	-1,089756848	-1,139580366	-1,428572406
A_42_P822726	Myo1g	5,02E-12	0,692908712	1,119948102	1,114669102	1,134118948
A_42_P824369	Foxf1	6,48E-22	0,882136732	1,005763289	-1,781627288	1,257571912
A_42_P828242	Lgr4	9,43E-12	0,685275637	-1,113399338	-1,115599931	-1,229638482
A_42_P829031	Coasy	2,27E-13	0,70919253	1,072530311	1,174997486	-1,121532789
A_42_P834031	Hrasls	4,59E-12	0,652269679	1,341227031	-1,677763511	-1,655095427
A_42_P841193	Ddit4l2	2,67E-21	0,875440963	1,545396473	-1,216501492	1,054035823
A_42_P842580	RGD1566239	1,09E-14	0,70073722	1,107938967	1,410666993	1,163615436
A_42_P842823	Reep6	5,65E-09	0,637185947	-1,061755285	-1,196686295	1,421381818
A_43_P10047	Slc20a2	7,41E-14	0,721247418	-1,061203592	-1,072104303	-1,198633175
A_43_P10102	Spry4	1,06E-17	0,814599362	-1,319432768	1,125416139	2,395024643
A_43_P10133	Spns2	7,25E-28	0,92405592	1,103742297	1,027361304	1,457401014
A_43_P10136	Amz2	6,43E-12	0,669859485	-1,014671535	1,277457935	-1,129909363
A_43_P10290	Tspan1	5,82E-29	0,907871474	1,042094868	1,109819321	-1,302475378
A_43_P10292	St6galnac4	4,32E-11	0,665989363	1,183892152	-1,168954694	-1,028416791
A_43_P10498	Bag2	6,17E-28	0,924520993	-1,083210966	1,178183615	-1,023263145
A_43_P10824	Cib2	5,79E-10	0,630169569	-1,251025997	1,308016691	-1,154233077
A_43_P10935	RGD1562339	2,49E-09	0,649420917	-1,258643823	1,266713449	-1,048682018
A_43_P11044	G0s2	1,50E-20	0,855785142	-1,189778753	1,329978081	1,75683926
A_43_P11116	Prss30	9,77E-13	0,671614147	1,13957114	1,079352029	-2,26533768
A_43_P11136	Col9a1	4,74E-12	0,673668725	1,202937352	1,19489906	-1,566166714
A_43_P11144	Pdrg1	5,77E-10	0,608049886	1,017609257	1,216232837	1,488954141
A_43_P11146	Lyplal1	6,87E-13	0,652589017	1,131738921	1,105505994	-1,614959292
A_43_P11259	Mrpl19	3,25E-14	0,688722186	-1,001390136	1,190363748	1,065662336
A_43_P11268	Cdkn1b	1,83E-11	0,608922725	-1,066167714	1,022075329	-1,82233867
A_43_P11380	Ncapd3	7,89E-11	0,613551103	1,085378977	-1,328630118	-1,091547362
A_43_P11441	Atp4b	1,80E-19	0,853197569	1,705369406	-1,688204389	-1,938334689
A_43_P11471	Grin2c	4,61E-23	0,910101011	1,071645696	-1,158628421	1,587831916
A_43_P11476	Inha	6,98E-11	0,638455962	1,078862596	-1,096760988	-1,178806834
A_43_P11560	Aqp5	1,97E-12	0,783598759	2,081141659	-1,035854538	1,435027715
A_43_P11580	Abcc2	1,21E-13	0,734328621	1,169353874	1,108756883	-2,065330122
A_43_P11616	Atf3	2,58E-17	0,845844687	-1,102245961	1,463559978	-1,25769112
A_43_P11654	Pnoc	2,03E-18	0,838601154	-1,366691951	1,028704221	-1,202304252
A_43_P11685	Id2	4,67E-17	0,830209422	-1,151262859	1,289650437	-1,027136792
A_43_P11820	Kcnj8	1,31E-13	0,750344385	-1,010145401	1,160163049	-1,242524036
A_43_P11897	Gabrd	2,10E-19	0,797364984	-1,025244035	-1,091195477	-1,159181648
A_43_P11901	Kcnj5	7,45E-31	0,935482859	1,081799938	-1,444833481	1,171602762
A_43_P11932	Nr4a3	1,78E-34	0,961556291	-1,300122713	1,596073467	1,319697615
A_43_P12023	Nr4a2	4,96E-15	0,748295786	-1,461707961	1,520075424	-1,985457941
A_43_P12073	Uts2r	8,63E-15	0,742990395	1,190260677	1,573602429	1,195373177
A_43_P12090	Pold1	8,10E-17	0,799578825	1,187668909	-1,116344452	-1,14349283
A_43_P12246	Prpsap1	5,02E-12	0,651096535	-1,028590497	1,189912864	-1,02713136
A_43_P12460	Lrrn3	7,38E-18	0,802966625	-1,06906917	1,036994738	-2,050308498
A_43_P12584	Nfyb	3,65E-13	0,739998929	-1,021805793	-1,075408118	1,098785868
A_43_P12825	Entpd6	2,69E-10	0,641170114	-1,026262646	1,107597723	-1,203350318
A_43_P12827	Nme3	1,04E-14	0,758335556	-1,005813961	1,208519155	-1,033230102
A_43_P12924	Abcg5	1,98E-17	0,795520142	1,227490535	-1,365487446	-1,373812199
A_43_P12927	Dusp1	5,16E-21	0,849908009	-1,135136742	1,120985961	-1,993355463
A_43_P12967	Cap2	1,96E-14	0,783444359	-1,141610728	1,158627943	-1,149841734
A_43_P13023	Neu3	1,35E-14	0,71965811	1,18036858	-1,28234647	-1,682037069
A_43_P13029	Acsl3	2,05E-12	0,662504383	-1,089458116	-1,082904358	1,250967726
A_43_P13041	Nr0b2	6,74E-11	0,660117737	1,298432035	-1,098815602	-2,063484126
A_43_P13109	Cyp26a1	1,97E-13	0,74629897	1,107568808	-1,126872414	1,745327676
A_43_P13290	Pbld1	3,02E-22	0,865016362	-1,03640204	-1,252261283	-1,327125862
A_43_P13418	Kcnk2	2,80E-11	0,650828656	1,202495312	-1,261739679	-1,128136478
A_43_P13931	Grem2	7,78E-11	0,636960134	-1,078909043	-1,022123892	-1,926123127
A_43_P14131	Rgs2	3,39E-12	0,656142246	-1,07847114	1,033630331	-1,602287572
A_43_P14648	Art3	1,88E-12	0,684947845	-1,262006088	-1,016432147	-1,572460808
A_43_P14687	Fez2	1,51E-10	0,627667566	-1,19445493	1,148717942	-1,123864044
A_43_P14752	LOC102554727	8,04E-13	0,714043632	1,030618439	1,233182063	-2,421732153
A_43_P14802	Ncoa6	5,14E-14	0,683551966	-1,05817239	1,011317214	1,319372134
A_43_P14809		1,87E-09	0,633808146	1,157683025	1,036144562	1,526940896
A_43_P14954	Sos1	7,20E-20	0,834358412	-1,112818046	1,244017449	1,248903303
A_43_P15021	Slc25a29	1,61E-26	0,897483223	1,096708534	1,104991278	-1,45155295
A_43_P15205	Srf	2,41E-21	0,85412917	-1,005033696	1,299987346	1,711161621
A_43_P15246	Pthlh	1,65E-31	0,944745077	-1,079573149	1,134634856	-2,046928345
A_43_P15253	Icam1	5,50E-17	0,771031514	-1,099712891	1,213740659	-1,368502528
A_43_P15390	Smad7	2,05E-14	0,783034366	1,080812916	1,127815957	1,373414414
A_43_P15406	Grm4	4,06E-15	0,731765389	1,011943642	-1,007384469	1,282559894
A_43_P15447	Bglap	1,21E-15	0,743496572	-1,090854246	1,251928448	1,392652002

A_43_P15548	Gabra6	3,08E-13	0,741748159	-1,206154192	-1,164325132	-1,127376828
A_43_P15600	Rab29	3,12E-17	0,792019025	-1,000567003	-1,030047266	-1,130979814
A_43_P15701	Rab13	5,23E-11	0,642432356	1,047445359	1,147323116	-1,181703602
A_43_P15824	Acvr2b	2,43E-11	0,630104835	-1,010208419	1,093899079	-1,724435293
A_43_P15837	Ntrk2	5,08E-13	0,736576686	-1,028657047	-1,299413879	1,423236643
A_43_P15933	Chrm4	2,32E-12	0,720167366	-1,006734912	-1,228741541	1,530857706
A_43_P16449	Pelo	5,38E-11	0,682635509	-1,039961413	1,174232282	-1,060304478
A_43_P16457	Creld2	2,78E-15	0,735493768	1,165455448	-1,057948488	1,279403531
A_43_P16491	Col16a1	9,36E-24	0,891163634	1,045283492	1,003735122	1,845148652
A_43_P16529	Gadd45b	2,49E-17	0,793753291	-1,015152401	1,125312177	-1,443102335
A_43_P16989	Snx9	1,68E-10	0,626125629	-1,02598904	-1,131004878	-1,199942637
A_43_P17271	Eva1c	5,71E-11	0,618176986	1,094608679	-1,708173479	1,192577398
A_43_P17298	Naaa	8,42E-15	0,743228901	1,149519333	-1,72093245	-1,13377319
A_43_P17330	Nup35	4,01E-15	0,731895379	-1,011768564	1,054975831	1,100516469
A_43_P17925	Abca8	1,72E-11	0,657229861	-1,079272482	1,19446145	-1,909345917
A_43_P18332	Lrfn2	2,49E-26	0,920592462	1,226451731	-1,575091813	1,443567231
A_43_P18356	Ift57	7,48E-10	0,604113042	1,011855684	-1,213418675	-1,206021662
A_43_P18571	Tekt4	2,37E-18	0,824971662	1,059733394	-1,340228434	-1,340063588
A_43_P18638	Asb18	1,06E-14	0,740982854	-1,18774722	1,028475887	-1,75614507
A_43_P19743	Krtcap3	5,30E-18	0,819487294	1,146897245	1,206849503	1,167317383
A_43_P19889	Angptl6	3,20E-11	0,60093684	1,011606723	-1,178016225	-1,164784328
A_43_P19891	Rrh	2,29E-25	0,905495126	1,070006826	-1,217844513	2,249400637
A_43_P20684	Tceanc2	3,64E-14	0,728633743	1,299070232	-1,232896071	-1,492897624
A_43_P20963	Wdr86	3,37E-21	0,839555377	1,263002872	-1,355943401	-1,216104327
A_43_P21118	Klhl24	7,44E-14	0,721201993	-1,211314846	-1,155802747	-3,316916444
A_43_P21279	Pip5k1b	6,66E-10	0,605872172	-1,009026294	-1,453449047	-1,184442243
A_43_P21414	Atg10	2,06E-16	0,740519411	1,273843223	1,017715909	-1,257719714
A_43_P21913	Usp53	8,24E-13	0,694626213	-1,585258805	-1,379092519	-1,219820697
A_43_P22555	Fam189a2	9,92E-25	0,890949219	-1,236663293	1,108552692	-1,464896209
A_43_P22811	Cd6	1,40E-22	0,927130608	1,549968461	-1,56036968	-1,74592901
A_43_P22825	Cabyr	2,26E-16	0,758818104	1,100076353	-1,593538852	-1,031058578
A_43_P22864	Rad54l	3,70E-29	0,925398513	1,002107548	-1,18377242	1,270800885
A_43_P23014	Lpxn	4,18E-11	0,622577919	1,145887372	-1,493376072	-1,44075757
A_43_P23152	Rhoh	2,98E-11	0,650007134	1,160323913	1,015432014	-1,841833488
A_43_P23155	Mcm10	6,18E-11	0,617037221	1,038382166	-1,507749357	-1,203622399
A_43_P23187		8,58E-16	0,780526318	-1,183267706	-1,036189514	1,062403386
A_44_P1000400	Safb2	1,04E-12	0,670809483	1,018886112	1,332437174	-1,178776259
A_44_P1003794	Nr3c1	6,73E-13	0,676118417	1,048096243	-1,181727708	1,233731516
A_44_P1006261	Nme4	1,59E-17	0,781208651	1,258753858	-1,186458427	-1,286801123
A_44_P1011386	Znhit6	1,49E-09	0,67538751	-1,178913018	-1,382172825	1,521903538
A_44_P1011953	Gpcpd1	6,95E-12	0,646865115	-1,094960131	-1,387809502	1,307993889
A_44_P1012556	Stbd1	8,51E-10	0,700405534	1,344544132	-1,112839659	1,003233721
A_44_P1012940	RGD1306072	4,61E-12	0,627887891	1,257687584	-1,074727928	-1,281924397
A_44_P1014018	Mex3d	4,88E-14	0,759963918	-1,005662963	1,269105065	2,181645962
A_44_P101533	St6galnac5	2,17E-22	0,877313431	1,252742526	-1,020342708	1,100420865
A_44_P1015754	Slc22a3	1,43E-16	0,822389127	-1,022059538	-1,338089374	1,441816036
A_44_P1015839	Mroh7	1,86E-26	0,921491231	1,265303106	-1,457078192	1,469406132
A_44_P1016170	Prpf4b	4,91E-27	0,892020203	-1,123511397	1,135941233	1,339876008
A_44_P1016691	Fhl4	4,38E-27	0,932236694	1,164616005	-1,510797572	-1,188844892
A_44_P1016829	Trib1	1,37E-18	0,783273152	-1,431212061	1,03700151	-2,673845118
A_44_P1018090	Lgi4	5,61E-22	0,849755462	1,151072808	1,118435989	1,442253605
A_44_P1018447	Micall2	9,39E-11	0,65567414	1,04313411	1,246261799	1,417788133
A_44_P1018696	Muc19	2,91E-12	0,679656348	1,082921541	-1,28608774	-1,133038411
A_44_P1019407	Rnf103	5,99E-11	0,640568409	-1,057848748	-1,292169386	-1,135810221
A_44_P1019480	Cdc42ep2	5,00E-15	0,765090953	1,110199586	1,547080031	1,120214463
A_44_P1021808	RGD1561796	4,93E-08	0,622600385	1,268015029	-1,046821602	-1,204189422
A_44_P1022002	Ccl7	7,20E-32	0,934587173	1,209190584	-1,039730626	1,414532868
A_44_P1022395	Fbxl3	1,23E-16	0,764122017	-1,034005601	-1,093073331	-1,711275292
A_44_P1024065	Hdac5	5,36E-21	0,881850931	-1,129391133	1,343293958	-1,703216865
A_44_P102774	Polr3e	3,24E-12	0,604759344	-1,142246224	1,074117658	1,417392097
A_44_P1029253	Mas1	1,63E-11	0,657975451	1,216529951	2,089408527	-2,039292388
A_44_P1029805	Krt7	1,19E-29	0,935019361	1,173655313	-1,223590767	1,455282362
A_44_P1031390	Trappc4	4,10E-18	0,791843836	1,103779139	1,168994876	-1,151447234
A_44_P1034018	N4bp3	1,36E-11	0,613117007	-1,052254865	1,272425042	1,545390217
A_44_P1034541	Pim3	3,65E-18	0,822059173	1,051913567	-1,017671803	1,32579764
A_44_P1035071	Nmt2	1,42E-11	0,699109593	-1,071381144	-1,196121812	-1,071802265
A_44_P1035706	Lyrm9	1,22E-12	0,645358147	1,211457579	1,253957121	1,195992921
A_44_P1037706	Glod5	4,46E-20	0,860991454	1,13345454	-1,93469782	1,109314623
A_44_P1037806	Hpx	4,06E-27	0,902509589	1,227904134	1,200183402	1,901298434
A_44_P1038028	Tnfrsf12a	5,97E-14	0,703837029	-1,031030278	1,107584544	1,564899491

A_44_P1039213	Cpa2	1,77E-14	0,695455461	1,212147682	1,059355628	-1,384487732
A_44_P1039809	Rasip1	1,40E-12	0,707833068	1,212473445	-1,042073864	1,396083276
A_44_P1042696	Smim29	1,62E-16	0,761781466	1,121570221	1,14543412	-1,248226893
A_44_P1043302	Crb3	2,94E-15	0,785187838	-1,024277633	-1,318163685	-1,182712034
A_44_P1044030	Klhdc9	2,01E-27	0,921057741	1,047046685	1,050359482	-1,345989868
A_44_P1044046	Ndufaf1	5,45E-25	0,893355505	1,113977876	-1,182342447	-1,199558611
A_44_P1044565	Cdadc1	1,46E-14	0,755187181	-1,20239098	1,18029222	-1,157753405
A_44_P1045734	Stac3	7,10E-14	0,771845705	-1,23544127	1,570641438	2,212080672
A_44_P104652	Slc38a2	2,80E-29	0,910245219	-1,10890266	-1,2665305	-1,401310441
A_44_P1047315	Lynx1	6,13E-12	0,624045632	-1,018675321	1,430097395	-1,170830619
A_44_P1047924	Herpud1	9,88E-22	0,898196144	-1,237013015	1,064316733	1,278026579
A_44_P1050200	C1qtnf12	1,91E-17	0,823802738	-1,018192356	-1,127150749	2,273940697
A_44_P1050510		9,97E-20	0,81836946	1,11097355	-1,371744822	-1,840587199
A_44_P1053605	Hcn1	1,64E-13	0,778756892	-1,38428454	1,166097944	-1,149540424
A_44_P1053951		1,24E-26	0,915415157	1,180453539	1,090065631	1,307329161
A_44_P1054213	Hspa5	1,44E-21	0,830512849	1,095522577	-1,208482995	1,665993417
A_44_P1057412	Ddx25	8,86E-16	0,764115857	1,106748371	1,074678374	1,249679378
A_44_P105749	Rimbp3	4,69E-27	0,918472924	1,302337436	-1,33458517	1,469628388
A_44_P1058112	Yars2	8,46E-18	0,801954885	1,000541206	-1,256158663	1,827257303
A_44_P1059903	Lzts1	5,04E-28	0,925095734	-1,126376039	1,295220774	-1,27484849
A_44_P109927	Ahsa2	2,17E-11	0,654232207	-1,058026816	1,108100374	1,667446392
A_44_P112082	Ccdc89	1,31E-11	0,681242714	-1,079334377	1,281706434	-1,82769447
A_44_P114692		4,14E-17	0,804673899	1,056804548	-1,148392804	1,437382673
A_44_P114788		9,33E-11	0,611134308	1,011598701	1,402029611	1,633394608
A_44_P115192	Slc5a5	1,31E-09	0,694691105	1,242386286	-1,276468512	1,081929842
A_44_P116283	Klhl41	1,96E-13	0,710811997	-1,118392189	-1,447173654	-1,026975554
A_44_P117119	Hpdl	2,33E-16	0,791256331	1,118385623	-1,043830961	2,250433432
A_44_P118724	Arc	3,20E-24	0,89552772	1,183641545	1,292799594	1,501182867
A_44_P119177	Parp2	1,94E-11	0,608093873	1,003028406	-1,212739468	-1,230389588
A_44_P119575		2,07E-11	0,607152322	1,030863726	-1,522697512	-1,656350439
A_44_P127597	Dnajb9	3,77E-11	0,667761118	1,128929105	-1,306787962	-1,131267887
A_44_P128062	Syt17	1,07E-10	0,692277315	-1,035891257	-1,235742945	1,431231554
A_44_P131470	Hexb	9,80E-16	0,779399882	-1,090314199	-1,075463124	-1,164869105
A_44_P132610	Slc20a1	5,70E-12	0,671362773	1,021147945	-1,266990854	1,448936015
A_44_P135224	Plk3	5,05E-10	0,652686008	1,049188188	1,532022266	1,4430432
A_44_P135356	RGD1565844	3,66E-16	0,787613829	-1,130063075	1,184446947	1,062044488
A_44_P144277	Pkn1	1,62E-10	0,603068155	-1,030593833	1,260552233	1,159566348
A_44_P152275		3,57E-10	0,615164069	-1,432403652	1,577266025	1,491531679
A_44_P156262	Myod1	1,07E-20	0,832630999	-1,043871407	-1,120348413	1,932593347
A_44_P159171	Mylip	4,46E-14	0,685172481	-1,06753568	1,290875433	-1,444295155
A_44_P160846	Rnasel	1,81E-20	0,829390681	1,056442568	-1,618664051	-2,262661234
A_44_P161770		5,94E-12	0,670849458	1,157444847	1,755837023	1,075725401
A_44_P163242	Epcam	7,65E-16	0,810010766	1,583443939	-1,030716554	1,048307453
A_44_P165999	Coq8a	3,74E-15	0,750965223	-1,028531001	1,014775005	-2,265443091
A_44_P166967	Npy1r	8,72E-18	0,76837784	1,091071593	1,787278998	-1,241938374
A_44_P180268	Lrrc8d	4,21E-16	0,770630611	-1,12700362	-1,359487965	1,466959204
A_44_P184484	Orc1	6,00E-17	0,751766311	1,200539773	-1,21011715	-1,225413033
A_44_P184685	Sertad1	1,80E-09	0,613276981	1,007333026	1,45319069	1,318323251
A_44_P187195	C1galt1	3,81E-15	0,797316672	-1,412515031	1,350321344	-1,508462111
A_44_P191285	RGD1564171	2,71E-17	0,758746636	1,061582468	-1,535819989	-1,871149175
A_44_P192083	Ffar4	7,29E-22	0,860495687	1,026713806	-1,59629701	-1,679537724
A_44_P192406	Dusp9	2,95E-27	0,926891303	1,457173133	1,111476727	1,754924543
A_44_P194925	Pigx	4,56E-12	0,712513353	1,094764803	1,090191135	-1,173429169
A_44_P195648	Ufm1	1,98E-10	0,600111923	1,119750825	1,024931058	1,145713933
A_44_P196856	Cir1	5,05E-11	0,619897592	1,066532767	-1,26634654	-2,196473418
A_44_P198620	Nos3	1,42E-19	0,842876363	-1,034952101	1,076150721	-1,479104086
A_44_P198705	H1f0	5,44E-22	0,908525853	1,318059127	-1,36531989	1,095076586
A_44_P203859	Piezo2	9,07E-10	0,601177517	-1,581767728	-1,066408427	-1,535341017
A_44_P204207	Smyd4	2,96E-10	0,617931362	1,13778374	-1,195671702	-1,67977265
A_44_P209788	Enpep	1,85E-11	0,608764992	-1,076584381	-1,477854963	-1,153350252
A_44_P210492	Syt2	6,59E-25	0,909833124	1,187793547	-1,253868538	1,190653479
A_44_P211037	Adgrb2	2,32E-20	0,85338351	1,05695493	1,283376386	1,293293036
A_44_P211061	Etv1	1,88E-11	0,656076242	-1,123454682	-1,025315621	-1,51543611
A_44_P213175	Ccdc68	2,95E-13	0,685815734	-1,01347221	-1,394880605	-1,699192967
A_44_P214288	Cabp7	1,00E-32	0,939117486	1,37646716	-1,075378706	1,58900581
A_44_P222004	Il15	1,99E-13	0,667778309	-1,184052992	-1,149696496	-1,302053799
A_44_P222533	Actrt3	2,23E-14	0,751116003	-1,014507795	1,17188902	-1,95060116
A_44_P224020	Kcna4	2,85E-31	0,943592452	-1,215384358	1,079702326	-1,909481708
A_44_P224547	Plcb1	3,06E-11	0,601589375	-1,165988315	-1,183934022	-1,054710516
A_44_P226658	Scnn1g	3,60E-28	0,918813445	1,526422455	-1,3472726	-1,713135778

A_44_P227221	Syde2	1,20E-19	0,876227354	-1,119569275	-1,254313816	-1,330624959
A_44_P228942	Runx1	2,68E-18	0,810314111	-1,109062653	-1,289758076	1,58243558
A_44_P229299	Mepce	8,25E-11	0,612917683	-1,033778784	-1,026051866	-1,929969589
A_44_P229677	LOC363337	1,56E-18	0,827755619	-1,220908269	-1,557709931	1,284611396
A_44_P230697	Igdcc4	1,96E-17	0,779535605	-1,051275773	-1,103949616	1,437704818
A_44_P231243	Insig2	9,33E-11	0,655766369	-1,227662636	1,034534609	-1,74381175
A_44_P233080	Egr1	4,99E-30	0,957373853	1,184373569	1,329195082	-3,506859699
A_44_P236407	Lnx1	8,62E-24	0,900365503	-1,063732863	-1,084017975	-1,618376224
A_44_P236600	Lrrtm3	5,03E-24	0,884141799	-1,073025834	-1,037491927	-1,211814522
A_44_P237621	Txnip	2,09E-22	0,896160474	1,960751087	-1,994386414	-2,188297672
A_44_P240696	Nppc	2,79E-20	0,873824691	-1,05440623	1,85128014	3,109486083
A_44_P241409	Vgll4	3,76E-17	0,80539583	-1,007686343	1,130029731	-1,244480936
A_44_P243355	Atp10a	1,44E-20	0,844041242	-1,22328779	-1,121244426	-1,514506637
A_44_P243477		6,70E-15	0,818981266	1,163617735	-1,55575972	-1,257949058
A_44_P243534	Pxdc1	5,49E-12	0,710365891	-1,075008584	-1,002042041	-1,700855832
A_44_P246114	Tle1	1,00E-13	0,736212666	-1,21824483	-1,270914659	-1,461290358
A_44_P248090	Thbs2	1,91E-09	0,689639607	1,273460044	-1,19874866	-1,091542845
A_44_P250203	Rbm12	1,78E-25	0,906395956	1,043301769	-1,014493556	1,297963874
A_44_P252483	Ackr3	1,31E-19	0,830618454	-1,23232744	-1,227257259	-2,360387675
A_44_P253012	Spry2	6,71E-13	0,67614065	-1,125682054	-1,524198146	-1,598882255
A_44_P253208	Adamts9	2,01E-17	0,79543505	-1,05593115	1,126213738	1,412716168
A_44_P256677	Plcxd3	1,07E-11	0,702506766	-1,263496907	1,174549023	-1,254610055
A_44_P257681	Zfp78	8,66E-20	0,833208296	-1,102981735	-1,106004488	-1,857318828
A_44_P258241	Dbx2	6,36E-14	0,703138308	-1,092607295	1,17637381	-1,067924726
A_44_P263440	Hbp1	1,53E-15	0,741262146	-1,143885139	-1,44416852	-4,228484235
A_44_P265013	Pear1	1,65E-18	0,781831453	1,189017479	-1,281295355	-1,455552701
A_44_P265434	Mif4gd	3,13E-11	0,601265045	1,237776589	-1,073681432	1,305774543
A_44_P265544	Crybb3	4,49E-16	0,838485367	1,311832734	1,112214276	-1,073093578
A_44_P267041	Lat	2,24E-11	0,631233025	1,121879247	1,334326919	1,276424009
A_44_P267199	Spred3	4,10E-16	0,77086586	-1,308150836	1,431863388	1,40262262
A_44_P269522	Tmem183a	7,78E-10	0,625835734	-1,078027622	-1,173600524	1,145769215
A_44_P269930	Mfsd2a	4,28E-10	0,634525367	1,03062926	-1,028887923	1,609373564
A_44_P271658	Reg3b	4,09E-16	0,75350531	1,313121244	-1,893125993	-1,311950511
A_44_P273777	Masp1	2,43E-13	0,727011863	-1,301741061	-1,075332766	1,930294671
A_44_P274762	Cpped1	3,31E-16	0,772707545	1,13821394	-1,337500133	-1,172121935
A_44_P276087	Ampd1	2,02E-12	0,684043541	1,128319386	-2,249751414	-1,179089662
A_44_P277669	Myo19	4,23E-10	0,612678568	-1,027249095	-1,146253199	2,145527868
A_44_P279222	Faap20	1,18E-11	0,639844229	1,101358483	1,060015838	-1,276139169
A_44_P279337	Slc22a5	7,86E-25	0,881836841	-1,114322007	-1,242930237	1,030643793
A_44_P280786	Cyp2c11	1,99E-09	0,611735069	1,154700595	2,195895637	-1,559594854
A_44_P282164	Pdia2	1,68E-27	0,921595318	1,132855766	-1,082024504	2,036065396
A_44_P283176	Spats1	1,15E-17	0,799688798	1,115385329	1,436555086	-1,254459679
A_44_P284067		1,41E-10	0,670081942	-1,326313394	-1,014333543	1,064280437
A_44_P284753	Ankrd1	1,78E-14	0,753266058	1,358304251	1,033036142	-1,381813661
A_44_P287301	Bcl6	1,24E-15	0,792397826	1,048605945	-1,155096905	-1,791673095
A_44_P287958	Dnmt3b	1,30E-24	0,899029071	1,13760354	1,288814617	1,549786379
A_44_P290168	Fah	3,47E-23	0,864288637	1,014737269	1,045953778	-1,658561547
A_44_P290187	Sfrp4	1,89E-13	0,729657452	-1,330006217	1,155482095	1,106584791
A_44_P291028	Arhgef19	1,57E-17	0,837492777	-1,103830851	1,38960773	1,035521638
A_44_P292437	Riox2	4,11E-14	0,727396714	1,272384445	-1,600039343	1,274804706
A_44_P292980	Slitrk1	1,87E-14	0,752840373	-1,201424267	1,010152909	-2,615593621
A_44_P293315	Bbs4	2,28E-09	0,609636454	-1,231874367	-1,176421291	-1,419489611
A_44_P296180	Arg2	1,95E-11	0,655594557	1,272096309	-1,146494932	1,294336629
A_44_P297633	Irx3	2,29E-26	0,896152124	-1,001724441	-1,056303167	-1,715156914
A_44_P298331	Gsdma	2,04E-17	0,795302633	1,29881607	3,109948198	1,231778285
A_44_P302536	Pnn	4,74E-12	0,67365655	-1,140238279	-1,395201752	1,510334809
A_44_P303883	Tbx2	2,92E-15	0,735020012	1,00220923	-1,47223072	1,089515824
A_44_P305801	Jakmip1	6,77E-31	0,935711575	1,03227596	-1,076313144	1,489313735
A_44_P306602	Irf1	4,54E-10	0,63370084	1,045252925	1,002318919	-1,766469806
A_44_P306664	Tbxa2r	2,48E-26	0,895847601	1,219715706	1,004583193	-1,52500394
A_44_P309081	Hspa2	1,47E-15	0,74163209	1,271565695	-1,189990826	1,15365344
A_44_P309455	Fam207a	7,45E-15	0,744415627	1,103441557	1,101345472	1,104152214
A_44_P309780	Dnajb1	2,10E-14	0,6935995	1,007278095	1,084196329	1,172667909
A_44_P311620	Ahnak	2,16E-07	0,617074443	1,18108401	1,049250179	-1,412164701
A_44_P311917	LOC679711	1,48E-21	0,878289098	-1,453204643	-1,245609299	1,354084996
A_44_P313272	Slc6a13	8,95E-12	0,704645209	1,208119477	1,393893532	1,268710055
A_44_P314295	Zmynd12	3,81E-26	0,903423721	-1,359991562	-1,253242283	-1,28604159
A_44_P316319	Shkbp1	4,30E-16	0,770455353	1,186666576	1,032428055	1,406947855
A_44_P316611	Pknox1	8,06E-13	0,673939582	1,007491438	-1,453724646	-1,450037338
A_44_P317388	Mcam	1,57E-22	0,868263472	1,046354179	-1,226287472	1,339838209

A_44_P318662	Tac3	1,31E-32	0,93853331	1,173649211	1,238692827	1,226822899
A_44_P320752	Rasl11a	1,86E-23	0,878340096	1,136878745	1,085417271	1,473853369
A_44_P321329	Polr2c	2,38E-12	0,660636846	-1,039203936	-1,013657139	1,180990132
A_44_P321510	Slc25a33	1,30E-12	0,689261528	-1,328282921	-1,065087052	1,796088614
A_44_P321605		1,07E-13	0,717311696	1,084983723	-1,131945796	1,246857567
A_44_P323404	Cntf	4,28E-13	0,720968323	-1,116366123	1,087594379	1,088913088
A_44_P327992	LOC100360244	1,64E-17	0,796990093	1,104769243	-1,086841911	-1,296315107
A_44_P328046	Masp2	2,35E-23	0,896404427	1,093715569	1,074747556	1,793276562
A_44_P328283	Fan1	1,07E-11	0,641146318	-1,154426789	-1,579571267	-1,177425395
A_44_P328489	Rprd1a	1,39E-09	0,617152777	-1,136501446	-1,243783011	-1,333979781
A_44_P333078	Adamtsl2	2,39E-25	0,896589689	1,092173388	1,656597366	1,67872603
A_44_P335079	Tff3	2,99E-23	0,90386701	1,224182076	1,149996253	-1,208621993
A_44_P335446	Dusp2	1,09E-23	0,899460386	1,22192064	1,285344391	1,452552089
A_44_P337300	Il4r	1,10E-12	0,670133972	-1,000512603	1,45463797	-1,576015365
A_44_P337335	Ctrc	1,63E-20	0,843300817	1,183734077	-1,29096247	1,132099608
A_44_P337351	Cxcl12	3,50E-19	0,824251053	1,056167714	1,004730954	1,339422242
A_44_P341870	Prkag2	2,02E-16	0,77687687	-1,165717224	1,053317029	1,253883142
A_44_P342538	Tcaf1	1,21E-14	0,720787093	-1,076934137	-1,019095428	-1,590476295
A_44_P344181	Map6	1,35E-11	0,66036737	-1,118002179	1,198728061	-1,094785612
A_44_P346832	Tesc	7,40E-18	0,842308436	1,537532749	-1,250791782	1,427581687
A_44_P346913		2,16E-19	0,827387405	1,285989765	1,081824031	1,270010249
A_44_P348812	Hist1h1t	4,23E-21	0,823849193	-1,0242696	-2,123801689	-1,250983621
A_44_P349475	Cdh2	6,95E-10	0,627503551	-1,105676734	-1,216863513	1,023971549
A_44_P351211	Phlda1	3,30E-22	0,864566884	1,014148163	-1,093059223	-1,280439954
A_44_P351723	Cldn10	4,09E-12	0,69536705	1,250154773	1,12816836	1,58265884
A_44_P352268	Plk2	1,52E-09	0,675084427	-1,234232252	1,282534985	1,050634795
A_44_P352331	Bves	2,11E-09	0,631945262	1,096991114	-1,102745632	1,468754091
A_44_P354078	Hist1h1a	2,77E-10	0,640737498	1,029055697	1,032620103	-1,690307506
A_44_P355073	Adssl1	2,49E-10	0,699165401	1,035647004	-1,265078703	-1,161888824
A_44_P356027	Sc5d	1,33E-12	0,667889827	-1,043040594	-1,032859039	1,45649595
A_44_P356829		3,21E-12	0,698217543	-1,10798063	1,195429624	2,52647473
A_44_P357089	Fbxl20	7,37E-14	0,721308545	-1,041865677	-1,216081703	-2,310969149
A_44_P357870	Cpt1a	7,04E-24	0,901147006	1,219731731	-1,557265724	-1,357294138
A_44_P358227	Mapk15	4,26E-12	0,653216844	1,718642301	1,65087371	1,222525959
A_44_P359052	Otub2	5,03E-14	0,70569222	-1,091819947	1,735800627	-1,260969342
A_44_P363291	Kcnk10	1,75E-11	0,657046793	-1,0561582	1,103490061	-1,297194291
A_44_P363549	Nsun6	5,74E-15	0,707558484	-1,039589134	1,020021793	-1,288927735
A_44_P367465	Fzd9	8,11E-13	0,69480948	-1,016777647	-1,026200917	1,71758036
A_44_P369997	Trpm4	1,63E-14	0,717650006	1,097139504	1,050171761	1,468070244
A_44_P370052	Ldhc	2,28E-11	0,711247321	-1,263844948	-1,294677633	2,47435517
A_44_P374708	Cry1	4,72E-11	0,620851193	-1,158626026	1,224189353	1,410585173
A_44_P374824	Klk1c3	3,70E-13	0,703756198	1,614575673	1,865839435	1,952148834
A_44_P375658	Rbm11	4,83E-19	0,835327096	-1,11314729	-1,046316581	-2,038888427
A_44_P378742	Flrt3	2,16E-11	0,711917398	-1,195299291	1,07234795	-1,331081114
A_44_P379891	Pspc1	7,26E-13	0,715175472	-1,001789045	-1,304157758	-1,066379876
A_44_P380182	Mthfd2	2,86E-13	0,686191653	-1,060506101	1,238682326	1,498077344
A_44_P381917	Sphk1	2,65E-13	0,759329845	1,323071777	1,019556064	-1,335816523
A_44_P383899	Fut8	4,75E-11	0,643723832	1,025182197	-1,440702661	1,220788621
A_44_P384090	Des	9,65E-25	0,908487136	1,237391475	-1,14030735	-1,462803618
A_44_P388959	Lrp3	2,30E-16	0,775790499	1,001867184	1,05725296	1,465356689
A_44_P389178	Cracr2b	2,12E-15	0,772731607	1,040681774	1,300964691	1,306928123
A_44_P390385	Phf13	2,31E-17	0,822478042	-1,185292456	1,354939749	1,338034996
A_44_P393696	LOC308990	1,99E-11	0,632833059	-1,077864182	-1,222967114	1,425312238
A_44_P393822	Plekho1	1,80E-25	0,897659543	-1,129735735	1,144261098	-1,348212307
A_44_P398230	Heca	3,44E-17	0,774952238	-1,289875632	1,40903362	-3,547670201
A_44_P399414	Klk1	1,25E-12	0,689786914	1,321996887	1,966538812	1,17705941
A_44_P400324	Maf	6,04E-10	0,62955023	-1,238027808	-1,148864327	-1,496773073
A_44_P401955	Zfp706	6,58E-13	0,697222782	-1,08466084	-1,233447833	-1,01125822
A_44_P402980	Nkx6-2	3,79E-11	0,623935291	-1,006789643	-1,003427387	1,173545732
A_44_P403475	Slc43a2	3,87E-17	0,755640575	1,019278842	-1,319691239	-1,498722643
A_44_P403755	Dusp12	1,73E-12	0,685949379	1,006972099	1,248419722	-1,267955839
A_44_P409518	Gmpr	6,17E-12	0,670366599	-1,044517143	1,281123	-1,200636298
A_44_P409729	Opa1	3,99E-11	0,704555643	1,008544252	-1,113699108	1,154019442
A_44_P409820	Faslg	3,85E-19	0,848772737	-1,004322667	1,085708712	1,375066747
A_44_P409860		4,60E-16	0,752435924	1,142108244	1,013863373	-1,415233389
A_44_P412121	Zfp335	5,81E-12	0,649199256	-1,175562	1,21103442	1,133026889
A_44_P412236	Stag3	7,98E-12	0,620455067	1,011834531	-1,015917599	1,418685563
A_44_P414527	Efna2	1,51E-15	0,775697554	1,087028345	1,285986146	1,116666245
A_44_P416738	Fscn3	2,69E-10	0,619314796	-1,267453026	-1,120027325	1,485325579
A_44_P419676	Zc3hc1	9,17E-11	0,63467522	-1,019835208	1,268842825	-1,101690888

A_44_P419922	Haghl	2,08E-10	0,644775541	1,270172945	1,102694432	-1,449854363
A_44_P420640	Nup43	1,68E-19	0,841844413	1,015718086	-1,003800314	1,157580673
A_44_P421295	Cyp11b3	9,66E-12	0,642537631	1,318460847	1,279111149	2,460589853
A_44_P423490	Palmd	3,41E-23	0,851795304	1,219080724	-1,43003686	-1,788975091
A_44_P424076	Dhdh	5,59E-17	0,787403314	1,198803832	-1,467996176	-1,482527494
A_44_P424723	Sik1	3,26E-18	0,822827036	1,037251835	1,539912765	2,014857335
A_44_P428326	Hmgcs2	6,88E-10	0,605384196	1,300389922	1,804493229	-2,813071457
A_44_P428455	Cygb	3,06E-19	0,7946134	-1,107468873	1,429099338	1,375864409
A_44_P428467	Homez	1,04E-11	0,61674681	1,25831097	-1,207960358	-1,423434196
A_44_P430547	Ntrk1	1,24E-27	0,941252005	1,473962222	-1,506394482	1,356991139
A_44_P430581	Nsmce4a	8,21E-14	0,770501682	-1,062314462	-1,00817956	-1,290575336
A_44_P432314	Fam98a	3,61E-16	0,771952367	-1,08253031	-1,228738096	1,682673102
A_44_P435713	Amy1a	1,98E-15	0,756871488	-1,010543195	1,988362555	-1,953794456
A_44_P436040	Rassf6	9,92E-18	0,840442574	1,406584707	-1,041631285	-4,522497797
A_44_P437594	Gin1	2,94E-16	0,737201508	-1,058511072	-1,415827792	-1,092103728
A_44_P437896	Bdnf	5,01E-18	0,772946166	-1,295005358	-1,015756024	-1,916361812
A_44_P437945	Bbc3	2,60E-20	0,840550173	-1,25028401	1,087802619	-2,561753596
A_44_P437956	Stc1	2,16E-24	0,905588152	1,145535843	1,223492587	1,507652877
A_44_P440558	Wfs1	1,32E-14	0,719859652	-1,027049894	-1,086241276	1,179144511
A_44_P440944		4,56E-28	0,938035432	-1,02160706	1,032836342	1,480867847
A_44_P442780	Kcnab1	9,98E-15	0,789268909	-1,326939073	1,118538202	-1,791769841
A_44_P445031	Fbxo32	6,88E-12	0,669008138	-1,296713759	1,158342978	-4,89276686
A_44_P448051	Vpreb3	3,70E-10	0,711084256	1,021929321	-1,462748836	1,091601022
A_44_P450307	Eps8	5,54E-16	0,784189896	-1,040730697	-1,720175485	-1,945401183
A_44_P450486	Ttll1	2,33E-11	0,711035543	1,093352186	1,24015179	-1,510710499
A_44_P451120	Lmbrd1	3,95E-16	0,753828062	-1,081976401	-1,003126044	-1,251055567
A_44_P456172	Mterf2	9,01E-11	0,656228694	1,141254303	-1,092483613	-1,957685166
A_44_P456599	Zdhhc23	3,20E-10	0,616777708	1,133322103	-1,908560752	1,876855187
A_44_P457099	Plpp5	8,36E-12	0,686753337	1,149024373	-1,24863988	1,36217315
A_44_P457548	Parp9	5,52E-11	0,618641028	-1,001675887	-1,741353012	-1,161020584
A_44_P458021	Tnfrsf11b	1,97E-10	0,645533227	-1,005257528	-1,575133573	-1,369888432
A_44_P459492	Lmo2	1,39E-13	0,672027519	-1,161344303	-1,058982182	1,423130439
A_44_P461383		7,45E-16	0,796498063	1,005802588	-1,791511991	1,351645105
A_44_P463488	Amh	1,46E-19	0,829916594	1,145500747	1,202801773	1,222098466
A_44_P465880	Snap47	6,49E-16	0,749277157	1,09792387	-1,02566397	-1,269718048
A_44_P466434	Fam212b	5,57E-13	0,699130704	1,053185096	1,308425952	1,415518629
A_44_P467202	Pcdh8	8,84E-22	0,859486932	-1,029493763	-1,144814581	1,155276434
A_44_P468239	Sgtb	5,03E-13	0,700290857	-1,292048208	1,574488423	-1,582641672
A_44_P470528	Ndufaf4	1,58E-14	0,754423021	-1,01772108	-1,224260766	1,901323909
A_44_P472661	Klf2	3,68E-18	0,821999782	1,135162346	-1,140471425	-1,476413997
A_44_P473234	LOC102554302	1,00E-12	0,692362996	1,081652368	1,109821518	-1,322038841
A_44_P474101	Slc7a1	4,63E-21	0,862130684	1,067901113	-1,082627725	1,459783733
A_44_P475406	Hsf1	4,00E-16	0,771082503	1,032911359	1,053509045	1,401047385
A_44_P477406	Slc2a4	2,30E-15	0,755484697	1,051092932	-1,027345521	-1,259111085
A_44_P477568	Sdccag8	3,57E-12	0,655471087	-1,027172191	1,208350646	-1,113144319
A_44_P479942	Kcnk15	8,01E-21	0,819763466	1,073214967	1,394706804	1,29095481
A_44_P480623		6,53E-24	0,901435277	-1,017337521	-1,028673154	1,231305624
A_44_P480696	Ap1s2	1,12E-18	0,801536666	1,025905447	-1,054843162	-1,643972026
A_44_P480925	LOC691995	5,96E-13	0,677559542	1,030037484	1,241428956	-1,023689159
A_44_P481892	RGD1309079	2,36E-13	0,727329462	-1,14976936	-1,213562053	-1,332249138
A_44_P481915	Id4	1,87E-13	0,762673314	1,031216355	-1,441177726	1,180128502
A_44_P483728	Rps6ka1	1,48E-11	0,636844124	1,012829864	1,14292778	1,195785896
A_44_P484836	Tjp2	3,17E-23	0,864749593	-1,18610976	-1,166231706	-1,366151802
A_44_P484984	Gnmt	1,86E-11	0,656262525	1,205745542	1,289569904	-1,183715895
A_44_P485388	Chd7	1,59E-14	0,736966676	1,130659901	1,020664578	-1,359651911
A_44_P489688	B3gnt8	1,51E-15	0,721286733	1,084720161	-1,101752882	-2,101800766
A_44_P491393	Nudt7	7,93E-10	0,603238402	1,028774437	-1,075442246	-1,590113316
A_44_P493005	Foxq1	1,59E-12	0,724339605	1,198875657	1,407706734	-6,097260563
A_44_P493980	Nptxr	1,79E-17	0,810859116	1,299630251	-1,255519269	1,376654852
A_44_P497174	Gramd1b	1,31E-09	0,618084755	-1,128249094	1,091799208	-1,342119913
A_44_P497193	Senp8	4,73E-16	0,732648014	1,027965823	-1,214726616	-1,205764392
A_44_P500431	Lman1	4,50E-09	0,640632932	1,052552529	-1,020568607	1,269945003
A_44_P501051		1,52E-21	0,856594367	1,053701795	1,208422784	-1,139250813
A_44_P501761	Crybg2	4,81E-19	0,835342262	1,539744452	1,339053194	1,197146407
A_44_P501827	Cpsf6	1,59E-10	0,626948053	-1,232028406	-1,035630567	1,151734315
A_44_P503669	Zbed5	6,19E-23	0,848591535	-1,06336288	-1,214753314	-2,832143939
A_44_P503699	Sergef	2,07E-20	0,841896053	1,097734371	1,10025833	1,05561152
A_44_P504750	Rassf9	3,33E-09	0,603712828	1,146466993	1,126474332	-1,274281382
A_44_P506299	Bcl2l15	4,94E-26	0,918455181	1,284473058	-1,666216882	1,317569807
A_44_P506635	Ifrd2	4,35E-13	0,701927746	-1,025482452	-1,001711068	1,430899622

A_44_P507571	Ccne1	5,43E-16	0,731334437	-1,08135139	1,464312754	2,168799025
A_44_P508055	Bcl2l11	5,58E-12	0,625313283	1,048901446	1,233049597	-2,731230284
A_44_P508691	Tm6sf2	1,36E-28	0,92170148	1,080492456	-1,028265622	1,245593629
A_44_P512136	Dhcr7	3,10E-23	0,864846271	1,094898512	1,211112678	1,523627783
A_44_P513385	Dab2	4,46E-08	0,62424575	-1,125627063	-1,084168559	-1,270147439
A_44_P514927	Fam69b	2,41E-15	0,755074798	1,008960762	1,21611405	1,277719098
A_44_P514987	Tshz1	3,77E-14	0,728278237	-1,17428739	-1,344080815	-1,35805791
A_44_P517033	Rab11fip5	5,63E-12	0,649609423	-1,018308056	1,007277595	1,451559889
A_44_P520700	LOC501266	7,15E-18	0,830451337	1,043568621	-1,507877951	1,014421551
A_44_P520929	C1qtnf1	6,41E-11	0,616536438	1,452404867	1,760518735	-3,1003503
A_44_P522047	LOC684871	3,96E-12	0,747354883	1,109198474	-1,800500155	-1,294810437
A_44_P527270	Pth1r	6,13E-12	0,648504846	1,188772178	-1,019717481	-1,296841703
A_44_P529240	Rhbg	1,23E-18	0,784103033	1,311206671	-1,386211521	-1,553522676
A_44_P529904	Bend7	1,17E-11	0,662298694	-1,059041423	1,64457077	-2,226977413
A_44_P530131	Asb2	2,05E-30	0,933013633	1,208506054	-1,286813789	-1,549606059
A_44_P532249	Fkbp5	2,06E-12	0,783155091	1,203286879	-1,142524589	1,459264901
A_44_P532476		4,28E-19	0,822925587	1,018636007	-1,512978871	-3,05588455
A_44_P533560	Fxn	2,60E-11	0,629168785	1,029438043	1,293466568	-1,143017681
A_44_P534089	Ccnb1	2,07E-11	0,654840673	-1,200140557	1,130900577	1,163729861
A_44_P537652	Arid2	2,25E-15	0,772237761	-1,045262249	-1,045350434	1,187528387
A_44_P538165	Tspan17	4,15E-11	0,62265911	-1,032536406	1,351068545	-1,054317362
A_44_P538870	Calhm2	5,64E-14	0,682495116	-1,011582402	-1,282474153	-1,396043794
A_44_P538970	Ptpdc1	2,16E-13	0,709742604	-1,032812699	-1,022846768	-1,503464137
A_44_P540517	RGD1564664	2,62E-13	0,707625437	1,08743164	1,330400799	-1,248378789
A_44_P541020	B3galt4	4,39E-17	0,789320196	1,126026523	1,092019895	-1,273971532
A_44_P543917	Fbxo42	2,47E-11	0,673238871	-1,153026838	1,200851995	1,017609398
A_44_P545193	Hsd11b2	5,46E-12	0,671913136	-1,074852973	1,555883522	1,464125782
A_44_P545887	Ccdc155	3,73E-19	0,809379286	1,109665082	1,185798527	-1,276191385
A_44_P547771	Cited1	2,22E-22	0,854766183	1,213827112	-1,010936402	1,271658199
A_44_P548241	Zfp51	7,81E-15	0,72522987	1,000712934	-1,703856635	-1,407252245
A_44_P548420	Pmvk	7,54E-17	0,76835875	1,037928375	1,089471083	-1,031613173
A_44_P548426	Ankrd35	1,36E-10	0,650645321	-1,188993024	2,340784903	-1,593051194
A_44_P548590	Ribc1	1,82E-11	0,608978315	1,175846845	-1,134391777	-1,568725568
A_44_P548726	Zfp414	6,88E-21	0,835318762	1,158675339	1,189074175	-1,351250171
A_44_P548730	Bcl9l	7,17E-12	0,646463772	1,070012322	1,022247461	-1,426133156
A_44_P553017	Gmpr2	2,71E-12	0,658964626	-1,147749052	1,13475768	1,102603595
A_44_P553341	Lrfn5	2,04E-14	0,693896301	-1,270495724	-1,309976926	-1,15938547
A_44_P555253	Dnaja1	4,26E-21	0,838175629	1,03393913	-1,046882585	1,144569557
A_44_P556319	Fgf18	1,48E-16	0,762557242	1,199629754	-1,085066565	1,431848775
A_44_P556989	Ngfr	2,58E-16	0,830563218	1,583322795	-1,045833911	1,438599592
A_44_P557376	Drd1	5,82E-12	0,624751147	-1,4531028	-1,583619265	-3,745614956
A_44_P558411	Scrg1	8,70E-10	0,601824044	-1,090506472	1,329306778	-1,259150726
A_44_P576822	Inhbb	1,46E-26	0,906816725	1,226532107	1,106151529	1,227412686
A_44_P577108	Rasgef1c	3,08E-20	0,839534473	-1,019663482	1,109617775	1,16262344
A_44_P579382		4,25E-12	0,746599062	1,362497894	-1,121829137	1,13163175
A_44_P586876	Tmem41a	1,07E-10	0,632485039	1,032847348	1,168581814	1,48508468
A_44_P588645	Rrp1b	9,64E-16	0,745598983	1,045198428	-1,077232546	1,65159726
A_44_P592627	Samd10	1,12E-10	0,608508544	1,017215972	1,126318804	1,214382697
A_44_P606049		5,41E-11	0,61893277	1,166290678	-1,067145675	-1,806701617
A_44_P607542	Btbd11	1,80E-14	0,753158724	-1,035107927	-1,927479118	1,325078427
A_44_P607583	Trmt61a	6,31E-12	0,648135695	1,103735726	-1,103928451	1,893222634
A_44_P608167	Tmem116	5,06E-23	0,87372165	-1,095909898	-1,442456543	-1,437493268
A_44_P612186	Mylip	4,80E-24	0,873756372	-1,141975089	-1,084244797	-1,203273751
A_44_P612762	Lrrc8c	2,84E-12	0,679967328	-1,093070088	1,222820593	1,185559073
A_44_P613170	Ndp	2,34E-15	0,771887242	1,082621552	-1,2076398	-1,746703314
A_44_P620106	Tgfbi	5,69E-11	0,64127927	1,057898577	-1,096122181	-1,366007562
A_44_P623083	Slx1b	9,64E-13	0,671780522	1,011101058	-1,070244695	-1,427214275
A_44_P623694		9,68E-16	0,763336593	1,070419718	1,020637214	-1,226532728
A_44_P625333	Ctdspl	6,90E-12	0,668977549	1,016990399	1,065271619	1,263330612
A_44_P628515	Rgs7bp	4,46E-14	0,77610683	-1,285232029	1,020355952	-1,637848347
A_44_P634770	Cxxc5	9,77E-22	0,846672796	1,041857762	1,077838441	1,122778793
A_44_P635769	Mks1	1,36E-12	0,667595728	1,016873012	-1,14960658	-1,333792184
A_44_P638290	Zfp467	1,10E-21	0,846028823	1,026957395	1,143996955	1,493132743
A_44_P638511	Paqr5	2,82E-26	0,927060066	-1,003691166	-2,052475783	1,400279478
A_44_P639365	Col24a1	1,30E-10	0,723900863	-1,002407087	-1,242013069	1,238702229
A_44_P668185	Endod1	5,03E-11	0,66400832	1,047315712	-1,20478858	1,262370914
A_44_P671089	Foxc2	2,03E-12	0,683990762	-1,016103562	1,468229849	2,118508034
A_44_P671340	LOC498601	2,56E-11	0,60415737	1,12847873	-1,002722129	-1,553844307
A_44_P680749	RGD1565033	5,85E-14	0,682070297	1,048455275	-1,036255529	-1,536953322
A_44_P684795	Irs2	8,87E-14	0,719343757	-1,135217404	-1,261147746	-1,9797754

A_44_P685298	Ice2	4,79E-09	0,619514777	-1,0327481	-1,242157274	1,163322032
A_44_P686406	RGD1562310	4,17E-26	0,893851035	1,044806907	1,186810525	1,161097159
A_44_P714007	Cidec	1,89E-19	0,798135703	1,243219838	1,213420121	-1,676250195
A_44_P715030	Vstm2a	1,68E-11	0,6101296	1,074070714	1,530268861	1,334661372
A_44_P720576	Lrrn4cl	6,79E-10	0,605587991	1,111093541	-1,510612029	1,407780366
A_44_P727824	Ppp2r5a	8,63E-14	0,737747517	1,17617694	-1,158240941	1,278490907
A_44_P728793	Tppp2	9,30E-12	0,618358144	1,422941903	-1,513553436	-2,766932904
A_44_P729856	Ppp1r3d	1,69E-14	0,811774028	1,036561942	1,249512722	1,577720163
A_44_P732517	Zadh2	8,01E-19	0,787400204	1,219155678	-1,460991673	-1,267169055
A_44_P745585	Tprn	2,02E-19	0,797634295	-1,00240526	1,203692219	1,281330904
A_44_P745710	Olfml2a	1,20E-12	0,669102548	-1,151617925	1,135479799	-1,482173052
A_44_P760697	Nhej1	1,25E-10	0,630306565	1,379668103	1,212819723	-1,3744515
A_44_P761066	Atg5	1,43E-10	0,604957201	1,018713956	-1,131724335	1,110234723
A_44_P770253	Rfx8	5,65E-10	0,608359276	1,076509402	2,738757443	-1,943720528
A_44_P777016	Ypel4	6,62E-19	0,833322469	-1,144104725	1,030762459	-3,03103214
A_44_P777424	LOC100362783	2,89E-13	0,75845308	1,061530891	-1,390520289	1,031939606
A_44_P781945	Tbx4	6,74E-11	0,638953169	1,244203742	2,242265541	1,057596861
A_44_P787936	Stat3	2,52E-15	0,771216365	1,030475132	1,166694352	-1,295018942
A_44_P792628	Spef1	1,31E-09	0,618064807	1,152741721	-1,353937675	1,037363001
A_44_P793551	Lin28a	2,34E-18	0,779071027	-1,068369696	-1,116623892	-1,956626247
A_44_P794669	Afdn	2,34E-14	0,733131714	-1,225423451	-1,026107819	1,494179362
A_44_P807585	Prcd	1,45E-30	0,933870119	-1,022004388	1,15932933	1,240223903
A_44_P809486		7,19E-22	0,8605643	1,131724986	-1,524933894	1,047190707
A_44_P810179	Acd	7,04E-29	0,930486502	1,017689127	1,136009192	1,149155236
A_44_P810190	Srrt	4,87E-13	0,679948926	-1,095725186	1,05615436	1,356246627
A_44_P817760	Tspan15	1,29E-22	0,857608887	-1,091474647	1,435080723	1,186696985
A_44_P819401	Slc40a1	8,16E-13	0,731564894	-1,317534062	-1,62728378	-1,565591686
A_44_P822447	Ctla2a	3,25E-15	0,784336771	1,285749417	-1,022489069	-1,699517715
A_44_P823749	Scn5a	6,78E-10	0,648517067	1,123216505	-1,246079228	1,613759032
A_44_P832655	Dedd2	2,23E-09	0,63113308	1,087505232	1,265269969	-2,511358065
A_44_P838583	Psrc1	5,16E-21	0,837036103	-1,029711895	-1,022502716	-1,516251108
A_44_P847319	Luzp2	4,17E-14	0,776710041	-1,431640085	-1,016418627	-1,567178499
A_44_P852017	Gpr146	1,13E-12	0,669857625	1,035571869	1,362162073	1,000443158
A_44_P852545	Tmem196	9,58E-22	0,870172597	-1,183034347	1,405287623	-1,426345515
A_44_P853916	Slc43a2	2,48E-26	0,90494715	1,020096192	-1,567542433	-1,446207136
A_44_P868890	Gli2	1,86E-17	0,810594262	-1,080721379	-1,297392753	1,39136892
A_44_P876363	Vav2	9,13E-24	0,935240588	1,320266416	-1,279888968	1,661184137
A_44_P878711		2,90E-09	0,605912324	1,434134278	-1,623499758	-2,341541488
A_44_P883462		6,32E-24	0,872485356	1,009857304	-1,554341921	-2,345849388
A_44_P884080	Smim11	9,83E-12	0,617586413	1,03264357	1,152696099	1,078056843
A_44_P885003	Arap2	1,75E-11	0,609504005	-1,242384955	-1,209727251	-1,332736991
A_44_P885889	Fam81a	6,92E-15	0,745126607	1,13524226	-1,193362612	1,128345321
A_44_P886690	Lpar2	7,73E-11	0,613850435	1,153600528	-1,194373019	1,945999932
A_44_P898436	Lrrc17	2,68E-16	0,790137703	-1,244272411	-1,278417839	-1,372246729
A_44_P899127	LOC500300	1,72E-31	0,944662039	1,037951613	-1,617665277	-2,419647102
A_44_P899293	Snai1	7,45E-20	0,834139402	1,199670599	1,020695106	1,357288145
A_44_P899938	Fam124a	1,53E-12	0,706827724	1,030080966	1,061783274	1,227223675
A_44_P901088	Ccnb1ip1	9,92E-13	0,671428741	-1,174346981	1,054396383	-2,208825063
A_44_P913774	Cct8l1	5,67E-13	0,654966534	1,084511687	-1,398739373	-1,752749994
A_44_P914607	Plxnd1	4,06E-12	0,629608933	-1,088874029	1,072240564	1,349692756
A_44_P917327	Lrtm2	8,31E-32	0,946166942	1,106703993	1,208454027	1,853116985
A_44_P917564	Mat2b	1,32E-15	0,77687816	-1,078614976	-1,055693767	-1,903648597
A_44_P929423	Itga9	2,84E-16	0,774004621	1,156315332	-1,350884347	1,203432163
A_44_P930152	Mpped1	1,28E-22	0,898155721	1,33016812	-1,352025976	1,966836686
A_44_P940501	Tnrc6c	1,28E-11	0,638767333	-1,180382824	-1,272800555	1,478852227
A_44_P944353	Armc2	5,47E-13	0,699333674	-1,097300252	-1,052272954	-2,18543141
A_44_P945404	Zfp703	4,19E-16	0,753285216	1,147467315	1,235274763	1,398612447
A_44_P956272	Ogt	2,08E-14	0,715122327	-1,045302323	-1,379230525	-1,120542742
A_44_P960146	Gga3	1,39E-14	0,69811416	-1,0394767	1,06432509	1,17305536
A_44_P962701	Nol4l	1,50E-20	0,855786916	-1,022817273	-1,10240754	1,359175827
A_44_P963812	Ctnnal1	3,33E-10	0,616225652	-1,076958256	-1,129756823	1,512034649
A_44_P964460	Itk	2,89E-12	0,634113597	1,240377774	2,359243617	-2,095557488
A_44_P974651	Tmem74b	1,26E-24	0,86830854	1,096963559	1,003641572	1,656738873
A_44_P975327	B3gnt2	1,98E-11	0,655449337	1,057087866	-1,257387489	1,376369779
A_44_P975731	Zfp292	1,14E-12	0,669730557	-1,117226826	-1,03688601	1,183409389
A_44_P989558	Slfnl1	3,36E-12	0,677914856	1,008972019	1,43005001	1,187167342
A_44_P990713	Rrp9	8,57E-15	0,703311267	1,154856511	-1,032660122	1,262700774
A_44_P991472	Rnf39	3,05E-22	0,875724014	1,01767133	1,482668375	-1,211932594
A_44_P997843	Csrp3	5,34E-20	0,848630737	-1,082623253	-1,12984122	-1,388843105
A_44_P998638	Pprc1	1,65E-17	0,780920463	-1,01292678	-1,160104001	2,253865133

A_44_P999125	Masp2	1,44E-18	0,852322783	1,006647305	1,144663097	2,885306637
A_44_P999395	Lmbr1l	2,06E-09	0,688606121	-1,17499486	1,274847253	-1,982806996
A_64_P000136	Slc35f2	1,40E-13	0,645970655	-1,106705941	1,144744823	1,244730634
A_64_P000171	Dhcr24	6,60E-17	0,750920825	1,170642182	1,038830995	2,064035909
A_64_P000281	Nkain4	1,30E-11	0,613671846	1,067242339	1,228267283	1,048056759
A_64_P000386	Isl2	2,32E-09	0,609365796	1,747793614	1,638216477	-1,242045022
A_64_P000708	Rnf125	2,15E-13	0,689470927	-1,042582956	-1,206912077	-2,272468316
A_64_P000895	LOC108348336	8,74E-15	0,742869556	1,115809387	-1,393480583	1,135627964
A_64_P001219	Mthfs	7,29E-10	0,647495278	1,225733741	1,137987018	-1,256228323
A_64_P001344		2,46E-13	0,687933459	-1,094050583	1,002699342	-1,607966294
A_64_P001364	Prickle1	4,79E-17	0,753771174	-1,331018976	-1,319182906	-1,349681446
A_64_P001461	Dnmt3l	3,55E-19	0,809720541	1,335012333	1,073187834	-2,013439552
A_64_P001799	Fam110d	5,67E-11	0,641323103	1,191978133	-1,165629569	1,628965049
A_64_P002110	LOC363337	1,24E-20	0,867749973	-1,235819943	-1,394517013	-1,001784434
A_64_P002339	Zbed4	8,75E-11	0,676383332	-1,286473661	1,137387922	2,157550832
A_64_P002809	Grhl1	4,70E-22	0,850720237	1,409666902	-1,294714974	-1,152367198
A_64_P002899		2,34E-13	0,744546856	1,298008526	1,292678312	2,454368003
A_64_P003261	Tbc1d5	1,71E-14	0,695864169	-1,102450903	-1,352072104	-1,385367492
A_64_P003378	Tuba3b	5,26E-12	0,728128832	1,123848073	1,372935833	-1,423904053
A_64_P004007	Rd3l	1,11E-28	0,922281475	-1,199546896	1,188867369	-2,021906383
A_64_P004307	Pipox	1,29E-12	0,689378333	-1,022511706	-1,097895833	-1,689194685
A_64_P004344	Hist2h2aa2	1,05E-15	0,744784296	1,041930934	-1,165118061	-1,19320568
A_64_P004406		1,38E-14	0,755725398	1,034377243	1,160391715	1,745654489
A_64_P004539	Rnase12	5,75E-29	0,907907262	1,637206954	-1,533675006	-2,438555994
A_64_P004584	Cacna1g	2,31E-30	0,925774713	1,037137504	1,060507152	1,411679809
A_64_P004702	Akap3	1,41E-26	0,88786419	1,45187324	-1,061969373	-1,618403113
A_64_P005987	Agfg2	2,34E-13	0,727431096	1,045174847	-1,027884963	1,835739515
A_64_P006285	Lhx1	6,78E-19	0,805151492	1,018817934	-1,416085191	1,196135144
A_64_P006473		1,55E-23	0,889047561	1,100188509	-1,375390695	1,014777474
A_64_P006478	Slc25a34	1,30E-20	0,831444365	-1,040907865	1,158418779	1,223245351
A_64_P007163	Mybphl	7,29E-17	0,750024395	1,545657607	1,261462076	-1,826449418
A_64_P007508	Csf3	7,82E-16	0,747555044	1,255133611	1,437544544	1,233427229
A_64_P007783	LOC103691261	6,80E-12	0,669146417	-1,221863299	1,292972463	-1,234969627
A_64_P007793	Prob1	7,57E-16	0,765518502	1,329481111	-1,814491577	1,086288402
A_64_P008042	Ankrd9	1,20E-13	0,696139182	1,02315454	1,074576259	1,808331357
A_64_P008747	Stambpl1	1,21E-13	0,734258895	-1,098430571	1,027671632	1,221644534
A_64_P008777	Ccdc28a	1,10E-19	0,831703174	-1,047243195	1,097416133	-4,416790796
A_64_P009355	Reep4	9,78E-15	0,758926133	1,14766996	1,099899372	1,396566244
A_64_P009565	Vmo1	2,01E-24	0,913639105	1,064972923	-1,250157367	-1,488662343
A_64_P009718	Btbd17	6,71E-36	0,969565118	1,350058254	1,002855608	-1,249140278
A_64_P009738	RGD1559714	1,42E-18	0,81477548	1,032815853	1,783802115	-1,268550014
A_64_P009803	Btg2	1,49E-24	0,945382706	-1,042345819	1,242701604	-1,303407743
A_64_P009857	Tbx1	1,30E-17	0,813192721	-1,090005134	-1,094662587	1,546634875
A_64_P009892	Lbx1	1,31E-13	0,733490937	-1,001527337	1,071421291	1,439130429
A_64_P009897	Ppp2r1b	2,43E-22	0,895521942	-1,086157456	-1,30474686	-1,494873215
A_64_P009996	Tac1	2,07E-15	0,772945611	1,102562409	1,266572725	-1,89101583
A_64_P009999	Tac1	2,64E-11	0,651621671	1,109260798	-1,216273031	-1,87935104
A_64_P010037	RGD1309139	6,96E-11	0,615343878	-1,348971707	1,706811755	-1,12433943
A_64_P010234	Gys1	3,35E-18	0,79338216	-1,15018537	1,039789907	1,41733986
A_64_P010269	LOC108351885	2,93E-12	0,633915252	-1,002120208	1,046765811	1,142787071
A_64_P010298	Sh2d3c	9,29E-19	0,854869571	-1,216344791	1,987383455	-1,063267301
A_64_P010960		4,51E-15	0,781524476	-1,03117801	-1,301981274	1,258550643
A_64_P011045	Hrh3	2,89E-13	0,70652162	1,0079978	1,213467492	1,267214475
A_64_P011654	Zfp296	2,33E-18	0,825098792	-1,057697978	-1,06391676	-1,275190689
A_64_P011979		6,38E-12	0,669956291	1,036839912	1,279335665	-1,02562108
A_64_P012198	Agrp	3,52E-13	0,72306365	1,37528217	-1,681457682	1,007132152
A_64_P012328	Slc35d1	2,31E-14	0,692516613	-1,714072664	-1,562657244	-1,284811223
A_64_P012902	Sdf2l1	6,30E-22	0,849113724	1,130337246	1,077806376	1,313402285
A_64_P012972	Tecta	1,13E-12	0,669820811	-1,21004766	1,443102335	-1,2507915
A_64_P013288	Stmn4	1,19E-14	0,720942701	-1,185268565	1,68141126	-1,031169145
A_64_P013398	Gne	4,15E-13	0,658829194	-1,095822962	1,040792362	1,14348133
A_64_P013402	Rab33a	3,20E-17	0,820192329	-1,129388714	1,193670549	1,039117573
A_64_P014159	Plpp5	4,01E-10	0,613463501	1,105274911	-1,196589485	1,291739064
A_64_P014178		4,09E-11	0,686125166	1,113303282	-1,33682216	1,208491788
A_64_P014356	Map2k6	8,11E-17	0,826410798	1,376722493	-1,179888025	-1,033967257
A_64_P015479	Rfc5	6,77E-16	0,748895626	1,071531105	-1,005374654	1,282783729
A_64_P015549	Lyl1	2,39E-13	0,72718632	1,073749042	-1,069962983	1,445288176
A_64_P016578	Rassf7	1,11E-12	0,646484111	1,086359491	1,303856267	1,207726323
A_64_P016746	Lhx4	7,10E-15	0,726194473	-1,475895819	1,114164752	-1,72467478
A_64_P016751	Tmem243	4,59E-11	0,621242793	-1,117392733	1,417128711	-1,262783732

A_64_P016766		5,58E-14	0,758674546	1,468674715	-1,023698546	-1,727389997
A_64_P016828	Myo1b	3,33E-12	0,67801528	1,037367114	-1,307681746	-1,00618164
A_64_P017243	Tbc1d8	7,64E-18	0,802714699	-1,528968921	-1,025407973	-1,368075642
A_64_P017363	Slc30a3	3,23E-19	0,810372711	1,089662123	1,038062	1,20764828
A_64_P017758	Morn2	4,68E-17	0,788810201	1,114076935	1,175500431	-1,382894118
A_64_P018427		3,48E-11	0,668823578	-1,259772532	-1,109560289	-1,733591278
A_64_P018633		1,75E-16	0,807780768	1,305919891	-1,478010327	1,681221359
A_64_P018761	Calca	4,61E-10	0,611412326	1,004285563	1,338609799	-1,183949183
A_64_P019016	LOC501396	3,20E-11	0,707231675	-1,123114332	-1,953586004	1,475224524
A_64_P019081	LOC690490	6,48E-14	0,722665265	-1,415507752	-1,528311729	1,072933454
A_64_P019130	Ccpg1	2,03E-17	0,795334103	-1,101222898	1,258319384	-1,732998433
A_64_P019135	Ccpg1	1,54E-17	0,781479037	1,05187758	1,031995713	-2,290908122
A_64_P019175		2,07E-24	0,905733367	-1,077360816	-1,457979028	1,091865235
A_64_P019200	RGD1560608	7,27E-11	0,63790576	1,159302804	1,669908414	1,903403699
A_64_P019280	Pik3ip1	1,24E-30	0,927424846	-1,041638256	-1,228981788	-3,989273897
A_64_P019401	Sdf2l1	1,02E-22	0,890320788	1,296481266	-1,020326863	1,264239836
A_64_P019673	Arhgap20	3,50E-23	0,885557752	-1,26721072	-1,010087967	-1,402546789
A_64_P019756		1,25E-13	0,715716683	1,285717022	3,10631234	-1,782292842
A_64_P019836	Ceacam18	2,87E-19	0,795093117	-1,13600891	-1,362151197	-1,123475996
A_64_P020154	Plxnb2	7,67E-13	0,695463468	1,094830662	-1,10373874	1,260354519
A_64_P020238		7,49E-10	0,647100765	1,148803285	-2,027191643	-1,467533666
A_64_P020269	Hist1h2bh	1,26E-21	0,845228885	1,453153101	-1,39520262	-1,501044138
A_64_P020471	Lrrc3b	9,67E-20	0,832517867	-1,238200314	1,144027015	-1,420950827
A_64_P020621	Dlk2	4,29E-10	0,612470419	-1,106093052	1,28125068	1,564233461
A_64_P021206		1,97E-19	0,813793561	1,080108061	-1,265755471	-1,564132906
A_64_P021241	Nradd	2,43E-10	0,620824386	1,107830761	1,082227212	1,38235593
A_64_P021320	Pqlc1	8,60E-14	0,677637477	-1,083842514	1,183997616	1,225091775
A_64_P021428	Slc16a6	5,85E-14	0,723729836	1,098179382	1,051634415	1,924974827
A_64_P021521	Plekhh3	7,26E-13	0,651886667	-1,006485547	1,233959783	1,950351803
A_64_P021686	Epor	2,41E-11	0,673511922	1,159880766	-1,897376179	1,727986468
A_64_P021702		8,04E-11	0,657763379	1,050146073	-1,191765509	1,334638762
A_64_P021845	Car7	2,47E-29	0,92651594	-1,231798665	1,419778882	-1,171826353
A_64_P022015	Sstr2	2,56E-24	0,887029972	1,452963076	-1,132779004	1,655328215
A_64_P022155	Slc2a5	2,89E-13	0,663262752	-1,055141387	1,086469466	1,658420437
A_64_P022813	Exosc10	9,90E-16	0,74535753	1,160521632	1,122406647	1,439003584
A_64_P022913	Nat8f1	9,45E-18	0,767709924	-1,155117787	-1,346068604	-2,564361564
A_64_P022943	Rara	3,09E-25	0,904406059	-1,146957553	1,279313788	-1,001085988
A_64_P023179	Olr1388	1,16E-11	0,701619923	1,071016206	1,113346806	-2,778059174
A_64_P023381	Col6a5	1,57E-10	0,64862935	1,036169993	-1,307911433	-1,252333294
A_64_P023561	Cox10	9,03E-13	0,69357435	1,027905687	-1,049179695	1,249364663
A_64_P024112		3,47E-11	0,647969625	-1,040984415	-1,1808567	1,206380614
A_64_P024356	Sh2b2	2,65E-13	0,687097801	1,09777002	1,024971542	1,865359526
A_64_P024474	Stum	2,84E-18	0,83647863	1,107135825	1,223369366	-1,153055136
A_64_P024813		2,35E-13	0,665742395	1,220684037	1,131863776	-1,628748624
A_64_P025050	Ccl1	3,87E-16	0,850541594	2,605745411	-1,546653132	-1,677290408
A_64_P025098	Cebpg	2,05E-16	0,819834489	1,011642667	1,156410121	-1,038306803
A_64_P025118	Cebpb	3,07E-14	0,819479878	-1,207637683	1,386927707	1,158207176
A_64_P025169	Phlda2	1,78E-18	0,826897445	-1,032289362	2,289071824	1,867712117
A_64_P025173	Phlda2	4,64E-17	0,788881894	-1,340431216	1,848318716	1,220727257
A_64_P025248		1,83E-10	0,601296828	1,101730224	1,105154178	3,769796771
A_64_P025325	LOC501396	7,42E-18	0,80292841	1,082021908	-1,156025959	1,278563364
A_64_P025496	Maff	1,67E-10	0,686563268	-1,078829524	1,141856496	-3,203841974
A_64_P025599	Eif4g2	2,35E-09	0,60912454	1,000303462	-1,18994216	1,183934223
A_64_P025618	Slc25a35	1,17E-12	0,709857273	1,121775609	-1,127702149	-1,449926471
A_64_P025678	Tbc1d16	1,53E-18	0,851939912	1,173217235	-1,024532166	1,420624331
A_64_P025963	Ccnf	9,99E-11	0,674649903	-1,120822954	1,446219015	1,113165954
A_64_P026297	Insl6	2,61E-24	0,904885744	1,737793586	-1,401591713	-1,588281795
A_64_P026883	Ick	1,43E-11	0,637285975	-1,143878999	1,551366496	-1,740349331
A_64_P027530		2,89E-14	0,711662726	1,005469117	1,211014404	-1,077242422
A_64_P027565	Zfp451	6,97E-13	0,675691315	-1,056693474	-1,092792563	1,211476987
A_64_P027707	Erich4	2,65E-15	0,735946252	1,051892344	-1,109854536	-1,979993467
A_64_P027847	Fer	6,54E-11	0,732024483	-1,172079887	1,195338206	-1,018853611
A_64_P027911		4,24E-18	0,821032908	1,690520499	-1,244193608	1,288243131
A_64_P028290	Yrdc	4,30E-27	0,910958362	1,128077942	1,08989805	1,287661377
A_64_P028379		1,59E-12	0,706344701	-1,045168887	-1,833840786	-2,675375493
A_64_P028788	Mcrip2	2,13E-14	0,714876369	1,000721616	1,231353135	1,290802503
A_64_P029077		5,18E-11	0,701352264	1,230381742	3,149916838	1,258252095
A_64_P029213	H1f0	9,58E-14	0,676378639	1,336106085	-1,295327371	-1,363790745
A_64_P029337	F8a1	1,65E-08	0,620535661	1,045930616	1,125437899	1,011891468
A_64_P029482		4,36E-11	0,62197362	1,169587453	-1,077079552	-2,319302502

A_64_P029501	Tnnc1	2,40E-17	0,82220434	1,046319339	1,227975226	-1,321244476
A_64_P029805	Junb	1,32E-21	0,868576206	-1,023644521	1,345485478	-1,360492968
A_64_P029887	Focad	5,14E-14	0,742969187	-1,121691726	1,667997735	-1,640950705
A_64_P030090		1,29E-13	0,672900604	-1,198686755	-1,31538166	-1,674509313
A_64_P030362	Ephb3	1,55E-21	0,856489752	1,368609939	-1,279975273	2,112174222
A_64_P030534	Tpcn2	3,46E-27	0,926439896	1,373415099	-1,031898549	1,47903798
A_64_P030614	Nyx	1,30E-12	0,668080894	1,040919904	-1,012913305	-1,62327843
A_64_P031046	Draxin	1,88E-15	0,757353941	1,068399346	1,029374843	-1,618035207
A_64_P031190		5,58E-21	0,822086901	1,144112283	-1,26556795	-1,383608738
A_64_P031476	Slc26a9	3,81E-11	0,623857616	2,372258721	1,536105805	-1,772809515
A_64_P031511		5,77E-21	0,871651749	-1,083441554	-2,026763876	1,157564754
A_64_P032127	Ttll7	4,24E-11	0,685651324	-1,370911637	1,138369134	-1,162129539
A_64_P032358	Mustn1	3,28E-15	0,811870887	1,178125687	1,154548512	-1,374720892
A_64_P032574	Hk2	4,56E-11	0,621340636	-1,022671251	-1,188306182	-1,425794736
A_64_P032806	RGD1308106	1,81E-12	0,704846971	-1,03816632	-1,007069954	1,407835734
A_64_P032836	Rprm	1,42E-21	0,844577352	-1,01609785	1,100486006	-1,294667205
A_64_P032904	Mbnl2	9,58E-18	0,801030513	-1,023263777	-1,136534312	-1,520542004
A_64_P033569	Tep1	2,24E-09	0,609879611	1,092624085	-1,342464176	1,029966076
A_64_P033845	Lmo1	1,67E-23	0,897760515	-1,001950795	1,108482898	1,378696783
A_64_P034090	Adm2	1,05E-10	0,654216305	1,479690638	1,004407398	1,45895064
A_64_P034155	Ccdc166	2,48E-29	0,94982725	1,298650305	-1,092932445	1,370733911
A_64_P034684	Rpain	1,05E-09	0,621332606	1,15195046	1,103757798	-1,432798554
A_64_P034744	Acrbp	1,18E-13	0,716294252	-1,126371855	-1,001621857	-1,150244015
A_64_P035238		3,90E-29	0,93816837	-1,122782506	-1,269699478	-1,188877607
A_64_P035663		4,35E-27	0,918710049	1,220593354	1,116605711	1,292148606
A_64_P036174	Slc25a47	7,70E-26	0,900854478	-1,104552399	-1,326332563	-1,696932995
A_64_P036514	Chrm2	9,87E-15	0,74168803	-1,308152241	-1,092467272	-1,721139817
A_64_P036676	Klf6	1,27E-10	0,671536587	-1,037263217	-1,197989165	-1,590500218
A_64_P036700	Hist1h1c	9,47E-28	0,907552926	1,033555428	-1,33597912	-1,334410966
A_64_P037245	Shisa2	8,57E-30	0,929349475	-1,094424544	-1,022694311	-1,594458889
A_64_P037259	Ankar	1,85E-16	0,807345913	1,449831702	1,818979331	1,019468359
A_64_P037328		1,39E-11	0,612814071	1,140273524	1,071778707	1,582323441
A_64_P037758	Stk24	3,57E-11	0,647591376	1,158193379	-1,173225387	-1,269217686
A_64_P038352	Appl2	5,42E-15	0,747451112	-1,049361939	-1,307106542	-1,27171471
A_64_P038625		1,89E-14	0,768810676	1,002184139	-1,280571707	1,018471634
A_64_P038767	Aste1	5,98E-14	0,741444707	-1,013482098	1,061354774	1,40008313
A_64_P038839		1,31E-11	0,717790094	1,389203143	-1,312106696	2,125596563
A_64_P038902		2,96E-17	0,820740995	-1,231927834	-1,233898202	1,076522371
A_64_P038926	Prodh1	3,28E-09	0,603956249	1,002532905	1,078112872	1,427592067
A_64_P039737	Scx	6,02E-27	0,917697816	1,063000061	1,369930117	1,133550449
A_64_P039911		7,85E-14	0,700825808	1,147159212	-1,059558556	-1,385760194
A_64_P040095		1,07E-13	0,735517546	-1,338564882	1,125475467	1,669070102
A_64_P040320		2,11E-18	0,825765616	1,233911754	1,945692065	1,018997209
A_64_P040351	LOC102553962	1,27E-13	0,673046515	1,172826657	1,212191457	1,181010507
A_64_P041184	Vipr2	1,08E-14	0,740845663	-1,009326492	1,123355958	-1,234091521
A_64_P041219	Pcsk1	1,17E-20	0,832064465	-1,282341592	-1,048605678	-1,562146979
A_64_P041498	Pla2g4f	9,96E-20	0,818380465	1,221013685	1,182719035	1,171744286
A_64_P041996	Stac	4,59E-20	0,84949569	-1,215806397	1,154364893	-1,098228823
A_64_P042249	Acvr1c	8,24E-19	0,844208698	-1,205225272	1,28305066	-2,022788245
A_64_P042278	Crem	6,48E-21	0,860348992	-1,276980058	1,096261578	-1,123877326
A_64_P042283	Crem	6,31E-27	0,900924146	-1,070512946	1,054440903	-1,200292991
A_64_P042885	Fn3k	1,83E-10	0,62491671	-1,048795712	1,120126903	-1,772291177
A_64_P042930	Hcfc2	1,26E-13	0,695569389	-1,05753115	1,103413009	1,38099949
A_64_P043033	Cbr3	9,66E-11	0,693596233	1,070586726	1,310481857	1,008198302
A_64_P043259	P2rx6	7,80E-16	0,747582023	-1,086290737	1,357054185	1,415421118
A_64_P043361	RGD1307443	7,01E-17	0,800690543	-1,369424425	-1,013367967	-1,886332501
A_64_P043561	LOC494538	1,35E-11	0,660441803	-1,528541667	1,127944772	2,189893834
A_64_P043614	Slc39a5	2,85E-19	0,850548775	1,40738175	-1,004034407	1,862871163
A_64_P043731	Vps37b	2,47E-10	0,620543465	-1,028850769	1,166123395	1,248728917
A_64_P043776	Fat4	1,63E-16	0,821469821	-1,31015953	1,198208556	1,270164453
A_64_P044087	Nap1l5	1,15E-10	0,631453313	-1,130918904	-1,079692686	-1,189646834
A_64_P044101	Mgme1	8,31E-15	0,7433596	1,093425912	-1,966048675	-1,354395702
A_64_P044440	Fam43a	7,27E-22	0,848319439	-1,504691794	1,080272024	1,491603927
A_64_P044470	Mrpl50	3,42E-12	0,677675589	-1,044808566	-1,247634014	-1,470816616
A_64_P045022	Arhgap42	1,91E-10	0,600652771	-1,361554004	1,208173234	-1,462517411
A_64_P045300	Tesk1	1,03E-12	0,647523237	-1,043211948	-1,013889846	1,431542498
A_64_P045353		3,21E-13	0,705343552	-1,360415596	-2,144961689	-2,218212916
A_64_P046358	LOC102546495	5,89E-16	0,730549507	1,722796742	-1,506234432	-1,459645137
A_64_P046552	Sdcbp2	5,78E-25	0,910289906	1,240872392	-2,063902453	-1,375308147
A_64_P046853	Il17ra	1,85E-16	0,777631235	1,136465107	-1,081697323	1,248056148

A_64_P046885	LOC363337	2,06E-22	0,877558882	-1,137067104	-1,322333052	-1,254785706
A_64_P047131		3,33E-10	0,616218506	1,488574131	-1,466836519	1,449137209
A_64_P047386		1,46E-08	0,622422426	1,198528695	-2,026689715	1,107712226
A_64_P047730	Eif1	5,38E-18	0,819392156	-1,036467816	1,05416492	1,240785988
A_64_P047774		1,23E-10	0,607071514	1,339394157	-1,19430754	-1,741751735
A_64_P048004	Bfsp2	2,81E-07	0,631321851	-1,278236111	1,770811024	-1,048828301
A_64_P048055	Alkal2	1,68E-19	0,841842333	-1,100601134	-1,323265672	-2,147153153
A_64_P048136	Brca2	1,46E-14	0,718854165	-1,038498602	1,132186008	-1,424888322
A_64_P048606	Rabgef1	3,40E-13	0,661262217	1,101232362	1,112140291	1,192517995
A_64_P048765	Cbs	3,76E-11	0,738367648	-1,148046892	1,580928335	-1,064840749
A_64_P048852		5,35E-10	0,651864183	1,013907188	-1,063395046	1,345487922
A_64_P048897	Tmem60	3,09E-16	0,773297062	1,079766199	1,19439187	1,040350405
A_64_P049161		2,31E-24	0,877095005	1,289251532	-1,086071203	2,082543456
A_64_P049401	RGD1310507	2,20E-25	0,876273156	1,094357175	-1,447644218	-1,278790029
A_64_P049648	RGD1562963	1,93E-18	0,797522739	1,033072926	-1,677945805	-1,215467485
A_64_P050030	Nptxr	7,52E-10	0,626335299	1,678899106	2,03068768	-1,366047453
A_64_P050052	Pde9a	1,75E-26	0,921675356	-1,10710509	1,055949383	1,347892135
A_64_P050079	Zfp951	3,16E-12	0,657017312	1,041522329	-2,551934376	-1,288216156
A_64_P050099		9,38E-25	0,908589895	1,037448551	1,228680727	-2,165795235
A_64_P050802	Syngr3	3,45E-12	0,655933368	-1,153584611	1,307398736	-1,264277448
A_64_P051098	LOC102554371	6,36E-18	0,788465124	1,641911537	-1,810119621	-2,882012614
A_64_P051104	Lmx1a	2,03E-10	0,623436972	-1,022213506	1,029017497	-1,451527264
A_64_P051139		1,72E-16	0,778251143	1,055347426	-1,257017879	-1,477657758
A_64_P051430	Gpx2	2,46E-14	0,691822236	1,363506568	-1,589235241	-1,526235364
A_64_P051631	Pde4b	8,83E-14	0,737525276	-1,573081607	1,128313793	-1,477446306
A_64_P052076	Sox8	3,75E-14	0,661989356	-1,014932935	1,051785892	1,596660851
A_64_P052422	Otud3	7,18E-14	0,701801938	1,305394808	1,069028711	1,468242497
A_64_P052770	Cdh11	4,09E-15	0,78237378	-1,12975048	-1,481510339	1,539523404
A_64_P052882	Cdpf1	3,89E-13	0,703195852	1,19870914	-1,061818982	-1,277510811
A_64_P053084	Grifin	8,37E-27	0,916660572	-1,023994652	1,297452214	-1,468226453
A_64_P053353	Jun	4,21E-16	0,753233012	-1,169405272	1,03544874	-1,583886394
A_64_P053461	Bag2	3,63E-22	0,884759581	1,099473569	1,232454632	-1,02065161
A_64_P054153	Nudt6	6,76E-25	0,909741906	-1,057224476	-1,573386336	-1,611398067
A_64_P054193	Rsrp1	3,73E-11	0,647014881	-1,027346449	-1,006894145	-1,385662419
A_64_P054337	Efna2	8,41E-16	0,727068831	1,04633324	1,048835837	1,514195305
A_64_P054441	Adra2c	5,46E-10	0,608862172	-1,033873009	1,165820492	1,483515537
A_64_P054461	Bhlha9	3,80E-12	0,654698106	1,43869642	-1,133443396	1,689224433
A_64_P055135		5,12E-18	0,819737331	1,037400202	1,085921702	1,123238686
A_64_P055279	Tmem121	1,24E-10	0,630388127	1,00289158	1,290789037	1,306967868
A_64_P055313	Disp3	3,97E-24	0,903319379	1,021170736	-1,215116024	1,318695527
A_64_P055433	Tmem131	2,04E-10	0,665189688	-1,450188904	-1,370624729	-1,13998752
A_64_P055699	Timm9	2,32E-21	0,876129468	1,067316771	-1,201839805	1,308629139
A_64_P055763	Rhbdl1	6,96E-14	0,721907806	-1,073416381	1,05648447	1,255881854
A_64_P057295		2,70E-19	0,811625804	-1,126495383	-1,263483453	-1,994355282
A_64_P057397		1,10E-12	0,710543664	-1,053103987	-1,219198988	-1,086420351
A_64_P057516	Mrto4	1,21E-12	0,645449748	1,042337388	-1,067636073	1,256237673
A_64_P057975	Hmgn1	3,91E-11	0,623522475	-1,003705183	1,175722776	1,123795696
A_64_P058015	Gimap1	3,22E-16	0,788647615	1,057414504	-1,08721125	-1,68883708
A_64_P058087	Rbp4	1,92E-18	0,861402225	1,211208378	-1,223711092	-1,242395836
A_64_P058336	Ucn	3,41E-22	0,875183724	-1,194654124	1,376534349	1,127887801
A_64_P058539	Zmynd19	1,05E-15	0,724869232	1,178778789	-1,053256978	1,815366664
A_64_P058749		1,13E-16	0,764894502	-1,081183597	1,150706496	1,143093607
A_64_P058878	LOC102554532	1,81E-12	0,664022576	1,364266848	-1,064974407	1,678363188
A_64_P059081		2,03E-15	0,71828913	1,111759818	-1,01865798	1,225319985
A_64_P059120	Aen	8,26E-13	0,694607849	1,143567302	-1,011999165	1,299628836
A_64_P059192	Cdc42ep5	1,54E-09	0,615622588	1,330072033	1,096670097	-1,266878105
A_64_P059495	Ddit3	1,66E-19	0,874595564	1,029464927	1,443275725	-1,103241881
A_64_P060094	Lrrc2	2,57E-14	0,749773262	-1,07126331	1,038662637	-1,855153881
A_64_P060302	LOC365085	1,23E-08	0,644610112	1,223674789	1,116750763	1,216993532
A_64_P060303	Lsr	3,46E-11	0,625214302	1,111464603	1,130659794	1,623768609
A_64_P060319	Gsg1l	3,44E-21	0,852160841	1,436731458	-1,385972498	1,029400437
A_64_P060608		1,90E-24	0,888270089	1,126140685	-1,06688313	-1,793460287
A_64_P061291	Micall1	1,59E-12	0,724349046	-1,015394014	1,334566643	1,367619091
A_64_P061323	Kcna10	3,21E-12	0,698227306	1,587215544	2,207181088	-1,665148103
A_64_P061615	Slc6a7	2,85E-36	0,954788591	1,343594346	-1,211154405	1,274852909
A_64_P061735	Fam78b	4,19E-15	0,731469042	1,098079098	1,049025865	1,643302264
A_64_P061740	Trpv1	3,52E-15	0,797959193	1,142234044	-1,521970445	1,176674347
A_64_P061746	Nlrx1	9,49E-17	0,747649429	1,223481587	-1,010423004	-1,198291944
A_64_P061765	Fam89a	7,92E-10	0,625570122	-1,097019948	1,112715245	1,790699032
A_64_P061770	Psrc1	3,09E-18	0,776836177	-1,305263002	-1,186640884	-1,9672024

A_64_P062048	Znf354b	1,67E-11	0,67818764	-1,043424367	-1,55601401	1,015345339
A_64_P062223	Terb1	8,71E-22	0,880769653	-1,164408156	-1,220138272	1,395678259
A_64_P062285	Vpreb2	4,72E-10	0,633133088	-1,016974778	2,081677386	-1,127899012
A_64_P062388		8,78E-14	0,719459026	1,133017711	1,153173	1,233117563
A_64_P062506		6,86E-28	0,930909909	-1,12611733	-1,322261158	-1,138989778
A_64_P062593	Chst5	1,26E-20	0,867689371	1,092558932	1,454379813	1,039962927
A_64_P062608	Chst7	1,19E-16	0,745616527	-1,015108529	1,068922764	1,326706448
A_64_P062670	Chst8	7,89E-21	0,847508578	1,058649722	-1,688768882	-1,026516915
A_64_P062771	Washc1	9,38E-15	0,723370189	1,029144962	1,19435644	-1,191141734
A_64_P062890	Oaz3	7,54E-18	0,817038035	1,499153908	-2,076657796	-1,350986539
A_64_P062899	Bcar1	5,90E-11	0,61770426	-1,15713885	1,473258408	1,531682028
A_64_P063019	Aox4	1,37E-17	0,798331621	-1,290434424	1,409692414	-1,615371286
A_64_P063035		5,10E-13	0,719046783	1,297346292	-1,203150228	-1,610346238
A_64_P063047	Rpp25	5,39E-10	0,722614254	1,200095146	-1,315993953	1,16276541
A_64_P063644		1,09E-12	0,710649678	1,104692113	-1,10154042	1,457613979
A_64_P063993	Ttc4	5,39E-19	0,85799654	1,012740148	-1,165086775	1,142081439
A_64_P064321	Scrn3	2,39E-17	0,794088871	-1,045727277	-1,275608876	-1,397975377
A_64_P064371	Spata2L	9,54E-17	0,783086503	-1,321839167	1,427229608	-1,306356213
A_64_P064498		1,90E-17	0,779807517	-1,25348015	1,291495628	-1,244309522
A_64_P064531	Npas4	6,04E-37	0,965604441	-1,226049732	1,450937766	-1,726204395
A_64_P064866	LOC100909776	1,58E-20	0,855506879	1,29475421	1,198796411	-1,36911757
A_64_P065032	P3h4	6,25E-10	0,669002395	1,255035997	-1,085173171	-1,161724776
A_64_P065063	Fosl2	3,23E-12	0,678385535	-1,116198165	1,300392175	-1,227248748
A_64_P065102	Evpl	1,83E-19	0,841316455	-1,198116677	1,47593146	1,336243472
A_64_P065301	LOC102554444	3,24E-20	0,862710149	-1,169048894	-1,576504294	1,124398317
A_64_P065492	RGD1565222	6,39E-17	0,75121023	1,213832623	-1,431890519	-1,822687206
A_64_P065691	Adat3	4,29E-12	0,653141304	1,016293912	1,474127366	-1,138861494
A_64_P065868	Acvr1c	5,04E-29	0,931364524	-1,210656729	1,453010099	-1,640679225
A_64_P065939	B3galt2	4,03E-13	0,682192739	1,032558671	-1,169833895	-1,641834718
A_64_P066544	Smim3	1,24E-10	0,607029104	1,161419147	-1,109636477	-1,340058776
A_64_P066586		3,37E-16	0,755264585	1,080799333	-1,158386613	-1,170966855
A_64_P066621	Msc	4,25E-20	0,861253575	1,127190721	-1,241874027	1,063939784
A_64_P066782	LOC108348347	1,80E-25	0,928167651	1,129858966	-1,593684474	1,060892518
A_64_P067000	Prima1	9,86E-27	0,923393256	-1,062083369	-1,19497341	-1,522766989
A_64_P067366	Cacna1h	2,24E-14	0,733560864	1,538279202	-1,838682625	1,761871614
A_64_P067659	Aldh1a1	4,39E-11	0,644807753	-1,090084664	1,416849597	-1,108086004
A_64_P067868	B3gnt9	1,43E-10	0,604966247	1,146547512	-1,160392379	1,608828261
A_64_P067883	Trim47	4,45E-23	0,874322221	1,144670944	1,036412492	1,2211449
A_64_P067884	Trim47	8,68E-27	0,9085981	1,197839116	-1,030286375	1,415896882
A_64_P067908	Trim17	2,16E-34	0,957036291	1,27510681	-1,865553016	-2,030357112
A_64_P067915	Nudt8	5,63E-10	0,670435834	1,250882322	1,305746272	-1,039011789
A_64_P068027	LOC363337	1,18E-14	0,757193507	-1,12443975	2,731293989	2,402422514
A_64_P068038	Hexb	3,69E-14	0,687298279	-1,071317577	-1,23327561	-1,251965789
A_64_P068152	Smad6	2,75E-22	0,886008758	1,068854784	1,345669599	1,109231353
A_64_P068237	Fam149a	3,87E-11	0,64651287	-1,114521095	1,116248348	-1,067367394
A_64_P068252		4,63E-13	0,630837142	-1,117250089	1,006768767	-1,219341646
A_64_P068594	Car6	4,03E-14	0,708092803	1,20004893	-1,141634496	-1,173922315
A_64_P068786	Zfp629	1,48E-13	0,693703745	1,045675521	1,124578398	1,332323773
A_64_P068965	Sowaha	7,49E-13	0,674821415	1,019586559	-1,036574062	-1,378655691
A_64_P069071	Lpar6	1,52E-08	0,601093345	1,252728037	1,099624117	-1,177162142
A_64_P069081	RGD1310495	2,54E-10	0,681111588	1,023395856	-1,826511466	1,158782579
A_64_P069136		4,81E-16	0,73249857	-1,122932256	-1,029112818	-1,426348541
A_64_P069211	Cdc42bpg	1,36E-09	0,617470588	1,1789701	-1,334998323	1,155024105
A_64_P069261	Arl4d	5,48E-11	0,641784946	-1,219904237	2,079159202	-1,551798568
A_64_P069374	Scn10a	3,24E-28	0,938869208	1,218413057	-1,861672319	1,054511918
A_64_P069394	Styxl1	2,22E-19	0,81294887	1,018944463	1,291058587	-1,271992258
A_64_P069654	Smtn	2,08E-17	0,77908298	-1,02918068	1,199112826	-1,174673243
A_64_P069739	Ankrd23	3,02E-19	0,810836666	1,06099773	-1,308130943	-1,337215534
A_64_P069819	Rrp8	2,14E-11	0,654370151	-1,005197227	-1,049921009	1,207697481
A_64_P069942	Prss56	2,67E-20	0,852578277	1,480111304	1,515931653	1,493269126
A_64_P070184	Exosc9	4,94E-13	0,700493654	1,044638499	-1,406839152	1,180217913
A_64_P070891	Cacng6	1,40E-12	0,688399269	1,072616994	1,012123767	-1,428575599
A_64_P070937	LOC501346	3,43E-15	0,798174992	1,24261944	2,766808857	-1,021932211
A_64_P071178	Mgat5b	2,27E-16	0,758798904	-1,005139004	1,013609482	1,221372426
A_64_P071810	Gpr143	1,31E-08	0,603506634	-1,259564989	1,000688542	1,000242215
A_64_P071983	Ppp1r3c	5,27E-10	0,65207486	-1,182819309	1,210349752	-1,398613514
A_64_P072088	Lrrtm2	1,26E-11	0,639021619	-1,054091584	-1,101373407	-1,951553136
A_64_P072227	Arrdc2	3,16E-12	0,749714612	1,654617505	-1,134533789	-2,586977022
A_64_P072748	Cage1	7,28E-10	0,604523064	1,136974565	1,278314793	-2,402086843
A_64_P073129	Map3k12	1,58E-12	0,641987674	-1,055091955	1,140597681	1,176744451

A_64_P073550	March11	6,27E-13	0,653721804	-1,182537665	1,298133385	1,529474347
A_64_P073845	Cysrt1	1,36E-21	0,857223117	1,258132223	1,109613196	1,048043501
A_64_P074267	Pnrc1	5,69E-12	0,671395352	-1,165749976	-1,298897421	-3,418021531
A_64_P074490	Wipi1	5,35E-18	0,819422822	-1,00394913	1,113232642	-1,678707412
A_64_P074544	Ankrd61	1,37E-14	0,698264863	-1,084703597	1,271348655	1,327390818
A_64_P075008	Birc7	1,25E-23	0,880148773	1,020500465	1,336771972	-1,223486905
A_64_P075087	Camk1g	5,37E-15	0,807989105	-1,13152489	1,387353975	-1,252824299
A_64_P075124		4,48E-11	0,621594328	1,003277657	-1,06616349	1,505895075
A_64_P075910	Ajap1	1,83E-14	0,735614874	1,064788666	-1,218770107	1,415813797
A_64_P076555	Jph1	5,58E-15	0,779678788	-1,395307886	-1,190242668	-1,225033159
A_64_P076832	RGD1311447	9,47E-24	0,929122166	-1,039429133	-1,268049725	1,495162199
A_64_P076900	Nedd1	1,26E-11	0,700603248	1,16116487	-1,402243083	1,03164854
A_64_P076997	RGD1561442	5,95E-11	0,617590205	-1,092843852	1,22820456	1,905286773
A_64_P077240	RGD1562660	2,28E-27	0,927624521	-1,127637315	-1,293582743	1,064780754
A_64_P077242		1,93E-13	0,729443644	-1,205829224	-1,541844754	1,168494465
A_64_P077392		4,74E-14	0,789882183	-1,245974722	-1,730488987	-2,724657785
A_64_P078140	Amigo2	2,56E-26	0,895726882	-1,061030516	-1,161496799	-1,527927617
A_64_P078230	RGD1312005	2,04E-13	0,690099615	-1,084528924	-1,139756278	-1,620610216
A_64_P078303	Angpt4	1,94E-17	0,810290381	-1,175011275	1,928749523	-1,207325512
A_64_P078875		3,72E-13	0,68314265	-1,007751789	-1,71118099	-1,023686029
A_64_P078894	Il23a	7,20E-28	0,908474381	1,179150331	-1,590266814	-1,074654716
A_64_P079260	Ttc36	2,89E-13	0,686087039	1,381575869	-1,119941904	1,805636321
A_64_P079523		2,81E-21	0,853272474	1,042796286	-1,504084836	1,082232664
A_64_P079553	Myh8	8,46E-11	0,695269386	-1,001855333	1,091282039	1,377406796
A_64_P079666	Cyp4f5	9,76E-22	0,870078772	-1,125017451	1,137458311	-1,13076379
A_64_P079731	Zfyve21	4,41E-16	0,770228091	1,09873104	1,083195391	-1,254856158
A_64_P079822		7,41E-16	0,765706944	-1,055916315	1,007893442	1,413901855
A_64_P080154	Klf11	9,18E-15	0,742391432	-1,208668129	1,22216494	-1,338482281
A_64_P080329	Paxx	3,07E-11	0,649604763	1,080307731	1,181102362	1,054687853
A_64_P080667		1,95E-14	0,734965778	1,126632676	-1,518802206	-1,564252837
A_64_P080941	LOC102556967	1,70E-14	0,695928876	-1,174551739	1,121912619	1,112064217
A_64_P081063		1,14E-24	0,88021415	1,212982772	-1,787710256	-1,712882667
A_64_P081328	Hmgn2	1,38E-19	0,800411862	1,06049234	1,394881756	1,133962829
A_64_P081546	Plag1	5,59E-13	0,678323807	1,092322406	-1,137674116	-1,720642812
A_64_P081822	Ticrr	4,62E-10	0,65392158	1,008096588	1,591217679	-1,098751473
A_64_P082356		2,66E-11	0,628830171	1,08912185	-1,188058964	1,692584793
A_64_P082386	LOC102553270	1,70E-09	0,654965418	-1,294510009	1,101581972	1,493448025
A_64_P082495	RGD1564937	2,76E-14	0,690556298	-1,235668419	-1,004406402	-1,955878022
A_64_P082693	Egr1	1,15E-25	0,935547258	1,215104338	1,549733351	-5,081059805
A_64_P082949	Ube2j2	7,18E-12	0,621897387	-1,041403656	1,133515795	1,379546472
A_64_P083029	Arhgef33	1,35E-25	0,878439536	1,003256936	-1,379699891	-1,612195779
A_64_P083034	Asmt	1,80E-31	0,938799441	1,130287842	-1,528869619	-1,056741084
A_64_P083625	Klhl3	1,60E-11	0,635781415	1,070867809	-1,294097406	-1,094824709
A_64_P083944	Samd11	4,07E-13	0,702696285	1,06661596	-1,29131102	-1,286741899
A_64_P083978	Zfp68	4,01E-20	0,871983576	-1,321972641	1,026955529	-1,103356035
A_64_P084333	Lrfn4	8,23E-11	0,612946437	1,059843741	1,131044883	1,490703213
A_64_P084473	Pear1	1,87E-16	0,760518042	1,125357739	-1,134506046	-1,654255929
A_64_P084483	Immp1l	1,59E-13	0,692941888	1,162614764	1,350632898	1,239465298
A_64_P084851	Rcan2	8,73E-13	0,693956726	-1,160426325	1,440141121	-1,511300974
A_64_P085004	Kiss1r	4,66E-25	0,902905464	1,122812772	-1,130721552	-1,349248609
A_64_P085058		8,57E-12	0,686455169	1,13187303	1,55676828	-1,453861833
A_64_P085530	Scd	8,23E-11	0,657453423	-1,117325063	1,213206027	1,823570821
A_64_P085671		7,44E-20	0,868791872	-1,175231105	-1,505463114	-1,432066512
A_64_P086383	Mboat4	1,36E-16	0,744371883	1,225524508	-1,394776421	-1,865346532
A_64_P086398		9,62E-12	0,664759813	-1,039482025	-1,24623544	-1,217435973
A_64_P086445	Nr2c2ap	3,70E-13	0,683197713	1,173232755	1,194244531	1,158388624
A_64_P086472	Fbxl19	2,45E-15	0,716384809	1,033203804	-1,12594018	1,253432721
A_64_P086565	Rras2	2,11E-18	0,779863239	1,115532828	1,139536484	1,09847594
A_64_P086784	Runx1t1	9,06E-15	0,759637827	-1,041847941	-1,110452821	-1,517586237
A_64_P086989	Slc13a1	6,57E-13	0,653143464	-1,001342799	-1,187132226	-1,346431253
A_64_P087014	Slc18a1	9,44E-20	0,818736979	-1,012954484	-1,678835599	-1,426703853
A_64_P087233	Pias4	6,60E-11	0,660403043	-1,029241233	1,272662581	-1,234280615
A_64_P087238	Harbi1	4,01E-13	0,682246231	1,039902465	-1,236532606	-1,510824119
A_64_P087380	Efna3	6,70E-25	0,882523927	1,181151538	-1,112578537	1,695105892
A_64_P087801	St14	1,09E-14	0,740759993	1,125674502	-1,021121961	-2,040859011
A_64_P087867	Sys1	2,80E-10	0,61873004	1,075669308	1,243106841	-1,241023616
A_64_P088126	Mrps27	2,56E-12	0,659701206	-1,054448402	1,236956598	1,021799617
A_64_P088382	Angptl4	6,78E-16	0,823697943	1,025193374	-1,129827875	-2,440595787
A_64_P088392	Hbb-b1	7,98E-09	0,734603203	-1,480053206	2,368289308	1,327497643
A_64_P088393	Hbb-b1	5,21E-09	0,638425709	-1,380939701	3,267698235	1,216327558

A_64_P088397	Sytl1	1,77E-22	0,912687538	1,228739514	1,077759538	1,262250344
A_64_P088695	Tmtc4	2,48E-11	0,692356573	-1,234456021	-1,036031817	-1,581341544
A_64_P088995	LOC685668	2,13E-19	0,840413303	1,043995458	-1,168071227	1,258921538
A_64_P089020	Ier5	3,11E-17	0,806814052	1,152204446	1,27621005	1,105190066
A_64_P089201	Tcp11	1,70E-13	0,712326301	1,49094031	1,263432194	1,666229124
A_64_P089400	Hpse	6,60E-13	0,697187499	1,046538532	-1,115805417	-1,30383257
A_64_P089475		2,26E-15	0,717193487	1,10508012	1,363646731	1,810913153
A_64_P089662	Atp8b2	1,13E-11	0,615611722	-1,075256439	1,151526103	1,217464538
A_64_P090304	Ccdc88b	5,14E-26	0,931722153	1,14554391	-1,032332599	1,153685666
A_64_P091208		1,79E-17	0,796306017	1,199661975	-1,340233187	-1,522478832
A_64_P091953	Spink4	8,86E-15	0,759845218	-1,135761214	1,781537943	-1,48110426
A_64_P092122	Zmynd8	9,55E-09	0,6086459	-1,117819471	1,092886205	-1,470779548
A_64_P092534	Sspo	1,11E-24	0,915643325	1,522351041	-1,192002262	1,189758532
A_64_P093026	Anks6	4,86E-12	0,693315995	-1,06134846	-1,119449046	1,388499785
A_64_P093044		5,29E-18	0,844407699	1,044759328	1,042365028	1,598857209
A_64_P093389		6,39E-15	0,762856755	-1,48387845	-1,350694142	1,333048712
A_64_P093452	RGD1305464	9,42E-22	0,846877533	1,059142679	1,206796996	1,23769439
A_64_P093522	Dtna	2,59E-12	0,700745232	-1,040276832	1,022567477	-1,23450785
A_64_P093599	Abcc4	2,16E-11	0,60659399	1,047248992	-1,261197653	1,14269444
A_64_P094515	LOC100360619	1,87E-17	0,823946711	1,027890568	-1,130505942	-1,02251374
A_64_P094722		2,72E-18	0,810199864	1,153183089	-1,376039887	1,093128801
A_64_P095005	Mmd2	2,72E-13	0,774060842	-1,204038989	1,259231358	1,010494781
A_64_P095586	LOC498316	1,23E-17	0,813574721	-1,052951277	1,463281251	-1,604350133
A_64_P095830	Adgrg1	4,84E-14	0,706114556	-1,057817807	1,023137126	1,138534596
A_64_P095900	Rgs12	4,03E-12	0,653928835	1,005051633	1,256769038	1,192760734
A_64_P096006	Xrra1	1,30E-09	0,618170926	-1,484951529	-1,045427485	-2,108743118
A_64_P096083		1,24E-12	0,689852935	1,119159919	1,297533161	1,373007523
A_64_P096782	Ier5l	6,49E-16	0,782876826	1,360993513	1,278935281	1,620174662
A_64_P096787	Plpp4	4,49E-15	0,781557368	1,014533038	1,221987388	-1,074132844
A_64_P097024		1,39E-15	0,742119895	1,036816096	-1,413418336	-1,471250902
A_64_P097218		4,26E-14	0,72700958	-1,05924674	1,076172058	-1,852028873
A_64_P097340	Zfp867	1,98E-15	0,71851833	-1,105275917	-1,060944163	-1,30568035
A_64_P097598	Mtus1	3,60E-12	0,696882745	-1,007169735	1,1551473	-2,733758062
A_64_P097947	Pus7l	2,01E-13	0,66760148	-1,07915332	-1,317108358	-1,150028568
A_64_P097974	St3gal5	8,92E-12	0,665714717	1,108523383	-1,059017713	-1,003462873
A_64_P098006	Fitm1	1,95E-20	0,854334418	-1,015607729	1,112486516	-1,068424327
A_64_P098334	Ttc22	1,82E-31	0,932339272	1,49779824	-1,276941866	1,376593116
A_64_P098622	Rpusd2	7,27E-18	0,787427263	-1,00031181	1,100692259	1,314294023
A_64_P098698	Nkpd1	9,21E-20	0,857001306	1,173069101	-1,266191688	1,24558338
A_64_P099055	Lsmem2	1,77E-11	0,65691802	-1,547503991	1,312889054	-2,599270177
A_64_P099151		1,43E-10	0,628400067	1,122832237	-1,303871577	-1,316305937
A_64_P099307	Tcf7l2	2,70E-14	0,731699287	1,151098388	1,370234391	-1,006622214
A_64_P099624	Lcn12	6,87E-10	0,605412841	1,098077378	1,21967992	-1,137109667
A_64_P099923	Dap	7,04E-12	0,724857117	1,118303012	1,041647989	-1,309262501
A_64_P099983		2,17E-13	0,709674463	-1,129657504	-1,19250607	1,636959502
A_64_P100279	Zdhhc8	4,12E-11	0,645674365	1,002346459	1,264859982	1,056829183
A_64_P100346		2,15E-32	0,953736642	1,203372706	1,132971468	1,088005236
A_64_P100447	Hoxd9	1,30E-11	0,700203942	1,074901193	2,618817746	-1,43788321
A_64_P100516	Aoc3	1,89E-15	0,828412426	-1,158915188	-1,778227526	1,875500893
A_64_P100544	Itsn2	6,54E-11	0,660523398	-1,223248573	-1,087785633	1,463545725
A_64_P101204	Slc40a1	1,19E-14	0,720914696	-1,156934541	-1,08937323	-1,577150592
A_64_P101336	Rfx5	1,31E-10	0,629623715	-1,079936272	1,1403752	2,863171344
A_64_P101553	Sec63	1,15E-12	0,710069849	1,026720669	1,052719875	1,272312753
A_64_P101618	Slc25a28	1,61E-14	0,77025553	-1,211574507	1,30676	-1,187897388
A_64_P101804	Efnb1	1,92E-17	0,810346874	-1,052706389	1,396229075	1,221496153
A_64_P101908	Hmcn2	1,83E-15	0,789154827	1,015895277	-1,068439868	1,762089801
A_64_P102141		3,68E-13	0,72260109	-1,101551523	-1,24237581	-1,362952799
A_64_P102545	Tmod4	3,08E-11	0,670376935	1,04149367	1,107907112	-1,441795786
A_64_P102801	LOC108348140	1,90E-10	0,600690781	-1,089346409	2,01106346	2,439100663
A_64_P103055	Pnpla3	1,32E-19	0,816517328	-1,080483245	-1,2461788	-1,036124668
A_64_P103238	Zfp52	1,27E-12	0,668395485	-1,020230842	1,00718205	-1,260875387
A_64_P103254	Klhl36	1,13E-16	0,764924524	-1,160844452	1,48538759	-1,035410662
A_64_P103716	Sco1	2,24E-12	0,682815342	1,086718072	-1,072079591	1,175028753
A_64_P104590	Cfap157	4,69E-14	0,706453589	-1,009823426	-1,577923267	-1,271730656
A_64_P104810	Podnl1	1,47E-08	0,601738529	1,025964868	1,643156347	-1,241461654
A_64_P104865	Lyrm1	6,66E-16	0,749048067	-1,112038664	1,594094443	-1,21363295
A_64_P105122	Smim5	1,25E-26	0,898426687	2,692774463	-1,224074359	1,422020438
A_64_P105152	Sall2	1,79E-14	0,695333505	1,003806036	1,084199367	1,169287404
A_64_P105591	Tnfaip8l1	1,31E-17	0,764941497	1,121308077	1,416654205	1,001172036
A_64_P106643		6,47E-24	0,860357227	1,702440409	-1,85033932	-4,396482938

A_64_P107185		3,91E-12	0,654304638	1,186602445	1,853691122	-4,047083457
A_64_P107851	Smarcd3	8,31E-14	0,700189869	1,166446816	1,163832078	1,219726891
A_64_P108245		8,94E-12	0,618891997	1,08980375	-1,335680101	-1,675077714
A_64_P108379	Cops7b	3,08E-11	0,649554405	-1,050758692	-1,226359408	1,372168918
A_64_P108454	Spata5l1	1,84E-13	0,691291115	-1,015411708	1,148666602	1,78901688
A_64_P108459		2,00E-22	0,904583474	1,316105736	-1,424692453	1,098585534
A_64_P108876	Dusp13	8,64E-15	0,703228409	1,102375477	-1,120788508	-3,035893307
A_64_P108948	Atxn2	3,43E-12	0,63182576	-1,162667022	1,138872195	1,319651275
A_64_P109531	Tgfb2	5,21E-10	0,652234652	-1,280912369	-1,153698341	-1,283048326
A_64_P109899	Lgals4	3,02E-15	0,714232196	1,487475195	1,105170609	-1,722045975
A_64_P109900	Lgals4	3,67E-23	0,875231444	1,523314196	-1,356165708	-2,02580976
A_64_P109938	Gnb1l	7,75E-13	0,69533923	1,207721928	1,207890596	1,322854446
A_64_P110469	Fdxr	1,93E-12	0,704143658	1,010529501	1,003622747	1,300928894
A_64_P110544	Spdef	6,20E-14	0,801090847	1,30980536	-1,198551367	1,000498386
A_64_P110574		6,11E-12	0,624084617	1,008363347	-1,343325039	-1,118288587
A_64_P110599	Plekhf1	1,52E-23	0,906428279	-1,064674805	1,244733105	-1,619352898
A_64_P110798	Serinc2	7,57E-10	0,603937607	-1,157167532	1,363251526	1,07751021
A_64_P111500	Slc1a5	1,39E-11	0,63769923	1,251590124	-1,548392431	-1,244026608
A_64_P111599	Bcor	4,44E-15	0,766167124	-1,132853045	-1,095078474	1,375559905
A_64_P111674	Olr206	7,09E-17	0,785491154	-1,362628257	-1,552943121	-1,39406816
A_64_P111776	Rab4a	8,89E-16	0,764092801	-1,01656007	1,207418193	-1,036819696
A_64_P111791	Abhd18	3,72E-12	0,696497401	-1,091159596	1,205481349	-1,486630302
A_64_P111903	Mmp17	2,83E-12	0,679987176	1,065507634	-1,007237646	1,271523789
A_64_P112185		4,59E-18	0,833368359	-1,02475407	1,014089841	-2,100561608
A_64_P112295	Slc1a7	5,23E-10	0,609512993	2,163564109	1,057342943	-1,365568325
A_64_P112327	Ftsj1	3,48E-12	0,655825109	-1,001172209	-1,055777512	-1,251756633
A_64_P112406	Chac1	9,83E-15	0,758882557	-1,042388014	1,284874935	-1,237885417
A_64_P112767	Ralbp1	4,19E-13	0,702346666	-1,069880588	1,021178069	-1,646525905
A_64_P112816	gen-01	2,01E-21	0,842567171	-1,33007832	-1,177389196	-1,204412929
A_64_P114263	RGD1564786	3,25E-16	0,736247758	1,06454946	-1,215822178	-1,184999245
A_64_P114396		7,09E-24	0,909206562	1,905927974	1,267416805	-1,224707391
A_64_P114960	Kcnk4	3,48E-12	0,655810165	1,453388947	1,227496805	1,872883783
A_64_P115222	Pitpnm2	6,83E-17	0,827608605	-1,187389648	1,040605581	-1,090330228
A_64_P115417	Ntrk1	6,73E-14	0,702522566	2,185187128	-1,443955298	1,299354868
A_64_P115786	LOC299312	5,26E-11	0,619328791	1,019828209	-1,049418561	1,366347161
A_64_P116171	Ak8	7,14E-18	0,842528857	1,092752471	1,46044153	-1,497479237
A_64_P116299	LOC102557206	5,14E-11	0,663710321	-1,262579026	-1,771914535	1,219515462
A_64_P116301	Adam33	1,18E-09	0,640548093	1,170364877	-1,277843065	1,7711376
A_64_P116606	Rcan1	1,44E-10	0,628291338	1,169583181	-1,078730313	-1,526492541
A_64_P117126	Slc25a19	1,12E-17	0,814220259	-1,044830749	1,167219961	-1,17330082
A_64_P117173	Wfdc10	4,62E-13	0,701258718	-1,025789849	1,494819397	-1,407800126
A_64_P117726	Slu7	2,40E-08	0,614446395	1,037962848	1,102804045	-1,261585292
A_64_P117901	Trib3	3,59E-20	0,838620102	1,139475377	-1,067850508	1,264209849
A_64_P118308		8,53E-20	0,819406287	1,040788495	-1,749781403	-1,740518803
A_64_P118457	Cited4	3,15E-28	0,926420908	1,00608012	1,418377982	-1,152453148
A_64_P118526		8,20E-11	0,636228764	-1,098821009	-1,300258438	-2,524190528
A_64_P119314	LOC689065	2,47E-15	0,736636994	1,236560293	-1,391318948	-1,082753496
A_64_P119369	Baat	1,57E-21	0,856430231	-1,022845497	-1,16851963	-1,393871388
A_64_P119680	Alkal2	1,37E-12	0,688718302	-1,307935312	-1,15144057	-2,351933832
A_64_P119821	RGD1311084	1,97E-24	0,927518576	1,278723011	-1,018860937	1,149623063
A_64_P120070	Insl6	1,41E-21	0,857021007	1,100137584	-1,030475297	-1,512107701
A_64_P120445	Cacng6	8,37E-21	0,869776082	1,015285871	1,263091891	-1,348361089
A_64_P120861	Efna1	5,07E-13	0,679478673	1,063381948	-1,214527398	-4,312106171
A_64_P120881	Hils1	1,22E-15	0,743352137	1,09777519	1,099790866	1,704353977
A_64_P121136		3,02E-23	0,876128317	-1,365948541	-1,801472839	-3,332095893
A_64_P122238	Tuba4a	2,92E-13	0,7064118	-1,134835506	1,236153086	1,522438848
A_64_P122624	Sphkap	9,93E-29	0,935890037	-1,232130729	1,054842643	-2,096824041
A_64_P122790	Acss1	5,72E-15	0,746949636	1,114420074	-1,072657852	-1,407973226
A_64_P122944	Phf20l1	2,29E-19	0,827009308	-1,198893984	-1,494445318	1,347127236
A_64_P122975		1,14E-13	0,716682191	1,099912828	-1,302751901	1,440984912
A_64_P123949	Gk	2,03E-25	0,905927222	-1,001489845	1,133353306	1,126511101
A_64_P124090	Ntsr1	2,03E-29	0,954842786	1,290512609	-1,170437807	1,334191356
A_64_P124210	Greb1l	1,47E-15	0,74161451	-1,08655852	-1,489592093	-2,087134176
A_64_P124898	LOC363337	3,79E-18	0,821807074	-1,323887746	-1,015265097	2,007724199
A_64_P125305	B9d2	1,89E-11	0,633518103	1,208272818	1,054476212	-3,256174598
A_64_P125350	Kcnh6	9,43E-27	0,916284175	1,091051756	-1,202928605	-1,162384443
A_64_P125611	Tbx6	4,75E-20	0,849302168	-1,134496293	-1,363699195	-2,403791625
A_64_P125858	Socs5	1,39E-11	0,680494975	-1,002635385	1,074872866	1,766343267
A_64_P126030	Slc22a7	8,44E-14	0,784795012	-1,206837482	-1,178343956	1,86498247
A_64_P126265	Rbm20	3,30E-12	0,632360714	-1,134592854	-1,160084239	-1,521146527

A_64_P126955	Rybp	1,11E-12	0,710398095	-1,063252176	1,576131787	1,821947717
A_64_P127223	Mocs3	1,48E-20	0,815762267	1,078319295	1,10729154	1,069653663
A_64_P127327	Sys1	2,71E-14	0,749232571	-1,095093216	1,201674656	-1,038329819
A_64_P127833	Ppfibp1	4,23E-11	0,666281799	-1,053987071	-1,035716143	1,164926296
A_64_P128454	Nrf1	1,03E-11	0,641629245	-1,07838845	1,125610694	1,209624093
A_64_P128725	Mthfd2	3,11E-13	0,685214172	-1,075269236	1,253357862	3,401289156
A_64_P128874	MGC116197	4,47E-12	0,674387588	1,042548933	-1,180642191	1,339902226
A_64_P129004	Kcnj12	1,99E-19	0,863527901	1,109291599	-1,140467069	1,004624358
A_64_P129730	Tmem79	1,51E-18	0,84048715	1,177669321	-2,017786804	-1,013453433
A_64_P129951	Nup205	3,78E-20	0,824712792	-1,110387665	-1,08231306	1,382429821
A_64_P130025	Dll1	7,51E-14	0,721109771	1,312097156	1,181032962	1,58376425
A_64_P130074	Pot1b	1,33E-10	0,605983842	-1,226009355	1,191661521	-3,661671174
A_64_P130239	Slitrk3	1,40E-09	0,617053559	-1,050495389	-1,078438255	-1,606708557
A_64_P130324	Lcn5	1,56E-20	0,830318702	1,05192156	1,294354974	-1,271531974
A_64_P130380		1,67E-18	0,827311127	-1,045731253	1,308792728	-1,020139041
A_64_P130390	LOC681364	2,99E-19	0,83831524	1,069665266	2,461341194	-1,125317106
A_64_P130873	Csrnp3	1,75E-19	0,814576283	-1,026916159	-1,28582044	-1,86488212
A_64_P131051	Fos	7,66E-33	0,945574198	-1,079664296	-1,094223261	-1,613589946
A_64_P131086	Rab30	1,15E-15	0,743978339	-1,285219284	1,395799751	-1,160551569
A_64_P131122	Iqank1	8,39E-16	0,780716544	1,240285843	2,180825339	-1,148446141
A_64_P131141		9,80E-16	0,763220363	1,135330067	-1,314915587	-1,067509834
A_64_P131151	Opn4	3,95E-15	0,767227623	1,114571655	-1,660075642	-1,232283242
A_64_P131409	Pdgfa	2,56E-11	0,629386286	1,120982076	1,163721874	1,272841677
A_64_P131477	Twist2	2,80E-11	0,650862426	1,027355372	1,128455424	2,033964132
A_64_P131791	Shroom3	5,20E-20	0,848778653	-1,116149099	1,178138642	-1,4392117
A_64_P131869	Cntrob	6,63E-16	0,729391945	1,118861918	-1,307750635	-1,110492462
A_64_P132199	B3galt5	2,48E-24	0,887166797	1,172550477	1,199879676	1,316294586
A_64_P132327	Dnase1	1,41E-12	0,707750799	1,06825466	1,173144344	-2,881353682
A_64_P133096	Neurl2	1,72E-12	0,740274718	1,323475254	1,044514898	-1,474715817
A_64_P133152	Neurod6	2,01E-10	0,684186644	-1,100109698	1,287763948	-1,099504463
A_64_P133167	Duoxa2	1,04E-15	0,793788071	1,052403098	1,211720374	2,162038929
A_64_P133276	Dact2	4,41E-22	0,837557844	1,119576989	1,117659042	-2,001287783
A_64_P133430	Mat2b	1,88E-10	0,6008753	-1,032858786	-1,165066074	-1,819334363
A_64_P133632	Card19	1,94E-19	0,82807898	1,104097861	1,330292855	-1,172818089
A_64_P134699	Rft1	2,09E-15	0,717989046	1,046373812	-1,310775125	-1,337108678
A_64_P134744	Hpn	2,13E-18	0,825690953	-1,034198437	1,564225687	1,616764669
A_64_P135152	Msantd1	1,40E-19	0,830185087	-1,094310237	-1,057497593	1,43636863
A_64_P135502		1,15E-14	0,740198696	-1,034232811	-1,048128587	-2,783973734
A_64_P135843	Grb7	3,28E-30	0,931828003	-1,006702722	-1,293683334	-2,703818293
A_64_P136367	Cbx8	1,49E-09	0,637114942	1,035324084	1,312527854	-1,05100687
A_64_P136394	Col20a1	1,76E-11	0,656931213	1,330536899	-1,315841163	1,375942136
A_64_P136432	Armcx6	1,42E-17	0,782164468	-1,00868835	1,187629064	-1,298074063
A_64_P136512	Ecel1	5,39E-15	0,764419578	1,337419623	1,213129841	1,352520396
A_64_P136751	Rgs6	6,42E-10	0,628660836	-1,270716934	1,161535864	-1,274095863
A_64_P137070	Zfp318	1,81E-12	0,664069349	-1,096569153	-1,012816641	1,328069705
A_64_P137075	Col7a1	6,20E-18	0,788666211	1,094004748	-1,283729244	-1,079377291
A_64_P137320	Ccdc113	1,75E-25	0,877296126	-1,094343612	-1,089230118	1,497427547
A_64_P137436	LOC688286	3,24E-26	0,904007594	1,52302203	-1,39962544	-1,34666126
A_64_P137461	Bspry	1,12E-12	0,744739134	1,51109506	-1,039102023	1,58118882
A_64_P137784	Plvap	3,84E-27	0,911334354	1,232213981	1,045380237	1,590510945
A_64_P138086	Pcdh20	2,28E-11	0,605775487	1,105463147	-1,144753432	-1,30184335
A_64_P138216	Gch1	4,02E-14	0,761802708	-1,191388402	1,028186354	-1,188446426
A_64_P138291	Cenpk	2,86E-22	0,876027387	-1,045199283	-2,195034705	-1,105237463
A_64_P138415	Cldn19	6,33E-17	0,786406354	1,007982891	1,223618366	1,237404358
A_64_P138430	Odf3b	1,14E-19	0,855787011	-1,007823738	1,122986823	-1,367827386
A_64_P138560	Narf	3,87E-19	0,774089751	1,066491428	-1,287670681	-1,463826167
A_64_P138575		1,95E-13	0,746419185	-1,250467013	-1,467497169	-1,036862443
A_64_P138636	Nr2f1	2,25E-18	0,779356671	-1,166656379	-1,120611353	-1,35270186
A_64_P138800	Myadml2	9,87E-28	0,915705662	-1,027050229	-1,07060769	-1,703202214
A_64_P139485	Plcd4	1,43E-08	0,60215902	-1,135099125	1,319051022	-1,209071473
A_64_P139559		4,98E-14	0,705805099	1,242648837	1,76921693	2,336108482
A_64_P139802	Prph	2,06E-11	0,745081454	1,455981183	-1,088898957	1,382038984
A_64_P140000	Myo15b	2,11E-25	0,921015326	1,250175868	1,011442485	1,420391862
A_64_P140025	Tyrp1	6,08E-12	0,690618941	1,177134514	-1,289963666	1,171547046
A_64_P140652	Ccdc84	1,18E-08	0,605298499	-1,078160355	1,119631475	1,396849926
A_64_P140658	Anks1a	1,29E-11	0,661027898	1,05911117	-1,146404939	1,281947464
A_64_P141737	RGD1566325	5,85E-13	0,677778004	-1,244231187	-1,556900267	-3,082495183
A_64_P142046	Nudt15	4,84E-13	0,656944882	1,240879479	-1,384865052	-2,235256929
A_64_P142141	Cldn23	3,51E-13	0,704359338	-1,094776472	1,450854203	1,169392384
A_64_P142266	Tex38	1,25E-13	0,695625058	1,214587595	-1,373548505	-1,610778086

A_64_P142320	Zfp458	3,47E-14	0,746842803	-1,066403494	1,12547984	-1,455877843
A_64_P142625	Gprc5a	4,95E-24	0,873622214	1,181273845	-1,5733393	-1,301734276
A_64_P142809	Sipa1l3	3,50E-15	0,751577731	1,031795944	1,513538806	1,5486504
A_64_P142942	Scx	4,16E-29	0,925074452	1,024832235	1,484022742	-1,131929496
A_64_P142988	Tmprss9	1,99E-29	0,954877387	1,24095801	-1,397633288	1,140595859
A_64_P143186	LOC108353713	5,70E-18	0,804874123	-1,177593204	-1,187898771	-1,691696472
A_64_P144123	Olig2	3,03E-24	0,886315176	1,111669987	1,314329334	1,514115121
A_64_P144134	RGD1566029	3,76E-16	0,801912623	1,163836978	3,104688011	1,593969363
A_64_P144253	Shank2	4,60E-11	0,621224284	-1,291251585	1,407545296	-1,439609402
A_64_P144968	Calb2	7,22E-18	0,787487676	1,216995337	1,148899632	1,466393301
A_64_P145433	Ahr	1,56E-08	0,621429522	-1,173674704	-1,218800519	1,029459904
A_64_P146135		8,22E-12	0,620047028	1,090667954	-1,1146376	-1,352813236
A_64_P146243	Pou2f3	1,88E-21	0,855477914	1,216347809	-1,677877488	-4,560669514
A_64_P146964	Unkl	1,01E-15	0,725226062	-1,048129667	-1,046800929	-1,303493908
A_64_P146970	Cacng2	3,49E-17	0,805955506	-1,042475537	-1,003705514	1,797897246
A_64_P147164	Tubg1	3,62E-13	0,703996965	1,029364552	1,152394105	1,292925663
A_64_P147190	Fdps	3,24E-11	0,648875176	1,018775154	1,229139708	1,60069459
A_64_P147373	Gja5	1,83E-11	0,608939442	1,058974302	1,15060124	1,427168878
A_64_P147403	LOC100911864	1,20E-11	0,661954829	1,04181842	1,090324023	-1,600694072
A_64_P147769	Gucy1b2	1,67E-17	0,780814829	1,237693755	-1,196735433	-1,558909049
A_64_P148508	Prr7	7,09E-14	0,756373858	1,171140245	1,187987116	1,519495487
A_64_P148535	Napepld	9,86E-13	0,746091641	1,475189416	1,099239209	-1,369476253
A_64_P149300	Cacnb4	1,56E-22	0,878848619	-1,241941334	-1,113930389	-1,208793787
A_64_P149689	RGD1305733	3,60E-09	0,623888327	-1,264262822	1,213121811	-1,121870529
A_64_P149735	Sqstm1	1,31E-15	0,76065426	-1,053945086	1,249425797	-1,313262958
A_64_P150181	Rab23	5,98E-20	0,821742577	-1,174070746	-1,071656674	-1,234406129
A_64_P150338	Tmem132c	1,85E-13	0,711452001	-1,203098384	-1,184203966	1,036686765
A_64_P150397	Tex38	1,66E-15	0,740463961	1,208486193	-1,751231949	-1,572305324
A_64_P150509	Ccr10	1,88E-17	0,795935719	1,229192935	-1,307212931	-1,203873848
A_64_P150814	Rnf19a	4,09E-15	0,78236148	-1,177583686	1,425345829	-1,048884797
A_64_P150876	Cst6	8,20E-11	0,657499619	-1,080452706	1,450118204	-1,294498156
A_64_P151071	Hmgcs1	2,67E-19	0,811691073	-1,012281341	1,348485913	2,376197678
A_64_P151627	Trim16	8,64E-20	0,845817465	1,254955413	-1,230615393	1,154117843
A_64_P152214	Rtn4r	8,01E-11	0,613336054	1,201161821	1,523115038	1,138207625
A_64_P152252	Fscn2	1,31E-26	0,907196407	1,234988399	-1,105917871	-1,754097572
A_64_P152878	Shisa8	2,72E-19	0,795478276	1,266386297	1,147437839	1,1734449
A_64_P152919	Gprin3	1,67E-10	0,626189694	-1,011582399	-1,441595297	-1,309494164
A_64_P152993	Sipa1l1	7,59E-14	0,720995582	-1,000768094	-1,086521257	1,455744431
A_64_P153002	Gpr20	2,19E-26	0,913567671	1,313478604	1,566512017	1,597455231
A_64_P153091	Sel1l3	9,96E-13	0,671383127	1,000470765	-1,167038369	-1,270691831
A_64_P153105	RGD1562890	1,85E-15	0,719199589	1,165502161	-1,054902439	-1,807292854
A_64_P153282	Fpr2l	7,24E-17	0,800438915	1,218122733	-1,925405771	1,229284462
A_64_P153363	Dmrtc1b	1,45E-17	0,797892114	1,253503479	-1,104520792	-1,040009892
A_64_P153442	Zfp717	2,80E-17	0,758474691	-1,011212765	-1,047783214	1,49764307
A_64_P153533	Ubtd1	1,60E-14	0,717882746	-1,039030159	1,161076894	1,387689792
A_64_P153694	LOC108348293	2,33E-18	0,837751856	-1,125874685	1,143667571	-1,221389578
A_64_P154034	Hapln4	2,62E-18	0,795238622	1,046170039	-1,083750456	1,370970093
A_64_P154082	Tp53bp2	4,29E-10	0,634505697	1,096815638	-1,245057007	1,060926014
A_64_P154292	Hnrnpa3	6,49E-10	0,606275926	-1,018508671	1,237848803	1,194749679
A_64_P154430		1,45E-11	0,698898255	-1,146313354	-1,169482392	-1,790106833
A_64_P154525	Ypel4	3,83E-16	0,771458212	-1,054186696	1,148487037	-3,967210283
A_64_P154952	Nr4a2	8,01E-15	0,760781268	-1,390407121	1,567216965	-2,077689548
A_64_P155265		4,19E-30	0,916148794	-1,00822281	-1,097050121	-1,824381948
A_64_P155311	Prss22	1,40E-20	0,844200963	-1,007615584	2,482135613	1,030996689
A_64_P155895	Thnsl2	8,30E-11	0,612819707	-1,034087199	1,287852066	-1,516711972
A_64_P155983	Vegfa	8,19E-12	0,723151336	-1,448083255	-1,022738844	-1,222916584
A_64_P156303	Cyb5d2	1,34E-19	0,830438436	1,173609729	-1,03757963	-1,400812507
A_64_P156632	Itpr1	5,85E-10	0,630012598	-1,187906816	1,043876414	1,072627101
A_64_P156762	Gpr156	1,80E-11	0,634168753	1,079985715	1,240342103	-1,652729363
A_64_P156791	Hagh	2,54E-15	0,736351925	1,08358695	1,214462161	-1,093445777
A_64_P157029	Usp10	2,81E-14	0,690356836	-1,015199039	1,114240901	1,443590596
A_64_P157044	Cbx2	3,75E-21	0,85168597	1,137159474	1,249865195	1,423211102
A_64_P157131	Pkig	1,12E-10	0,608499321	-1,109086847	1,271364416	1,011054523
A_64_P157296	Efcab8	7,14E-16	0,78207109	1,391487723	1,257779921	-1,164963295
A_64_P157385	Dus4l	2,01E-17	0,779341167	1,724524052	-1,620233318	1,274422904
A_64_P157490	Luc7l3	1,12E-15	0,74417388	1,048991499	-1,23873939	1,572055531
A_64_P157873	Nhlh2	1,54E-11	0,611349625	1,226673889	-1,018506986	-1,72562203
A_64_P158441	Rfx2	1,32E-15	0,742644587	-1,120300743	-1,133968832	-1,252299779
A_64_P158504	Klhl2	5,76E-11	0,641096151	-1,206542945	1,080655476	1,381528039
A_64_P158778	Ocm2	3,67E-10	0,636759568	1,625716877	2,433459999	-1,346292753

A_64_P158883	Mxd1	5,60E-10	0,608500245	-1,119290425	1,102251874	-1,59854842
A_64_P158945	Tcap	1,09E-22	0,880493391	-1,115237347	1,231019658	-2,278028307
A_64_P159275	Inip	3,75E-17	0,805413312	-1,04151742	1,156196291	1,134765465
A_64_P160008	Nxnl2	1,90E-14	0,768733953	-1,077128856	1,100617887	1,305377638
A_64_P160072	RGD1309651	1,06E-14	0,802504832	-1,075932876	1,615555978	-1,600414162
A_64_P160091	Gpr62	5,39E-22	0,872988534	1,085544366	-1,169563765	-1,337654404
A_64_P160096	Mei1	1,19E-13	0,673889358	1,377760585	-1,350002707	-1,576968781
A_64_P160373		1,46E-15	0,759663231	1,202213254	-1,363701162	-2,960949633
A_64_P160393	Tcf4	2,81E-10	0,618713208	-1,155794348	-1,062174206	-1,600030482
A_64_P161057	Actr3b	2,35E-13	0,688480381	-1,141907714	1,08192043	-1,623095964
A_64_P161265	Zfand2b	5,04E-16	0,825836404	1,163974127	1,186807583	1,199089047
A_64_P161354		2,52E-11	0,604353856	1,244735811	1,310228717	2,329907049
A_64_P161630	Amigo3	3,50E-22	0,852322588	1,1081294	1,142249333	1,339666224
A_64_P162760		6,48E-09	0,654296236	1,207131493	1,202331581	-1,236197623
A_64_P162940	Crxos1	1,04E-18	0,854202024	1,280982352	-2,098716068	-1,21866458
A_64_P163100	Ripply2	7,30E-16	0,781887953	1,021642686	1,097843861	-1,409344864
A_64_P163165		3,81E-15	0,750805746	1,473933219	-1,497994919	-1,98204988
A_64_P163244	Myom1	5,99E-10	0,607481811	-1,087861136	1,04091732	-1,240863369
A_64_P163698	Onecut2	9,15E-14	0,78407322	-1,038080871	-1,054102383	1,71207225
A_64_P163822	Cks2	6,20E-12	0,670312376	-1,071077976	1,25461856	-1,008368761
A_64_P164215		8,77E-12	0,704885628	-1,041951684	-1,42549012	1,160103996
A_64_P164240		1,47E-12	0,687874428	1,063721433	-1,139700239	1,166819094
A_64_P164682	Etl4	4,15E-12	0,675293632	-1,049469897	-1,053015754	-1,770453705
A_64_P164961	Cyp2j3	4,54E-10	0,633678453	-1,022338353	-1,062504256	-2,067299093
A_64_P165913	Syt6	1,34E-27	0,929095258	1,318178806	-1,201820237	1,424213665
A_64_P165964	Hpca	3,69E-22	0,838593868	1,104889659	1,288974689	1,020693917
A_64_P166385	Kifc1	5,54E-12	0,691742707	-1,049233877	1,201581924	1,081595256
A_64_P204094	RGD1308134	1,25E-12	0,689761351	1,134614047	1,190627284	1,013757403
A_64_P236773	Nudt6	2,04E-23	0,854493903	1,227234759	-1,255818996	-1,789040069
A_64_P236835	Ccdc96	2,58E-16	0,757661253	-1,007767615	1,33883059	1,4534083
A_64_P237784	Rbm24	8,63E-30	0,947020639	-1,158739672	1,035213052	1,346695144
A_64_P246030	Pfkfb1	1,81E-10	0,646684508	1,22091008	-1,841126086	-1,247122463
A_64_P247541	Pcdh7	1,07E-19	0,817907768	-1,125734962	-1,129387499	-1,221660783
A_64_P264316	Mfsd5	4,15E-12	0,675312803	-1,061451954	1,219470121	1,14593661
A_64_P271601	Nol4l	3,67E-10	0,636730149	1,063816272	2,671988317	1,072804505
A_64_P280765	Cd44	8,11E-09	0,631667418	-1,378275711	-1,847180771	1,207932719
A_64_P281840	Nrg1	3,28E-11	0,706930438	1,368872456	1,074220174	-1,426875836
A_64_P307602	Tmem116	1,27E-16	0,745006832	1,236059541	1,548556466	-2,034498617
A_64_P312847	Zfp618	2,64E-19	0,811777196	1,11531752	-1,125887892	1,208230022
A_64_P327151	Rxrb	6,50E-10	0,628482816	1,154843719	1,230108703	-1,146860756
A_64_P370146	Nrip1	7,78E-10	0,60351711	-1,200027319	-1,057593372	-2,722578893
A_64_P386965	Ccdc191	1,77E-25	0,897729952	-1,0552849	-1,055631005	-1,369119847

Supplementary Table 4. Lists of genes with significant temporal expression changes in CGNs during rescue by SP (K5 + SP vs K5).

ProbeName	GeneSymbol.x	p-value	R-squared	K5_SP_vs_K5_05h	K5_SP_vs_K5_1h	K5_SP_vs_K5_3h
A_42_P453976	Ifitm6	2,45E-18	0,849122394	-1,060851677	1,088543523	1,797760081
A_42_P457003	Pdgfra	4,38E-13	0,658164838	-1,171915058	-1,142897366	-1,118185483
A_42_P461794	Clic3	1,19E-23	0,869492097	2,075140861	1,504242636	1,483977893
A_42_P462257	Rprml	2,45E-31	0,953960826	1,075478035	1,119658118	-1,045325031
A_42_P463998	Abcc6	9,33E-16	0,763663563	-1,162333991	1,179056315	1,569801344
A_42_P464378	Itih3	6,43E-14	0,722735839	1,158130698	1,115343879	-1,347033108
A_42_P471507	Unc50	6,25E-17	0,769956405	-1,115490427	-1,084799232	-1,01780693
A_42_P471608	Kif16b	3,73E-13	0,6336236	-1,17431447	-1,143385524	1,330863353
A_42_P473398	Cxcl1	2,97E-26	0,912550029	-1,053409153	2,032382434	2,851477799
A_42_P473425	Unc13d	5,48E-19	0,86832868	2,022084519	1,121722216	1,021940499
A_42_P473594	Egr2	3,03E-26	0,895081447	1,349467189	2,314300912	2,886768465
A_42_P484738	Ctgf	2,06E-21	0,876696956	-1,155110941	1,878860194	1,699506771
A_42_P491454	Gnat1	2,92E-23	0,886339724	1,029276694	1,115176255	1,392144726
A_42_P492882	Nckipsd	1,80E-11	0,634187812	1,122335231	-1,028061468	1,217203907
A_42_P493925	Usp48	3,88E-14	0,745738479	1,028742216	-1,074820998	-1,090520666
A_42_P494276	Armc9	1,06E-09	0,642175204	-1,026712868	1,021245825	-1,172534864
A_42_P499221	Fgd5	5,70E-14	0,704336224	-1,234516013	-1,182762622	-1,19977053
A_42_P501233	Adra1d	1,17E-24	0,907811724	-1,022372652	1,013865215	1,34458774
A_42_P502621	Avil	9,70E-18	0,767493965	1,650532298	1,557995029	1,765946333
A_42_P505320	Cipc	1,75E-13	0,669286368	-1,140566204	-1,277660607	-1,269198921
A_42_P506402	Slc25a25	3,29E-12	0,678163418	1,20674196	1,193462057	1,282435378
A_42_P508921	Aqp3	6,40E-17	0,801384405	1,194170529	-1,069806949	1,600612188
A_42_P522194	Atp2a3	5,03E-27	0,918255793	-1,373276517	-1,207926923	1,036486841
A_42_P522638	Pqlc3	3,54E-13	0,704239891	-1,034050031	-1,11459505	-1,289440944
A_42_P523517	Ttyh2	3,17E-19	0,810512686	-1,092468807	-1,159068411	-1,329662268
A_42_P535644	Adamts1	4,73E-13	0,700981015	-1,087695846	1,364291176	1,252810944
A_42_P536292	Ngb	6,38E-25	0,909944188	1,186175739	1,064794644	1,131021489
A_42_P536702	Sftpc	2,61E-23	0,886838817	1,191332182	-1,364787171	-1,341661754
A_42_P536741	Derl3	4,78E-11	0,620670756	1,09527385	-1,245626895	1,437432699
A_42_P542960	Rerg	4,20E-10	0,655246957	-1,074247165	1,144911116	-1,192395024
A_42_P547036	Pak4	2,91E-10	0,697205897	1,124620589	1,087768105	1,210571901
A_42_P547629	Fam49a	1,15E-11	0,719245696	-1,164608118	-1,171324126	-1,29092002
A_42_P548410	Acot1	1,18E-12	0,69045879	-1,096764979	-1,152772106	-1,801999936
A_42_P554157	Dnajb11	4,36E-14	0,685415329	1,004503172	1,002412385	1,278321135
A_42_P555140	Izumo4	5,38E-13	0,718463966	1,093981158	1,079109383	1,275968347
A_42_P559038	Plekhg2	1,57E-11	0,611053723	-1,126813099	1,396302697	1,113441931
A_42_P567527	Traf4	4,67E-12	0,673855816	-1,274748862	1,055107123	-1,143494558
A_42_P588785	Mthfd2	8,01E-20	0,81982588	1,144468467	1,096518414	1,066548187
A_42_P589201	Slc15a4	4,20E-11	0,622503408	-1,015221352	-1,105507907	-1,299404755
A_42_P599074	Srsf3	5,69E-12	0,625071293	-1,151169447	-1,230862818	-1,289047208
A_42_P604033	Eaf2	8,62E-18	0,801815603	-1,040585551	-1,26429855	1,308469776
A_42_P619806		1,10E-28	0,922310651	1,343510308	1,198570383	-1,079239009
A_42_P625147	Pglyrp1	4,12E-10	0,635105336	1,129865031	1,197342654	1,130733003
A_42_P629321	Wisp2	4,74E-11	0,664771209	-1,158849406	1,036077188	1,083590265
A_42_P636761	Gzmm	4,82E-15	0,808847477	1,076423923	-1,036434755	1,419606554
A_42_P637189	Apln	1,50E-19	0,815644541	-1,018192434	1,061246643	1,594677401
A_42_P648475	Plxnc1	8,43E-14	0,700041456	-1,073978409	-1,157176909	-1,217727224
A_42_P664544	Rftn2	4,98E-12	0,673053345	-1,29295707	-1,131617357	-1,198387915
A_42_P668682	Dusp6	2,35E-22	0,825786822	-1,004818631	-1,068478888	1,496045548
A_42_P673905	Mrnip	3,96E-20	0,861635086	1,007973646	1,005523121	-1,268987924
A_42_P676553	Tnni3	9,18E-13	0,672374677	1,299201412	1,24817403	1,770571654
A_42_P682589	Nr4a1	1,03E-28	0,951719014	-1,053491117	2,276225249	2,296712225
A_42_P683837	Serpinb6b	3,05E-10	0,617484041	-1,130392924	-1,017825111	1,597657044
A_42_P683840	Ccl27	3,87E-20	0,850478647	1,011452763	1,038319869	1,183125282
A_42_P684538	Slc2a1	1,72E-17	0,796629922	-1,124377142	1,217412842	1,313050624
A_42_P684885	Atp12a	6,04E-18	0,788865975	-1,041025869	-1,14370953	-1,348585041
A_42_P685330	Ap1s2	1,89E-16	0,760380359	-1,003410138	-1,205757597	-1,316300334
A_42_P686234	Muc4	2,84E-10	0,618531385	1,384108644	-1,054251446	1,656293838
A_42_P686879	Siglec1	6,32E-10	0,649523091	1,072887796	1,177355143	1,414087771
A_42_P688266	Arap3	1,98E-12	0,684276925	1,391954613	1,221486967	1,30258715
A_42_P694928	Arl5c	5,61E-19	0,834376323	-1,071952211	-1,03122411	1,572691752
A_42_P695042	Csf1	2,40E-18	0,83756443	-1,098676027	1,015560825	1,325896443
A_42_P695401	Ccl2	2,50E-33	0,942131263	-1,035890235	1,001230615	1,945563408
A_42_P712297	Fa2h	3,63E-14	0,687492189	-1,228272979	-1,078805902	-1,183357568
A_42_P714311	Ccl3	1,78E-16	0,793430339	1,020942234	1,038437245	-2,223585378

A_42_P718331	Sstr3	6,97E-37	0,975058259	1,300042151	1,140644809	-1,012538033
A_42_P723173	Id1	1,08E-31	0,950760412	1,005714068	1,02190103	1,196161344
A_42_P723540	Dyrk3	2,60E-20	0,827108147	-1,182195333	-1,235756333	-1,280267296
A_42_P726234	Tex33	6,40E-09	0,614973196	1,915907309	1,032323815	1,397861524
A_42_P729012	Cercam	1,35E-13	0,714857127	1,02692999	1,161986462	1,393089946
A_42_P734476	Ttll3	1,77E-15	0,719676831	1,286140559	1,319193157	1,287985838
A_42_P746838	Spib	5,54E-30	0,923364885	1,461946635	1,100467547	1,680930295
A_42_P754888	Dlk1	1,65E-11	0,635330857	4,949481299	10,53143811	7,853028049
A_42_P762829	Cebpd	7,83E-26	0,900794423	1,021052608	1,263046593	1,391224332
A_42_P764359	Serbp1	4,44E-10	0,611960788	-1,090811802	-1,122604581	-1,192786827
A_42_P768355	Hrk	1,27E-34	0,953265179	1,101530224	1,18482655	-1,312301577
A_42_P768883	Perp	5,81E-17	0,787097571	-1,241881312	-1,213627534	-1,778994154
A_42_P791436	Gfra2	2,64E-19	0,826102124	-1,050794297	-1,087940694	1,328797883
A_42_P794613	Mvd	2,53E-11	0,629516431	-1,269518088	-1,093062767	-1,004432127
A_42_P797218	Gpt	1,99E-09	0,611722902	1,299314249	1,18934666	1,669489412
A_42_P802358	Fam110b	9,61E-10	0,60030019	-1,052367035	-1,108951772	-1,2596648
A_42_P803810	Myo5c	9,61E-11	0,634015497	-1,686357929	-1,305279396	1,743906752
A_42_P812805	Smoc2	9,92E-09	0,608037116	1,272237917	1,007940502	-1,195401132
A_42_P816878	Rgs7bp	6,46E-10	0,606328068	-1,501263391	-1,32255346	-1,192844397
A_42_P820657	Il1r2	8,45E-13	0,747680923	-1,028735416	1,024567469	-1,168233578
A_42_P821898	Efcab7	2,14E-14	0,734007734	-1,177900646	-1,087867967	-1,148373704
A_42_P828242	Lgr4	9,43E-12	0,685275637	-1,212443786	-1,238830699	-1,075988889
A_42_P829031	Coasy	2,27E-13	0,70919253	1,179048227	1,114724363	1,043178326
A_42_P841193	Ddit4l2	2,67E-21	0,875440963	1,35041303	-1,532665354	-1,819724187
A_42_P842823	Reep6	5,65E-09	0,637185947	1,076168233	-1,156766395	1,14206719
A_43_P10047	Slc20a2	7,41E-14	0,721247418	1,116436515	1,013557387	-1,086304192
A_43_P10102	Spry4	1,06E-17	0,814599362	1,093972483	-1,044944112	2,088289751
A_43_P10498	Bag2	6,17E-28	0,924520993	1,118604284	1,087394262	1,18561442
A_43_P10824	Cib2	5,79E-10	0,630169569	-1,048393679	-1,105007985	-1,292451622
A_43_P10935	RGD1562339	2,49E-09	0,649420917	-1,210925354	-1,196660883	-1,058459831
A_43_P11044	G0s2	1,50E-20	0,855785142	-1,061227782	1,107838997	1,376588822
A_43_P11144	Pdrg1	5,77E-10	0,608049886	1,347383717	1,142037937	1,122852185
A_43_P11441	Atp4b	1,80E-19	0,853197569	1,516135231	-1,602626803	1,101942695
A_43_P11472	Hmox1	3,61E-09	0,602466186	-1,166372605	1,052108633	-1,524818062
A_43_P11527	Adm	4,16E-18	0,807159068	-1,00483004	1,14930906	1,247494331
A_43_P11560	Aqp5	1,97E-12	0,783598759	1,69971978	1,099060106	-1,050009332
A_43_P11563	Cnr1	1,94E-11	0,633168463	-1,547254931	-1,381893496	-2,144024765
A_43_P11580	Abcc2	1,21E-13	0,734328621	1,03541743	-1,374813373	1,06294349
A_43_P11616	Atf3	2,58E-17	0,845844687	-1,053392756	1,319174403	-1,294979679
A_43_P11685	Id2	4,67E-17	0,830209422	-1,123155343	-1,035106646	-1,468320012
A_43_P11754	Akr7a3	2,24E-21	0,827800924	1,070620513	-1,169040566	-1,301073532
A_43_P11820	Kcnj8	1,31E-13	0,750344385	1,002473924	1,190212287	-1,005502599
A_43_P11861	Dio3	1,36E-24	0,898876428	1,405999645	-1,084124125	-1,009686348
A_43_P11932	Nr4a3	1,78E-34	0,961556291	-1,291590917	1,117031678	1,65987799
A_43_P11985	Ccl20	1,34E-21	0,814817137	1,172191654	1,213552124	1,831283371
A_43_P12018	Kcnn1	1,82E-16	0,760713135	1,302097779	1,518759665	1,659026004
A_43_P12160	Unc5b	2,88E-12	0,634166721	-1,420877964	1,050022171	-1,732953051
A_43_P12384	Birc3	2,05E-15	0,694758875	-1,196515155	1,272748707	1,544538572
A_43_P12451	Slc16a3	1,09E-14	0,740692149	-1,00479835	1,09936456	1,412486687
A_43_P12460	Lrrn3	7,38E-18	0,802966625	-1,202274549	-1,14927544	-1,080453422
A_43_P12559	Camkk2	9,77E-13	0,671617743	1,183519683	-1,095205067	-1,440300158
A_43_P12584	Nfyb	3,65E-13	0,739998929	-1,082266556	-1,112604925	-1,223731102
A_43_P12716	Kalrn	1,41E-13	0,645893102	-1,18723403	-1,02486119	1,60416412
A_43_P12827	Nme3	1,04E-14	0,758335556	-1,098205324	-1,082685694	-1,127108483
A_43_P12908	Klf4	1,21E-23	0,907266589	-1,040555799	1,204656976	1,332697703
A_43_P12967	Cap2	1,96E-14	0,783444359	1,019671737	1,040201705	-1,19027943
A_43_P13023	Neu3	1,35E-14	0,71965811	-1,039295918	-1,337181824	-1,373489841
A_43_P13057	Baiap2	7,82E-13	0,650961657	-1,123243326	-1,062670552	-1,191887691
A_43_P13109	Cyp26a1	1,97E-13	0,74629897	-1,22029717	-1,386010517	-1,169271843
A_43_P13355	Bambi	1,21E-16	0,781136286	-1,253888939	-1,093272144	-1,327389236
A_43_P13931	Grem2	7,78E-11	0,636960134	-1,143523753	-1,22480088	-1,412342214
A_43_P14690	Cdk17	1,81E-12	0,640206616	1,03171563	-1,059845842	-1,139799875
A_43_P14752	LOC102554727	8,04E-13	0,714043632	1,079046697	-1,003802386	-1,347364292
A_43_P14809		1,87E-09	0,633808146	1,228829977	1,171058578	1,481967512
A_43_P14872	Sdc1	2,79E-21	0,840669296	1,129422497	-1,111310326	-1,358346271
A_43_P14882	Errfi1	8,14E-21	0,847325477	-1,121897964	1,315864658	1,489890806
A_43_P14911	Il1b	2,68E-14	0,765574431	-1,11812682	1,346269741	-1,294728858
A_43_P15154	Ihh	2,29E-18	0,81143918	1,133622085	-1,335953404	1,75543953

A_43_P15205	Srf	2,41E-21	0,85412917	1,092698476	1,154320526	1,273689358
A_43_P15390	Smad7	2,05E-14	0,783034366	1,261943314	1,162093025	1,276718301
A_43_P15447	Bglap	1,21E-15	0,743496572	1,257198321	1,129010442	1,260042329
A_43_P15544	Ltb4r	1,70E-11	0,609931184	1,55529996	1,079365141	1,585513432
A_43_P15548	Gabra6	3,08E-13	0,741748159	-1,363235977	-1,22425955	-1,123454596
A_43_P15600	Rab29	3,12E-17	0,792019025	1,061072423	1,016031862	1,173959184
A_43_P15701	Rab13	5,23E-11	0,642432356	-1,05626005	-1,042188132	-1,569543398
A_43_P15837	Ntrk2	5,08E-13	0,736576686	-1,272535232	-1,214048789	-1,085156676
A_43_P15933	Chrm4	2,32E-12	0,720167366	1,009144283	1,079673325	1,410304657
A_43_P16449	Pelo	5,38E-11	0,682635509	1,153945931	1,038366889	-1,087062847
A_43_P16491	Col16a1	9,36E-24	0,891163634	1,20733799	1,100263514	1,309090602
A_43_P16529	Gadd45b	2,49E-17	0,793753291	-1,144974033	1,020104408	-1,232614109
A_43_P16567	Glyctk	1,07E-21	0,832333544	1,333028532	1,047061658	1,10565087
A_43_P16989	Snx9	1,68E-10	0,626125629	-1,328234736	-1,142499857	-1,262546374
A_43_P17060	Them5	4,81E-18	0,773266417	1,6554036	1,098669936	1,170190004
A_43_P17061	Pcgf3	1,83E-10	0,601236514	-1,079192136	-1,13581668	-1,253059656
A_43_P17681	Jmjd6	2,60E-11	0,603913998	1,171761489	-1,18204745	-1,201696004
A_43_P18356	Ift57	7,48E-10	0,604113042	-1,180558884	-1,141258178	-1,138323977
A_43_P18571	Tekt4	2,37E-18	0,824971662	1,020590275	-1,040261444	-1,26576177
A_43_P19743	Krtcap3	5,30E-18	0,819487294	1,06984938	1,148423741	1,28151416
A_43_P21118	Klhl24	7,44E-14	0,721201993	-1,580841735	-1,446135484	-1,594813442
A_43_P21279	Pip5k1b	6,66E-10	0,605872172	-1,203656805	-1,069051679	-1,054264037
A_43_P22225	Mdga1	3,27E-22	0,87538247	1,341206649	-1,07442302	1,207924179
A_43_P22811	Cd6	1,40E-22	0,927130608	1,660092925	-1,140835231	1,066693207
A_43_P23014	Lpxn	4,18E-11	0,622577919	-1,095408257	1,008869143	-1,513919873
A_43_P23155	Mcm10	6,18E-11	0,617037221	-1,313041914	-1,415521675	-1,174526798
A_43_P23274	Dmrtc1a	2,87E-18	0,777445995	1,134403123	-1,364886952	-1,344997042
A_44_P1004376	Ins2	2,16E-15	0,737951764	1,700218739	-1,008215712	1,759645364
A_44_P1004840	Tnfsf9	7,51E-10	0,604055585	1,169988355	1,04257195	-1,542489288
A_44_P1006068	Exoc3l4	2,53E-18	0,824547262	1,294439951	1,093323365	-1,444932601
A_44_P1007215	Upp1	4,27E-12	0,694842222	1,175914957	1,016536214	1,164506603
A_44_P1011386	Znhit6	1,49E-09	0,67538751	-1,091587314	-1,02496756	1,304305931
A_44_P1012556	Stbd1	8,51E-10	0,700405534	1,147194665	1,183437941	1,219984831
A_44_P1015754	Slc22a3	1,43E-16	0,822389127	-1,237393422	-1,242400349	1,542212963
A_44_P1017595	Mospd4	1,32E-09	0,618010056	1,007853384	-1,053363591	1,67225849
A_44_P1018622	Rflnb	5,57E-18	0,772083308	-1,021312834	-1,167866433	-1,172263668
A_44_P1019407	Rnf103	5,99E-11	0,640568409	-1,129506302	-1,138231523	-1,152237487
A_44_P1019480	Cdc42ep2	5,00E-15	0,765090953	-1,101070038	1,14805599	-1,383659597
A_44_P1021808	RGD1561796	4,93E-08	0,622600385	1,30750589	1,006935574	1,145011787
A_44_P1022002	Ccl7	7,20E-32	0,934587173	1,060851155	1,002019686	1,809829888
A_44_P1024065	Hdac5	5,36E-21	0,881850931	-1,168220639	1,135749949	-1,111430349
A_44_P1024627	Kcnv1	2,78E-23	0,886552252	-1,403221687	-1,433815646	-1,250262388
A_44_P1025790	Gsto1	2,71E-13	0,664017486	1,03436348	-1,002624945	-1,19325094
A_44_P1028743	Cfc1	1,67E-22	0,867959854	1,493818386	-1,263176941	-1,38176196
A_44_P1030258	Cnr1	2,17E-17	0,778723157	1,04071857	-1,073822593	-1,307757225
A_44_P1034541	Pim3	3,65E-18	0,822059173	1,283091996	1,089343615	1,224627725
A_44_P1034668	Shroom1	1,42E-25	0,898565077	1,345585515	1,169303436	-1,113530751
A_44_P1035071	Nmt2	1,42E-11	0,699109593	-1,015538919	-1,099382817	-1,212805659
A_44_P1037041	Map3k6	1,13E-27	0,897559618	1,556339312	1,025176938	1,260375923
A_44_P1037706	Glod5	4,46E-20	0,860991454	1,495032731	-1,511092033	-1,342709215
A_44_P1039809	Rasip1	1,40E-12	0,707833068	1,226183716	1,227794624	1,149626004
A_44_P1039927	Eepd1	1,32E-17	0,764915506	1,199846068	1,201524744	1,408604308
A_44_P104054	Crhbp	4,65E-31	0,942537979	1,195314702	1,323801821	1,49912827
A_44_P1043302	Crb3	2,94E-15	0,785187838	-1,303139689	-1,2015023	-1,095711813
A_44_P1044046	Ndufaf1	5,45E-25	0,893355505	1,111696887	-1,140507774	-1,201951647
A_44_P1045734	Stac3	7,10E-14	0,771845705	2,05546928	1,447889642	3,295497326
A_44_P1047924	Herpud1	9,88E-22	0,898196144	1,031819638	-1,100953953	1,032366548
A_44_P1050200	C1qtnf12	1,91E-17	0,823802738	1,309938391	-1,069174385	1,606742001
A_44_P1055082	Dyrk2	1,23E-18	0,800868363	1,069091253	-1,041026306	1,237283496
A_44_P1057412	Ddx25	8,86E-16	0,764115857	1,251078716	1,023449299	1,100990164
A_44_P1058112	Yars2	8,46E-18	0,801954885	1,094609402	1,052996696	1,790960402
A_44_P1058570	Sigirr	5,32E-17	0,80278447	1,241721582	1,109471812	1,426646596
A_44_P109927	Ahsa2	2,17E-11	0,654232207	1,090978921	1,059616595	1,329497146
A_44_P114692		4,14E-17	0,804673899	1,097389657	1,079876127	1,131015452
A_44_P114788		9,33E-11	0,611134308	1,096575419	1,454830302	1,541875812
A_44_P115192	Slc5a5	1,31E-09	0,694691105	-1,168869016	-1,610367509	1,357964674
A_44_P118724	Arc	3,20E-24	0,89552772	-1,002560667	1,610556586	1,372214722
A_44_P122386	Hspb3	2,31E-18	0,811368271	1,163881701	-1,072695348	2,360909948

A_44_P128062	Syt17	1,07E-10	0,692277315	1,09562469	-1,137547312	1,346396004
A_44_P128488	Pbx4	1,74E-13	0,669356452	1,196375744	1,3448848	1,053242345
A_44_P133335		1,97E-25	0,906037183	1,421692642	1,173229653	-1,125067063
A_44_P138301	B9d1	3,18E-10	0,616902601	-1,198591776	-1,082557233	-1,165571438
A_44_P144277	Pkn1	1,62E-10	0,603068155	1,141325125	1,224287876	1,163021672
A_44_P151482	Il22ra2	2,59E-11	0,629230568	-1,126388516	-1,510430927	1,444663309
A_44_P151582	Il17re	1,68E-16	0,742415305	1,334140721	1,128208256	1,254983068
A_44_P152275		3,57E-10	0,615164069	-1,44433405	1,039193555	2,478539035
A_44_P153774	Plce1	6,12E-11	0,617188916	-1,278231043	-1,209234248	1,005903815
A_44_P156262	Myod1	1,07E-20	0,832630999	-1,066429911	-1,083504586	1,823855585
A_44_P156966	Ube2q2l	7,25E-18	0,817314131	-1,183996446	-1,227697849	-1,152810683
A_44_P160846	Rnasel	1,81E-20	0,829390681	-1,467920911	-1,309259675	-1,374604417
A_44_P161770		5,94E-12	0,670849458	1,197880381	1,322538261	2,516506981
A_44_P166161	Adh7	1,02E-21	0,869875625	1,477554842	-1,06928703	1,097043703
A_44_P173568	Pop1	1,95E-18	0,812547603	-1,130858167	-1,046793059	-1,38963127
A_44_P174445	Lrrn2	4,65E-15	0,730430792	1,172927417	1,067124882	1,179660509
A_44_P179145	Dgkg	1,41E-10	0,628658868	-1,330017418	-1,285037277	-1,446868042
A_44_P185294		6,16E-32	0,946775522	1,585064425	1,064663034	-1,378819397
A_44_P187195	C1galt1	3,81E-15	0,797316672	-1,215993688	-1,188056416	-1,803336542
A_44_P189406	Spint1	6,87E-10	0,605404501	-1,39370061	-1,186554017	1,067932158
A_44_P192406	Dusp9	2,95E-27	0,926891303	1,468613534	1,03789372	1,314968864
A_44_P196146	Aplnr	5,23E-11	0,642431508	-1,725217302	1,02266803	-1,152579328
A_44_P198620	Nos3	1,42E-19	0,842876363	-1,288632066	-1,276526095	-1,103016674
A_44_P198705	H1f0	5,44E-22	0,908525853	1,180696037	-1,02390352	1,333765068
A_44_P202201	Grp	1,33E-22	0,879589088	1,360119423	-1,315122685	1,122996865
A_44_P203859	Piezo2	9,07E-10	0,601177517	-1,251086677	-1,16878094	-1,768808735
A_44_P210492	Syt2	6,59E-25	0,909833124	1,012481018	1,063505057	-1,289438174
A_44_P210736		7,49E-16	0,765607405	1,147726482	-1,099375547	1,966473827
A_44_P213175	Ccdc68	2,95E-13	0,685815734	-1,292717993	-1,200984174	1,834749495
A_44_P220610	Fahd1	1,43E-12	0,643307906	-1,192433548	-1,235910832	-1,006810202
A_44_P221077	Tmem100	1,07E-19	0,802216441	-1,214771781	-1,084704404	-1,201990047
A_44_P222124	Tssk3	2,17E-22	0,841636909	-1,30061548	-1,180700244	1,507204837
A_44_P222533	Actrt3	2,23E-14	0,751116003	1,270609103	-1,048603161	1,328600093
A_44_P224020	Kcna4	2,85E-31	0,943592452	-1,446885883	-1,174857874	1,02584531
A_44_P226658	Scnn1g	3,60E-28	0,918813445	1,017244394	1,030156217	-1,551802225
A_44_P227221	Syde2	1,20E-19	0,876227354	1,176474785	1,123198956	-1,285013575
A_44_P228942	Runx1	2,68E-18	0,810314111	1,145180303	1,160344422	1,963459309
A_44_P231243	Insig2	9,33E-11	0,655766369	-1,377584925	-1,261933302	-1,66182493
A_44_P231583	Kcnip3	1,20E-22	0,869594075	1,277714461	-1,01624966	-1,132056011
A_44_P236407	Lnx1	8,62E-24	0,900365503	-1,205428265	-1,163721974	-1,140683453
A_44_P236600	Lrrtm3	5,03E-24	0,884141799	-1,25131237	-1,127379813	-1,070468097
A_44_P237621	Txnip	2,09E-22	0,896160474	2,240469365	1,039134931	-1,293652201
A_44_P238257	Trip10	7,13E-22	0,834732239	1,08009458	1,142388841	1,286612393
A_44_P243004	Amigo2	8,10E-33	0,939592801	-1,081449681	-1,019805207	1,407849874
A_44_P243477		6,70E-15	0,818981266	1,35114065	-1,083156188	1,027474914
A_44_P243534	Pxdc1	5,49E-12	0,710365891	-1,095098469	1,038952895	-1,31299234
A_44_P246114	Tle1	1,00E-13	0,736212666	-1,504698504	-1,347692811	-1,299596866
A_44_P247880	Csf2	2,51E-24	0,876717145	1,170279879	1,719410725	3,192896881
A_44_P248090	Thbs2	1,91E-09	0,689639607	1,059523792	1,013020693	1,250981139
A_44_P252483	Ackr3	1,31E-19	0,830618454	-1,099132851	-1,074490703	-1,474418635
A_44_P252657	Pou6f1	8,50E-22	0,833692852	-1,037703611	1,041037833	1,292308624
A_44_P253208	Adamts9	2,01E-17	0,79543505	1,000693831	-1,04089092	1,27444914
A_44_P256677	Plcxd3	1,07E-11	0,702506766	-1,323634697	-1,180346834	-1,282383875
A_44_P260751	Nptx1	8,15E-28	0,923716062	-1,203471436	1,010495439	-1,402418079
A_44_P265544	Crybb3	4,49E-16	0,838485367	1,389478422	1,07913667	1,155432251
A_44_P269085	Chrna4	1,21E-13	0,673631157	1,03094781	1,150759071	-1,25921677
A_44_P269930	Mfsd2a	4,28E-10	0,634525367	-1,214577872	1,049786638	1,320202047
A_44_P269984	Kdm4d	4,95E-15	0,729808658	1,313750269	1,017053422	1,200910304
A_44_P273777	Masp1	2,43E-13	0,727011863	1,063708428	1,125151043	2,498167133
A_44_P277669	Myo19	4,23E-10	0,612678568	1,129181925	-1,078332308	1,552155803
A_44_P280786	Cyp2c11	1,99E-09	0,611735069	1,111352566	1,006115114	1,82254887
A_44_P282164	Pdia2	1,68E-27	0,921595318	1,536240991	1,32302508	1,808097781
A_44_P283176	Spats1	1,15E-17	0,799688798	1,136773525	1,031638053	1,769605531
A_44_P283790	Mfhas1	3,06E-13	0,685406098	-1,335401902	-1,272115973	-1,243188911
A_44_P284067		1,41E-10	0,670081942	-1,093070982	1,033339558	1,203302125
A_44_P284753	Ankrd1	1,78E-14	0,753266058	-1,164550571	2,060866483	1,584979851
A_44_P288241	Medag	8,07E-16	0,747262089	-1,140330928	-1,280621427	-1,72016541
A_44_P288982	Zbtb8a	3,28E-16	0,736157867	-1,214258883	-1,04235116	-1,278474114

A_44_P290187	Sfrp4	1,89E-13	0,729657452	-1,744097218	-1,069198765	1,135633695
A_44_P291028	Arhgef19	1,57E-17	0,837492777	-1,41047142	-1,192082048	-1,044866265
A_44_P292980	Slitrk1	1,87E-14	0,752840373	-1,444006954	-1,198376288	-1,345119521
A_44_P293315	Bbs4	2,28E-09	0,609636454	-1,369619265	-1,16275368	-1,37308603
A_44_P307978	Cited2	3,72E-13	0,683141076	1,296128665	1,287217317	1,299519966
A_44_P309455	Fam207a	7,45E-15	0,744415627	1,182896918	1,008178223	-1,090944932
A_44_P311106	Pou3f3	2,52E-18	0,778481314	-1,052997835	1,080982339	1,340668125
A_44_P311620	Ahnak	2,16E-07	0,617074443	1,022311319	1,21142635	-1,395506196
A_44_P313272	Slc6a13	8,95E-12	0,704645209	-1,641169995	-1,340419472	1,033233769
A_44_P316319	Shkbp1	4,30E-16	0,770455353	1,204441894	1,225338708	1,282164011
A_44_P316342	Det1	1,43E-13	0,69414636	-1,582572113	-1,003173373	-1,056986683
A_44_P316611	Pknox1	8,06E-13	0,673939582	-1,15365353	-1,306594589	-1,470726618
A_44_P322860	Camkk1	6,16E-12	0,623993167	-1,162990342	-1,129611026	-1,16371943
A_44_P325189	Rcan2	4,96E-16	0,769212532	-1,219894006	1,189727433	1,249289523
A_44_P328046	Masp2	2,35E-23	0,896404427	1,215769642	1,040256628	1,752906081
A_44_P328489	Rprd1a	1,39E-09	0,617152777	-1,287582434	-1,361381547	-1,287455529
A_44_P330594	Prkd2	2,25E-09	0,631001934	-1,601902485	-1,135947059	1,386405862
A_44_P332896	Kctd14	4,92E-19	0,791083567	1,089734324	1,046724555	-1,527141496
A_44_P334715	Klc3	2,08E-15	0,772896884	-1,371658005	-1,211077047	1,449247905
A_44_P334736	Edn1	2,60E-16	0,757568535	-1,166522957	2,01174545	2,229797087
A_44_P335578	LOC108348348	1,55E-16	0,779101875	2,093418148	1,423536776	1,221919463
A_44_P339535	Tfpi2	5,03E-12	0,651067762	-1,19217937	1,808515939	2,927022772
A_44_P342538	Tcaf1	1,21E-14	0,720787093	-1,302366258	-1,163803323	-1,322200193
A_44_P349475	Cdh2	6,95E-10	0,627503551	-1,236779996	-1,125837098	-1,082599908
A_44_P349553	Col6a1	9,15E-10	0,623437642	1,030612058	1,232828527	-1,031046286
A_44_P351211	Phlda1	3,30E-22	0,864566884	-1,140089615	1,349508073	1,400482154
A_44_P351723	Cldn10	4,09E-12	0,69536705	1,220099449	-1,165403548	1,696390254
A_44_P352331	Bves	2,11E-09	0,631945262	1,038046733	1,052982434	1,874188878
A_44_P353157	Tmem30b	5,68E-16	0,78398024	1,403784958	-1,46554354	1,668232997
A_44_P354078	Hist1h1a	2,77E-10	0,640737498	1,165802475	-1,051937915	-1,53915716
A_44_P355073	Adssl1	2,49E-10	0,699165401	1,234827379	1,098408882	-1,128279156
A_44_P356829		3,21E-12	0,698217543	1,044260826	-1,085156491	1,912501633
A_44_P357870	Cpt1a	7,04E-24	0,901147006	-1,015719765	-1,24869291	-1,817608592
A_44_P358227	Mapk15	4,26E-12	0,653216844	1,578344986	1,201409971	2,225442769
A_44_P358974	LOC316124	1,32E-12	0,708465665	1,000095243	1,575329611	1,83662616
A_44_P359684	Nfkbiz	7,40E-13	0,651645672	1,045943745	1,543387482	2,170662094
A_44_P363291	Kcnk10	1,75E-11	0,657046793	-1,232319296	-1,160559991	-1,265119303
A_44_P367465	Fzd9	8,11E-13	0,69480948	1,143323645	1,237887871	1,058899669
A_44_P368249	Spag8	2,66E-12	0,635205188	-1,143509103	1,641598704	1,171384127
A_44_P369997	Trpm4	1,63E-14	0,717650006	1,142721161	1,244702353	1,290019151
A_44_P370052	Ldhc	2,28E-11	0,711247321	1,018200035	-1,428450477	1,862283481
A_44_P374618	Cdkn1c	5,67E-13	0,717896613	-1,161673885	-1,16852107	-1,334258775
A_44_P374824	Klk1c3	3,70E-13	0,703756198	1,304208177	-1,068638352	1,939388521
A_44_P375185	Slc39a14	1,60E-13	0,692860363	1,066108255	-1,073541182	-1,219577661
A_44_P375658	Rbm11	4,83E-19	0,835327096	-1,429862381	-1,226259106	-1,135672376
A_44_P378742	Flrt3	2,16E-11	0,711917398	-1,482659414	-1,092695763	-1,131519745
A_44_P379891	Pspc1	7,26E-13	0,715175472	-1,112961502	-1,118846668	-1,125514802
A_44_P381917	Sphk1	2,65E-13	0,759329845	1,077005062	1,237120962	-1,045451499
A_44_P384090	Des	9,65E-25	0,908487136	1,192787431	1,290849727	-1,261158236
A_44_P388959	Lrp3	2,30E-16	0,775790499	-1,010535518	1,070046973	1,179860252
A_44_P389178	Cracr2b	2,12E-15	0,772731607	1,364265817	1,142483997	1,304444624
A_44_P393343	St3gal6	1,85E-11	0,656276789	-1,268260865	-1,090135559	1,193490539
A_44_P399414	Klk1	1,25E-12	0,689786914	-1,041709095	1,124073475	1,913304029
A_44_P400324	Maf	6,04E-10	0,62955023	-1,428527441	-1,169451665	-1,227610712
A_44_P403755	Dusp12	1,73E-12	0,685949379	-1,143698848	-1,137546378	-1,288568105
A_44_P409709	Phldb1	1,76E-12	0,640609137	1,14115086	1,038906194	1,137909822
A_44_P409729	Opa1	3,99E-11	0,704555643	-1,035901101	-1,078024861	1,257156432
A_44_P409820	Faslg	3,85E-19	0,848772737	1,181436664	-1,292046605	1,201970385
A_44_P412236	Stag3	7,98E-12	0,620455067	-1,074690546	1,225501627	2,5226098
A_44_P414460	Rnd3	1,31E-12	0,668030342	-1,215169825	1,283094869	1,176383374
A_44_P419746	Dennd1c	1,43E-11	0,612384246	1,556571341	1,235419605	1,752926591
A_44_P420640	Nup43	1,68E-19	0,841844413	-1,039880127	-1,110144363	-1,096848713
A_44_P421295	Cyp11b3	9,66E-12	0,642537631	5,650246467	8,149869735	8,68400189
A_44_P424076	Dhdh	5,59E-17	0,787403314	-1,017934124	-1,208788399	-1,394974413
A_44_P425619	Pgm3	1,22E-11	0,614566734	-1,124586396	-1,203300603	-1,25269667
A_44_P428326	Hmgcs2	6,88E-10	0,605384196	1,123608046	1,398228836	-2,227465829
A_44_P429453	Lama5	4,17E-11	0,704018627	-1,173120105	1,022913115	1,134130472
A_44_P430547	Ntrk1	1,24E-27	0,941252005	1,441925069	1,036475274	1,478645589

A_44_P430581	Nsmce4a	8,21E-14	0,770501682	-1,104329786	-1,192430006	-1,096892947
A_44_P434271	Cntn2	1,54E-11	0,61135328	1,099778135	-1,101897368	-1,212507491
A_44_P436040	Rassf6	9,92E-18	0,840442574	1,276579787	-1,31663751	1,354346604
A_44_P440556	Muc13	2,47E-11	0,652512125	-1,141241298	1,133938196	1,644129001
A_44_P440944		4,56E-28	0,938035432	1,405464857	1,21621324	-1,144188857
A_44_P442780	Kcnab1	9,98E-15	0,789268909	-1,268725066	-1,434893259	-1,324907307
A_44_P448051	Vpreb3	3,70E-10	0,711084256	1,043024219	-1,361848435	1,143451092
A_44_P450307	Eps8	5,54E-16	0,784189896	-1,42654083	-1,277860057	-1,990037615
A_44_P450486	Ttll1	2,33E-11	0,711035543	1,036241744	-1,000054206	-1,379953181
A_44_P450518	Rangrf	1,47E-18	0,828137049	1,087664387	-1,130458664	1,335339109
A_44_P456172	Mterf2	9,01E-11	0,656228694	1,316291384	1,073911859	1,177057876
A_44_P457099	Plpp5	8,36E-12	0,686753337	-1,016786901	1,001260311	1,296569014
A_44_P458021	Tnfrsf11b	1,97E-10	0,645533227	-1,133717363	1,218243415	-1,453561245
A_44_P458901	Neurod2	7,85E-11	0,636836903	-1,113607081	-1,033306942	-1,497886993
A_44_P461383		7,45E-16	0,796498063	1,442895822	1,129856538	1,134982773
A_44_P463488	Amh	1,46E-19	0,829916594	1,111052708	1,286448372	1,383422264
A_44_P463749	Rasd1	9,48E-18	0,785346524	1,253498822	1,202139466	1,454273513
A_44_P463831	Zfp423	1,20E-17	0,799339751	-1,172005604	1,085186977	-1,198329753
A_44_P465953	Rgs8	2,14E-22	0,841717232	-1,172203557	-1,022449951	-1,261578781
A_44_P469584	Cd14	2,23E-12	0,637529702	-1,153467756	-1,149473745	-1,642132204
A_44_P472661	Klf2	3,68E-18	0,821999782	-1,398601599	1,04131922	-1,215166127
A_44_P474101	Slc7a1	4,63E-21	0,862130684	1,181965602	1,144151991	1,127532783
A_44_P475406	Hsf1	4,00E-16	0,771082503	1,060501628	1,089417426	1,359636592
A_44_P477406	Slc2a4	2,30E-15	0,755484697	1,065672729	1,184089966	1,355879652
A_44_P480623		6,53E-24	0,901435277	-1,048460456	-1,181568369	1,001571399
A_44_P480696	Ap1s2	1,12E-18	0,801536666	-1,035718799	-1,202539222	-1,458845903
A_44_P481892	RGD1309079	2,36E-13	0,727329462	-1,32393813	-1,17080934	-1,334616921
A_44_P481915	Id4	1,87E-13	0,762673314	-1,197859308	-1,086095963	1,343623906
A_44_P485388	Chd7	1,59E-14	0,736966676	-1,006504338	-1,01850934	-1,311433851
A_44_P491393	Nudt7	7,93E-10	0,603238402	-1,095430426	-1,16489916	-1,241116626
A_44_P496447	Ntf3	3,16E-24	0,89557226	1,086076142	1,245988005	-1,051189667
A_44_P500431	Lman1	4,50E-09	0,640632932	1,036943925	-1,004525258	1,16850235
A_44_P501761	Crybg2	4,81E-19	0,835342262	1,171586269	1,137057146	1,488985908
A_44_P506635	Ifrd2	4,35E-13	0,701927746	1,036344155	1,115508309	1,087009501
A_44_P508691	Tm6sf2	1,36E-28	0,92170148	1,269355947	1,186060556	1,194561698
A_44_P513385	Dab2	4,46E-08	0,62424575	-1,368235683	1,065139242	-1,396884439
A_44_P514987	Tshz1	3,77E-14	0,728278237	-1,106731456	-1,276871173	-1,531570779
A_44_P517033	Rab11fip5	5,63E-12	0,649609423	1,153284775	1,015228236	1,126238957
A_44_P521587	Arhgef3	1,97E-22	0,826855678	-1,011405634	-1,063143069	-1,387686945
A_44_P522047	LOC684871	3,96E-12	0,747354883	-1,092195413	1,375792862	1,544415266
A_44_P522396	Etfdh	9,66E-13	0,648288101	1,008012123	1,122784736	1,090299326
A_44_P522994		3,14E-11	0,626562489	-1,246262311	-1,082956982	1,38356538
A_44_P523028		3,76E-27	0,919156763	1,277255378	1,047570516	-1,80116831
A_44_P532249	Fkbp5	2,06E-12	0,783155091	1,4030669	1,046169347	1,197917405
A_44_P538521	Star	9,76E-15	0,741795502	1,106124586	-1,023912498	1,318157589
A_44_P538970	Ptpdc1	2,16E-13	0,709742604	1,027559549	-1,135511612	-1,303996023
A_44_P543917	Fbxo42	2,47E-11	0,673238871	1,000096103	-1,186164738	-1,258271806
A_44_P552533	Upb1	1,80E-23	0,855129944	1,297932899	-1,104183527	2,115055176
A_44_P558411	Scrg1	8,70E-10	0,601824044	-1,167151585	-1,189589329	-1,674658421
A_44_P579382		4,25E-12	0,746599062	1,283763484	1,170368276	1,176738717
A_44_P586876	Tmem41a	1,07E-10	0,632485039	1,027053798	1,097120556	1,192123019
A_44_P608318	Fam167a	5,47E-11	0,618773296	-1,465416119	-1,051150869	-1,166476211
A_44_P608485	Robo3	1,29E-20	0,856642841	1,268308231	-1,17778707	2,694102611
A_44_P612762	Lrrc8c	2,84E-12	0,679967328	-1,145709672	1,140504851	1,244973169
A_44_P613170	Ndp	2,34E-15	0,771887242	-1,256420267	-1,075219662	-1,309472934
A_44_P622113	Sept8	3,42E-13	0,704643869	-1,03221608	-1,165055102	-1,212449501
A_44_P628515	Rgs7bp	4,46E-14	0,77610683	-1,406927036	-1,132427398	-1,278121493
A_44_P638511	Paqr5	2,82E-26	0,927060066	-1,157987256	-1,77511848	-1,520739982
A_44_P639365	Col24a1	1,30E-10	0,723900863	1,19281039	1,10208067	1,73575084
A_44_P653193	Dtx1	3,53E-19	0,793559808	1,218144777	1,071306602	-1,155385
A_44_P668185	Endod1	5,03E-11	0,66400832	1,063975129	-1,086458424	1,220853288
A_44_P670825	Tmem182	5,06E-11	0,619887435	-1,349179939	-1,081769277	-1,285984078
A_44_P671089	Foxc2	2,03E-12	0,683990762	1,22836918	1,605923834	1,705997107
A_44_P684579	Zbtb39	4,91E-10	0,61045977	1,10748388	-1,127182986	-1,495876572
A_44_P694872	Fubp1	9,52E-11	0,610847536	-1,694319499	-1,129922818	1,086028558
A_44_P715030	Vstm2a	1,68E-11	0,6101296	1,512063408	2,501517387	3,00148567
A_44_P716637	Msx2	2,41E-19	0,826702301	-1,13975072	-1,144896648	1,003840334
A_44_P720576	Lrrn4cl	6,79E-10	0,605587991	1,456453584	1,186184455	1,794497819

A_44_P729856	Ppp1r3d	1,69E-14	0,811774028	1,027260889	-1,135912234	1,169240932
A_44_P745710	Olfml2a	1,20E-12	0,669102548	1,245270624	1,787843953	1,104594998
A_44_P764174	Fcmr	4,45E-23	0,893798951	-1,2219951	-1,493305004	1,078213367
A_44_P768777	Klf14	2,37E-19	0,812520175	-1,349392885	-1,220124351	-1,245453415
A_44_P777424	LOC100362783	2,89E-13	0,75845308	1,222450841	-1,096592985	1,002445511
A_44_P794669	Afdn	2,34E-14	0,733131714	1,087989055	1,077558824	1,3938659
A_44_P807585	Prcd	1,45E-30	0,933870119	1,007977643	-1,065320475	-1,135303324
A_44_P808710	Rd3	4,22E-12	0,675090931	-1,114455059	-1,248339052	-1,638489809
A_44_P809374	Clcf1	6,00E-14	0,723462738	1,096247186	1,092044079	1,330289895
A_44_P810179	Acd	7,04E-29	0,930486502	1,115222049	1,02872564	1,230793987
A_44_P810190	Srrt	4,87E-13	0,679948926	1,049726346	1,084082735	1,252223933
A_44_P819401	Slc40a1	8,16E-13	0,731564894	-1,668249579	1,156716115	-1,438350722
A_44_P822447	Ctla2a	3,25E-15	0,784336771	1,259426265	1,321210509	-1,818974654
A_44_P847319	Luzp2	4,17E-14	0,776710041	-1,423662051	-1,238681657	-1,419993248
A_44_P853916	Slc43a2	2,48E-26	0,90494715	-1,121344211	-1,293522366	-1,419054502
A_44_P876363	Vav2	9,13E-24	0,935240588	1,331400006	1,028838191	1,367784836
A_44_P879764	B4galt1	1,47E-10	0,604474192	1,043604232	1,181465566	1,18309887
A_44_P885889	Fam81a	6,92E-15	0,745126607	1,12478164	1,080784243	1,171518795
A_44_P898436	Lrrc17	2,68E-16	0,790137703	-1,328125438	-1,202399034	-1,436885536
A_44_P899938	Fam124a	1,53E-12	0,706827724	-1,124479445	-1,130586652	-1,274011015
A_44_P917327	Lrtm2	8,31E-32	0,946166942	1,256668241	1,015006038	1,085645371
A_44_P917564	Mat2b	1,32E-15	0,77687816	-1,176039717	-1,143155967	-1,251409256
A_44_P929530		5,58E-33	0,951401963	-1,022622065	-1,206692899	-1,807904912
A_44_P930534	Gas2	1,72E-16	0,778206333	1,415895528	-1,45386411	1,167380359
A_44_P945723	Zc3h12d	4,79E-15	0,781007713	1,783184399	-1,23877583	1,881664084
A_44_P951676	LOC497899	3,80E-21	0,863164468	1,318979306	1,07155768	1,084181729
A_44_P961602	Rasl12	1,29E-18	0,800506993	1,284985358	2,147100111	-1,179775374
A_44_P975327	B3gnt2	1,98E-11	0,655449337	-1,023662848	1,26864343	1,622349103
A_44_P988767	Crem	6,12E-12	0,648535038	1,065994977	1,377102848	2,207206943
A_44_P991472	Rnf39	3,05E-22	0,875724014	1,122837553	1,013426662	-1,303381389
A_44_P997843	Csrp3	5,34E-20	0,848630737	1,015689724	-1,042725027	-1,260938725
A_44_P999125	Masp2	1,44E-18	0,852322783	1,252992562	1,03876001	2,003514776
A_44_P999395	Lmbr1l	2,06E-09	0,688606121	-1,018435617	1,420775018	-1,022163757
A_64_P000041	Cfap58	6,07E-25	0,910121777	1,487418045	1,182542554	-1,647577695
A_64_P000386	Isl2	2,32E-09	0,609365796	1,485872205	1,616111055	1,597489778
A_64_P000645	Ttc30a1	4,69E-18	0,773481369	1,080367728	1,245205384	1,379726296
A_64_P000876	Kcna7	3,91E-16	0,753905225	1,181400264	-1,089226253	1,490585716
A_64_P001219	Mthfs	7,29E-10	0,647495278	1,207312251	1,065682769	1,196371692
A_64_P001799	Fam110d	5,67E-11	0,641323103	1,257048833	-1,168069567	1,730466989
A_64_P002339	Zbed4	8,75E-11	0,676383332	-1,060633605	-1,009165205	1,320244371
A_64_P002899		2,34E-13	0,744546856	1,053343925	1,03029923	1,576380819
A_64_P003694	Abcc12	1,21E-14	0,699647684	1,116212556	-1,030860582	1,872367593
A_64_P003749	Pax4	3,71E-13	0,722491728	-1,079724444	-1,241777766	1,66245674
A_64_P003972	Slc22a4	1,69E-10	0,602480725	1,393066231	1,029236717	1,616035623
A_64_P004307	Pipox	1,29E-12	0,689378333	-1,343771316	-1,179012963	-1,1567814
A_64_P004337	Dtl	1,11E-11	0,640713473	-1,615867127	-1,617148057	1,498925531
A_64_P004406		1,38E-14	0,755725398	-1,160811366	-1,096002531	-1,096291654
A_64_P005987	Agfg2	2,34E-13	0,727431096	1,208108396	1,113648033	1,092830937
A_64_P006525	LOC681364	3,25E-29	0,932498521	1,230461246	1,057684345	-1,226736589
A_64_P007178		4,26E-17	0,789570019	11,67234641	4,041062459	1,271866017
A_64_P007508	Csf3	7,82E-16	0,747555044	1,088368502	1,476494385	2,089160083
A_64_P007783	LOC103691261	6,80E-12	0,669146417	-1,404647616	-1,2266966	-1,583731975
A_64_P007822	Fezf1	4,80E-15	0,709430202	1,042125336	-1,221890277	1,88675775
A_64_P007904		5,06E-13	0,656380337	1,449920451	1,237461517	2,205776473
A_64_P008177	Tchh	1,05E-12	0,691838949	-1,792414248	-1,152768581	1,654485216
A_64_P008879	Thpo	4,70E-14	0,706444159	-1,092569827	-1,00698658	1,355362537
A_64_P009355	Reep4	9,78E-15	0,758926133	1,274794999	1,05557086	1,189307837
A_64_P009565	Vmo1	2,01E-24	0,913639105	1,18539531	-1,18178963	1,188954248
A_64_P009718	Btbd17	6,71E-36	0,969565118	1,168266206	-1,073482282	-1,398807852
A_64_P009803	Btg2	1,49E-24	0,945382706	-1,293524698	1,391388759	1,048390132
A_64_P009892	Lbx1	1,31E-13	0,733490937	1,324374364	1,017990134	1,46557815
A_64_P009999	Tac1	2,64E-11	0,651621671	1,099201149	-1,299599113	-1,27735638
A_64_P010139		2,75E-10	0,640858268	-2,066947722	-1,2501969	1,722777993
A_64_P010234	Gys1	3,35E-18	0,79338216	1,304683302	1,200375015	1,608808713
A_64_P010648	Ccl12	1,36E-16	0,722463108	1,041779064	-1,065718167	-2,425237895
A_64_P010960		4,51E-15	0,781524476	-1,023777981	1,084982551	1,287953154
A_64_P011045	Hrh3	2,89E-13	0,70652162	1,112112634	1,144090931	1,447130392
A_64_P011070	Asic4	5,95E-24	0,883415057	1,149956912	1,127823313	1,358671893

A_64_P011099	Rpl3l	5,86E-14	0,682055841	1,24069256	1,089123291	1,284579635
A_64_P011489	Gdf15	1,08E-09	0,621019743	1,52493795	3,411914395	-1,027504705
A_64_P011779	Sult1a1	9,01E-18	0,768110257	1,360355346	1,209346062	1,521242236
A_64_P012471	LOC363324	1,30E-27	0,922357832	1,360887133	1,010082464	-1,752190944
A_64_P012846	LOC108349386	4,57E-28	0,931987964	1,168779582	-1,164489469	-1,510415526
A_64_P012868	Tnxa-ps1	7,30E-11	0,659063682	-1,112731889	1,300066211	-1,115388669
A_64_P013753	LOC108348296	8,89E-29	0,929868614	1,061303559	-1,217203114	-1,645289433
A_64_P014178		4,09E-11	0,686125166	1,407429687	1,109859843	1,454689242
A_64_P015366	Mfsd4a	9,16E-18	0,785619778	-1,28591485	-1,181677427	1,431501617
A_64_P015370	Mfsd3	7,44E-12	0,621406509	1,314236468	-1,020070002	1,169816408
A_64_P015395	Spesp1	1,87E-10	0,600958978	1,511132494	1,016192893	1,802302607
A_64_P015460	Ccl4	9,75E-12	0,703632987	1,102401809	1,117993407	-2,291472036
A_64_P015479	Rfc5	6,77E-16	0,748895626	-1,062304647	-1,106767946	-1,174952342
A_64_P016578	Rassf7	1,11E-12	0,646484111	1,117492083	1,250604211	1,197812697
A_64_P016746	Lhx4	7,10E-15	0,726194473	-1,575194728	-1,320245908	1,010556721
A_64_P016766		5,58E-14	0,758674546	1,569752544	-2,052700677	1,138448555
A_64_P016828	Myo1b	3,33E-12	0,67801528	-1,022717042	1,033229845	1,262239687
A_64_P016951	LOC108348348	1,80E-24	0,897774414	1,770235255	1,141604974	-1,414514534
A_64_P017495		6,27E-11	0,639933386	1,180468954	1,368471197	1,068727465
A_64_P017706	LOC102547811	4,34E-31	0,942688137	1,092531997	-1,038788678	-1,328547767
A_64_P017758	Morn2	4,68E-17	0,788810201	-1,067970377	-1,078513202	-1,406941438
A_64_P018427		3,48E-11	0,668823578	-1,413942485	-1,292928867	-1,3946712
A_64_P018633		1,75E-16	0,807780768	3,896437675	3,785283224	3,657644421
A_64_P018766	Calca	8,02E-25	0,909142511	1,485064508	-1,008171048	1,215195136
A_64_P019016	LOC501396	3,20E-11	0,707231675	1,289059449	-1,013314114	1,911308824
A_64_P019200	RGD1560608	7,27E-11	0,63790576	-1,015831812	-1,471253472	2,444508429
A_64_P019608	Prkca	1,67E-11	0,697220967	-1,319067918	-1,093244416	-1,112386507
A_64_P020238		7,49E-10	0,647100765	-1,510605537	-1,501242907	1,091145544
A_64_P020248	Hist1h2bo	3,39E-16	0,755210738	1,112130818	-1,103356677	1,370916156
A_64_P021043	Pcsk9	8,06E-21	0,819727107	1,078621578	-1,236897518	-1,304506018
A_64_P021141	Rgs1	4,41E-14	0,707124502	1,078903147	1,265919986	-1,961943397
A_64_P021221		1,72E-29	0,940090103	1,164348521	-1,075343696	-1,581179768
A_64_P021428	Slc16a6	5,85E-14	0,723729836	1,134385793	1,330351243	1,621971736
A_64_P021702		8,04E-11	0,657763379	1,153043898	-1,033928539	1,04071557
A_64_P022015	Sstr2	2,56E-24	0,887029972	2,001577077	1,281074733	1,057524436
A_64_P022813	Exosc10	9,90E-16	0,74535753	1,301084343	1,200540863	1,265132803
A_64_P022907	LOC108349337	1,35E-28	0,935118543	1,785945926	1,122803199	-1,265731414
A_64_P022943	Rara	3,09E-25	0,904406059	-1,258974533	1,098436012	-1,398650159
A_64_P023179	Olr1388	1,16E-11	0,701619923	-1,434874512	-1,566725334	1,303300805
A_64_P023250	Ascl2	8,51E-19	0,803518275	-1,021463224	-1,00895151	-1,256165115
A_64_P024112		3,47E-11	0,647969625	-1,088933052	1,334013281	1,365242186
A_64_P024356	Sh2b2	2,65E-13	0,687097801	1,535027557	1,243331403	1,400679908
A_64_P024474	Stum	2,84E-18	0,83647863	1,026670445	-1,12316982	-1,4571295
A_64_P025050	Ccl1	3,87E-16	0,850541594	1,593260137	-1,229464655	1,452161893
A_64_P025098	Cebpg	2,05E-16	0,819834489	1,017878011	-1,113870472	-1,390673436
A_64_P025118	Cebpb	3,07E-14	0,819479878	-1,312504632	1,09941034	-1,022766818
A_64_P025173	Phlda2	4,64E-17	0,788881894	-1,787567153	-1,304407146	-1,427339077
A_64_P025248		1,83E-10	0,601296828	4,51523351	3,901045529	10,13356746
A_64_P025325	LOC501396	7,42E-18	0,80292841	1,124484548	-1,00190841	1,161008794
A_64_P025535		1,43E-33	0,958335831	1,264147819	-1,033801164	-1,313946833
A_64_P025599	Eif4g2	2,35E-09	0,60912454	1,064049861	-1,032234422	1,145877245
A_64_P025678	Tbc1d16	1,53E-18	0,851939912	1,267573894	1,062547539	-1,24083023
A_64_P025963	Ccnf	9,99E-11	0,674649903	1,063830077	1,162721304	-1,272140248
A_64_P026374	Flt3	3,85E-13	0,65976289	1,121845327	1,392722684	1,586393685
A_64_P026943	Gck	3,29E-13	0,684577489	-1,012257838	1,034271523	1,149166897
A_64_P027263	RGD1584023	4,96E-11	0,701900274	-2,059948186	-1,293079494	1,654825736
A_64_P027847	Fer	6,54E-11	0,732024483	-1,203673523	-1,004919764	-1,18749845
A_64_P027911		4,24E-18	0,821032908	1,276612483	-1,229849031	-1,582160764
A_64_P028290	Yrdc	4,30E-27	0,910958362	1,20263701	1,008480501	1,149531221
A_64_P029077		5,18E-11	0,701352264	1,413152674	1,712885778	2,13919085
A_64_P029282	Man1c1	1,13E-16	0,764914128	-1,239030327	-1,314520153	-1,128489113
A_64_P029337	F8a1	1,65E-08	0,620535661	1,135164506	1,04571924	-1,061332587
A_64_P029501	Tnnc1	2,40E-17	0,82220434	-1,204416408	-1,030498125	-1,120724163
A_64_P029878	Clcnkb	2,67E-12	0,635173704	1,275723232	1,068466274	1,463019359
A_64_P029887	Focad	5,14E-14	0,742969187	-1,918787711	-1,146779102	-1,052455183
A_64_P030464	Tnxb	9,12E-15	0,79003426	-1,082831521	1,330213382	-1,002374279
A_64_P030534	Tpcn2	3,46E-27	0,926439896	1,203376476	1,183827295	1,173199206
A_64_P031046	Draxin	1,88E-15	0,757353941	-1,209090878	-1,008507414	-1,558240265

A_64_P031058	Trpm6	5,90E-17	0,770434509	-1,378398599	-1,020386219	1,576898061
A_64_P032148		4,14E-18	0,791770714	1,475616484	1,256166587	-1,029510754
A_64_P032358	Mustn1	3,28E-15	0,811870887	1,024820287	1,079580732	-1,093798918
A_64_P032904	Mbnl2	9,58E-18	0,801030513	-1,250703111	-1,067167037	-1,229473611
A_64_P033559	LOC679711	1,34E-26	0,915158253	1,109165476	1,277455603	-1,347769142
A_64_P033569	Tep1	2,24E-09	0,609879611	1,203571282	1,057625004	1,274433107
A_64_P033795	Hgd	1,73E-20	0,866050272	1,132142611	-1,779156512	1,359093584
A_64_P033901	Pars2	3,98E-14	0,727714416	1,009294383	-1,044309077	-1,207296281
A_64_P034090	Adm2	1,05E-10	0,654216305	1,067543361	1,188783774	2,086402351
A_64_P034155	Ccdc166	2,48E-29	0,94982725	1,560254638	1,012910286	1,123688634
A_64_P034466	Nkain1	8,92E-24	0,858738976	1,143860647	-1,049554621	-1,115858706
A_64_P034684	Rpain	1,05E-09	0,621332606	1,169747385	1,016264945	-1,124016813
A_64_P035192	Kif26a	3,70E-14	0,709015652	-1,013871329	-1,082257614	-1,380973887
A_64_P035238		3,90E-29	0,93816837	1,038694523	-1,089043556	-1,406012967
A_64_P035663		4,35E-27	0,918710049	1,296739906	1,112970513	1,184227696
A_64_P036083	LOC367516	6,36E-31	0,941852305	-1,001222509	-1,122629532	-1,448033822
A_64_P036576	RGD1563667	1,39E-14	0,738307693	1,557693415	1,27819501	1,907996203
A_64_P036676	Klf6	1,27E-10	0,671536587	-1,694555255	1,066084207	1,032729663
A_64_P036770		1,13E-12	0,66980167	-1,649488649	-1,26460991	-1,297731292
A_64_P036900	Kcnk3	3,53E-14	0,709519153	-1,178937388	-1,081467193	-1,215264022
A_64_P037068		2,94E-11	0,627487162	-1,135615855	1,441706087	1,157463508
A_64_P037259	Ankar	1,85E-16	0,807345913	2,044458077	1,361464011	1,494498656
A_64_P037626	Bricd5	6,49E-13	0,676553598	-1,401799962	-1,202397838	1,117370619
A_64_P037758	Stk24	3,57E-11	0,647591376	1,002936579	-1,145102804	-1,255724153
A_64_P038625		1,89E-14	0,768810676	1,082826339	1,014278731	1,284829569
A_64_P038767	Aste1	5,98E-14	0,741444707	1,143158513	1,134645733	1,182562603
A_64_P038839		1,31E-11	0,717790094	1,627569026	-1,364551268	2,153526386
A_64_P038926	Prodh1	3,28E-09	0,603956249	1,255248917	1,04767517	1,213418767
A_64_P039165	Gcat	4,12E-12	0,629404963	1,054414767	1,02815841	1,377672269
A_64_P039432	Clk1	2,34E-10	0,621364981	-1,804539759	1,038999594	-1,061950158
A_64_P039737	Scx	6,02E-27	0,917697816	1,279189374	1,107456974	-1,144521846
A_64_P039809		1,11E-28	0,935609318	1,277953497	1,129657228	-1,449786038
A_64_P040095		1,07E-13	0,735517546	1,151153379	1,46284518	1,779880213
A_64_P041104	Dpf1	2,70E-16	0,737999302	-1,372051705	-1,100612859	-1,12192596
A_64_P041219	Pcsk1	1,17E-20	0,832064465	-1,063493636	1,061407928	1,288309264
A_64_P041498	Pla2g4f	9,96E-20	0,818380465	1,284374538	1,261362747	1,192192795
A_64_P042249	Acvr1c	8,24E-19	0,844208698	-1,371035123	-1,211931355	-1,41437404
A_64_P042885	Fn3k	1,83E-10	0,62491671	-1,040718058	-1,080069598	-1,194431841
A_64_P042930	Hcfc2	1,26E-13	0,695569389	1,113415338	1,032336777	1,040752115
A_64_P043033	Cbr3	9,66E-11	0,693596233	1,263782795	1,173135429	-1,165129036
A_64_P043361	RGD1307443	7,01E-17	0,800690543	-1,493095361	-1,05742324	-1,421545819
A_64_P043561	LOC494538	1,35E-11	0,660441803	-1,119777751	1,198138867	2,167438142
A_64_P043614	Slc39a5	2,85E-19	0,850548775	1,88096609	1,467856749	2,704131668
A_64_P043731	Vps37b	2,47E-10	0,620543465	1,254621464	1,065109494	1,004166492
A_64_P043776	Fat4	1,63E-16	0,821469821	-1,066288594	1,028762785	1,219478286
A_64_P044440	Fam43a	7,27E-22	0,848319439	-1,142294455	-1,070163691	1,5071993
A_64_P044470	Mrpl50	3,42E-12	0,677675589	-1,100757543	-1,652539447	-1,113983319
A_64_P045062	Otog	2,00E-13	0,667678697	1,301490788	1,105834528	1,564692575
A_64_P045122	Mesp1	1,14E-27	0,915240915	1,556401095	1,196854702	1,122849523
A_64_P046082	RGD1560585	3,25E-11	0,626073486	-1,003623078	-1,127874566	1,299779176
A_64_P046120		1,25E-12	0,668606284	-1,002961272	-1,322815523	-2,01975078
A_64_P046885	LOC363337	2,06E-22	0,877558882	-1,014435969	-1,286777826	-1,38601205
A_64_P047201	Tmem181	1,55E-16	0,762144722	-1,068879878	-1,21559224	-1,185988442
A_64_P047730	Eif1	5,38E-18	0,819392156	-1,004595477	1,008579669	1,250575709
A_64_P048004	Bfsp2	2,81E-07	0,631321851	-1,163985091	1,335559037	-1,399330156
A_64_P048033	Agpat3	5,85E-14	0,704048984	-1,094230311	-1,034687888	-1,408960337
A_64_P048055	Alkal2	1,68E-19	0,841842333	1,114757244	-1,096510621	-1,268866686
A_64_P048235	Tmem42	3,66E-14	0,687410429	1,121976031	1,069579523	-1,009310313
A_64_P048705	LOC100363225	3,35E-24	0,903957109	1,197287548	-1,078355831	-1,176669617
A_64_P048712		1,45E-11	0,612195346	1,305993494	1,174065503	1,633481913
A_64_P048765	Cbs	3,76E-11	0,738367648	-1,370329523	1,048313121	-1,483555373
A_64_P048852		5,35E-10	0,651864183	1,188804894	1,008787203	1,12089946
A_64_P049161		2,31E-24	0,877095005	1,54222918	1,27830624	1,5863435
A_64_P050052	Pde9a	1,75E-26	0,921675356	1,107206271	1,127143589	1,167466902
A_64_P050171	Sema4c	4,86E-17	0,80346452	1,250341859	1,252706924	1,138585877
A_64_P051104	Lmx1a	2,03E-10	0,623436972	-1,107362546	-1,08329367	-1,326313064
A_64_P051139		1,72E-16	0,778251143	1,255742414	1,12207255	1,48688297
A_64_P051507		1,44E-32	0,954449804	1,089802054	-1,110473728	-1,269250387

A_64_P051967	LOC103690296	1,33E-11	0,638240719	1,542021339	-1,46329009	1,674273828
A_64_P053031	LOC501467	1,92E-12	0,639466899	2,321216276	2,580234253	1,238400964
A_64_P053084	Grifin	8,37E-27	0,916660572	1,054376952	1,003083689	-1,345299228
A_64_P053353	Jun	4,21E-16	0,753233012	-1,292698975	-1,233167797	-1,373757655
A_64_P053461	Bag2	3,63E-22	0,884759581	1,257069194	1,163497572	1,269918842
A_64_P053785	Adra2a	8,59E-14	0,719683358	1,011887786	-1,25719395	1,488161602
A_64_P053861	Ppp4r4	7,77E-21	0,834582447	1,229831351	-1,146162655	-1,258716864
A_64_P054153	Nudt6	6,76E-25	0,909741906	1,077938552	1,004592486	-1,40624318
A_64_P054461	Bhlha9	3,80E-12	0,654698106	1,873891276	-1,040325916	1,547541201
A_64_P054626	Hcn4	6,60E-11	0,616102619	-1,21519335	-1,051367074	-1,519228052
A_64_P054765	Eml1	2,93E-17	0,792491885	1,124371009	-1,074438815	-1,347232363
A_64_P055135		5,12E-18	0,819737331	1,017969692	1,031226749	1,155494225
A_64_P055433	Tmem131	2,04E-10	0,665189688	-1,97245536	-1,223007423	-1,185434869
A_64_P055699	Timm9	2,32E-21	0,876129468	1,08002301	-1,121571338	1,225874135
A_64_P055763	Rhbdl1	6,96E-14	0,721907806	1,126716925	-1,011763711	1,378049986
A_64_P057587	Kcnn1	4,02E-15	0,731872176	1,295323633	1,533952074	1,514431369
A_64_P057877	Aatk	1,61E-14	0,696521698	-1,114291166	-1,034383825	-1,225780721
A_64_P058015	Gimap1	3,22E-16	0,788647615	-1,14450402	1,306859558	1,521715334
A_64_P058087	Rbp4	1,92E-18	0,861402225	1,338527426	-1,009624944	1,06207283
A_64_P058336	Ucn	3,41E-22	0,875183724	1,435479184	1,161058279	-1,343191625
A_64_P058878	LOC102554532	1,81E-12	0,664022576	1,531541836	1,199065376	1,305792844
A_64_P058988		4,30E-17	0,77309383	2,159521216	1,440879571	1,221059329
A_64_P059495	Ddit3	1,66E-19	0,874595564	1,181857592	1,101343925	-1,210362051
A_64_P060302	LOC365085	1,23E-08	0,644610112	1,126785729	1,173513149	1,314848328
A_64_P060608		1,90E-24	0,888270089	1,322519688	-1,15061788	-2,407180792
A_64_P060804	Dcbld1	2,33E-13	0,688557791	1,065922575	1,049506926	1,230809931
A_64_P061291	Micall1	1,59E-12	0,724349046	-1,132040835	-1,033346731	-1,162691322
A_64_P061735	Fam78b	4,19E-15	0,731469042	1,080122334	-1,008633425	-1,272945489
A_64_P061740	Trpv1	3,52E-15	0,797959193	1,250677476	-1,827741738	1,380125344
A_64_P061765	Fam89a	7,92E-10	0,625570122	2,100126397	3,164686389	3,7090714
A_64_P062223	Terb1	8,71E-22	0,880769653	1,061088295	1,09643352	1,412069894
A_64_P062285	Vpreb2	4,72E-10	0,633133088	1,101041746	1,183542473	2,043221201
A_64_P062388		8,78E-14	0,719459026	1,301114018	-1,061485891	-1,295422879
A_64_P062506		6,86E-28	0,930909909	1,380021334	1,015387799	-1,21281441
A_64_P062593	Chst5	1,26E-20	0,867689371	-1,118899997	1,214473104	-1,049233518
A_64_P062618	Chst1	3,14E-15	0,713832727	-1,015851204	-1,185955502	-1,203477475
A_64_P062899	Bcar1	5,90E-11	0,61770426	1,011695161	1,115434934	1,071569631
A_64_P063047	Rpp25	5,39E-10	0,722614254	1,378030542	1,015139482	1,321203531
A_64_P063398	Ptger3	4,08E-17	0,789910346	-1,720133921	-1,230540465	1,755690326
A_64_P063644		1,09E-12	0,710649678	1,203511813	1,107891358	1,198140944
A_64_P063953	Xkr7	1,28E-13	0,673030475	-1,137287172	1,20189303	1,73130304
A_64_P063962	LOC108350705	3,76E-12	0,654818613	1,141892058	1,244231392	1,139172086
A_64_P064321	Scrn3	2,39E-17	0,794088871	-1,218127292	-1,308379146	-1,207911871
A_64_P064371	Spata2L	9,54E-17	0,783086503	-1,290470602	-1,006905954	-1,179278641
A_64_P065032	P3h4	6,25E-10	0,669002395	-1,002024817	-1,195473533	-1,156992105
A_64_P065629	LOC102552540	7,93E-29	0,930172759	-1,091281858	-1,131285653	-1,26505087
A_64_P065939	B3galt2	4,03E-13	0,682192739	-1,137539608	-1,266605032	-1,115345444
A_64_P066181		6,38E-13	0,653514405	-1,122434661	-1,13461329	-1,135734109
A_64_P066577		6,13E-27	0,917641431	1,442592322	1,132592381	-1,161631113
A_64_P066586		3,37E-16	0,755264585	1,086992522	-1,025847045	1,134285466
A_64_P066621	Msc	4,25E-20	0,861253575	-1,092540211	1,396776859	1,832917498
A_64_P066782	LOC108348347	1,80E-25	0,928167651	1,753416817	1,094987447	-1,46852365
A_64_P067447	Acot4	4,05E-22	0,851535284	1,522867793	1,104150752	1,15891632
A_64_P067654	Aldh3a1	9,85E-13	0,711780211	1,037747682	-1,012877232	-1,341794142
A_64_P067659	Aldh1a1	4,39E-11	0,644807753	-1,020453335	1,137917433	-1,190438652
A_64_P067868	B3gnt9	1,43E-10	0,604966247	1,457075789	1,153659157	1,393741977
A_64_P067884	Trim47	8,68E-27	0,9085981	1,321477684	1,127267666	-1,00805559
A_64_P068027	LOC363337	1,18E-14	0,757193507	2,222399531	1,088332782	1,741925834
A_64_P068152	Smad6	2,75E-22	0,886008758	-1,071975775	-1,03061061	1,389460518
A_64_P068333	Zc3h12a	6,80E-20	0,805399559	-1,2288505	1,424889764	1,58848911
A_64_P068594	Car6	4,03E-14	0,708092803	-1,237854537	-1,04612016	-1,34442014
A_64_P068965	Sowaha	7,49E-13	0,674821415	-1,021991304	1,038097683	-1,422494525
A_64_P069071	Lpar6	1,52E-08	0,601093345	1,202152561	1,321040991	-1,081641732
A_64_P069374	Scn10a	3,24E-28	0,938869208	1,345012052	-1,321317169	1,105584044
A_64_P069739	Ankrd23	3,02E-19	0,810836666	1,31076137	1,093148478	1,214074834
A_64_P069819	Rrp8	2,14E-11	0,654370151	-1,001340075	1,018685249	1,196798329
A_64_P069942	Prss56	2,67E-20	0,852578277	2,239590491	1,640387414	1,363411824
A_64_P070068	LOC501416	9,37E-23	0,870783748	1,38554287	1,120948735	1,026486391

A_64_P070937	LOC501346	3,43E-15	0,798174992	1,39603978	2,136257238	1,506854585
A_64_P071810	Gpr143	1,31E-08	0,603506634	-1,40338588	1,214185227	1,324129817
A_64_P072088	Lrrtm2	1,26E-11	0,639021619	-1,201495552	-1,264334489	-1,21953763
A_64_P072227	Arrdc2	3,16E-12	0,749714612	1,756627154	1,257136617	-1,183927715
A_64_P072343	LOC501317	5,37E-27	0,918056731	1,254708135	-1,071916072	-1,327932762
A_64_P073845	Cysrt1	1,36E-21	0,857223117	1,365852042	1,120477672	-1,024787533
A_64_P073944	Sox11	1,15E-09	0,620014492	-1,003038262	-1,297668132	-1,30940016
A_64_P074102	Wnt2b	2,44E-13	0,70840093	-1,734420802	-1,114858112	1,316225794
A_64_P074127	LOC685203	5,51E-28	0,924840347	1,086370982	-1,087425351	1,39214984
A_64_P074490	Wipi1	5,35E-18	0,819422822	-1,125838591	-1,141679404	-1,164623512
A_64_P074851	Atp4a	4,41E-15	0,710314455	1,109848758	1,051325291	-1,444367637
A_64_P075087	Camk1g	5,37E-15	0,807989105	-1,318465764	1,101532771	-1,126694902
A_64_P075124		4,48E-11	0,621594328	1,097931491	-1,031965782	1,23455387
A_64_P075357	Cyr61	2,92E-23	0,886349261	1,108357234	2,2324417	2,693516495
A_64_P075910	Ajap1	1,83E-14	0,735614874	1,448200135	1,049150044	1,170427961
A_64_P076097	Obscn	4,64E-24	0,884490936	1,331077691	1,132106961	1,306687631
A_64_P076436		1,63E-26	0,914533957	1,745704402	1,035091766	-1,090375688
A_64_P076555	Jph1	5,58E-15	0,779678788	-1,58186309	-1,299984134	1,005954951
A_64_P076832	RGD1311447	9,47E-24	0,929122166	1,329271527	-1,126896627	1,136239214
A_64_P076997	RGD1561442	5,95E-11	0,617590205	-1,162439055	1,162709965	2,049777989
A_64_P077392		4,74E-14	0,789882183	-1,493491321	-1,245161746	-1,347624208
A_64_P079220	Gprin3	1,45E-22	0,868663831	-1,353327533	-1,005006373	-1,367294465
A_64_P079260	Ttc36	2,89E-13	0,686087039	3,959877031	4,448428068	4,935704914
A_64_P079553	Myh8	8,46E-11	0,695269386	1,122116129	1,060958628	1,179072107
A_64_P080580		9,46E-22	0,880383957	1,441154731	1,215768127	-1,177117551
A_64_P081222	Ptrh2	8,25E-19	0,818509412	1,047550259	-1,129561555	-1,049812056
A_64_P081738	LOC108349423	1,67E-26	0,914440791	-1,024270778	-1,109822252	-1,367346137
A_64_P081762	Trnau1ap	1,02E-12	0,711393897	-1,001799138	-1,145293289	1,17824505
A_64_P081822	Ticrr	4,62E-10	0,65392158	-1,19266285	1,027509626	1,247361296
A_64_P081977	Ppcdc	4,52E-10	0,611699692	1,244151817	1,13199611	1,183198001
A_64_P082356		2,66E-11	0,628830171	1,559887192	1,256916403	1,609344717
A_64_P082386	LOC102553270	1,70E-09	0,654965418	1,160777122	1,292932388	1,719439245
A_64_P083034	Asmt	1,80E-31	0,938799441	1,377000659	1,111754397	1,870784784
A_64_P083625	Klhl3	1,60E-11	0,635781415	1,166510234	-1,205807211	-1,151484347
A_64_P083978	Zfp68	4,01E-20	0,871983576	-1,318310631	-1,200405363	-1,277057514
A_64_P084059	LOC685183	8,02E-30	0,941839758	1,194434606	-1,027960896	-1,383383832
A_64_P084458	Egfl8	4,80E-12	0,627363151	1,087539654	1,217868512	1,276441067
A_64_P085530	Scd	8,23E-11	0,657453423	-1,370521566	-1,150889825	1,304676402
A_64_P085650	Slc22a14	1,69E-23	0,878775338	1,613413933	1,019832847	1,554777786
A_64_P085671		7,44E-20	0,868791872	1,020511349	-1,202770276	-1,632457237
A_64_P086398		9,62E-12	0,664759813	1,078900753	-1,016738667	-1,018711855
A_64_P086404	Polr2h	3,75E-09	0,601873132	-1,145790423	-1,078277784	-1,00509158
A_64_P086550		1,72E-11	0,634831095	-1,309684907	-1,316761008	-1,666948958
A_64_P086784	Runx1t1	9,06E-15	0,759637827	-1,246326672	-1,32493884	-1,12965844
A_64_P087039		1,11E-22	0,880412924	1,222787348	-1,005934403	-1,261761031
A_64_P087040		1,09E-29	0,935241312	1,293709743	1,054915423	-1,162105945
A_64_P087238	Harbi1	4,01E-13	0,682246231	-1,025839249	-1,216541197	-1,298603407
A_64_P087424	F2rl1	2,57E-11	0,651999303	-1,058667309	-1,121791734	1,556012737
A_64_P087801	St14	1,09E-14	0,740759993	1,156445855	-1,045624195	-1,443007602
A_64_P087867	Sys1	2,80E-10	0,61873004	-1,175201006	-1,097420669	-1,21662282
A_64_P088382	Angptl4	6,78E-16	0,823697943	1,15717901	1,117988137	-1,978234879
A_64_P088392	Hbb-b1	7,98E-09	0,734603203	-1,781012053	1,259478396	-1,585326775
A_64_P088393	Hbb-b1	5,21E-09	0,638425709	-1,751195266	1,277944099	-1,960272026
A_64_P088397	Sytl1	1,77E-22	0,912687538	1,149487613	1,154265119	1,207905483
A_64_P088695	Tmtc4	2,48E-11	0,692356573	-1,672484627	-1,046794007	-1,215749923
A_64_P088817	Carns1	9,34E-12	0,642977244	1,332590875	1,068807813	1,295866078
A_64_P088995	LOC685668	2,13E-19	0,840413303	1,062527264	1,055352832	1,181704593
A_64_P089016	LOC108349297	1,11E-25	0,908064093	1,034438698	-1,166660801	-1,368748136
A_64_P089040	Ovol1	2,72E-20	0,883493769	-1,568227757	-1,27162434	1,465414575
A_64_P089201	Tcp11	1,70E-13	0,712326301	3,221387539	3,730549367	3,140606189
A_64_P089457	Pcca	2,01E-20	0,828713221	-1,013264475	1,032403659	1,168497227
A_64_P089637	Rnf223	2,37E-19	0,796487	1,373025101	1,095521312	1,416720145
A_64_P090304	Ccdc88b	5,14E-26	0,931722153	1,153818932	-1,054953168	1,111470918
A_64_P090354	Syne3	9,22E-12	0,665305814	-1,638403327	-1,173041497	1,091670022
A_64_P092122	Zmynd8	9,55E-09	0,6086459	-1,249306248	1,004682805	-1,457098301
A_64_P092534	Sspo	1,11E-24	0,915643325	1,325164591	1,198115788	1,509847354
A_64_P093044		5,29E-18	0,844407699	1,14999359	1,104629405	1,373816865
A_64_P093522	Dtna	2,59E-12	0,700745232	1,003125958	-1,081226113	-1,191338987

A_64_P094249	Card14	9,38E-15	0,742176208	-1,005966484	1,690101921	1,013869424
A_64_P094515	LOC100360619	1,87E-17	0,823946711	1,14667239	1,079579749	1,227007222
A_64_P095005	Mmd2	2,72E-13	0,774060842	-1,172203474	-1,048023667	-1,350526198
A_64_P095586	LOC498316	1,23E-17	0,813574721	1,074033974	1,179098312	-1,543192567
A_64_P096006	Xrra1	1,30E-09	0,618170926	-1,67319204	1,641433861	1,395772101
A_64_P096748		1,16E-18	0,829713385	2,155118353	1,240029042	-1,297768991
A_64_P096787	Plpp4	4,49E-15	0,781557368	-1,086910162	-1,125873567	-1,215923952
A_64_P097598	Mtus1	3,60E-12	0,696882745	-1,103449026	-1,068508025	-1,269735886
A_64_P097857	Fam196a	4,24E-30	0,931176629	-1,00634276	1,000289823	-1,333984312
A_64_P098006	Fitm1	1,95E-20	0,854334418	1,112563175	-1,001353398	1,277356707
A_64_P098698	Nkpd1	9,21E-20	0,857001306	1,61686196	1,107620238	1,122465575
A_64_P099307	Tcf7l2	2,70E-14	0,731699287	1,157714782	1,303564763	1,350539906
A_64_P099633	Lcn10	7,95E-24	0,871410702	1,498658112	1,338509479	1,348586256
A_64_P099923	Dap	7,04E-12	0,724857117	-1,072772994	1,037404318	-1,225921431
A_64_P099983		2,17E-13	0,709674463	-1,062887371	1,032076643	1,601099038
A_64_P100318	Marc1	6,91E-14	0,721989896	1,177456498	-1,636540165	1,430120389
A_64_P100346		2,15E-32	0,953736642	1,168651255	1,143547561	1,232259668
A_64_P100447	Hoxd9	1,30E-11	0,700203942	1,722666464	1,357142094	2,265098518
A_64_P100516	Aoc3	1,89E-15	0,828412426	1,283597611	-1,197713211	1,658870481
A_64_P100544	Itsn2	6,54E-11	0,660523398	1,162353494	1,023083867	1,264006918
A_64_P100788	Ing3	8,38E-11	0,612681925	1,1238477	1,192254138	1,082614578
A_64_P100853	LOC689600	3,18E-14	0,688971986	1,414891583	1,268837601	1,433730535
A_64_P101204	Slc40a1	1,19E-14	0,720914696	-1,451416685	1,011112584	-1,613094614
A_64_P101336	Rfx5	1,31E-10	0,629623715	3,886999898	6,515737576	8,383360388
A_64_P101553	Sec63	1,15E-12	0,710069849	1,085796632	-1,009429917	1,19396579
A_64_P101908	Hmcn2	1,83E-15	0,789154827	1,224729132	1,265132904	1,964051623
A_64_P102141		3,68E-13	0,72260109	-1,483832927	-1,199450853	-1,486576699
A_64_P102801	LOC108348140	1,90E-10	0,600690781	3,551156644	13,71939921	8,041992218
A_64_P102935	Cd27	1,73E-15	0,719912585	1,344264096	1,458873104	1,644886228
A_64_P104590	Cfap157	4,69E-14	0,706453589	1,116177508	1,067676563	1,29988978
A_64_P104810	Podnl1	1,47E-08	0,601738529	-1,450205821	1,244082423	1,208861732
A_64_P106490		1,53E-28	0,934812335	1,26653992	1,04926986	-1,162363334
A_64_P106893		7,40E-14	0,679378117	1,227213952	1,09074853	1,610022556
A_64_P108009	Nrarp	9,58E-24	0,891070792	-1,185353378	-1,150318906	1,408170154
A_64_P108459		2,00E-22	0,904583474	1,821968858	-1,248389908	-1,372037583
A_64_P110469	Fdxr	1,93E-12	0,704143658	1,26841272	1,059856351	1,098242053
A_64_P110544	Spdef	6,20E-14	0,801090847	1,286295402	-1,214258369	-1,257699733
A_64_P110599	Plekhf1	1,52E-23	0,906428279	-1,239964167	-1,019291813	-1,193906883
A_64_P110898		2,67E-11	0,672212587	2,448557762	1,596377577	1,047381843
A_64_P112181	RGD1562660	8,83E-32	0,946043771	1,084076825	-1,119501038	-1,343245682
A_64_P112185		4,59E-18	0,833368359	1,04210891	-1,38429473	1,100672908
A_64_P113695	Myo7a	5,19E-20	0,822668027	-1,053784	-1,126422674	-1,451244641
A_64_P114338	RGD1563263	2,77E-15	0,753780544	1,366840148	1,069475846	2,293992032
A_64_P114396		7,09E-24	0,909206562	3,366786524	-1,022710878	-1,49832314
A_64_P114838		4,96E-30	0,942910665	1,12914461	-1,070217368	-1,577516177
A_64_P114900	Prelid2	1,37E-15	0,699032904	1,298334838	1,357442445	1,024523275
A_64_P114960	Kcnk4	3,48E-12	0,655810165	-1,03999897	-1,284484183	2,223384066
A_64_P116171	Ak8	7,14E-18	0,842528857	1,050787519	1,468510031	-1,087682358
A_64_P116298		2,68E-33	0,952736211	1,147481828	-1,048157211	-1,469586216
A_64_P116301	Adam33	1,18E-09	0,640548093	1,126118259	1,092832023	3,012346922
A_64_P116501	Kcnf1	2,22E-12	0,637599384	-1,119852599	-1,259733605	-1,60361408
A_64_P116600	Rcan1	1,94E-22	0,896461393	-1,046661517	1,222960746	1,16469377
A_64_P116606	Rcan1	1,44E-10	0,628291338	-1,098937601	1,272984612	1,59052406
A_64_P117726	Slu7	2,40E-08	0,614446395	1,026360687	1,002326578	-1,180670001
A_64_P118427		8,16E-13	0,69474442	-1,068639315	1,023410642	1,557409757
A_64_P118602	Rtel1	2,32E-10	0,621455218	1,153764213	1,056406073	1,466902395
A_64_P119314	LOC689065	2,47E-15	0,736636994	1,271991831	1,077283907	2,254742575
A_64_P119777	Pxdn	1,24E-10	0,607005122	1,06029607	1,047037788	-1,300699414
A_64_P119821	RGD1311084	1,97E-24	0,927518576	1,153156435	1,049845794	1,155938569
A_64_P120036		2,02E-12	0,684042969	1,051610004	1,192027972	1,605350924
A_64_P120445	Cacng6	8,37E-21	0,869776082	1,045653578	1,230446201	1,442291494
A_64_P120882	Hils1	4,50E-15	0,730766368	1,13364037	-1,581200076	-1,231463357
A_64_P120931	Asb3	6,87E-12	0,647034051	1,069572101	1,105553405	1,136713871
A_64_P121051	Myo15b	1,14E-18	0,78468393	1,536361723	1,476863848	1,522485513
A_64_P121136		3,02E-23	0,876128317	-1,985927842	-1,398482015	-1,252650286
A_64_P121796	Perm1	2,01E-34	0,952461101	1,246588905	1,007327538	1,247045842
A_64_P122247	Hcrtr1	2,60E-17	0,793421552	2,548636865	1,264341688	1,003064584
A_64_P122790	Acss1	5,72E-15	0,746949636	-1,067909233	1,288499217	1,401835515

A_64_P122975		1,14E-13	0,716682191	1,31270358	1,018267331	1,18756351
A_64_P123118	Fosl1	6,11E-20	0,847840396	1,322653783	1,139090735	1,457414521
A_64_P123949	Gk	2,03E-25	0,905927222	1,043861493	-1,049015682	1,171249766
A_64_P124090	Ntsr1	2,03E-29	0,954842786	1,307182684	-1,106252553	1,185654114
A_64_P124126	LOC100910970	2,16E-15	0,717652832	1,2631685	1,163633995	1,028996582
A_64_P124210	Greb1l	1,47E-15	0,74161451	-1,190153868	-1,131062825	-1,333330349
A_64_P124898	LOC363337	3,79E-18	0,821807074	2,22649012	1,386408668	1,355164982
A_64_P125611	Tbx6	4,75E-20	0,849302168	1,223395649	1,132565988	1,592385412
A_64_P125715	Serinc4	7,89E-10	0,603312399	1,740076755	-1,143975686	1,981961585
A_64_P125978	Atp2a1	4,28E-16	0,753098095	1,32726401	1,205387517	1,202879503
A_64_P126030	Slc22a7	8,44E-14	0,784795012	-1,070300056	-1,570369524	1,776920419
A_64_P126265	Rbm20	3,30E-12	0,632360714	1,175299473	1,046873425	1,911405626
A_64_P126818	Tspan18	3,91E-21	0,838683327	-1,020835716	1,019145111	-1,589475082
A_64_P127327	Sys1	2,71E-14	0,749232571	-1,267650363	-1,22063773	-1,10417806
A_64_P127332		9,10E-13	0,693480629	-1,15335329	-1,074397446	1,314394182
A_64_P127833	Ppfibp1	4,23E-11	0,666281799	1,006143463	1,125174194	1,110416015
A_64_P128725	Mthfd2	3,11E-13	0,685214172	1,135324063	1,192831978	2,041762698
A_64_P129004	Kcnj12	1,99E-19	0,863527901	-1,1600923	-1,090514581	-1,297637377
A_64_P129855	Meis1	1,02E-12	0,647577708	1,058747003	1,044984001	1,108402208
A_64_P130201	Trpv1	9,05E-13	0,693549958	1,82778328	-1,149771826	1,986381045
A_64_P130239	Slitrk3	1,40E-09	0,617053559	-1,199243234	-1,210552795	-1,587704823
A_64_P130568	Hist1h2bcl1	2,04E-13	0,690074536	2,248465207	2,735445699	2,344054722
A_64_P131122	Iqank1	8,39E-16	0,780716544	1,282784272	1,193108867	2,288705602
A_64_P131370	Calr4	2,56E-16	0,738503782	1,447240413	-1,111653713	1,2256508
A_64_P131451	LOC102550991	1,17E-30	0,940488869	1,251953588	1,040604893	-1,350308077
A_64_P131477	Twist2	2,80E-11	0,650862426	1,009767193	1,229904008	1,319375315
A_64_P131736	Fas	2,99E-14	0,71130191	-1,029644849	1,020553341	1,837169834
A_64_P132044	Spocd1	8,96E-13	0,693660956	1,134234131	-1,613541551	1,980814252
A_64_P132716	Oacyl	2,00E-25	0,897271772	-1,956906763	1,058840255	-1,051935976
A_64_P133167	Duoxa2	1,04E-15	0,793788071	1,018156935	-1,28674082	1,4384247
A_64_P133502		8,39E-15	0,703536581	-1,346747792	-1,824384351	-1,351898932
A_64_P134150	Tex26	1,38E-17	0,764541052	-1,615482811	-1,436146067	-1,153665834
A_64_P134263		9,38E-15	0,759315033	2,47489972	1,269163582	-1,022795416
A_64_P134460	Jsrp1	6,06E-18	0,804419086	-1,603713989	-1,075537774	1,086976914
A_64_P134744	Hpn	2,13E-18	0,825690953	1,173046831	2,309076227	1,618331432
A_64_P135152	Msantd1	1,40E-19	0,830185087	1,326523285	1,015063485	1,043088632
A_64_P136394	Col20a1	1,76E-11	0,656931213	1,382172375	-1,007075363	1,87837876
A_64_P136512	Ecel1	5,39E-15	0,764419578	1,33223537	1,522368906	1,132790483
A_64_P136751	Rgs6	6,42E-10	0,628660836	-1,067178939	-1,087543874	-1,576679253
A_64_P137070	Zfp318	1,81E-12	0,664069349	1,013805489	1,058610009	1,142215288
A_64_P137262	Fam163a	1,26E-12	0,66854866	1,316852309	1,366175647	1,289450061
A_64_P137461	Bspry	1,12E-12	0,744739134	1,834336985	1,103484223	1,204544197
A_64_P137784	Plvap	3,84E-27	0,911334354	1,412854102	1,036657965	1,093510065
A_64_P138011	Adra1b	3,63E-21	0,851869344	1,33058352	1,064433095	1,320681241
A_64_P138565	Narf	4,61E-18	0,773612625	1,17105239	1,071150638	1,165069735
A_64_P138575		1,95E-13	0,746419185	1,27718983	1,229465055	2,139033756
A_64_P139250	Tmprss9	7,87E-32	0,934374645	1,490418451	1,407697201	1,997344654
A_64_P139457	Zfp14	8,26E-13	0,67365378	1,083328041	-1,438504755	1,241512141
A_64_P139485	Plcd4	1,43E-08	0,60215902	-1,202808335	-1,074180413	-1,305916722
A_64_P139802	Prph	2,06E-11	0,745081454	1,165139915	-1,019148734	1,426657515
A_64_P140000	Myo15b	2,11E-25	0,921015326	1,412932377	1,23111019	1,444084036
A_64_P140025	Tyrp1	6,08E-12	0,690618941	-1,059246214	1,400128257	2,194010774
A_64_P140225		7,50E-25	0,892077596	1,38422153	-1,112360039	-1,245348997
A_64_P140323	LOC108349386	3,69E-24	0,885477122	-1,058324888	-1,399939111	-1,813009394
A_64_P140652	Ccdc84	1,18E-08	0,605298499	-1,010504285	-1,015567365	1,304517308
A_64_P141737	RGD1566325	5,85E-13	0,677778004	-1,731170776	-1,604718632	-1,239438511
A_64_P142141	Cldn23	3,51E-13	0,704359338	-1,246069757	-1,152863367	-1,258186738
A_64_P142937	Cabp2	1,01E-16	0,747120738	1,060461849	1,023537003	1,168456317
A_64_P142988	Tmprss9	1,99E-29	0,954877387	1,374141013	1,110322872	1,89093792
A_64_P143480		8,47E-27	0,916625045	1,551121933	1,096066658	-1,369610246
A_64_P143810	Vom2r46	1,94E-18	0,780529526	1,706752328	-1,148254629	1,791575323
A_64_P144123	Olig2	3,03E-24	0,886315176	1,061129858	1,026196848	1,304714059
A_64_P144134	RGD1566029	3,76E-16	0,801912623	1,345285919	1,843125421	1,324987363
A_64_P145433	Ahr	1,56E-08	0,621429522	-1,029154231	1,012938328	1,214923867
A_64_P145829	LOC685324	2,57E-28	0,926989822	1,15189411	1,163584283	-1,377958845
A_64_P146970	Cacng2	3,49E-17	0,805955506	1,278642601	1,006360641	1,279574698
A_64_P147164	Tubg1	3,62E-13	0,703996965	1,171517523	1,018695018	1,137776501
A_64_P147190	Fdps	3,24E-11	0,648875176	1,060196196	-1,017261693	1,117306042

A_64_P147373	Gja5	1,83E-11	0,608939442	-1,035177726	1,028483237	1,230013261
A_64_P147438	Slc35f4	1,38E-17	0,764507535	-1,114856821	-1,045710947	1,817957534
A_64_P147913	Optn	3,55E-14	0,687735859	-1,017589733	-1,121669757	-1,232698448
A_64_P148508	Prr7	7,09E-14	0,756373858	1,197226983	1,007996137	-1,198481553
A_64_P148535	Napepld	9,86E-13	0,746091641	1,015132185	-1,383421229	1,032674223
A_64_P149280	Vegfb	1,34E-18	0,783422439	1,007359497	-1,077066552	1,175473716
A_64_P149735	Sqstm1	1,31E-15	0,76065426	-1,18087167	-1,250682435	-1,442275649
A_64_P150397	Tex38	1,66E-15	0,740463961	1,414299337	1,056807676	1,046092916
A_64_P150509	Ccr10	1,88E-17	0,795935719	1,427580272	1,006859116	1,261779179
A_64_P150726	Ahsp	5,40E-11	0,618951568	-1,489506181	-1,126652871	1,004981469
A_64_P150876	Cst6	8,20E-11	0,657499619	-1,122891431	-1,078514629	-1,29510414
A_64_P152252	Fscn2	1,31E-26	0,907196407	1,155984321	-1,087398136	-1,515775507
A_64_P152800		3,02E-23	0,886203979	1,45576765	1,319628151	-1,187790501
A_64_P152919	Gprin3	1,67E-10	0,626189694	-1,382150101	-1,250477943	-2,212792758
A_64_P153062	Tspan9	1,20E-23	0,890135748	-1,071658008	1,055245575	-1,379507691
A_64_P153149	Kcnab1	1,38E-14	0,719399649	1,09066183	1,073895446	1,100239978
A_64_P153596	Adam4l1	2,29E-12	0,637181711	1,068700338	1,030777366	1,532907943
A_64_P153694	LOC108348293	2,33E-18	0,837751856	-1,057536742	-1,102434501	-1,305427377
A_64_P154082	Tp53bp2	4,29E-10	0,634505697	-1,024588629	-1,09761165	-1,132086104
A_64_P154430		1,45E-11	0,698898255	-1,668175182	-1,060742288	1,085229116
A_64_P155311	Prss22	1,40E-20	0,844200963	-1,303087715	-1,006756293	-1,567537764
A_64_P155393	Ccnd1	1,78E-12	0,685550633	-1,212571633	-1,318543648	-1,092181633
A_64_P155795	Lpo	3,59E-12	0,655394585	1,2640536	1,237144369	1,284547323
A_64_P155983	Vegfa	8,19E-12	0,723151336	-1,145972997	1,138099766	-1,536748461
A_64_P156263	Lmcd1	9,71E-17	0,782946717	-1,190723311	1,557031844	1,838003011
A_64_P156632	Itpr1	5,85E-10	0,630012598	1,08351085	1,055192569	1,271463688
A_64_P156662		1,25E-18	0,815687745	1,056288942	1,045141949	1,214913416
A_64_P156762	Gpr156	1,80E-11	0,634168753	1,855100247	1,282267748	1,496481454
A_64_P157239		1,12E-19	0,831608708	-1,173339401	-1,036771812	1,202551884
A_64_P157244	Sfxn2	1,56E-15	0,759017729	-1,016867123	1,077173528	1,393200118
A_64_P157604	Cpne7	6,25E-11	0,639984224	1,01570491	1,039923174	1,504807548
A_64_P157743	Ccl20	1,72E-29	0,911804023	1,031789471	1,091675077	1,519894094
A_64_P158441	Rfx2	1,32E-15	0,742644587	-1,333101338	-1,103005991	-1,278146849
A_64_P158883	Mxd1	5,60E-10	0,608500245	-1,215794245	-1,110973737	-1,061139846
A_64_P158903	Has2	1,15E-16	0,836111691	-1,545603483	2,555967071	2,185300897
A_64_P159608	Chml	7,70E-11	0,613902173	-1,697280664	-1,497840444	-1,77630021
A_64_P160008	Nxnl2	1,90E-14	0,768733953	1,010306922	-1,049251736	1,29683488
A_64_P160072	RGD1309651	1,06E-14	0,802504832	-1,061787659	1,249532252	-1,737003788
A_64_P160091	Gpr62	5,39E-22	0,872988534	-1,049640659	-1,060527749	-1,332288478
A_64_P160373		1,46E-15	0,759663231	-1,085102697	1,068508104	-1,631949597
A_64_P160393	Tcf4	2,81E-10	0,618713208	-1,337913695	-1,272229707	-1,506850887
A_64_P161265	Zfand2b	5,04E-16	0,825836404	1,138718815	1,031931506	-1,06514091
A_64_P161354		2,52E-11	0,604353856	5,694416823	10,69859766	8,627856012
A_64_P162002		2,20E-09	0,610182662	-1,095281829	1,036646418	1,763014023
A_64_P162599	Psat1	3,93E-10	0,613753741	1,243406818	-1,017707626	1,098899644
A_64_P162700	Kank3	2,07E-17	0,795187491	1,062090299	1,029351462	1,190404966
A_64_P162760		6,48E-09	0,654296236	1,160653012	1,276465675	1,178173975
A_64_P163160	Lyrm7	7,42E-19	0,787980078	1,06225697	-1,000496729	1,243840933
A_64_P163244	Myom1	5,99E-10	0,607481811	-1,336838465	-1,077046535	-1,043600008
A_64_P163698	Onecut2	9,15E-14	0,78407322	1,205296091	1,020279907	1,220695117
A_64_P164340	Tead1	5,00E-12	0,626799567	1,20613107	1,006080448	1,44308319
A_64_P164547	LOC108349417	1,29E-24	0,899059413	2,284967872	1,149158828	-1,232044615
A_64_P164642	Dqx1	6,06E-12	0,624217126	-1,159929354	1,24339336	1,688178587
A_64_P164682	Etl4	4,15E-12	0,675293632	1,044881865	1,161413308	1,312585998
A_64_P164961	Cyp2j3	4,54E-10	0,633678453	-1,291035651	1,054097071	-1,296655417
A_64_P165913	Syt6	1,34E-27	0,929095258	1,194374691	1,073829959	-1,583148295
A_64_P165986	Sh3bgrl2	2,16E-13	0,689418566	1,731593807	-1,814386348	1,700678339
A_64_P229432	Pparg	6,53E-21	0,860307858	1,055724031	-1,112648964	-1,407276192
A_64_P237784	Rbm24	8,63E-30	0,947020639	1,045962856	1,054257081	1,273855319
A_64_P280765	Cd44	8,11E-09	0,631667418	-2,018398823	-1,030645079	1,681468439
A_64_P281840	Nrg1	3,28E-11	0,706930438	1,256995546	-1,583636528	-1,204498252
A_64_P286274	Ttll7	2,15E-17	0,778781069	-1,074740978	-1,11531116	1,282215657
A_64_P370146	Nrip1	7,78E-10	0,60351711	-1,604281655	-1,696298858	-1,879850204
A_64_P393739	LOC108348277	1,28E-13	0,715422798	1,267923686	1,154754982	-1,246776338

Supplementary Table 5. Lists of genes with significant temporal expression changes in CGNs at the intersection of the four experimental conditions (K5 vs K25, K5+SP vs K5, K5+PACAP vs K5, K5+IGF1 vs K5)

			0.5 h				1 h				3h			
ProbeName	GeneSymbol	p-value	K5	PACAP	IGF	SP	K5	PACAP	IGF	SP	K5	PACAP	IGF	SP
A_42_P4539	Ifitm6	2,45E-18	-1,0334406	-1,3512965	-1,1842331	-1,0608517	1,16335269	1,10241	1,31211744	1,08854352	3,20491946	1,69952349	-1,6320475	1,79776008
A_42_P4622	Rprml	2,45E-31	1,10105413	1,20447471	1,03861315	1,07547803	1,56284435	1,19677727	1,28210575	1,11965812	3,97345923	1,29315499	1,20779474	-1,045325
A_42_P4734	Unc13d	5,48E-19	-1,9683134	1,84955438	2,10206692	2,02208452	1,42355959	1,13344939	-1,0715609	1,12172222	5,5690394	1,41804528	1,18497104	1,0219405
A_42_P4847	Ctgf	2,06E-21	1,29623908	-1,0326849	-1,1410669	-1,1551109	-1,085141	-1,0501954	-1,0886356	1,87886019	1,04272173	-2,0973893	-1,3227585	1,69950677
A_42_P4914	Gnat1	2,92E-23	1,12424253	-1,1713045	-1,0236475	1,02927669	1,0501505	1,02096309	1,08010012	1,11517626	1,82092153	1,33570721	1,51200467	1,39214473
A_42_P4942	Armc9	1,06E-09	-1,1083207	1,05147837	1,00755796	-1,0267129	-1,1111632	1,08717253	1,11866058	1,02124583	-1,3825815	-1,0939087	-1,0765886	-1,1725349
A_42_P5012	Adra1d	1,17E-24	1,23392339	-1,1149746	-1,0346297	-1,0223727	-1,1455338	-1,2247693	-1,0526608	1,01386522	-3,7348388	1,38582434	1,37322665	1,34458774
A_42_P5064	Slc25a25	3,29E-12	1,00593257	1,01651303	-1,0500541	1,20674196	-1,4163981	-1,0147405	1,17942435	1,19346206	-1,8889274	1,22710019	1,3790065	1,28243538
A_42_P5367	Sftpc	2,61E-23	-1,4081882	1,56992879	1,52410229	1,19133218	2,95506575	1,14127817	-1,0888162	-1,3647872	6,83282245	1,45210029	-1,5242111	-1,3416618
A_42_P5470	Pak4	2,91E-10	-1,1607371	1,2055466	1,11530591	1,12462059	-1,1218131	1,05137724	1,1246795	1,08776811	1,0786148	-1,0078112	1,43830009	1,2105719
A_42_P5476	Fam49a	1,15E-11	1,9248158	-1,3898281	-1,3850747	-1,1646081	1,13361807	-1,2552141	1,21163147	-1,1713241	-1,6767022	-1,5668711	-1,5601939	-1,29092
A_42_P5484	Acot1	1,18E-12	1,18861001	-1,1241586	-1,04697	-1,096765	1,33561555	-1,1153939	1,03475663	-1,1527721	2,15731723	-1,4248677	-1,5713753	-1,8019999
A_42_P5551	Izumo4	5,38E-13	1,08003399	1,0618453	1,09818372	1,09398116	1,1335836	-1,1465744	1,0838229	1,07910938	-1,1711033	-1,341338	-1,2558126	1,27596835
A_42_P5675	Traf4	4,67E-12	-1,0055682	1,01300387	-1,0362928	-1,2747489	-1,5300782	1,44424311	1,29768578	1,05510712	-2,0713376	1,22417236	1,56982724	-1,1434946
A_42_P5887	Mthfd2	8,01E-20	1,11841787	1,09301842	-1,0242211	1,14446847	-1,0741379	1,0445351	1,2389173	1,09651841	-2,0007078	3,46777576	2,15858651	1,06654819
A_42_P6367	Gzmm	4,82E-15	1,71794387	-1,245285	-1,0256451	1,07642392	1,52144548	-1,1656878	-1,2862662	-1,0364348	2,32879208	1,26998301	1,49966639	1,41960655
A_42_P6739	Mrnip	3,96E-20	-1,0832755	1,01830099	1,09626874	1,00797365	1,13166542	-1,0719505	1,01803716	1,00552312	2,12078592	-1,2538152	-2,1229098	-1,2689879
A_42_P6825	Nr4a1	1,03E-28	1,08714639	2,08413458	-1,0568255	-1,0534911	-6,2555637	4,48161681	1,63414217	2,27622525	-15,254276	1,70030517	-1,1291646	2,29671222
A_42_P6838	Ccl27	3,87E-20	-1,0334433	1,03932039	-1,0356958	1,01145276	-1,0016371	-1,03982	1,30259937	1,03831987	-1,4951499	-1,1400056	-1,0404217	1,18312528
A_42_P6868	Siglec1	6,32E-10	-1,3184952	1,54418244	1,28585906	1,0728878	-1,1646284	1,35249671	-1,3455918	1,17735514	-1,3737382	1,30203818	1,14564642	1,41408777
A_42_P6950	Csf1	2,40E-18	1,11629557	1,09014916	-1,0361405	-1,098676	-1,0415658	-1,3179141	-1,2147347	1,01556083	-1,6259941	-1,1932584	1,47226148	1,32589644
A_42_P7183	Sstr3	6,97E-37	-1,1722252	1,19914989	1,39159317	1,30004215	2,44261491	1,19338849	1,19416934	1,14064481	6,66084034	-1,2358069	1,35239548	-1,012538
A_42_P8023	Fam110b	9,61E-10	1,04957882	1,07097328	1,09887413	-1,052367	1,23292693	-1,1584145	1,08241967	-1,1089518	2,36812358	-1,3515576	-1,3222503	-1,2596648
A_42_P8128	Smoc2	9,92E-09	1,07393431	1,00982902	1,03236535	1,27223792	1,09294138	-1,1998225	-1,2707789	1,0079405	1,48914598	1,97958594	-1,7788309	-1,1954011
A_42_P8206	Il1r2	8,45E-13	1,02536688	1,01358369	1,07454527	-1,0287354	1,15816304	-1,1056068	1,21190232	1,02456747	1,40537115	1,03941769	-1,1775753	-1,1682336
A_42_P8282	Lgr4	9,43E-12	1,28231733	1,08517775	-1,1133993	-1,2124438	1,12842997	-1,1782765	-1,1155999	-1,2388307	-1,1063061	-1,2850823	-1,2296385	-1,0759889
A_42_P8428	Reep6	5,65E-09	1,28517257	-1,1996526	-1,0617553	1,07616823	1,00841367	-1,3368749	-1,1966863	-1,1567664	-1,0399129	1,09707125	1,42138182	1,14206719
A_43_P1010	Spry4	1,06E-17	1,24440278	1,15193314	-1,3194328	1,09397248	-1,3889774	1,89760679	1,12541614	-1,0449441	-7,9117163	2,74256288	2,39502464	2,08828975
A_43_P1049	Bag2	6,17E-28	-1,0073166	-1,0399545	-1,083211	1,11860428	-1,1705656	-1,1155787	1,17818362	1,08739426	-3,0937402	-1,5972256	-1,0232631	1,18561442
A_43_P1093	RGD1562339	2,49E-09	1,26379209	-1,1786787	-1,2586438	-1,2109254	1,12238754	-1,13035	1,26671345	-1,1966609	-1,5043506	-1,3070012	-1,048682	-1,0584598
A_43_P1104	G0s2	1,50E-20	1,45487274	-1,0066303	-1,1897788	-1,0612278	1,27256867	1,024339	1,32997808	1,107839	-2,4635727	1,73254809	1,75683926	1,37658882
A_43_P1144	Atp4b	1,80E-19	-1,2430364	1,3927677	1,70536941	1,51613523	1,83586982	-1,7257548	-1,6882044	-1,6026268	8,26700323	1,03892701	-1,9383347	1,10194269
A_43_P1156	Aqp5	1,97E-12	-1,8380563	1,94073983	2,08114166	1,69971978	1,17914654	-1,1294538	-1,0358545	1,09906011	2,78692591	-1,0315794	1,43502771	-1,0500093
A_43_P1161	Atf3	2,58E-17	2,97416849	1,07051569	-1,102246	-1,0533928	1,40441286	1,20363274	1,46355998	1,3191744	2,57524232	1,49897841	-1,2576911	-1,2949797
A_43_P1168	Id2	4,67E-17	1,57473862	-1,0524717	-1,1512629	-1,1231553	1,41068378	1,32114404	1,28965044	-1,0351066	2,68718912	1,06417472	-1,0271368	-1,46832
A_43_P1182	Kcnj8	1,31E-13	1,08521012	1,23290708	-1,0101454	1,00247392	1,08281077	1,22527259	1,16016305	1,19021229	1,22578144	1,57263235	-1,242524	-1,0055026
A_43_P1193	Nr4a3	1,78E-34	-2,0909356	1,5081482	-1,3001227	-1,2915909	-13,034838	2,14501917	1,59607347	1,11703168	-51,540778	1,40096062	1,31969762	1,65987799
A_43_P1258	Nfyb	3,65E-13	1,12121904	1,02733813	-1,0218058	-1,0822666	1,25269201	-1,0841017	-1,0754081	-1,1126049	1,27319364	1,17124883	1,09878587	-1,2237311
A_43_P1282	Nme3	1,04E-14	1,0554642	1,02797177	-1,005814	-1,0982053	-1,0334268	-1,0963718	1,20851916	-1,0826857	-1,5213976	-1,2794299	-1,0332301	-1,1271085
A_43_P1296	Cap2	1,96E-14	1,16753963	-1,0080834	-1,1416107	1,01967174	1,0067968	1,02548144	1,15862794	1,04020171	-1,2972843	-1,2038951	-1,1498417	-1,1902794
A_43_P1310	Cyp26a1	1,97E-13	1,29178902	-1,0260458	1,10756881	-1,2202972	1,52796347	1,31584759	-1,1268724	-1,3860105	-1,127381	2,6375059	1,74532768	-1,1692718
A_43_P1393	Grem2	7,78E-11	1,52537998	-1,2891402	-1,078909	-1,1435238	1,43889634	-1,4062546	-1,0221239	-1,2248009	1,88814996	-1,334074	-1,9261231	-1,4123422
A_43_P1475	LOC1025547	8,04E-13	-1,0906763	1,10634003	1,03061844	1,0790467	1,34850753	-1,0219873	1,23318206	-1,0038024	2,23968682	-1,3106647	-2,4217322	-1,3473643
A_43_P14809		1,87E-09	-1,2382026	1,16348997	1,15768303	1,22882998	-1,3886769	-1,0166233	1,03614456	1,17105858	-1,0603952	1,23096687	1,5269409	1,48196751
A_43_P1539	Smad7	2,05E-14	1,01411188	1,38314805	1,08081292	1,26194331	1,54595653	1,02375412	1,12781596	1,16209302	-1,0171116	-1,0629793	1,37341441	1,2767183
A_43_P1554	Gabra6	3,08E-13	1,00233643	-1,1336747	-1,2061542	-1,363236	1,27300555	-1,0249289	-1,1643251	-1,2242595	-1,2821861	-1,4704045	-1,1273768	-1,1234546
A_43_P1570	Rab13	5,23E-11	-1,1312491	1,21277274	1,04744536	-1,05626	1,0441529	1,11286908	1,14732312	-1,0421881	1,26490273	-1,1476537	-1,1817036	-1,5695434
A_43_P1593	Chrm4	2,32E-12	1,20374722	-1,1122362	-1,0067349	1,00914428	-1,2156978	-1,3336729	-1,2287415	1,07967333	-3,0868681	-1,0438725	1,53085771	1,41030466
A_43_P1644	Pelo	5,38E-11	1,01523587	-1,0794834	-1,0399614	1,15394593	-1,2931319	-1,2340567	1,17423228	1,03836689	-1,7011369	-1,1743177	-1,0603045	-1,0870628
A_43_P1649	Col16a1	9,36E-24	1,07543212	-1,0064308	1,04528349	1,20733799	1,13619037	1,00084086	1,00373512	1,10026351	1,66063113	1,87744709	1,84514865	1,3090906
A_43_P1857	Tekt4	2,37E-18	1,18321578	1,07820524	1,05973339	1,02059028	2,42737079	-1,4748629	-1,3402284	-1,0402614	2,49596882	-1,3868668	-1,3400636	-1,2657618
A_43_P1974	Krtcap3	5,30E-18	1,47033262	1,09694262	1,14689724	1,06984938	1,76794859	1,03584418	1,2068495	1,14842374	1,39032487	1,33177971	1,16731738	1,28151416
A_43_P2111	Klhl24	7,44E-14	1,54220305	-1,0578358	-1,2113148	-1,5808417	1,22905115	-1,0186945	-1,1558027	-1,4461355	1,3958385	-1,6571497	-3,3169164	-1,5948134
A_43_P2281	Cd6	1,40E-22	-1,3504958	1,52227154	1,54996846	1,66009293	2,32209185	-1,7544367	-1,5603697	-1,1408352	13,371904	-1,021495	-1,745929	1,06669321
A_44_P1011	Znhit6	1,49E-09	1,14480565	-1,3224645	-1,178913	-1,0915873	1,02545737	-1,0722884	-1,3821728	-1,0249676	-2,4962699	1,20255751	1,52190354	1,30430593
A_44_P1012	Stbd1	8,51E-10	-1,2790927	1,64837193	1,34454413	1,14719467	-1,218187	1,17865499	-1,1128397	1,18343794	1,13120563	1,79855508	1,00323372	1,21998483
A_44_P1015	Slc22a3	1,43E-16	1,14499022	-1,4661555	-1,0220595	-1,2373934	1,47476846	-1,2308399	-1,3380894	-1,2424003	2,44147985	1,61099777	1,44181604	1,54221296
A_44_P1019	Cdc42ep2	5,00E-15	-1,063985	1,19245641	1,11019959	-1,10107	1,17423325	1,52901338	1,54708003	1,14805599	2,68473228	1,28845049	1,12021446	-1,3836596
A_44_P1021	RGD1561796	4,93E-08	-1,334471	1,38589438	1,26801503	1,30750589	1,15699563	-1,1555361	-1,0468216	1,00693557	1,45802242	-1,0114108	-1,2041894	1,14501179
A_44_P1024	Hdac5	5,36E-21	1,09687609	-1,0407354	-1,1293911	-1,1682206	-1,3948917	1,16270372	1,34329396	1,13574995	-2,7848247	-1,512149	-1,7032169	-1,1114303
A_44_P1035	Nmt2	1,42E-11	1,26856816	-1,0179161	-1,0713811	-1,0155389	1,06631749	-1,0833563	-1,1961218	-1,0993828	-1,1210614	-1,2258643	-1,0718023	-1,2128057
A_44_P1039	Rasip1	1,40E-12	-1,2684579	1,03879014	1,21247344	1,22618372	1,01991687	1,12934354	-1,0420739	1,22779462	1,67105423	1,31899665	1,39608328	1,149626
A_44_P1043	Crb3	2,94E-15	1,38732326	1,04938813	-1,0242776	-1,3031397	1,73702379	-1,3268065	-1,3181637	-1,2015023	2,55673572	1,24397147	-1,182712	-1,0957118
A_44_P1045	Stac3	7,10E-14	-1,8308201	-1,1011874	-1,2354413	2,05546928	2,13215014	-1,0356682	1,57064144	1,44788964	2,07834045	2,56601624	2,21208067	3,29549733
A_44_P1047	Herpud1	9,88E-22	1,30511428	-1,1112784	-1,237013	1,03181964	-1,1227343	-1,0252906	1,06431673	-1,100954	-1,1048727	3,38054942	1,27802658	1,03236655
A_44_P1050	C1qtnf12	1,91E-17	1,76478789	-1,2102004	-1,0181924	1,30993839	1,75434402	-1,4085148	-1,1271507	-1,0691744	1,78537845	1,52981297	2,2739407	1,606742
A_44_P1057	Ddx25	8,86E-16	-1,1279104	1,18401561	1,10674837	1,25107872	1,13183319	1,0285718	1,07467837	1,0234493	1,45626103	1,21164279	1,24967938	1,10099016
A_44_P1099	Ahsa2	2,17E-11	-1,1738938	1,09079666	-1,0580268	1,09097892	-1,0455755	1,25434076	1,10810037	1,05961659	-1,8291799	1,18595363	1,66744639	1,32949715
A_44_P114692		4,14E-17	1,00771073	-1,2100576	1,05680455	1,09738966	1,24497462	-1,1380218	-1,1483928	1,07987613	1,38357006	-1,0924242	1,43738267	1,13101545
A_44_P1151	Slc5a5	1,31E-09	1,4176546	1,18753613	1,24238629	-1,168869	1,48821175	-1,3904438	-1,2764685	-1,6103675	1,41609983	1,74526517	1,08192984	1,35796467
A_44_P1187	Arc	3,20E-24	5,52644333	1,28511414	1,18364154	-1,0025607	3,58059005	1,76856256	1,29279959	1,61055659	4,2350681	1,89034281	1,50118287	1,37221472
A_44_P1280	Syt17	1,07E-10	1,07889986	-1,0125194	-1,0358913	1,09562469	-1,134524	-1,1487774	-1,2357429	-1,1375473	1,55988332	1,2830567	1,43123155	1,346396
A_44_P152275		3,57E-10	1,36809956	-1,7302559	-1,4324037	-1,4443341	1,09936647	1,03203639	1,57726603	1,03919356	3,72759319	1,40461503	1,49153168	2,47853903
A_44_P1871	C1galt1	3,81E-15	1,72177265	-1,2964562	-1,412515	-1,2159937	1,14406228	-1,3663571	1,35032134	-1,1880564	-1,9400543	-1,6737045	-1,5084621	-1,8033365
A_44_P1924	Dusp9	2,95E-27	-1,0678188	1,16777158	1,45717313	1,46861353	4,45686314	1,12224083	1,11147673	1,03789372	5,72570245	1,86879096	1,75492454	1,31496886
A_44_P1987	H1f0	5,44E-22	-1,1851964	1,30009479	1,31805913	1,18069604	1,63279515	-1,0639567	-1,3653199	-1,0239035	2,88354299	1,36954845	1,09507659	1,33376507
A_44_P2240	Kcna4	2,85E-31	1,25321207	-1,1535717	-1,2153844	-1,4468859	-1,2138716	-1,0175226	1,07970233	-1,1748579	-5,9124241	-2,4333604	-1,9094817	1,02584531
A_44_P2272	Syde2	1,20E-19	1,25615692	-1,008219	-1,1195693	1,17647478	2,66487937	1,3060972	-1,2543138	1,12319896	2,40900514	-1,1775228	-1,330625	-1,2850136
A_44_P2312	Insig2	9,33E-11	1,49739372	-1,2365742	-1,2276626	-1,3775849	1,24373943	-1,1088589	1,03453461	-1,2619333	-1,1164049	-1,436384	-1,7438117	-1,6618249
A_44_P2364	Lnx1	8,62E-24	1,1421257	-1,1064154	-1,0637329	-1,2054283	1,25514987	-1,0559388	-1,084018	-1,163722	-1,2988639	-1,5562637	-1,6183762	-1,1406835
A_44_P2366	Lrrtm3	5,03E-24	-1,0015637	-1,0161237	-1,0730258	-1,2513124	-1,2005355	-1,0571939	-1,0374919	-1,1273798	-1,7949226	-1,5810729	-1,2118145	-1,0704681
A_44_P243477		6,70E-15	-1,0953081	-1,0687861	1,16361773	1,35114065	1,48315788	-1,2467525	-1,5557597	-1,0831562	3,35167997	1,02886914	-1,2579491	1,02747491
A_44_P2435	Pxdc1	5,49E-12	1,30119068	-1,2800953	-1,0750086	-1,0950985	1,34728307	1,05563279	-1,002042	1,03895289	1,4240923	-1,5290905	-1,7008558	-1,3129923
A_44_P2461	Tle1	1,00E-13	1,62376929	-1,1038729	-1,2182448	-1,5046985	1,20467609	-1,1949365	-1,2709147	-1,3476928	-1,3762078	-1,4812348	-1,4612904	-1,2995969
A_44_P2480	Thbs2	1,91E-09	-1,1777825	1,41447675	1,27346004	1,05952379	-1,0893103	1,04309282	-1,1987487	1,01302069	1,12823528	1,17537426	-1,0915428	1,25098114
A_44_P2532	Adamts9	2,01E-17	-1,0510509	1,07163773	-1,0559312	1,00069383	-1,0798354	1,06111609	1,12621374	-1,0408909	-1,4154181	1,33130014	1,41271617	1,27444914
A_44_P2655	Crybb3	4,49E-16	-1,7590166	1,60376446	1,31183273	1,38947842	1,42409171	-1,1261884	1,11221428	1,07913667	2,04283583	1,19153263	-1,0730936	1,15543225
A_44_P2699	Mfsd2a	4,28E-10	1,04802116	1,00748678	1,03062926	-1,2145779	1,28849784	-1,0142849	-1,0288879	1,04978664	1,25269002	1,25584048	1,60937356	1,32020205
A_44_P2737	Masp1	2,43E-13	1,05608664	-1,0554758	-1,3017411	1,06370843	1,18210254	1,14796958	-1,0753328	1,12515104	-1,735803	2,96385387	1,93029467	2,49816713
A_44_P2821	Pdia2	1,68E-27	1,30803204	-1,0704213	1,13285577	1,53624099	2,39616539	-1,0919741	-1,0820245	1,32302508	1,8226514	1,4102831	2,0360654	1,80809778
A_44_P284067		1,41E-10	1,1242129	-1,1698713	-1,3263134	-1,093071	1,01134613	1,07956681	-1,0143335	1,03333956	-2,116537	1,21715265	1,06428044	1,20330213
A_44_P2901	Sfrp4	1,89E-13	1,76675083	1,07323492	-1,3300062	-1,7440972	1,86577517	1,28942113	1,15548209	-1,0691988	2,50737839	2,85437602	1,10658479	1,13563369
A_44_P2910	Arhgef19	1,57E-17	1,34880264	1,06486917	-1,1038309	-1,4104714	1,5920778	1,00420159	1,38960773	-1,192082	3,204406	1,62001514	1,03552164	-1,0448663
A_44_P2929	Slitrk1	1,87E-14	1,40496638	1,04699435	-1,2014243	-1,444007	-1,081514	1,04890543	1,01015291	-1,1983763	-1,1930852	-2,4308093	-2,6155936	-1,3451195
A_44_P2933	Bbs4	2,28E-09	1,44463934	-1,1474814	-1,2318744	-1,3696193	1,10189445	-1,0660558	-1,1764213	-1,1627537	-1,3721333	-1,3770016	-1,4194896	-1,373086
A_44_P3116	Ahnak	2,16E-07	1,06682848	1,23325237	1,18108401	1,02231132	-1,2308755	1,16126649	1,04925018	1,21142635	1,43924748	-1,3613213	-1,4121647	-1,3955062
A_44_P3132	Slc6a13	8,95E-12	1,47106162	1,03657094	1,20811948	-1,64117	1,72434748	-1,5742242	1,39389353	-1,3404195	2,14658425	2,14587479	1,26871005	1,03323377
A_44_P3163	Shkbp1	4,30E-16	-1,0892705	1,168664	1,18666658	1,20444189	1,24563389	1,08330853	1,03242805	1,22533871	1,76663315	1,27087969	1,40694786	1,28216401
A_44_P3280	Masp2	2,35E-23	-1,8572012	1,50187287	1,09371557	1,21576964	2,2956637	-1,2442596	1,07474756	1,04025663	5,657664	2,2211087	1,79327656	1,75290608
A_44_P3284	Rprd1a	1,39E-09	1,30713895	-1,1753999	-1,1365014	-1,2875824	1,12668062	-1,2893551	-1,243783	-1,3613815	-1,9549251	-1,2315065	-1,3339798	-1,2874555
A_44_P3512	Phlda1	3,30E-22	1,13223419	1,15874284	1,01414816	-1,1400896	-1,8947748	1,48128034	-1,0930592	1,34950807	-2,8042024	1,20155901	-1,28044	1,40048215
A_44_P3517	Cldn10	4,09E-12	-1,1811726	-1,0034368	1,25015477	1,22009945	1,2820798	-1,0424573	1,12816836	-1,1654035	1,7743672	1,59275206	1,58265884	1,69639025
A_44_P3540	Hist1h1a	2,77E-10	1,21619565	1,22006547	1,0290557	1,16580248	3,06787427	-1,0000292	1,0326201	-1,0519379	3,62477891	-1,6142726	-1,6903075	-1,5391572
A_44_P3550	Adssl1	2,49E-10	1,15510434	-1,3081959	1,035647	1,23482738	-1,1458741	-1,0171381	-1,2650787	1,09840888	1,39977601	-1,1393101	-1,1618888	-1,1282792
A_44_P356829		3,21E-12	2,01027281	1,34127224	-1,1079806	1,04426083	2,88694507	-1,2912196	1,19542962	-1,0851565	1,89778275	1,84259396	2,52647473	1,91250163

A_44_P3748	Klk1c3	3,70E-13	-1,6252583	1,39338454	1,61457567	1,30420818	1,67298541	-1,1287524	1,86583943	-1,0686384	3,61253847	2,11095665	1,95214883	1,93938852
A_44_P3756	Rbm11	4,83E-19	-1,0111093	-1,1740494	-1,1131473	-1,4298624	4,0092092	-1,2913437	-1,0463166	-1,2262591	6,32184253	-1,3263928	-2,0388884	-1,1356724
A_44_P3787	Flrt3	2,16E-11	1,50656825	-1,003344	-1,1952993	-1,4826594	-1,1440401	-1,5305841	1,07234795	-1,0926958	-1,4810881	-1,2613267	-1,3310811	-1,1315197
A_44_P3798	Pspc1	7,26E-13	-1,0617055	1,11134044	-1,001789	-1,1129615	1,12518647	1,14329256	-1,3041578	-1,1188467	1,13879541	1,00172067	-1,0663799	-1,1255148
A_44_P3819	Sphk1	2,65E-13	-1,0866784	1,47210798	1,32307178	1,07700506	1,37865398	1,67773936	1,01955606	1,23712096	2,94847345	-1,0421029	-1,3358165	-1,0454515
A_44_P4003	Maf	6,04E-10	1,6915538	-1,3185942	-1,2380278	-1,4285274	1,16160517	-1,2225225	-1,1488643	-1,1694517	-1,2020259	-1,2915682	-1,4967731	-1,2276107
A_44_P4098	Faslg	3,85E-19	1,12268204	1,01848359	-1,0043227	1,18143666	1,37690293	-1,0597823	1,08570871	-1,2920466	1,65660294	1,43990362	1,37506675	1,20197038
A_44_P4206	Nup43	1,68E-19	-1,1069585	1,05216966	1,01571809	-1,0398801	-1,0466283	-1,0641963	-1,0038003	-1,1101444	-1,7362478	-1,1401108	1,15758067	-1,0968487
A_44_P4283	Hmgcs2	6,88E-10	-1,4375247	1,40225664	1,30038992	1,12360805	1,12405389	1,58840829	1,80449323	1,39822884	4,27882166	-3,8085226	-2,8130715	-2,2274658
A_44_P4305	Ntrk1	1,24E-27	-1,210288	1,44402726	1,47396222	1,44192507	1,5363989	-1,1404822	-1,5063945	1,03647527	5,68274173	1,65025789	1,35699114	1,47864559
A_44_P4305	Nsmce4a	8,21E-14	1,02691895	-1,154572	-1,0623145	-1,1043298	1,26667075	-1,0345491	-1,0081796	-1,19243	-1,2076018	-1,1507451	-1,2905753	-1,0968929
A_44_P440944		4,56E-28	1,17106312	-1,4218196	-1,0216071	1,40546486	-1,0239548	1,29707607	1,03283634	1,21621324	2,13121148	4,67903883	1,48086785	-1,1441889
A_44_P4427	Kcnab1	9,98E-15	1,2764726	-1,0409345	-1,3269391	-1,2687251	1,32605666	-1,0923047	1,1185382	-1,4348933	-1,2840445	-2,3844049	-1,7917698	-1,3249073
A_44_P4480	Vpreb3	3,70E-10	1,75336014	-1,0638533	1,02192932	1,04302422	2,04230934	-1,5894075	-1,4627488	-1,3618484	2,85489459	-1,0030443	1,09160102	1,14345109
A_44_P4504	Ttll1	2,33E-11	-1,214121	1,05730031	1,09335219	1,03624174	1,10370381	1,15160575	1,24015179	-1,0000542	-1,1729407	-1,2385477	-1,5107105	-1,3799532
A_44_P4580	Tnfrsf11b	1,97E-10	1,27902416	-1,2255886	-1,0052575	-1,1337174	1,25016335	-1,355091	-1,5751336	1,21824341	1,46367769	-1,8671614	-1,3698884	-1,4535612
A_44_P461383		7,45E-16	1,50836903	-1,7603903	1,00580259	1,44289582	1,35828562	-1,2176537	-1,791512	1,12985654	2,17589789	1,01311785	1,35164511	1,13498277
A_44_P4741	Slc7a1	4,63E-21	-1,0362265	1,06234501	1,06790111	1,1819656	-1,3305081	1,1041838	-1,0826277	1,14415199	-1,4761583	2,20455818	1,45978373	1,12753278
A_44_P4774	Slc2a4	2,30E-15	-1,0561596	1,13244911	1,05109293	1,06567273	1,12162889	-1,0343684	-1,0273455	1,18408997	1,97499496	-1,4896613	-1,2591111	1,35587965
A_44_P4819	Id4	1,87E-13	1,32053667	1,11470981	1,03121635	-1,1978593	1,46040605	1,25175786	-1,4411777	-1,086096	1,24091371	1,45839417	1,1801285	1,34362391
A_44_P4913	Nudt7	7,93E-10	1,06757273	-1,0450115	1,02877444	-1,0954304	1,37016353	-1,1381143	-1,0754422	-1,1648992	2,71775706	-1,4002486	-1,5901133	-1,2411166
A_44_P5004	Lman1	4,50E-09	-1,0066081	1,02504811	1,05255253	1,03694393	1,09529539	-1,0906926	-1,0205686	-1,0045253	-1,3023399	1,20856718	1,269945	1,16850235
A_44_P5017	Crybg2	4,81E-19	-1,4592009	1,10934798	1,53974445	1,17158627	1,31496925	-1,0569955	1,33905319	1,13705715	2,76866743	1,74332917	1,19714641	1,48898591
A_44_P5086	Tm6sf2	1,36E-28	1,15561529	-1,0872373	1,08049246	1,26935595	1,57086648	-1,0453905	-1,0282656	1,18606056	2,41863437	1,28382964	1,24559363	1,1945617
A_44_P5133	Dab2	4,46E-08	1,41820655	-1,0105748	-1,1256271	-1,3682357	-1,0781318	1,25383962	-1,0841686	1,06513924	1,0949432	1,14476062	-1,2701474	-1,3968844
A_44_P5220	LOC684871	3,96E-12	1,43838233	1,2726956	1,10919847	-1,0921954	-1,1486812	1,55889686	-1,8005002	1,37579286	-1,9527353	2,53796227	-1,2948104	1,54441527
A_44_P5322	Fkbp5	2,06E-12	-1,1639663	1,19736946	1,20328688	1,4030669	-1,2357626	-1,0295866	-1,1425246	1,04616935	-1,3270833	1,35873654	1,4592649	1,19791741
A_44_P579382		4,25E-12	-1,799739	1,66937985	1,36249789	1,28376348	1,19946017	1,16145659	-1,1218291	1,17036828	1,91395915	1,0866809	1,13163175	1,17673872
A_44_P6131	Ndp	2,34E-15	-1,1449877	1,05341591	1,08262155	-1,2564203	1,44088148	-1,0931937	-1,2076398	-1,0752197	2,83997616	-1,5354486	-1,7467033	-1,3094729
A_44_P6285	Rgs7bp	4,46E-14	1,37453303	-1,167957	-1,285232	-1,406927	1,01881068	1,04678381	1,02035595	-1,1324274	-1,5521996	-1,3748133	-1,6378483	-1,2781215
A_44_P6385	Paqr5	2,82E-26	1,23429569	-1,2246629	-1,0036912	-1,1579873	1,71565502	-1,7483497	-2,0524758	-1,7751185	10,0257196	1,11865681	1,40027948	-1,52074
A_44_P6393	Col24a1	1,30E-10	1,10760624	-1,1612311	-1,0024071	1,19281039	1,38257363	-1,2435888	-1,2420131	1,10208067	-1,5102983	1,12403075	1,23870223	1,73575084
A_44_P7298	Ppp1r3d	1,69E-14	1,16894542	-1,0358389	1,03656194	1,02726089	1,53221685	-1,1819223	1,24951272	-1,1359122	-1,0372114	1,05022496	1,57772016	1,16924093
A_44_P7774	LOC1003627	2,89E-13	1,06273439	-1,4330863	1,06153089	1,22245084	-1,0236042	-1,4742358	-1,3905203	-1,096593	1,35780007	-1,3153491	1,03193961	1,00244551
A_44_P8224	Ctla2a	3,25E-15	-1,8757656	1,31629247	1,28574942	1,25942627	1,21041617	1,33372815	-1,0224891	1,32121051	2,09346624	1,53498707	-1,6995177	-1,8189747
A_44_P8473	Luzp2	4,17E-14	1,48033695	-1,1643918	-1,4316401	-1,4236621	1,33501641	-1,1196391	-1,0164186	-1,2386817	-1,1894122	-1,7770831	-1,5671785	-1,4199932
A_44_P8763	Vav2	9,13E-24	-1,412457	1,20337757	1,32026642	1,33140001	-1,1810569	-1,1212876	-1,279889	1,02883819	2,44500175	1,91064321	1,66118414	1,36778484
A_44_P8984	Lrrc17	2,68E-16	1,31104668	-1,1597586	-1,2442724	-1,3281254	1,60031757	-1,186434	-1,2784178	-1,202399	1,40828019	1,32557987	-1,3722467	-1,4368855
A_44_P9173	Lrtm2	8,31E-32	1,13851295	-1,1514816	1,10670399	1,25666824	3,11287708	-1,0087774	1,20845403	1,01500604	6,76959742	1,26530533	1,85311699	1,08564537
A_44_P9175	Mat2b	1,32E-15	1,09966482	-1,2167165	-1,078615	-1,1760397	-1,0194229	-1,0930277	-1,0556938	-1,143156	-1,6334545	-1,580013	-1,9036486	-1,2514093
A_44_P9978	Csrp3	5,34E-20	1,1592823	-1,2571412	-1,0826233	1,01568972	-1,0449348	-1,3922161	-1,1298412	-1,042725	-2,1392431	-2,1528551	-1,3888431	-1,2609387
A_44_P9991	Masp2	1,44E-18	-1,2350913	-1,0655748	1,0066473	1,25299256	4,5164003	-1,6475375	1,1446631	1,03876001	2,78807206	2,02990871	2,88530664	2,00351478
A_44_P9993	Lmbr1l	2,06E-09	1,03321276	1,02325651	-1,1749949	-1,0184356	-1,8286847	1,28505247	1,27484725	1,42077502	-1,5258627	-1,249976	-1,982807	-1,0221638
A_64_P0012	Mthfs	7,29E-10	-1,2448475	1,31191655	1,22573374	1,20731225	-1,0109596	1,10006934	1,13798702	1,06568277	1,42473707	1,19338435	-1,2562283	1,19637169
A_64_P0023	Zbed4	8,75E-11	1,0370189	-1,13419	-1,2864737	-1,0606336	-1,0663903	1,01934994	1,13738792	-1,0091652	-2,8066728	1,34562355	2,15755083	1,32024437
A_64_P002899		2,34E-13	-1,3576737	1,44937955	1,29800853	1,05334393	-1,3009662	1,20243709	1,29267831	1,03029923	-1,7093234	2,35882992	2,454368	1,57638082
A_64_P0043	Pipox	1,29E-12	-1,0706266	-1,007414	-1,0225117	-1,3437713	2,36032387	-1,4435341	-1,0978958	-1,179013	3,95163802	-1,2888297	-1,6891947	-1,1567814
A_64_P0093	Reep4	9,78E-15	-1,0058519	1,12644119	1,14766996	1,274795	1,36693706	1,18336969	1,09989937	1,05557086	1,80898687	1,42839126	1,39656624	1,18930784
A_64_P0095	Vmo1	2,01E-24	1,06327378	1,02439385	1,06497292	1,18539531	1,91461179	-1,4210157	-1,2501574	-1,1817896	5,3143583	-1,0007657	-1,4886623	1,18895425
A_64_P0097	Btbd17	6,71E-36	-1,1028507	1,30473386	1,35005825	1,16826621	2,81475041	1,31137658	1,00285561	-1,0734823	17,3583366	1,14233239	-1,2491403	-1,3988079
A_64_P0098	Btg2	1,49E-24	2,55903431	1,64817245	-1,0423458	-1,2935247	1,10646526	2,93279636	1,2427016	1,39138876	3,41781017	1,16189334	-1,3034077	1,04839013
A_64_P010960		4,51E-15	1,16154038	-1,4138927	-1,031178	-1,023778	1,21110657	-1,0828041	-1,3019813	1,08498255	1,65944916	1,14686275	1,25855064	1,28795315
A_64_P0110	Hrh3	2,89E-13	-1,0700218	-1,1595951	1,0079978	1,11211263	1,23659667	-1,0990714	1,21346749	1,14409093	1,64910282	-1,1847909	1,26721447	1,44713039
A_64_P016766		5,58E-14	-1,2485214	1,30203443	1,46867471	1,56975254	2,46072811	-1,6672805	-1,0236985	-2,0527007	5,82205916	-1,6543064	-1,72739	1,13844856
A_64_P018427		3,48E-11	1,68390491	-1,5630931	-1,2597725	-1,4149325	1,05085065	-1,5289469	-1,1095603	-1,2929289	-1,3521945	-1,6764969	-1,7335913	-1,3946712
A_64_P018633		1,75E-16	-1,7586415	-1,0566381	1,30591989	3,89643767	1,0774251	1,09896557	-1,4780103	3,78528322	1,47733888	1,77117999	1,68122136	3,65764442
A_64_P0190	LOC501396	3,20E-11	-1,287544	-1,0920707	-1,1231143	1,28905945	3,19039435	-1,5296981	-1,953586	-1,0133141	1,21382802	-1,1001451	1,47522452	1,91130882
A_64_P0220	Sstr2	2,56E-24	-1,2704406	1,29135566	1,45296308	2,00157708	1,60068189	2,1885859	-1,132779	1,28107473	5,6176739	4,05156223	1,65532822	1,05752444
A_64_P0244	Stum	2,84E-18	-1,0868412	1,06827823	1,10713583	1,02667045	1,18078785	-1,3803738	1,22336937	-1,1231698	2,58592053	-1,2916815	-1,1530551	-1,4571295
A_64_P0250	Ccl1	3,87E-16	-3,4319472	2,04382811	2,60574541	1,59326014	2,6164724	1,04696165	-1,5466531	-1,2294647	11,8936696	1,42919592	-1,6772904	1,45216189
A_64_P0250	Cebpg	2,05E-16	1,15275895	1,05315307	1,01164267	1,01787801	1,15293719	-1,0809731	1,15641012	-1,1138705	1,4385732	1,22390658	-1,0383068	-1,3906734
A_64_P0251	Cebpb	3,07E-14	1,49391748	1,07669214	-1,2076377	-1,3125046	-1,105283	1,62128724	1,38692771	1,09941034	1,00376573	1,95145863	1,15820718	-1,0227668
A_64_P0256	Tbc1d16	1,53E-18	1,13505169	1,31068966	1,17321723	1,26757389	1,1255469	-1,1058723	-1,0245322	1,06254754	1,96230623	1,14596851	1,42062433	-1,2408302
A_64_P027911		4,24E-18	-1,260145	1,63546102	1,6905205	1,27661248	1,28132902	1,45476237	-1,2441936	-1,229849	4,1896634	2,68278846	1,28824313	-1,5821608
A_64_P029077		5,18E-11	-1,7380171	1,39006858	1,23038174	1,41315267	-1,4423754	1,9571367	3,14991684	1,71288578	2,90780112	3,44392255	1,2582521	2,13919085
A_64_P0293	F8a1	1,65E-08	-1,0808753	1,04061814	1,04593062	1,13516451	-1,0630015	1,0493439	1,1254379	1,04571924	-1,1459514	-1,1991152	1,01189147	-1,0613326
A_64_P0295	Tnnc1	2,40E-17	-1,028019	1,14454667	1,04631934	-1,2044164	1,04840832	1,17735388	1,22797523	-1,0304981	1,762582	1,03030689	-1,3212445	-1,1207242
A_64_P0305	Tpcn2	3,46E-27	-1,2486358	1,21447281	1,3734151	1,20337648	1,43844438	1,05098863	-1,0318985	1,1838273	3,53257159	1,41950084	1,47903798	1,17319921
A_64_P0323	Mustn1	3,28E-15	-1,2799385	1,20203068	1,17812569	1,02482029	1,21042037	-1,0567997	1,15454851	1,07958073	1,42497253	-1,6624184	-1,3747209	-1,0937989
A_64_P0335	Tep1	2,24E-09	-1,1329069	1,03475638	1,09262408	1,20357128	1,11432173	-1,1384877	-1,3424642	1,057625	1,66714964	-1,221011	1,02996608	1,27443311
A_64_P0341	Ccdc166	2,48E-29	1,44600436	1,04445099	1,2986503	1,56025464	9,08089478	-1,4913227	-1,0929324	1,01291029	4,71328565	-1,2349426	1,37073391	1,12368863
A_64_P0346	Rpain	1,05E-09	-1,2435641	1,1139835	1,15195046	1,16974739	1,00821505	1,00610672	1,1037578	1,01626494	1,27545773	-1,2224778	-1,4327986	-1,1240168
A_64_P035238		3,90E-29	1,17331303	-1,4930174	-1,1227825	1,03869452	1,35537793	-1,0393443	-1,2696995	-1,0890436	3,24160209	2,66695429	-1,1888776	-1,406013
A_64_P0366	Klf6	1,27E-10	1,31423221	-1,2399267	-1,0372632	-1,6945553	-1,8741033	-1,6789367	-1,1979892	1,06608421	-1,6073773	-1,2365219	-1,5905002	1,03272966
A_64_P038625		1,89E-14	-1,0068624	-1,039644	1,00218414	1,08282634	1,13737898	-1,1877084	-1,2805717	1,01427873	1,53580084	1,07555448	1,01847163	1,28482957
A_64_P0387	Aste1	5,98E-14	1,08386186	1,048959	-1,0134821	1,14315851	-1,3775343	1,0977507	1,06135477	1,13464573	-2,7254446	1,26007706	1,40008313	1,1825626
A_64_P038839		1,31E-11	-1,3532648	1,14318733	1,38920314	1,62756903	1,97582869	-1,8088368	-1,3121067	-1,3645513	4,03314099	1,90519768	2,12559656	2,15352639
A_64_P0389	Prodh1	3,28E-09	1,04323996	-1,1874315	1,00253291	1,25524892	1,234738	-1,271054	1,07811287	1,04767517	1,44440124	1,23990096	1,42759207	1,21341877
A_64_P040095		1,07E-13	1,05870377	-1,5249298	-1,3385649	1,15115338	1,1332487	-1,01778	1,12547547	1,46284518	1,90407968	2,4259319	1,6690701	1,77988021
A_64_P0422	Acvr1c	8,24E-19	1,26126666	-1,0240169	-1,2052253	-1,3710351	1,11470631	-1,1534384	1,28305066	-1,2119314	-1,7704528	-2,2489728	-2,0227882	-1,414374
A_64_P0430	Cbr3	9,66E-11	-1,1268118	1,04006118	1,07058673	1,26378279	-1,1169983	1,59326366	1,31048186	1,17313543	-1,4780422	1,44973023	1,0081983	-1,165129
A_64_P0436	Slc39a5	2,85E-19	-1,3135569	1,12826503	1,40738175	1,88096609	1,43141499	-1,1469931	-1,0040344	1,46785675	3,00584851	1,74622335	1,86287116	2,70413167
A_64_P0437	Fat4	1,63E-16	1,20203148	-1,2423262	-1,3101595	-1,0662886	-1,1961613	-1,0232123	1,19820856	1,02876279	-3,1146067	1,18630422	1,27016445	1,21947829
A_64_P0444	Mrpl50	3,42E-12	-1,044671	1,20207112	-1,0448086	-1,1007575	2,22017366	-1,2018572	-1,247634	-1,6525394	3,31293898	-1,3568767	-1,4708166	-1,1139833
A_64_P0468	LOC363337	2,06E-22	1,2766239	-1,0886699	-1,1370671	-1,014436	1,49935178	-1,1830706	-1,3223331	-1,2867778	2,35232927	3,0257001	-1,2547857	-1,386012
A_64_P0480	Bfsp2	2,81E-07	-1,1053594	1,15119817	-1,2782361	-1,1639851	-1,1195363	1,12368428	1,77081102	1,33555904	1,84490186	-1,2203169	-1,0488283	-1,3993302
A_64_P0487	Cbs	3,76E-11	1,41626073	-1,1739135	-1,1480469	-1,3703295	1,03617616	1,29811171	1,58092833	1,04831312	-1,0465422	1,78001237	-1,0648407	-1,4835554
A_64_P048852		5,35E-10	-1,0621376	-1,0982826	1,10390719	1,18880489	-1,1460688	-1,179438	-1,063395	1,0087872	-1,4409185	-1,314407	1,34548792	1,12089946
A_64_P0500	Pde9a	1,75E-26	-1,0176623	-1,0408459	-1,1071051	1,10720627	-1,0242775	1,06357659	1,05594938	1,12714359	3,45160916	1,4058988	1,34789214	1,1674669
A_64_P051139		1,72E-16	1,20348366	-1,0214878	1,05534743	1,25574241	1,57338751	-1,1616539	-1,2570179	1,12207255	2,89742523	-1,2341954	-1,4776578	1,48688297
A_64_P0534	Bag2	3,63E-22	-1,2593605	1,06920928	1,09947357	1,25706919	-1,1239787	1,04604955	1,23245463	1,16349757	-2,8980397	-1,8806881	-1,0206516	1,26991884
A_64_P0541	Nudt6	6,76E-25	-1,0147498	-1,1359089	-1,0572245	1,07793855	1,74929304	-1,1215318	-1,5733863	1,00459249	3,28001543	-1,2681623	-1,6113981	-1,4062432
A_64_P0556	Timm9	2,32E-21	-1,0437595	-1,0296296	1,06731677	1,08002301	-1,1604535	-1,2161271	-1,2018398	-1,1215713	-2,7496411	-1,007492	1,30862914	1,22587414
A_64_P0580	Gimap1	3,22E-16	1,04207162	1,11901652	1,0574145	-1,144504	1,66553716	-1,2866316	-1,0872113	1,30685956	2,83757548	1,26892737	-1,6888371	1,52171533
A_64_P0580	Rbp4	1,92E-18	-1,0177956	1,06317463	1,21120838	1,33852743	1,22972973	-1,1781124	-1,2237111	-1,0096249	1,92237808	1,13686685	-1,2423958	1,06207283
A_64_P0594	Ddit3	1,66E-19	1,10075792	1,22774421	1,02946493	1,18185759	1,21089192	1,2314034	1,44327572	1,10134392	2,49712597	1,98322258	-1,1032419	-1,2103621
A_64_P0603	LOC365085	1,23E-08	-1,2475428	1,24070117	1,22367479	1,12678573	-1,2821621	1,07787977	1,11675076	1,17351315	1,12028574	-1,0778717	1,21699353	1,31484833
A_64_P060608		1,90E-24	1,24666915	1,13171919	1,12614069	1,32251969	1,89913795	1,21801152	-1,0668831	-1,1506179	6,20341224	3,0098302	-1,7934603	-2,4071808
A_64_P0612	Micall1	1,59E-12	1,19779399	1,1232307	-1,015394	-1,1320408	1,25056029	1,30176763	1,33456664	-1,0333467	-1,0667305	1,34609752	1,36761909	-1,1626913
A_64_P0617	Trpv1	3,52E-15	-1,0976444	1,30593725	1,14223404	1,25067748	2,51041897	-1,8949162	-1,5219704	-1,8277417	4,6537179	1,19264113	1,17667435	1,38012534
A_64_P0617	Fam89a	7,92E-10	-1,3402672	1,16309384	-1,0970199	2,1001264	-1,0781758	1,26483584	1,11271525	3,16468639	-1,3818886	1,54920995	1,79069903	3,7090714
A_64_P0622	Terb1	8,71E-22	1,18957605	-1,7079975	-1,1644082	1,06108829	1,49680279	-1,2890508	-1,2201383	1,09643352	3,79162661	-1,0076638	1,39567826	1,41206989
A_64_P062506		6,86E-28	-1,0612789	-1,0587549	-1,1261173	1,38002133	1,21459263	1,0550576	-1,3222612	1,0153878	2,65567605	3,03698735	-1,1389898	-1,2128144
A_64_P0625	Chst5	1,26E-20	-1,1324103	1,1557683	1,09255893	-1,1189	1,80389796	1,21585773	1,45437981	1,2144731	7,24136615	1,3213461	1,03996293	-1,0492335
A_64_P0630	Rpp25	5,39E-10	-1,0042336	-1,0498212	1,20009515	1,37803054	1,18536572	-1,3618284	-1,315994	1,01513948	1,45705134	1,02270251	1,16276541	1,32120353
A_64_P063644		1,09E-12	-1,1361071	-1,1110299	1,10469211	1,20351181	1,13195646	-1,0434585	-1,1015404	1,10789136	1,3872461	-1,1664958	1,45761398	1,19814094
A_64_P0650	P3h4	6,25E-10	-1,0916582	1,38924114	1,255036	-1,0020248	1,15302236	-1,0971537	-1,0851732	-1,1954735	1,72329322	1,00616025	-1,1617248	-1,1569921
A_64_P0667	LOC1083483	1,80E-25	1,12484735	-1,2365205	1,12985897	1,75341682	1,24724955	-1,0942516	-1,5936845	1,09498745	5,36170967	3,7844706	1,06089252	-1,4685237

A_64_P0690	Lpar6	1,52E-08	-1,0224703	1,10956634	1,25272804	1,20215256	-1,1829231	-1,1643143	1,09962412	1,32104099	1,41689772	-1,1768274	-1,1771621	-1,0816417
A_64_P0693	Scn10a	3,24E-28	-1,0578109	-1,0646817	1,21841306	1,34501205	1,355231	-1,4522463	-1,8616723	-1,3213172	5,0626872	1,42059765	1,05451192	1,10558404
A_64_P0699	Prss56	2,67E-20	-2,2262008	1,5722613	1,4801113	2,23959049	1,62456714	1,55767271	1,51593165	1,64038741	6,66630396	1,46202938	1,49326913	1,36341182
A_64_P0709	LOC501346	3,43E-15	-1,2542336	1,14847423	1,24261944	1,39603978	-1,4183637	1,9033666	2,76680886	2,13625724	4,77228205	4,32519237	-1,0219322	1,50685458
A_64_P0718	Gpr143	1,31E-08	1,19653927	-1,1855934	-1,259565	-1,4033859	1,2470589	-1,0098328	1,00068854	1,21418523	1,34995875	1,1825119	1,00024221	1,32412982
A_64_P0722	Arrdc2	3,16E-12	-1,6469133	1,70074211	1,6546175	1,75662715	1,84216024	1,23587049	-1,1345338	1,25713662	4,37634599	-1,3075229	-2,586977	-1,1839277
A_64_P0750	Camk1g	5,37E-15	1,2972911	-1,0980365	-1,1315249	-1,3184658	-1,0504847	1,40373229	1,38735398	1,10153277	-1,4111668	1,79477109	-1,2528243	-1,1266949
A_64_P0765	Jph1	5,58E-15	1,80182007	-1,4631127	-1,3953079	-1,5818631	1,45268981	-1,2802188	-1,1902427	-1,2999841	-1,7499977	-1,699297	-1,2250332	1,00595495
A_64_P0768	RGD1311447	9,47E-24	1,43025065	-1,3329524	-1,0394291	1,32927153	1,81239619	-1,2043019	-1,2680497	-1,1268966	4,65800601	1,2372639	1,4951622	1,13623921
A_64_P077392		4,74E-14	1,60528624	1,71787588	-1,2459747	-1,4934913	-1,1777491	1,29401176	-1,730489	-1,2451617	1,83548001	1,02164067	-2,7246578	-1,3476242
A_64_P0795	Myh8	8,46E-11	-1,331936	1,50041179	-1,0018553	1,12211613	-1,1202691	-1,0103724	1,09128204	1,06095863	1,13921667	-1,0483071	1,3774068	1,17907211
A_64_P0823	LOC1025532	1,70E-09	1,51457205	-1,456832	-1,29451	1,16077712	1,11741129	-1,2272032	1,10158197	1,29293239	-1,3872962	-1,0494584	1,49344803	1,71943924
A_64_P0839	Zfp68	4,01E-20	1,22593673	-1,229354	-1,3219726	-1,3183106	-1,1636506	-1,1114576	1,02695553	-1,2004054	-1,7859217	-1,1662486	-1,103356	-1,2770575
A_64_P085671		7,44E-20	1,34918596	-1,2606881	-1,1752311	1,02051135	1,45022819	-1,0920178	-1,5054631	-1,2027703	2,75434043	2,37718388	-1,4320665	-1,6324572
A_64_P0867	Runx1t1	9,06E-15	1,21555898	-1,0196397	-1,0418479	-1,2463267	1,2529529	-1,1134038	-1,1104528	-1,3249388	-1,2455218	-1,3457434	-1,5175862	-1,1296584
A_64_P0878	St14	1,09E-14	-1,1364494	1,23345232	1,1256745	1,15644585	1,63425103	-1,3546345	-1,021122	-1,0456242	3,82205013	-1,875708	-2,040859	-1,4430076
A_64_P0883	Angptl4	6,78E-16	1,59197992	-1,1310105	1,02519337	1,15717901	1,3828939	-1,6454362	-1,1298279	1,11798814	3,78910889	-3,4574484	-2,4405958	-1,9782349
A_64_P0883	Hbb-b1	7,98E-09	1,44932375	-2,1558591	-1,4800532	-1,7810121	-1,0464846	1,39306232	2,36828931	1,2594784	2,40481648	-1,5059028	1,32749764	-1,5853268
A_64_P0883	Hbb-b1	5,21E-09	1,60407514	-2,2786047	-1,3809397	-1,7511953	-1,0163249	1,62928354	3,26769824	1,2779441	2,85582742	-1,5001236	1,21632756	-1,960272
A_64_P0883	Sytl1	1,77E-22	-1,1421212	1,15269705	1,22873951	1,14948761	1,2313567	-1,1056692	1,07775954	1,15426512	1,78965885	-1,0971477	1,26225034	1,20790548
A_64_P0889	LOC685668	2,13E-19	1,01155799	-1,1504625	1,04399546	1,06252726	1,18690467	-1,0368461	-1,1680712	1,05535283	1,45602738	-1,0618467	1,25892154	1,18170459
A_64_P0925	Sspo	1,11E-24	-1,2765665	1,09665741	1,52235104	1,32516459	1,41539091	-1,1659368	-1,1920023	1,19811579	4,6228209	1,73693733	1,18975853	1,50984735
A_64_P093044		5,29E-18	1,12429084	1,33661358	1,04475933	1,14999359	1,74173766	-1,2031463	1,04236503	1,1046294	1,46435968	1,88238392	1,59885721	1,37381686
A_64_P0935	Dtna	2,59E-12	1,09724431	-1,0777711	-1,0402768	1,00312596	-1,3005448	-1,1626889	1,02256748	-1,0812261	-1,4195654	-1,2350672	-1,2345078	-1,191339
A_64_P0945	LOC1003606	1,87E-17	-1,0427243	1,0263322	1,02789057	1,14667239	1,01481592	-1,1453241	-1,1305059	1,07957975	1,52813835	1,12462338	-1,0225137	1,22700722
A_64_P0950	Mmd2	2,72E-13	1,38465051	-1,2031431	-1,204039	-1,1722035	1,13008379	-1,0560346	1,25923136	-1,0480237	1,3994537	1,04915437	1,01049478	-1,3505262
A_64_P0967	Plpp4	4,49E-15	1,00323389	1,08792244	1,01453304	-1,0869102	1,08208316	1,00181942	1,22198739	-1,1258736	1,43651174	1,21640741	-1,0741328	-1,215924
A_64_P0975	Mtus1	3,60E-12	-1,1158226	1,11913515	-1,0071697	-1,103449	1,10302586	1,00457356	1,1551473	-1,068508	1,26242181	-1,6566985	-2,7337581	-1,2697359
A_64_P0986	Nkpd1	9,21E-20	-1,0394624	-1,1164577	1,1730691	1,61686196	1,36975816	-1,3121066	-1,2661917	1,10762024	3,35003507	-1,0601978	1,24558338	1,12246558
A_64_P0999	Dap	7,04E-12	-1,083706	1,33171974	1,11830301	-1,072773	-1,0454749	1,12664953	1,04164799	1,03740432	1,81748745	-1,0506708	-1,3092625	-1,2259214
A_64_P100346		2,15E-32	-1,0707005	1,11010585	1,20337271	1,16865126	1,25773487	-1,1303995	1,13297147	1,14354756	2,88947102	1,00461554	1,08800524	1,23225967
A_64_P1004	Hoxd9	1,30E-11	-1,5803312	1,25698433	1,07490119	1,72266646	-1,0942638	1,44106413	2,61881775	1,35714209	3,17759487	3,17197412	-1,4378832	2,26509852
A_64_P1005	Aoc3	1,89E-15	1,68659331	-1,1388179	-1,1589152	1,28359761	2,63908142	-1,6178195	-1,7782275	-1,1977132	5,76880281	1,85171061	1,87550089	1,65887048
A_64_P1015	Sec63	1,15E-12	-1,0539689	-1,0009305	1,02672067	1,08579663	-1,3960632	-1,0934897	1,05271987	-1,0094299	-1,8927015	1,21598865	1,27231275	1,19396579
A_64_P1019	Hmcn2	1,83E-15	1,2396464	-1,2884367	1,01589528	1,22472913	1,31416404	-1,0626721	-1,0684399	1,2651329	1,4788393	1,6584859	1,7620898	1,96405162
A_64_P108459		2,00E-22	-1,1121306	1,00840003	1,31610574	1,82196886	1,31967745	-1,0138559	-1,4246925	-1,2483899	3,79405378	4,82231436	1,09858553	-1,3720376
A_64_P1104	Fdxr	1,93E-12	1,29655787	-1,2069904	1,0105295	1,26841272	1,21021475	-1,0276808	1,00362275	1,05985635	1,47432032	1,03518722	1,30092889	1,09824205
A_64_P1105	Spdef	6,20E-14	1,05628387	-1,4786778	1,30980536	1,2862954	1,05107	-1,2963235	-1,1985514	-1,2142584	1,91175864	-1,4712474	1,00049839	-1,2576997
A_64_P1105	Plekhf1	1,52E-23	1,06661402	-1,0721828	-1,0646748	-1,2399642	-1,6156416	-1,0606334	1,24473311	-1,0192918	-1,4268065	-2,0418832	-1,6193529	-1,1939069
A_64_P112185		4,59E-18	-1,3455563	1,07026212	-1,0247541	1,04210891	1,26539584	-1,1168385	1,01408984	-1,3842947	4,95035717	2,37913378	-2,1005616	1,10067291
A_64_P114396		7,09E-24	-1,5073319	2,37475306	1,90592797	3,36678652	1,68173418	2,8184986	1,26741681	-1,0227109	9,14845849	3,77700816	-1,2247074	-1,4983231
A_64_P1163	Adam33	1,18E-09	-1,1291141	1,17352284	1,17036488	1,12611826	1,2655733	-1,0075157	-1,2778431	1,09283202	-2,3851941	2,1376918	1,7711376	3,01234692
A_64_P1166	Rcan1	1,44E-10	1,35797734	1,24909918	1,16958318	-1,0989376	1,26976295	1,33691855	-1,0787303	1,27298461	1,50992139	1,24402702	-1,5264925	1,59052406
A_64_P1177	Slu7	2,40E-08	1,04435395	-1,0158307	1,03796285	1,02636069	-1,1190253	-1,0821281	1,10280404	1,00232658	-1,0228467	-1,2243099	-1,2615853	-1,18067
A_64_P1198	RGD1311084	1,97E-24	-1,3819364	1,4750991	1,27872301	1,15315644	1,37623058	1,0178881	-1,0188609	1,04984579	3,15769382	1,46845724	1,14962306	1,15593857
A_64_P1204	Cacng6	8,37E-21	-1,1155177	1,01151838	1,01528587	1,04565358	-1,1839897	1,08601153	1,26309189	1,2304462	-1,6079355	-2,433313	-1,3483611	1,44229149
A_64_P1239	Gk	2,03E-25	-1,0550892	1,00905314	-1,0014898	1,04386149	-1,0809017	-1,0606425	1,13335331	-1,0490157	-2,0413894	-1,2158834	1,1265111	1,17124977
A_64_P1240	Ntsr1	2,03E-29	1,03163856	-1,1850875	1,29051261	1,30718268	1,36743005	-1,3194139	-1,1704378	-1,1062526	8,96517944	1,52518379	1,33419136	1,18565411
A_64_P1248	LOC363337	3,79E-18	-1,078446	-1,203562	-1,3238877	2,22649012	-1,0969183	1,53883732	-1,0152651	1,38640867	3,30063121	5,98709225	2,0077242	1,35516498
A_64_P1260	Slc22a7	8,44E-14	1,15651305	-1,4478503	-1,2068375	-1,0703001	2,02545552	-1,3758419	-1,178344	-1,5703695	1,45898112	1,27292287	1,86498247	1,77692042
A_64_P1273	Sys1	2,71E-14	1,16893489	-1,0190439	-1,0950932	-1,2676504	-1,0720715	-1,1648591	1,20167466	-1,2206377	-1,5798618	-1,1616524	-1,0383298	-1,1041781
A_64_P1278	Ppfibp1	4,23E-11	1,12128973	-1,0649961	-1,0539871	1,00614346	-1,1977263	1,01686032	-1,0357161	1,12517419	-1,4953184	1,30413728	1,1649263	1,11041601
A_64_P1287	Mthfd2	3,11E-13	1,09526879	-1,0044928	-1,0752692	1,13532406	-1,4551846	1,31398977	1,25335786	1,19283198	-3,1776261	4,33169018	3,40128916	2,0417627
A_64_P1302	Slitrk3	1,40E-09	1,32808317	1,00669249	-1,0504954	-1,1992432	1,45013076	-1,3138806	-1,0784383	-1,2105528	1,21337806	-2,1100035	-1,6067086	-1,5877048
A_64_P1311	Iqank1	8,39E-16	-1,3118388	1,44227158	1,24028584	1,28278427	1,14736285	1,52134729	2,18082534	1,19310887	3,72184029	1,3936048	-1,1484461	2,2887056
A_64_P1314	Twist2	2,80E-11	-1,1992399	1,81940322	1,02735537	1,00976719	-1,03513	1,81214458	1,12845542	1,22990401	1,90682773	2,98812073	2,03396413	1,31937531
A_64_P1347	Hpn	2,13E-18	-1,2740309	-1,2648935	-1,0341984	1,17304683	1,53348228	1,76146194	1,56422569	2,30907623	5,18455261	1,84982488	1,61676467	1,61833143
A_64_P1351	Msantd1	1,40E-19	1,2103448	-1,1006332	-1,0943102	1,32652329	1,46566513	-1,1323809	-1,0574976	1,01506348	2,48986798	1,48735595	1,43636863	1,04308863
A_64_P1365	Ecel1	5,39E-15	-1,3342249	1,27630071	1,33741962	1,33223537	-1,2515237	1,3373121	1,21312984	1,52236891	2,28707491	1,86120188	1,3525204	1,13279048
A_64_P1374	Bspry	1,12E-12	-1,8346524	1,42969782	1,51109506	1,83433699	-1,0475904	1,09104166	-1,039102	1,10348422	1,10996376	1,12432129	1,58118882	1,2045442
A_64_P1377	Plvap	3,84E-27	1,00544621	1,23708281	1,23221398	1,4128541	1,44432582	-1,0782734	1,04538024	1,03665797	4,37961545	1,71425556	1,59051095	1,09351007
A_64_P138575		1,95E-13	-1,5390752	-1,3317316	-1,250467	1,27718983	1,52908048	1,03264715	-1,4674972	1,22946506	2,54790017	-1,0394252	-1,0368624	2,13903376
A_64_P1394	Plcd4	1,43E-08	1,17368634	-1,0056471	-1,1350991	-1,2028083	1,19646334	-1,0269459	1,31905102	-1,0741804	2,1212288	-1,463618	-1,2090715	-1,3059167
A_64_P1398	Prph	2,06E-11	-1,3664975	1,59675345	1,45598118	1,16513991	1,12962997	-1,0254442	-1,088899	-1,0191487	1,54405779	1,62884176	1,38203898	1,42665751
A_64_P1400	Myo15b	2,11E-25	-1,2786614	1,11300529	1,25017587	1,41293238	1,4116832	1,0860039	1,01144249	1,23111019	3,2823606	1,52302941	1,42039186	1,44408404
A_64_P1400	Tyrp1	6,08E-12	-1,0720096	1,2700323	1,17713451	-1,0592462	-1,284936	1,46654681	-1,2899637	1,40012826	-1,4693247	3,05957966	1,17154705	2,19401077
A_64_P1406	Ccdc84	1,18E-08	1,23674085	-1,171722	-1,0781604	-1,0105043	1,166586	-1,2075928	1,11963148	-1,0155674	-1,5134938	1,0638663	1,39684993	1,30451731
A_64_P1429	Tmprss9	1,99E-29	1,18673214	-1,0248575	1,24095801	1,37414101	2,4669108	-1,3236367	-1,3976333	1,11032287	8,54118416	1,36518191	1,14059586	1,89093792
A_64_P1441	Olig2	3,03E-24	-1,1257766	1,22130662	1,11166999	1,06112986	-1,1862922	1,20437799	1,31432933	1,02619685	-1,4585438	1,29841253	1,51411512	1,30471406
A_64_P1441	RGD1566029	3,76E-16	-1,0678889	1,33857086	1,16383698	1,34528592	-1,059212	1,70298273	3,10468801	1,84312542	3,34582607	1,47391967	1,59396936	1,32498736
A_64_P1454	Ahr	1,56E-08	1,32477757	-1,3283411	-1,1736747	-1,0291542	1,03749879	-1,0645339	-1,2188005	1,01293833	-1,1660839	-1,2027222	1,0294599	1,21492387
A_64_P1469	Cacng2	3,49E-17	-1,0938659	-1,0347834	-1,0424755	1,2786426	-1,0739054	-1,1517719	-1,0037055	1,00636064	1,35463517	1,49253697	1,79789725	1,2795747
A_64_P1471	Fdps	3,24E-11	-1,2363638	1,17946587	1,01877515	1,0601962	-1,0178257	-1,0009388	1,22913971	-1,0172617	-1,2825434	1,14249344	1,60069459	1,11730604
A_64_P1485	Napepld	9,86E-13	-1,187747	-1,0061861	1,47518942	1,01513219	1,70926418	-1,2976592	1,09923921	-1,3834212	2,50498803	-1,0389354	-1,3694763	1,03267422
A_64_P1522	Fscn2	1,31E-26	1,15488559	1,08008066	1,2349884	1,15598432	1,60162944	-1,2950638	-1,1059179	-1,0873981	10,4448633	-1,5910465	-1,7540976	-1,5157755
A_64_P1536	LOC1083482	2,33E-18	1,10746906	-1,1949772	-1,1258747	-1,0575367	1,27673211	-1,0354955	1,14366757	-1,1024345	2,75136152	1,82347169	-1,2213896	-1,3054274
A_64_P154430		1,45E-11	-1,1944937	1,39960946	-1,1463134	-1,6681752	1,00692884	1,03688866	-1,1694824	-1,0607423	1,89466787	1,06077403	-1,7901068	1,08522912
A_64_P1559	Vegfa	8,19E-12	2,04419512	-1,6991046	-1,4480833	-1,145973	-1,165899	-1,1754524	-1,0227388	1,13809977	1,25581421	-2,1562172	-1,2229166	-1,5367485
A_64_P1600	Nxnl2	1,90E-14	1,17076977	-1,2520474	-1,0771289	1,01030692	1,3120758	-1,562737	1,10061789	-1,0492517	-1,8890131	-1,2930385	1,30537764	1,29683488
A_64_P1600	Gpr62	5,39E-22	1,37527549	-1,0322562	1,08554437	-1,0496407	2,08232348	-1,028026	-1,1695638	-1,0605277	4,1466834	-1,4637613	-1,3376544	-1,3322885
A_64_P160373		1,46E-15	1,2040986	1,27041678	1,20221325	-1,0851027	1,47975776	-1,1171018	-1,3637012	1,0685081	6,73264455	-1,6214188	-2,9609496	-1,6319496
A_64_P1612	Zfand2b	5,04E-16	-1,228413	1,19713342	1,16397413	1,13871882	-1,1621553	1,02536519	1,18680758	1,03193151	-1,6660157	-1,1641592	1,19908905	-1,0651409
A_64_P162760		6,48E-09	-1,0583648	1,41929164	1,20713149	1,16065301	-1,1748517	1,30912317	1,20233158	1,27646567	1,75680022	1,06470771	-1,2361976	1,17817397
A_64_P1632	Myom1	5,99E-10	1,30883783	-1,124141	-1,0878611	-1,3368385	1,2076421	1,0194425	1,04091732	-1,0770465	-1,0419948	-1,3509136	-1,2408634	-1,0436
A_64_P1636	Onecut2	9,15E-14	-1,0518346	-1,0133139	-1,0380809	1,20529609	1,15913469	-1,0379912	-1,0541024	1,02027991	-1,3792803	1,50276403	1,71207225	1,22069512
A_64_P1646	Etl4	4,15E-12	1,0149626	-1,4441763	-1,0494699	1,04488187	-1,3259555	-1,0744279	-1,0530158	1,16141331	-1,4053851	-1,5156877	-1,7704537	1,312586
A_64_P1659	Syt6	1,34E-27	-1,0662915	1,10537846	1,31817881	1,19437469	2,8691345	1,29118576	-1,2018202	1,07382996	7,22434905	1,59881897	1,42421366	-1,5831483
A_64_P2377	Rbm24	8,63E-30	1,2878993	-1,0835923	-1,1587397	1,04596286	-1,1877709	-1,5856495	1,03521305	1,05425708	-2,2068517	-1,8853971	1,34669514	1,27385532
A_64_P2818	Nrg1	3,28E-11	-1,0808761	-1,0009976	1,36887246	1,25699555	1,24988241	-1,1440871	1,07422017	-1,5836365	-1,1421558	-1,3197169	-1,4268758	-1,2044983
A_64_P3701	Nrip1	7,78E-10	1,60066245	1,2875694	-1,2000273	-1,6042817	1,45748459	-1,1120672	-1,0575934	-1,6962989	1,18543281	-2,2419039	-2,7225789	-1,8798502

Supplementary Table 6. Lists of *core set* genes at the intersection of apoptosis (K5 vs K25) and GF-mediated neuronal rescue (K5+SP vs K5, K5+PACAP vs K5, K5+IGF1 vs K5)

		0.5 h				1 h				3h			
ProbeName	GeneSymbol.x.x	K5	PACAP	IGF	SP	K5	PACAP	IGF	SP	K5	PACAP	IGF	SP
A_64_P0250	Ccl1	-3,4319472	2,04382811	2,60574541	1,59326014	2,6164724	1,04696165	-1,5466531	-1,2294647	11,8936696	1,42919592	-1,6772904	1,45216189
A_64_P0699	Prss56	-2,2262008	1,5722613	1,4801113	2,23959049	1,62456714	1,55767271	1,51593165	1,64038741	6,66630396	1,46202938	1,49326913	1,36341182
A_43_P1193	Nr4a3	-2,0909356	1,5081482	-1,3001227	-1,2915909	-13,034838	2,14501917	1,59607347	1,11703168	-51,540778	1,40096062	1,31969762	1,65987799
A_42_P4734	Unc13d	-1,9683134	1,84955438	2,10206692	2,02208452	1,42355959	1,13344939	-1,0715609	1,12172222	5,5690394	1,41804528	1,18497104	1,0219405
A_44_P8224	Ctla2a	-1,8757656	1,31629247	1,28574942	1,25942627	1,21041617	1,33372815	-1,0224891	1,32121051	2,09346624	1,53498707	-1,6995177	-1,8189747
A_44_P3280	Masp2	-1,8572012	1,50187287	1,09371557	1,21576964	2,2956637	-1,2442596	1,07474756	1,04025663	5,657664	2,2211087	1,79327656	1,75290608
A_43_P1156	Aqp5	-1,8380563	1,94073983	2,08114166	1,69971978	1,17914654	-1,1294538	-1,0358545	1,09906011	2,78692591	-1,0315794	1,43502771	-1,0500093
A_64_P1374	Bspry	-1,8346524	1,42969782	1,51109506	1,83433699	-1,0475904	1,09104166	-1,039102	1,10348422	1,10996376	1,12432129	1,58118882	1,2045442
A_44_P2655	Crybb3	-1,7590166	1,60376446	1,31183273	1,38947842	1,42409171	-1,1261884	1,11221428	1,07913667	2,04283583	1,19153263	-1,0730936	1,15543225
A_64_P0722	Arrdc2	-1,6469133	1,70074211	1,6546175	1,75662715	1,84216024	1,23587049	-1,1345338	1,25713662	4,37634599	-1,3075229	-2,586977	-1,1839277
A_44_P3748	Klk1c3	-1,6252583	1,39338454	1,61457567	1,30420818	1,67298541	-1,1287524	1,86583943	-1,0686384	3,61253847	2,11095665	1,95214883	1,93938852
A_64_P1004	Hoxd9	-1,5803312	1,25698433	1,07490119	1,72266646	-1,0942638	1,44106413	2,61881775	1,35714209	3,17759487	3,17197412	-1,4378832	2,26509852
A_44_P5017	Crybg2	-1,4592009	1,10934798	1,53974445	1,17158627	1,31496925	-1,0569955	1,33905319	1,13705715	2,76866743	1,74332917	1,19714641	1,48898591
A_44_P4283	Hmgcs2	-1,4375247	1,40225664	1,30038992	1,12360805	1,12405389	1,58840829	1,80449323	1,39822884	4,27882166	-3,8085226	-2,8130715	-2,2274658
A_44_P8763	Vav2	-1,412457	1,20337757	1,32026642	1,33140001	-1,1810569	-1,1212876	-1,279889	1,02883819	2,44500175	1,91064321	1,66118414	1,36778484
A_42_P5367	Sftpc	-1,4081882	1,56992879	1,52410229	1,19133218	2,95506575	1,14127817	-1,0888162	-1,3647872	6,83282245	1,45210029	-1,5242111	-1,3416618
A_64_P1398	Prph	-1,3664975	1,59675345	1,45598118	1,16513991	1,12962997	-1,0254442	-1,088899	-1,0191487	1,54405779	1,62884176	1,38203898	1,42665751
A_43_P2281	Cd6	-1,3504958	1,52227154	1,54996846	1,66009293	2,32209185	-1,7544367	-1,5603697	-1,1408352	13,371904	-1,021495	-1,745929	1,06669321
A_64_P0617	Fam89a	-1,3402672	1,16309384	-1,0970199	2,1001264	-1,0781758	1,26483584	1,11271525	3,16468639	-1,3818886	1,54920995	1,79069903	3,7090714
A_64_P1365	Ecel1	-1,3342249	1,27630071	1,33741962	1,33223537	-1,2515237	1,3373121	1,21312984	1,52236891	2,28707491	1,86120188	1,3525204	1,13279048
A_42_P6868	Siglec1	-1,3184952	1,54418244	1,28585906	1,0728878	-1,1646284	1,35249671	-1,3459918	1,17735514	-1,3737382	1,30203818	1,14564642	1,41408777
A_64_P0436	Slc39a5	-1,3135569	1,12826503	1,40738175	1,88096609	1,43141499	-1,1469931	-1,0040344	1,46785675	3,00584851	1,74622335	1,86287116	2,70413167
A_64_P1311	Iqank1	-1,3118388	1,44227158	1,24028584	1,28278427	1,14736285	1,52134729	2,18082534	1,19310887	3,72184029	1,3936048	-1,1484461	2,2887056
A_64_P0323	Mustn1	-1,2799385	1,20203068	1,17812569	1,02482029	1,21042037	-1,0567997	1,15454851	1,07958073	1,42497253	-1,6624184	-1,3747209	-1,0937989
A_44_P1012	Stbd1	-1,2790927	1,64837193	1,34454413	1,14719467	-1,218187	1,17865499	-1,1128397	1,18343794	1,13120563	1,79855508	1,00323372	1,21998483
A_64_P1400	Myo15b	-1,2786614	1,11300529	1,25017587	1,41293238	1,4116832	1,0860039	1,01144249	1,23111019	3,2823606	1,52302941	1,42039186	1,44408404
A_64_P0925	Sspo	-1,2765665	1,09665741	1,52235104	1,32516459	1,41539091	-1,1659368	-1,1920023	1,19811579	4,6228209	1,73693733	1,18975853	1,50984735
A_64_P0220	Sstr2	-1,2704406	1,29135566	1,45296308	2,00157708	1,60068189	2,1885859	-1,132779	1,28107473	5,6176739	4,05156223	1,65532822	1,05752444
A_44_P1039	Rasip1	-1,2684579	1,03879014	1,21247344	1,22618372	1,01991687	1,12934354	-1,0420739	1,22779462	1,67105423	1,31899665	1,39608328	1,149626
A_64_P0534	Bag2	-1,2593605	1,06920928	1,09947357	1,25706919	-1,1239787	1,04604955	1,23245463	1,16349757	-2,8980397	-1,8806881	-1,0206516	1,26991884
A_64_P0305	Tpcn2	-1,2486358	1,21447281	1,3734151	1,20337648	1,43844438	1,05098863	-1,0318985	1,1838273	3,53257159	1,41950084	1,47903798	1,17319921
A_64_P0012	Mthfs	-1,2448475	1,31191655	1,22573374	1,20731225	-1,0109596	1,10006934	1,13798702	1,06568277	1,42473707	1,19338435	-1,2562283	1,19637169
A_64_P0346	Rpain	-1,2435641	1,1139835	1,15195046	1,16974739	1,00821505	1,00610672	1,1037578	1,01626494	1,27545773	-1,2224778	-1,4327986	-1,1240168
A_43_P1144	Atp4b	-1,2430364	1,3927677	1,70536941	1,51613523	1,83586982	-1,7257548	-1,6882044	-1,6026268	8,26700323	1,03892701	-1,9383347	1,10194269
A_64_P1471	Fdps	-1,2363638	1,17946587	1,01877515	1,0601962	-1,0178257	-1,0009388	1,22913971	-1,0172617	-1,2825434	1,14249344	1,60069459	1,11730604
A_64_P1612	Zfand2b	-1,228413	1,19713342	1,16397413	1,13871882	-1,1621553	1,02536519	1,18680758	1,03193151	-1,6660157	-1,1641592	1,19908905	-1,0651409
A_44_P4504	Ttll1	-1,214121	1,05730031	1,09335219	1,03624174	1,10370381	1,15160575	1,24015179	-1,0000542	-1,1729407	-1,2385477	-1,5107105	-1,3799532
A_44_P4305	Ntrk1	-1,210288	1,44402726	1,47396222	1,44192507	1,5363989	-1,1404822	-1,5063945	1,03647527	5,68274173	1,65025789	1,35699114	1,47864559
A_44_P1314	Twist2	-1,1992399	1,81940322	1,02735537	1,00976719	-1,03513	1,81214458	1,12845542	1,22990401	1,90682773	2,98812073	2,03396413	1,31937531
A_44_P1987	H1f0	-1,1851964	1,30009479	1,31805913	1,18069604	1,63279515	-1,0639567	-1,3653199	-1,0239035	2,88354299	1,36954845	1,09507659	1,33376507
A_44_P2480	Thbs2	-1,1777825	1,41447675	1,27346004	1,05952379	-1,0893103	1,04309282	-1,1987487	1,01302069	1,12823528	1,17537426	-1,0915428	1,25098114
A_44_P1099	Ahsa2	-1,1738938	1,09079666	-1,0580268	1,09097892	-1,0455755	1,25434076	1,10810037	1,05961659	-1,8291799	1,18595363	1,66744639	1,32949715
A_42_P7183	Sstr3	-1,1722252	1,19914989	1,39159317	1,30004215	2,44261491	1,19338849	1,19416934	1,14064481	6,66084034	-1,2358069	1,35239548	-1,012538
A_44_P5322	Fkbp5	-1,1639663	1,19736946	1,20328688	1,4030669	-1,2357626	-1,0295866	-1,1425246	1,04616935	-1,3270833	1,35873654	1,4592649	1,19791741
A_42_P5470	Pak4	-1,1607371	1,2055466	1,11530591	1,12462059	-1,1218131	1,05137724	1,1246795	1,08776811	1,0786148	-1,0078112	1,43830009	1,2105719
A_44_P6131	Ndp	-1,1449877	1,05341591	1,08262155	-1,2564203	1,44088148	-1,0931937	-1,2076398	-1,0752197	2,83997616	-1,5354486	-1,7467033	-1,3094729
A_64_P0883	Sytl1	-1,1421212	1,15269705	1,22873951	1,14948761	1,2313567	-1,1056692	1,07775954	1,15426512	1,78965885	-1,0971477	1,26225034	1,20790548
A_64_P0878	St14	-1,1364494	1,23345232	1,1256745	1,15644585	1,63425103	-1,3546345	-1,021122	-1,0456242	3,82205013	-1,875708	-2,040859	-1,4430076
A_64_P0335	Tep1	-1,1329069	1,03475638	1,09262408	1,20357128	1,11432173	-1,1384877	-1,3424642	1,057625	1,66714964	-1,221011	1,02996608	1,27443311
A_43_P1570	Rab13	-1,1312491	1,21277274	1,04744536	-1,05626	1,0441529	1,11286908	1,14732312	-1,0421881	1,26490273	-1,1476537	-1,1817036	-1,5695434
A_64_P1163	Adam33	-1,1291141	1,17352284	1,17036488	1,12611826	1,2655733	-1,0075157	-1,2778431	1,09283202	-2,3851941	2,2176918	1,7711376	3,01234692
A_64_P1057	Ddx25	-1,1279104	1,18401561	1,10674837	1,25107872	1,13183319	1,0285718	1,07467837	1,0234493	1,45626103	1,21164279	1,24967938	1,10099016
A_64_P0430	Cbr3	-1,1268118	1,04006118	1,07058673	1,26378279	-1,1169983	1,59326366	1,31048186	1,17313543	-1,4780422	1,44973023	1,0081983	-1,165129
A_64_P1441	Olig2	-1,1257766	1,22130662	1,11166999	1,06112986	-1,1862922	1,20437799	1,31432933	1,02619685	-1,4585438	1,29841253	1,51411512	1,30471406
A_64_P0975	Mtus1	-1,1158226	1,11913515	-1,0071697	-1,103449	1,10302586	1,00457356	1,1551473	-1,068508	1,26242181	-1,6566985	-2,7337581	-1,2697359
A_64_P1204	Cacng6	-1,1155177	1,01151838	1,01528587	1,04565358	-1,1839897	1,08601153	1,26309189	1,2304462	-1,6079355	-2,433313	-1,3483611	1,44229149
A_42_P4942	Armc9	-1,1083207	1,05147837	1,00755796	-1,0267129	-1,1111632	1,08717253	1,11866058	1,02124583	-1,3825815	-1,0939087	-1,0765886	-1,1725349
A_64_P0480	Bfsp2	-1,1053594	1,15119817	-1,2782361	-1,1639851	-1,1195363	1,12368428	1,77081102	1,33555904	1,84490186	-1,2203169	-1,0488283	-1,3993302
A_64_P0097	Btbd17	-1,1028507	1,30473386	1,35005825	1,16826621	2,81475041	1,31137658	1,00285561	-1,0734823	17,3583366	1,14233239	-1,2491403	-1,3988079
A_64_P0617	Trpv1	-1,0976444	1,30593725	1,14223404	1,25067748	2,51041897	-1,8949162	-1,5219704	-1,8277417	4,6537179	1,19264113	1,17667435	1,38012534
A_64_P0650	P3h4	-1,0916582	1,38924114	1,255036	-1,0020248	1,15302236	-1,0971537	-1,0851732	-1,1954735	1,72329322	1,00616025	-1,1617248	-1,1569921
A_44_P3163	Shkbp1	-1,0892705	1,168664	1,18666658	1,20444189	1,24563389	1,08330853	1,03242805	1,22533871	1,76663315	1,27087969	1,40694786	1,28216401
A_64_P0244	Stum	-1,0868412	1,06827823	1,10713583	1,02667045	1,18078785	-1,3803738	1,22336937	-1,1231698	2,58592053	-1,2916815	-1,1530551	-1,4571295
A_44_P3819	Sphk1	-1,0866784	1,47210798	1,32307178	1,07700506	1,37865398	1,67773936	1,01955606	1,23712096	2,94847345	-1,0421029	-1,3358165	-1,0454515
A_64_P0999	Dap	-1,083706	1,33171974	1,11830301	-1,072773	-1,0454749	1,12664953	1,04164799	1,03740432	1,81748745	-1,0506708	-1,3092625	-1,2259214
A_42_P6739	Mrnip	-1,0832755	1,01830099	1,09626874	1,00797365	1,13166542	-1,0719505	1,01803716	1,00552312	2,12078592	-1,2538152	-2,1229098	-1,2689879
A_64_P0293	F8a1	-1,0808753	1,04061814	1,04593062	1,13516451	-1,0630015	1,0493439	1,1254379	1,04571924	-1,1459514	-1,1991152	1,01189147	-1,0613326
A_64_P1400	Tyrp1	-1,0720096	1,2700323	1,17713451	-1,0592462	-1,284936	1,46654681	-1,2899637	1,40012826	-1,4693247	3,05957966	1,17154705	2,19401077
A_64_P0043	Pipox	-1,0706266	-1,007414	-1,0225117	-1,3437713	2,36032387	-1,4435341	-1,0978958	-1,179013	3,95163802	-1,2888297	-1,6891947	-1,1567814
A_44_P1924	Dusp9	-1,0678188	1,16777158	1,45717313	1,46861353	4,45686314	1,12224083	1,11147673	1,03789372	5,72570245	1,86879096	1,75492454	1,31496886
A_64_P1659	Syt6	-1,0662915	1,10537846	1,31817881	1,19437469	2,8691345	1,29118576	-1,2018202	1,07382996	7,22434905	1,59881897	1,42421366	-1,5831483
A_64_P0693	Scn10a	-1,0578109	-1,0646817	1,21841306	1,34501205	1,355231	-1,4522463	-1,8616723	-1,3213172	5,0626872	1,42059765	1,05451192	1,10558404
A_64_P4774	Slc2a4	-1,0561596	1,13244911	1,05109293	1,06567273	1,12162889	-1,0343684	-1,0273455	1,18408997	1,97499496	-1,4896613	-1,2591111	1,35587965
A_64_P1015	Sec63	-1,0539689	-1,0009305	1,02672067	1,08579663	-1,3960632	-1,0934897	1,05271987	-1,0094299	-1,8927015	1,21598865	1,27231275	1,19396579
A_64_P1636	Onecut2	-1,0518346	-1,0133139	-1,0380809	1,20529609	1,15913469	-1,0379912	-1,0541024	1,02027991	-1,3792803	1,50276403	1,71207225	1,22069512
A_64_P2532	Adamts9	-1,0510509	1,07163773	-1,0559312	1,00069383	-1,0798354	1,06111609	1,12621374	-1,0408909	-1,4154181	1,33130014	1,41271617	1,27444914
A_64_P0444	Mrpl50	-1,044671	1,20207112	-1,0448086	-1,1007575	2,22017366	-1,2018572	-1,247634	-1,6525394	3,31293898	-1,3568767	-1,4708166	-1,1139833
A_44_P4741	Slc7a1	-1,0362265	1,06234501	1,06790111	1,1819656	-1,3305081	1,1041838	-1,0826277	1,14415199	-1,4761583	2,20455818	1,45978373	1,12753278
A_64_P0690	Lpar6	-1,0224703	1,10956634	1,25272804	1,20215256	-1,1829231	-1,1643143	1,09962412	1,32104099	1,41689772	-1,1768274	-1,1771621	-1,0816417
A_64_P0580	Rbp4	-1,0177956	1,06317463	1,21120838	1,33852743	1,22972973	-1,1781124	-1,2237111	-1,0096249	1,92237808	1,13686685	-1,2423958	1,06207283
A_64_P0500	Pde9a	-1,0176623	-1,0408459	-1,1071051	1,10720627	-1,0242775	1,06357659	1,05594938	1,12714359	3,45160916	1,4058988	1,34789214	1,1674669
A_64_P0541	Nudt6	-1,0147498	-1,1359089	-1,0572245	1,07793855	1,74929304	-1,1215318	-1,5733863	1,00459249	3,28001543	-1,2681623	-1,6113981	-1,4062432
A_44_P3756	Rbm11	-1,0111093	-1,1740494	-1,1131473	-1,4298624	4,0092092	-1,2913437	-1,0463166	-1,2262591	6,32184253	-1,3263928	-2,0388884	-1,1356724
A_44_P5004	Lman1	-1,0066081	1,02504811	1,05255253	1,03694393	1,09529539	-1,0906926	-1,0205686	-1,0045253	-1,3023399	1,20856718	1,269945	1,16850235
A_64_P0093	Reep4	-1,0058519	1,12644119	1,14766996	1,274795	1,36693706	1,18336969	1,09989937	1,05557086	1,80898687	1,42839126	1,39656624	1,18930784
A_42_P5675	Traf4	-1,0055682	1,01300387	-1,0362928	-1,2747489	-1,5300782	1,44424311	1,29768578	1,05510712	-2,0713376	1,22417236	1,56982724	-1,1434946
A_43_P1554	Gabra6	1,00233643	-1,1336747	-1,2061542	-1,363236	1,27300555	-1,0249289	-1,1643251	-1,2242595	-1,2821861	-1,4704045	-1,1273768	-1,1234546
A_64_P1377	Plvap	1,00544621	1,23708281	1,23221398	1,4128541	1,44432582	-1,0782734	1,04538024	1,03665797	4,37961545	1,71425556	1,59051095	1,09351007
A_42_P5064	Slc25a25	1,00593257	1,01651303	-1,0500541	1,20674196	-1,4163981	-1,0147405	1,17942435	1,19346206	-1,8889274	1,22710019	1,3790065	1,28243538
A_44_P4305	Nsmce4a	1,02691895	-1,154572	-1,0623145	-1,1043298	1,26667075	-1,0345491	-1,0081796	-1,19243	-1,2076018	-1,1507451	-1,2905753	-1,0968929
A_64_P1240	Ntsr1	1,03163856	-1,1850875	1,29051261	1,30718268	1,36743005	-1,3194139	-1,1704378	-1,1062526	8,96517944	1,52518379	1,33419156	1,18565411
A_44_P9993	Lmbr1l	1,03321276	1,02325651	-1,1749949	-1,0184356	-1,8286847	1,28505247	1,27484725	1,42077502	-1,5258627	-1,249976	-1,982807	-1,0221638
A_64_P0023	Zbed4	1,0370189	-1,13419	-1,2864737	-1,0606336	-1,0663903	1,01934994	1,13738792	-1,0091652	-2,8066728	1,34562355	2,15755083	1,32024437
A_42_P8023	Fam110b	1,04957882	1,07097328	1,09887413	-1,052367	1,23292693	-1,1584145	1,08241967	-1,1089518	2,36812358	-1,3515576	-1,3222503	-1,2596648
A_44_P2737	Masp1	1,05608664	-1,0554758	-1,3017411	1,06370843	1,18210254	1,14796958	-1,0753328	1,12515104	-1,735803	2,96385387	1,93029467	2,49816713
A_64_P1105	Spdef	1,05628387	-1,4786778	1,30980536	1,2862954	1,05107	-1,2963235	-1,1985514	-1,2142584	1,91175864	-1,4712474	1,00049839	-1,2576997
A_64_P0095	Vmo1	1,06327378	1,02439385	1,06497292	1,18539531	1,91461179	-1,4210157	-1,2501574	-1,1817896	5,3143583	-1,0007657	-1,4886623	1,18895425
A_64_P1105	Plekhf1	1,06661402	-1,0721828	-1,0646748	-1,2399642	-1,6156416	-1,0606334	1,24473311	-1,0192918	-1,4268065	-2,0418832	-1,6193529	-1,1939069
A_44_P3116	Ahnak	1,06682848	1,23325237	1,18108401	1,02231132	-1,2308755	1,16126649	1,04925018	1,21142635	1,43924748	-1,3613213	-1,4121647	-1,3955062
A_44_P4913	Nudt7	1,06757273	-1,0450115	1,02877444	-1,0954304	1,37016353	-1,1381143	-1,0754422	-1,1648992	2,71775706	-1,4002486	-1,5901133	-1,2411166
A_64_P0387	Aste1	1,08386186	1,048959	-1,0134821	1,14315851	-1,3775343	1,0977507	1,06135477	1,13464573	-2,7254446	1,26007706	1,40008313	1,1825626
A_42_P6825	Nr4a1	1,08714639	2,08413458	-1,0568255	-1,0534911	-6,2555637	4,48161681	1,63414217	2,27622525	-15,254276	1,70030517	-1,1291646	2,29671222
A_64_P1287	Mthfd2	1,09526879	-1,0044928	-1,0752692	1,13532406	-1,4551846	1,31398977	1,25335786	1,19283198	-3,1776261	4,33169018	3,40128916	2,0417627

A_44_P1024	Hdac5	1,09687609	-1,0407354	-1,1293911	-1,1682206	-1,3948917	1,16270372	1,34329396	1,13574995	-2,7848247	-1,512149	-1,7032169	-1,1114303
A_44_P9175	Mat2b	1,09966482	-1,2167165	-1,078615	-1,1760397	-1,0194229	-1,0930277	-1,0556938	-1,143156	-1,6334545	-1,580013	-1,9036486	-1,2514093
A_44_P6393	Col24a1	1,10760624	-1,1612311	-1,0024071	1,19281039	1,38257363	-1,2435888	-1,2420131	1,10208067	-1,5102983	1,12403075	1,23870223	1,73575084
A_42_P5887	Mthfd2	1,11841787	1,09301842	-1,0242211	1,14446847	-1,0741379	1,0445351	1,2389173	1,09651841	-2,0007078	3,46777576	2,15858651	1,06654819
A_43_P1258	Nfyb	1,12121904	1,02733813	-1,0218058	-1,0822666	1,25269201	-1,0841017	-1,0754081	-1,1126049	1,27319364	1,17124883	1,09878587	-1,2237311
A_64_P1278	Ppfibp1	1,12128973	-1,0649961	-1,0539871	1,00614346	-1,1977263	1,01686032	-1,0357161	1,12517419	-1,4953184	1,30413728	1,1649263	1,11041601
A_44_P2364	Lnx1	1,1421257	-1,1064154	-1,0637329	-1,2054283	1,25514987	-1,0559388	-1,084018	-1,163722	-1,2988639	-1,5562637	-1,6183762	-1,1406835
A_44_P1011	Znhit6	1,14480565	-1,3224645	-1,178913	-1,0915873	1,02545737	-1,0722884	-1,3821728	-1,0249676	-2,4962699	1,20255751	1,52190354	1,30430593
A_44_P1015	Slc22a3	1,14499022	-1,4661555	-1,0220595	-1,2373934	1,47476846	-1,2308399	-1,3380894	-1,2424003	2,44147985	1,61099777	1,44181604	1,54221296
A_64_P1522	Fscn2	1,15488559	1,08008066	1,2349884	1,15598432	1,60162944	-1,2950638	-1,1059179	-1,0873981	10,4448633	-1,5910465	-1,7540976	-1,5157755
A_44_P3550	Adssl1	1,15510434	-1,3081959	1,035647	1,23482738	-1,1458741	-1,0171381	-1,2650787	1,09840888	1,39977601	-1,1393101	-1,1618888	-1,1282792
A_44_P5086	Tm6sf2	1,15561529	-1,0872373	1,08049246	1,26935595	1,57086648	-1,0453905	-1,0282656	1,18606056	2,41863437	1,28382964	1,24559363	1,1945617
A_64_P1260	Slc22a7	1,15651305	-1,4478503	-1,2068375	-1,0703001	2,02545552	-1,3758419	-1,178344	-1,5703695	1,45898112	1,27292287	1,86498247	1,77692042
A_64_P1273	Sys1	1,16893489	-1,0190439	-1,0950932	-1,2676504	-1,0720715	-1,1648591	1,20167466	-1,2206377	-1,5798618	-1,1616524	-1,0383298	-1,1041781
A_44_P7298	Ppp1r3d	1,16894542	-1,0358389	1,03656194	1,02726089	1,53221685	-1,1819223	1,24951272	-1,1359122	-1,0372114	1,05022496	1,57772016	1,16924093
A_64_P1394	Plcd4	1,17368634	-1,0056471	-1,1350991	-1,2028083	1,19646334	-1,0269459	1,31905102	-1,0741804	2,1212288	-1,463618	-1,2090715	-1,3059167
A_43_P1857	Tekt4	1,18321578	1,07820524	1,05973339	1,02059028	2,42737079	-1,4748629	-1,3402284	-1,0402614	2,49596882	-1,3868668	-1,3400636	-1,2657618
A_42_P5484	Acot1	1,18861001	-1,1241586	-1,04697	-1,096765	1,33561555	-1,1153939	1,03475663	-1,1527721	2,15731723	-1,4248677	-1,5713753	-1,8019999
A_64_P0718	Gpr143	1,19653927	-1,1855934	-1,259565	-1,4033859	1,2470589	-1,0098328	1,00068854	1,21418523	1,34995875	1,1825119	1,00024221	1,32412982
A_64_P0437	Fat4	1,20203148	-1,2423262	-1,3101595	-1,0662886	-1,1961613	-1,0232123	1,19820856	1,02876279	-3,1146067	1,18630422	1,27016445	1,21947829
A_64_P0867	Runx1t1	1,21555898	-1,0196397	-1,0418479	-1,2463267	1,2529529	-1,1134038	-1,1104528	-1,3249388	-1,2455218	-1,3457434	-1,5175862	-1,1296584
A_44_P3540	Hist1h1a	1,21619565	1,22006547	1,0290557	1,16580248	3,06787427	-1,0000292	1,0326201	-1,0519379	3,62477891	-1,6142726	-1,6903075	-1,5391572
A_64_P0839	Zfp68	1,22593673	-1,229354	-1,3219726	-1,3183106	-1,1636506	-1,1114576	1,02695553	-1,2004054	-1,7859217	-1,1662486	-1,103356	-1,2770575
A_42_P5012	Adra1d	1,23392339	-1,1149746	-1,0346297	-1,0223727	-1,1455338	-1,2247693	-1,0526608	1,01386522	-3,7348388	1,38582434	1,37322665	1,34458774
A_44_P6385	Paqr5	1,23429569	-1,2246629	-1,0036912	-1,1579873	1,71565502	-1,7483497	-2,0524758	-1,7751185	10,0257196	1,11865681	1,40027948	-1,52074
A_64_P1406	Ccdc84	1,23674085	-1,171722	-1,0781604	-1,0105043	1,166586	-1,2075928	1,11963148	-1,0155674	-1,5134938	1,0638663	1,39684993	1,30451731
A_43_P1010	Spry4	1,24440278	1,15193314	-1,3194328	1,09397248	-1,3889774	1,89760679	1,12541614	-1,0449441	-7,9117163	2,74256288	2,39502464	2,08828975
A_44_P2240	Kcna4	1,25321207	-1,1535717	-1,2153844	-1,4468859	-1,2138716	-1,0175226	1,07970233	-1,1748579	-5,9124241	-2,4333604	-1,9094817	1,02584531
A_64_P0422	Acvr1c	1,26126666	-1,0240169	-1,2052253	-1,3710351	1,11470631	-1,1534384	1,28305066	-1,2119314	-1,7704528	-2,2489728	-2,0227882	-1,414374
A_64_P1035	Nmt2	1,26856816	-1,0179161	-1,0713811	-1,0155389	1,06631749	-1,0833563	-1,1961218	-1,0993828	-1,1210614	-1,2258643	-1,0718023	-1,2128057
A_44_P4427	Kcnab1	1,2764726	-1,0409345	-1,3269391	-1,2687251	1,32605666	-1,0923047	1,1185382	-1,4348933	-1,2840445	-2,3844049	-1,7917698	-1,3249073
A_44_P4580	Tnfrsf11b	1,27902416	-1,2255886	-1,0052575	-1,1337174	1,25016335	-1,355091	-1,5751336	1,21824341	1,46367769	-1,8671614	-1,3698884	-1,4535612
A_42_P8282	Lgr4	1,28231733	1,08517775	-1,1133993	-1,2124438	1,12842997	-1,1782765	-1,1155999	-1,2388307	-1,1063061	-1,2850823	-1,2296385	-1,0759889
A_42_P8428	Reep6	1,28517257	-1,1996526	-1,0617553	1,07616823	1,00841367	-1,3368749	-1,1966863	-1,1567664	-1,0399129	1,09707125	1,42138182	1,14206719
A_42_P4847	Ctgf	1,29623908	-1,0326849	-1,1410669	-1,1551109	-1,085141	-1,0501954	-1,0886356	1,87886019	1,04272173	-2,0973893	-1,3227585	1,69950677
A_64_P0750	Camk1g	1,2972911	-1,0980365	-1,1315249	-1,3184658	-1,0504847	1,40373229	1,38735398	1,10153277	-1,4111668	1,79477109	-1,2528243	-1,1266949
A_44_P2435	Pxdc1	1,30119068	-1,2800953	-1,0750086	-1,0950985	1,34728307	1,05563279	-1,002042	1,03895289	1,4240923	-1,5290905	-1,7008558	-1,3129923
A_44_P1047	Herpud1	1,30511428	-1,1112784	-1,237013	1,03181964	-1,1227343	-1,0252906	1,06431673	-1,100954	-1,1048727	3,38054942	1,27802658	1,03236655
A_44_P3284	Rprd1a	1,30713899	-1,1753999	-1,1365014	-1,2875824	1,12668062	-1,2893551	-1,243783	-1,3613815	-1,9549251	-1,2315065	-1,3339798	-1,2874555
A_64_P1632	Myom1	1,30883783	-1,124141	-1,0878611	-1,3368385	1,2076421	1,0194425	1,04091732	-1,0770465	-1,0419948	-1,3509136	-1,2408634	-1,0436
A_44_P8984	Lrrc17	1,31104668	-1,1597586	-1,2442724	-1,3281254	1,60031757	-1,186434	-1,2784178	-1,202399	1,40828019	1,32557987	-1,3722467	-1,4368855
A_64_P0366	Klf6	1,31423221	-1,2399267	-1,0372632	-1,6945553	-1,8741033	-1,6789367	-1,1979892	1,06608421	-1,6073773	-1,2365219	-1,5905002	1,03272966
A_64_P1454	Ahr	1,32477757	-1,3283411	-1,1736747	-1,0291542	1,03749879	-1,0645339	-1,2188005	1,01293833	-1,1660839	-1,2027222	1,0294599	1,21492387
A_64_P1302	Slitrk3	1,32808317	1,00669249	-1,0504954	-1,1992432	1,45013076	-1,3138806	-1,0784383	-1,2105528	1,21337806	-2,1100035	-1,6067086	-1,5877048
A_44_P6285	Rgs7bp	1,37453303	-1,167957	-1,285232	-1,406927	1,01881068	1,04678381	1,02035595	-1,1324274	-1,5521996	-1,3748133	-1,6378483	-1,2781215
A_64_P1600	Gpr62	1,37527549	-1,0322562	1,08554437	-1,0496407	2,08232348	-1,028026	-1,1695638	-1,0605277	4,1466834	-1,4637613	-1,3376544	-1,3322885
A_64_P0950	Mmd2	1,38465051	-1,2031431	-1,204039	-1,1722035	1,13008379	-1,0560346	1,25923136	-1,0480237	1,3994537	1,04915437	1,01049478	-1,3505262
A_64_P1043	Crb3	1,38732326	1,04938813	-1,0242776	-1,3031397	1,73702379	-1,3268065	-1,3181637	-1,2015023	2,55673572	1,24397147	-1,182712	-1,0957118
A_64_P0487	Cbs	1,41626073	-1,1739135	-1,1480469	-1,3703295	1,03617616	1,29811171	1,58092833	1,04831312	-1,0465422	1,78001237	-1,0648407	-1,4835554
A_44_P1151	Slc5a5	1,4176546	1,18753613	1,24238629	-1,168869	1,48821175	-1,3904438	-1,2764685	-1,6103675	1,41609983	1,74526517	1,08192984	1,35796467
A_44_P5133	Dab2	1,41820655	-1,0105748	-1,1256271	-1,3682357	-1,0781318	1,25383962	-1,0841686	1,06513924	1,0949432	1,14476062	-1,2701474	-1,3968844
A_44_P2933	Bbs4	1,44463934	-1,1474814	-1,2318744	-1,3696193	1,10189445	-1,0660558	-1,1764213	-1,1627537	-1,3721333	-1,3770016	-1,4194896	-1,373086
A_64_P0883	Hbb-b1	1,44932375	-2,1558591	-1,4800532	-1,7810121	-1,0464846	1,39306232	2,36828931	1,2594784	2,40481648	-1,5059028	1,32749764	-1,5853268
A_43_P1104	G0s2	1,45487274	-1,0066303	-1,1897788	-1,0612278	1,27256867	1,024339	1,32997808	1,107839	-2,4635727	1,73254809	1,75683926	1,37658882
A_44_P8473	Luzp2	1,48033695	-1,1643918	-1,4316401	-1,4236621	1,33501641	-1,1196391	-1,0164186	-1,2386817	-1,1894122	-1,7770831	-1,5671785	-1,4199932
A_64_P0251	Cebpb	1,49391748	1,07669214	-1,2076377	-1,3125046	-1,105283	1,62128724	1,38692771	1,09941034	1,00376573	1,95145863	1,15820718	-1,0227668
A_44_P2312	Insig2	1,49739372	-1,2365742	-1,2276626	-1,3775849	1,24373943	-1,1088589	1,03453461	-1,2619333	-1,1164049	-1,436384	-1,7438117	-1,6618249
A_44_P3787	Flrt3	1,50656825	-1,003344	-1,1952993	-1,4826594	-1,1440401	-1,5305841	1,07234795	-1,0926958	-1,4810881	-1,2613267	-1,3310811	-1,1315197
A_43_P1393	Grem2	1,52537998	-1,2891402	-1,078909	-1,1435238	1,43889634	-1,4062546	-1,0221239	-1,2248009	1,88814996	-1,334074	-1,9261231	-1,4123422
A_43_P2111	Klhl24	1,54220305	-1,0578358	-1,2113148	-1,5808417	1,22905115	-1,0186945	-1,1558027	-1,4461355	1,3958385	-1,6571497	-3,3169164	-1,5948134
A_43_P1168	Id2	1,57473862	-1,0524717	-1,1512629	-1,1231553	1,41068378	1,32114404	1,28965044	-1,0351066	2,68718912	1,06417472	-1,0271368	-1,46832
A_64_P0883	Angptl4	1,59197992	-1,1310105	1,02519337	1,15717901	1,3828939	-1,6454362	-1,1298279	1,11798814	3,78910889	-3,4574484	-2,4405958	-1,9782349
A_64_P3701	Nrip1	1,60066245	1,2875694	-1,2000273	-1,6042817	1,45748459	-1,1120672	-1,5075394	-1,6962989	1,18543281	-2,2419039	-2,7225789	-1,8798502
A_64_P0883	Hbb-b1	1,60407514	-2,2786047	-1,3809397	-1,7511953	-1,0163249	1,62928354	3,26769824	1,2779441	2,85582742	-1,5001236	1,21632756	-1,960272
A_44_P2461	Tle1	1,62376929	-1,1038729	-1,2182448	-1,5046985	1,20467609	-1,1949365	-1,2709147	-1,3476928	-1,3762078	-1,4812348	-1,4612904	-1,2995969
A_44_P1005	Aoc3	1,68659331	-1,1388179	-1,1589152	1,28359761	2,63908142	-1,6178195	-1,7782275	-1,1977132	5,76880281	1,85171061	1,87550089	1,65887048
A_44_P4003	Maf	1,6915538	-1,3185942	-1,2380278	-1,4285274	1,16160517	-1,2225225	-1,1488643	-1,1694517	-1,2020259	-1,2915682	-1,4967731	-1,2276107
A_42_P6367	Gzmm	1,71794387	-1,245285	-1,0256451	1,07642392	1,52144548	-1,1656878	-1,2862662	-1,0364348	2,32879208	1,26998301	1,49966639	1,41960655
A_44_P1871	C1galt1	1,72177265	-1,2964562	-1,412515	-1,2159937	1,14406228	-1,3663571	1,35032134	-1,1880564	-1,9400543	-1,6737045	-1,5084621	-1,8033365
A_44_P4480	Vpreb3	1,75336014	-1,0638533	1,02192932	1,04302422	2,04230934	-1,5894075	-1,4627488	-1,3618484	2,85489459	-1,0030443	1,09160102	1,14345109
A_44_P1050	C1qtnf12	1,76478789	-1,2102004	-1,0181924	1,30993839	1,75434402	-1,4085148	-1,1271507	-1,0691744	1,78537845	1,52981297	2,2739407	1,606742
A_64_P0765	Jph1	1,80182007	-1,4631127	-1,3953079	-1,5818631	1,45268981	-1,2802188	-1,1902427	-1,2999841	-1,7499977	-1,699297	-1,2250332	1,00595495
A_42_P5476	Fam49a	1,9248158	-1,3898281	-1,3850747	-1,1646081	1,13361807	-1,2552141	1,21163147	-1,1713241	-1,6767022	-1,5668711	-1,5601939	-1,29092
A_64_P1559	Vegfa	2,04419512	-1,6991046	-1,4480833	-1,145973	-1,165899	-1,1754524	-1,0227388	1,13809977	1,25581421	-2,1562172	-1,2229166	-1,5367485

Supplementary Table 7: The results of upstream regulator analysis from up-regulated *core set* genes based on iRegulon.

Motif id	AUC	NES	ClusterCode	Transcriptior	Target genes
homer-M001	0.129017	560.781	M1	Nf1,Nfic,Nfia	Masp1,Fat4,Sftpc,Adam33,Bspry,Aqp5,Slc25a25,Spry4
hdpi-CCDC25	0.107354	444.514	M2	Ccdc25,Pax7,	Ecel1,Spry4,Nr4a3,Onecut2,Twist2,Ntrk1
transfac_pub	0.107354	444.514	M3	Ovol1,Ovol2	Ctla2a,Sec63,Onecut2,Nr4a3,Olig2,Twist2
elemento-AA	0.106736	441.197	M4		Sstr3,Spry4,Ecel1,Nr4a1,Sftpc,Adam33,Onecut2
transfac_pub	0.105843	436.406	M1	Nfic,Nfia,Nfi	Masp1,Fat4,Adra1d,Adamts9,Adam33
transfac_pro	0.102822	420.192	M5	Irf8,Irf6,Irf7,I	Twist2,Fat4,Nr4a3,Onecut2,Siglec1,Adamts9
transfac_pro	0.101449	412.821	M6	Mef2a,Mef2c	Spry4,Nr4a3,Nr4a1,Slc25a25
transfac_pub	0.101277	4.119	M7	Stat3,Sfpi1,S	Nr4a1,Onecut2,Nr4a3,Spry4,Slc25a25,Adra1d,Twist2,Fat4,H1f0,Sstr2,Aqp5,Olig2,Lmbr1l,Adamts9
yetfasco-124	0.0964364	385.919	M8	Prkaa2,Prkaa	Nr4a3,Cbr3,Fam89a,Spry4,Adra1d,Fkbp5
transfac_pro	0.0962991	385.182	M9	Lef1,Tcf7l2,P	Ntrk1,Spry4,Nr4a1,Adam33,Adamts9,Fkbp5,Nr4a3
encode-UW.I	0.0962304	384.814	M10	Ctcf,Tcf3,Ctc	Adra1d,Nr4a1,Prph,Siglec1,Spry4,Onecut2,Ecel1
transfac_pub	0.0957841	382.418	M7	Stat6,Stat3,S	Onecut2,Spry4,Nr4a3,Adra1d,Slc25a25,Nr4a1,Sstr2
hdpi-SSBP3	0.0936556	370.994	M11	Ssbp3,Stat1,I	Adra1d,Onecut2,Nr4a3,Adamts9,H1f0,Spry4
jaspar-PF003	0.0922823	363.624	M6	Mef2a,Mef2c	Nr4a3,Ecel1,Nr4a1,Znhit6,Slc25a25,Olig2
encode-UW.I	0.0920077	36.215	M1	Nfic,Nfia,Nfi	Masp1,Aqp5,Nr4a1,Sftpc,Slc25a25,Adra1d,Adam33,Ecel1,Onecut2,Fat4,Prph,Ntrk1,Bspry,Spry4,Fkbp5
homer-M000	0.0911837	357.728	M10	Runx1,Ets1,E	Slc25a25,Nr4a3,Crybb3,Nr4a1,Ccl1,Adamts9
yetfasco-111	0.0909434	356.438	M5	Prkaa2,Prkaa	Onecut2,Fat4,Nr4a3,Spry4,Adamts9
tfdimers-MD	0.0908748	356.069	M1	Pparg,Rxrb,N	Fat4,Ecel1,Adamts9,Spry4,H1f0,Twist2,Ccl1,Ntrk1,Slc7a1,Prph,Nr4a1,Nr4a3,Sftpc,Onecut2,Aqp5,Siglec1,Unc13d,Fam89a,Sstr3
yetfasco-126	0.0907031	355.148	M12	Foxp1,Zfp105	Prph,Fkbp5,Onecut2,Spry4,Slc25a25,Mthfd2,Fat4
transfac_pro	0.0906001	354.595	M2	Pax7,Pax3,Cc	Ecel1,Spry4,Onecut2,Ntrk1,Twist2
transfac_pro	0.0899821	351.279	M9	Rhox11,Tgif2	Nr4a1,Spry4,Ntrk1,Fat4,Adamts9
transfac_pub	0.0896045	349.252	M7	Stat4,Stat5a,	Spry4,Nr4a3,Sstr2,Adra1d,Onecut2,Fat4,Adamts9
jaspar-PF008	0.0893299	347.778	M7	Gabpa,Etv6,E	Slc25a25,Adamts9,Onecut2,Spry4,Mthfd2,Ecel1,Ntrk1,Nr4a1
taipale-YTGC	0.0891582	346.856	M1	Nfix,Nfia,Nfi	Masp1,Spry4,Slc25a25,Fat4,Bspry
hdpi-AVEN	0.0884716	343.171	M13	Aven	Nr4a1,Adra1d,Spry4,Nr4a3,Onecut2
transfac_pub	0.0883342	342.434	M5	Irf8,Irf6,Irf4,I	Twist2,Siglec1,Onecut2,Fat4,Tpcn2,Spry4
yetfasco-152	0.0875103	338.012	M2		Ecel1,Onecut2,Twist2,H1f0,Sstr2,Adamts9,Vav2
tfdimers-MD	0.0873386	337.091	M9	Pou2f1,Klf4,F	Aqp5,Slc25a25,Nr4a1,Onecut2,Znhit6
transfac_pro	0.0872013	336.354	M6	Cdx2,Hoxb9,F	Spry4,Nr4a3,Nr4a1,Olig2
hdpi-SCMH1	0.0869267	334.879	M11	Scmh1	Adra1d,Nr4a3,Ecel1,Slc25a25,Adamts9
yetfasco-707	0.0869267	334.879	M3		Ntrk1,Olig2,Prph,Spry4,Twist2,Slc7a1,Sec63,Onecut2
homer-M014	0.0861714	330.826	M1	Tbp,Tbpl2,Tb	Nr4a3,Onecut2,Fat4,Slc7a1,Ecel1,Fam89a,Twist2,Tpcn2,Aqp5,Olig2,Adra1d,H1f0
tiffin-TIFDMI	0.0856564	328.062	M12	Foxi1,Irf1	Ecel1,Nr4a3,Lman1,Onecut2,Fkbp5,Olig2
homer-M004	0.0850385	324.745	M14		Nr4a3,Ecel1,Onecut2,Fkbp5,Adamts9,Spry4
transfac_pro	0.0844892	321.797	M14		Nr4a3,Fat4,Twist2,Slc25a25
transfac_pro	0.0843518	32.106	M1	Zfp410,Bcl6b	Fat4,H1f0,Nr4a1,Ntrk1,Spry4,Sftpc,Onecut2
homer-M000	0.0842832	320.691	M12	Foxa1,Foxa2,	Masp1,Nr4a1,Nr4a3
transfac_pro	0.0840772	319.586	M14		Nr4a3,Onecut2,Twist2,Fat4
yetfasco-876	0.0839742	319.033	M1	Mecp2,Stat5	Ecel1,Twist2,Nr4a3
homer-M017	0.0838369	318.296	M3		Ntrk1,Olig2,Prph,Slc7a1,Spry4
yetfasco-218	0.0838025	318.112	M1	Mecp2,Pax3,	Ecel1,Olig2,Nr4a3,Ntrk1,Onecut2
stark-YGYGG	0.0831159	314.427	M15	Runx3,Runx1	Adra1d,Twist2,Nr4a3,Ahsa2,Adamts9,Fat4,Spry4
transfac_pro	0.0829099	313.321	M9	Tcf7l2,Lef1,P	Ntrk1,Spry4,Adamts9,Adam33,Nr4a1,Onecut2,Fkbp5,Sec63,Nr4a3
transfac_pro	0.082292	310.004	M9	Rhox11,Tgif2	Nr4a1,Spry4,Ntrk1
iDMMPMM-(	0.082189	309.452	M9	Cdx2,Hoxb9,H	Nr4a3,Spry4,Onecut2,Twist2,Adamts9
yetfasco-120	0.0821546	309.267	M12	Srf,Foxp1,Tgi	Fkbp5,Spry4,Onecut2,Fat4
transfac_pub	0.0819143	307.978	M16		Ahsa2,Fat4,Nr4a3,Onecut2,Spry4,Adamts9,Sec63
yetfasco-161	0.0814337	305.398	M2	Yy2	H1f0,Ecel1,Spry4,Tyrp1,Onecut2,Olig2,Slc7a1
flyfactorsurv	0.081262	304.477	M16	Bcl6,Bcl6b,Pr	Adra1d,Onecut2,Slc25a25,Nr4a1,Adamts9
homer-M015	0.081056	303.371	M3		Ntrk1,Spry4,Olig2,Prph,Slc7a1,Adamts9,Sec63,Nr4a1
transfac_pub	0.080953	302.818	M6	Arid3c,Arid3a	H1f0,Ntrk1,Ecel1,Onecut2,Nr4a1,Aqp5,Nr4a3
yetfasco-123	0.0808844	30.245	M12	Irf3,Irf6,Irf8,I	Fkbp5,Onecut2,Fat4,Mthfd2,Slc25a25,Spry4,Prph,H1f0
yetfasco-174	0.0807814	301.897	M1	Abt1,Vsx2,Nc	Ecel1,Fat4,Ntrk1
jaspar-MA03	0.0804724	300.239	M4	Klf5,Pura,Zbt	Prph,Nr4a1,Ecel1,Sstr3,H1f0,Ntrk1,Sftpc,Spry4
yetfasco-531	0.0804724	300.239	M4	Klf5,Zbtb14,F	Prph,Nr4a1,Ecel1,Sstr3,H1f0,Ntrk1,Sftpc,Spry4
tfdimers-MD	0.0804381	300.054	M16	Tfap4,Nfe2l1	Prph,H1f0,Nr4a1,Slc25a25

Supplementary Table 8: The results of upstream regulator analysis from down-regulated *core set* genes based on iRegulon.

Motif id	AUC	NES	ClusterCode	Transcriptior	Target genes
homer-M003	0.10972	495.322	M1		Slitrk3,Ndp,Kcna4,Maf,Vegfa,Ahnak,Pipox,Tnfrsf11b
flyfactorsurvey	0.108209	486.559	M2	Rorb,Rorc,Ro	Vegfa,Kcna4,Pipox,Luzp2,Ahnak,Tnfrsf11b,Jph1
elemento-AG	0.103242	457.744	M3		Maf,Slitrk3,Kcna4,Vegfa,Tnfrsf11b,Ndp,Ahnak
encode-UW.M	0.0991459	433.984	M1		Maf,Vegfa,Kcna4,Rgs7bp,Slitrk3,Fam110b,Slc22a7,Jph1,Ndp,Mtus1,Rbm11,Fam49a,Grem2,Nudt6,Klhl24,Pipox,Tnfrsf11b,Nrip1,Cd6,C1galt1,Luzp2
homer-M006	0.0975192	424.547	M1	E2f1,Irf3	Slitrk3,Maf,Ndp,Vegfa,Kcna4,Ahnak
tfdimers-MD0	0.09566	413.762	M4	Nfya,Nfyc,Hnf	Maf,Crb3,Lrrc17,Pipox,Vmo1,Ndp,Vegfa,Tnfrsf11b
homer-M006	0.0945561	407.359	M1		Kcna4,Vegfa,Slitrk3,Ahnak,Maf,Ndp,Pipox
transfac_pro-M	0.0945561	407.359	M1		Kcna4,Vegfa,Slitrk3,Ahnak,Maf,Ndp,Pipox
homer-M002	0.0935684	401.629	M1	E2f1,Irf3,Zfp6	Slitrk3,Ndp,Maf,Vegfa,Kcna4
yetfasco-333	0.0935684	401.629	M5	Elf5	Kcna4,Pipox,Ahnak,Ndp,Nrip1
transfac_publi	0.0934232	400.787	M6	Pax5,Pax4,Pa	Maf,Slc22a7,Pipox,Ndp,Slitrk3,Insig2,Vegfa,Fam110b,Ahnak,Kcna4,Lrrc17,Fam49a
transfac_pro-M	0.092726	396.743	M1		Kcna4,Slitrk3,Ndp,Vegfa,Ahnak,Maf
tfdimers-MD0	0.0919417	392.193	M7	Ep300,Bptf,S	Ndp,Maf,Slitrk3,Kcna4,Grem2,Angptl4
homer-M002	0.0907506	385.284	M3		Slitrk3,Ndp,Kcna4,Vegfa,Ahnak
homer-M004	0.0905473	384.104	M1		Slitrk3,Maf,Ndp,Vegfa
jaspar-SA000	0.0895306	378.206	M1	Pax4,Foxa2,F	Rgs7bp,Insig2,Kcna4,Jph1,Slitrk3,Vegfa,Nrip1,Nudt6,Maf,C1galt1,Vpreb3,Fam49a,Grem2,Tle1,Scn10a,Paqr5,Pipox,Fam110b,Aoc3,Mtus1,Slc22a7
transfac_pro-M	0.0885719	372.645	M8	Mef2a,Mef2c,	Rgs7bp,Fam49a,Luzp2,Slitrk3,Ndp,Klhl24,Mtus1
elemento-AAG	0.0865675	361.018	M9	Nr4a1	Vegfa,Jph1,Klhl24,Slitrk3,Ndp
encode-UW.M	0.0857832	356.468	M6	Nfic,Nfia,Nf1,N	Insig2,Slitrk3,Maf,Fam49a,Vegfa,Gpr62,Pipox,C1galt1,Ndp,Ahnak,Tnfrsf11b,Paqr5,Klhl24,Slc22a3,Trpv1,Mtus1,Kcna4
transfac_pro-M	0.0854927	354.783	M6	Tcf3,Myf6,My	Crb3,Atp4b,Ahnak,Ndp,C1galt1,Slitrk3
encode-UW.M	0.0853184	353.772	M10		Angptl4,Pipox,Ndp,Fam49a,Vegfa
transfac_publi	0.0853184	353.772	M7	Elk1,Ets1,Ets	Fam49a,Slitrk3,Insig2,Maf,Ahnak,Paqr5
tfdimers-MD0	0.0851731	35.293	M4	Tcf4,Crx,Otx2	Tle1,Maf,Fam49a,Slitrk3,Nrip1,Fam110b,Jph1
jaspar-CN003	0.085086	352.424	M11	Pou2f2,Pou2f	Kcna4,Slitrk3,Pipox,Vpreb3,Aoc3
tiffin-TIFDME	0.0850569	352.256	M3		Slitrk3,Ndp,Vegfa,Kcna4,Ahnak
transfac_pro-M	0.0850569	352.256	M1	Nr2f6,Hnf4a,F	Jph1,Maf,Vegfa,Klhl24,Pipox,Paqr5,Slitrk3,Slc22a7,Slc22a3,Angptl4
elemento-AG	0.0848536	351.076	M12	Tgif2,Meis1,T	Maf,Vpreb3,Ahnak,Klhl24,Fam49a,Insig2,Jph1,Atp4b
tfdimers-MD0	0.0843307	348.043	M1	Ep300,Sirt6,P	Kcna4,Slitrk3,Fam49a,Ndp,Vegfa
yetfasco-124	0.0829654	340.123	M13	Foxn4,Foxn1,	Rgs7bp,Ndp,Insig2,Pipox,Fam110b,Kcna4,Slc22a3,Fam49a,Hmgcs2
elemento-CT	0.0822972	336.247	M3		Vegfa,Slitrk3,Kcna4,Ndp
stark-GAGAG	0.0822972	336.247	M3		Vegfa,Slitrk3,Kcna4,Ndp
yetfasco-692	0.0822972	336.247	M4	Nkx6-1,Arid3	Tle1,Crb3,Vegfa,Ahnak,Slitrk3
factorbook-T	0.0812514	330.181	M6	Tcf12,Ascl2,T	Pipox,Ahnak,Atp4b,Slitrk3,Fscn2,Crb3,Ndp,Vegfa,Aoc3,Trpv1
tiffin-TIFDME	0.0810481	329.001	M14		Fam110b,Jph1,Fam49a,Mtus1,Ndp,Paqr5
flyfactorsurvey	0.0806124	326.473	M15	Prdm1,Zfp68	Cd6,Grem2,Scn10a,Maf,Nrip1,Slitrk3,Vegfa,Klhl24,Pipox,Ahnak,Fam110b,Paqr5,Lrrc17,Tnfrsf11b,Luzp2,Fam49a,Jph1,Tle1,Rbm11,Ndp
transfac_pro-M	0.0805252	325.968	M1	Tfap2e,Tcf7l1	Slitrk3,Maf,Ndp,Grem2,Klhl24,Kcna4
factorbook-S	0.0803509	324.957	M16	Pou5f1,Nano	Pipox,Maf,Tle1,Grem2,Rbm11,Lrrc17
yetfasco-1907	0.0803509	324.957	M17	Pparg,Rorb,R	Fam110b,Vegfa,Slitrk3,Jph1,Nrip1,Mtus1,Slc22a7,Ndp,Klhl24
jaspar-PF010	0.079828	321.924	M14	Foxa2,Foxa1,	Insig2,Pipox,Plcd4,Ahnak,Grem2,Ndp,Fam110b,Klhl24
flyfactorsurvey	0.0797409	321.418	M9		Vegfa,Slitrk3,Slc22a7,Jph1,Slc22a3,Klhl24
transfac_publi	0.0796247	320.744	M18	Klf5,Zfp622,K	Ahnak,Maf,Vegfa,Fscn2,Paqr5,Jph1,Cd6,Klhl24,Ndp,Slitrk3,Slc22a7,Mtus1
elemento-CA	0.0795666	320.407	M19		Atp4b,Paqr5,Insig2,Klhl24,Angptl4
elemento-AG	0.0790437	317.374	M3		Slitrk3,Ndp,Kcna4,Vegfa
elemento-AG	0.0783175	313.161	M20	Tcf3,Zeb1	Crb3,Atp4b,Rbm11,Mtus1,Maf,Kcna4,Fscn2,Slitrk3,Ndp,Plcd4,Paqr5,C1galt1,Slc22a7
tfdimers-MD0	0.0783175	313.161	M15	Tcf4,Foxo1,F	Ndp,Rbm11,Klhl24,Tle1,Mtus1,Fam110b
tfdimers-MD0	0.0779979	311.307	M4	Myb,Pax4,Hn	Vegfa,Vmo1,Crb3,Fam49a,Kcna4
yetfasco-756	0.0776493	309.285	M5		Ndp,Ahnak,Kcna4,Mtus1,Fam49a,Nrip1,Slc22a3,Pipox
swissregulon-	0.0773298	307.432	M21	Spz1	Slitrk3,Vegfa,Ndp,Paqr5,C1galt1
transfac_pro-M	0.0773298	307.432	M6	Tcf3,Myod1,M	Atp4b,Crb3,Ahnak,Fscn2,Pipox,Ndp,Slitrk3,Cd6,Grem2,Fam49a,Vpreb3,Jph1,Rgs7bp,Paqr5
tfdimers-MD0	0.0771264	306.252	M12	Yy1,Mycs,Ne	Fam49a,Ahnak,Ndp,Jph1,Gpr62,Pipox,Slitrk3
yetfasco-142	0.0771264	306.252	M8	Mef2b,23100	Rgs7bp,Fam49a,Luzp2,Slitrk3,Klhl24,Mtus1
hdpi-C19orf2	0.0770393	305.746	M15	2310011J03	Nrip1,Maf,Klhl24,Luzp2,Tle1,Mtus1
homer-M000	0.0770393	305.746	M16	Cebpb,Atf4,A	Tle1,Fam110b,Ndp,Slitrk3,Pipox,Vegfa,Ahnak
transfac_pro-M	0.0770393	305.746	M1	Irf7,Irf5,Irf2,Ir	Slitrk3,Nrip1,Jph1,Tle1,Vegfa,Pipox
taipale-NRTT	0.0768359	304.567	M4	Hnf1a,Hnf1b,	Crb3,Vmo1,Vegfa,Kcna4,Klhl24,Fam49a,Ndp
flyfactorsurvey	0.076284	301.365	M22	Zfp513,Foxj3,	Maf,Pipox,Slitrk3,Klhl24
tfdimers-MD0	0.0760516	300.017	M4	Hnf1b,Runx1,	Crb3,Maf,Tle1,Slitrk3
